# Diversity, Phylogeny and Expression Patterns of Pou and Six Homeodomain Transcription Factors in Hydrozoan Jellyfish *Craspedacusta sowerbyi*

**DOI:** 10.1371/journal.pone.0036420

**Published:** 2012-04-30

**Authors:** Miluse Hroudova, Petr Vojta, Hynek Strnad, Zdenek Krejcik, Jakub Ridl, Jan Paces, Cestmir Vlcek, Vaclav Paces

**Affiliations:** 1 Department of Genomics and Bioinformatics, Institute of Molecular Genetics, Academy of Sciences of the Czech Republic, Prague, Czech Republic; 2 Department of Biochemistry and Microbiology, Faculty of Food and Biochemical Technology, Institute of Chemical Technology Prague, Prague, Czech Republic; Trinity College, Ireland

## Abstract

Formation of all metazoan bodies is controlled by a group of selector genes including homeobox genes, highly conserved across the entire animal kingdom. The homeobox genes from *Pou* and *Six* classes are key members of the regulation cascades determining development of sensory organs, nervous system, gonads and muscles. Besides using common bilaterian models, more attention has recently been targeted at the identification and characterization of these genes within the basal metazoan phyla. Cnidaria as a diploblastic sister group to bilateria with simple and yet specialized organs are suitable models for studies on the sensory organ origin and the associated role of homeobox genes. In this work, *Pou* and *Six* homeobox genes, together with a broad range of other sensory-specific transcription factors, were identified in the transcriptome of hydrozoan jellyfish *Craspedacusta sowerbyi*. Phylogenetic analyses of Pou and Six proteins revealed cnidarian-specific sequence motifs and contributed to the classification of individual factors. The majority of the *Craspedacusta sowerbyi Pou* and *Six* homeobox genes are predominantly expressed in statocysts, manubrium and nerve ring, the tissues with sensory and nervous activities. The described diversity and expression patterns of Pou and Six factors in hydrozoan jellyfish highlight their evolutionarily conserved functions. This study extends the knowledge of the cnidarian genome complexity and shows that the transcriptome of hydrozoan jellyfish is generally rich in homeodomain transcription factors employed in the regulation of sensory and nervous functions.

## Introduction

The proper development and function of metazoan organs depends on sophisticated network of regulatory elements [1]–[4]. Namely transcription factors and their binding partners are key players in ontogenetic processes; their dysfunctions lead to severe disorders in humans [5], [6]. These phylogenetically ancient factors could be traced back before the origin of metazoans [7]–[14]. In the area of sensory organ evolution and development research, attention has been paid to several classes of homeobox genes encoding homeodomain transcription factors Six, Pou, Paired, Lim, Dlx, and several others [15]–[17]. Members of these groups make the decisions on the cell fate and differentiation during the embryonic period of sensory organs development and organize the structure and function of adult tissues.

Four major types of sensory receptors – photoreceptors, mechanoreceptors, chemoreceptors and thermoreceptors – have been described together with a number of organ types formed by them. Development and functions of visual organs are driven by sophisticated mechanisms consisting of several signaling pathways, which include a number of transcription factors, signaling molecules and receptors [17]–[22]. Photoreceptors constitute visual and light-responding sensory organs with various degree of complexity ranging from pigment spots and cups to ocelli or lens-equipped eyes [17], [23]. Development of visual organs is driven by a wide range of homeobox genes mainly from the *Six*, *Paired*, *Pou*, *Dlx* and *Lim* classes, by bHLH factors from the groups Atoh, Olf (EBF), Hes, NeuroD, Msc, and other DNA-dependent factors such as Dach, Eya or Lozenge [15], [18], [19], [22], [24]–[38]. Mechanoreceptor cells are present on the surface of animal bodies to detect the mechanical stimuli and changes of the pressure. They are parts of auditory and vestibular organs, ears, tactors, balance and gravity sensing systems, and they mediate the response to the sound fluctuation and to the changes of body position and acceleration. The regulation of the auditory and vestibular organ development is mediated mainly by transcription factors from the Six, Pou and Paired homeobox class, by bHLH factors from the groups Atoh, Neurog, Hes and Barlh, by zinc finger proteins Gata3 and Gfi1, and other DNA-dependent factors such as Eya, Ntf or Sox [16], [19], [24], [25], [28], [29], [32], [39]–[43]. Olfactory and gustatory systems are built of chemoreceptor cells and form taste buds, olfactory mucosa, nose and other nasal structures. Development of olfactory organs is driven by a complicated mechanism distinguishing the developmental pathways leading to several types of receptors, promotive cells, at least three groups of olfactory neurons and a wide range of projection motor neurons [44]. The typical homeobox gene class regulating development and function of chemosensory-based organs is the *Lim* class, but also *Pou*, *Six* and *Paired* members are employed. Of bHLH factors the Olf group should be mentioned as regulatory proteins, as well as Eya1 and Eya2 DNA-dependent factors [16], [24], [28], [29], [31], [44]–[52].The thermoreceptor organ development and its regulation is poorly understood. Hobert and co-workers report the role of several *Lim* homeobox genes in the thermoregulatory network [46], [53], [54]. Especially homeodomain proteins Ttx1 (Otx), Ttx3 (Lim class), Lin11 (Lim class) are also often mentioned by other authors to be involved in the thermoregulatory pathways and thermo-sensing [55]–[59]. The *Caenorhabditis elegans Unc-86* gene homologous to *Pou4* has been described as a gene required for thermotaxis [57]. Temperature sensing is mediated by olfactory neurons in *Caenorhabditis elegans* which are sensitive to temperature [58].

All of the above-mentioned mainly mammalian genes have their orthologs in lower organisms reaching as far as to diploblastic bilaterian sister group Cnidaria. In this ancient phylum, we can find complex genetic regulation cascades for sensory organ establishment, development and function. This implies that most of metazoans including Cnidaria share, from this point of view, a common regulatory strategy [18], [20], [60], [61]. Therefore, it is important to study the processes of sensory organ origin, development and evolution at such a basal metazoan clade. The role of evolutionarily conserved transcription factors, including members of the Six and Pou gene families, in cnidarian neurogenesis has been extensively discussed in several studies [8], [62].

**Figure 1:**
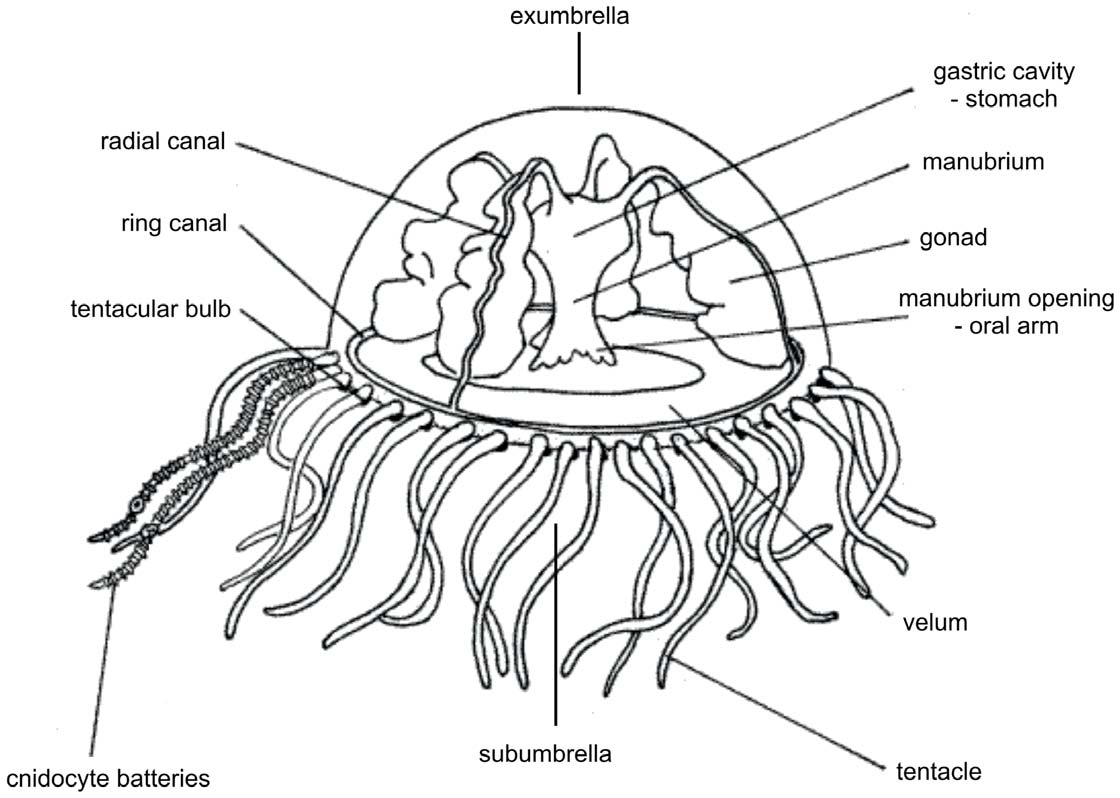
*Craspedacusta sowerbyi* body plan scheme. Radial symmetric body plan of *Craspedacusta sowerbyi* is divided in subumbrella and exumbrella regions from the side projection. The most distinct features are four gonads and the presence of up to two hundreds of tentacles. Particular organs are described in the scheme. Body size is up to 2.5 cm in diameter.

The freshwater jellyfish *Craspedacusta sowerbyi* is a member of class Hydrozoa, which includes a family with the life cycle reduced to asexual stage of polyps (Hydridae), or family Cladonematidae, cnidarians with the life cycle alternating asexual polypoid and sexual medusoid stage, equipped with primitive eyes (ocelli). Our model organism *Craspedacusta sowerbyi* (class Hydrozoa, order Limnomedusae, family Olindiidae) alternates both sessile and swimming form during its life. It is eyeless and manifests no morphological features of another sensory organ except for the statocysts located in tentacular bulbs at the bases of tentacles (Figure 1). This genus is interesting and suitable for research of regulatory genes thanks to its body simplicity, the predaceous way of life and frequent worldwide occurrence in freshwater ecosystems. Homeobox genes are subjects of special interest in this study because of their high structural and functional conservation across the animal kingdom and their clearly proven ancient origin before the split of uni- and multicellular organisms.

From the list of the above-mentioned factors employed in the regulation of sensory organ development it is obvious that several homeobox gene classes are common for visual, auditory and olfactory systems. *Pou* and *Six* homeobox genes are typical representatives of these regulatory genes, and their identification and description in the genome and transcriptome of suitable model organisms can answer the questions about the origin and development of sensory organs [29], [30], [34], [36], [40], [52], [63]–[69]. Phylogenetic analyses of protein products of these genes in *Craspedacusta sowerbyi* have been compiled and revealed some typical features of the genomes and transcriptomes of hydrozoans. Further, morphological areas with significant expression of *Pou* and *Six* genes were identified by whole-mount *in situ* hybridization with the intention to describe the association of their expression with neural structures and sensory receptors localization.

## Materials and Methods

### Animals

The clonal female population of adult jellyfish *Craspedacusta sowerbyi* originates from the locality Kojetice, Czech Republic. No specific permits were required for the described field study. The location is not privately-owned or protected in any way. Our field study did not involve endangered or protected species. The animals were collected in late summer at the time of their maximal occurrence, transferred into drinking water to remove heterogeneous organisms from their surface and starved for two days to diminish the microbiological contamination of the digestive cavity. Two days of starving are sufficient for cleaning while no autodigestion starts. Animals were wiped dry and frozen at −80°C for subsequent DNA and RNA extraction. For the whole-mount *in situ* hybridization experiments, 4% paraformaldehyde fixation was performed for at least one hour. The animals were washed in PBSt buffer (137 mM NaCl; 2.7 mM KCl; 100 mM Na_2_HPO_4_; 2 mM KH_2_PO_4_, pH 7.4; 0.1% Tween 20), transferred to methanol and stored at −20°C up to several months.

### DNA extraction

Chromosomal DNA was extracted after disruption of the jellyfish tissues by SDS (1%) and proteinase K (1.25 mg/ml) at 55°C overnight. After osmotic lysis (1.7 M NaCl) DNA was precipitated with isopropanol (70%) and washed twice with ethanol (70%). DNA was dissolved in TE buffer (10 mM Tris-HCl pH 7.5; 1 mM EDTA) to the required concentration and analyzed spectrophotometrically and by agarose electrophoresis.

### RNA extraction

Jellyfish tissues were disrupted with RNase-free proteinase K as described above and total RNA was isolated from homogenized tissues with RNeasy Mini Kit (Qiagen, USA). Final concentration was estimated spectrophotometrically and the integrity of RNA was assessed by Agilent bioanalyzer 2100 Nano-chip assay.

### cDNA synthesis and RACE

*Craspedacusta sowerbyi* full-length cDNA was prepared with SMART PCR cDNA Synthesis Kit (Clontech, USA). Direct cDNA sequencing was performed (see the Transcriptome analysis paragraph). To extend the coding sequences of *Pou* and *Six* genes both 3′ and 5′ RACEs were performed for each transcript with the SMART RACE cDNA Amplification Kit (Clontech, USA). *Pou* and *Six* sequences were amplified using primers derived from the sequences obtained from transcriptome analysis (Table S1).

### Transcriptome analysis

Full-length cDNA from one adult jellyfish prepared by SMART PCR cDNA Synthesis protocol was normalized using Trimmer cDNA normalization Kit (Evrogen, Russia) to decrease the number of copies of abundant genes and equalize cDNA before the sequencing procedure. cDNA in this form was sent to the high-throughput sequencing center (Eurofins Medigenomix GmbH, Germany) and processed on the GS FLX platform. Basic assembly (Newbler) was done in the service laboratory. The contigs were further screened for particular genes in the transcriptome of *Craspedacusta sowerbyi* in our laboratory. UniRef and Non-redundant protein databases were searched for putative sequence homologs using BlastX and Fasta.

### Phylogenetic analyses

We used appropriate Six and Pou protein reference sequences (NCBI database) from a wide range of multicellular organisms from sponges to humans to construct phylogenetic trees based on the conserved class-specific domains and homeodomains. PhyML 3.0 [70] was used for phylogenetic analyses with subsequent visualization in NJPlot software. For IDs of the used NCBI reference sequences see Text S1.

### Whole-mount *in situ* hybridization

mRNA probes for the *Craspedacusta sowerbyi* genes *csPou6*, *csPou4f1*, *csPou4f2*, *csPou4f3*, *csSix1/2A*, *csSix1/2B*, *csSix3/6A*, *csSix3/6B*, *csSix-X* and *csSix4/5B* were prepared following the instructions of the DIG RNA Labeling Kit manufacturer (Roche, USA). PCR conditions of DNA templates for particular RNA probe synthesis were: 3 min at 94°C, 35 cycles of 30 sec at 94°C, 30 sec at annealing temperature (listed for particular probes in Table S2), and 2 min at 72°C, followed by 7 min at 72°C. Primer sequences and product lengths are also listed in Table S2. Sense probes of all genes were also prepared and used as negative controls.

Material stored in methanol was rehydrated stepwise from 100% methanol to 100% PBSt. Permeabilization was performed by proteinase K treatment (10 µg/ml, 37°C, 10 min). After washing in PBSt (1×) with glycine (2 mg/ml) animals were washed twice with hybridization buffer (5×SSC buffer, pH 7.4; 50% formamide; 0.1% Tween 20) and prehybridization was carried out at 60°C for two hours after addition of DMSO (final concentration 0.1%), heparin (final concentration 50 µg/ml) and tRNA (final concentration 50 µg/ml). Hybridization was carried out at 60°C overnight in a shaker by adding the DIG-labeled mRNA probe at final concentration 0.1 ng/µl. Subsequently, stepwise 30 min long washes from hybridization cocktail to 100% PBSt were accomplished followed by two hours of blocking with 10% sheep serum. Anti-DIG alkaline phosphatase was applied at final dilution 1/2000 at 4°C overnight. Material was washed with MABT (100 mM maleic acid, 150 mM NaCl, 0.1% Tween 20, pH 7.5, three times, 20 min, room temperature), with washing buffer 1 (100 mM Tris-HCl, 100 mM NaCl pH 8.5, once, 5 min, room temperature) and with washing buffer 2 (100 mM Tris-HCl, 100 mM NaCl, 50 mM MgCl_2_, pH 8.5, twice, 5 min, room temperature). Mixture NBT/BCIP (100–225 µg/ml NBT and 175 µg/ml BCIP or 20 µl NBT/BCIP (Roche, USA) per 1 ml of final staining cocktail) was used for colorimetric visualization of the areas of gene expression (several hours in the dark).

## Results

### *Craspedacusta sowerbyi* transcriptome sequencing and analysis

Sequencing of *Craspedacusta sowerbyi* cDNA produced 39.55 million bases (189,558 reads with average length 208 bp) with coverage 1.9. A total number of 14,081 contigs was generated by the assembly. Contigs include 4.3 million bases (102,310 reads). This sequence resource (14,081 queries) was used for analyses with the BlastX and Fasta tools to find similarities at the protein level. Uniref database search identified 10,993 contigs with hits and 9,479 contigs with hits were identified in the case of NCBI database (Nr) (E-value<10 in both cases). Thirty-three contigs containing sequences with hits (identity >30%) to homeodomain proteins were found in the UniRef or Nr databases (Table S3). Assembled sequencing data in contigs are available at the webpage http://craspe.img.cas.cz. This webpage enables searches for particular sequence similarities of user interest.

Six contigs were found with significant similarity to Pou and Six proteins (2040, 7026, 5641, 7065, 388 and 8120). There are three different Six sequences (2040, 7065 and 8120) and two different Pou sequences (388 and the same protein similarity in 7026 and 5641). These sequences were used to design primers for 3′ and 5′ RACEs to obtain complete protein-coding sequences.

Generally, a relatively high number of nucleotide sequences similar to genes encoding transcription factors, bHLH and zinc-finger proteins and other DNA-dependent regulatory proteins or their binding partners involved in sensory organ developmental processes and regulation cascades were found in the *Craspedacusta sowerbyi* transcriptome. For example, members of Pax-Six-Eya (Pax, Six, Eya, Dach) and Notch (Notch1, Delta1, Jagged1, Jagged2, Lfng, Numb) regulation pathways are present as well as visual organ development-specific factors from groups Atoh, Dlx, Vsx, Crx, Lhx, SOHo1, Isl1, Gfi1, auditory organ developmental factors Gata3, Hes5, Otx1, Hmx3, Pax3, Sox2, Sox10, olfactory organ developmental factors Olf1, Lin11, Mec4, Mec7, Idx1 and other less specific factors and receptors (Math3, Math5, Fox1, Msx1, Irx, Neurog1, TrkA, Ngf). These similarities were found by nucleotide Fasta search for the homologs of known regulators of sensory organ development across the metazoan species mainly from human (Table S4). Some of the similarities are too low to predict a protein sequence long enough to identify proteins by BlastX because of the short length of contigs, and that is why some of the homeobox transcription factors identified at the nucleotide level are not mentioned in Table S3.

### *Craspedacusta sowerbyi* Six and Pou proteins

Sequence analyses of 3′ and 5′ RACE products revealed more Six and Pou transcripts than estimated from the homology search. Each RACE amplification of the predicted *Six1/2*, *Six3/6* and *Six4/5* genes produced two different sequences. It was obvious that there are six different Six transcription factors designated csSix1/2A [GenBank:JF819719], csSix1/2B [GenBank:JF819720], csSix3/6A [GenBank:JF819721], csSix3/6B [GenBank:JF819722], csSix4/5B [GenBank:JF819723] and csSix-X [GenBank:JF819724] according to the best BlastX hits to the known homeodomain proteins from other organisms. csSix-X was designated X because of its unclear relationship to any Six subclass. Similarly, *Pou4* RACE product sequencing generated three different *Pou4* sequences. These transcription factors were designated according to their best BlastX hits and phylogenetically closest relatives csPou4f1 [GenBank:JF819715], csPou4f2 [GenBank:JF819716] and csPou4f3 [GenBank:JF819717] using the vertebrate nomenclature. Sequencing of RACE products based on primers designed on contigs 7026 and 5641 resulted in identical sequences with the best hits to Pou6 class transcription factors. In our case the factor was designated csPou6 [GenBank:JF819718]. Altogether four transcription factors of the Pou class and six members of the Six class were identified in *Craspedacusta sowerbyi* transcriptome (Text S2). Degenerate primers were designed to all *Six* and *Pou* subclasses (based on *Six* and *Pou* genomic sequences from the species *Hydra magnipapillata*, *Nematostella vectensis* and *Homo sapiens*) to estimate the total number of *Six* and *Pou* genes in the genome of *Craspedacusta*. No additional subclass or member of Six and Pou proteins was detected by PCR using genomic DNA as a template (unpublished data).

These transcripts retain all typical features and domain structure of the appropriate homeodomain protein class such as the conserved Pou (PD) and Six (SD) specific domains and homeobox domain (HD) and variable linker (VL) in the Pou proteins (Figure S1). There are some exceptions in csSix-X mentioned further. Pou4 proteins differ from each other within the conserved region of PD-VL-HD in only two amino acid positions in the variable linker.

### Phylogenetic analyses of Pou and Six conserved regions

Homeodomain amino acid sequence alignment and phylogenetic tree were constructed from the HD regions of representative Pou proteins (Figures S2 and S3). Proteins csPou4f1, csPou4f2 and csPou4f3 are defined by the presence of typical Pou4 subclass motifs such as five basic amino acids KKRKR at the start of the homeodomain followed by TSI (position 1–8 of HD), conserved motif SLEAXF inside helix1 (position 15–20 of HD), Pro between helix1 and helix2 at position 26 of HD, motif IXXXAXXL in helix2 (position 31–38 of HD) and highly conserved helix3 sequence KNVVRVWFCNQRQKXKR with basic amino acid terminus (position 42–58 of HD) (Figure S2). Gly at position 9, Met at position 22, Asp at position 29 and Lys at position 56 are specific for Pou4 factors in phylum Cnidaria (Figure S2). These amino acids have the same polarity and acid-base character as consensual amino acids at the particular positions. The Pou4 sequences show closest relationship to other cnidarian Pou4 subclass members. The csPou6 protein is characterized by Phe at position 8 and Leu at position 56 typical of the Pou6 subclass generally and cnidarian-specific Thr at position 44 in helix3 of the homeodomain (Figure S2). The closest relationship to csPou6 is held by the Pou6 sequence from hydrozoan jellyfish *Eleutheria dichotoma* and other two cnidarian species, namely *Nematostella vectensis* and *Condylactis gigantea* belonging to class Anthozoa.

The result of phylogenetic analysis (Figure S3) of the Pou HD region led to unambiguous classification of csPou6 to the Pou6 subclass with the highest similarity to Pou6 from *Eleutheria dichotoma*. Pou6 and Pou4 homeodomain sequences from cnidarians form separate branches from bilaterian proteins. Division of Pou4 factors also clearly corresponds to taxonomic classification. The Pou6 subclass can be divided into two branches of hydrozoan (*Craspedacusta sowerbyi* and *Eleutheria dichotoma*) and anthozoan (*Nematostella vectensis*) sequences. *Schistosoma mansoni* (phylum *Platyhelminthes*) protein Pou4 forms a branch standing separately from either other bilaterian species or cnidarians. An interesting feature of *Craspedacusta* Pou4 sequences is their high similarity to each other. They do not separate into the Pou4f1, Pou4f2 and Pou4f3 bilaterian clusters. It seems that all of them belong to the Pou4f2 group with Pou4f2 from *Nematostella vectensis* and Pou from *Hydra magnipapillata*. The criterion for classification of particular csPou4 factors should be other than the phylogenetic tree of the highly conserved homeodomain.

The sequence alignment of the Pou4 variable linker region, connecting the Pou-specific domain with the homeodomain, shows differences in two amino acids when comparing csPou4f1, csPou4f2 and csPou4f3 (Figure S4). Gly in position 1 indicates that csPou4f1 belongs to the Pou4f1 transcription factor subclass described in vertebrates. csPou4f2 and csPou4f3 differ from each other at position 5 in Ile and Met. While Met is common in other two *Craspedacusta sowerbyi* Pou4 proteins and also in cnidarians *Hydra* and *Aurelia*, Ile is unique at this position across the species. Other differences are found upstream of the Pou-specific domain in the non-conserved regions. The distinction to Pou4f2 and Pou4f3 is finally based on the result of BlastX search against the Non-redundant database that shows higher max score for the similarity of Pou class 4 homeobox 3-like protein from vertebrate *Oryctolagus cuniculus* with csPou4f3 compared to csPou4f2. The best hits with appropriate cnidarian sequences cannot answer this question because of the absence of extensional subclassification of these sequences to f1, f2 and f3. Particular cnidarian sequences of the variable linker region cluster together and point at close relationship of these species and their unique position in animal phylogenesis.

**Figure 2:**
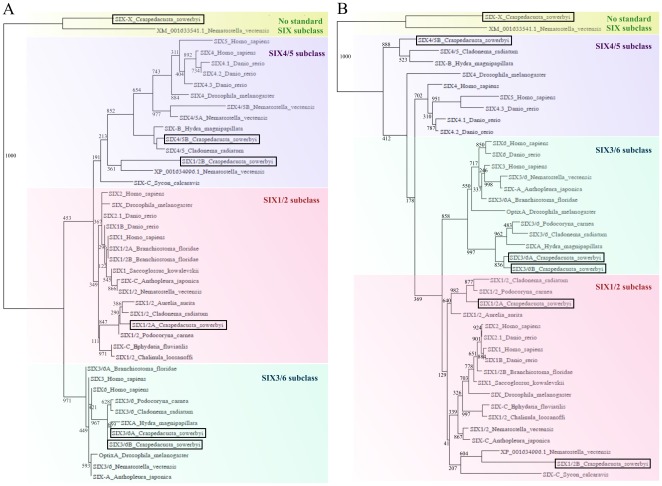
Comparison of phylogenetic trees of HD (A) and SD (B) regions of Six class transcription factors. The phylogenetic trees were calculated by the maximum likelihood method (WAG protein substitution model, bootstrap 1000) and processed by NJ Plot software. Phylogenetically representative sequences from the following metazoan animals were selected for comparison: sponges (*Chalinula loosanoffi, Ephydatia fluviatilis, Sycon calcaravis*), cnidarians (*Nematostella vectensis*, *Anthopleura japonica*, *Podocoryne carnea*, *Hydra magnipapillata*, *Cladonema radiatum*, *Aurelia aurita*), invertebrates (*Saccoglossus kowalevskii*, *Drosophila melanogaster*, *Branchistoma floridae*) and vertebrates (*Danio rerio* and *Homo sapiens*). For the list of ID numbers of reference sequences see Text S1.

Six protein sequences also form clusters dependent on taxonomic units in the alignment. Cnidarian-specific branches are shown in the phylogenetic trees constructed for SD and HD regions (Figure 2) as well as in amino acid alignment of HD (Figure S5). Classification of csSix-X into any of the three Six subclasses is difficult. The ETSY (typical of Six1/2 subclass), ETVY (typical of Six4/5 subclass) and QKTH (typical of Six3/6 subclass) diagnostic motifs located at the start of the homeodomain are not present in csSix-X. Closely related factor XP_001633591.1 (*Nematostella vectensis*) displays the QKST sequence in this position. This is similar to the Six3/6 motif, but the RRTN sequence of csSix-X does not match any subclass motif.

Phylogenetic analyses of HD (Figure 2A) and SD (Figure 2B) conserved regions of Six proteins resulted in two different trees. Differences are mainly in the case of csSix1/2B, which clusters together with *Nematostella vectensis* protein XP_001634996.1 and Six-C protein from sponge *Sycon calcaravis* with subclass Six1/2 when the SD region is used while this triplet clusters with subclass Six4/5 when the HD region is used. Closely related protein sequences csSix-X and *Nematostella vectensis* XP_001633591.1 do not match any Six subclass in both cases. Classification of these proteins in basal metazoans would not be convincing due to their ancient character. However, it is supported by the results of tissue-specific expression experiments showing patterns typical of *Six* gene expression in higher organisms and is also determined by protein similarities found by BlastX.

### Tissue-specific expression patterns of *Pou* and *Six* genes

**Figure 3:**
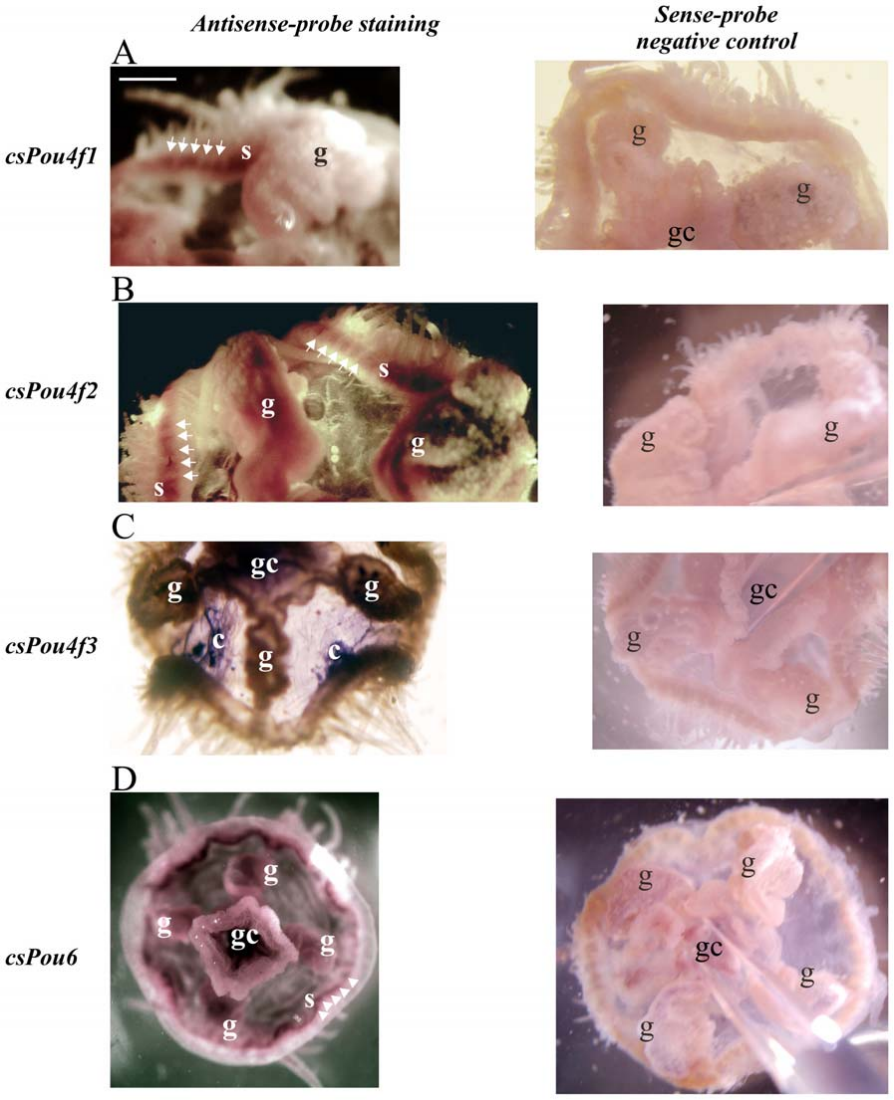
Expression of *Craspedacusta sowerbyi Pou* genes. Violet or blue coloring (depending on light intensity and conditions of the documentation process) shows sites of expression of particular genes. (A) *csPou4f1* expression in statocysts (s), (B) *csPou4f2* expression in statocysts and gonads (g), (C) *csPou4f3* expression in the bell quadrant centers (c), (D) *csPou6* expression in gonads, statocysts and gastric cavity (gc). Appropriate sense-probe negative controls are shown in the right column. A pipette tip was used for manipulation during the documentation and is present in some pictures. Scale bar (A, white) is in (A), (B) and appropriate negative controls 70 µm and in (C), (D) and appropriate negative controls 100 µm.

The method of whole-mount *in situ* hybridization with RNA probes was selected to describe the tissue-specific transcription profile of homeobox genes. The simple structure and composition of jellyfish body enable effective and uniform penetration of the probe to the tissues and direct colorimetric visualization of the bound probe. The high number of transcribed *Pou* and *Six* genes in adult jellyfish requires description of the expression pattern of these genes to obtain a general idea about their function and relative differences. Different expression profiles of *Pou4* genes offer the hypothesis on multiplication and specialization of the ancestral *Pou4* gene in the hydrozoan class. *csPou4f1* and *csPou4f2* expression colocalizes at the bell margin in statocysts between the tentacles. Besides that, *csPou4f2* is expressed in the gonads (Figure 3A and 3B). Probe *csPou4f3-*specific staining is dominant in the regions close to the center of bell quadrants and also in the region of gastric cavity, which probably represents trapped background (Figure 3C). *csPou6* is expressed mainly in statocysts and to a lesser extent in the gonads (Figure 3D).

**Figure 4:**
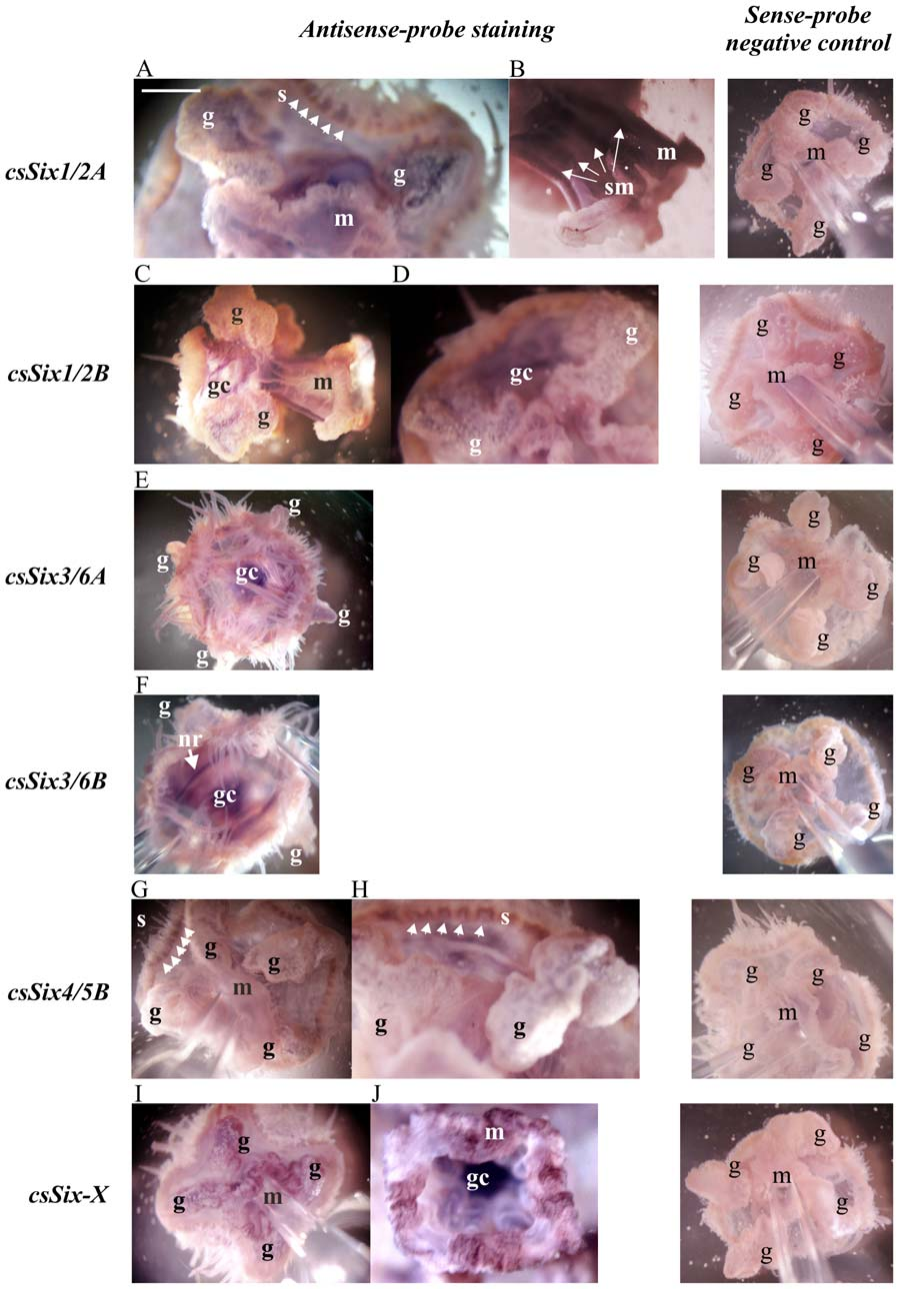
Expression analysis of *Craspedacusta sowerbyi Six* genes. Violet or dark red coloring (depending on light intensity and conditions of the documentation process) show sites of expression of particular genes. (A) *csSix1/2A* expression in gonads (g), statocysts (s) and gastric cavity (gc), (B) four smooth muscle strands (sm) along with the tubular part of manubrium (m), (C, D) *csSix1/2B* expression at low level in gonads, at high level in gastric cavity except for manubrium opening, (E) *csSix3/6A* expression in gastric cavity, (F) expression of *csSix3/6B* in the nerve ring (nr) and gastric cavity except for manubrium, (G) *csSix4/5B* expression specifically in the statocysts, (H) detail of these structures shows a narrow strip of colored tissue around the tentacle bulb, (I, J) expression of *csSix-X* in gonads, gastric cavity and at the manubrium opening (frilly lips, mouth) in four zones concurring to four muscle strands in the manubrium, the site of *csSix1/2A* expression. Appropriate sense-probe negative controls are shown in the right column. A pipette tip was used for manipulation during the documentation and is present in some pictures. Scale bar (in A, white) is in (A), (B) (D), (H), (J) 70 µm and in (C), (E), (F), (G), (I) and all negative controls 160 µm.

The expression patterns of *csSix1/2A* and *csSix4/5B* (Figure 4A, 4G and 4H) colocalize with *csPou4f1*, *csPou4f2* and *csPou6* in statocysts, and *csSix1/2A* expression is also visible in four strands of smooth muscles in manubrium (Figure 4B). These strands lead to manubrium opening, where the expression of *csSix-X* takes place at four appropriate regions equipped with mechanoreceptors as well as in gonads (Figure 4I and 4J). *In situ* experiments with *csSix1/2B*, *csSix3/6A* and *csSix3/6B* probes resulted in staining of gastric cavity with no specific site of increased expression (Figure 4C–F). Here it is problematic to distinguish the background and real expression of these genes in gastric epithelium. *csPou4f2*, *csPou6*, *csSix1/2A* and *csSix-X* are expressed in gonads, but no significant expression was observed in free oocytes (data not shown). Expression of *csSix3/6B* is localized predominantly in the circular structure in jellyfish bell (Figure 4F) that corresponds to the nerve ring described in other cnidarians [71]–[73].

## Discussion

Shotgun sequencing of *Craspedacusta sowerbyi* full-length cDNA resulted in more than four million bases assembled in more than fourteen thousand contigs (Text S3 or webpage http://craspe.img.cas.cz). Although the GS FLX platform produced short reads with lower accuracy than offered today by more advanced techniques, the obtained data quality is sufficient to perform searches for protein similarities. We focused on homeodomain proteins as regulators of sensory organ development and function and found a surprisingly high number of these factors present in the transcriptome of adult eyeless hydrozoan jellyfish in contrast to several studies [28], [34], [74]–[84]. Rather than development, in adult stage of *Craspedacusta sowerbyi* these factors control the maintenance, operation and rearrangement of sensory neurons and organs in the diffuse nerve net. This tight control is necessary mainly due to the predaceous way of jellyfish life demanding precise movement coordination and rapid reactions to exogenous stimuli. The presence of the representatives of all *Six* subclasses as well as existence of three different *Pou4* genes and one *Pou6* gene compared to other radiata (sponges, placozoans and ctenophores) confirms the results of several studies on the unexpected complexity of cnidarian genomes [11], [85]–[88]. Three homeobox genes of class *Six* were described in cnidarians, mostly one member of each *Six1/2*, *Six3/6* and *Six4/5* subclass [16], [34]. Recent genome study on *Nematostella vectensis* (six *Six* genes and later discussed hybrid *Six* gene XM_001633541.1, Text S1) together with this study show that cnidarian *Six* genes have undergone evolutionary divergence including duplication and have reached the same number and similar functions as in vertebrates [24], [76], [89]. It seems that the generally accepted opinion based on *Drosophila melanogaster* studies that Protostomes have only one gene of each *Six* subclass is not correct [28], [29], [90], [91].

Phylogenetic analyses of Pou and Six homeodomain protein classes show a tendency of cnidarian factors to group separately and form clusters based on specific sequence motifs typical of this phylum. This suggests that in their evolution cnidarians followed a separate way leading to a high degree of specialization of the entire classes of homeobox transcription factors and in the case of *Pou4* subclass even to generation of three new genes from one ancestor Pou4 gene. There are only a few amino acid differences between csPou4f1, csPou4f2 and csPou4f3 in the variable linker connecting the PD and HD domains. The close relationship of these proteins in structure and function indicates recent evolutionary amplification of one ancestor gene to three new individuals in the hydrozoan class. This is supported by the fact that only one *Pou4* gene can be found in basal metazoan clades Placozoa [12], [74], [92], Porifera [81], [93], [94] and Ctenophora [92], [94] as well as in other cnidarians [8], [62], [79], [82]. This study, to the best of our knowledge, is the first to deal with extensional classification of Pou4 transcription factors in a cnidarian.

In Cnidaria, the medusae of Scyphozoa and its sister-group Cubozoa uniquely possess rhopalia at their bell margin. Rhopalia receive sensory inputs both from the sensory nerve net of the umbrella and from specialized sensory domains within rhopalia, and generate sensory-input-dependent, as well as spontaneous and regular, electrical impulses to coordinate bell contractions [95]. Developmental genetic as well as ontogenetic evidence suggest that rhopalia and hydrozoan marginal sensory structures (e.g. eyes and tentacle bulbs with statocysts) might be related [96]. Comparative analyses of developmental gene expression patterns among these potentially homologous sensory structures will be important for clarifying early history of cnidarian sensory structure evolution. Recent data on scyphozoan jellyfish (*Aurelia sp.*) *Pou* and *Otx* genes propose that rhopalia evolved from preexisting sensory structures that developed distinct populations of sensory cells differentially expressing *Pou* genes within Otx oral-neuroectodermal domains [97]. Data from *Aurelia sp.* combined with the existing data from Bilateria thus suggest that the last common ancestor of Cnidaria and Bilateria may have used *Otx* to define neuroectoderm around the mouth from which distinct sets of sensory cells differentially expressing Pou-I/Pit-1 and Pou-IV/Brn-3 developed. This cooperation of Otx and Pou4 factors is supported in this study by the fact that a transcription factor with high similarity to Otx was also found in *Craspedacusta sowerbyi* transcriptome (Table S3).

The function and evolutionary conservation of Pou domain transcription factors is not limited to the nervous system and sensory organs. For example, Pou domain protein Oct4 is considered central to pluripotency in mammals. Analysis of the expression pattern and function of a *Pou* gene from marine cnidarian *Hydractinia echinata* shows that it is expressed in the embryo and adult stem cells of the animal and that its ectopic expression in epithelial cells induces stem cell neoplasms and loss of epithelial tissue [98].

Difficult classification of the csSix-X factor to any of the Six subclasses shows how the Six genes could have evolved. The recognition motif in homeodomain helix3 does not match the Six-specific WFKN but corresponds to the WFAN motif typical of homeodomain of class Irx (Iroquois), a member of both the Atypical homeodomain superclass and the Six class. The BlastX search of the whole csSix-X EST using Swissprot database resulted in the best hit (E-value 5e-20, Max score 99,4%, Query coverage 63%, Max identity 29%) to the mouse Six2 protein. This result reflects the existence of a Six specific-domain located upstream of the homeodomain and the absence of consensual amino acid IRO motif PATKPKIWSLADTA in the C terminal region [99], [100]. The phylogenetic tree was constructed from *Nematostella vectensis* XP_001633591.1, all *Craspedacusta sowerbyi* Six factors and representative metazoan Irx amino acid sequences. Here csSix-X and its closest relative *Nematostella vectensis* protein XP_001633591.1 form one branch together with the Six sequences detached from the Irx cluster (Figure S6). The presence of the two different classification features in one protein may be explained by the existence of the ancestor *Irx* gene and its fusion with a part of the *Six* gene encoding the SD region and loss of the sequence encoding the IRO motif in the hydrozoan branch. Another possibility is that this hybrid gene originates in an ancient gene belonging to the Atypical homeobox gene superclass from which both *Six* and *Irx* genes evolved sometimes in early metazoan evolution. The highly similar Atypical superclass gene was also identified in this study in the *Nematostella vectensis* genome (nucleotide sequence ID XM_001633541.1, predicted protein XP_001633591.1), which indicates the conservation of this gene across the Cnidaria phylum.

Expression data imply that in *Craspedacusta sowerbyi*, the Pou and Six transcription factors are employed in the development of sensory organs and nervous system and in the control of their function. With the exception of *csPou4f3* all the *Pou* genes are expressed in statocysts located in the cup-like structures at the highly innervated bell margin. Different expression patterns of *csPou4f1*, *csPou4f2* and *csPou4f3* support the notion of functional differentiation inside the *Pou4* subclass. *csSix1/2A* and *csSix4/5B* expression, also detected in statocysts of *Craspedacusta sowerbyi*, may be associated with the regulation of genes for Na+/K+ ATPase transporters involved in the development of the sensory system as a binding partner of Eya4 regulator [33], [35]. In vertebrates, subclass *Six1/2* genes are expressed during formation of the statokinetic apparatus of inner ear and in nasal placode epithelium [101]. It is reasonable to suppose that the subclass *Six1/2* function is associated with simple statokinetic organs also in hydrozoans. The high expression level of *Six1/2* genes was also observed in hydrozoan *Podocoryne carnea* tentacle bulbs carrying sensory organs, and weak expression was detected in the same organs in hydrozoan *Cladonema radiatum* [34]. Muscle strand-specific expression of *csSix1/2A* supports the opinion about the conservative role of Six1 factors in cooperation with Eya2 as regulators of transcription of the *MyoD* gene family employed in formation of muscles across vertebrates and invertebrates [89], [102]–[104]. Four zones of *csSix-X* expression at manubrium opening probably correspond to accumulated tactile and olfactory receptors conducting information about the contact with prey to four manubrium muscle strands [16].

*csSix3/6B* expressed in a circular structure lengthwise in the bell could be involved in formation and reconstitution of neural structures in a similar way as described for neurons in higher organisms, where *Six3* controls the equilibrium between proliferation and differentiation of defined precursor populations during mammalian neurogenesis [105]. Regulation of maintaining and releasing progenitor cells in or from undifferentiated state may be important for cnidarians because of their high degree of regeneration ability.

Expression of *csSix1/2A*, *csSix-X*, *csPou4f2* and *csPou6* in gonads is probably associated with gonadal tissue activity during maturation and release of oocytes. No expression of these factors was detected in free oocytes (data not shown). *csSix1/2A* could play a role in the control of cell cycle in G2/M checkpoint as described in proliferating human cells [106]. Expression analyses suggest that Pou and Six proteins are involved in the molecular mechanisms similar to those in bilaterian animals including vertebrates. This indicates evolutionary conservation of signal pathways regulating development and function of sensory organs from basal metazoans to vertebrates.

We found a relatively high number of homeobox transcription factors involved in sensory organ development in the *Craspedacusta sowerbyi* transcriptome, especially in the case of Pou and Six classes. This suggests that this eyeless hydrozoan jellyfish possesses a more sophisticated system of perception, nerve impulse conduction and reactions to sensory stimuli than expected in such a simple animal. The insight into the presence, diversity and expression of these factors in hydrozoan jellyfish highlights their evolutionarily conserved functions. *Craspedacusta sowerbyi* as a basal metazoan model organism has a potential to study early evolution of sensory organs and stabilization of rising regulatory mechanisms that are conserved throughout the bilaterian species. This study extends the findings about the complexity of cnidarian genomes by showing that the transcriptome of hydrozoan cnidarian is rich in homeodomain transcription factors employed in the regulation of development and function of sensory organs.

## Supporting Information

Figure S1 **Domain structure of conserved regions of** ***Craspedacusta sowerbyi*** **Six and Pou transcription factors.** HD abbreviates homeodomain, SD Six-specific domain, PD Pou-specific domain and VL variable linker. The length of SD varies from 115 to 120.
(TIF)

Figure S2 **Amino acid sequence alignment of the HD region of Pou class transcription factors.** Maximum likelihood method (WAG protein substitution model, bootstrap 1000) output with the sequence name on the left and length in amino acid residues at the bottom. Bold font emphasizes *Craspedacusta sowerbyi* sequences, violet box and arrow emphasize Pou6 cnidarian sequences and Pou6 specific amino acid residue and red box and arrows emphasize Pou4 cnidarian sequences and Pou4 specific amino acid residues. Phylogenetically representative sequences from the following metazoan species were selected for comparison: sponge (*Amphimedon queenslandica*), cnidarians (*Nematostella vectensis*, *Eleutheria dichotoma*, *Hydra magnipapillata*), invertebrates (*Oikopleura dioica*, *Schistosoma mansoni*, *Drosophila melanogaster*, *Branchistoma floridae*) and vertebrates (*Xenopus laevis/tropicalis*, *Danio rerio* and *Homo sapiens*). For the list of ID numbers of the reference sequences see Text S1.
(TIF)

Figure S3 **Phylogenetic tree of the HD region of Pou class transcription factors.** The phylogenetic tree was calculated by the maximum likelihood method (WAG protein substitution model, bootstrap 1000) and processed by NJ Plot software. For the list of ID numbers of the reference sequences see Text S1.
(TIF)

Figure S4 **Amino acid sequence alignment of the Pou4 variable linker region.** Maximum likelihood method (WAG protein substitution model, bootstrap 1000) output with marked sequence name on the left and length in amino acid residues at the bottom. Black arrows point at positions of differences among csPou4f1, csPou4f2 and csPou4f3. Bold font emphasizes *Craspedacusta sowerbyi* sequences and the red box groups cnidarian sequences. Phylogenetically representative sequences from the following metazoan species were selected for comparison: cnidarians (*Nematostella vectensis*, *Aurelia sp.*, *Hydra magnipapillata*), invertebrates (*Drosophila melanogaster*, *Saccoglossus kowalevskii*, *Branchistoma floridae*) and vertebrates (*Xenopus laevis*, *Danio rerio*, *Mus musculus* and *Homo sapiens*). For the list of ID numbers of the reference sequences see Text S1.
(TIF)

Figure S5 **Amino acid sequence alignment of the Six class transcription factor homeodomain.** Maximum likelihood method (WAG protein substitution model, bootstrap 1000) output with marked sequence name on the left, length in amino acid residues at the bottom. Bold font emphasizes *Craspedacusta sowerbyi* sequences, blue box cnidarian sequences of Six3/6 subclass, red box Six1/2 subclass, violet box Six4/5 subclass and green box groups unclassified cnidarian Six sequences. Yellow stripe and brace at the top of the figure mark the four amino acid diagnostic motif region.
(TIF)

Figure S6 **Phylogenetic analysis of Six and Irx transcription factors (N-terminal, SD and HD region).** Phylogenetic tree compiled of *Craspedacusta sowerbyi* Six amino acid sequences (Text S2), *Nematostella vectensis* protein XP_001633591.1 (Text S1) and representative metazoan sequences from the Irx class of homeodomain proteins (Text S1) using the maximum likelihood method (WAG protein substitution model, bootstrap 1000).
(TIF)

Table S1 **List of RACE primers.** Primer nomenclature GSP1 or 2 and NGSP1 or 2 corresponds to Clontech manuals and abbreviations mean Gene Specific Primer 1 (reverse) or 2 (forward) and Nested Gene Specific Primer 1 (reverse) or 2 (forward). Conditions of PCR and nested PCR amplifications were given by the Clontech manual. Pou primers: Instead of GSP1 and NGSP1 for *csPou4f1* and *csPou4f2* primers, GSP1-Pou4f3 and NGSP1-Pou4f3 were used. The nucleotide sequences for *csPou4f1*, *csPou4f2* and *csPou4f3* are the same in the given region. RACE products were cloned and sequenced as mentioned in the previous paragraph. Six primers: Nested PCR reactions were required only in the case of *csSix3/6B*, in both directions, and in the cases of *csSix1/2A*, *csSix1/2B* in forward direction.
(DOC)

Table S2 **List of primers and PCR parameters for mRNA probe synthesis.**
(DOC)

Table S3 **List of contigs with the best hit to homeodomain protein (defined by the lowest E-value).**
(DOC)

Table S4 **Similarity search for sensory development regulatory factors at the nucleotide level.** Cut-off parameters: E-value<2, Bit score >30, Identity >60%, for each factor contig with the best E-value hit.
(DOC)

Text S1 **List of NCBI ID numbers of reference sequences.** Sequences used in Figure 2, S2, S3, S4,S5 and S6.
(DOC)

Text S2 **Nucleotide (CDS) and amino acid sequences of** ***Craspedacusta sowerbyi*** **Pou and Six homeobox transcription factors.**
(DOC)

Text S3 **Transcriptome of** ***Craspedacusta sowerbyi*** **.** List of contigs from 454 pyrosequencing, service Newbler assembly.
(DOC)
