# Supplementary material for: Diversity, Phylogeny and Expression Patterns of Pou and Six Homeodomain Transcription Factors in Hydrozoan Jellyfish Craspedacusta sowerbyi
Source: PLoS One. 2012 Apr 30;7(4):e36420. doi: 10.1371/journal.pone.0036420 (PMC3340352; doi:10.1371/journal.pone.0036420)
Supplement: Table S1 — List of RACE primers. Primer nomenclature GSP1 or 2 and NGSP1 or 2 corresponds to Clontech manuals and abbreviations mean Gene Specific Primer 1 (reverse) or 2 (forward) and Nested Gene Specific Primer 1 (reverse) or 2 (forward). Conditions of PCR and nested PCR amplifications were given by the Clontech manual. Pou primers: Instead of GSP1 and NGSP1 for csPou4f1 and csPou4f2 primers, GSP1-Pou4f3 and NGSP1-Pou4f3 were used. The nucleotide sequences for csPou4f1, csPou4f2 and csPou4f3 are the same in the given region. RACE products were cloned and sequenced as mentioned in the previous paragraph. Six primers: Nested PCR reactions were required only in the case of csSix3/6B, in both directions, and in the cases of csSix1/2A, csSix1/2B in forward direction. (DOC) [file pone.0036420.s007.doc]

| **Gene** | **Forward primer** | **Reverse primer** |
| --- | --- | --- |
| csPou4f1 | GSP2-Pou4f1 (5’-GTTCTGTCCGCTTGGCTGGACGAG-3’) | GSP1-Pou4f3(5’-GTCCAACTTTTCGGCGATCGCAGC-3’) |
| nested | NGSP2-Pou4f1 (5’-GGGGGCGAACAGAATGAAGGCCAAG-3’) |  |
| csPou4f2 | GSP2-Pou4f2 (5’-GAGGAAGCCGAGCGAGCTAACAGAATC-3’) | GSP1-Pou4f3(5’-GTCCAACTTTTCGGCGATCGCAGC-3’) |
| nested | NGSP2-Pou4f2 (5’-GGCCAAAGACAGTGCTTTCTTACCG-3’) |  |
| csPou4f3 | GSP2-Pou4f3 (5’-GGCATTGAAGCCTGTTCTGTCCGC-3’) | GSP1-Pou4f3(5’-GTCCAACTTTTCGGCGATCGCAGC-3’) |
| nested | NGSP2-Pou4f3 (5’-CGAGGCCGAGAGGGCGAACAGAATG-3’) | NGSP1-Pou4f3 (5’-GGCGAAATACGCCTTCCAGTGACCG-3’) |
| csPou6 | GSP2-Pou6 (5’-GGCTCGAGAAGACAGCAGTGGTCTTTCG-3’) | GSP1-Pou6(5’-GGCTGTACGTAGGCCCATTCAGAACCG-3’) |
| nested | NGSP2-Pou6 (5’-CTTTAAGGCCAGAAGGATCGCTCTCGGC-3’) | NGSP1-Pou6 (5’-CATTTCACGCCCGACATCATCCTGTGT-3’) |
| csSix1/2A | GSP2-Six1/2A (5´-TCGGCGTAAATACCCACTTC-3´) | GSP1-Six1/2A(5´-GACCGCCTTTGCTTTTACCACCGACTC-3´) |
| nested |  | NGSP1-Six1/2A (5´‑CGACCACAGAAACCTAGCCAGGCGATC-3´) |
| csSix1/2B | GSP2-Six1/2B (5´‑CATGCTCGACGCGCACTGCACGAAGC-3) | GSP1-Six1/2B (5´-CCAACAGGCTGGTCTTTGGATACCGCC-3´) |
| nested |  | NGSP1-Six1/2B (5´-CAGGTTGACCGCTGCTGATCAGGCATTC-3´) |
| csSix3/6A | GSP2-Six3/6A (5´‑CATGGAGGCAGAGAGGATTC-3´) | GSP1-Six3/6A (5´-CCAGTTCCCGCTTCTTCGAAGGGCTCGG-3´) |
| csSix3/6B | GSP2-Six3/6B (5´-AGCCGAGGAAGGCCACTTGGTCC-3´) | GSP1-Six3/6B (5´-CATTCATCACGTCGAACATCTCCGGTG-3´) |
| nested | NGSP2-Six3/6B (5´‑GTTCGCAAGCGCTTTCCGCTTCCTCGGAC-3´) | NGSP1-Six3/6B (5´-GTCTCCACACTCCTCCAACGTCTCG-3´) |
| csSix4/5B | GSP2-Six4/5B (5´-GGTGAGAGGTCGCCCGTTAGGAGCTGTA-3´) | GSP1-Six4/5B (5´-GAGTCAGTTCAGTGCGCTTG-3´) |
| csSix-X | GSP2-Six4/5A (5´-GCGCGAGAAGAACGAACTCCCTACCACG-3´) | GSP1-Six4/5A (5´‑CTCGCGCCTTGCGGGCATATAGAAG-3´) |
