# Supplementary material for: Diversity, Phylogeny and Expression Patterns of Pou and Six Homeodomain Transcription Factors in Hydrozoan Jellyfish Craspedacusta sowerbyi
Source: PLoS One. 2012 Apr 30;7(4):e36420. doi: 10.1371/journal.pone.0036420 (PMC3340352; doi:10.1371/journal.pone.0036420)
Supplement: Table S2 — List of primers and PCR parameters for mRNA probe synthesis. (DOC) [file pone.0036420.s008.doc]

| Probe | Forward primer | Reverse primer | Product length/bp | Anneal. temp./ °C |
| --- | --- | --- | --- | --- |
| csPou4f1 | POU41H-F (5’-GAATTCGGCTTCTGGGGTACAC-3’) | POU41H-R (5’-GACAGAAAACTTCATCCGTTTC-3’) | 419 | 52 |
| csPou4f2 | POU42H-F (5’-ATGCCCATATTCTCAGTTCTCAAC-3’) | POU42H-R (5’-ACTTCATGCGCTTCTTCTTTTGC-3’) | 858 | 55 |
| csPou4f3 | POU43H-F (5’-TAAGATCTTTCCATGCCTACGAAG-3’) | POU43H-R (5’-CAGAAAACTTCATCCGTTTCTTC-3’) | 945 | 52 |
| csPou6 | POU6H-F (5’-GAAAGGCATCAGCTGTTTCAG-3’) | POU6H-R (5’-TTGCTTCGAGTTTCGACCTTC-3’) | 1285 | 55 |
| csSix1/2A | SIX1/2AH-F (5’-CGCATCTGATGGGAGGCTTTGTG-3’) | SIX1/2AH-R (5’-GCAAATGGCTGTGATGCTTTCTGG-3’) | 965 | 60 |
| csSix1/2B | SIX1/2BH-F2 (5’-GTAGACCACTTGCAGCGTTAGAT-3’) | SIX1/2BH-R2 (5’-AGGGGTTCTTCTTGTAGGCTTC-3’) | 615 | 57 |
| csSix3/6A | SIX3/6AH-F (5’-TGGCTCCTAAAGTGTGTACGAACC-3’) | SIX3/6AH-R (5’-TCAGCACTCGTTTACTCTTGCCTG-3’) | 968 | 60 |
| csSix3/6B | SIX3/6BH-F2 (5’-GTGTTTGATTGGGTCTCTTCTTG-3’) | SIX3/6BH-R1 (5’-CTTGAACCAATTGCCGACTT-3’) | 674 | 55 |
| csSix4/5B | SIX4/5BH-F (5’-CGTCGGGTTTGGATTCCCACAGTA-3’) | SIX4/5BH-R (5’- TGCCTCAGAGTCCGAGTCAG -3’) | 787 | 60 |
| csSix-X | SIX4/5AH-F1 (5’-GACGGCAGTGTACAGGTTGA-3’) | SIX4/5AH-R (5’-ACGAACTGCGTGACCACTTGG-3’) | 916 | 60 |
