# Supplementary material for: Diversity, Phylogeny and Expression Patterns of Pou and Six Homeodomain Transcription Factors in Hydrozoan Jellyfish Craspedacusta sowerbyi
Source: PLoS One. 2012 Apr 30;7(4):e36420. doi: 10.1371/journal.pone.0036420 (PMC3340352; doi:10.1371/journal.pone.0036420)
Supplement: Table S3 — List of contigs with the best hit to homeodomain protein (defined by the lowest E-value). (DOC) [file pone.0036420.s009.doc]

| **Query** | **Protein ID** | **Identity [%]** | **Bit score** | **Organism** | **Protein definition** |
| --- | --- | --- | --- | --- | --- |
| contig00388 | [GenBank: ADB66165.1] | 95 | 88.6 | Aurelia sp. 1 NN-2010a | **Brn-3**-like protein |
| contig00452 | [GenBank: BAF91572.1] | 90 | 139 | Scolionema suvaense | **Msx** protein |
| contig08120 | [GenBank: AAT11875.1] | 87 | 84.3 | Cladonema radiatum | sine oculis-like transcription factor **Six4/5** |
| contig07713 | [GenBank: ACM62736.1] | 80 | 71.6 | Clytia hemisphaerica | **Mox** homeodomain transcription factor protein |
| contig03286 | [NCBI RefSeg: XP_002164016.1] | 79 | 112.0 | Hydra magnipapillata | PREDICTED: similar to homeobox protein **Pknox2** |
| contig07026 | [GenBank: ABU92523.1] | 67 | 142 | Eleutheria dichotoma | **POU** class **6** |
| contig13696 | [NCBI RefSeg: XP_727979.1] | 65 | 44.7 | Plasmodium yoelii yoelii | hypothetical, homeobox-containing protein |
| contig01932 | [NCBI RefSeg: XP_002157088.1] | 58 | 106 | Hydra magnipapillata | PREDICTED: similar to homeobox protein **Otx** |
| contig01141 | [GenBank: ACH89437.1] | 56 | 107 | Capitella teleta | **NK**-like homeobox protein **5** |
| contig07065 | [GenBank: AAT11872.1] | 54 | 40.8 | Podocoryna carnea | sine oculis-like transcription factor **Six3/6** |
| contig13614 | [NCBI RefSeg: XP_724544.1] | 48 | 33.1 | Plasmodium yoelii yoelii | Homeobox-containing protein |
| contig07711 | [NCBI RefSeg: XP_002125149.1] | 48 | 48.5 | Ciona intestinalis | PREDICTED: similar to **cut-like** homeobox **1** |
| contig04855 | [GenBank: ADY47187.1] | 48 | 54.7 | Ascaris suum | Homeobox protein **ceh-37** |
| contig10979 | [GenBank: EFV58496.1] | 47 | 102 | Trichinella spiralis | homeobox protein **Hmx1** |
| contig05641 | [NCBI RefSeg: XP_002168431.1] | 46 | 101 | Hydra magnipapillata | PREDICTED: similar to **POU** class **6**, partial |
| contig00913 | [NCBI RefSeg: NP_200030.1] | 46 | 32.0 | Arabidopsis thaliana | **HDG7** (Homeodomain Glabrous 7) transcription factor |
| contig09982 | [NCBI RefSeg: XP_001520389.1] | 45 | 35.8 | Ornithorhynchus anatinus | PREDICTED: similar to **orthodenticle-related** homeobox **5** |
| contig00318 | [NCBI RefSeg: XP_002913157.1] | 45 | 34.7 | Ailuropoda melanoleuca | PREDICTED: homeobox protein **cut-like 2**-like |
| contig01247 | [NCBI RefSeg: NP_001090515.1] | 43 | 33.5 | Xenopus laevis | homeobox protein **DLL-2** |
| contig09753 | [NCBI RefSeg: NP_001133035.1] | 43 | 34.7 | Salmo salar | homeobox protein **HoxA1aa** |
| contig01505* | [NCBI RefSeg: XP_002160331.1] | 43 | 123 | Hydra magnipapillata | PREDICTED: similar to **cut-like** homeobox **1** |
| contig12654 | [GenBank: AAI63896.1] | 42 | 32.7 | Danio rerio | **Iroquois** homeobox protein **1 a** |
| contig03422 | [NCBI RefSeg: XP_002715836.1] | 42 | 36.2 | Oryctolagus cuniculus | PREDICTED: **aristaless-like** homeobox 3 |
| contig11560 | [NCBI RefSeg: XP_643746.1] | 41 | 33.1 | Dictyostelium discoideum | Homeobox protein 2 |
| contig02867 | [NCBI RefSeg: XP_001110975.2] | 41 | 32.0 | Macaca mulatta | PREDICTED: similar to **tetra-peptide repeat homeobox** |
| contig07030 | [NCBI RefSeg: NP_001036563.1] | 41 | 35.8 | Drosophila melanogaster | orthopedia, isoform C |
| contig09252 | [NCBI RefSeg: XP_001103636.2] | 40 | 38.9 | Macaca mulatta | PREDICTED: homeobox protein **notochord**-like |
| contig00692 | [GenBank: AAG52245.1] | 39 | 31.6 | Arabidopsis thaliana | homeobox protein (**Glabra2**) |
| contig02822 | [NCBI RefSeg: XP_002401118.1] | 38 | 34.7 | Ixodes scapularis | **Lim** homeobox protein, putative |
| contig02216 | [NCBI RefSeg: XP_002420762.1] | 38 | 32.3 | Candida dubliniensis | Homeobox transcription factor, putative |
| contig00596 | [NCBI RefSeg: XP_002928754.1] | 37 | 40.4 | Ailuropoda melanoleuca | PREDICTED: homeobox protein **Ventx**-like |
| contig02040 | [GenBank: ADY45809.1] | 34 | 90.1 | Ascaris suum | Homeobox protein **SIX3** |

* Contigs 1505 and 1506 have the same hit to cut-like homeobox 1 (*Hydra magnipapillata*) and after manual control of the nucleotide sequence it was found out that these two contigs have not been assembled because of low sequence quality. It is the reason why only 32 protein matches are mentioned in the table (contig 1506 was left out).
