# Supplementary material for: Diversity, Phylogeny and Expression Patterns of Pou and Six Homeodomain Transcription Factors in Hydrozoan Jellyfish Craspedacusta sowerbyi
Source: PLoS One. 2012 Apr 30;7(4):e36420. doi: 10.1371/journal.pone.0036420 (PMC3340352; doi:10.1371/journal.pone.0036420)
Supplement: Table S4 — Similarity search for sensory development regulatory factors at the nucleotide level. Cut-off parameters: E-value<2, Bit score >30, Identity >60%, for each factor contig with the best E-value hit. (DOC) [file pone.0036420.s010.doc]

| **Transcription Factor** | **NCBI ID** | **Contig ID** | **Contig Length [bp]** | **Bit Score** | **Identity [%]** |
| --- | --- | --- | --- | --- | --- |
| **Vsx2** (Homo sapiens) | CCDS9827.1 | contig07713 | 394 | 34 | 82 |
| **Crx** (Homo sapiens) | CCDS12706.1 | contig10337 | 215 | 36 | 82 |
| **Irx2** (Homo sapiens) | CCDS3868.1 | contig12337 | 394 | 36 | 81 |
| **Sox2** (Homo sapiens) | CCDS3239.1 | contig11523 | 362 | 59 | 76 |
| **Hes5** (Homo sapiens) | CCDS41233.1 | contig13883 | 546 | 30 | 75 |
| **Mec7** (Caenorhabditis elegans) | NM 076912.2 | contig11205 | 945 | 413 | 75 |
| **Neurog1** (Homo sapiens) | CCDS4187.1 | contig00076 | 732 | 34 | 71 |
| **Lhx1 (Lim1)** (Homo sapiens) | CCDS11316.1 | contig05269 | 247 | 35 | 70 |
| **Isl1** (Homo sapiens) | CCDS43314.1 | contig10674 | 117 | 32 | 70 |
| **Lhx6** (Homo sapiens) | CCDS6838.2 | contig00778 | 624 | 34 | 69 |
| **Olf1 (Ebf1)** (Homo sapiens) | CCDS4343.1 | contig08897 | 514 | 32 | 68 |
| **Eya3** (Homo sapiens) | CCDS316.1 | contig04672 | 246 | 35 | 67 |
| **Dach1** (Homo sapiens) | NM 004392.5 | contig04241 | 216 | 31 | 67 |
| **Delta1** (Homo sapiens) | CCDS5313.1 | contig07227 | 188 | 39 | 67 |
| **Neurod4 (Math3)** (Homo sapiens) | CCDS8886.1 | contig00182 | 2425 | 38 | 67 |
| **Msx1** (Homo sapiens) | CCDS3378.2 | contig00452 | 241 | 83 | 67 |
| **Irx6** (Homo sapiens) | CCDS32449.1 | contig00602 | 1547 | 38 | 67 |
| **Eya2** (Homo sapiens) | CCDS13403.1 | contig05602 | 233 | 34 | 66 |
| **Dlx2** (Homo sapiens) | CCDS2248.1 | contig09522 | 206 | 35 | 66 |
| **Gata3** (Homo sapiens) | CCDS31143.1 | contig13822 | 620 | 32 | 66 |
| **Sox10** (Homo sapiens) | CCDS13964.1 | contig11523 | 362 | 47 | 66 |
| **Atoh7 (Math5)** (Homo sapiens) | CCDS7276.1 | contig10762 | 1554 | 35 | 66 |
| **Irx1** (Homo sapiens) | CCDS34132.1 | contig10988 | 758 | 34 | 66 |
| **Pax3** (Homo sapiens) | CCDS48294.1 | contig04855 | 240 | 41 | 65 |
| **Eya4** (Homo sapiens) | CCDS5165.1 | contig07709 | 260 | 34 | 65 |
| **Numb** (Homo sapiens) | CCDS32116.1 | contig13552 | 807 | 33 | 65 |
| **Hmx3** (Homo sapiens) | CCDS41575.1 | contig01141 | 668 | 76 | 65 |
| **Lin11** (Caenorhabditis elegans) | NM 060295.3 | contig07713 | 394 | 32 | 65 |
| **Mec4** (Caenorhabditis elegans) | NM 078311.4 | contig11239 | 1279 | 39 | 65 |
| **Ntrk1 (TrkA)** (Homo sapiens) | CCDS30891.1 | contig01922 | 237 | 48 | 65 |
| **Pax2** (Homo sapiens) | CCDS7499.1 | contig04855 | 240 | 32 | 64 |
| **Jagged2** (Homo sapiens) | CCDS9998.1 | contig03244 | 552 | 41 | 64 |
| **Lhx2** (Homo sapiens) | CCDS6853.1 | contig10295 | 879 | 35 | 64 |
| **Notch1** (Homo sapiens) | CCDS43905.1 | contig08941 | 327 | 43 | 63 |
| **Dlx6** (Homo sapiens) | CCDS47647.1 | contig03830 | 175 | 36 | 63 |
| **Dlx3** (Homo sapiens) | CCDS11556.1 | contig04913 | 395 | 31 | 63 |
| **Vsx1** (Homo sapiens) | CCDS13168.1 | contig06668 | 433 | 32 | 63 |
| **SOHo-1** (Gallus gallus) | NM 205386.1 | contig01141 | 668 | 63 | 63 |
| **Irx4** (Homo sapiens) | CCDS3867.1 | contig10416 | 769 | 36 | 63 |
| **Lfng** (Homo sapiens) | CCDS34587.1 | contig04433 | 244 | 33 | 62 |
| **Gfi1** (Homo sapiens) | CCDS30773.1 | contig13840 | 353 | 39 | 62 |
| **Otx1** (Homo sapiens) | CCDS1873.1 | contig04855 | 240 | 38 | 62 |
| **Irx5** (Homo sapiens) | CCDS10751.1 | contig05918 | 231 | 39 | 62 |
| **Jagged1** (Homo sapiens) | CCDS13112.1 | contig03341 | 600 | 40 | 61 |
| **Rbfox1 (Fox1)** (Homo sapiens) | NM 001142333.1 | contig10302 | 943 | 34 | 61 |
| **Irx3** (Homo sapiens) | CCDS10750.1 | contig01566 | 1244 | 48 | 61 |
| **Ngf** (Homo sapiens) | CCDS882.1 | contig00807 | 422 | 31 | 61 |
| **Eya1** (Homo sapiens) | CCDS34906.1 | contig12619 | 937 | 37 | 60 |
| **Atoh1** (Homo sapiens) | CCDS3638.1 | contig05150 | 208 | 33 | 60 |
| **Dlx4** (Homo sapiens) | CCDS45728.1 | contig01141 | 668 | 49 | 60 |
| **Idx1 (Pdx1)** (Homo sapiens) | CCDS9327.1 | contig01141 | 668 | 53 | 60 |
