# Supplementary material for: Diversity, Phylogeny and Expression Patterns of Pou and Six Homeodomain Transcription Factors in Hydrozoan Jellyfish Craspedacusta sowerbyi
Source: PLoS One. 2012 Apr 30;7(4):e36420. doi: 10.1371/journal.pone.0036420 (PMC3340352; doi:10.1371/journal.pone.0036420)
Supplement: Text S1 — List of NCBI ID numbers of reference sequences. Sequences used in Figure 2, S2, S3, S4,S5 and S6. (DOC) [file pone.0036420.s011.doc]

POU sequences

>gi|51702521|ref|NP_005595.2| POU domain, class 3, transcription factor 2 [Homo sapiens]

>gi|5453936|ref|NP_006227.1| POU domain, class 3, transcription factor 3 [Homo sapiens]

>gi|110624765|ref|NP_002690.3| POU domain, class 3, transcription factor 1 [Homo sapiens]

>gi|110624763|ref|NP_000298.2| POU domain, class 3, transcription factor 4 [Homo sapiens]

>gi|119589273|gb|EAW68867.1| POU domain, class 1, transcription factor 1 (Pit1, growth hormone factor 1), isoform CRA_b [Homo sapiens]

>gi|169144954|gb|ACA49233.1| POU domain class 4 transcription factor 2 [Homo sapiens]

>gi|4505965|ref|NP_002691.1| POU domain, class 4, transcription factor 3 [Homo sapiens]

>gi|110347449|ref|NP_006228.3| POU domain, class 4, transcription factor 1 [Homo sapiens]

>gi|55957721|emb|CAI15171.1| POU class 2 homeobox 1 [Homo sapiens]

>gi|4505959|ref|NP_002689.1| POU domain, class 2, transcription factor 2 [Homo sapiens]

>gi|148664218|ref|NP_055167.2| POU domain, class 2, transcription factor 3 [Homo sapiens]

>gi|42560248|ref|NP_002692.2| POU domain, class 5, transcription factor 1 isoform 1 [Homo sapiens]

>gi|23463326|ref|NP_694948.1| POU domain, class 5, transcription factor 2 [Homo sapiens]

>gi|223890225|ref|NP_002693.3| POU domain, class 6, transcription factor 1 [Homo sapiens]

>gi|157743257|ref|NP_009183.3| POU domain, class 6, transcription factor 2 isoform 1 [Homo sapiens]

>gi|110339141|gb|ABG67834.1| POU3A [Nematostella vectensis]

>gi|110339143|gb|ABG67835.1| POU3B [Nematostella vectensis]

>gi|82621599|gb|ABB86471.1| POU1-POU class homeobox protein [Nematostella vectensis]

>gi|156401033|ref|XP_001639096.1| predicted protein [Nematostella vectensis]

>gi|82621545|gb|ABB86444.1| NVHD084-POU class homeobox protein [Nematostella vectensis]

>gi|47086331|ref|NP_998016.1| pituitary-specific positive transcription factor 1 [Danio rerio]

>gi|47086419|ref|NP_997972.1| POU domain, class 4, transcription factor 2 [Danio rerio]

>gi|66392152|ref|NP_571353.1| POU domain, class 4, transcription factor 3 [Danio rerio]

>gi|41152054|ref|NP_958442.1| POU domain, class 4, transcription factor 1 [Danio rerio]

>gi|18859255|ref|NP_571188.1| POU domain, class 6, transcription factor 1 [Danio rerio]

>gi|167859585|gb|ACA04747.1| PouI [Amphimedon queenslandica]

>gi|268053987|gb|ACY92480.1| BRN3 transcription factor [Saccoglossus kowalevskii]

>gi|11066192|gb|AAG28492.1|AF196575_1 class IV POU-homeodomain protein [Xenopus laevis]

>gi|10441360|gb|AAG17008.1|AF184979_1 class IV POU-homeodomain protein [Xenopus laevis]

>gi|158518452|ref|NP_001103520.1| POU class 6 homeobox 1 [Xenopus (Silurana) tropicalis]

>gi|83751826|gb|ABC42926.1| class IV POU transcription factor [Branchiostoma floridae]

>gi|22833164|gb|AAF48447.2| abnormal chemosensory jump 6, isoform A [Drosophila melanogaster]

>gi|56694824|gb|AAW23074.1| POU4 [Oikopleura dioica]

>gi|221114520|ref|XP_002158636.1| PREDICTED: similar to predicted protein [Hydra magnipapillata]

>gi|256072096|ref|XP_002572373.1| pou4/brn-3 [Schistosoma mansoni]

>gi|256076863|ref|XP_002574728.1| pou6f1/brn-5 [Schistosoma mansoni]

>gi|156636505|gb|ABU92523.1| POU class 6 [Eleutheria dichotoma]

SIX sequences

>gi|47155914|gb|AAT11871.1| sine oculis-like transcription factor Six1/2 [Podocoryna carnea]

>gi|47155916|gb|AAT11872.1| sine oculis-like transcription factor Six3/6 [Podocoryna carnea]

>gi|47155918|gb|AAT11873.1| sine oculis-like transcription factor Six1/2 [Cladonema radiatum]

>gi|47155920|gb|AAT11874.1| sine oculis-like transcription factor Six3/6 [Cladonema radiatum]

>gi|47155922|gb|AAT11875.1| sine oculis-like transcription factor Six4/5 [Cladonema radiatum]

>gi|50841484|gb|AAT69263.1| homeobox protein sine oculis six 1/2 [Aurelia aurita]

>gi|260788250|ref|XP_002589163.1| SIX class homeodomain transcription factor [Branchiostoma floridae]

>gi|260788242|ref|XP_002589159.1| SIX class homeodomain transcription factor [Branchiostoma floridae]

>gi|158937637|gb|ABW83199.1| homeodomain transcription factor Six1b [Danio rerio]

>gi|12744793|gb|AAK06772.1|AF323497_1 SIX1 [Homo sapiens]

>gi|13242167|gb|AAK16583.1|AF332198_1 SIX2 [Homo sapiens]

>gi|6230605|dbj|BAA86223.1| SIX4 [Homo sapiens]

>gi|40354216|ref|NP_787071.2| homeobox protein SIX5 [Homo sapiens]

>gi|186910311|ref|NP_031400.2| homeobox protein SIX6 [Homo sapiens]

>gi|4321417|gb|AAD15753.1| Six3 [Homo sapiens]

>gi|182890536|gb|AAI64650.1| Six2.1 protein [Danio rerio]

>gi|68085152|gb|AAH66428.2| Six4.1 protein [Danio rerio]

>gi|11344511|dbj|BAB18511.1| homeobox protein six4.2 [Danio rerio]

>gi|11344513|dbj|BAB18512.1| homeobox protein six4.3 [Danio rerio]

>gi|82621637|gb|ABB86490.1| SIX1/2-SINE class homeobox protein [Nematostella vectensis]

>gi|82621629|gb|ABB86486.1| SIX4/5b-SINE class homeobox protein [Nematostella vectensis]

>gi|82621573|gb|ABB86458.1| SIX3/6-SINE class homeobox protein [Nematostella vectensis]

>gi|82621547|gb|ABB86445.1| NVHD093-SINE class homeobox protein [Nematostella vectensis]

>gi|82621513|gb|ABB86428.1| SIX4/5a-SINE class homeobox protein [Nematostella vectensis]

>gi|156389434|ref|XP_001634996.1| predicted protein [Nematostella vectensis]

>gi|156385345|ref|XP_001633591.1| predicted protein [Nematostella vectensis]

>gi|144369366|dbj|BAF56229.1| Six-C [Anthopleura japonica]

>gi|144369363|dbj|BAF56228.1| Six-A [Anthopleura japonica]

>gi|291230538|ref|XP_002735213.1| PREDICTED: SIX homeobox 1-like [Saccoglossus kowalevskii]

>gi|259013340|ref|NP_001158378.1| SIX homeobox 3 [Saccoglossus kowalevskii]

>gi|5106876|gb|AAD39863.1|AF099184_1 homeobox protein SIX3 [Drosophila melanogaster]

>gi|5106878|gb|AAD39864.1|AF099185_1 homeobox protein SIX4 [Drosophila melanogaster]

>gi|27923824|sp|Q95RW8.1|OPTIX_DROME RecName: Full=Protein Optix; AltName: Full=Homeobox protein SIX3; Short=D-Six3

>gi|2495294|sp|Q27350.1|SO_DROME RecName: Full=Protein sine oculis

>gi|144369357|dbj|BAF56226.1| Six-C [Ephydatia fluviatilis]

>gi|299906605|gb|ADJ58017.1| Six1/2 [Chalinula loosanoffi]

>gi|144369360|dbj|BAF56227.1| Six-C [Sycon calcaravis]

>gi|221121056|ref|XP_002156969.1| PREDICTED: Six-B [Hydra magnipapillata]

>gi|221128975|ref|XP_002162399.1| PREDICTED: Six-A [Hydra magnipapillata]

IRX sequences

>gi|284927658|gb|ADC29552.1| iroquois homeodomain protein b [Suberites domuncula]

>gi|284927656|gb|ADC29551.1| iroquois homeodomain protein a [Suberites domuncula]

>gi|259013289|ref|NP_001158359.1| iroquois [Saccoglossus kowalevskii]

>gi|193788691|ref|NP_001123285.1| iroquois homeobox A [Strongylocentrotus purpuratus]

>gi|193083641|gb|ACF10241.1| iroquois C [Branchiostoma floridae]

>gi|260834613|ref|XP_002612304.1| iroquois 3 [Branchiostoma floridae]

>gi|193083633|gb|ACF10237.1| iroquois A isoform 1 [Branchiostoma floridae]

>gi|193083637|gb|ACF10239.1| iroquois B [Branchiostoma floridae]

>gi|193083641|gb|ACF10241.1| iroquois C [Branchiostoma floridae]

>gi|51479177|ref|NP_077313.3| iroquois-class homeodomain protein IRX-1 [Homo sapiens]

>gi|197100900|ref|NP_001127694.1| iroquois-class homeodomain protein IRX-2 [Homo sapiens]

>gi|226371735|ref|NP_077312.2| iroquois-class homeodomain protein IRX-3 [Homo sapiens]

>gi|7705555|ref|NP_057442.1| iroquois-class homeodomain protein IRX-4 [Homo sapiens]

>gi|139394646|ref|NP_005844.4| iroquois-class homeodomain protein IRX-5 [Homo sapiens]

>gi|42544241|ref|NP_077311.2| iroquois-class homeodomain protein IRX-6 [Homo sapiens]

>gi|157116233|ref|XP_001658394.1| iroquois-class homeodomain protein irx [Aedes aegypti]

>gi|221104313|ref|XP_002166749.1| PREDICTED: similar to iroquois homeobox protein 4b [Hydra magnipapillata]

>gi|193202365|ref|NP_492533.2| IRoquois subclass of homeoboX family member (irx-1) [Caenorhabditis elegans]
