# Supplementary material for: Diversity, Phylogeny and Expression Patterns of Pou and Six Homeodomain Transcription Factors in Hydrozoan Jellyfish Craspedacusta sowerbyi
Source: PLoS One. 2012 Apr 30;7(4):e36420. doi: 10.1371/journal.pone.0036420 (PMC3340352; doi:10.1371/journal.pone.0036420)
Supplement: Text S2 — Nucleotide (CDS) and amino acid sequences of Craspedacusta sowerbyi Pou and Six homeobox transcription factors. (DOC) [file pone.0036420.s012.doc]

>*csPou4f1*_CDS

atggaagtgc taagatcttt ccatgcctac gaagacacga gacattttat gggcaagctt 1

tttgtttcag gcatgtttga cagtttggac gaagctcatc ttctcaattc tcaactcgac 61

tcaatggcca agtacttacc gaagcaaagc tcagcgtcat cgacaaaaat gcctacagca 121

tttcttcata gccttcatgc aaccaaccct gatccaacac aatatgaccc acatgacatt 181

ctagatcaaa tatctgcgtc actgcaacca ccagctgtgt ccgagtcagc gtacgaaatt 241

ccatcgacat ccgtttcgac caacactagc aactactcaa ccagtcactt gttgtcgcca 301

gctacaccat ctttctccac ctccttcact aataacagct tcactaatac gccttttaat 361

aatactttcg cggctgctgc ggccgcggct gcagctgcgg cagcggctgc tcaactaggt 421

caggaaacgg aatgtacgcc aagggagctg gagtggttcg cagagaggtt caagcaacgc 481

cgaattcggc ttctggggta cactcaggat gacgtaggtg cggctctggc ccatctgaaa 541

ttgcctggtg ttggctcact cagtcagtcg acgatatgca ggttcgagtc tctgacatta 601

agtcacaaca acatgatggc attgaagcct gttctgtccg cttggctgga cgaggccgag 661

ggggcgaaca gaatgaaggc caaagacagc gccttcttgc caaatgccga taagaagaga 721

aaacgcactt caataggtgc tgcagaaaaa cggtcactgg aggcgtattt cgccatgcag 781

ccgagaccgt ccagcgataa aattgctgcg atcgctgaaa agttggactt atctaagaac 841

gtcgtccgcg tttggttttg caaccaacgg cagaagaaga aacggatgaa gttttctgtc 901

cattga           907

>csPou4f1_translated protein

MEVLRSFHAYEDTRHFMGKLFVSGMFDSLDEAHLLNSQLDSMAKYLPKQSSASSTKMPTA

FLHSLHATNPDPTQYDPHDILDQISASLQPPAVSESAYEIPSTSVSTNTSNYSTSHLLSP

ATPSFSTSFTNNSFTNTPFNNTFAAAAAAAAAAAAAAQLGQETECTPRELEWFAERFKQR

RIRLLGYTQDDVGAALAHLKLPGVGSLSQSTICRFESLTLSHNNMMALKPVLSAWLDEAE

GANRMKAKDSAFLPNADKKRKRTSIGAAEKRSLEAYFAMQPRPSSDKIAAIAEKLDLSKN

VVRVWFCNQRQKKKRMKFSVH

>*csPou4f2*_CDS

atgttcgaca gccttgacga tgcccatatt ctcagttctc aactcgactc aatggcgaag 1

tatttgccta agcaaagttc ctcttcgtcg acgaaaatgc caacggcgtt cctacatagc 61

ctccacgcga caaaccctga tccaacgcaa tatgatccac atgacattct agatcagata 121

tctgcgtcac ttcagccgcc aactgtgtca gaaacaggtt acgaaaatcc gtcgacgtct 181

gtctcaacca acgctagcaa ctactcaaca agccacttgc tgtcaccatc aacgccgtcg 241

ttctcttcct cgttcaccaa cagcagtttc aataacaccc cgtttaataa tacctttgca 301

gctgctgcgg cggcggcggc agctgctgcg gcagcagttc aagtaggtca ggaaaccgaa 361

tgtacaccaa gggagctcga atggttcgcg gagaggttca agcaacgccg catcaaactt 421

ggtgtcacac aggctgacgt cggagcggct ttggcacatc tgaaactgcc tggcgtcggt 481

tcgctaagtc agtcgaccat atgcaggttc gagtctctga cgttgagtca caataacatg 541

atggccttga agccggtttt atcagcgtgg cttgacgaag ccgagcgagc taacagaatc 601

aaggccaaag acagtgcttt cttaccaaat gctgacaaaa aaagaaaacg cacgtctata 661

ggtgccgctg agaaacgatc cctggaagca tattttgcca tgcagccgag accgtctagc 721

gataaaattg cagcgattgc tgaaaagttg gacttgtcca agaacgtcgt ccgagtgtgg 781

ttttgcaatc aacggcaaaa gaagaagcgc atgaagttct ctgtgcactg a 832

>csPou4f2_translated protein

MFDSLDDAHILSSQLDSMAKYLPKQSSSSSTKMPTAFLHSLHATNPDPTQYDPHDILDQI

SASLQPPTVSETGYENPSTSVSTNASNYSTSHLLSPSTPSFSSSFTNSSFNNTPFNNTFA

AAAAAAAAAAAAVQVGQETECTPRELEWFAERFKQRRIKLGVTQADVGAALAHLKLPGVG

SLSQSTICRFESLTLSHNNMMALKPVLSAWLDEAERANRIKAKDSAFLPNADKKRKRTSI

GAAEKRSLEAYFAMQPRPSSDKIAAIAEKLDLSKNVVRVWFCNQRQKKKRMKFSVH

>*csPou4f3*_CDS

atgcgactaa agagcagtgc gtgcaggcta acgctggtta tcgttacgag ggcttttcgg 1

aagatggaag tgctaagatc tttccatgcc tacgaagaca cgagacattt tatgggcaag 61

ctttttgttt caggcatgtt tgacagtttg gacgaagctc atcttctcaa ttctcaactc 121

gactcaatgg ccaagtactt accgaagcaa agctcagcgt catcgacaaa aatgcctaca 181

gcatttcttc atagccttca tgcaaccaac cctgatccaa cacaatatga cccacatgac 241

attctagatc aaatatctgc gtcactgcaa ccaccagctg tgtccgagtc agcgtacgaa 301

attccatcga catccgtttc gaccaacact agcaactact caaccagtca cttgttgtcg 361

ccagctacac catctttctc cacctccttc actaataaca gcttcactaa tacgcctttt 421

aataatactt tcgcggctgc tgcggccgcg gctgcagctg cggcagcggc tgctcaacta 481

ggtcaggaaa cggaatgtac gccaagggag ctggagtggt tcgcagagag gttcaagcaa 541

cgccgcatca agcttggggt cacacaggct gacgtaggtg cggctctggc ccatctaaaa 601

ttgcctggtg ttggctcact cagtcagtcg acgatatgca ggttcgagtc tctgacatta 661

agtcacaaca acatgatggc attgaagcct gttctgtccg cttggctgga cgaggccgag 721

agggcgaaca gaatgaaggc caaagacagc gccttcttgc caaatgccga taagaagaga 781

aaacgcactt caataggtgc tgcagaaaaa cggtcactgg aggcgtattt cgccatgcag 841

ccgagaccat ccagcgataa aattgctgcg atcgccgaaa agttggactt atctaagaac 901

gtcgtccgcg tttggttttg caaccaacgg cagaagaaga aacggatgaa gttttctgtc 961

cattga           967

>csPou4f3_translated protein

MRLKSSACRLTLVIVTRAFRKMEVLRSFHAYEDTRHFMGKLFVSGMFDSLDEAHLLNSQL

DSMAKYLPKQSSASSTKMPTAFLHSLHATNPDPTQYDPHDILDQISASLQPPAVSESAYE

IPSTSVSTNTSNYSTSHLLSPATPSFSTSFTNNSFTNTPFNNTFAAAAAAAAAAAAAAQL

GQETECTPRELEWFAERFKQRRIKLGVTQADVGAALAHLKLPGVGSLSQSTICRFESLTL

SHNNMMALKPVLSAWLDEAERANRMKAKDSAFLPNADKKRKRTSIGAAEKRSLEAYFAMQ

PRPSSDKIAAIAEKLDLSKNVVRVWFCNQRQKKKRMKFSVH

>*csPou6*_ CDS

atgacatcaa gtatgcctgg tcttgctcct atcaatgtct attcagcaac ttcgttgggc 1

ggaaaggcat cagctgtttc aggggtactg ttacatttga aaacccctga cgggacgtcg 61

tccggtccgc acttgatgac gtcatcaatg ctaaagcgct ctctttcggc tcgagaagac 121

agcagtggtc tttcgatcga tatgcagaac tttataaacg acttcaaagc cagaaggatc 181

gctctcggct atacacagga tgatgtcggg cgtgaaatgt cggttctgaa tgggcctacg 241

tacagccagt cattcatatc aagatttgaa ggcaagcagt tggggatgaa ggcagctgag 301

cgaatgcgac ctatccttga cgcttggatt cagagcaaag aggaagagtt tcatcgcggt 361

agcaagtttg cgaaaaagcg aaggaaaaga acaagttttt ctccagatct tctcgacgtt 421

cttcttgatt actttaacaa aaatcagaag cccactcctg aagaattgca aatgattgcg 481

cagaagacgg gtcttgatgt gacaactgtc aaggtttggt tctgcaacaa gaagcagagt 541

ttgaaacgcg ctggacagcc tgtgcaagat aacactcttc gtgccgaaat cgatgcgaag 601

cgtaaactta agtctgagga tgagctaaat gataatgtgt ttgctttcgc ccagtctgct 661

tctctgaaga cattcgttcc ggtttccacg cctgctggcg ccgtgacagt acctttcttc 721

gtaaaccagg acgggagcag tattgcgatt gtatctgctg gaacagctgc aaatcagagc 781

gatgttcaga ggaacttgcc cgcagatgat gctggtgatc acatggcgac caccctttcc 841

caacatgtta tattatccaa cgtgaatata ttaccatcaa tggctgtagt tgataacgct 901

ctccagtctc ctgcctctat tgcttttcag ggtgcatccc agtcgcacaa cttggtggat 961

ggtagtcgtt tgctgcaagg tggcactgct ttgcaccaca ttcaggggtt gactcggtct 1021

atgactgaac ctaactgcgc cttgaatgat aatgacgact tgggagctga gtcgcgtagt 1081

catgtatctc acgtaccgcg caacaaaagc cgtggcgtag gctccttatc tggaatactt 1141

gaagttgtgc ataatgaaac ttctttgaac tctggttgca ttgcaaatag cggatgtgac 1201

gtctctggta tggctactga ttctgtcatc ctccagagtg aggccactaa catggagctg 1261

cggcagaagg tcgaaactcg aagcaagaag tcgttcgttt caaagcagaa gcaaaattac 1321

gtagcagcag cagcagcaac agaaagtaat tcagatgatg atagaaacga tgctgtgcta 1381

caatga           1387

>csPou6_translated protein

MTSSMPGLAPINVYSATSLGGKASAVSGVLLHLKTPDGTSSGPHLMTSSMLKRSLSARED

SSGLSIDMQNFINDFKARRIALGYTQDDVGREMSVLNGPTYSQSFISRFEGKQLGMKAAE

RMRPILDAWIQSKEEEFHRGSKFAKKRRKRTSFSPDLLDVLLDYFNKNQKPTPEELQMIA

QKTGLDVTTVKVWFCNKKQSLKRAGQPVQDNTLRAEIDAKRKLKSEDELNDNVFAFAQSA

SLKTFVPVSTPAGAVTVPFFVNQDGSSIAIVSAGTAANQSDVQRNLPADDAGDHMATTLS

QHVILSNVNILPSMAVVDNALQSPASIAFQGASQSHNLVDGSRLLQGGTALHHIQGLTRS

MTEPNCALNDNDDLGAESRSHVSHVPRNKSRGVGSLSGILEVVHNETSLNSGCIANSGCD

VSGMATDSVILQSEATNMELRQKVETRSKKSFVSKQKQNYVAAAAATESNSDDDRNDAVL

Q

>*csSix1/2A*_ CDS

atgaagctaa gcgacaagct aaacccttac ttctctcaaa atatccaaag aatggagtcg 1

catctgatgg gaggctttgt ggattgcagc gcaacaatta ctggacaacc atctgtggtg 61

aacttcactc cagagcaggt tgcttgcgtg tgcgaggttt tggagcagag cggaaacatc 121

gatcgcctgg ctaggtttct gtggtcgctt ccttcttacg acgacatcta tatgaacgag 181

tcggtggtaa aagcaaaggc ggtcgtcgca tttcatcagg gcagcatgca ggaactttac 241

tcgcttatcg aaaacaatca cttctctcct tcttctcatt ctaaaatgca gatgctgtgg 301

ttaagagcac attacatgga agccgaaaaa atacgcggtc ggcctctagg ggccgttgga 361

aagtacaggg ttcggcgtaa atacccactt ccacgcacga tctgggacgg agaagagacc 421

agctattgct tcaaggagaa gtcgagggcg gttctaaggg attggtacac cagcaatccc 481

tatccatcgc cacgcgagaa gaaagaattg tcagacagta ctggattgtc catcactcag 541

gtcagcaact ggttcaaaaa ccggaggcaa cgagatcgag ctgccgagat gaaggagaga 601

gatggcgaat ccaccgagga taaattcagc agccagtctc gttgggaatc ctcgcttgaa 661

catcatggcg tcgccaaagc atgcaagact gctccagggt ctcctcctgc ccagatttca 721

catcaatcca ctgacgagga agactccgag gcagatgtcg acacgctaca ccacgactca 781

attaagtcca actctagcac agaaaatgga tccactgaat actctcattc cgtgacgaat 841

cagcgcatga agcaggagag cccagccagc ccactgtatc cgatatacca gtccctggcc 901

tcggaaaggg gaagcgtgaa aaaagaagaa tctgcctcgc cagaacgcat cacagccatt 961

tgcatgccca tggcgtctgc ttacgatgtt ctgagta     998

>csSix1/2A_translated protein

MKLSDKLNPYFSQNIQRMESHLMGGFVDCSATITGQPSVVNFTPEQVACVCEVLEQSGNI

DRLARFLWSLPSYDDIYMNESVVKAKAVVAFHQGSMQELYSLIENNHFSPSSHSKMQMLW

LRAHYMEAEKIRGRPLGAVGKYRVRRKYPLPRTIWDGEETSYCFKEKSRAVLRDWYTSNP

YPSPREKKELSDSTGLSITQVSNWFKNRRQRDRAAEMKERDGESTEDKFSSQSRWESSLE

HHGVAKACKTAPGSPPAQISHQSTDEEDSEADVDTLHHDSIKSNSSTENGSTEYSHSVTN

QRMKQESPASPLYPIYQSLASERGSVKKEESASPERITAICMPMASAYDVLS

>*csSix1/2B*_ CDS atgcatagtg gcttcagttt aaacaagaac tttagcacga acatttacag tctgctatct 1

ccacaaatgc aatctgcgcc tattcgtcta gaagcaacag gtgaaactag catagaaagc 61

ggtaacaaaa agtcttgggc atgcagcacc cacatagaca ggcagatgcc tgtctttagc 121

gaacaccaca tcgaactagt gtcggaatgc ctgatcagca gcggtcaacc tgagcgactt 181

cggcgcttcc tctgggcggt atccaaagac cagcctgttg gcgactcgga agccgcgctt 241

gttgccaggg cgtacgttta cttctggcag aaggacttcg acagcctgta tcgagtgctg 301

cagatgcgga acttctcgaa gaaaaaccac gaacggttac aatcgctgtg gaggatagcc 361

cactacctag aggcagaagc acagcgaggg cggccgttag gggccgtcgg aaaatatcgc 421

atccgaagga agttccccct gccaaggaca atctgggacg gtgagcagaa ctcttactgc 481

tttcgcgaac atgctcgacg cgcactgcac gaagcctaca agaagaaccc ctaccccacg 541

gcaaaggaga aagcgaacct agcggctgaa acgagcctca gtgtcacgca agtcagcaac 601

tggttcaaaa accgccggca gagggtacgg gcgtcggaga gcagaaagag caggtaa 658

>csSix1/2B_translated protein

MHSGFSLNKNFSTNIYSLLSPQMQSAPIRLEATGETSIESGNKKSWACSTHIDRQMPVFS

EHHIELVSECLISSGQPERLRRFLWAVSKDQPVGDSEAALVARAYVYFWQKDFDSLYRVL

QMRNFSKKNHERLQSLWRIAHYLEAEAQRGRPLGAVGKYRIRRKFPLPRTIWDGEQNSYC

FREHARRALHEAYKKNPYPTAKEKANLAAETSLSVTQVSNWFKNRRQRVRASESRKSR

>*csSix3/6A*_ CDS

atggctccta aagtgtgtac gaaccattgt tgcacgaagg gccaaccagc acaagttcat 1

gatggcatgt ccgagcaagt caatcgccag cgggttgaac gtagacggcc atcattggca 61

cctcccagcc ggccttgcgc agccagacca gtgcctattt cgtcggaaca gatctctcgc 121

gtttgcgaga ctctagagga gtgcggtgac attgaaaggc tatcgcgttt tttgtggtca 181

ttgcccaacg caccagagtt gatggacgtg atgaatggaa acgaaactat tcttcgagct 241

cgcgcgctcg tgtcatttca ccacagcaac tttaacgagc tttaccacat tttagagcat 301

tttcgtttta gcaagaaatc tcacgcaaaa ctccaagcga tgtggttaga ggcgcattac 361

atggaggcag agaggattcg aggtaggcca ctaggacctg ttgacaagta tagagttcgc 421

aagcgcttcc ctcttcctcg gactatatgg gatggcgagc agaaaactca ttgcttcaag 481

gaaaggacaa gaaaactatt aagagagttc tatttgcaag atccataccc gagcccttcg 541

aagaagcggg aactggctga cgtcactcac ctcaccccga ctcaagtggg aaattggttt 601

aagaatcgac ggcaaagaga tagggctgct gccgcgaagc ataggtccca gaagcaacag 661

aagatatcct caagcctgaa cgacgaagtt gaagatgagg acagcgatgt ctttgatgaa 721

atattggacg atgatggctc cgagaacgac gttgatttag acgacatttc acattcgcca 781

caaatgcaag gaatgacagc ccaaacatcg agaagcaaga atttcatggt atccagtata 841

ttgagacaga cagacacttt ccgcatcgat gagaatatag gtcatcaggc aagagtaaac 901

gagtgctga           910

>csSix3/6A_translated protein

MAPKVCTNHCCTKGQPAQVHDGMSEQVNRQRVERRRPSLAPPSRPCAARPVPISSEQISR

VCETLEECGDIERLSRFLWSLPNAPELMDVMNGNETILRARALVSFHHSNFNELYHILEH

FRFSKKSHAKLQAMWLEAHYMEAERIRGRPLGPVDKYRVRKRFPLPRTIWDGEQKTHCFK

ERTRKLLREFYLQDPYPSPSKKRELADVTHLTPTQVGNWFKNRRQRDRAAAAKHRSQKQQ

KISSSLNDEVEDEDSDVFDEILDDDGSENDVDLDDISHSPQMQGMTAQTSRSKNFMVSSI

LRQTDTFRIDENIGHQARVNEC

>*csSix3/6B*_ CDS

atgaacggaa agatcttaaa tcctgatgca tttcatgtgt ttctagatgg gatgcccagg 1

gcggttgatc accagttagt ggcaagtcga cggatacttt tgccaactcc atgccgacct 61

tgctcctcca gttcagtgcc gttttcttcg gaacaaatct ctcgcgtttg cgagacgttg 121

gaggagtgtg gagacattca aaggttgtcg cgctttctgt ggtcattgcc caacgcaccg 181

gagatgttcg acgtgatgaa tggaaatgag actattcttc gagctcgagc cctggtatca 241

ttctaccaca acaactttaa cgaactctac tacattctag agcacttcag attcagcaag 301

aaatcccacg caaagctcca agctatgtgg ttagaggcac attacattga agcggagaga 361

agccgaggaa ggccacttgg tcccgttgat aaatacagag ttcgcaagcg ctttccgctt 421

cctcggacta tatgggatgg ggaacagaaa acgcattgtt ttaaggaaag gacaagaaag 481

cttttgaggg agttctattt acaagatcca tacccaagcc cgtccaagaa gcgagagctg 541

gctgacgcca ctcacttgac tccgacacaa gtcggcaatt ggttcaagaa tcgacgacaa 601

cgagatagag cggccgccgc aaaacacagg tctcagatga agaaccataa ttcttcgaac 661

cagcaggccg atgccgaaga tgaagacagc gatgtctttg atgaaatatt agctgatgcg 721

gcctctgacg aggacgaaga tttcgaagac gtcccgtacc cacctgactc ccacacaaga 781

gcggcccaac catcaagaag ctcaagtttc atggtatcca gtattttaaa acagacagac 841

acttttcaaa ttggttga         859

>csSix3/6B_translated protein

MNGKILNPDAFHVFLDGMPRAVDHQLVASRRILLPTPCRPCSSSSVPFSSEQISRVCETL

EECGDIQRLSRFLWSLPNAPEMFDVMNGNETILRARALVSFYHNNFNELYYILEHFRFSK

KSHAKLQAMWLEAHYIEAERSRGRPLGPVDKYRVRKRFPLPRTIWDGEQKTHCFKERTRK

LLREFYLQDPYPSPSKKRELADATHLTPTQVGNWFKNRRQRDRAAAAKHRSQMKNHNSSN

QQADAEDEDSDVFDEILADAASDEDEDFEDVPYPPDSHTRAAQPSRSSSFMVSSILKQTD

TFQIG

>*csSix4/5B*_ CDS

atgtcgtcgg gtttggattc ccacagtaat gttgagcttc agaagcatgc cagcgctgtt 1

ttaggttaca cggccgacca ggttgactgt atttgcgatg ctcttgtaca gagccaggat 61

catgagactt tagcaaagtt tttatggtcg attccaagaa gtgatctatt tttaagcagt 121

gagagtgttg tgaaagctaa ggcgcacgtg gcctttcagc agtgtcgcta tagggaactg 181

tacagtcttc tggagacaca cgaatttgac ccggactgtc atcctatcct tcagcaaatg 241

tggcacgaag cccactacgc tgaagcggag aaggtgagag gtcgcccgtt aggagctgta 301

gaaaagtacc gaatccgaag gaaatttccc ttaccaagaa caatttggga tggtgaggaa 361

acagtgtatt gctttaagga aaagtccaga caaattcttc gtgaatggta tgacaaaaat 421

aagtacccaa cgccgcagga taaacgcttg ttagccaagc gcactgaact gactctagtg 481

caagtctcaa actggtttaa aaatcggcgt caaagggata agccacaagg cgaagcagaa 541

gacaataagg gtcacgtaag atcgatgagc atggatggct ggtctggacc tagtctgcac 601

gttccattca tgagcaaccc ctacttaggt aaaacatcag gcagtgaaga cagcgactcc 661

gacggcaaaa cagtgatcaa gacggagcct cggtaccaag aagagactga ctcggactct 721

gaggcaaggc agtcggatta ttttggagat atggcatgcc atatttga   769

>csSix4/5B_translated protein

MSSGLDSHSNVELQKHASAVLGYTADQVDCICDALVQSQDHETLAKFLWSIPRSDLFLSS

ESVVKAKAHVAFQQCRYRELYSLLETHEFDPDCHPILQQMWHEAHYAEAEKVRGRPLGAV

EKYRIRRKFPLPRTIWDGEETVYCFKEKSRQILREWYDKNKYPTPQDKRLLAKRTELTLV

QVSNWFKNRRQRDKPQGEAEDNKGHVRSMSMDGWSGPSLHVPFMSNPYLGKTSGSEDSDS

DGKTVIKTEPRYQEETDSDSEARQSDYFGDMACHI

>*csSix-X*_ CDS

atggccaccg cgaacgctga tgctgcacag cgacctaaag aaatcgccca tgctgatttg 1

caagatgaga aacgctcgcc aggttttcat cccgaaaaga gatcgttcga tcgtttgcaa 61

attctgctgc agctgtggaa agaaaagaat cgctcctcgc atgcgacctt gcacgacaac 121

taccttgttg gagtagcttc tgaagtttgc tccttgctgc ttcaaacgaa cagctttgat 181

gctctggcta tatttttgga taggcttcct gacaaaaacg tctacaggca agatgaaatc 241

atccttcgcg ctaaagtaca tcttgctctc cgccaaggcg atacgacggc agtgtacagg 301

ttgattaagg atggttcgtt tgttgacggt gaagatctca ttaaagtctg ggacgacgct 361

ttgtatatgg atgaggagag acgacttgga aagccattaa caccactgat tcgcttccgg 421

cttcgtaaac gaaatcctcc tccctcttct atatgcccgc aaggcgcgag aagaacgaac 481

tccctaccac gagaggctac aagtgttctt aaatcgtggc tgcattgtca tgccgcggat 541

ccttacccat ccgcacttga gaagcaagag ctcgctagat tgtctggtct ctccggtggc 601

caggttaaaa cctggtttgc caacgcacga aggcgaagca aaaaagtcga gttacgtggt 661

gcttccccgc ctacgacgga ccatgcaaga tcgttatgta gcaatgaccc tatcgagttt 721

caaaagagca cttcgtctaa tgccttcgct ttccggccaa cagggagtac caatgagttt 781

ctctctggtt atgaaagcga caccttttcg tacatgcacg ggttctttca tgatcctgag 841

agctctttat tccatcatcc agatcatttc cacgggtcca gccagctaca actccatgct 901

catcagccat cggcttttca tagtcgtcat ctctatccaa gcctgggatg tccatttgtt 961

gcatctgctc acatgggact ctttcagcat ccttccagct ctagtggact gtttgttgaa 1021

agcttggctt gcccatccag ccttccatct aacttttctt cttgtgatgc tatgagattg 1081

caccggattc cacattgtcc gtcggagaac gtttgcctca atgctgcgaa agttctcgta 1141

aatttgtcaa ctgggcgcgg atattttcac ccttccatac caagtggtca cgcagttcgt 1201

ccgttctga           1210

>csSix-X_translated protein

MATANADAAQRPKEIAHADLQDEKRSPGFHPEKRSFDRLQILLQLWKEKNRSSHATLHDN

YLVGVASEVCSLLLQTNSFDALAIFLDRLPDKNVYRQDEIILRAKVHLALRQGDTTAVYR

LIKDGSFVDGEDLIKVWDDALYMDEERRLGKPLTPLIRFRLRKRNPPPSSICPQGARRTN

SLPREATSVLKSWLHCHAADPYPSALEKQELARLSGLSGGQVKTWFANARRRSKKVELRG

ASPPTTDHARSLCSNDPIEFQKSTSSNAFAFRPTGSTNEFLSGYESDTFSYMHGFFHDPE

SSLFHHPDHFHGSSQLQLHAHQPSAFHSRHLYPSLGCPFVASAHMGLFQHPSSSSGLFVE

SLACPSSLPSNFSSCDAMRLHRIPHCPSENVCLNAAKVLVNLSTGRGYFHPSIPSGHAVR

PF
