# Supplementary material for: Diversity, Phylogeny and Expression Patterns of Pou and Six Homeodomain Transcription Factors in Hydrozoan Jellyfish Craspedacusta sowerbyi
Source: PLoS One. 2012 Apr 30;7(4):e36420. doi: 10.1371/journal.pone.0036420 (PMC3340352; doi:10.1371/journal.pone.0036420)
Supplement: Text S3 — Transcriptome of Craspedacusta sowerbyi . List of contigs from 454 pyrosequencing, service Newbler assembly. (DOC) [file pone.0036420.s013.doc]

>contig00001 length=238 numreads=3

AAGCAGTGGTaTcAACgcAGAGTACGCGGGGctGGCCAATTTATTTAGGCAtcGCATcTc

cTTACgAGACGaTGACGAACTCGCtGCTTTtCaTTtCtgTGGCTGCGTGTCTTTGGttgA

CTTCGGCAGCTGCTGCGCGATGCCGTAATGAATATCTCCtttGCTTGGATAACGGCGAAT

CAAAACGGCAGTGCATCgccaatgttctcaaatgtctcacggcgaaaccacagttgga

>contig00002 length=421 numreads=4

CCACAGGGCACATCTTCTATAGGTTCATTCTTTGGGCCCATCATAAGAATTGTTCTTTGA

ATGGCTTTTTGGTGCAGGTCGTCCTTTTTtCCAGGTACATAGTTTGGACCCATGATTCGA

ACCTTAAGACCACTACCAATTTTCCCAaGAAAAAaCACGACCGAAGGCATAAAAACcGCC

CTTTTTCAGGGGACGgTACCATCTTTGAGATATACATCAtCaGGGGAGcTTCtgggtcac

agttcttgattccgttaaacgctgcatcatcctgcggcccttcatagagtagctcagcac

ggtatctttgagcggttacaggggatggtaagtgaatagttataagctgcagcaatgtat

caccagcaggcaaccatttctgcatgatttttctaagtagaggcttttcctctagatcct

t

>contig00003 length=382 numreads=2

actctcagcattcttattcttaacaaagcagtcatcggtaccattgctctctgagtcagg

tatatctacttgagaccaatcctgtagagttttcagcaagctgtagtacaattcacttgc

cgggctttgcgtgactttccttgactttagactgttccagttagcgatatttcccgtatc

aatcagaattctggacacttctccaagaaCTGCTTgCCcTTGCAAGTTCTTAAGACCGTT

TTTAATTCGAGcttctacctggttcactcgaacatgatgttggcgactaccagttagcac

ttccgtagccattccagtgtcctagtgatatattttataataaataagatcttgtcggat

ctcttctctggtgccggagcat

>contig00004 length=200 numreads=2

GGCTTGGTTTCGctCCTTGATCTTCTTGCTGATGTCGAGAAGGGCTGCCCGGAACCTCTT

CAGAGCCGCAGTAGAAATCGGATCATACTGGTACTGCACttCAAAaTCGGCCAGCTTCTT

CGTCGCTCTCTCACTCAATAGCTTCcGCCcAaCCAAAATGatgttcgacgtgatttgctt

gctgggcaggtggtctatta

>contig00005 length=1034 numreads=24

GACTAAGATAAAGGCTGCTCAGAGAATGAAtGAAGATTACGTCTGTCCGCGTGAGTTCAA

CGAAATTCTtGCCATGAAAAAGCTGTACGTTGCGGGTCTGGATCGAAACACATCGGATGA

GgAaCTTCAGGACTtATTTTTctACGTTtGGTGAaGTCGTTGACGGCGTAGTAATCAAAA

GAGACAATGGCAAACCTAAGACGtttGgTTtCGTtACATTCGCTcTTTGTGATGACGTGG

ACGAGtGTTTGTTAAAGCGGGACAGTCTGAAACTgAACGGCTGTCGATTGGACGTTAAGC

GTGCCGTCCCTAAGGATGACACGAGCGAGACcTCTCAACTGAGAACAAAGCAACTTTTCA

TATCCAACGTTACCCCAGGGCTCACAGAGGAGAAGGTTCGCGATTATCTGGAAAGTCGCC

ATCCTTCGAGGTTTGGGAAaATAGAGAAGATCATTTTGATGAAGgCCAGAGACCAATCGA

CCGGTAAACTCACAGATAAAAATCGTGGTTTCGGTTTCATCACCCTGGACTCGGAAGATT

TGGCTGACCGAATAGCCATTGGCGATGCTGTTTTtAACATTGGTGGTATGAGTCGACCTC

AAGGGATACAGAAAGCCCTTCCAAGGGACGATCCACACGCGGCCCCGCATCCAATATCGT

CGCACGGTAGTGTTCATGGGCGAGAAGGAAGACcTGGgCGCAGTAGTAtCAGAGACGGCG

GCTCGCGtGGTAGAGACCAGGCAGCGGAGTGGGATgATTATGGGTAtgactcctattcgA

GAGGTTtCGACGAtAGGCGTGGTGaGAaCtACGGAGGCCGaTATGATGATGACTATGATG

ACTACTACAGCGACAACcGGGgTGGTGgTGGAaGtCGTGgAACcTATGGAAGTGgAGGCG

GTTgCTCCTCAAGATACGGTCAACCGCAGCAAAGTCGCGGAAGGGGTGGCGGCGGGGGCT

CATCTGCGTATCGCGGAGGTGCCGTTTCGCGCTTTAGTGGTGGCATGAGTCATGGGCGTG

GTGGTCGGTTTAGt

>contig00006 length=211 numreads=1

aaccccttttatcgtaagttagcaatattttgtctatccaacacgtaattctacttttca

tacgccatacacaagcattaggaaacttttaatgaatgctaagatatcccatcttcgcag

tctgtcccatgccatattgcaattatcctaaggttttaatctcttgacaggtgaaattat

gagagactagatttctaagaaatccaatggg

>contig00007 length=1947 numreads=187

GgTAGCACATAGAaTGGCTCACTACAACTTCAAGAAGATTCAAGTTGTGCCTACCTCTAA

GGACTTCATTGACATAATTCTTTCAAAGACCCAAAGGAAAACACCAACTGTGGTTCATAA

GCATTACCAAATCAGTCGcaTTCGCCAGTTCTACCTGCGCAAGATCAAGTACACCCAGCA

AAACTaCcATGACAAGCTGTCTCAAATTATTGCTGACTTTCCAAAAaTTGAGGATGTGCA

CCCGTTCTATGCCGATCTGATGAATGTGTTGTATGACAAAGATCATTACAaGCTAGCTCT

TGGACAGATAAATACGGCCCGGCACCTTATAGACAATGTTTCCAAAGACTATGCAAGACT

TATGAAGTACGGTGACTCATTGTATAGATGCAAGCAGCTAAAGAAGGCTGCCCTTGGCAG

GATGTGCACTATAATGAAGCGGCaGAaCCAAAGCCTTCAGTACCTGGAACAGGTGCGGCA

GCATCTGTCTCGATTGCCTTCAATTGATCCGAATACAAGGACCTTGCTTTTGACTGGATT

TCCTAATGTAGGCAAGTCAAGTTTCATGAACAAGGTGACTAGAGCAGATGTTGAAGTGCA

GCCGTACGCGTTCACAACCAAGTCACTCTTTGTTGGTCACATGGATTATAAGTATTTGCG

ATGGCAGGTTGTCGATACGCCTGGAATATTGGATCACTCGTTAGAGGAAAGAAACACTAT

TGAAATGCAGGCTATAACAGCATTGGCACATCTGCGAGCTGCAATTTTGTATGTGATGGA

TATAAGCGAGCAGTGCAACAAGTCcATAAACGAGCAGGTTCAGCTGTTTGAAAGCATCAA

GCCaCTTTTTGTAAACAAGCCgaTATTGGTaTGCCTGAATAAGACAGATATAGTgAAGTT

ATCGGAATTGCCCGAGGAAAAGAAAAGTTTTcTTGAAAAATTTCAGGAAATGGGTGTGCC

TGTTATTGAGATGAGTACGATTTCGGAAGAAGGCATTATGAATGTCAAGACCGAAGCATG

CGAGCGTCtgCTCGCCCAGCGAGTTGAAATGAAGTTGAAGAACAAAAAGGTTAATGACGT

GATCAACAGGCTTCACGTGGCTACGCCCAAGCCCCGTGACGACAAGGAAAGACCTGCTTT

CATTCCTGATAGCTTTAAGCTGAAGGGGAAAGGTATGGATGTGGAGCGCCCGCAGAGAAA

GCTTCAGAAGCAGCTGGAGCAGGAACTGGGAGAGGACTACAGGTTTGACGACAAGGCGCT

GTATCTGATTAAGGGTAATGAAAAATACGACATTATTCCTGAAATAATTAACGGTAAAAA

TATCGCAGATTATATCGATCAGGATATTTTTGAGAAGTTGGAGCAACTCGAAAGGGAGGA

AGAATTGCGAGAGGCAGCTGGATTTTACGAGTCTGATTCGGAGGAACTTGGCCCCGAGGA

AGAGGAAATTCGGAAGCAAGCAGTCGAGATCCGTAAGCAGAAGAAGATTCGTATGCAAGA

ACATCGGATGAAGAAAGGCCCCAGCAATGCGCCTGTTTTGCCACGCAAAGCCGCCATGCG

CGTTGAGAAATTCTCGCGAGCCGGTCAGAAGAGGAAATTTACGGACATGGCcGTCGACAT

GATCGATGAGGGAGATGAGGAGATGCAGGACGTCAGCGTTAAGAAGCGAGCACGTTCTCG

TTCGGCGCAAAGTGCACGGCAAGCGAAGTTGGATGGATCagTTGCCAGAAGCAAGTCTCG

TGGACACAGCGTTGCCAGCACTCGCAACCaGTCCATTGCTGCCCACCCcGTGCTGAAGTC

CAAGGCGaagaGAaTGTaCAAAATTGGGTCAAAaGAAAAATGATtCAGTTTGGTCGTGCT

GGCGAATCGGATCGTCACATTGCCaCTAAAATGCCAAAGCATCTATtCtCcGGCAAGCGA

AagTCGGGGAAgaCTCaGAGGCGTTAA

>contig00008 length=1500 numreads=61

GACTAAGAAAAATGCACGAAACATTACtGAGGCGTAGTTGGCTAAaTATATCTCATTTAC

CGAGACGGCCAaTTtCTCGCTACTTtGGAGTTTGgTCAGTCCGATCAGAGTATGAATGTG

TCGACTGCATGCTAACCAGATGCcACCCCTTTGCTTtAAAGCAGATACGATGTtACCAGG

CTCCCCCaccAaaGCAGGGTCTCCTTtCAAaGTTCATTAACAaCaTAAGAGAAGgTATAG

AACGAGATAAGCAGCTTCAGgAAAACTTGAaGCGgTTCCGAAAAGAGGCTCAAAaGGTGG

AAGAACATGAAGTCTTtAAAGGGGCTAAAGCGAGGATAAGTTTATTCGGTCAGCTTAAAG

AGAAGGTCTCGCcTTtCGCAaGTAAAGTTCAAGAGTCTGTCCAAGAAATATCGAAGACAG

CTTCTACTACTGCTGGAAAGGTTtACAAGGATGTGTCAGAAaGCGAAGTGTTtAAAAAAA

GCCAGGAGGTGACCGAAGAGCTTGGCAAATCGGCACAGGAGGCTGCTTCGAAGATATCTG

AACAGGGAGAGACCTTAAGTAAAAGCGAAACTTTTAAGCAGGCATCCAAAGCCTTTTCAG

CTGTGAAGAGTGACTTATTtGATGACATTGCTAAGGAATCTAGACCTTACCAACGACCAG

AAGTTCTGAAGCgAAGGACGAATGCTGGCAAGAGTGAGAAACCTGCCAAAGCTATTGAAG

TTAACGAAGATGCAAGAAATGTGGTTCTCCATAAAGACTCCAAGTGGCAGCAGCAGTGGA

AGGACTTCCGAGATAATAACCCAGTAGTTACTGGCTTGTTTAGTCTGAAAACAAAATACG

ACGAATCAGATAACGTGGTCATCCGTGCCAGTCGTGTTGTAACTGATAaGCTTGCAGATG

TGTTTAGCGATGTATTTTCCCAGTCGGACATGGCTAAAACTATTGCAGAGATCACGAAAA

TAGATCCTCAGTTCAACAAAGACCAGTTCATTAAAGAGTGCGAGTTTGAAATCATCCCCA

CTGTTTtGGAGGCATATTTGAGAGGGGATCTTGAAGTGCTGAAAGACTGGTGTCATGACG

GAGCGTTCAATATCCTCTCGACtCACATAAAGCACAATCAGGCGCTAGGGTTGAAAGTGC

AGTCGAAAGTTTTAGATGTCCGAGATGTCGACcTTGCGCTGGCAAAAGTAaTGGACCAAG

GTCCTGTCCTTATCCTCACCTTTCAAGTTCAGCAGACGgTCATGGTCACTGATAGTTCtA

aCAaGATtGtCGAaGgTGGAATTGACCATATtGAAAACaTcAACtacGTTtGGGCaTTaT

GTcgCGATCaGAGcaTCTTCGACCACAAGACGGCTTGGCGATTGTTGgAGTTTGCAATTC

aGAACGCAACGCcTTACCTCTAATGTAGCTCCAGATTTGTACGCGCGCGCTACTtGCCGG

CCTTTtGAACtCGTTTGTCTCTATTtGAATAGAATTtCTATTtGaCcGaaaaaaaaaaaa

>contig00009 length=112 numreads=5

aTCCATTTCAAACATTTGTAAACGGTATTGTGAATAGAATAACCTTGTATTTTTAAGCAA

GAATTTCAAAAATTCTGCTATTGAATAAAAATTTtATAAGCAACAtCAgaaC

>contig00010 length=183 numreads=3

cTTCcTCGGCCGTCTATGgATCCTGGcACGTCGAACCCGAGAAAGCGACGGAGAAaGAAG

GCTCTTGTAAAGACGCcGCTAGaTTTTATAGAGAGCCcTtACAACGgCCCTTTtCAaGCT

GAaTCGCcTGTTTGCGCGGCCCTCAAcccGTCACCCTTCCCGTgcggccaatcgatggcc

agg

>contig00011 length=916 numreads=81

AAGCAGTGGTATCAACGCAGAGTACGCGGGGCTTTTTAAGGCGAGTGCCTCGAACTGCAA

GTGTGGTCtCACTGCACTGCTAACCGTACcgtAAGTTTCCAGTGAACTTGGTGGAATTAT

TACTGCTAACTAAAAAgCGTTTgCCATGGCTGACAGCGAGGAAACTGAGACTTTTGCCTT

TCAGGCAGAAaTTGCGCAGCTGATGAGTCTGATCaTAAACACGTTTTATtCCAACAAAGA

AGTATTTCTGAGGGAACTGATTTCCAaCGCTTCTGATGCTTTGGACAAAATTCGGTACCT

GTCGTTAACGGATCCTACTGTGCtGGAAACCGGCAAAGACCTCAAAATCGATTTAATTCC

AAACAAGGACGAAAAGACTCTGACTCTCATTGATTCTGGCGTTGGTATGACGAAGGCTGA

CCTTGTAAATAATCTAGGCACGATCGCCAAgTCAGGTACTAAGGCTTTCATGGAGGCCCT

TCAGGCTGGAGCTGATATATCGATGATTGGTCAGTTCGGTGTGGGTTTCTACTCGGCTTA

CCTTGTTGCTGAGAAAGTCAAAGTTATTTCCAAGCATAATGATGACGAGCAGTACATTTG

GGAATCGGCTGCTGGTGGGTCTTTCACTGTCACACGAGATACAGTAAGTGAACCACTTGG

ACGTGGAACCAAGATAATTTTGTACATGAAGGAAGACATGGTTGATTATCTAGAAGAGAA

GAAAATCCAAGACATTGTTAAGAAGCACTCGCAATTTATTGGTTACCCTATCAGCTTGCA

AAAGCAGAAGACAAgagACAAGGAAGTTAGTGATGAcGAGGAGGAGGAGGACAAGGATAA

AAAGGAGGATAAGAAAGAAGGGGAAGATAAATCTGACGAAGAGCCTGAAGTTGAAGATGT

AGAGGATGAAGAGAAG

>contig00012 length=168 numreads=2

aGgCGCttACATGTAACCTACGACGCCACGAtgacctagaagtacacggactatgaagaa

aaggttaggtggagaatagccatctcttgactgacgaaagtgagcataccaacatgtggc

cgccccctgccacgtcgataatcagaccagagcagcgagaacagaaaa

>contig00013 length=140 numreads=1

tgaagctaaaagagacagatatggctaagattgctcgtggttactcaacctatacctgtg

tgctcggcatacttctgatttgctttggtttgggagcttcaatagctgcctgggttttgg

ggagcaaggtgtctgacgta

>contig00014 length=818 numreads=43

tttCCAATTTCACAGTTACTGACTGTCCAAAGaCCGCCACACgTGAACACGcATGCAGAA

GACCACTAACTGCTGTGATCTGCTTCGTCATCTTCGATGCCTTCGTCGAGGTCAGCATTT

TCTTCGGGACTCGAGAGTTCTTCAAATTTCCCATTCGAATGCCACACGCACTTCAGCGTC

ACGTGAAaGTTTACCAGAGTGTGGGAGAAGAGTTTCAAAATCCGACACTGCTCGGgAgTG

AGTTTATCGCCTTTCTTGCACACGCTGAAACTGTCAGTTAAATGGATTATGCCTTTCTTA

AGAACAGTAGGTAATCCGAGTTGTCGTAGATTCGGCTCCATCGAATGAGGGAAGGTGTCA

CTGTCTAAAGGTCCTTCAGGAAGAACTACTGTGTCTGTaGCCGTATTTCCCGATCTCGCA

TAATCAGGCTCcGAGAACGTACTGAACCACCTTATTACGGACTCCTGCTCGTCGTTAGTG

AaCATCAGACCAACATTTCcAACCAAATGCTGgCTGATTTTATGCATATTTTCTTTGTAT

tcTGTTTCCGGTGTGCGACCAAGGGCTAAACCcATGACTTTGTtCTTCCCAAAGAAAAAT

CTGCTcGTTTtCCAAtGTTGACGAACATCcTTAaTTTtAGTATTTCTCATGTTTTCTACC

GAAAACACATAAGCTGAGGCATATTCATCACATGATTGTTGAACTAGCTGCACAAGGTCT

TTCTTCAGCTCAAAGCCTTTCTTCGTAGTTTTAGACAAAGACACCACTTTGTTTCTTTTG

GACTTAGGCATTTTTTTtttCACCAATGCAtcATCTGC

>contig00015 length=155 numreads=3

tGTCAACTACAGATTCATCTGCTCCTCCTTTACTGATATTTCCTTGGGCAAGCTTCAGAG

ATCGCTTCATTTGTAATGGtCCTCCTGGGTGtGAGGTTACAACTAATCcTAGTGAATGGA

TgACTGGGGAAgTCTTtCTCTAATTTgtggatgta

>contig00016 length=235 numreads=5

tACGCATGTCGGGAGTGCTCTGAGGCTGCGATGAGAAaGTGTTACGACGAGCGGAAAAGC

GACATGTTCACGTgtGGAGCAaGTTTTCCCGACATTGCCAAATCGGGCGCGTtCTGCATC

TTCAACTACAGCATGAGCCTCGTGCAATTtCTCACTAtgacaccgttctgtgtcctctgt

tggctgaaggctgctgccagttgcggcagcgttacgatcggacccagactgtcgg

>contig00017 length=762 numreads=15

ATATGTCTGTCCTACAGAGGTCATTGCATTCAGTGACAGGGCAAACGAGTTTCGTGCCaT

TAACTGCGAGGTTCTAGCTTGTTCTGTGGATTcAcACTTCTCTCACCTTGCTTGGACCCA

ACAGCCTAGAAACACTGGTGGACTTGGAAAAATGAATATTCCAATTTtGTCAGACATCAC

CAaGCAGATTTCCCGAGACTATGGAGTTCTTATAGAGGAGGCtggCATTTCCcTAaGGGG

CCTGTTTATCATTGATGGGAAAGGTGTTCTGCGACAGATTACCGTAAAaTGACCTtCCCA

TTGGTCGTTCCGTtGACGAAACaTTGCgTcTaGTGCAaGcACTCCAGTTCgTCGagAAGC

ATGGcGAAgTTTGCCCCGCTggATGgAAaCCAGGTAGCGCTACGATTAAGCCAGGTGTGG

TGGAAAGCAAGAAATACTTTTCTAAGCAGTAAAGAAGTCGATGCGGCTTGAATCCGACAT

CCCAGTtGgCGCCTTCCATAAGGGATTGTCAAATTTAATGTGCTCCCATGCCGGAAGCAt

ATtGtAACGAAtaTTtgATTCCTaTaTtaTTtGAtaTTTCTTATGCttGtcGGTGAAATA

TgtGATCCTGAGTGTCGagatttgtgttatgtttagttaagatttcggttcgggtttctg

gacttcgttcagtattcgtggaaaatacttgtgaaatcgttatctttaatccgcgagcat

gatgtcgcttcattaaaagtcccaagaaggcccggcctttat

>contig00018 length=208 numreads=1

ttcaaaggaaatatcatgaacatgtgcaaatcctaaagacataaagtgtatttacctata

cgaagaaaacatcaacaaggaaaatttcgctaaatcactgatacagcagtaggatcttgc

acacataataagaacaggatatgaaatactggacttgcaagagccagctcctgcaaactt

ccaggcaaggtgtgagtagtgggagtct

>contig00019 length=792 numreads=16

cACCGTCGCTCGcGcACCATGTTCATACGGCTGGTCACCAAAaCACCGTCACTACCAAAT

CACcGTCGCTCGCGcACcAGCaGCAAGCAGTTGTCTAGGAACTATCTTCCTGATCcGACC

cGCTCTTCGAGAGAAACCGCTCGACCGCCAACCTCTGGAAACTGGAAAGTTCTCGAGCAG

CAATCCGACAAAGCTGAACACATTGGAAATACTCGGtAACAGAAAATTGCCcTACTGTAT

GCGAGGTGATTACATtGTATGATTGACtATCGAAaGCAaTCGTCATAACGCATCGTGCTT

CCGTCTCTTGAACCTTGTCAGGATCAATAACATGCTGACCATCGCTTGTGATAGCGCAcG

TGGTaGcAAtCACcAtcGATttcaTTGGAaTgCCAGCATGCATGAGcGCCATGCAAGCAC

CGTTGATCAAACATGACAAGAgAGATCCTGAatCCTGCgTTaTTTGAAGAACAaTTtgAa

tggCGgATCGAGGGTGGAGGTTTGTAAGAACGATCTCCTCGCACGTGCCACTGAGAATCG

CTTCGATAGCCCGTTCCTTGCATCCAGGCTtGGTTTGTCTTGGGCTTGACGATACAATCG

ATTGTGGCCTTAtcAACAATCTCCCGACCACCTTTCACTTCGGCAGGGCCATACACTCCA

CAAATAACCTTGGTATCACCCTGCTTGAAGATGACTGATCCGTCAGGCTTCTCCACGGAG

GCAAGTTCACAGTTCAGCgccctcatggcaagtcaagcaacactattctcttaaaaccat

aatctaccccta

>contig00020 length=180 numreads=1

ctatacacatgaggatgccatgcataggggatttaatgaaagtgcttggggtatgaatat

atccttagacttttgtcgatcaggccggccggtcgtttgaaacgaacctgacttggcaaa

agtttttgactggccgtcatttgtgctgatcgtggagcaagtcctacaagaatgtgtcaa

>contig00021 length=202 numreads=3

AAGCAGTGGTATCAACGCAGAGTTACTTTTTTTTtTTTTTTTTTTGtAaCGGTAGTAACA

ATCGCtttttttCtAAtttaTTTCttAGCAACAaGTCTGCCCCgATTTAAtCGGGAgATA

aCATCGACtCCcGgTTtGCAaGCAcTGGAGAGGcaTGAAaTGCaGGAGGggAAgCcGAAA

GAaGGGCAGATcAtGTgTTacA

>contig00022 length=180 numreads=1

caaacttcttggatcttgaaatctgcttatgcactcagttgtcgtgaagttttgttgctc

cggtgatctgtaaagctttcaaggatatagtggaaatttgcttgaccgtgtttcttcaaa

atgctcgaagataccattttaatcctcttcattgccatcgccacagccctattcagtgaa

>contig00023 length=185 numreads=1

gtataacgtattctccaatctgaagtccataagtcaatacaaacaaagtatgctataaat

tgtcgctggatcaaacggttcttccaacccttgctgcagcaaaactccttccctaaatga

acttgatccacaagtaattggctcatggtcagactcccagatgtatctctgaccgtagct

tggca

>contig00024 length=377 numreads=7

AAACTTGcGGTGCCGGGTAAATCGAGAACTCGAGCTTAGACTTCTTGCCGTAGTCAaCTG

AAAGGCGCTCCATAAGAaGCGACGAGAAACCAGAGCCAGTACCTCcTCCGAAGGAgTGGA

AGATCATGAaCCCcTGAAGgCCAGTGcACTGGTCGgCAAGCTTACGTATACGATCGAGCA

CCAGgTCGATGTATTCCTTGCCGACTGTGTAGTGACCACGAGCGTAGTTGTTGGCGGCAT

CTTCCTTGCCGGTGAtCAGcTGCTCCGGGTGGAACAACTGACGGTATGTGCCAGACCGCA

CCTCGTCTACGACGCTAGGCTCCAGGTCAACGAAAACAGCACGGGGGACATGCTTGCCAG

CTCCTGTTTCGCTAAAg

>contig00025 length=243 numreads=12

gACGAGACAATTTgccAaTCAAGCGGTTGAGATTGGTGTAAGTAGGCCGTTCGATGTCCA

AGTTGCGCCTGCAGATATCGTATATGGCTTCGTTGTCGACCATgAAGgCGCAGTCCGAGT

GCTCCAGAGTGGTATGCGTGGTCAGGATTGAATTGTACGGCTCGACGACcGgCAGTGGAT

ATCTGCGGTGAcGGATAGATGGCGAACTCCAGCTTGGATttCttGCCATAGTCGACCGAG

AGg

>contig00026 length=1699 numreads=143

AAaGACcTcGGCtgTGCcaCcTCGGCAAaTGgTTtCTaCTCTGTtGACAaTTTGTGCcAa

TCTTACTACCAGTGcTTGAACggTTACAGGACAGATTTCtCTTGCGCGGCTGGTACTtAC

ATtAAtGCcGGTACCGgCACGTGTCAgTcGAcTTtGCCAGCTGGCTGTCAAGGTGGATTT

TGCACTGGACTTGCGAACGGAATTTACGTaGCcAATGAgATTTGCACAGCGTaTTAcaCT

TgCGTtAGCGGCAACGCCACTTtGACGCGCTGCCCGACCAATACCtATTTtAACTCAAcT

tCGTtGTCAtGTGGCACCAgTCGGCCCATTAATtGCAAAGACCCTGCCTGTATTGGAGCC

AGCAACGGTTTTTACGTTGCCGATGCCTTCTGTAAGAGCTACTACAGGTGCTTAAATGAA

TTCCGCACGGATTTCAATTGCCcGTCCGGTCAGTTCTTCAATATTGCCACTGGACAATGC

CAGAGCACATTGCCTGCCGGATGTCAAGCTGCCAGTTTCTGTGACGGTAGACCGAATGGC

TTCTACCGTGTCGACGATAAGTGCGCTGCGTACTACTCCTGCTCCGGCAATGTCGGAAAT

TTAGTCAACTGTCCGTCGGGACAGTTCTTCAACACGCTAACCGGTGCGTGTCAGACGGCC

TTGCCGGACAACTGTCAGGATCCCATTTGCATAAGTAATGGAGTCGGTTACCAATGGGCG

ATCAACGGTGACAGCTGCAGCTCTGCTTACTACGCCTGCTCGTCGCAGCTGTACCGGCTT

ACTTCCCAGTGCCCCCTCTCGTCAGGCGGCAATTACTTTAATtCGGTCACGCGGGTTTGC

ACCTCAGGAACTGTACCCAGCAGTTGCGCGGCAGCTCGACTAGATTACGTTtGCATCGGT

AAGACGAACGGTAACTACTTGGATCCTACCAGTGCCTCGTGTAGCAATTATTTCGCTTGT

GCGAGTCAAGCTGGAACTAGAGCAACGTGTGGCGGCAGCACTATCTACTTCAGCTCAGCC

TTGTCGCAGTGTGTGTCAACCGCCCCCGCTGGATGCTCTGTCTCCTTCACGTGCAGTGGG

CGTAGCAACTTCGATCGCATTCCGCACGAGACTGACTGCACGAAGTACTATGAATGTCAA

AGTGGTCTTGGAAACCTGAAGTCTTGCCCTAGCGCTTCGCTCTTCCCGTTCTCGACTGCC

AACGGAAGGTTCTTCTCGCCGTACTTTAAGACGTGCGTGAACACTACGACAGCTACCTGT

CCCAAGGCTGCGAGTACGAACGCTCAAGCGTGTTCCGAGATTTCCGATTACACCGTATTT

GGTTTCACCGGCACAAAACTACCCGACGTCACCAACTGCCGGCGATGGATCACTTGTGCT

GGTCAGCAGGTTATCAGCGCGGGTTTCGGAGACTGCGTCAGCCAGCCTTTCGATCCGGAT

AATCAGTTCTGTAGCAGCAGCTTCCGGTGCATTTTCCCGATtCCTTAAACCGGTCGCACT

TCAACGATGCATGGGCGTAGCTGTTGCGATAGCGACTGAGGgTTTTATTtGAGCTATTAA

CCATGCtGGTTGAACTTGGCCTCGCAAAATTGAATGGGTAAAACTTTGCTTTGCAtCTTT

TTTtGTGAGTTAaTAATaTGTGTTCAAaCTTTTGTATACTTAAGGTTGTATCGTAAATAT

aTCTGTTGTCGTtGAtcac

>contig00027 length=188 numreads=5

GTCGCACcAAACAGATGCGATTCCAaGCAAGGCcAAtCcAGCGCCAAGATTCATAAACaG

TGGCACCACACTAAATTTTCCAGCCTTTCCGTTGACAaGAaTTTCAAaTTTCATtCcgTA

AGCCTTCgTCAGGATTCTGTACTGCCTGCCATCAaCTGCGTAGTATGTCGGGTATCTAAA

GTTGTAGC

>contig00028 length=1504 numreads=149

taGATATAAaCAGCTTCAGCACCCAAGGAGCTAACTATGGTTCGGCCAAAAAAACGAGCA

AGCGAATATCTTGTAACAAAAGATATAAAGTCATCAAAAaGGTCAAGgAaCATCACAGAA

AAGTACGGAAGGAAGAAAAaCATAAATCCATTGCTAAAAGGAAGCCAAAAGATCCAGGCA

TTCCAAACtCTTAtCcATTCAAAGAACAGCTTTtAAAGCAAAtcAGGAGAAGAGGGAAAa

GgAAGCCGAAGAAAGAAAAAAGAAGAAGGAAAATGCTACTAAAGAGAGCAGGAAAAGGAA

ATTGCAAGATTtGAAAAAAGATGCTGAAAATCGAGGCAGGGAGTTTGACAAGAAGCAGTT

GGTGGATCGTAACgTGgATTCAAAGGCTGGTTCTGTGGAAACGTCCAGAAAATCGTTTTA

TAAGGAATTtAAAAAGGTTGTTGATGCAGCGGATGTAGTGATTGAAGTCCTTGATGCTAG

AGATCCTCTCGGCTGCCGGTGTCTTCAGTTAGAAGAGATGGTCATGTCATCAGGACCTAA

CAAAAAGTTGCTTCTCTTGTTGAATAAAATTGACCTTATTCCTAAGGACATTGTCCAAGA

ATGGCTTAAGTACTTGAgaCGAGAATTGCCAgCAATCGCATTTAAAGCTTCgACTCAGAC

GCAACGAAACAAACTGGCCCGCAGTAAGGTTTCCGTCGAAGTGGCATCGGAGGACATGCT

CAAGACCAGTGCATGTATAGGATCGAATACCCTGATGAAACTTCTCGGCAATTACTGTCG

AAATTTGGATATAAAGACGGCGATTACAGTTGGGATTGTTGGATTTCCAAACGTGGGGAA

GAGTAGCGTGATTAACAGCTTGAAGCGGTCTCGGGCTTGCAGTGTTGGTGCTACGCCGGG

CATGACGAGAACGATGCAGGAAGTACAGTTaGACAAGTACGTAAAGCTGCTTGACAGTCC

TGGCATTGTAATGGCCACGGGATCAAGtGATACGCAAATTATTCTTCGtAATGCcGTAAa

GGTCGAGCAAATCGAAGACCCTTCCGTGCCTGTGGAGGCCATATTAAAGCGATGCAACAA

AAACCAAGTAATGGAGAAGTATTGCATTGCGGACTACAAAGATACAAGCGAATTTCTAAG

TCTCCTAAGCATCCGATTGGGAAAACTAAAGAAAGGTGGCGTTCCTGACACTCACGCTGC

TGCTCGTGTATTGCTCCAGGATTGGAATAGTGGCAAGATCACGTTTTACACACACCCGCC

GGAGCGACCGATTGACGCGGAGCATGAAAGTACCCAAATCGTTCCTTACCTCGGTGCAGG

ATTCGACCTGAATGCCATAGCCAAGGAGGAAGCAGACGATATGAAGGGACTTCTTGAGGC

GCTAGGGTCATCCCTGGTCCTGGATCCAGGCAAGCCGGGAGTCATGGAAACCGAGCCAGT

TGtGCTtaGCGAAGATGAAAACGAATCCAaTGCAGACTTGGATAGTCGGCCTGCTGATCA

CGAC

>contig00029 length=401 numreads=8

gACAACGATGCAGGATGCTCCGATACCGTTCTTGTATCAGCGCGGACAACACCAATCCGC

GCTCCATCTTCGGCACCCCACTGTACATtGCCATAacTTAaTACAGCAAATGCTGCACAG

CCATCAGTTACATAaGCAaGCTGGAAGCTATTgCGCGGgtAAAAGCAtCCgCAAGGAGCc

GCGTttGCCCATtCCaCGATGAGCATGTAGTCAGCGTTCAaTCCTGACAAtCCGGcGcTT

TGAaCAACGgtCGaTGCGgCCAGGgATAGCTCACTTGGaaTTTCaGAAAAGCGATAACTC

ACGGTtCCCAGATGATAGCCGTTTATATCTGCCCAgAAaGGTGCGATGAAGGatACTCCC

CTCTCAAaCGAAGgCCAACGgTCGACATCAAATGACTGAAA

>contig00030 length=173 numreads=1

ccctcttcgttgcagcctacctaatttataggaaaagttatgaatccactaacaaatgtg

cggaatatttcaaagcttaatgaccaagacttaggtagtggaagatttgagtatcaccat

acgtggcattaccaatacaaagatagtgcatatatattcatcggtgggctgcc

>contig00031 length=692 numreads=73

AAGCAGTGGTATCAACGCAGAGTtACTTTTTTTTTTTTTTTTTTTGTTTTTTtttGtCAC

AAGGCAATTATTTTACTTGGTTTAACTGGTGAGAATACAATTAGgAAAAACGACATCTAC

AGGCCGTCGAACATGTTGAGGGACGTACGTGAGCAACCGCGATAAAaCCcTTGACAGAAA

CCTAACAGCTTGCGCTCAAGAAATCTTGGCTCAGCATCGTCCCTTAcGAATGAAGCATGC

GCACCTGTTCTtCAAAGACAaTCATAGACGACCCGCAGGTACTTTGACACTTTTGGACAC

GGATCGCCAAAGATTGAAGTCCATGGCATCAATACGCATGACACCTTGTTAGTGCACGTC

TTAATTTTGTCGAAGGAgTTCTCGGCTCTGCAGCGTAGATtaGTCGTGAAaTTACCGTAC

TGGCACACTTTATCCGAGAACCTGCCGTAGAAAACgTCGATAATGGCGACCTTTTGCGGA

TACGTGCAACTGATGACTGTCATGTTGTTGTCGCAGACAaGAACACTGTTCATGGAtAGG

CTCAGCTCTTTGCATGTCCTTTGGTCTTTGTCCAAATAAAATTGATCGGGACAACTGCAT

AGGTAGGAGCCTTGCACGTTGTGACAGAGATCTGAGCAACCACCATTATCAGTCAAACAT

TCGTTAACATCTTCGCATTTTTTTGCTGTTGC

>contig00032 length=180 numreads=3

TGGTTtGGTACAgTGgCGAGCATATaGCAGTATCCCcTCCTACGGCATCATCATGCAAgA

GGTTGgTAGGAaGAATGTTACAGCTGTAGCTGGGGTACACATCTTATGGACCATTCCGCA

ATTTGTGTTCCTTGGTATTAGCgAAATTTTTGTTGGTCTAACAggtttaggcttagcctg

>contig00033 length=113 numreads=23

CTACTAAAATGGCGGCTTtCCCGAAAGGTAGTTTTAACAATTTTTTCCAGTTCAGTGTTG

GCGCGATCGCCCTTCATCTTGTTGGTTACGATATTCCAAGCCATGTTGTCGAT

>contig00034 length=113 numreads=9

GTAGCAAAaTGgCGgCTTTCCCGAAAGGTAGCTTTAACAATTTTTtCCAGTTCAGTCTTG

GCGCGATCGCCCTTCATCTTGTTGGTTACGATATTCCAAGCCATGCTGTCGAC

>contig00035 length=347 numreads=41

CAAGGATCTGAGGTAAATTCTTCCCATGATAGTTGCTGACATTTTCGCACAGGACGTGAA

GTCCGATTATGATCAGCGCCAGTTGACCACCATCAACGTTAGTGACACTTCTCGAGTTGT

TGATCATGATTTCGGAGAACCCTGATCGCGCTCGATCAAGAGTCTCGCTGCAAATAGACT

TATCGCGAACATAACCAGCTGCgTGCAAATGCcaCAAAaCcGCTCCAGTAATATGTGGAC

TGAACCACATACCGTTACTTTTCTGCTGCTGCCGAAGCCATTCGATACCTCGTGCCCGGG

CCAGAGTTACATTTtGCAACAACTCCTTGCCAGCATTCAGACAGGga

>contig00036 length=1132 numreads=48

TgCAAAAATGAGGgAATCAAAaaTTGCAAAGCAGCCGGggAATCCTGTTTTGGCATGCCA

AATCAaTTTAGAAAAGAATTTTGCGTTTCTTGAGTTTCGgTCtGTGGAAGAGACAACGCA

AGCAATGGCGTTCGACGGTATAATGCTCCAGGGgCAGGCACTAAAAATTCGCAGGCCGAA

AGATtACCAGCCAATCCCAGGCATCACAGAAGCTCAAGCTACACATATTCCTGgtGTTGT

GTCTACTGTGGTGCCTGACACtATCCACAAGATATTCGTCGGCGGGTTGCCgAaCTATTt

AAATGAAGATCAGgTAAAaGAGCTCTTATCGTCATTCGGTGAGCTGAGAGCATTCAACCT

TGTCAAAGACAGTGCAACTGGTTTGTCGAAAGGTTATGCATTCTGTGAATATGTCGATGT

TGGGATGACGGACGTGGCTATTACAGGAATGAATGGTATGCAACTTGGTGATAAGAAGTT

AATAGTTCAGCGGGCAAGTGTGGGTGCAAAGCAAATGACAACGCAGCTTGCTATACCGGG

TCTTGATATCAGTCGAGAGGTAATGGCGACCAACATCCTTTGCCTAATGAATATGATTAC

ACCTGAGGAGCTAGTTGACGACGAAGAGTATGATGAGATTTTCGATGACATTCGGGAGGA

ATGTTCAAAATATGGACGCATTCGCAGTCTTCATATTCCTCGGCCTAACACTGACTTCCA

AGTACCTGGTGTTGGAAaGAtCTttATCGAATATGCCACTTCTAGTGATGCCAAGTCTGC

TAGCGAAGCCCTAGCTGGTCGCAAGTTCGCAAGTCGTGTCGTAGTGACTGCGTACTATGA

TCCGGATCTATATGCAAGGCAGGAGTTTCAGTAaGCTCCAGgTGGGATCGAGGTCTATCT

ACAGTTATCTGCTCTTTAATGGATtGaGTTCTCGGAGCCTcTAATACTCATCTGTGTTAA

ACGGACcAGTtGTCACTGTGACCATAGCCTGAATAAGAAGTTGAAGtACCcTCGACGCTT

TGTTTgtGGGCatCACcTAAAGGCCAtCTCCtGTTcGCTTTTttGAGGAGaTTTTTGctt

tgaacagacaactctatttgttacttcttacatatatagagtcagcgtgaac

>contig00037 length=239 numreads=7

tGAACGTCGCgCcGGTaCTGgAGTTAGGCGATcAAAgagACCGACCATGTGGGATGTtCC

tccAAAAGgCTATGAAGaTATTACACCGGTGCAGTTTAAagCCTTGCGAGCGgCCGgTcA

GgTGGAGACGCCgACgCcTCTTTCTGGTAGTGCGGTCCCTGCTGTAGCTTTGCCACAGGG

GGCTCAAATGACTCGCCAAGCCAGAAGACTTTACTGTGGAAaTCTGCcTTTTGGTATAA

>contig00038 length=459 numreads=6

CTTTCTTGCTCTGCGGTGTAACCAAAAACATAAGAGTCGTGATCGCATTCAGGACGGTTT

CCTCGCATGAGCTGgACAAGCAACTTatAACGTGGTCCACACCATCATTTGCCAAGATGT

GTTCCTGATTggCTTTATCCAAACATAGATTGCAAATTCCAGCAAGACCGAATTCCAcAA

ATTTTTCgCATCTTCagtgagcaTGTCCAGGAATAgaTCAATAACATTtCAGCTGTCGAA

GGTATTCATAATTAATAGGATCGTATGCGAAATTaGCcAagTTAGCCAGCACTTGGTACT

TGtGCTCTcTGTtGTCCGTaTCTTGAAACTCAGTCAcTAGCGACTGAAgAAaCTCAAATC

GACCGCGACCAcTCTTTgCTCcTTTctCTTCGATGTAgtCTTTGtCaGAAAACaTGGtCC

AGCTAGAAAgCTAGcTTTCTTTAATGAAGCCACttttca

>contig00039 length=197 numreads=4

AAGCAGTGGTATCAACGCAGAGTACGCGGGGACGGATAAGTCTCTTGTTTATTATCTTTT

GAGGGACTAAGGAAGTTCAAAATAAGAGTTGCGGTCTTGATTGGAATATCAGCATGGGGT

GATTTtCcGTAGAATGGTTGAACTACATGGCGATGACagCATtCTCgAAACGAAATGGAT

ATGCtgtaaatttctaa

>contig00040 length=413 numreads=7

ATAaGCACACGAGcAaTGTCAGCActCACTCCAATATTCCCACCTGAGGCAGGGgATCCT

GTCATATAGTAAAGCACAGACTCCCAACGCTCCTTTGCATACTTATCAAGGAaGGGAATA

TCCTTtGCGTGCTTTTCTTCTACAGCTTTTAAGGGCTCATTATCGACAACTCCACCTCCA

CATAATCCAacTCGTAAGTTCTTTTTGAAaTTGAAATTTACTTCAAaCCTTTTtAAAACA

TCACCTGCACCCTcAATCCAAATTtgCAatCcTTTTAGTTtATtGACAGcaaCaTtATGA

AGTTGAATGCTTTCCTTTGCAACCCacctggcaagagtagcttctggtataggctgctcg

ataaacagagttctcataacataatgctttgcaagttcagggagtccacggaa

>contig00041 length=224 numreads=3

gAaTGGgAGTTgAGAAAAGCtGGCCAGTGGCtGACAGCGTACTTGgAATGGAGTTCGCAG

aTttctAcAAgCTGTCCACcTTTATTTCcGCTGGAaCCTTCGGGGACTtCtACGcaTGCG

TGTCGaCAAaCTCtgaacaaaagtacaatctggcagtcaaggttattcgtgtcgagcgcg

ataagaagaccggatgcgagctacaatcgagaggcgacaccgaa

>contig00042 length=160 numreads=1

agtcactgattaatctactctcgaagcacaccttaaataagtacacaattttggacacac

ctgtttaaagccgataacaagtcacaaataaaatcgtaacacacatggagggaaggcaag

gtcaagaaagtttaagttttgcaaattattttcagctaca

>contig00043 length=371 numreads=18

GGGAATGATTCCATCACAGCCACCACCCATGTCAATGGGTATGATGCCTCCtGGTGCTGG

AGCATTTAcACCCCCTGCATTTTCAGTtGCTGGATTAGTTCCGCCTCCTGGTATgCCACc

TGGAaTGCcTCCTGGTTTGCcaCcACTTCCGTTTCAGCcTATGCATGGTGGGATGCAACC

ACCATTACcAATGCATGGCATTCCAGGTATGCCGCCAATGATGGCCTTAGCTACCATGAT

GCAACcTCcACCTATTCCGGTGATGGCTGCCGTCGAACAGCCtCCCGACCCTAAGGAtAT

GATTCAAGATAaGGAGGAGAAATCCTCTGATAGAGAGGATGATGACAAAgATGATTCTGG

ATCGCCAGAAc

>contig00044 length=1045 numreads=77

AAGCAGTGGTATCAACGCAGAGTACGCGGGGCTTtCCGACGATATCAACAGCGAAGTTGC

CCTAAAACCAGTGCTACATTTTAAGCCTAGACTAACAACGAaTTAtGGgTTTtGTCAAAG

TCATCAAAAACAAGGCGTATTTTAAGCGCTTCCAAGTGAAATTCAAGAGgCGAAGAGAaG

GTAAAaCTGACTACTACGCCCGAAAGCGTCTTACCACCcAAGACAAAAaCAAGTACAaCA

CTCcGAAGTATCGCTTAATAGTTCGTTTtACGAACAAAGATGTTATATGCCAAGTAGCCT

ATGCTAAAATAGAGGgAGACGTGATTGTATGTGCTGCATACTCCCACGAATTGCCGAAGT

ACGGTGTGAAGGTTGGTTTAACTAACTATGCTGCATCGTACTGCACTGGGTTGTTGGTAG

CACGTCGATTGCTGCACAAGCTGAAACTAAACGAGACCTACAAAGGACAAGAGCAGGTAG

ATGGAGGGGAGTACAATGTGGAAAGTGTTGCGGATGGCCCTGGTGCTTTTAGATGTTACT

TGGATACTGGGCTTAACAGAACAACcACTGGTGCACGAGTTTTTGGAGCTCTGAAAGGCG

CTGTTGATGGTGGACTaGAAATTCCTCATAGCACTCGCCGATTCCCAGGATTTGATGAAG

AGGCCAAGGAGTTTAAGGCAGAAGTTCACAGAAATCATATATTTGGAAAGAATATTGCTG

CATACATGGAGTCCCTGCAAGAAGAcGAGGaGgCaTtCAAGCGTCAGTTTTCTCAATACA

TCAAGGAAGGgATAACACCTGATACTATTGAGGACATGTACAAGAAATGTCATGCAGCTA

TCCGGGCTGATCCCACTCACGCATCGAAGgAcAaGgCCAaGAaTtaCAaGGGAAAGAGaT

TTGgCCGAAAGAAGTtGTCCAaGGCGCAGCGAGACAATCGCGTTGCTCAGAAAAAGGCAG

CTTTCTTGAAAaaGATGGAGGAAGGGGgAGAAGAaTAACTTTTtATTCTGATCTGCAAAA

GAAATTTTTGgAGTCCTgAACAAAA

>contig00045 length=186 numreads=1

gacagcagacaagaaaatgagggtcactttagtcactcttggcactacagtatctacagt

tgcagtggtggcaaatgctttttttcagaagaagcagttctatccgtctgttgtttacct

tactaaatctaatcccagtatggctgtcatgtatgcacaggttatttgttttggtgtttc

ttgtta

>contig00046 length=456 numreads=5

aataaattgtacccaagtcattttttcagattgattGGGTGAATCCAAATGGCTGACAAA

GGGCAGGCGAAGTATTCAGCTCTGCCATTTATAGATGCAAGTGGAAGGGATGTGTTTGAA

ACtGgAGAGGAATTGAAACCTGATGCTAGAgACAAaaTtGATTTCTCAAGCAGTGACGAA

ATTGAAAGGATAAGCTTGAACCAcAAGGgaTCATTTAAtGTGTtCaGTGACAAATaTCTT

GaTtCTTCTAAtATtGaTTtttCTGACCGCATtcACCCCACGCATAAAAGTGGATACGAG

ACAAagACTATATTtGAAatAATtGGAGAGCAAGTAgCcAAGGAATCCCCTCAGCAAAAG

TACCAACGCCTCCAACaTGAGCTtCGGGAACTGGGAGAAGAAGTTTCtCAAATAACAGGC

GCAATGCAGTccccTGAGAGTATATCAGCATTAAGt

>contig00047 length=1447 numreads=49

CTAATGTGCTACGTCAAGTCAAGTCTTTCAAACCAATTCTCATTCATAGTAAATCTTGGA

ACTGCCAATATAATGAAAACAACAAAACTGATCACGCGAAATTTGGCTGCATATTCTATT

TaTCTGTCACgTTCATATTATGCCATGCCCTTCAAAAACACaCGCTACCAAATTAACCTA

CCACCAAAAACTAACCGAAAAGCTGACTGCAAGGGAGCGAACATAaTGCCACcACTTTGG

AGGgAaTGTAAAGCATTTCACCCTCTtGAAGGACGCACTCAAAAaTATGGGAGACCATTA

AACAACGGATACTTTTCCACGTCGGGATTCTCGACATCGACCCGGCTGCTATTGTGAAGC

ATtGGCTCATCGAACGGGTATAAACATGACGACAGTGCCTTGTCATATAATCGGATGTAC

TTTtGACcAACAACTtGAaCGAAAAGATTATGATAAGGGTCGTGATGTAGAGGAGATACT

GTCCCCTTGGGTCCAAGCCAGGCATTGATGATAACTCGGGAGTCATCTGACCcACCCAGG

CAACagTAGTCTGGAATTTGAATGTCATCTCTTAATTCAGATACCTGGTCAAACAACTCA

TGCTGcGCTAAGTACCcACGTTCAGCAAGCTtcGAGTCACTTACAACATAGtcATCGAGG

AACTCATTCATGGTCATAAGCCTCTGTGTCCAATCTTCCGAAGTATACCTGGTGCCTAAT

TCAATAGGCACAGTACGGTGTCCAGCTACTTGCCTGATGTAGTGTGTACTCCATCGGCGG

gTCGTAAACGCAGGCCAGTGTGAAACCGCGTCTCGAATTACGATAGGAGTCTCTGTAGCG

ATAAAATCTCTCTGAAATTTCAACAATGACGGAGAACTTATGGTtAATAGAGGATTCACA

ACTTTCGAATCaCcAAGGTtGTCcTtACTtcGCACATGTTTCGTAAAGTCTTCAAAGTCT

TCACAAAGATCTATGCCTTGATTACACACATCCATCTTTGAATCAAACGCCATGTTCTTG

GTCATATCGCGGTCATGGACTTTAGATAGCCATGTAACAAaTCGGGAAAGAaTGTTATCT

AaTATGGGTgCTCCCAAAAGCAGTCCCATGTCACAAGCTATCGTTGCTTCAGAGTATTTA

CCAATATTGATCAAGATAaTGGCCTTATATAGCGAAGCCAAAGAGTAAGCGTGTCTCCAT

GTTATGTCTACATCCTTCCAATAaCcagTATTAaGCcTTtCCCAAaTGCaaTCcAAAACA

TACTGACAATGGCCCAAACCTTtGCTGAATTGCTTTGAAAGAATCAaGTCAAGCACTTGG

AAAGAAGCtCGGTGACGACCTGACCTCCAAGgTTGCATAGCTTGGATAGCTGTTCCCGTG

CTTCGTAGCTTTtCACCTCCATGAAAACAAATAAAACGTCGCTTGCAACAAAGTCTGCCA

TTtCTAA

>contig00048 length=443 numreads=8

aTCCTCAAAttCCTCCtCAgCATcaGGGTAGggCCGTGAGcAaGtGcAGTACTTGCCGAA

AAAGTTCTGGCTATAGCAATTATCCGTGTTGAGCGGATCCTTGTTCTCCtgCAaTCGGCa

cTTCTCAGAAAATTTGGTGTTACCACAGTCGCATCggAAATTTcgtttggtatagagctc

cacaagttcatgcccatcatgacaatgaatagaacaggccagacaaactccagccacttt

ctcctttgttgagttgcaCgTtagACAGGAGtaCAAaGCTTGCCGgtcGACATaGCCCGa

GTtGtAaCTACATTGGGAaGCATtAcTACcACCTAGAATAGCAACAGcATCCTCGTGCTC

TTGCTCCTTCTGAGTAaGGAACTCCCCCAAaGgTgAggACTTCATCTTCATCGTCATTCA

TTTCTCCCTAAGTTACTACTATT

>contig00049 length=188 numreads=1

tcaccaaggtcttattggttttcttgagaaagctggggaagtagggctgcatatcgaaga

cttggcaaaacttctgccaaccttttctcgaaaagatttacttaatggactctatgactt

acatcacaagggtgaattgacacgaacgccaggcaatatatggaagctgcttcacatatc

acaagtag

>contig00050 length=583 numreads=3

cggcagaagcaaaaagttatccgccgcattaacagagaaacaagtataacctaagtcaca

aaggtttgtcatcactggcgataaagaacactagtgagttgcagacatacgaagtaagaa

gatgacatgcttgaccggtacaaactgttaccaactttcccaaagaaaagtcgtactgca

aagaaaaggtactcccaaagaaaaggtactgtcaacaaggacggcattacagaataaacG

CACTCCTTCCttttGATGAAGAAAGCAACTCCGAACACAtaaacagcttagcgtaaagaa

gcttggaaaatgtaatgacaccatataacttccgaactcctcaacacgctaacaatacga

ttggctgtcactcgctaatgaaaactcgcctaggtaaaaataaggtCTCGACCAAAAAtt

ttggCAATacATATAAGCACCTATCAACGATTGTGTCAGAGTTCAGCTTTCGGCAAGTCA

CCCtcACTTGaGACCTCACTggcaacaccagcgtggctactgtccgaaggctgatcggca

ctgtggagttgcggtttgctcgatagctgactagctattgtat

>contig00051 length=181 numreads=2

cTGAGAATATTTATACATAACTATAGGTTTTATATATCTATTATAATTAATTATATTCGT

CAATTTATGTTACTTTTATCTATGGTTATAAGTTAACCTAAAGGCTTGAGGTAAATGAGT

CTTGATTTTCAATATTGATAAGCGCAgcagaagtcaaaagtggggaagaattttgaccta

c

>contig00052 length=325 numreads=4

AAGCAGTGGTATCAACGCAGAGTACGCGGGGACGGATAAGTCTCTTGTTTATTATCCTCT

GAGAGACTAAGTTGCAGTTAGCAATTTGTAGGTTGGTTCTTGAAGTTGCTATGTTTCAAA

GTCATATaTCcAAaGGTCgATCCTTTAtGGCTTACTATGGAtGAAgCTCTAGaTGCTATG

AgaCTGaCaTGTAGCGCAAcTGaCTACCaTGCTGCAGTGAagcTCTTGTATAcatatgtg

aagaatatagtcagtaatCCGTTTGAtGAAAGAATCAaGGAAAATCAGAATTGGAAATCC

AAAATTTCGAGATGTTATTTGGAAt

>contig00053 length=231 numreads=9

GACCAGCAATCAGGTTTTtAAgtCCCCAGCCGCAACGAAACCTTGCgCCTCTTAAATCTT

CTGgTCTCGAAAGAATAACTtGGAGgcGGAaTtcTTGAtCCTTGACGATAATGTAACCGT

CATGAAAGGTTTTCTtCACATCTTGCGGCACCAAGAGGAAaTGCTGGTCgTACGGGCTCA

CtGAAGCGACTGACATCCCAAAGTTTTTCCAAAATAACCGGACACAACAGT

>contig00054 length=794 numreads=8

ttttgaGAgCTtAAGCTTTTtGTTAAATAGAGAATGTCTATCGAAAAAaCTTATAACGAC

ACCTTCGATAGCGGTTTGGACCATAAACCTCGCATGGACGATCTGCTGCATTGTCTGCga

GAtAGACCCGaTTgCAAAGCTCGGgTtCtGAAtATAGGCAGTGGCCCTGGGGAATGTGCA

GGTGTGTTTGTTACAGCCGcttcaccctactgttcaaaagtaattttaacgaaccgaatc

aagtttcttgtgctACGTATCGCAAGATGAGTTGGTACGATGCCAATAAGGACAAgATTT

TtggCATGTGTggaaaaaTCGAGGACATACTTTCTGCAGGTGGTGTTGAGGATGAAAGTG

TCGACTATATCTTTCTCGAGCACGTTCTCTATTATTGGAGATtGTCCGAAATCCCgTTCa

tAATcAAAGgaCtATCAAAgCTGATGCcTGTTCAGGgCGGGCgTTTATTtGTtGgtCATt

CCAATGACaaTtCACCAaCTGTgAAAcagttcaggaaaattgatcccaattatcgccaca

tgaaggcttttaaagaaGCTTTGGATGAGGCTGGACTTGACTACAACATGCAAACAGATC

TTGTTCCCTACCGCTTTCGACGACCCAAAGAAATATTGGCGCTTGTCTTTGAAAGCAGCG

TTTTCTACCatgaagattacattaatgctggtgaaggacctctgtctgtggaacaccaaa

aaatgctcgatgcagcagttgatgcaattatagaaggatattcttcgccaattccaggat

caagcgatcacaca

>contig00055 length=411 numreads=15

AAGCAGTGGTATCAACGCAGAGTACttttttttttttttttttcgtttGACATTCGCGCA

CTTGATCTTTTCCTATATTTTTGACTAGAAGATCACAAATGTTTATTATCAAAGTCACCA

AAACCTTTTCAGCGCAAGcaTACTTTCGATCGCTAGACTTTCGCGGCGAAGcTTACgTcA

CCTcTTGAGAGATACCaTATCTTTGGCTATCTTCACTTTCGGATAGGTCTTGAGTTtACt

GTcAAaCAaCGCTCGACATTCcTCCATTGTGCTCGTTCGGTGGTCGGTACCGTGTTCcTG

ATAAGCAGCCATCACCTTCTTCCTTAGCTTCTTAACAGGCAGCTGGTTGTCGGTGGCCTC

CTTCAGAGCTCGCTTTATtGCCCTGTGCCAATGAAATTTTCCCTTATCATG

>contig00056 length=338 numreads=4

aagcagtggtatcaacgcagagtacgcggggACCCTTCCTGTTGCTGCGCTCTATTTCAG

GTGATCATTGGCAAGTTGCTTGCTGACTTGCACCGGtgCATTCTATTcattgttattgaa

tctAaGTCATGGAGCATCGTATTGAGTTGGAGAGGCGTGGCcAAaGATCCGCACGAGGTA

TTGGAGCtcAaTCTtGACAaCTGCCGTTCGACAaaTaTAGTGGGTTtAaCTGACGATTAT

GATAATCTGGAGTTGCTCTCACTAATCAATGTTGGCTTGACTACCCTAAAAGGATTCCCA

TCACttcctcgacttaccaagttggagctcagtgacaa

>contig00057 length=283 numreads=11

TCTGTAAGGCAATTTtATCAGGCTGCAGTCTCAACCATTTGCTGCCATGGATATACCACC

CAACCAAACAATATACATCAATAATCTGAaTGAAAAAATTAAAAAAGAAGATTTAAAGAA

ATCGCTGTATGCCATCTTTTCTCAATTTGGGCAAATATTAGATATTATTGCTCTTAAGAC

TCTAAaGATGCGTGGTCAAGCCTTTGTTGTATTTCGAGATATCCCTAGTGCTACTAATGC

GCTGCGTTCAATGCAGGGATTTCCTTTCTATGACAAGCCCATg

>contig00058 length=341 numreads=3

ggcattgacaaggacaatggatgtcgcaaaaagacacttcgattttgaaagctatacgcc

ttctagacggtccgctcaaagctctgcagtcGTaCCaTCTTAagActctCCTTTTtCACg

ctACgAagAATAAAGAGTtGCGCTGGAGCGAAAaGGATCTCGCTGATCGTGTATTCGGCT

TGATGCATGATCTTCAAATTGCGCTTGAAAAGCGAGATTTGAACAATTTCTTTGTTTCTA

AAATGAATTTGTTTGAATCCTTGCAGCCTGTAACAGTTGCAAATGTGGCCGATAGGAttc

gccgattgcttcgaaacgaagaagagtctatgagtgttatt

>contig00059 length=1475 numreads=41

GgCATGGGTGATGATAaCCGTGTTGGAaGCTTTGAACATTTTGATGCTGTGGACAGCCAT

CAGAATAATTCAGAAATGACTGAACAGAACAGGGAAATATTGGCTTCATTTCAGGAGATC

ACTGGTATtGAcAGTACAGATCTATGtGTTTCAAAGCTGGAaCAGCATTAgttggaattt

agaggctgctgtgcaaagtacttttaatGAAAACGAAaGGAaTTGCCATCAGTTTATAAC

GAGACAGGTCATGGGCAAGAGATGaatacgagctatcctgtagcaagttcaGATTCGCcA

CTTGGgCTTAGAAgAAGGCaCcATAGTCcTGgTTCcAGACAGTTTTCGGgtgCAGGTAGa

GCTGcATCTGATCTCATACGAcAAAATaCGTGGTGgCATTGGCTTGTTGGTACTGCATAT

TCACCTTATCGTCATATTATGACGTCGTtCCGTTTGACTTACCTTGCTGTATCCAGACTT

TTtCtCTTTATCGTGCGGTTtGTGTGGCCTAGCTATGGCAGAGCGATCACTCAACCTTCG

GAGGACGTAATATCGTTTCGAAGGCAGTTTGAAGaTCAGTACGGAACAACGCACCCTCCC

TTTTTATGGGAAGCTACAGTGAGGCGTTGGCTTCAGCGaagaaGGACCTGAAATTTTTGA

TCGTCTACCTTCATTGCAAGACTCACCAGGATACTGACGCATTTTGTAGGAGcGTtATGA

CAAACCtGGCTTTCgTTAaTTTTATGAGTGACAATCGCAtATTGTGGGCATGCGATGTGT

CGTCGAATGAAGGTTGTCGGgTTTCACGGgCTATGAAGGAgaCTACGTACCCTTTCCTTG

CAGTTGTCTGCCTGCGTGATAaCAGAATGACCATTGTTtGGCGTGTGGAAGGGTTCTTGC

CTGTTGATCATCTGATCGCCCTGTTGTCCCAAGTAGCAGATGACAATGAGCCTAGCTTGA

TCGCAGCTCGGCACGAAAGAAATGAGCGATCTCATGCGCAGATTCTTcGCAACGAGCAAG

ATGCTGCTTACCAAGCGTCTTTGCAAGCGGACAGAGAGAAGGAGGAACTGCGACGTCGCG

AGCAGGAGGCGGAACGAAAaGCCGAGgAAGACAGGCGgCACAAGGAGGTCTCGAAGCAGC

TTAAGAAACAGGAAATTTTGACAGAGAAGGCACGTTGtCGTAAAATCATGGAATCCCATT

GCGAGCcGaaTGACGCAGACTCGGTGAAGCTCCGTATTAAATTGCcGAACGGCTTGATTT

tGCAGAGGAACTTtCTTAAATCcGACATAaTTCAGCTCCTGTTCGACTACGTGATGTCTC

ATGAGGATtCTCCCGGCGAGTTCGTGCTGCGGTCAAACTTtCCTCGTGCATCCTATCATG

CAAaGGATTGTAGCGGAAAAACgCTCGAGCAGGTCGGGAtCACGTCtCCCGcTACCctGT

TTGTtCAGGATTTATTAGACGAAGACACTTCGGAc

>contig00060 length=307 numreads=5

CGaGCAgaggCCGCcTgCTACAGAGAAGAGCGAGATGTTCTTGTATTCGGCGACAAGCGG

TGGATTACGAAGCTGCATTACGCTTTTCAGGaTGAAAGCAATCTCtACTTTgtCaTGGAC

TATTACAGTGGTGGTGATCTCttaaCTCTGCTTAGCAAGTATGAGGACCATTTGCCCGAG

CACATGCTTAAATTCTATGTGGCGGAGATGGTGCTGGCAATAGACTCTATTCACAAGCTT

GGATATGtCcACAGAGATATCAAGCCTGACAATGTTTTGATTGACTTAACTGGTCATGTG

CGATTAg

>contig00061 length=290 numreads=4

tAGAATGGGTAAATGGTGCAATGAAATTCTTGGAAGCCACGTTAAAAACCGATCCACATC

CACCAAACCTTTGATTTTCGCATCCAtAAACAACATTAACTACTCCCACAATTCTCAAAG

CAGCAGCACACATAATGCAAGGCTCAGTAGTTACATAAAGCCAACACTGATCAAATACCG

ACGACTCCTTCTGATTATACCTCCGACACCATGCTcGAaCTtGgTcAATCACCAACaTTT

CAGCATGTCTTGTAGCGTTCTTAGTACGGTTTACATCATTACAGCCTTGT

>contig00062 length=128 numreads=2

GTAAAGATAGCTTTGCCTATTAACTGGGTTGATTTGTTTTAAATTTGGCTATTGAATAAT

GCATTGTGTTTCTTTTGGCAATAATTATGTTAGTTTtGGgTGTTTtGGgAATTTTtGGgA

ATTTAAGA

>contig00063 length=505 numreads=7

CAGTTCTTTTCAGTTGTCTTTAGGTTTTAGCGAACCGCATATTCCACATAAATTGGTgcA

AgTGACCAGGGATCATGGCCAAgCTGAaGACaCTGaGGaGAaaCTGgaCCCaCcAGGCAA

AGAaagCTGAtGCACTGATTCGCGAGGTAAGCgAGTTTCTCGCACAACCCCAGTTGCAAA

AaTCAGTTGCCGATGATTATCGAATTGCtttGAAGGACAAGctGGGAGTCAtCGAGCACC

TGgCAATGGAACTCTCACCGCTGCTGGATTCAGACGATGAGGCCcAGgACCAGTtCtGCG

AGgAAtcAAACACCCTCGCAGACACCATTCGGGATGCCATGCGGAAACTGTACCTGAAAA

GCGCACAGGAACTCGCAAaGTAATCCAGATAGTACTACTTCCTTTGTTGATTTTATTACA

CAAACGCATACCACCCACCCGCTGCTTAATGCACAAGCTACCAACCCACCGCTAAaTGCA

CAaGCAAACGATCCGCCAACTTCCg

>contig00064 length=223 numreads=2

tACGTAAAATACCATGATTGTCTACAAAGAGTCTCAACTGTCTAACTAACGAGGTAGGTT

GTTCAGACAGTTCACGTAAATAATACcTtATACTGAACAGCACGAGTCCACAACaattct

gcgtcgctaatctcctttccggtcagcatgagcgtacgcgatttctttgttcgtaggcgt

tcgatgaaacgcttgacgtacgcggtgacccgtagtaatttgt

>contig00065 length=702 numreads=18

gatgcgtaaacgatgatgtctgtctaagatgaatagccgttggtcggtctgcttacttat

gcttgtgccgcgacaggctggactcttaagcttgAATAAGTTATTAATTGGTGTGAACAG

TTCTTCGCTCCACTCACTTCTGATTCCCGCGAGATGCAAaGCGAAATCCTCGTCTCCATT

TCCCCGGGaaGGTGGCTTGACTCGCATAcAGCAGCTGAGTGAAAAaTtCAATCCTCCTCC

GAAGATGAAGCCTGGCGAGACTCTGCCAAacGACATAGCAATCAAAATAGACAGCACCGG

ACAGATCTTTAGTTCgTACGTGCCGCTCGAGCGAAAGTCCTTTCTGCTCACAcTAGAGGG

TgCGAAGCAGCGCTGGGAaGCCCTCAAAGCATTGgTTGTTTCcAcTtACAGTATTGGAAC

GATCAAGCGATATGTCAAGCCTTTtAAAATCGCagATTTCGCGaGGTCTGCGCAGAAGCA

GTTCGTCGAAGTGAACAATGCGCTGCAAGTGGAGAACGTgTCTGCAGAGCAAGCCATCCG

AGAGAACGTGACTTtgaTTCTAGCTCGAGCCcTTAAAGCTCGCTTTCAAGATCCcGCTCG

AAAAGTGCACTGGCGTTTCGTCTCGGAAGTCGAGAGACCCAGACTtGgTGCACGCGCAGG

TGGCCCCTGTTGCCGACAAGGACAATCTGTATGCTCAAGCAA

>contig00066 length=103 numreads=1

agcgctaacataattgccgtggggaaattgcaaaataatgctttaacagtaaagaaagta

agatatactaaaaaccgtgcttacctttttttaattctctggt

>contig00067 length=1318 numreads=67

GGACATCTGAGTTCCGatGTGATCTGGAGCTAAGTGGTATGGCGAATAAGaagATATTCT

TGCACATCTATCAGAAGACTCAGATCTATGCCAATAAGTACTGGAGCGATGCCGTGGACA

AGTACAACGtcATgAaGGAACAGTCcAAGGTCATGTACTCTGACGCCGTAAGGTCTGCAA

AGCAATACGTAGAGAAGGCTAAATCCatgtcaaaggaagcttacacgaaggctattgaga

tggtgatgagggcttacgagaatccgatcGCTGTTTACAACGATGTCTCAAAGGCGGTCG

TTATCTTCCatAAGGACACCTGCTCGAaGgCTtGgAAGatGTACGAAGaGTATGCTCCAc

TtGCtCGAGtAAgTgTCAaGAAATaCATGGAgCAGATGAAAATCAAGGCcTCCGAAAtGA

ACAGCcTGCTGGAGCCATACGCTtCTTCAGTtAGAGCTGCAGCcAGAAAAATTGCAAGCG

GAGAGTCAGTCAAGACGGTATTGAGCCCTGTCTACAATGACGCTATGCGTGCAGCCCGAC

AACAACTTCAAGCCCTCTACAGCCAAGTCGATGCCGCTCAGACCCAGATCTGCAaCATGg

ACcAGGAGCTATGCAACCATGTCACCTTGAGCTGGAAGATGCACAAGACccTTTTtAGCA

AGTACGCCGATCGCTTtACAaGCTACTACAACaTTgCcAAGATCCGCACTCTTCGAGCTT

TGCGCATGAGCCGGATCGCCCTGTCGATGCAGAAAAACAAaCTTTCCATGATGGGACCAA

CCTACCGTGCCGCTGCTATGATCTtcGGAAATAGCCATGTCCTCACCTTCGACCAAAAGT

ATTACGACTTTGTCGACTACAAGAATgAAAaGTGCACCTACGTCCTTGCTCGTGACTTCT

TAGATGGAAAGTTTACAGTCTACAGCCAaCAGAATAATCTCATCATCGAGGcAGCTGAtA

TGAAGGTGGTGGTTGCGTCAGATGGTActACCGTTACGACCATGGGCACACAGACGACAg

CCAGCCTGCCAGTgAaGTCCgaGTCTGGATCGTGTGAAAGGATGGGCCACGTAATCAAGT

GCTTCTTCATGGACCTGAAGTTTAAGGTGGTTATTGATCTGAAACACTTTGCTGCCGTGG

TGAGCCTTAGCGGATGGCATCATGGGAAAaCACAAGGTCTGCTAGGAACCAACAACCGTG

AGACGTACGACGAGTGGCGACTACCGAGCGGCAAGATATCATCTGACATATACGAATTCG

CTAATGCTTACGAAGTGACCGGAGAGGCTAAGTGTGTCGTGAGCAAGAGCTCATTCCG

>contig00068 length=793 numreads=56

GAAGCGCTCTACGCCCTGACTGAAGAGGCCAATGCCACCCTCTTTGTCTTCGACCAGCTG

CAGTTCAAGAGCGTCGGACAGCGAGTCATCATCGGCCAGACGACCACCAAGGCATACTCG

GACAGCAACTCTGAGTCGCCTCTACCCAGCACTGATTTGCCATCCAGCGAATTCAAGGAG

ATGGTTGAGGCTTCCAAGGGGGGATTGTTCTCGAACAAGATCAAGAAAGTCCACCGCGTC

GCCCATGCCATCTACGAGGGAATCAATGAGTGGTTGGATAAGGACAGCCAGCTTTGCAAG

AGATGTCGTCTTGATCCATGGTACTTCGATTCCCGTCCGATCTGCCAAGCCAAGGATGAC

GTCAAGTGCTAAGCGGGATCACAGAAGTCGCTTTTCTTAAACTCTGGAGGTCATTTTAAG

GACTACGTTTTGTAAACCCGCAGTAACCATTTGTTTCATGTGAATCATAGTCTTTtTTTA

TATAGTGATTTTttAATGAATTTTtGgTGCGTTTCTGTTTTGTTAATTTCTTTTCTCGtC

TGCGTCTGGCcGCACTTGGATGTAAATAAAGTCGTATAGCCTGGATTTGCGTCACGCTTG

AAAGGGaTGAAAGTTGCGGCACTAGATgTAAGtcTtAActCTGGTTCATGaCGTGTCACC

GtATGTCAGTTCGCTGGATTTGTTTACGTCTGCTGTGTTGGTTTtGTTTTAAATAACCAC

GCAGTGCAAActGTAACTTTGTATCGCGTGTTTTGCTTAATTTttCtACAGGTAAaTATT

AaGGCATGGTtaa

>contig00069 length=170 numreads=30

AGGACGCAGCGATAGTTAATGCCGGTCTCGACATCGAAATTGAATGGCCATCAAACGATG

GCGAGGCGACCATAGCTTCGCTACACTCGGTAACAAAGTCAGAGTCAGCTTCGTTAAGCA

GATCTACCAAAAACATCAGGTTGCTGTCAGATTCTGTCAGCTCCGAACTT

>contig00070 length=170 numreads=29

AAGACGCAGCGACGGTTAATGCCGGTCTCGACATCAAAaTCGAATGGCCATCAAACGATG

GCGAGGCGaCcATAGCTTCGTTACACTCGGTAACAAAGTCAGAGTCCGCTTCGTtAAGCA

GATCTACCAAAAaCATCAGGTTGCTGTCAGATTCTGTCAGCTCCGAACTC

>contig00071 length=656 numreads=8

ccgtctttaggtaatatccttacgtccgcacagttgacaaatgtttcttgctgtggATtG

aGCCCTACtCCGCAGACGCCTGGACTTGAGCAGCCCCAGTTATTtCcACAGTGGTACCAC

CACTGgAGAACGCAACGATCGCACGTCAGGTCAGCTGgCAGCCTTAGTGGGATCGTAGTG

GAAAaGGAATaGGGGGCCGTATCGATCGACcATCTGGTTCCGCCTCCAACAaTAAGCAAT

GTGTGCTTGAGgCTTtCTtGGGTGAGTGGCAGGACTCcTATGTCaCCAATGCGCCaaGTG

AaGTaTCCCCGATGGTTAGCACTGATTTGTAcTGTCACTTcGaTGTCTTgTCCTTGCGTG

TACGTGGCAGTAAtAaCTCCTGTGGCGTACTTTCCAGGATACACATACTCTCCAGtaTTG

TCAGCATTCCAAGGGTCACCGCAAACTCCACATTTGcccccGttgacattccattgtgtG

gCGAAaCCaCcgCaGTTCAGCtGGTTGTCGGTGTacTgTGgCGGAGtGCCgAAGCCCATT

cTCcACGCGTTGTTTCGAGATGCTGGCTTCACCATCATGCCATGCCCCCAAGTCATCGTC

CACCAGCCGGAGAGATGCAGCATGGCACTTAAAAACCTCAGAGTCGCCATAGTTCt

>contig00072 length=825 numreads=32

GAGACTAAGTCAAGGGAAAAAtAGCATGGCTCCTGTTCTTGGTTACTGGAAAaTCCGAGG

GTTGGCTCAGCCATtAAGGTTGTTGCTTGGATACACGAAGACCGATTTTGAAGATAAaCA

aTATGACCTTGGAGATGCCCcTGAATACAATGCAGATGGTTGGGCAGCTGACAAGTTTTC

GCTAGGATTGGACTTTCCGAATCTTCCATATTTCATTGATGGTGATTTTAAGCTAACACA

AAGCAATGCAATTCTGCGTCATCTTGGGCGCAAGTTTAAGTTGGATGGAAACACGGAGGA

GGAAAGAGCCCGTGTTGACTTGGCTGCTGAACAGGTGGTTGACTTCAAGGgCGGATTTGT

CAAGCTATGCTACGGCCCCAACTTCGAGGAAAACCTTAAAACcTACAAGACCTCTGTTAA

AGGCTTtCTGAAGcttcTTtGAAACCTTCCTCGGACACAAGTCATTTCTCATTGGCGACA

AGTTGACATACGTTGACTTCACTTTCTGGGAGGCCcTCGACGTCCACcTGgCGTTGGACA

GCACcaTCCTgAACGACTtCGaGCACCTGTCGGCGTATCACAAGAGGTTCCAGGAGGTTC

CTCAAATCGctGAGTTCATGAGCTCAGAGAAATTCTTCCGAGGACCTATCAACAACAAGA

TGGCCAGCTTCAAGTGATTtCTGACGACAAGTgAAGACGGGGGgACGTCGTGGGGTACAA

AAaTGCCACGAATCGaGTAGTTTGTTTTGGgTTCTGGATTTTGTGGTTGAAAATtAGTcg

AAaTtGAGgAATTAAaTATTGTTGCGttAAAaaaaaaaaaaaaaA

>contig00073 length=186 numreads=2

CTCTTGTAATGAGTACAAAAATGTATGTATTATGGAGATCCAATTGGCAATACGTCAAGG

CTGGGCGAAAACTGATGCTTCTTTCTGTAAGGAGTAAATCTAAGTGCAATTCAGACGTCA

GAAATCTTTTCGAATTGTATCAGAAAAATCCATCATGGACAGGACAGGTTGTCCAGCCtg

gaaaaa

>contig00074 length=410 numreads=34

AAGCAGTGGTATCAACGCAGAGTACGCGGGGGGCGCGTCAACAAGTGTTGGAAGCGAAGC

GAAAAGCGTCGCGGCAAGGTGGTCAGTCCttcgggattggtgttataggccgcgttCGGC

CAGCTCGcgTTCGACAGAGgTGTCaTTCTGGTGCAGCACATGCtCCctCgTTCGGGGGgg

CTGGCTGGCcTGTCTCGAGTCGGTTGTCGACcATGGCGGACTGCTTGCAGTGCGCCTGAA

CCGCGGTCGACTCTCTGTtCAGGTTtACGAAaGCACAAGTGTGCTAAGGTTGTTGGCGAT

AAAaTGTTTtCATGCGACCCGTCTTgAACACGGACCAAGGAGTCTAACATGTGTGCAAGT

CTTTGGGTGATTGAAACCCGCGAGACGTAATGAAAGTGAAGGCCCCTAGC

>contig00075 length=187 numreads=4

gTCCGGATCAAGTATCATGCGAGCTCGCCGCACTTATTCACCGGACGCAATCTTTTTGAA

GAGCcAGtCATTCGGAAGaCATAGAAGtCtGGTGCTGCTGCAaCAAAgttGAATTtCTcG

CccTGgTgACAATCAGCTATCATAATGTCAATTTTCAAACCAGATAGTCTGTTAAGAAGC

GTATGCT

>contig00076 length=732 numreads=8

aggaagtacagtggcgaaacaataactgtcgagtttaacatcaatgagaatatcaacgtg

gatgaagctggtgaggaagagggatcgacaaatgctgagaacgctCCTGCCAGCCAGATT

GTGTCGTACCCTAATTTTTCCGTCCGGATCGCCAAGCCGTCCGGTCTGACGTTACACTTC

AACTGCGCCTGCAACACCGGCATggACGaTGATCcGGACgCCgAcGaGGAAgatcAGACG

TTcGACTTGTTtAGgTtCGACAACGTCTGCGTGTaCAaGAACAAGCTGGACAAGGCTGAC

GTTTATGAAGCCGAAACCGACAATATGGACGGCAATCTCTACTCGATGCTCATCACGACT

CTTacagagagaggcatcaacggccagttcgtgaacgatctcctcgatctgagcacggct

gtcgaacatCGCCATTACGTCAATTTCCtccAAtCTCTgcAGgagTtCGTttcGgAGAAG

taGTtgTCTtCTGAacgTccCGtATagAGGTCCGTGctAGCTCTGAGAtCGTATTTAtAT

GgAaTTgCGATTGCACGATTGAGCGATGTCGTAAATGAGCTTCCTGTGTAATGAAAAGCG

AATTTATTGTAGAGACTGTTCCTTGATGATCTGGTTGGCGTGGATCCTTCTGCGTTCGTC

ATCGCGGTTTCGTcgcggtgttgccatttcgtcgcgttgttgccgtttcgtaatattgtg

tcgtgctactaa

>contig00077 length=145 numreads=2

cttaagaactgcaggttagaaggtgcaaattctgaagtcactggtcAGTCTGAATTATGT

CTGCCTTGTTGAAGTACACTGTGACCACAGAAGCTGCTCGCATTGTTGATGACACAAGTG

CTGCGACTAATCAGCTTGTTCATcc

>contig00078 length=870 numreads=37

AAGCAGTGGTATCAACGCAGAGTACGCGGGGACCTGGAAGATTCGTCGTACGAGGAACAT

CGCTTGCGAtACAACGTTCCAAAAAaTGGCCAAAAaCGAGAAGAGTGTTCGCGAAAAGCT

TAGCGAATTCTTGCACGAGAAGAACTATTTtACTGATGGATTGGCGTTTGTTGAGGGAAA

GACTGGGGTTAACAGAATTTACTTGTTCGCAGGTTGTGCTGCACTACTAGCACTTTACTT

GATTGTTGGCTATGCATCCGGCTTTATTGTTTCCCTTCTCGGATTCTTgTACCCTGCTTA

TGCTtCCGTTAAaGCGATCGAGAGTGACCGGAAGGATGACGATACCCAATGGCTGACGTA

TTGGGTTGTCTATGCCGCATTCGGAATTGCGGAGTTCTTCTCTGACATCTTTCTCTCCTG

GTTCCCTCTCTATTTTCTTTTCAAGTGTGCTTTCCTGGCATGGTGTATGGCGCCGTTCTC

GTGGAATGGCTCTGAATTCATCTATCACCGTTTCATCTCGCCcTTCGTCAAGAAGCACGA

GAAAGAGCTAGACGAGTACATTCATAAAGTGGGTACcGTTGTAGAAGACGTGAAAAaTAA

agCCGAGAAGCAAGTGAAGCAaGTGGCTTTGGATTCATTCCAGAAGAAAATCAACGACgA

aGgCGAGAAGAAGTCGGAATGAaTGCACTGATAGCCTACtCTAGCAAgATCTGGcctAGA

GAAGATaGAATGCCTGCCAAGTAGCAGCTGGcTTTTCcGGcccaaaaaCGATTTCTTCGA

ACGTTTTtATAACTTTGGAGGTGTCCTTGGTGATTGAAGatgcgcacatttattttttgt

ctttttgcggtagtagaaaaaaaaaaaaag

>contig00079 length=144 numreads=4

CtGGAGAAAGATTATGAaGAAGTTGGGATGGATAGTGCTGACAATGCTGCAGGAGATGAA

GATTGAGAGACTGaTTGCtaTGCCTCGAAATTCTCCTTAcGTTGTATGATGCTGATATTG

CAAGgAAaTAATCGGTGCaaactt

>contig00080 length=1830 numreads=66

AGATTTAAGGCAGATtGTGTGGTTCATCATGGCTTtAATCTGTGCAATTAGTGGGGAAAT

TCCAGAGCATCCAGTTAtCTCTCCTGTTtCCAAGCATATATTTGAGAGGCGTCTTGTtGA

GAAGTTTATCTTGGAAAATGGAaCAGATCCCATTACTGGGgACCCACtATCTGAGGCTGA

TTTgattgaactaaaaactggaCCTGTAGCAAAGCCCCGTCCACCATCTGCTACTAGCAT

ACCTGCGATTCTAAAGGCTTTGCAAGACGAATGGGATGCTGTAATGCTGCACAGTTTTAC

CTTAAGACAGCAATTGCAaGCAACTAGACAAGAGCTGTCTCATGCTTTGTACCAGCATGA

TGCTGCATGTCGTGTTATtGCTCGTCTGACCAAAGaGGctACTGCTGCTCGTGAAGCTTT

GgCTACATtGAaGCCccAGGCTGGAAtGGTTACCACTGCAGCTCAGGTGGCTGCACAGGT

TTCAGCATCTGCAACTGCATCAATGCGTTCAGAAGATATGGAGGTTGCTCAGGATGCGAA

AGAAATCGGAATGTCTGAAGAGATTTtGCAAAAGCTGCAAGACAAGGCTACCGAGCTCAC

CACGGAGAGGCGAAAGAGAGGAAAGAAAGCGCCGGAAGAGTTGACTTCTGCCGACGATCT

CAAGCAGTTTCGCCAGATCGCCACCCACCCGGgCTTGCACAGTGCGAGCGTCCCAGGCAT

TCTGGCCCTGGACTTGCAAGAGGAAGACACATCTAAGGCCATCACAGGTGGTGCTGACAA

AAATGCTGTTGTCTTCAACAAGGAGAATGAACAGGTCATAGCTACCCTTAAAGGTcATAC

GAAGAAAGTCACCTGCGTCATATACCATCCGCGCGAGGAGATCGGCATCACCGCTTCTGC

TGACTCGACTGTCCGTGTCTGGAACATTCCGAACAGCTCTTGCGCGTACATCATTAAGGC

TCATGATGGTGGTGTGACAGGTTTAAGCTTGCACGCGACGGGTGACTACGTCCTGACGTC

TTCTTCTGATCAACACTGGgCTTTCTCCGATATCCAGACTGGAAAGATTTTGTGCAaGAA

CATCTCTGATCCGCAGGCCAGCCACGGTCTGACGTGCGCCCAATTTCATCCGGACGGTCT

CATCTTCGGCACCGGTACTACTGACAGTGTGATCAAAATTTGGGACTTGAAAGAGCGCGC

CAATGTAGCGAACTTCCCGGGACATTCTGGGCCGATCACTGCCATCTCTTtCTCGGAGAA

CGGTTATTACTTAGCGACATCTGCTGACGATTCCGTAGTCAAGCTGTGGgATTTGCGGAA

GCTGAAGAaCTTCAAGACAaTTGCATTGGaCGAAAGAGACGAGATCAAGTCGCTTTGCTT

TGATAAGAGTGGAAATTACTTAgCTGTTGCTGGCTCAAAtATTCAATTGTATGTGGTGAA

GCAGTGGAGCTTACTGAaGaCTTTAACAGATCATtCcGGATtGgTCACAaGCGTCAAaTt

CGgCAAGAAcGCCAGTTTCCTTGCGTCCGCAAGTATGGATCGTACCCTAAAGTATTTCGC

GCGATAAAACgGTCAGTCGcTCGcTTGCAGCTGCCCGAAGCATGCTCGCGGCTATGCTAa

GCTAATTGGAGtGgtATTTATtGCAAACCTACAGTGGACTATGCTTCTTGGAGAGGGAGA

AAACGTTGCGACGTAGCATTTATGCCAACTGCCAATGTTTGTAGCCGTCTGCTGTTGTGC

GACGTTTCCGCGGTTTGCGGTGACCTAGACAACAAGAGAAaCAAAAAAAAAAAAAAAAAA

AAAAGTAaCTCTGCGTTGATACCACTGCTT

>contig00081 length=543 numreads=14

CGAAGgATTGCAaTAgAAGTGTGTTCATGGATCAAtATTGTTAGATtCTCTATAGATTcT

CCCcTCGgCaTTAAAACcAAGCCGTTaTTATGTGTTTaGGTACCTTATCTTaGATGGTAT

GAAAATTTGCTTTTTTTtGCCATGTGaGTAAAAACAAATGTGTACAAGTGAGCTACATCC

TTGGTTGGCCAtAGCTTCTTAATTTGGTTGGgCAGTCGGGCTTTTAGGCATCTAGTACAG

CCTCAAGCCAGTCGACCTTtGATTATGCTTATAAAGTTACTATGGGACACaCTTGTGTAT

AATAATATCTAATGCATTGCCTTATGTTTCTTGCAAAACAAGTGAAAGGTTGATAGTGTT

ATCAAATCaGTATGCAAgAAATCaTTGCTTGAAGaGGGGagCcTCCGTCTCCTGTTtAGT

TATATcAATGTtGTAAGATGCCAGCATGTCAGTTTtGtACCcAACCccAacTTTTaTTgT

ACTGCaGGAgATgAGCAAGCcTGGCTGTAATACGAGCTTccAaCCTGAACcACCTTCAGA

TGA

>contig00082 length=193 numreads=1

ggatttgtgattgagcgaagtactccttgatcgttaagagagaaatgcagctggcaaaga

atacaaatcaaagatgtttaggaatgactatcagggtggtcaatactttgatgtcttcct

gactcaaggaccaaaccctctatcaaagtttaaagtaacaaaagctgctgactcaactgt

agaaaatgagttt

>contig00083 length=177 numreads=1

gtatcttctcaatgagaggcagcagaatagaattatcaagaagaagatgggttgcaagta

aaaccgtgtagactggttctcaacttttaccctaacttcaaaatgactcttatggctatt

ttttggtgctacttttgggtggccttttggtcctgcactggctatgtttgtggttac

>contig00084 length=161 numreads=1

aagctccagtgaagtcaagcaaaagaaaggagctaacccctctcatgtagttcattcatc

ctagcaggatgatgcctagctcttcaaagtgggatccatttgatttttctgaacttggag

aatcatatgaggttgttacttcaccttgctagttttcccaa

>contig00085 length=126 numreads=1

gtttttttaggaatttatttccttccatgccagttaatttttacttaggcacttgtgtta

tgccaggaagttggtagcagcctatgctaagtctgtacttcttcggcttgtctctaatct

ataaac

>contig00086 length=1010 numreads=27

aagcagagtaaaacttcagcatcatacttcaccgaaattcgagtgtcccgtttgcatggc

atgactctgtgcctgagcctggccggtcagcggcgcactgcAGACAAGACACCGCAACGA

GAAGCCGCTAAGGTCGGTGAACTGgCGCTTCTtCTTCGCATCATCAGCGATAGAAAGAGC

CTCAGCGATAaGCGTATCATCCGAAGTAGGAAaTACGGCTTGCAAGAAAACACCAGGGCG

GAcAGGATCCTCTCGGAAGAGCGGGTCATAATGGATCCCATCGTAAAGCAAGAAGACACG

GTTGGGATAGCGCTCCAGTTCACCTACcGATCTAATCTCCCACTCATTATGTCTGCAACA

TCAACCTCTACTTGATAAAAGTTAGATAATGCACCAATCTCAATGGCACCTCCCCAATGT

TGAGGATTGAgCAGCCATTGGCAATACGCTTCGTTCTTGGTGTTCAGGACGACATCGTTG

TAGGTTCCAGGATCCGCCGCAATACAATTTGCAGCACACTGACGAAgCTCACCGgCAAAC

GAaGTTtCTCCATTCAAAAGAACGAAGCTTAGACTGGTAAATAGGCACGAATTGTCTGCG

GgTACTACCTTTCGAACTAGCtGCGACCTGCGAGTGCTAGGGACAAaTGGCTTCAaCCcT

AGATTCTTCAATAATCAGCGAATCACCATGTTTAAtGCCAACACTCGCAAGCGAGGCCCT

AATGTCACAAatCATTAtCTCAGTGGGTGGAAaGCCATGTAAAACTCTTTGGCGAGCAGG

AGCAaTATtTGTTAgAACAAAAATCTCTTCCTGCAACTCCTGCACTGTAGATGCGATCGA

TAGGCTACTTtATTtGTTTTtCTTTTGTCCATtCTTTATattgacaacgcaaaatcaact

ttttatctgccatagatcacttgattcaattttggctcagaaaggcacaagataatgtat

catatttcattaagtggaatacaagctgaacattttataccacgccactc

>contig00087 length=705 numreads=12

TTAGACAAGATCTCAGCCAAGGCTCAATCTCGCGAACTTCAATCAACAAGGACGGGATAT

CATGCGTGTTGAGAATTCTCGTAGATACGCAAACTGGCAGTACTTCTATTGCTTCCGAAA

TATACCTCAGCATTGACAATGATTTCATAGATACAGTGAACATGATGTCTTCATCTtgCt

GCTTCAACTCCTGTCCAGTTCCACTGCTTTCTACGTATGTATTGTCATtCTTAAAaGTGA

CAATAAGTCGGTgaaGTTTTCtgTAGCAGAAGTCCACCAAGTCGAGTaTGCaGTCACCAG

CAGCTTCGCagaCTTCCTGGTGAAACAaAGTTGTTTCCAATAGTCCAGTtATAGTAGCTT

CATGAAaCAAAACCATATAAATAGGTAGAGTTGACGGCAGAAGGCTTTCGTTCTTAAGCA

GCTGCGGAAACACGTACTCCTTCCAAACCTCCGTTGTGAtcaaatcgcagatgaggatAG

GCACCTTTTCATGAGATACCAAGAAATCTTTAATGAATTCATCTGTGTTTGTTGACACAT

TAATGACAgcctgcatgttcaacttttcagcATACTCATGAATATtCCACCACCTAGGAC

TCGCTATCTCAGAGAGGGGCACAACTTCTAATGCCTGAACAAGCAACTCAGCTTCATTAG

CAGCCAAAATATTAATTTCTTGGTCACccATTAtAAGTGCTGCAT

>contig00088 length=463 numreads=10

CCTTCGaCTTTCTCCTTgATAGgTCcAATGGCCTtGCCCGaCGACtGGGCGGATCAGGTC

AAGGAAAGTGTtGAGAAATCTCAGGCTGTTGTCAACAAGTTCGTTgAGCGTGCAAAaGCG

CTCGGgATTGAGACTCAGACGTTTCTAAAAACCTCGGAGAACGgCGCTGGGCAGGGCATC

TGCGCGCTAGCGgAAGAGTtGAACCCGTCTGTGGTCATCATGGGATCCAGAGGGTTGAAC

ACTGTCCGACGCACCcTTTTGGGCAGTGTCAGTTCGTACGTAGTGAACCACTGCAGAACG

GCAGTCACAGTtACCCCTCCAGTGCACcACCcGTTGGGTAGCcGGGgTGGAGCCGATGGT

GTTCCACACCACTGGTCTGCCTGATTGATTCTGCAAAATGCCTTCcAGTAaCCcTTGTtG

ATGGAATGGGGAAAAAGCTGGACGGTGTCtgCGGAAGACGTCT

>contig00089 length=161 numreads=8

GCAATTGGAGGCGGTATGAATGGACTTCTGAAGACTAACCTTCTGTGAGGCATATGTTAA

GCCTAGCTAGTTGTTGAAGATGGGAAATGGTGAATCAACAGCACGACGCATATCAATGCA

AAGGTCAGATGAAGGTGTTGTGAAGaTATcAGAAGATTtaa

>contig00090 length=170 numreads=2

AAaTGGACTTTAGGttGGCAGCTcAACCCATTATAGAAGCTGTgggtGATCTTAGTAGCT

TGTATGTAGCTCTTGCTATTGTGGTGTTCACTGCAGTGTTCCTTTACTtCcAttGTAAGA

AGTACCAGGCCTACCAGGGATACAGTGCTTCcTtCTTGGGCTATGTGact

>contig00091 length=1152 numreads=36

AAGCAGTGGTATCAACGCAGAGACTTTTTTTTTTTTTtTTTTTTTTCGGACACTACTATG

TATATACATATATATGAGAAAaGGAtcacagcaaagacttaagctaggtgaaggcagatt

gtcgatcaacacagaaataaaaggcggatctttctgttgatcaggtACGACgtATGGAGC

CAGAGAGTCGGCCTCaTGGCtGGAAAAaTTCGCTggAGGgCcAaCcAgAGCGCTGACGTc

AAtGTCtGCAACGTCTAACCTCCATTTAtCTTtGCAGCGAACGaTtCTTTGgTtCTtAGA

TCTCACTTCAGCcGAgTCTGATAAcTTtGCATCTAAAaCACGCAATAgAGCTTGATCTTT

CTTtAACTCACGGACAAGCACATACTCCGAaGCTGTTTgAaTGGATGCCGGCTCCGAAaG

ACTCAAAGTATCAAGCATGCTCTttATTTTtttATAAaGCTTtAATGAAaGCCACGCATC

CGTGGCTGCGTACTTAaGCTGGCTTTCGTGgaGAGTGGAGgCTTCccagTTCGACTTTtG

GCGTtGcttCGAGAcTTTCCAACCAAACAGCTGCCCAGACAAATTTACTAAGCCCGTTCT

gCTGAGTCCCAACTTCgCAGCAAGTTCCTGAACGTCCCCAACATTAGCCGAGTTAATGTT

ATATTGTAAGTACAACTCATGGACATCTTGTGgTACTCCACATCCAACCTTTAAAaCTGA

TCTTTCACACAAAAGGTGCCTTAAACCTTGTGGAAAATCAGAATAAAGAAATTGCCTGTT

TCTTCTGAGCCTCCATATAACACATGCATCTTCACAAGCAAGTTGAATCAGTGAAATTGA

GGGAACATGGCTAGAAGAACCCCAAAATACAGCAGTTTCAGTATCGAATCCAAGAACATC

TGAGTCCTTCAGCCTGGAAATTatATCAGCATtCCTACTACTTCGCTCCTCATCTTGACT

ATTGATTACAACAATGCAaCCCTCAAAGGaTTTTACAGGATATCGGGgAATATGATCAAG

GTGGAAGTCAAATTGAAGAGCAGACCGTCTTAGATGCTCAATATACTGGACATGCATATA

AATGTTCTTGAGATGTCTGAACCGTTGCGCCTTGGCCAATGCATGGCATCGTATCATAAT

AGCATTACCTTA

>contig00092 length=174 numreads=2

ctgaagttgtcaatgaaggcgtaGGgCTTTGTTATCACAGAGACGAGCAAGACTATCATG

TAtaTCATCTaTtCGTTGGTGTTTTTCTTtCGGTGTGCTGGGTAATGGCActGTTGTGTA

CGTCGTTGGTCTAAAGTCAAAGGATAAGACTACATCTAAaTACgctgatgatat

>contig00093 length=1021 numreads=85

cGAAATTCCCCAATCATTGttttCATGAACTATCTTTTCTTCTTCTGCCTTTGGGACATC

AacaTCaGttctGCTCTCCAATTTtGCTTGATATACAaCTTGGCGCTTTTGGCAATGCCT

ATCATCACATGTGGGGTTAGGCTTCATTGACATAGTTGGAAAGAAATCTTGCAATGCATT

GTATCCTAGATAGTGCGAAACTGAGCCAAACTGTAACAAATATTTTAATGCATTTTGGAC

AAGTAAGCCAGCAATTATGGCCATGGTGGTAGGCAATGAAGCTGCACATACTCCATCTCG

CTTCAGGGTCTTTtCATCGATTTTTGAAGCAACTACCAGTGGAGGTGCACaGgCAAAaCA

GGCAGTTTCACCAGGCAAAATCAACTGAATGTGCCCTGaGaCAGCATTTtCACTAACTCC

AGATTCAAACCAGGCCTGACCAAGCTCATTACATGCAGTGTTTATGGTCATTCTAGCTTC

AAAGTTATCAACACAGCTAAGAACCAAGTCAACAGGACCACCTATcAAACTTCCATATTG

TATACGCTGCATGAAAAGACTGAAATTCTCCATTGTTGTTATGTTGTAATTACAAGCTTC

AAACTGCACATCAGGGTTTATATTCTCCAAGGTTCCTTTAGCTGCTTCAACCTTGCTCAA

CCCAGACTGATCCGgaCGAAAAAaCaGCCTATTCATGTTAGCCAATTCtACTTTATCATA

ATCAAAGAGAATTAGCTTtCCAATaCCACAACGAGTAAGCATCTCAGCAGCAACACTTCC

AaCTCCTCcAACACCGACAAcgCTACTgTAAaGTTtCGAATGCTTTCAtagTTTcgCAAC

AaTTCCCATTCGTTTtAGGgCCATcaGACGGCTGTAGGGGTTGCTGTCAATAACTTCTGA

GCTCATATGTTCAATCTTTGTTCTCACAGCATAAGTTGATTTACTCTCCATTTGAAGCAA

CTGCTCCagCTCCGTAATTCGCtGCTTCAGAGAGTTAACGTCATCCATGTTTAATGCTTG

C

>contig00094 length=193 numreads=9

CTTTCAAAGGTTGGCAAACCTGGTGGCTCTTAGCGACCTGCACCTATGGCTCTTTGGATC

GATAAGCACCGTCCAACATCCTTGGCAAAaCTAaGTTATCACCTGGAGCAGGCTGAAAAT

CTTAAAAAaCTaGTTTCTGGTGGTGACTTTCCACATTTGCTtATATATGGACCTTcTGGA

gCAGGCAAGAAAA

>contig00095 length=164 numreads=1

gtgagtcagtgtgaatggcatatgatcctcgccagtggtatagttatagtaagtcctaca

cagagccagtgaccatacctctgcttggcgatgtatatgaccaaaagagtgaaggtgttg

ctgaaagtggcacacaccagccgaaacacttgcaagaacagcaa

>contig00096 length=153 numreads=1

ttagaattgtctttatgccagtatgattttcatgttgaaatgtaatcatttgtatgcagg

taattgggaaatatgtgttggtagaggctattttagctgctattagtcgtttttccgatt

ttcgctataccttttccttatgtcaacatggca

>contig00097 length=131 numreads=1

tgactggattgcggatgtaaattatcccgtctaagaaaatagacagaccttgttgactat

gggtggaaggggcgtcatgcacatcctgaggcagctcacttgaagggctggcaaaagtat

ttcaatgccta

>contig00098 length=118 numreads=1

ggcgaatgcatgaggttgactagtctcgcaggaactggtctaaatattagttgcgtgtta

ggatatgtgtatctagcaatgctgtcatgggccagctttgtctatggaacctgatgtt

>contig00099 length=171 numreads=1

aaaatctattcattgatagattaccatgtgacctgtcaatggatagaggctaggcattgg

tttgtgaatattatgcttgatgatagttggattttggagcaacattgagttctttgaaaa

tacaggtttgggcagactgctggtcactattatggcatcaagggttattag

>contig00100 length=322 numreads=4

aGCTTGGATGGTCTCGATGAACGACTTTTTtAATCAAATTCCAGGCTTGACCTGCTCTAT

TGACCTCAATCAGCTTAAACCCGTCAGAAACATCAACGTCGGGCCAGTGTTTTGGTAGAT

CGAAACCGGTCTTTTTGCCTCTTCTGAAGTCATCAATCGACTTGAAGACCGGGCGGCgCC

TAaCGCTCCTTGCAGTTCTGTAtCTTtCATCTTTATtAACttGGATcATACGGGAGGTgT

CTATCGtGtACCTGAaTTCACTtGTCgAAAAATTAAAATGCCTTTTCCCCTCAGAGTAGC

GCTTTTCAATGAGATCGCTCGC

>contig00101 length=154 numreads=1

tcttaaaggttgctttccggttccagcctacaagaaaacatgtgattgtagttgctcgca

aggtcactttgagataatcaaaccttgatggtgattactacaattgatctcttgcctctg

gaagatatagttttgaatatgttataaatgaaag

>contig00102 length=303 numreads=29

TCATGCTATCAAGATTCTTGGGTGGGGAGTAGAGAATGGAACTCCTTATTGGTTAGTCGC

CAATTCGTGGAATGAAGATTGGGGTGATCAGGGATTCTTCAAGATCTTGCGCGGCAACGA

CGAGTGTGGTATTGAGGATAGCATTGTTGCCGGCGTTCCTCGGTAATTCGCGAGAATTCG

AACTCGAAATTCCGATGCTGGTGCTTGCTAGAGGACATATGATTCGTTTTCATTTTTAGC

TTGCGTTCCAATAGATCTAAAAGTTTGGTTGTTGATTCAACGAGGAGCTACTAGCTTTTT

TGg

>contig00103 length=193 numreads=3

TGGGCAAGCTGATTGGCCAAGGAAGACTTTTTgCAAAAGTAACCTCAAGGCCAGGTCAAG

ATGGCACtGTAAaTGGGTaCATTGTTgAAGgAGAAGaGCTAGTTAGTCTTCTGGAGAAaC

TGAAGCTGTATgAAAaGAAaTCTGTAGCATAAtGTtCAGTTGcTgCATaCTtGAAAGAGg

AGTTCTGTGcaaa

>contig00104 length=156 numreads=1

aagcagtggtatcaacgcagagttactttttttttttttttttttttttttccgttcaaa

aagaaactctcatgaactttaaatatcaaagatacatcatgcatcggtaaatagtcattc

acagcatctgtcaacattaactgaagagtaggtaag

>contig00105 length=206 numreads=4

ttCCGGCTCCAGCGTCTTCTTCAACCCAAGCGGCAAgTtCCcGaGAAATCGCGGCGATAG

AGCGATctCCCTTtCGTCGACAAGTtCTTCCACACAGCAGTGCTTTTgCCCGATCAATCc

cGACACTGtcTTATACGCTACGCGGCGGTTTACACGACGgTtgCTTTGACTGCCTTCCaC

CGgCATTATaGGCTATCttgatctct

>contig00106 length=700 numreads=22

GGAACTGGTTGGCCTACTGATCCCATGTGAGCAACAGGCTGGaTTGGAGcACCTCCAGAG

AATGaGcGACCTtCTTGCATGCTGCGAACAAACTGCTGCTGCAGCATCAAGTTCTGCATC

ACATCTGACTGCATGCTATGTGCACTAAtATGtGACATTCCGGATATATTgATTGGATCA

GAAATCAAAGATCCTTGCTGGTTCACCATAACAGAACCAGCTCCAAGACCCGAAGACACC

ACATTGCCGCTTGATGTCGATAAACTCATTTCGGATGATAACCCATTCAAAGCCCCATTa

GATGAAaTACTtGGCCCAGCGCTCATGCTCAACCcGTTTACGCTTCCACCATTTGATGTG

ACCGAGCCGGTAaGACATATTTCCAAATGCCTGATGCGcctGCTGCaCCtgAGGAGGCTG

ATgATGAgAGgTTaGGTTCTGCGGCTGCTGCTGCTGCTGCTGCTGCTGTTGTAAGtttAA

aGTTGAGTCTGCAAGAGTAGCAAgaaatatcagatttctatgcagaagctgcTGGTATTG

CACGCACTcgCGAGCCTTTCCTTGATTTTGGAAGTCGATGAAAGTTTGAATAAGTGCAGC

ATTCTCATctAGCATCTTCTGAACGGAAGCCTGGCTGATGGGCtctccttcttgtgcctt

gcgAAGTAGGTGgTGCAAACGCAGACGACATCCTCTAaTT

>contig00107 length=1348 numreads=31

cTCCCTCGCAGCGTTCAGATTCGTTGGAAATGTTTCGCGGACGATCTTTAAACtCTTTTa

CTAGgCgCCACACCTTcGCTCGAACAATCTCTTtGGCTTCTCCGTTAAGACGAGAGCTCT

CTACAGAATGTACGAGCAAGTCACTCAAGCAGTCGGTGCAAagACGATtGCGAAAGCGAA

aCATTTCCCcGTCAATCTTAACGAGAaGGTCTCCAGCATCCGGCATTGcTTCGTtACCAa

TTCTTTTAATGTTCTCTACgTTAATGACCTTGGCGCcgTAAGGAaCTATGTCTTtGTTgA

AATACCTCCGTCTTAaCAAGAAAaCCAGAAGAACGATCAAGAATAAAAGACATACGGCGC

CAGCTATGCCAACAACTGCGCCATtGATATTTGCGTCAGTTGCGTCACTTTGACAGGACG

GAACGgCGCAGAACTCCCACTCAGCAGCAGTGTCATTtGTAAAGCACCAAGGCCTTGTTC

GGTATCCATTAGGATTTCtGCAAAAATTaGCATCgCcAAATAAGTTtGTTgTGTtCAAAA

GAGATGgTCTAGTACACGTGGTTGCCCAAGGCtGGCAAGCGCGACCCGATATGGTGgtGT

TGTGGgTGCcATGGTAGCTTTCACCGACATCACTCAGGCACTGTTCGcTtGTGACTTCTG

aaCGGTAAGGaTCTGACAAATAGAAGCATTCGGGAACAGGCCCcGATGCCTTGgCAGGGA

AAAGtGGcGATTTCAAGCAagTTtCTTTGTTCATAATATTCATGAGCAATTTCTTTGTCT

TACGGgCAATtAAaGCGGTaTcTTGATCAATAaTtGTCACcAATTCTGAAATACTGCTAC

CACaaCcTCCAaTaTGAAGTCTGAGACAAGTTTCCtGGcATAAACTTTGATAAACCACAT

TTTCCTCTGCGTGCGCTTTGTAGCAAACTGGAAATATTatgtgacacaaaatatccataa

gcataaactcacatttgagtccatggctaggagttaaaataaaattagcatgtcccTGCA

TTTTTCGAAGATCTTCCTCAAACATGttgTTTTGTCCAGTAGGATAACTCTTAACAAAGG

AACTGCCAAGATGTGATGAgCAAaCaCcTTCACCATATTtCAAGCAAGCACCACCAATGT

GCAAGTCTATGGGCGTGCAACcAAaaCTcTtaGCCGCATAGGCACTGCTATAAATGAGAA

ccTTAACAATCCATTCCATGGTAGAACAGAGCATGTACAGgTATCAGCTTCAGAGGCCGA

aGGAGAAAATTACtGCTaGAaGCAATAGCCAaaCATgCGgTGCAAAGAAAGCAAAACTTT

ACTAATGcTATATtCttagtctctcaaa

>contig00108 length=589 numreads=15

GgtAGGATAGATTTTCGTACTGACCGTGGTGGAaTCAcACATAATGGAAaGTAAATCTAC

AGAACCTAtAAAAGTTgAGAAATGGGATGGAAATGCTGTAAAAAaTGCCTTGGATGATGC

TGTGGGCAAGATCTTTAAAGAAGACTTAGGgTTtAaGGAGAAAACtCtttAtGTGGACaT

tCGCCTGTTCCTCAGCACTCTTGGGTGTGTGgTTGCTCTGgCTGCACtGTTATATGACTA

CcTATACCCGTTtCCAGTCTCCAAATATGTTTTATtGGCATGCTCTGGAAGTTACTTtGT

TTTGATGTCTATACTAACTTTGTTCATGACATTCGTtGAGAGGAACATAATTTtGCAGGC

TTTGCAAAAGGACAAGGCTGGTTTGGATCCGGATTCACAGTGGACAGTAGGGACGTCGTT

ACGGAAGTTTGACGATAAGTATACCGTGCAGTTGgCAGTCATAAATGGAGTGACGAAGAA

AAAaCGCcAGGCTTCCTTtACTAAATCGgTGGCTACaTGGTTTACTGCTGATGGAGAGCT

GCTTTTCGACAAGTTtAAGAatGAcGTTATGTCTTGTCAGCGAAGCCtt

>contig00109 length=214 numreads=2

CATAAGAGAGGACCATGGAGTTGTACTAGACCAAGGCTTTTGTGGTAAACGTGATGAGAG

CAGAAATTTGAAACTTGTATGAGCAGATTGCAGTTGTAAAATTGAGGTTTACTTGGAGGT

GATGTTGCTTTCGTCACATTCGAAATtAACTTTGTAGAGCATAGCCttcATTGTATGTag

gtatttttattttttttaccgttatctcttgatt

>contig00110 length=183 numreads=2

ccATTACtAaTTGGTGTCCAGAGATTAGTTGATGATGTGTTAAATTTAAAGGCGCAGTCA

TTTTGCGAGCGAATAGACACCCAGGTGGATAAGTTCTTCATGGCCCGTCAGCATTTTGAG

ATTGGTGCCAGTTCGGGGGCTGAAGTGTCCGCAACAGTAGTTGAAACATCCTGTTCtttt

Caa

>contig00111 length=164 numreads=1

caacaaggggttacttatatcatgcagtacacgtagcatcgtttttcagcttttgatagt

aagcataggaagaaatgacttggagatattgatgagatgaaggtttacatacagcgtgac

tatacagaaggcacccagtgccaattccaagagaaattcccttt

>contig00112 length=225 numreads=45

TGCCCAAGCTGGCGCTAGACTGCTAGAACAGTATATATTACATGGGAGAGGAGACGACAC

GATCGCTAATTGGATCGCATCTCTTGATGCCGTGACTAGAGCGGAGATGCAGGCTGTCGT

TGCATCCAAGGATATCGCCCCGTTGGATGCGAGTAGAAAGTTTGGAGCTGCCTGCTATGT

TCCTGGAAACTTTCAAGTTGCCGTCCATTGTCTTCTAAACAACCC

>contig00113 length=225 numreads=13

GGCACAAGCTGGCGCTAGACTACTAGAACAGTATATATTACATGGCAGAGGAGACGACAC

GATCGCTAACTGGATCGCATCTCTTGATGCCGTTACTAGAGCGGAGATTGAGGCTGTGGT

TGCATCCAAGGATATCGCCCcGTTGGATGCTAGTAGAAAGTTtGGGGCTGCCTGCTATGT

TCCTGGAAACTTTCAAGTTGCCGTCCATtGTCTTCTAAACAACAC

>contig00114 length=1167 numreads=43

AAGCAGTGGTATCAACgCAGAGtCGCGGGATCGAAGAACTTTGCCACTtAaGTATCcTCT

TCCGGAACTtGTCGTTCTGCATAATGACAGCAATTTCATTCGCGATGTTAAGCTtCTGGA

AAaGTATATTTTGGAGGAGCTTAATGTGAAGTCAATCACCTGTTCTTCTGACAAGCAGAA

ATATGgCCTGCGACTCAAGGCTGATCTTGATTTtCAGCAGCTAGGAAGAAGGTTAAAaCA

AGACTTGAAAAAGgTtGTGGATGCATCCAAAGACCTTTCCGAAGcAaGTCTTCGGGCTTT

TCAAGAAACGGGTTTGCTCCATATTGCGGgCCACGAACTTTCGTCTGATGAAGTGCAGAT

TAACTATGCTTTTGGtAACACTGGCCAATCTTCGAAGTATGAAGCGCATTctAACAATGA

AGTAGTTATTCTCTTGGATGTCACACCCGACCAGTCCATGATGGACGAAGGCGTTGCCAG

GGAGATTATTAATCGCATTCAAAAGCTTCGCAAAAAGGCTAAACTTGCTCCTACAGAAGA

TATTACGGTCGTGTACGAAGTAGTGTCCGgCgAAAGCAGTGCTGCTCGTGATCAACTACT

AGCTGTAGCCAGCTCATTTTCAGATTATATTCAGGAATGCACGAAGCAGCCTGTTTTtGT

TtGTAGCACTGTCCTACCATTGGATGTATTGATCTCGGAAACTGTGCAGTGTAAGGGTGC

AGATCTAAAACTGACGATCTACAGGGGTCGCGTTGATGAATCGAGGACTAGGGTGGCCGA

TCTTCCATTTACAAGGTTTATTAACTtAGAaTATCAaGATGACGGGGGCTTCGTTAAGCG

AGCTACCGTATTGCTGGaGAATCCCGTTGGAGAGCTCGTTTTGACCGCACCGCAGCTTTA

CCGAGAGGCTTCAGTTCTATTCGGATTGCACGGAAAGAAATTCTTAGTGTCTCCGTCTGC

TTCGCTTAATCAAGATTTAAGTCCGTCGTTGGCTCAGTACCACGGCAAAaCGCTCTACGT

CGCTGTTAAAGGCGgTTCTTTGAAGCTGTAATAGACTCGTTAGgAATGCCGgCGTCgCAT

TCCcTCATGCAAGCGCGTTTCAGGAaTTGCGATCgCCTTcGCTtGTGTCGcatgcggatc

tacttttcgcgtaactgttggcagcga

>contig00115 length=202 numreads=1

caattttctggggtagctcacacattactgaaagcagtaactgcaatacatctgtgaaat

tgtcttcctcttcctcaaccgctaagtacactagaatgctcttaagttcgtcctcctgaa

tcccgttgtccttgataatcatctgcttgatcagaaggagtacaaatgctcttaatgaaa

tcacctcttcaacggtaacaac

>contig00116 length=147 numreads=6

AAGCAGTGGTATCAACGCAGAgTACGtCGGGGTCCGCGCTGCATACATCGgCCAACCAAT

ACCGAcAaGCTCCcAGaCTCTTTCAAGAATATCCCAGACCTTCACAgAGGGaagCGGgta

CTtCGAAATATAGAGATTGAAACcTCA

>contig00117 length=165 numreads=1

tcatcacaatgcatattgatccatacattcccgttattgttgtcagtgccttttgggctg

ttgttggcatagttgcaccaatctttgttagtggacctaacaggcaagttattcgagtta

gcttgaccctgactgctgtatgctgctggctttttggctatgtac

>contig00118 length=315 numreads=4

aTGACGAAGTAGGGGTGCGGGCTTGCCTCTGTGCTGTTTTGGCATGCGTTGCAGATTTTG

GGTGTGTTGCACCACGTTTCGTCAGCGTTTTTTTtATTGCGCGGACAGTcTCTgCCTCAC

TATATAaCGTGACGTgCTTCACAaCTaGTGTAGTCTTCTAGATACTCAGTCATCGTGGgT

ACcTAGTGTCATCTAGGCCcATTTCAgTTCAGGCGGCaGtAGTCcGTCAtCGTTCACACT

cGTgTCTACGAGTGGTGTCATATACTACAAAAAGTATCCGAGGTGTGGAAGAGGATAGCG

Ctgatcttggcaatt

>contig00119 length=454 numreads=7

GTGAGCGATTCTTGGCCATGGCTATGAGAGCTTTTAGCGTgATTTCATCACTGAGAGGAG

gCcTTTGCAAGGCAGGTaGCATGGTACTCGtCTAGCAATCTGAGGTTaGCcAcTGCTGCT

AGAGATgCACCAGCAGTTGATAcTATTCCCacAGACATGGagcaatctactggattagag

cgaaagaattagaggctatcctttctggtcacacagatccctttaacttaagtttaaacc

ggggcccctcctggaacaaaagataagcctacattagtgctctctatGTTCGAAGAAAGA

ATTATtGgTtGCATATGTGaGGAaGACTCGACAaCcATtAACTGgATGGTTTTGAAGAAA

GGATCAATGCAGCGTTGCACCTGTGGAAATTGCTTTCAGCTGGTAGCTGGATCCAGCAAT

GCACTTAAGGAGGATCACCATTAATGCTCAAggg

>contig00120 length=1188 numreads=33

ATTTTATGAAGACTCAGTTGCAGCTGAaGAAAGCTGTCTCCAAACcTTcAGGAGTTAAGG

GAAGCAGAGCATTGACTACTTCGGgTCCTGGAAATCTAACGTtGGCCAGCTTGACCGGCA

CTACAGATAAGCAGACGATTGTCGTGGACGAGTACAACCCGCTTAAGCCGAACGATTACT

TTGAAGTGAAGGAACGGTTGAAaCAAAAAGACGAGTCCGAGAAACGTGAGAAGGAGCGTG

TAGAGAGGCGGGAGAAAAaGGACGATCGAGACGAGGAGCGGGAAAGAGACAGATCAAGTC

ATCGGCTTCGTGACAGCGATACTGATCGCGAaCGGGATCGcGATAGAGATCGAGACcGAG

ATCGAAAAGATCGGGATCGTGATCGGGGTgATCGTGATCGAGATAAGGATAGAGAGCGAT

CGAGAACCAGCCGCGGTGGAGCCGCTATTGCTCCGCCGgcATCTTTGCTCACcACGCcAG

TAGACCCAAACGCAATTGTGAAgAAaGATGGCGCCGGCACGGGCGGATtCTCTACAGGGA

ACGTaGTGGCTTCGAACATAATGGCTAAATATGGTTGGAAGGAAGGACAAGGTCTGGGTC

GAGAAGAGCAAGGATTAAGTACTTGCCTTCAAGTGGAGAAGACTAGCAAAAGGGgCGgCA

AGATCGTCAATAAGGATGTCGAGAAACTCAAGACCAAGCCAGAGTTTGTTCCGCCTTCTC

CTCCTCAGGCCCAGCCAGTCTCCGATGGAATGGAAGAAGAAAAGAAGCAAAGTGACATCA

CGGAAATGATGAGAAATCCGTCGAAAGTCATCGTCTTGAAGAACATGGTTGGACCGGGCG

AGGTAGACGACGATTTGCAGCCTGAAaTAGAAGAGGAATGCGGCACCAAATaTGGCGAGA

TCACAAAaGgTGATCaTaTTtGAGATTCCTGGGGCGAAGGAGGATGAGGCTGTGCGAATC

TTCGTCGAATTCAAACGCATGGAATCTGCTGTTAAAGCAATCGTTGATCTGAATGGCAGA

TtCTTTGGCGGACGCGTCGTCTCGGCGTCTTTCTACAaCCTaGATCGTTTCcGCAGACTG

GATCTtGCCGgATGATCGATCGGTCCGgACACGATtCTGGGCGAAACaGTTAtGGGTAGC

CATGGAGGCGAaTTtAaGCCAAaTGCAGAcgtattcctgcacaatttt

>contig00121 length=807 numreads=16

ttgtacactttcatacccAaCTGCTGCAACCAGTAATAGTCTCGCGAAGCGGTTGCGTTG

AAGAAATTTTTCgAAGTATCTTGTAAGTCTCATGACAAACTTCCTGGCTTTGCGTCGaGA

AACTTGAACGCTTTGCgTAaCGTTCAGTAATGATCAGTAGTACAGATGTTTATACACAAA

GTTCACTTGCTGAATCATTCTGCATTCTAAaTCAATCACAAACAGACCATGGTCTTGCAA

ATTCGCATAAGAATTCTTGTGgAGCGTtCTtCGTTCCGATCCGATCACCCTCcGGATCAG

GTCGATCAGCcGgCAAGATCTAGTCGCCGGAAACGGTCAAGATtGTAGAAaGAAGCAGAG

ACGACGCGTCCACCAAaGAaTCTGCCGTTCAGATCAACGATTGCTTTGACTGCCGATTCC

AtACGTTTGAATTCGaCGAAAATTCGCACCGCTTCCTCCTCTTTTGCTCCAGGAATCTCA

AATATGATCACTTTGGTTATCTCGCCATATTTCGTGCCACATTCCTCTTCTATTTCTGGC

TGCAAATCGTCGTCTACTTCACCAGGTCCAACCATGTtCTTCAAGACGATGACcTtGgAT

GGATTTCTCATCATTtCaGTAATGTCACTTtGCTTCTTTtCCTCCTCTTCCATTCCTtCG

gCACCAgACGGGGGCTGCGGAGGAgACGGTGGAaCGAACTCTGGCTtCGTCTTCAGTTtC

TCGACATCCTTATTGACGATCTTGCCTCCCCTCTTGCTGGTCTTCTCAACTTGAAGACAG

GTGCTCAACCCTTGCTCCTCtcgaccc

>contig00122 length=160 numreads=1

cggtatagtgagcgagataaaaatcgccatagtgtggattttgtacttgcacatcgttaa

catctgacgcctagatgttctgctagcattaaaatgagtggttgtcttgacaatgctcac

tgtccgcaagtaaagtgtcctgatttgtctgaaagacgaa

>contig00123 length=1037 numreads=22

ACTAGTACATAGTACTTCCAAGATGAGGGTTATAAAGAATGTATGTAACATGTGACAATG

TAACTTCCCAATGTGAAGAACAGATTTAGTCATCTGGCTTAGTAAGTAATATTGAAGTGC

TAACATCATCAATAGTACGAATtGTTTCCAGTGttaccaaccgttaaagctctcatacga

atgtcttcagtAAGCTTTTGCTGCTTCACCAGCTTCATTTTCTCAATCATATTTTtCTcc

acaatagcattgactttggagcGATGttgCTcTAACTTtatACGaTAAAtATTTTTTTCA

TGCTCTGgTGCATtGCGCCACATGTGGGAAaGtAGCTtACTGATCTGTGCAAaTGAcATT

TtCGGgTTACGAGCAACAATCTTGGGTCGGTAACGACgaCAAAAAACAaaTACGCTGTCA

TAGTCTGCTTTTTTTTTtGtCTTGCCGCTCTTTGACTtGTTTGTGCAGAGCAGGTACCCA

ATAGCTTTTTGATATTCATCATTCATCAATTGCTCATGCAGCTTTTCTGCTTCTGGATCA

CCCcGTTGCGAACACAAGGtATTTATTTCCTGCTCCAAGATGTACTCATGAGGaCTACTA

ACGTCACTTtCtCTGTATGAAATAGACACCATATTGCATTCCTTAAGGTTTTTTGAGCTC

CCAGGTCCTGCATCACTCTCTTCTTCACCAACAACATCAaCGATAACCATTTCACATTCT

CCATCCCCTGGATAACGATTTGAGGAGTTGTGAGCaGCAGAACTTACCTCAaCTTCACCA

GCTGTGTCAAGCTCCCCACGACGGGCCCAGTACACATATTCCTGAACCGGTTCCTCGATG

GAaaCAGGGACTCGAACACCTTGGTGACCTTTAACTGtCTCAATGgCAGCAACAGCAGCT

GCCTGCTcGGTCAGGgCATTTAGATTATCCAGCAGCTCCACATTTGATGTcATGTCTTCA

TgCATGGATAAGTCATGAtCCaCAacTTGTCATGCCAACCTTCTGTGcTACCAGtAaGAA

CACACTTTtCTCCTTGA

>contig00124 length=311 numreads=3

ggcgatacgtcatagacgatcccgctgttagctgcccacatagcgtctcgtcgaaggcca

cgccattgatctctgtttcgcgctgcttcgtttcccgATTCGAGGTCAGCCTGCCTTCCT

GTCACGAACAAAGTGCCGGTCAGGTCATGGAGCACTTGGTCGGACCacttcttttttgga

gcattttttggcatcttccaatccggaggtttctcagcaaatagtaggcgttttggaaga

cgttcatcttgcatacgactgacgtGTCcAAACCAGCGGAGGCGGTGAGACCGAATGGaa

aGGCTGATTAG

>contig00125 length=380 numreads=6

aGTTctaGCCATaaTTCAAttGCtGTTgCtGCACCATTTAtCACTCATGgAATCGgaCAG

AGCATCTTtATGaCtGGCTTAACATTAGTTGTTCCAGATTACCAGCACCAAGGCATAAGT

AAATtGGTTGTTTGGCATTGTTTGTTGCATTCCATCctCCATGgCTCCTGTGATATTGTG

ATTAACATTGCAGCAAATCCTAGCTATTGGAGTCagatggaggcaacacagtatgatttc

tatcctcattggaagactccactagctgcacctaagaagtggcaagttgaaatagcttct

tttatgatggagtatcatcggaaccagttttcatgcagttctcttgccaaattggatgca

agtacccttgttattgttgc

>contig00126 length=688 numreads=29

cTGCTAGCcAAGtCTGTGCGTGGTTCTGCAGCAGCACAACTAGTTCACCAaGCTACAGAA

ACTCCAGGCATATTTGTTTTTGGTGAATTGGCAGAAATTCCAaGTATAAAACAaCTACAA

CTtGATTCACCAAaGGATtGgAAGTtGTTACAGTtATTTCCTTtGGgACTTTtCAAGACT

ATAAATCAAACAAAGATTATTATCCGgAGCTATCAGAAAAGCAGATTTTTAAGCTTAGGC

ATCTAACTATTGCTTTCCTTGCATCAAAATCCAAGCATGTCCCTTACAATTTGCTTCAGA

AGGAGCTTGAAATTGATAATTTGCGACAGCTGGAAGACCTCATTATTGAAGCGATATATG

CTGGAATTCTTAAAGGAAAaCTCGACCAAAGCAGGAAGCAGTTGGAAGTGGAGTTTGTTA

TTGGACGGGACCTTCAAGTAGAGACCATAGACTTtATGCTGGATATtctGGAAAGTTGGT

CCTCTAACTGtcGATCCGCACTTGCAACACTTGAGACTCAGAtctGTCGTGCcAATGATG

TGAaGgATCTCAAAGCATCACTCAGGAAGGACACTGAAGCTGAAGTGGAAAaCGTCAAAA

AGGCTCTGAAAGCCCAAaGTCAGGAAaTGaGCCAaGATGaTgTGGAGGCATCATCAaGTA

GCAgTTTGCCGATGActCCAAaCGAAGc

>contig00127 length=194 numreads=1

ttctttgagctgcggtaaatatggggaagccacaagctagaaagagatctcacaaaaaca

taaaagattttaaaagaaagcaccgaactcgcaagcgtaccaaagatcttgaccaaattc

atgacgaccttgtacctaagaaagcagacatcttgttacatcagaagttagatggggatc

ttcctggtggaggt

>contig00128 length=139 numreads=2

AATCAtgaatcagatcatcaaaggattgttgatttgcaagtgcgccgtcacaaaagcatc

ggctccaccacgtattgcgctagctcatagaactgtggcgtccctcagcgctttctcgta

ctcgacctccgacagtgcc

>contig00129 length=237 numreads=1

aagcagtggtatcaacgcagagtacgcggggacgtagagatctcttagtttattatcctt

cgagagatcaagtttgttagggaattattagccagaatatgtcagattctgaagatcgat

tgcttcttcattttatcatccttagcaaggatgctgggggaatcattggaaaggaaggcc

gaaatatcaggcaaatgcgggacgagagctctgcgaatattaacatatctggcagcg

>contig00130 length=808 numreads=13

agagacTTAAGGCTGGTTATAGTTGTGTTTCCCATGGATGAAATCATTGCAAAAGACACT

CCGGAATTCGTGTCCAAGgTGTCAGACCTTAACaGGCAGTTAAATGAAATAACAGCTCAT

GTAAgTGCTTTGTCTGATAAaGTAAGGAaTGGTGAaTTTGgTACAGAAGGAGgTCTAaGC

TTTTTGGAGACAAAGTTTCaTTTgcttctgcagtatgtcatCAATCTCTCATaTGtgAtC

cTTCTcAAGGCTgATGgCAGgAaTaTTGAGGATGCCTCTGtcATtGAGAGACTTGTAGAA

aTacGTaCTGTTCTGGAGAAGGTTAGgCCTATAGATAAAAAaCTCAAATATCAAGTTGAA

AAaCTTTtGAAACTAGCAACATCCGACATGCCTGTTACAGACAAGCACCCCCTGTCCTTC

AAACCAAACATTGACATGCTTACAAATCAAGAGAgTAAGgAGCAGGATGCTGATCAATCT

GATTCTGGGGAAGAGGGTAGCAATCAAGTTTATGTtCCACCAAAGGTtAGTGCTGTACCG

TaTGATGATGAGTCAAAACATGCAGAAAGGCATAGGAAAaaCaTGACATCAcAGAAGCAT

GCACTCAATAGCTCATTGCTCAAGGAGTTGAGAAGTGAGTATAGTGAAgagccagaggaa

attcgtgatgattatcaaaatggaaagatggcgaaatttcgagataaggagttagaacga

gagaaatttgaagaagataatttaagaagattgcagctatctaaaaaggatagacaggcc

aagcgaaagatgaatgagctagctgaaa

>contig00131 length=171 numreads=2

GCTTAAATGCGTCCAGCTGGATCTTTCAGGAACGCACAAAATTTGTAACATGGCTGCAGT

CGATGTATTGGTTAAAAACTTTCTGAAGAAAAAGTTGCCGAATGTTGACAATGATATCGT

TAATTATGTTGTAGATGTTGCAAGCACTAGCGCTGACGTATTTCAAaTCGT

>contig00132 length=323 numreads=7

aTCCGACTTGAACGCAAACCTTACAAAGCCTATtGCCTCGAACAGCAGGGgCTGGCTTGT

TGTCTAaCACTTtCAAAGAAGTGATCTCTCCGAAGGgACTGAATATCTCCTTCAGCTTCT

CTTCACTGACCCATCCAGGCAAACCGGCGATGTAAACATTCGTATCCTTTGTCTCAGgTG

ATGACTTTCTCGCGTAGCTGACGCGGATCGTCTTGCCGTCCAAATTCATCCCATTGATAC

TCTCAATCGCcTTCATCGCATGGGCGGCTGACTCGtACTCAaTGAaCGCGTATCCGAGAC

TTTCTCCCGTTTCGTAGTTCCGC

>contig00133 length=179 numreads=1

gatagtctttgtcagatacgttcttcagaggtctggtcccgtctcgtcctgtcttgcatt

tcgcttcttcgaagaagttgcgtgcccactcttaactggctactcgaaatgacgtgtcaa

atggcgcgcaagcgacgaattggtatcgttggatttggcaaaatcggacagtactttta

>contig00134 length=178 numreads=1

tagaccttgttgagtttcttctaagattctgacattgcacacatggcatggcagcacatt

acttggagctgtccaaggagcctttgagttttcctccagtgagcagagaagtcaatgtgt

tctttgatgagtgtaacaagcaagttttcgctgttgctgtagaaaatcgttttaccag

>contig00135 length=626 numreads=16

GACCTTaaaaCCTCCGCCATCCAAGGAATtGAGGTTAGAGaTAtGGCTaGAaatCAaGAG

gAaTCTtCGCAAGTGGTCGAGGAaGAGGATGCCACCGAgTTGCAGTTtCCcAAaGAGTTT

GAAAACGCCGAGACCCtCTTGAATTCCGAAGTATACATGTTACTAGAGcACCGAAAGGCG

CAGAACGAAAACGTCGAGGATGACCAGGAGATGAACGAAGTTTtCGTGAAAACCTTGACT

TACACGCAGCATTTTAGCAGCTTTAAGAACCGCGAGACCATCGCACAAGTTCGCGGACTG

CTTTCGAAGAAGAAGTTACACAAATTTGAACTGGCTGCCATGGCGAACTTATGTCCcGAG

ACGTCGGAAGAGGCTAAAAagCTAATACCCAGCTTGGAGGGCCGATTcGATGACGAAGAA

CTTCAGCAGATATTAGATGAGATCAAGACGCATAGAAGCTtCCAGTACTAACCGTGAcTA

GtAtGtGCGGTGCATGTTGGCCGGTTGTGCTgAGTTAAGGAGTCCATAGGgCGATTGTAT

GCGCAGTACGCTGTTACCAATTGTCTCCAtGTTTTGAGTGCATGGCAAGCCgAAcTATCg

TGAAgAAaGTAATTGAAAAATtAACg

>contig00136 length=675 numreads=15

aagcagtggtatcaacgcagagtacgcggggaccttttcaaaattgtccgttgttgatca

ggtcagcggatttggaggattaatataaagttgtgctagtgtggctcgtatcaattgctg

ttttAgAgTAACGCGTAaCcGTAAAAaCATGGCGAATtCGCtAAtGAGACTTGCCGCTCA

GCATAAAGGCTGCtCAGAAaTGGGgACgAACAATtATtGtCaGTTTaTCCATGGGgaTAT

GGACACCAATaTTAtCGTAACTGTACCTCATGGAGGTCAcTTGCTACcGaaTTCTATACc

GgAtCGTAGCCAGTATGGATGCGTTGTGGACGGCGAGAGAGTTTGGAgTCaTAATTGCGa

CGAaCTGTCTGGGAAAAaGTGTCGGACTACACTCGTTAACGATAAGTACACTAGAACTAT

GGCTGTCGCCCTTGTTAAGAATTTGTCCGAACTGACTGGCAAACGACCGCATCTCGTAGT

CAACCACTTGCATCGTCGAAAACTAGACGCTAaCCGCGAAATTAGAGAaGCTACATTCGG

TGTTCAGGAGGCAGCTATGgCCTACAAGGAGTTtCAAGAATTCATTGTTCGTGCAAAGAG

CACAGTtCAAGGTCGTGGATtACTCATtGATCTCCATGGTCACTCACACaaggagcagtg

ggttgaattgggtta

>contig00137 length=182 numreads=1

caagtatgaatctgatttttgctgtatggagtttcctttcaacataaactctatattgcg

agacaacgtaactgtgatccaaaaacaagatgtctctagtgccagggagccattgaatag

ttcccataccaagtcatcactaaaatttagtagcacaccctacttcaagctcgtaaactt

tt

>contig00138 length=179 numreads=1

tgaaatgggttccgttcaagattgggatcctcgcgcatcagttgtttgtccatatgaccc

acttcatgtagtgtctgcatcgagataccaaggacatattgttaaatgcagaaagaatca

tcctgacaaggattttgtgtcctgtccatataatgctcaacatattttcttaagaccgg

>contig00139 length=651 numreads=26

GATGATTCGGCTCcTCGTCTGTGTCGTACGACATGACGCACTTGTTCGTGATTAAAGAAA

TTCCAAGTACAGCCACCCCGGAATGGGCAGCCACGATAACCTCGGAACCGTGCTCATTCC

AACAGCgTCAGCGCCAATGGTTTGAATTAAGCGGAGCTCCGCGGgAGTCTCGTACGACGG

TCCGCTGACCATGCAGTAGACGCCACTTTtGGTGAAGTCGCCGTAGCCGAGGTCAGCGGC

TACCGACAgcgCTAAATTTTGCAGGCTCCGATTATACGCCTTGGTCATGGACGGGAAACG

GGgACCAAACCTGTCGTCGTTCCGCCCCACTAAAGGGTTCGAGCCAGCCATGACCGGCAT

GCCAATATGGTCCTTGATTATCATGATGTCCCCGAcAGCAAACGAGGCGTTtATACCGCC

GGCAGCATTGGTGACAATCAAAGTCTTGATCCCCAACGCCGCCAAAACTCGGACTGGGAA

AGTAATTTTATGAATAGAATGACCCTCGTAGAAATGAAATCGTCCTTGCAGACAGACCAC

AGCCTTACCCTTGATCGTTCcGAACACAAGCTTTCCGACATGACCGTGCACAGTCACAGC

TGGGAAgCCAGGAATATCTTCCCCGCGTACTCTGCGTTGATACCACTGCTT

>contig00140 length=166 numreads=3

gATCGACTAGTCGATAACCCAATATCTTATTACTGAAGCAATGGATGCTCTGCTTGGGCA

GCGACCTATATCATTTCGTGCCCTTGCTGACTTCAGCAAGCTGgATgCtCGGTcAAAGCC

caTCtGAaGAaTGTGTATGCTTGCCTTGCtAtcTCcacTCTTACAg

>contig00141 length=159 numreads=1

ggttgtgatctctacacttagaggagcacttcaaaagcgtcctctgttgatgaacatagc

tacttttgttggcttactgactgctgctgactgtacatgtcaagttatacagcacaagag

ccttacattcaaatatgactttccaagaactgctcgtat

>contig00142 length=162 numreads=1

aggtttttagagtcatgatagtggatcatcgatttctactgttgcattcgttataatgga

tcacctagtagcatatggtgaatcggacagtggtgatgatgacagcactgatccaactct

ttctcaagaaatgaaggatgtccctttggtagtgaatttcct

>contig00143 length=224 numreads=5

aTCGGCTCGAAAAGTCTTCACGTTTAACTTTGGTTTcAAATCGTCGCAGCTttGACCTAA

aTccTCcAGATACTtCGTGATGAACTCCTTTTCTCTCATCCGtcGCTTAGTGATCCTTTT

AACCTGGTACAATCGGTTTAATAGTCTATAGTTCTGCATCTCTATGTGAGTGCAGTTCTT

TCGCAAGAGaGAGTACTTCCGTTTCcAAGTAGATtCATCATTCG

>contig00144 length=166 numreads=2

tgctttcgtttagaacaagaaatgtctggagcgattggggttccaatcaaaGTTCTGCAT

GAAGCTGAAGGTCACATAGTAACTTTAGAGACTTTAACTGGTGAGGTATACCGTGGAAAA

CTGCTGGAGGCTGAAGATAACATGAACTGTCAGctaactcaaataa

>contig00145 length=193 numreads=4

CGGCTTGGTTCAAAGgTtCGTTtGCGTGGCGTCATCTCTGTTTATGGCGATTACAaCACT

GTTGTTTGGgTTCAGTTATAACCTCGAATGGGCCATAGTGACGAGATTTtCTTCAAGGCG

CTAGTTCCGGAATtGCTCGTTTCGTCAAAAACGATGCTTTACCAACTCTCCGACAATAcA

AATCAGCAATATg

>contig00146 length=161 numreads=1

gcctgtagcggctttctgatgtacctccacagtgtttgcacagagaattaactgccagta

attcaaaatgctggaggatgcccataagactcagaatccaatagagtccctttcttctgc

tagccaaatctgtgcgtggttctgcagcagcacaactagtt

>contig00147 length=182 numreads=1

tgataaatatttgtgaagttggcatgtatcgaggaccaagtatgagtgggcaaatcccac

ctgttccgtcaagtatttctgggcaatggccacctggggctagaccccctttaaatgctg

catcactgcctgtaagaccacccagttcagggactggggctacaccagaggctggtagca

aa

>contig00148 length=294 numreads=9

cctttaaccgctgggctatACGCTGGCGCATGCGGTTCAttttAACcTTAaTTTCCACTC

GGCTGCCcATAACCcTGgTGGGAGCACCTTAGACTCGACGACCTGGTTTACCAGAGAAGC

CCCACCAGACGGCGGACCAGAAGAAGAAGCcGAAACAGAcGCAGTCGAAGCAGCAGATTT

CGgAGGTGGTGGCGGTGATGGTTTTATGACcGCCATTGGCTGAGCAGGAGGCGGAGTTAC

CTTGGGAGGCTGCTtAGGAGGAGGCCCTGACACACTCAACtcAGCGgCGGcAaC

>contig00149 length=176 numreads=1

aagcagtggtatcaacgcagagtacgcgggggatttaaaaggtctgacttcgtttagaga

ctgcgttgctactttttgagagcttttttgtggaagatgagatctgtcaaccgaaaaatt

tcttacttgtagattggttctagtgagggtgacgcttcaagtggaaacggtttcga

>contig00150 length=1112 numreads=20

ttCCAATAACTTGCCATATCGATTCAACCGATATAGGTACATACAGCCCTCGAGGAGTAA

TCCTTCAACGGCTTCtGGCagTCACcAACcAAATaCGTAGTCCATGGCTTTTGTtATCAC

CGAGTCCTTCTTCTGTGGAGTCCCCTTATTACCTGTCATAGCATCAAGCTGAGCAGACGG

TGGCCTGTACGGCgTCCCCGCAAGAGGGCGCATAGTCgCTGGCTtCGCTCCAAGCGGGgT

AcAGATaCCCGaGGCGGAATCCGGGGCCGACACTCTAGCTGGAGTAGCGCAGTTGAAACT

CATTTCCGAATCAATCGAAGCGATTtCCTTTTtGATACACGGACAAaCACCAATCATGAT

ATTTCTAGAGAAgaTCTTGCAATTTTTGCTCAGGGCCTTTTTAGCTtgAATCCTTGTctG

gTaTTtGACgTGCATCCAGTtACCCGTGTTAtGAATCTcGTActGAaCGATAGATCCATA

CtGGGAAAACTGCTGCAGAATGTAGGAaGAGGCTgCcGGcGGAAATCCAAAAATCGTGAC

AgCTGTGTCATCCAGAACATCGGAACTGgTGATTGAATCGCCTTCCGTATAAAAAGGGTC

CACTTGCGCGGGCGACTGAAGAAGACGATtGTCAGGACGAGGACTGAACACGCTGACATT

TAGGTCATCCGCGCATGAAATTCcAGGCGTTCGAATCGTCTCATTACTAGCGTGATGAGG

TCCAAATGAAatACCCTTCTGTGCAAAAGGAAGATTGAAATCGGACTTGCTCACTTCCGC

TAGGGCAGGGTGTCCACCGTAGAGACCCTCGGTCGGTGGCGcaccAGatgTGTCTTtACC

CGCGGAtGCATGAGACGACAGCGGCGATGGTCTATATGTTGCCttctcattgccaccagc

aaatggagaatagtcaaactttcctaaagagatATTCCCGCTTGTAGGACCTGCTcTATc

tGGGCTTAAACGCAaCTTTCGTCCaGATGCAGCAAAAGTTCTGGGCGGCACAGAAGAAGG

GTGCTCACCCAAGAGGTAGCTGGGCAAGTATGCGCTGCCAGTAGAGGTAGTCTGCAAGAT

GGGGGACTGCATAATTGGCATTTTCAACttga

>contig00151 length=179 numreads=3

GTAGATGCTGCTGAAATAGCGTTTTGCGGAGCATTTTGTGAAGATTGCAGCGAGCAAGTA

GCAGTTAACGCTGGCGTGATAGTAATGGTGGGCGGAGCGAACCGTAcAAGGTcgaGTTAA

TTGGAgAAcAAAGGGAACACCACCATATAATGACTATGTGAGGGAGttgtttaaacaaa

>contig00152 length=150 numreads=1

gtttctagttgagagtttgtggtatcaacagtcagtcaaaatctaagcagtttcatgaag

atctttgaaagcatgatagacaggagttgctgaaactgaggtagaaaaagcaaatggaaa

agaagctagttttgacaagaaatatgccaa

>contig00153 length=1073 numreads=37

ttAAATGAttgTTTGGCTtGTAGTGGATGCATtACATCTGCaGAAACAGTCCTCATAGCG

CAGCAGAGCcAGGATGAATTGTATAAAGTGCTCAAGgAGAATGCAACAAAGCCAGTGGAG

ATTCAGAGAATtGTGGTTGTGTCCATATCGCCACAGTCCTTGGCATCTATTGCCGCGAGC

TACTCCTTGTCAATGCGGGAagCTATGTGCAAGGTTACATCATTTTTGAAGgATCTTGGA

TGCAGTTTTGTATTTGaTACTAATCTGGCTCGAAGTCTGgCGCTTTtGGAAaTAGGCAAG

GAATTTGTAGAAAGGTTCCAAGCTGTCCCTTCATTACTGCCTCTGCTGACCTCGGCTTGT

CCAGGTTGGATTTGCTATGCGGAGAAGACACATGGAAATTTCATTTTGCCCTACATCAGT

AAGACAAAATCCCcTCAGCAGGTGATGGGCTCGCTGGTAAAAaCTCTCTTAGGCAGGATC

TTGAACAAAACACCTGACTTGATCTACCACGTGAGCATTATGCCTTGCTATGACAAAAAG

CTTGAAGCCTCCAGAAGCGACTTCTACAATGACCTTTACAGAACTAGGGATGTCGATCTC

GTGATTACTACGATGGAAGTAGAGAAAATGTTTATTGAAAAGCAGATAGAACCGCGCGAG

CTCCCCtGTAGCCCTTTGGATGGAGATTTAAGTTTCTTAAGCGATGGAGCGACTCCAGAG

GTCCTTAGCCATGCTGGAAGTGGATCCGGAGGGTACTTGGAGCAGgTGTTTCGCCATGCT

GCTCAaTGTCTGTTCAATGTCGACGATCCAGAAATCAGATATAAGACACTGAAGAACAAG

GATTTCCAAGAGCTTAGTTTGGAAGTCAATGGCGAAGTGAAGCTTCGGTTCGCCCTTGCT

TATGGCTTTCGAAACATACAAAATATCGTTCAGAGAATCAAGCGGAAAAAATGTGAGTAT

CATTtCATAGAAGTtAtGGCTTGTCCGaGTGGCtGCGTGAaCGGGGtGGACAAaTTaGaG

CcgACAATAAGGACGACGCTtAAGGttttgatggataaggttgtgcgtttgta

>contig00154 length=164 numreads=1

tctccattactatgcagagggtagtgcagctccagaacgtaaactcatacctgatctgtg

gcctttgcaaaggctaccttattgatgcaacaaccttggtagaatgtttgcactcattct

gtcgaagttgcatagttcgttatgttgcatcatcgtattcttgt

>contig00155 length=790 numreads=16

GCCTCCAAGTAGTCTACCCTTGGTAGGACTGCAGGGAGCGTTACTAAAGCTTAAGGGTTG

CTGAGAAGTTCGGAAGGGcTTGGGGAAGGAAGACGTAATCAAGAAATCAGAAAATGCCAG

GATTTGCCGAAAGCATGCatgatttagtcgagcATCGACCGGAAGATaTGACTGGACAGG

CTGGTACGGACGGCCCAATAATTGGCATtCcTgACTCAGCCTCAGATTttCGGTGaCgAt

CATGCGCCGACGTTCATTTGCGCATCCTACGTCAGGTTCATTGAGTGCGCCGGTGGACGA

GTGGTGCCCAttCCCCCATCGATgCCGATCGAGGAAATGAAAACTCTCTTCcAgTATTtG

AaCGGCTTCTTCTTTTgCGgaGgACGAGCgCGGATCCCcGATTCAGAAAACTTTCGCCGG

gTTAAAgTGATTTACGATCTCGCAATCGAAGCTAACGATCGCGGTGATGTGTTCCCCATT

TTGGGCACATGCCACGGGCTACAAGCCCTAGCTGTGTGCACCGACAGTGGCACCGTCCTC

GATAACACGCCGCACCTTAATTCATCCGAcACTCTGGAGCTCTCcgACGAaGCAAAGGCC

GGCAAAaTGTTCCAGGGTTTtAGTGAACAGGAGTTaCGCAGTCTtGAGACGGAGAAGATC

ATCTtCAACAGTCACAAACGAGGCGTTAAGCTACAAACCtaCCGgAaTtCTGCGCgACTc

AGTAACTTCTATCGGGTgcTAgCTACTAaTTACGATAaCAATGGGGACGAATATCTTGGC

ACGATTGAAG

>contig00156 length=174 numreads=6

TGACGTCGGGGAATATTTACGACAGGAGGATTCCCTGATTTGACCGTGGTGCGAATCGTG

TGTCGGTCGCCTAAACGTGGgaGCTTTGCGCGCTCTTGGAATGGGAGCGTTGCTTCGGGA

AAAAGgtATTTCTGGAGACCTTTGGTTCTTTCTGAAGGGAAGGCTGTTCGTCgg

>contig00157 length=166 numreads=1

tgtcagatgcttcagcatcagtcagtatcgctttaggctaaagagtaatgtcaagcgacc

ctgtagatgcagtggatgagggacgacgaaaacccacatggaacttgaaggagttcatcg

tgcaaactccctgtgtccggaactcgttgctgtacggcatatatgg

>contig00158 length=1976 numreads=118

ttggtaCGCATCCATcGTAGGAaCATTTTATAGTTTtCAGCTGAAaGACAAaTCcAAATA

ATAGCCCTTTGCTCATGtCaTTTTtGTGGATAGAGTTGCATAGCAAGTCCACCATTAGaC

AAAAAAAAAAAACGaaCCGCgCACAAACTTGAGcAtAAGCGGAATAACGaGGGAGCTcAG

AGAAATGCAAGaTCCATCGATTtaTAGgAGgaTGgCTGTACAGCCGGCGGAcgAgAAGCT

TCAGCTGCACGTAAGTAGGGAGGTCAGCTTCAGCAaCCCATCAtCGTTTTTtGTCGAAAC

GATTAGGCCTGTCATGTcGAGGTTCCGACATAACGAACTCAAAACCCGTTAGAGACAGCC

TTATTTCTTCTTGAAGTCTTGCTTTACTTTTTCTTTtAAGAGCATTCTTATAACTGAAGA

TGTCACCTTGCCATTGTGTTATTGTCCTCTTCTCGCACACCCAAATCTCTTTGGCAACCT

GCATAATCAACCTGAAGTCATGGCTCACAaGAACTAAACCGCCATCgAAGTTGTTAATAG

CGTCAGCTAGAGCATCTATAGTTTCGATGTCTAAATGATTTGTCGGTTCGTCTAGgAGTA

GCAGATGTGGGTTCTGATGTGCCAACCAGGAAAATATGATACGGCTtCGTTGACCGTCAG

AAAGGTTTCGAATAGGGCAAGTCTGTTGtCGACCAGTGAGGCCATACCTTCCCAGGTAGC

GTCTGACCTGCTCCTCCTCTTTCTCCTCTGGGAAgCACTTCATCAACCATTTCAAGGAcG

ACATGTcgAGATCTAAGATGTCTTGCAAATGCTGGTGAAAGCGGCCGATTTTCAAATGTT

GATGCTTCCGAATtACACCATCGGTGGGTACAAGTTCGCCGACAAGCAGCTTCAACAACG

TGCTCTTTCCGGCTCCGTTCGGACCAACAAGAGCAACTCGCCGATCTAGATCCAAGCCGA

AGTCCAAGTCCTTGTATATCCACTTTTTAtCAGgTtGCATATCTGAAACTGACATTTTGA

ACTTGCAGTACGGGAGGAGGCAGCTTTCCACAATCCGGAAAGGAGAAcGAAACATTTTTA

TCTTTCACAACCTtCTCAGTCAGTCCTCCTTCAATCATTTTGGCCAgagTCTTCTCTTTG

CTTtGCGCTTGGCGTGCTAGCTTGGCGCTTCCATGGCCAAATCTGGCTATGTAATCCTTC

ATGTGCGCGATCTCATCTTGCTCGCGCCTGTACGCTTTCATTTGATTTTCCTCCAACTCA

GCTCTTGTGGTGACGTATTGGTCAAAGTTGCCCGTGTAGGTAAGCAGCTTTTTGTTTCTC

ATTAGTATAATATTCGTACAGACTCCGTTCAAAAaGTcTTGCGAGTGGGAAACCAGAAGT

AAAATTCGCTTGTAGGTCTTTAGCTCCTCTTCTAACCATACGCAAGCCTCAAGATCAAGA

TGGTTTGTGGGCTCATCAAGAAGGAGAATGGACGGCTGCACGAACAaGgCACGGGCCAAA

GCTATCCTCATCCGCCAACCTCCcGAGAAGTCCTTGGTTTTCGTAACTTGCATGGTAGGA

GTAAAACCTAACCcATGGAGAATTCTTGATGCcgTCGCCTCTGCTTTGCCTGCCTCAAGC

TCATCTagCCGTtCATAAaTTTCTAAAAGCCTTTCATGAATCGTATCATCTTCGGTCATG

TGTGCTAGCTCATCAGCTTCAGCTTCCAACCGCTTTCGCTCTTTCTGCACTTCCATAACA

CACTCGACGGCTGTCTTATCAGAAGCTTCAATCTCATTTGTCAAGTGAAAAATGTCGAAA

TGCTTAGGTATTACAATGTCACCATGAGACAAAGCATTTAGAAAGGTTGATTTGCCACAA

CCATTTGCACCAAGAAACCCATATCGCCGCCCAGTGTtAAGTTCAACTTCAGCATCCACT

AAAaGCTCAGCTCCATGATATCTGAGAGTTATTTGACCTAAATGTAAGTctcTGGA

>contig00159 length=730 numreads=106

GTTTgtCCcATAACGTCTTCTTTTCCTCATAAGCTTTCTAACAAAACATACTTTCCATAA

CCTTCCTTGGACACAtaTTTTtGTACTGCTAAGTCTCTTAACGAAAATAAACAACCTTAA

GAATGTTCGGCTAGCCGGAATaCGCAGGCGGGTAGGAGGAGCCGCTAGGCGGTTTACGAA

GAGAGGAGCTGTTGtAAGCGATAAaCGCTTCAGCGCGTTCAATTCCTTCTGCAGCGCGCT

GATCCGATTCTTCAGTtGGGTATTCTCTTCcGTCAGCTCCCcGACTCGCATCTGCGTTTC

GATGTGTTTtAGCTTGGCCTTGGTACGACTCTTACGCACAGCTACATTGTTCCTCTCGCG

CTTAATGACATACTCCGGAGTGCCCTTtGCTAACAACTTCCTAGgCTTACTGCGCGGGGC

CTTGGACACATGATCGGTCCCTTCTACTTTCACGGGgCTCGGACTAAGCGGGGTGGTGTT

TGCATCACCATACCTTGTTCCGAGACCGTCCAATGCAGATTTCAAGCGGTACGGGTCAAT

AGGAaGCGCGTTCCGATCACTATCCGAGTCCGAGGACTCGTCTTCCGAGTCTACGTCAGT

GGTTGCAGAGCTGCTGTTGAGCGACTGTAGCTGACACTTTAAGTTCAACCTGTTATAGGG

AACGAACGAGCTGCGCGACATATTTTTGGCACTTATTTGAAACTGAGGAGACGGGAAGTC

CCCGTGGTCA

>contig00160 length=853 numreads=19

ccACACCGAGTTCTAAGGAGGTGCTCTATGACGCCAGTAGGCATGCGCCAGAgAAgCAGC

TGAgCtCACTGCAGAGTTTcTTCCCCTTCCTGAATTTCTTTTAAGCTCAGCAACTCcAaG

TgACCCTTCCCGcGGgCTtCTtGcGAGATGACGTCaCCGATGGGACGgTATTGAccAGGG

TCAATCAAGCACACCATGTCAAGATTGCCGCATTCCcATTCcTCgCTTtcAATTtttCtA

CaCAAAGGGGCGAGcTTTTCACGGgCTttCCTtGcTGcTtCcTTGGgTAGCGAAaTCCGG

ATtCGCAtCTGAGCTCGCTCGATCGGGATGGACTCCTTCAGCAACCTGATCACCTCCAAA

GCTTGCTGCTTCGTATTGCGAGTGGCCTTCACagAATAATGAATTTCcTTtCATAGCACT

CTCGATCACCCCGACAGGgTAAGGCCTTTTAGTCTCAGGATTAACACTtTTGTCGCAGAC

GATAGTTGCTATATCGCGgAACATCGAATCTAGTTGCTGGGATCGCTCTTTCTCCGaCAC

CTGCAACTCACCTTtGgAGAGGatctgcaaacaacattgcatttgatcttcagtaccaaa

agccttaacaagatcttctttcttagctagctgaccttttgaaacattcagaaaaacttg

cgggTTTtGcAAaaCTTCATCGATATCCTTTtCTGCTCCTTttCTCCATGaCATGACTTT

ATTCTTATAACAAGCTATTTCAAATCTTTTCCCAGTTTTTTTCAGACGCACTATTGCAAt

GTTGgTTAATCTAATTTGATTTgtAGGAGTGAaGATAGCAGCcATGCTGAATTgAGACaC

TTTGCACTAGCTT

>contig00161 length=151 numreads=2

TAAGTGCCTTTCGTTTGGCTGACCTGGTGCTGGATGCCCGTGTGATGGGTTCAAGAGTTG

AGCTGTTACGAATTGGCGTATACATATTCTTTCCAGTAGCTAGTTTTTtatGCCTTCGAC

aaGcctaagttcttcgagatacgttaccaag

>contig00162 length=402 numreads=38

CTTGGACGTACAGCTGACTGCTATTGGGTGCTCAAAAGAGGACGAGGAGAAGCTAGAAGA

AGCATTGTCTGGAATGCGCTTGGCAAGTAGCCAGGAAGATcTTTTGGAAGGCCACGAACC

GCTTTCTGCGGAGACCAATGAAAGCAGCGAATCTTGTACAAACTCTGCCTCCGACTCCGA

AATTACTGCCGAAAACGAAAACGATGTTCAATTGTCTCACAACGAAAAAaGGCCCcTGTC

AGAGCTTGGCTCGAAATTCGAGCGCCTTTCCGGTCTATCGGATCCTGAAAGTAACGGATC

GGACGGGGAAGATCGGGACATTGAAATGAACGACAACGCGTTAAGCCGGCCTTTCCGAGA

CTCACCAGTGGAACGTGACGATAGGGCGTCGGGTGGGCCGTT

>contig00163 length=181 numreads=3

ACAATGTAAAGTAACAGAAATGTCTCGGCCTAGGTACTATACTCCAAGCGAGgTCAGcTT

AAAGAATacTATAAAAGAGCTATGGGTATCTTTtCTtGgTAACGTCtATGACcTGACTCC

ATTGTgCACAAAaTATGCTGGAgACATGCTTCtAAAGCCAATAATtGcTTcTGGgtGGAA

A

>contig00164 length=111 numreads=1

actctcctgcggataagtgcttctgccgaaaatgtcgttggaaacgagtcccttaaacat

gtccgtgctcgtcgttggttctgaaattcgacttgttatgcgaatattctt

>contig00165 length=170 numreads=1

gccctaaatttgcagattatttgaaattgttgaacggtgaaggagcataagctggaagtt

ttctgatctaaatgtagcagaaaagatgtcttgctcgagtttcacacttcatatgctgaa

gaatgtttgctttgctaatggacattggtctgttgtgcttttaagaagac

>contig00166 length=191 numreads=1

gctagttatttgttgcaaaccttgtgaaaggagggaaggccaagtgttactgttggctat

gtaaagttctataagcaattcacttgcaaattctgggcatgggttccacagaggagatta

caaagcactctgtacacaccttagtatttcgatcaataaagcgaacacacgacatgtttt

tggtctgataa

>contig00167 length=165 numreads=1

gtattgtagaccatggcatcagccaagtgataggaaaggaatccaagcatgtatgttaat

ggtgatttggataagttactgttaggtattgcaacagcacaaatatgtcagtcgctaggt

tggcatggcgtgcacaaaagttcacattatatcttgaccgatata

>contig00168 length=240 numreads=4

AAGCAGTGGTATCAACGCAGAGTACGCGGGGGTCGCGAGAAAGAGTTTTGATAGATCAGT

GGTGTCAaGATcAagCCGCGATAAATTGTCCTGATTAAACAGAATGGCAACATCTATTAT

GAGGTTAGCCCGACCAGgCAGTATCTGTGGGTTCTTCaGTCGAAGCCGCCCTGAaTCTGT

ATGCTCGCTCATGTGGCTGCGCAATGTGCATCGGCCAGcAtCcGGCcaCCGCgacgatga

>contig00169 length=180 numreads=2

AAGCAGTGGTATCAACGCAGAGTACgCGGGACCTGCATGTTCTCCGAATATTCCGAAGGT

GTAGAAATATTTTCTAAATCTACGGCAGATTTTGTTGTGCGTCGGATCATAAGAGCAGAG

CGCAATGATTTAAAGGGATTTGCAAAACGTGTTCGACAAGGACGCACCAGAAgatcgatt

>contig00170 length=176 numreads=2

ccgtgaatttccgaaacgaggacgatgcaaacacggggagctgaacagccgtaGATCCTG

CTTGTCGCTCCCATAAGAAGCTCAGTATCGGATTGGCCCTGATTCGACGTCGAAGATGAA

GAACACCTGCAGGAAGAGTGTATCCCTTTTGAACCTACTGATCATAGCGCGAGAGa

>contig00171 length=312 numreads=4

gataaagttaaggaTCAAGCCGAAGTAAATAGAGCTTATTATAGTACTGTTGAGGAGCTA

CCAAGtCCTGTtAaTGCAAAGGTGAAaGGCaCTGTGCCTTCATGGCTaCGtGGAaCCctA

ATTCGAgTTGGTCCtGGGAAaTaTGAaTGGGGTACTGaCcaTTATcGCCATGCTTTTgAC

GgAGACGCTATCGTTCacAACTTCACCATAgAGgACGGAGCtGTTTCTTTtAaCtCAAGA

TATCTGAGAAGCGACTCGTTCAGGGAATCTGAAAAGCACAACAGAATAAaTGAGATCTCA

GTTTGGCACTGt

>contig00172 length=140 numreads=1

atgcaccagtctggatcgctcgcctgaccgagactgaggaatgaatacatgtttacatga

tattggtctaaaattggtagttgcctatgatttgtgttacgtacctatcggttcgcttgc

ttagattgctctttacttat

>contig00173 length=633 numreads=11

cTGAGATACTTGCTGGTCGAAaCTTGCCTGGTGTTGGCTCATCCGTTCCTCAAGCATGGG

TCGGAAGTGGCGAATCAAGCCCTGAACAGGCCAAGCAGCACCGTCTCCcAGAGCaCAAAT

CGTATGCCCTTCAATCTGCTTGCTCACTTCCCAAAGCATGTCAATCTCTCcTGTCTTGGC

ACGACCGTCGACAAaTCTCTTCATAATCTTGTTCATCCAGCcAACTCCCTCACGGCACGG

CGTGCACTGGCCACAGCTCTCGTGTTTGTAGAACTCAaTCAGTCGTtGGATGCATCGTAT

TAAATCGCACTGCTTGTTCATTACAATgACGGCTGCTGTGCcGAGGCCcGTCTgTgCCCG

AATAAGGTCGTCGAAGTCCATCAACACGTCCTCGCAAACCTtGATCGGAATAAGAGGGGT

GGAGGAgCCACCAGGgATCACAGCTaGTAAGTTGTTCCATCCGCCTATGACACCGCCAGC

GTGCCTTTCGATGAGCTCTTTCAGCGGGATAGACATCTCCTCCTCAACAGTGCATGGGTT

GTtCACGTGCCCGGATAtGTTGAAAAGCttcgttcccgaatttctgggacggccaaggga

tgcaaaccagctacctacctaacttctacatat

>contig00174 length=222 numreads=3

AGTACGGGGAAGCGTGCATGTGCGTTTgTGTGTGTTTCTGAGAGCGAAGAATccACGCGT

ACGGTACATGAACGAGACGTTTCAGGTCGCGAAATGCCTAAaTAAAAAGAACAACACCAA

TACGGAAATTTCGTAcctGCAgAACaTATTGCAAgACCTTCaTAGGCCCTaGCCcAAcGC

GcaGGCCCtGATAGCTTCAGTGAAAGCGTTAATAAcatgtgg

>contig00175 length=538 numreads=10

aGCAAGCATCTCCTAGACTCGGGAtGAaGAACAGATATACTGACAcGTTGCGCAAGGTAG

CcACCATCTTTACGCcACTCCTTATTTtGGTAAACCTCCAAAATtGTtCctGTTGACTtA

CTAGCAAGCTTTACAACGCAACAATAaGGCAaaGGTCCTTCTTTGAGCTCACCGAaTCCG

TACCAACTCTGCGCATTCCTTTACcTAAAaTATCGCGTTCCTTATCaGCTGCTTTACAAG

AGCCAGTCGAGCGATCtGGGCGATGCCGaaaGTGAAAaCATAtAAACAAaCCCCTcTCAA

CGAAtAGGGAAAAGAAAGTTCTAAGaTCAGCCGATCGAAACCcAGaCGGAATGTTCGCGA

CaTAAATGTaGgTGAATCCTTCCCTTTcGAAAtCcGGATCAAAATTTGCTtcAGGtgATt

CAGTGTGCTCACAAACaGCTGGAATAaCTTCACTtGACGTTCCAaCATtgAgACTCTtGA

AAGCCATCTTCCgCTTCGCTTTCGACtGATTTTATTtCGTTGTTTaTTTTTGCTCGTA

>contig00176 length=167 numreads=4

TtCATATTAAATGTAGTTTATCACTAAGCATATTTGGGTTGTTATTCTTGGTGATGTTGG

TCTCCACaGgTTGGGATTAGATTGTCTGAGATAGAATGAATACCAGTCTAGAAAAAATCT

tGAaTGAATtATTTTGtCTTTTTCATGCTATTTAgaattctattttt

>contig00177 length=488 numreads=116

AAGCAGTGGTATCAACGCAGAGTTGGAGCATCTGCTTTCTTCAACCCAAAGAATTCCAGA

ATTCTTTCGTTGTCTGAAACCTCTGTGTCAATGTAAATAAAGAGCACTTTGCCCTTGAAC

AATTTTGCAGACTCCGTTAACTGATCAACAGTCTCCTTGAAGTAGTCAGCTTTCTTGCTT

ACAAAAGCCAACATATGCTTTTTTAtCTCGCCTCCAAAGATTTTGGGTGCAGCTTCATCA

CTGAACTCTGTCAGAAGGGCAAGAGATTCACCTTTCACAAATGCTCTGATAGCAACAGCA

TCTGCAGCCCCTTCATAATCAGCCCTGCCATCATCAAACTTTTTGAAAACAACAATCTTA

TCAGCAGTGATTTGGTAATCTGCTCCAGCTTCATAATTAGTGATAGCGAACTCTAAATCA

TCCACACCCTCAGCAGCCTTTAAAAAGCTTTTTGCAAGATCACTCTCATGTGATGAGAAA

AAGCCAAC

>contig00178 length=462 numreads=6

aagcagtggtatcaacgcagagtacgcgggaccttgaaaccgcccaagaagtaaaGAGCT

TTGTTGAtGGCcGTGATgtAGCTATtgTTGGTttttCtCgTCCcATGATGGTGATCTTGC

AAAAAGCTTtGTAAAGGCTGcTGAAGGtgTAGATGATTTGGAATTTgCcaTTACAAATCA

TGCAGCTGGAGCAGATTACCAAATCgCTACAGACAAAATTGTTGTCTTCAAAAAGTTTGa

TGATGGTAGGgCAGATtATGATGGaGCTGCAGATGATGTTGCGATTAAAGCTTTCGTGAA

AGgTgAaTCTATTGCccTTGTGACAGAaTTTAGCGATGAGgCTGCACCcAAAaTTTTTGg

aGgCGAGATtAaGAaGCaTaTGTTGGCGTTTGTAAgcaagaaagctgaagacttcaaaga

aactgttgatcaattaactgaatctgcgaaattatttaaagg

>contig00179 length=564 numreads=93

AAGCAGTGGTATCAACGCAGAGTACgCGGGGCTTTTCAACAAAaCATCGTCTACCTGATA

GTACACTTTCCTAGCGGCTTTTAACCcTAGAGATAGgCTtGCTAaTACAGTAAAATGATG

TTAGCAGGGGCTTtGTTTTtGTCTTTGCTGTGTGTGGTGATTTGTGAAgTAAaGGAAGAG

GAAGAaGTGTTAGTGATAACCACTGACAATTGGGATGAAGCTGTGACAGCAGATGGATAC

GTTTTGGTTGAGTTTTATGCCCCTTGGTGTGGACACTGTCAGGCCCTTGCCCCGGAGTAT

GCAAAGGCGGCAAAGAAGCTAAAAGATTCAAAGTCGGAAATAAAGCTGGGGAAAGTTGAT

TGCACAATTgAGAAGAAACTTGGTGAAAAGTTTGGTGTACAAGGTTTTCCTACTATCAAG

TTCTTTAAAAaGGGGACCGTTGTGGAATACACAGGGGGACGAACCGAGTCAGAAATTGTT

GGGTGGTTGAATAAGAAAACTGGACCACCTGCAAAACACATTGAAACCGCTCAAGACGTT

AAAAGCTTTGTGGATGGTCGTGAT

>contig00180 length=230 numreads=4

GAATTCGTCGTTTAGAAGCATAGCGTTTGCTAATCGAGCTCGTAACTTTGTACAAGTATg

gCCAAAGACGAGAAaAGCGTTCGTGAAAAgCTAAATGAGTTTTTGCACGAGAaGAACTAC

TTTACTGATGGATTGGCTTtCGTTgAaGGAAAGaCTGgTgTAAaCaGAcTTtACCTGTTT

gCAGgTTGCgCTGGaCTacTGgcGcTTTACTTGATTGGTTGggtATGCAt

>contig00181 length=778 numreads=10

ccaagtagcataggaggaggtgagtttcctggatacggtcccgtaggaatgccaggaggt

tccatggacgttaatcaatttgaggatcgcccagggctagatgaaggcgttggtggacca

atgaatagcttcatatctggtccAaTgAATGGTTTCaTGTCTGGTcgAaTgAaCGGTTTC

AtGTCtggTCcAATGGAcGGCCCTGGCTcAATaGGAATGCCTGGTCttAGAGGTTTCATg

ggaccacaaattactggaaacccagaggagaaaggcgagtacgccgatagtgaccaagga

tatgacGACCCAGCTACAGCAGGTTTTGATGGGgCCCCACCTGGAATCGAAAGTCcACAT

TtCaGGgACACAGGAAAtCCTCAAGGACAAGGACAAGAGTTTCAAGATCTGGAAGGCgCT

CCTGACGCTGATTCGCAGTTCGGCCACATCGCTCCACCGTCAGCAGAATCGTAcATGAAT

TTACCAGCTGACATGAGTGCGTCTATGAGTAACACACTTGACGCAATGGACAAAGCCAaC

ATTCAAGGAGACAGCGAAGTAGGGAAGATTGTTCGAGCGGCGAAGGGAAAATCcGaCATG

AGTTTTTCGCACAAAGCATTTGATCCCGCCGTAATCTCtCCATCTGAAGTTGGCGTCCAA

AACGGACcTTCAAtCAACGTGCCcTCAAaCACGAGAGCCAGTTTtGAGGACATGCAAATG

GaCCcAacAGTTGCTgACCAAGAGGGCGGaTGgAGTAGCGaGCAGAGAagCATGGACa

>contig00182 length=2425 numreads=247

AAGCAGTGGTATCAACGCAGAGTACGCGGGGCTTTTCCgtGCGGgTGCGCGAGACTGTtG

GCgTCTTTGAACcggaCAgTtATCGATACACTGAACcAtGGCTGACAGCGAGGAAACCGa

GACTTTCGCCTttCAGgCTGAAATTGCACAACTGATGAGCTTGaTCATCAACACCTTttA

TTCGAACAAGgAAGTTTTCCTAAGAGAATtGATatCCAACGCCTCTGATGCTTTGGACAa

aaTTCGGTACTTGTCTTTAaCTGAtccAaCAGTGCTTGAGACTGGCAAGgaCctcAAAAT

CGAGATAATTCCCAaCAAGGATGAAAAGACTATAACGCTGgTTGATTCtGGCGTTGGAAT

GACTAAGGCTGACCTGGTCAATAATCTAGGTACCATAGCCAAGTCAGGTACCAaGCATTC

ATGGAGGCTCTTCAAGCAGGAGCTGATATATCGATGATTGGCCAaTTtGGTGTTGGTTTC

TACTCGGCTTATCTTGTTGCTGAGAAGGTCAAaGTTATctccAAGCATAATGATGATGAG

CAGTACATTTGGCAATCAGCTGCTGGTGGGTCCTTTACTGTGACAAGAGATACAGTAAGT

GAACCTCTTGGACGTGGAACAAAGATtATTTTGTACATGAAGGAAGACATGGTTGACTAT

CTTGAAGAGAAAAAAATCCAAGACATCGTCAAGAAGCATTCTCAATTTATTGGTTATCCA

ATCAGTTTACAGAAGCAGAAGACAAGGGACAAGGAAGTAAGTGACGATGAAGAGGAGGAG

GACAAGGATAAGAAAGAGGATAAGAAAGAAGGGGAGGACAAGTCTGACGAAGAACCTGAA

GTGGAAGATGTAGAGGATGAAGATAAAAAAAaGGACAAGAAAAAGAAAAAGAAAATCAAG

GAGTCCTATACTGAAATGGAGCAACTTAACAAAACCAAGCCACTGTGGACTCGTAACCCT

GATGATATAAGTTCGGAGGAATACGCAGAGTTCTACAAAAGTCTAACAAATGATTGGGAA

GAGCAtCcTTGCTGTCAAaCATTTCTCaGTTGAAGGTCAACTGGAGTTCCGATCGATTCT

GTTCGTTCCCAAAAGAGCTCCCTTTGATCTTTTTGAAAACAAAAAGCAGAAGAACTCCAT

CAAATTGTATGTGCGCCGGGTCTTCATTATGGAGAATTGTGAGGAATTGATGCCAGAATG

GTTAAACTTTGTGAAAGGAGTCGTCGATTCTGAGGATCTGCCATTGAACATCTCAAGAGA

AATGCTTCAGCAAAGCAAGATTCTCAAAGTAATTCGCAAAAACTtGATAAAGAAATGTCT

TGAGTTGTTCCAGGAGATTTCTGAAGATAAAGATAATTACAAGAAGTTCTATGAGCAGTT

CAGTAAGAACATTAAGCTTGGCATTCACGAGGACACgCAGAATCGTAACAAGGTTGCTGA

TTTATTGCGgTACCACTCATCaTCAGCGGGTGATGAAATGACTGCTTTAAAaGACTATGT

ATCCCGCATGAAGGAAAGCCAGAAAGATATTTATTACATCACTGGAGAAAGCAAGGACAT

TGTTTCAAAGTCGGCTTTTACTGAGAAAGTTTTGAGAAGAGGCTTCGAAGTATTGTACAT

GGTAGACCCCATTGACGAGTATGCTGTGCAGCAGCTTAAGGAATATGACGGTAAGAAGTT

GGTTTGCATCACTAAAGAAGGACTAGAGTTGCCCCAAGAGGAGGACGAAAAGAAGAAACA

AGAGGAGCTTAAATCcCAGTTTGAGAATCtgTtGTAAGGTCATCAAGGAGATATTGGACA

AAAAAGTTGAGAAGGTGGTTGTCTCTAATCGACTCGTTGATTCGCCGTGTTGCATTGTTA

CCAGTCAaTATGGCTGGTCGGCTAATATGGAACGcATCATGAAAGCCCAGGCCTTGCGTG

ACACAAGcACCTTAGGATACATGGCAGCAAAGAAGCATCTTGAAATTAACCCAGAACATT

CCATCATGGTTGCCTTGAAAAAGAAAGTTGAGGCGGATAAGAACGACAAATCTATTCGAG

ATTTGGTCGTGCTTCTGTTTGAGACTTCACTCCTGTCTTCTGGGTTCACGCTTGAGGACC

CGCAGAGTCATGCcAAGCGCATTTATCGGATGGTCAAGTTAGGCCTTGGTGTCGATGAAG

AGGAAGTtGAAACATCTGTTGGAGCAACcGAAGTCAAAACTGAAGAAATCCCACCTCTTG

AAGAGGACGGCGATGCTGATCAAGCAAGAATGGAAGAAGTCGACTAGTTTTATTTtCACT

AgATGCTGTCCtCGTCCTTGCTTTCTTACGGATTAAGCAAGCCATAATCGGTTGAAGTGG

ACATTTCATTATGGAAATTTCTTGTCaAAAAAAAaTTTtCAAGTCGTTAAGCAAAAATAG

CCGCCGGATGGCATAAGACcTaaat

>contig00183 length=392 numreads=2

aagcagtggtatcaacgcagagtacgcggggatggataagtctcttgtttattatcctct

gagagactaaggttctcttttgacagacacttatgatctgttgctgttttgttggtttgt

cttattgaaagacaccaataagttggttggaattagtcaagaattttcttaaatcgatga

tggtagagtttccattggatcctcacctttcAGTTCGCAATGGTTTTGTCACTAGATGAC

CTAAATGGTTCTATTATTTTAtctgaaatcggagtgctacgtgctcacagcatgtgtact

cgcttgcaacctaccgattccatgtttatgttcttcctggcaacgtggacacttagcctc

cttacctttgaaggcgttgggtaacaactact

>contig00184 length=809 numreads=38

GAAGAATATCcAAAATGCAAAGgACATCGCcGCATTCGTTAGAgATACTATAGTTGCCAa

GGCAAAAGACTTCAAATGCGAGAATGTGCTTGCCCAGTCGATCTGTGACAGCATTCGTCG

TGTTGCTGAAAGAGTTGGAGTCCAAGTTGCAAGGGTTAATCAGATActGAAAGAAGCAGT

TGCAGATGGTGTGTCAGGAGCAAAAGAGTTATTTGAagCTGTTAAAAACAAAATACTGGA

AATCGTGGGAGATGAGGAAgCACTATTGGACGAAGAGTATGCGTACATCGCCCTTCCTAC

cAAgTGCGAaGATGTTCTGAGTGCTGAGGTCTGTGCCGATCTTCGGAACATTGCGAaGAA

GCTTGGGCAAaCTGCGCAGAGTGTCGATGACGCGGTTCGCGCAGCCGTCGAAAaGAAACT

GCAGAAGTCCAAAGAAATCATTCAGTTTGTTCGAGACAGCATCGTGGCCGCTGCTAAAAa

CTTCCAATGTGAAAACGTTCTTGCGCAACCtGTTTGCGATCGAATTCGAGAAGCAGCTAA

AGCGCTTGGAGTAGAGGCTGCCCGAGTGAATCAAATCATAAAGGAGGCTGTTGCTCAGGg

AATTACCCGCGTACAAGACTTCCTGGATTTCATCAAGAATAAAGTTATCAATGTTATTgg

AgACGAGCCTCATGATGAGGTCAGTGTTGAAAAGCGGGGGATAATTAAGGACGCTGTTCT

TGATGTCGCGGaGAAGATTCTGGATTCATACAAAGTTGTCAGTGCAAAAgTGCGCGCTAG

AGTGATAGAGATCATacgTaCAAAAGGTA

>contig00185 length=190 numreads=2

AAGCAGTGGTATCAACGCAGAGTACGCGGGGACGCTTGCAGAGGACCCTGGGGAGCATAG

ACGTACACAGACGCACGAAGAGTGAATATTGTAGTAAAAaCTTTAATCAGTTTTGCGATG

GCAAACGTTATTTCTTGCTAGGAAGTATGGTGAGCGAGGAAAGACCGGTATTGCAGAACT

TTTAAGGAAT

>contig00186 length=133 numreads=2

cACCGAGGACAACGTGGTCTCGTGCTTGCGGCAATTGCTTGGTGCACTGGACTACCTGCA

TGCGATGGACATAGTTCATCTGGACGTTAggccaagcaacatgatcatggaaggcactgg

tctaaagcttatc

>contig00187 length=1273 numreads=57

cATCGTTGTGTAGTTGTTCACATAGACCTGATCCCcAGTTTCTAAACGTGCTCAAAGTAC

ACGTTCTCTGCCATTGATACCTTCCTCAATtCcTGCCTCAACTTTTTCGGCGTCAGCTCG

TCCTTTCGAGGTGAAGATtCGATGTGCAGCCACAACTCTTGGTCACTCTCTGgagTCTCG

TCTAAGGAGATGGTCtCGTTCCATCTCAaCACCTcTtCACCTGTCCACGGGCAAGCCATC

ACCAgCGGATATTCaTAAACGGCTCCACGACCTCCAAAGTACACCTTCTtCTCCGAATtC

GGGTTAAGtAACTCcGTTTCGTACGAAAAACcGTCTCcTtCCATTCTTGAATCTTTTCAC

CGTGCATaGACAGAACAGCGCCCTTaCAATCcACgAAaGTCGTagCACCGTTtCCTTTCC

CGTCAGTCAccGATGGACatGTCTTGCAATATAAAaCGAATTCCCGTGGAATATAATCCT

CATAGCGATACAGCTTTtGGTCGCAaCCCaGATACTTTGGAGGCAAAGCCAAGTCAAaGT

GGAGCGGCAAGTAAAGGgCGCTGTTTACAATCCCAGGTACATCTTCGTCAGGTTGCACAG

ACTTCAACGCTCCGAAACCCCATTCCACAAAGCCTTTTGGCGAACGCTTTGCGTAAAATT

CCAACATTTGTTCAGGAGTTTgAAAACCTTGAAACCCTCGCAATACGACGACTCCGAACT

CCTTAACAAGTGCCGTCAATGCATACGGCGAAATGACGTGTGCCGAAAGTGGCTGAAATT

CCGGTCCCTGATTCACCTTCAACACGATGCCGCAGCTGCCTTTAACCATTGAAGCCTGAA

TtGATGCCCGAGTATTGTTTTGCACAACAAAATCTTCCTCGAAaTACAGTCGCAGAAAAA

aCCAGTCCTGGTCTTTGTACAGGACAGATACGAGgCAGGAGTTGGGCACAATTtCCTTCT

CAATATCTTCTCTGTGGGCGACTAAAAaCTGCGCTGACTTGGTgTCGAAcACGAcTGAAT

GgTGCCAGGCTGTCCTCAACACGCCTTGCACCTTGTTGTGGCCTTTAAACAAGTCCAAGg

AGAACTTtCcAGATTTTGGTGGATGGTGGTGAATGCTTAATCTtAaTATAATCAGTTAGC

TTTGTTTgCAaCTTCAAGAAGGCGTCCAGACATTGTCCTTGCaccatcataccacgtgcg

atgtttcggacaTATCTTCGACTAGCCTTGGAGCTGGGTGATCCACCCGGAGaacATGGA

GATCGGTTACCCa

>contig00188 length=618 numreads=10

cTCCAACGTGAAGTCCAGCGGTGCACAAAAGCATCTGCCGAGAAGGAAGAACATTCTCGA

AGGAATAGCAAGAGCGGGAAAGAAGcaTTAGGGAAATTGTTGGCAACAGTGGACTCTAGC

AGTGTGCCATCGGGAACGGAGGAAAAggGGCTGGTGGTCACGTAGTGAAACAAccAaCAC

CGAATGAAAGCTACGGTTTTCAGCTGAAttttaatctcTTTAAAAAGAAAGACTACCTGA

TGTTTCTACTAATTATGATGATCACtaactttacatattacattcctattactcatctGG

TGAAttaTGCTGAgggaTTTGgCACgAGtcctGGTAacGCAAGTTTGCTGATCACGATCT

GGTCACTGTGCAACATCGCcGgacGgaCGCTATTCGgACACTTCCTGAGTTAtCGCCGCC

ACTGGCTCATGCATATTTACCAAGTCTCCATGTTCTTCAGTGGGCTGTCcACCATCgTGg

CATCAGTTGCCGAGAGCtACTACCTCCTGgTGaTATATGTCGTCACATACGGCTTCCTGG

ATGGctcGTTTATCGGTCTACTGTCGCTTGTCACTCTTGagATAGTGGGcATTGAaGATT

TgggACAGGGGTATGGCa

>contig00189 length=179 numreads=1

atcatgtttcagtgaaagtttatagacaagtatgtatccacctcaagtccgccaagtaca

gctgacaagcaatggctctggagtatcaggaagagaacccattatgataatactgaaaat

cttcgtagtcaaagaagaagtctacctattttctatggtcgcagagctcttgtggaagg

>contig00190 length=193 numreads=5

AAGCAGTGGTATCAaCgCAGAGTACGtCGGGGACGGATAaGTCTCTCcTGTATtCATCTG

TCGAGAGCTTAAGTTATAGCgCAGACATTtgTTAGGACAGaagatATGCAatCTATaaaT

GGggaTGAAAAgcACCCTcGCcctATCGATgAgAAGCCTCGGTCTCCCCcAGGGGCGCgt

tctaatcgtcccc

>contig00191 length=107 numreads=1

aagcagtggtatcaacgcagagtacgcgggggatgtagcatgggcaattaaaaatggcgg

cgagcgtgcccgttccttgagtgaagattttgaggaatttttaagcc

>contig00192 length=100 numreads=2

gtgggcagcgttagcacattcaaggacagcactgagttgagggtAGCcTACCCCCGTCcT

TGATTCGCGTCGTGCAAACAGAACCTgggccaataccaac

>contig00193 length=1478 numreads=32

aaggcatGGGTCATTTAAAGAATTTAGTGGAAGATTTTTtGCAGGATTGCTGTGATTCGT

GTAAGGAGACAAGCGGCCATGGTATCTGTTCCGAAAGAATTCGTGTATCCTCTACaGTTg

TCGAgAaGTTGAAAGGCaCAGATGGTGTTAGCCTGCTTTGCGCCTTACAAGAAGTtCTGC

GAACCTGTGAGAAtGCTTCTACGGGAAAACATTCACCGTGTTTAGTGACATACGTTCATA

AGGTTTTCATGGAGGTGTTGTCCTCATCGACcTTCGgCTTGCTTCCTGCTCAATCTGGCT

TATTTGATGTGCTTCTGAGGATTTGCAaCACTCTGCTAGATTTGCCATTTACCGTTtCTC

CAATAAACGTCGAAGAGAATTCAAGTtCCGAAGCTGCACTTGAACGAAATATGAAACTTC

ATCCGTtAGTTTTCAGTGCTTGTTTCAAATTCTATTgTGCTGTGCTCTCTTTGGCTAGCG

GGCTTtACGAATCGAGGCATACAGTGGAGGAATGTAATGATGATAGTGAGATCACCGAGT

ATACGAGTATCTGTTTggAGGACAGCcACATAATTCGTGAACATATAATACCAGTGTTtA

aGCAAACTGCTGCCAAaGGCcTATATCAGGCtGTAGCACATACCGGGAAACATGCATGGG

TAACACAAGTGTCAAAGCGTGTCGCTGAGTATGTGTTGAGGACGTAtGTTCTCTTGTCGA

GGCATGAAaGTGTCTCATCTTTGCTTTCAGGAGTTTCGgAAAAGaTCGACAAaGTTTTAC

ATCGTCGTGgATTGTtCAGGCACGTtCTAGCCttGTTTtgTTCCaGTttCAcTCGAGACA

AGTGGAAGGCcGAtCCGTCTCtGAAACATAGATTtGTCTGGtGCcTtACTaaGGTGAaGT

ACCCTAAGCTTGgCGAATTTGTCTcACAGGTAGTCCCTTTCCTtCTCCTTCTGATTGaGg

ACTATGAaGTTGATAAtAAAGTGCttGgCaTaaGTGCTGTTCAGTACCTGATAGGAAATG

tGAATCCTGCAGATTTGCGtCTTtACAATCATaGTGAaGTTTtGTTCGAaGTACTGTATC

AGCAACttctATCCTGTAAaGATCCTGCaCTCTAcGTCCTCCTGCCCTGCCTCGTTCAGG

TCTTGAGGGTCATTGACAGCAATTCTCACACGATGAAGTCTGtGgAGTTCagtAGGTGGG

ACAAAGCCTTTGAAAAGGTTTTTCAAAGTATGGAATACGAAAGCAAGTTGACCACGAGAa

GGATATACTCAAGCATGATtCCcTGCGTTCATTGATGCcATGGGTGCAGCTGTGTtGAAG

CACACAGGCCGCACTTtAAAGGTtGCGTACAGTTTCTTAGAAATTtCTGACGgAGAGGAT

GAAGAAGCGAgactttattctttccaagttctcttggcagtgattcgcaacgcctggccg

gttatgtcgaagtataaggaggatattctcaaagtccg

>contig00194 length=225 numreads=3

AaGCAGTGgtAtCAACGCAGAGTACGCGGggaCGGATAAGTCTCTTGTTtATTATCTTTT

GAGagAcTAAGAGCGTTTCTCGCaGCAAGCccTGCAACacTGGTCATGGCATTGCGaCAT

GACGTACcTAcGGTGgTTGGCGTAtGATGCtGTCCGAATaCCGAAACAAAATGATAGTTC

GCgcttcggccgttgttacgcaccagctgcttcagcgatagcggt

>contig00195 length=629 numreads=8

tttagctggtcttgcctctgtatcttaagcaatgaTATTGAATGTGCAGTATAGGTCCGT

GGAGGCAGGAGTCATGGATTTGGATGACTTTAAAGCAAGTGACTCTGATTTACTTGAAGT

GGctATTGAGTCCAATGAGTTCGAAGATGAAGATATGGAAGATGTTTCCACGACTGACGG

TgCAgCCATTGTGaGCAGCTCATGCGCTGgTtCAAGACAggattgaccggcctagcgaag

ctccgaaaacgcggctgcaaagtgagggaaggcaggatcatgaaaggaataacttttgtc

tgaaggaaaaaCCTaCaCaGtATAAtGACaGGGAAGgTTTTAATAAGAAgCgCTTATtCA

AGACTcTTCACgTTCCTAAAGaTTCGAGTCCTGATTTTCTCGGCAGGGAACTTGCGTACA

GATTACaTGAGGTGAAgAAGGAGCTGTTTGTGCATGTGGTGCGAGtGTtGGgCGTtGAGA

AAGCCATCGATGTGTTCGAGGAGACGAAGaGTGTCTTGCGAAGTGGCGgCTTGCTAACGG

ACGACGCCAAGAGAaGGCGCACGCCAGGGGGTAcgTTCTTGTATctCATGAAAgCccgTG

gCTAtGCTgaCGAGAagcAAGTgAaGGAg

>contig00196 length=146 numreads=1

ttttgtttcctattaggtataaaggtgatatctaatcaatcagcttcatagcagaagcac

tttctgtattagcagtagggtttttccaacggctttcttaacctttttatagcagttagc

gacaaaaaaattctaagaagtcaggt

>contig00197 length=168 numreads=1

ttatgcgtaagagtgattttcactgaaatgggtacgattgatagtaaagacgttaggcag

gctcatgctccacaagaaaattcaggagatgtggacgagcaagctgctgatgagcaagga

caacatgaaatcattgctggtttaccgaaatggatgtggtggaggaag

>contig00198 length=456 numreads=10

AAGCAGTGGTATCAACGCAGAGTACTTTTTCGGGGtATTGCAAGTTCTGGCGACAACACT

CTTTGGTAGCGTCAAGGGACTTAATGCAATTTGACTGACACTTGCGTACGCCTATCGAAG

CTGGTAATtCATAATtGTGGAGTAAAaTGAgTTGGAAATAaGGTTGCAAGTATTACAATT

GTTTTACCAGCTATGgAGGATTTGCTGATGGAtgAGCACTCaGAAGGTgacATaTGtcGt

GTGTGTaGaatgggtgctagtgcagagaagcctttgCACTATCCTTGTGTATGCACTGGA

AGTATCAaGTATATtcACCAGGACtGTcTCGTTCAATGgTTAAAGCaCaGTAAAAAGGAg

cACTGtGAaCTGTGTTCAcACAAGTTTGtATTCAaGCcAGTCTACTcTCCGGATaTGCCG

TCGAcTATTCcTTtCGTGgAGCtGCTCCAAGGTTTA

>contig00199 length=156 numreads=1

ttgtaagactttcagagtgaatttgtcactttcttgtaaactgcaatgaagatagctgtt

gagcaagatggataagcagcaatctagtttaagtgctatcgatgctagtgatgatgaaat

tgaagtaactgcaagtgaggtcttagaaaagtctta

>contig00200 length=193 numreads=1

tgatttatggaggatcagcctcggttaaggtttacacttgcgaagaacgatagcggtcag

gatgacagcatggtcaatcccgaaggaatttagaagaaaacagtactccacttaaaagta

tccttaaaggcctagtgtggcctcaggaattgaccagttggaagaatgttcttatcgcag

ctcgtgcctggtc

>contig00201 length=798 numreads=23

CTGGACGgCCTGAATAGTTATATTGGAAGTGCGATACTCATACTCTAGGGCCCTTGAAAA

CTTCTCCACAAAAGCCTTAGATGCGCTGTAGTGAGACATCAATGGAGTAGGCTTAATTGA

GGAACCAGAAGAAACATTTATGATAGCCCCTTtACcTCTAGACACCATGCCAGGTAGAAC

AATACGACTCATCAAGACTGTGGCTTTAATATTTAGGTCAAGAATGGCTAaGATGCGATC

CTGAGGAACTGAAaGAAAATACTGAAGCTGATCATACATAACTCCAACGTTGTtCaCTAA

aaCTCCAaTGTCCAGAGCaCTtAAGGACCTCTCAAGAGTTTGCATGCAaTCTGCAAGTCC

CAAACACTGCAAGTCCATCGCCAAATATTCTGCAGATACCCcTTTTCTTCTTATCTGGTT

AGCTAAAATTTTCAGCTTTTCTTCATCTCTGCTTACCAAGAAAaCaTTCATCTTTCTATC

AGCCAGCTTTAGCGCATAaGATCTACCAATTCCTTCAGAACAACCaGTCACAACTGCCCA

CGACCCATAGTGCCTGAAGTCACTAGTGTACCAAAcTTTCGGAaCAAAaTaGATCcGAAT

GACTCTGAATAGCCTCCATACAAATGATACCATGCACTtACTGGTATAAAGAAGGCCAAT

AGCAGCCAATAGATCTCGAAAACTTGAAGCAAGATtCCAAATTtCCTGCAATAGCTGCAT

TTGAATGAGTCCGTCAACTTCAGTCATCAATGTATTAACGCCTTGTCACAGTTTTTATGA

AACCTGAAcTTAgTCTct

>contig00202 length=131 numreads=1

aagcagtggtatcaacgcagagtacgcggggacggataagtctctcttgtacacatctgt

tgagagcttaagtttaacgacgtagtgtgcaaatttctggcatcatctgactgttaatct

ttgtccattaa

>contig00203 length=591 numreads=10

tGCTCTCATTTACTGAAaCTTCGTCCTcAACAGAGCAGCACTtGTAAATcAgCCGGCTTG

ATAGAGAATGAtCCTTGCTGGCATGCAATCTAgCAGCTtCCaCATTTtCTTtGACAGGgT

CAATGCCAGTCACGGATGCCCCAAGCcGGgCTAATGGCTCAGACAAAATTCCTCCACCAC

TTCCAACATCTAGGACTGAAAATCCCATAATAGGTACaTTGACTTTgTGTGCTCTGGAAA

TtGgTTggTTACTTTtGAGGAGAATGTCCTTgAttAacGGAATGcGCAGcTtaTTCAaCG

ACGCcAaTAAAgCCAaGGcaCCCCCaGGCATCCACCAAtcctcagacagctggcgaaagt

tacgaacctccaaatcgtcaagagtacgagtactgctttgactatcaTGGGACTGgCttG

AAgCAccTGAAGTCCATATCAATTTtgATGATCTTATTATcAtGAAAcATCTGCCAaGTA

TtGttGATCCGtgTTTAAaaGTACAAGCCTTAaCAGTtGACTTGAATACTtAAGTGCATT

GTCAAAAGTATTTCATAACAGTTTATCTGAAAGTGAGGGCTGGTATCttag

>contig00204 length=162 numreads=1

gtttggtgctttctagctgatagaagtattttgttgagctaatgatacctatttgctatg

gaccctgtggatgaagattcttctgcattgcacaatcttttatctccttctggaggtgtc

cagcaggtcttgatcgaagatcctgacttcttgtcatcatca

>contig00205 length=193 numreads=2

GTAGAAAGCTTGTTGAAAATGTGAGGTGGATGGGACAAAAGGTTTTTGTCACAGTTGGGA

CGACCAAATTTGATAAGCTGGTTGAAACTATTTCAAGTTCTGAGCTGGTACTGACTTTAT

CGAAGCTGGGATATGAAGCTGTAGCAGTTCAGgTTagggcaaggatctaggaaaagtcag

atgtgggatattg

>contig00206 length=162 numreads=1

ccttagtgatatgatcctggtttgatgttactggtggatgcttgcaaaaagctggtgatt

tgcatagtccaatgcctggccattctgagttgcttcttctggtagcacttattgtagctg

tgagttcattgtctccactactattagagcttcattgtgtag

>contig00207 length=236 numreads=3

gggATCCCTCAGATCGTTGTAGAACGtGTTCTCGAtgAAGAAAAAaGCAGACGTGCATAT

CtCcTTGGCTGGCTtCGTACGCTCTGCAAGATCCcGGCATGAACTGAaCTCTCcAGCcAC

cACATTATCCGCATTACATTTGATCTTGTCGCGCAACTCCGTCAAATATTGCGAAGGAAT

AACAttgaattcgaccatatgccttcgcagcgtggggtggaaaatcgctacagtga

>contig00208 length=346 numreads=6

atgatgatgaccttccttcggagcaatctcaagctcatgaaggtcctggctttgtaggaa

ttcGCTTTTGTCAaGAaTGCAACAAcATGcTTTAtCCAAAAGAGgACAaGGaGCGAAaaT

TGCTTATGTACTCGTGCAGAAACTGTGATTATCAGGAGGAGGCTACGAaTACGTGCATCT

ATGTGAACATGGTGGTgCACGATATCAATGAAATGAcGCAGATtATtGCAGATGTGGTGG

CAGATCCTACTTTACCAAGGAATCACGAGAAGACATGTGCCAAGTGTGgTCaCAACGAAG

CGGTATtCTTtCAATCACaGTctAGCAAGGCcgATCATATGCGTCT

>contig00209 length=182 numreads=1

acttcgtgcttcgacagttatttcttggtaatccctactgggaacggtgggctatttccc

gagtagccgtgttcagatcaagggaaagagagcgtgggacagcttccgcaattaggaatc

ggatgtagaagtcgtcgcgtttctcgcggaaaaacgcaatcactggtgaacttgatagat

aa

>contig00210 length=483 numreads=6

AGCAAAGCCTAGAACCTGGAATGCGAGCAGAAGCATGTGTCCCCATGGTTGGCCATGTCA

GCAAATAAAATTCCGCGCAAGGTGACAATTGACTCAGGTCGATGTGTATCAAcTGGGGCA

AaGCAACcAGCTtCtGgaCctCcaCAaCGACgcTTAAGtGTCTTCCATAggcttggaagc

gtcaatgacaacggaagtggggttgttgattcggtgacagcgtccgttaaggaaagaaag

gcatttgaaagcaattattctgacgaagaagatacgattgaagttatggagaagagaaaa

ggtatttctttcccgctaagaaagaagaaCCCGaaCcTTGAAGAATCtGAaGAAAGTTTT

TGCAGCGCCTAAGAGGAAGAAAGGTGCTGCGCAAGTTCTTGAAGCGGAGTCTCTTGACGA

AGAGGACGAGCTGGCGGCGAAGAGCCGTAAGAAaGAAAAAGGATAAGCGAAAGGATGGCG

CCt

>contig00211 length=177 numreads=3

GTAGATGCTTTCTTGAGTACAGCTTTACCAGGACTAACGATGTCGACGCTGTCCTTTTCC

TCTACGGGCTCATTTCtACGGATtCtCAATTGATGCGAAATCGGTGTACCGcTCGAaTgT

TGaTaGAGAATAagCCTCctGtAGAaGCTTATtCCCGCCAGTGCCAGTATTCAAGCg

>contig00212 length=218 numreads=13

gAGAAATATCAACGCTGGTTCGTTGACGAttGTGAACGACGTAAATAAAGTTTTGTCGGA

TCGCTGGTCGATACCGAATTTTACTGCTGGTCAGTTAAAAATAaTTtGCAGACCAAAGTT

AAACTGGgAAgAAATCATCATCTCAGAAAAaTGgAAGCATTACAGCTGGATaGTCTTCTT

CATGCCGCTGCTGTtGagCTAGTTTTTatgcggcgagg

>contig00213 length=485 numreads=141

GTGAGATCTCTCCCTTGAGCAAGGAGAGCGCATCATTGACCGACTTATTCTACTCCTAGA

AAGGTTTGAGTGAGAGCACATCTGTTGGGACCCGAAAGATGGTGAACTATGCTTGAGTAG

GGCGAAGCCAGAGGAAACTCTGGTGGAGGCTCGTAGCGATTCTGACGTGCAAATCGATCG

TCAAACTTGGGTATAGGGGCGAAAGACTAATCGAACCATCTAGTAGCTGGTTCCCTCCGA

AGTTTCCCTTAGGATAGCTGGAACTCGGATTGACAGTTTTATCAGGTAAAGCGAATGATT

AGAGGTCTTAGGGTTGAAACAACCTTAACCTATTCTCAAACTTTAAATTGGTAAGAAGCC

CGACTTGCTTAACTGAAGTAGGGCACAGAATGAGAGTTCTTAGTGGGCCATTTTTGGTAA

GCAGAACTGGCGATGCGGGATGAACCGAACGCTGAGTTACGGCGCCCAAATCGACGCTCA

TCAGA

>contig00214 length=166 numreads=1

cattggaatgactgaagaaatgcccgagcctgttccctctgtcggccaggagagtagcac

tgaggctccagagccttcctgtggttgtccagaaacgaagaaggctcgtgaccaatgtat

tattgaacatggagaagagtattgtggtgatctcatagagaaacac

>contig00215 length=171 numreads=1

atataaatataggaaattcgtattgtttgagtcattatggtagcagaacaacttagagca

tgtatttcaagtgatgatgtggtacctttgtggatgaaagttgctgatgatgcaactact

ttatggaaaccgattgcactgacctatgttgactatcctgaaggtgatttg

>contig00216 length=180 numreads=2

CAAACAAACTTCTTGGATCTTGAAATCTGCTTATGCACTCAGTTGTCGTGAAGTTTTGTT

GCTCCGGTGATCTGTAAAGCTTTCAAGGATATAGTGGAAATTTGCTTGACCGTGTTTCTT

CAAAATGCTCGAAGATACCATTTTAATCCTCTTCATTGCCATCGCCACAGCCCTATTCAg

>contig00217 length=147 numreads=1

ttgagcagcaccaagcagttgacagtgtcagcaaggtaattgatcttgccaacctgcttg

aagtttgtgaatttaaacagttttgggttgatttggaggcaaaacaaagaacatatcgaa

ggaattaaaggatttcgtgactccata

>contig00218 length=168 numreads=2

AAATAAACCTACCCGAATAACTTTAAAGGCTTCTACTTGCATTGATAACATCTTTGTAAA

AACAAACTTTCAAGATAAaCTACATGCTGGAATAATTGCTGTTCACATAAGCGACCACCT

TCCACAGTTTCTTAattGTAGAGTCCTTCTCGTTGCCAAGTATTCCaa

>contig00219 length=152 numreads=4

gAGAACAaCAGCGAagCCAATGTATCTACCGacGCTCCTACTCATCAtCTTGTTGATCtC

GGCgACTCAAGTCGTTGGTCTACCACgAAACCTCAAGGagACGTCTTCGTCTgTtcgcAG

CAGTGCCGCGGTAACTTCGATATTTgtctcaa

>contig00220 length=295 numreads=9

cttggtcagcggacatttttccatgcgcatgcgcagtggcatgtactgcccttgcatgCT

tCTTAACACCGcAAACTCTTGATCcTCCATTTtCTTTAGAAaCAATCGCTCAGATTTTtC

CAAAGGGTGCAAGTCAGTAgTATTTGaTATtGTTTTCGGAAaTCcTCTtCTAAGTGTGTC

GTGGACTCCATAaGAGTCTGTCTTTGCGGATTCCATAACaGCAGGTTTAATGCTACCCTC

CaTACTTTTTACTATTgTCCTTAGTCTCTCAACaGATcTTATAAAGaGACCTAAC

>contig00221 length=180 numreads=3

AAGCAGTGGTATCAACGCAGAGTACGTCGGGGTcTGAcTAAaGTTAAGaGCGTCTTCGTG

GcAtGGCTtCGCGTGgTAAAACGgagACTgAAAAGCTgCaGCAcAACCTCGAAGAGCAGC

TTGACAGGCTAGTCAATCAGCTGAGTGACCTTGAGgAaTGCAGGGAggacctggacaaag

>contig00222 length=1171 numreads=82

GGTGACTAATATCTGCCTCCACTTTGAACGCGCGGTTCAATCGGATTGGATATTGGGTGA

AGACAAAGGCTTGTCTGTTTCACTTGATAAGACACTTCTTTGCTTGGATCAGAgCATCAG

TTCATCCTAGGTGGGAGCCAGCAACGGAaGCAATACCATGAGtcAAGCAaGTTTACTCAG

AGGGCCAGTCAAGGCAaTTTACGTCAACGTACCTGTAGGAGTGCCGACCGCTGATGGAGG

CTTGGAGATCCCGAACAGGgTGTTCTTAGGCGGCATTCCATCAGAAACCACAGAGCTCGA

ACTAGAATTATTTTTtAGTGACTATGGTACAGTGAAAGACGTGAGAATAGTGACTGATCG

GGTGACTGGAGAATGCAAGGGATACGGCTTCGTTACGTTTGATGAAAATGAAGACATTTC

AAAACTTGTGACAAAGAAGTCGATATTGATGAAAGGGCGGAAGCTTCGTATTCGCAAAGC

AGTGCGAAGGAATGGATCCCAATTtCCACAGGCTGGCCCTGATCAATCTTCCTCGTGTTC

CTCGTCATTACCGTCGAGTGCCAATACTTTCTACTTGGTGCCGGTAGACCAAGTGCCACA

CTCACCAACAGTTCAGTACGGATACATGAGCTATATTCAACCaGTCCCAACGCACGTCAa

CACGCCACCGTCAGTGCATGGCTCTGTTGCCTTAGCAACCGCCACTGCAGTTCCACCAGC

GATCATACCGACAGCTTCAGTCATGCCTCTACTTCCTCCAAGCATGCCCATCTATCAGGC

TCACGGTTTGGAGACATTTTGTCAACAAGTTCAAGCATGTCACCTGACCTGCTAATGCTA

AGTGGACTGTGCAACTTCCTTCGAGCACGCATCTCAGTGCGAGCAAGTGCCGCTTATTGC

TCTGATCACTGGAAGGTGTAAAAGCAAGATGctcTTAAGCTACCACTTCACGAAGACAAG

TAGACCAAATTTtACAATGTTTTAGAGCCcGCCGCTTAAATTATTATATGGGGCTTACTG

CTTTGTTAAAAGGTTTAAGaTGTATTTCCTATCCTATTAATGTATATAGATATCCTTAAT

AGTTCGTAAGTTATTAAATGTAAGTTtCGTCAAATTTGATATCAAAGTTGTGgCTTGTAG

AGAATGCATGGTAGAAGATATGTAGTTAATG

>contig00223 length=615 numreads=9

atttgactggctacatcactgaaggtcagaTCTACGTCGATCGCCAACTTCATAACAGGC

AGATTTACccGCCGatCAACGTGCtGCCTTCGCTCTCCCGTcTCaTGAaGTCTGCCATtG

GCGAGGGTATGACTAGAGAGGACCATGCTGATGTttCGAATCAACTCTATGCCAATtACG

CCATCGGCAAGGACGTGCAGGCCAtGAAAGCTGTCGTAGgTGAAGaGGCCCTCACGCCGG

ACGATCtGCTGTATCTGGAGTTCCTTCAGAAGTTTGAGAAAAACTTCATCGCGCAAGGAC

CGTACGAGAACCGTACTGTCTTCGAGTCGCTCGACATCGGATGGTcgCTGCtGCGTAtCT

TCCCTAaGgaGAtGTTAAaGCGAATtCCTCAgTCGATTCtCGCGtCTTACTATCCCCGag

ACGGGAaGAAGGGGCAATCGGAAAACGCTACATAAGCCTATTATTCGTGGCGTGCGTTCT

TATTGAATTATGGGTGGGCTACgtgggaactcttgcattgtagacgccttacggcgacta

atctatttctcaGCATTTTTtgCCTACTCAGTCGGGTAATCTTGAGTTGTATTGATATCA

ACCGACGCAAGttta

>contig00224 length=181 numreads=1

tttgagttcgtatctgttcggtcgaccggatgatggacgcgatgagttgtttttcacggc

acaatctgtgcgagaaatcaagaaggccggtattgaatgcatgactgggaggtggcgaga

gagagaagtgcgacagttgcacgaaaatgttatttggtactgtaatttcttcggtgtgtc

c

>contig00225 length=196 numreads=7

AAGCAGtGGTaTCAACGCaGAGTAGTttCCAGGACCaGCAGAGTAGTTTCCAGGACCAGC

AGAGtAGTTTCCAAGCCcAAAaCGAATGGCCGTACTAGCAGAGGAATATGAACTGCTtGT

ATCGGCGTGATTGATAGGTGCGACCCCTTTCTGTTTCTTGAATGAAACCTGCAGAACTTT

CCCGtCATACATATAC

>contig00226 length=599 numreads=15

TGATGAAAGTGCAAGTCTTcAGTCGTTTTGAGGAAAaCcTGGAAAaGtCCTTGCcACTTT

TGCAGACaTTAaTCcTtACGAATAACaGTGTCCAGGAACTGATAGATATtGATCCTCTTG

CTACTGTAAaGaCTTtGgAgCATCTCAGCTTACTACGTAATCCAGTGGCCAATAAGCCTA

ATTATCGcTACTATGTGATATACAAGCTGCCTCAAGTTCGTGTTCTGGATTTTCAGAAGA

TACGACAGAAGgAGCGCATTGCTGCAAaGCGCGTCTTCAGTGGTAAACAAGGGGAaCTTC

TTCAGAAAGAAaTTGCTGCAAAGAGAACAAGAACATTtGAAGTTAAaGACATCGCTGAGG

CAAGAGAGGAGGATGAAAaGCTCCTtGCAGAAAGGTATAAAGATCAAGAAGCCATTAAGG

AAGCCATTGCCAATGCGTCAaCGTTAGAaGAGgTGAGGAaGTTGgAGCtCCTTCTGCAAA

AGGGGCaTATtCcAGGTAGGGCTGAACCGTCTGAAGAAGCTGAGGCTGCCAACGGAGACG

AAGTTGATTTGGATACTTAAATTACGTTGCGCACGCGATGGGTTTCAGTGCTGAGtttt

>contig00227 length=584 numreads=13

GCACCGCACGTCAAAGCATGAAGCAAGAAACCATCTGATCCTACAACCACAGCTCGCGAG

CGgTTCtCGATTCCCTTAATgAtCAACTCCGCCACTGTCTCCGCAGGCAACACACCAACA

TCATCTGAGATCTGCTTTGTCAAATGTGGCTTCCTCTtGTTTtCCGCGCTGAaTCCAGgA

GTGTCGGTGTCAGCAGGATAAACCATTGTGATTCcTATATCGTTCACAGTTAACTCGTGC

ATTAGTGCTTCAGCCAGTCCACGCAGAGCGAATTTGGAAGCAGAGTAGGCGCTGTATCCA

TAGATAGACATTTGGCCTGCCAAAGAGGACAGAAATACAACTTGgCCAAAGTTTCTTTTC

TTCATTCTGGgAACAACGGCGTGAGTGATATAGACGGCACCGAGGTAATTCGTAGCCATC

ATGTTCTCAAAGTCTTCTGATGATAACTCGCAGAACTCTCCAGGGATCGAGTAACCAGCA

CAGTTTATCAGAaCaTCTATACATCcAAATGAtGCTTCAATTTTGTTTACTTGCTCTCTT

ATtAGACTTGCTTCACCAGTTAAATCAATACACATCCATTCGAT

>contig00228 length=177 numreads=3

GGTCGAgAgAGAGGTcTTTgCAGATGGCTtCTAGTtCAGCAGCAGATAGCAAGAAGaGAA

AAAGAGTGGTGCCAAacGaTGATGAAGATATgCCGGggAaGGAACCTAaGCTTTATaGAG

gCATtGTCTtGACTAGAaCGGATCCTGCTGAGTTCTATGACTCTGAACGTCCAAGCg

>contig00229 length=164 numreads=1

taaccaggcacaagaatattcattaacagctgcatcatgtcgtcaaggctgattaaatcg

acctcctcaaacagtcgcatttatgtgggaaatctaccccagcatgttagaaatcgagac

attgaggacatcttcaacaagtatgggaacattgctgctattga

>contig00230 length=570 numreads=17

GGGAAGAaGATGACGCgACCAGATGAAAGTGCATcGCAGACTGgCGCCAAAGTTCTAAaT

CCTGgCgCCATaGaGGAACTGTCAaTTTTACAAAAAGCCTAtAAAataaTCaCgCATGAC

CAGATGAAgTACATGGTAGCTGGTGgAATAGCTGGTGGGATTtCgaGAaCTGCTGTGTCT

CCCCTTGAGAGAGTCAAAaTtCTtCTGCAGTTGCAAACTGGATCTACTCAAGACATGAAG

TACAGAGGGTCTTGGGGTACTCTTGTAACCATATGGAAaGAAGAGGGCTGGATAGGGTAT

TTTAAGGGCAATGGTACCAATGTAGTGAGGATAGTTaCCCTACTCTGCAATCCAGTTtGc

AaGCTACGAAGAGTTCAAAAGgCTCTTCAAAATTCCTACTGaaCCCAGACAACAGACGCC

ATtGAAGCGgCTAACGGCcGGtGCTTGtGCtGgAATGGTGgCGGCCaCCGgCACCtACCC

TCTTGaTTtGATACGGACTCGTCTTGcTGCACAAGGTGAAGGTGCCcTAAAGAAATACCG

GAACATAACGCACTGCTTCTTTCTCGTAAt

>contig00231 length=151 numreads=1

ccagatatcgatcctgtagcagtcatggagtcagttacggagtcagtcaccgcactcttg

cctcacttctcactgcgtagtctgctatcgacgtacttgaatcacgagaccgagaccctt

cgcggtctagcagctagttatggtggtgact

>contig00232 length=558 numreads=29

aTTGCTGTGTTaTCCCCATTACTGCAAGaTtcAAGATTTtGAtGAAAAcTTTTACAGAGG

TTTCAataTtGTtGTCtgCggtCTTGATTCGATTGTTGCTCGAAGATGGATCAATGGAAT

GCTTCTGAGtCTACtGGAATATGATGAAGATGGCAACATTGATCCaGCAAGCGTTATCCC

CCTTGTTGATGGTGGTACAGAAGGTTTTAAGGGCAATGCTCGAGTAGTTATCCCTGGGCA

GACAGCATGCATCGAATGCAACCTGGATCTATTCCCTCCGCAGGTCAATTTTCCCGTGTG

CACGATCGCTGAAACTCCGAgaTTGCCTGAACATTGCATAGAATATGTGAAaTTGCTTGT

TtGGgATAAAGAATGGCCATTTGGAGTCAATGTTCCGATTGATGGTGACAGTCCAGACCA

CATCCAGTGGGTATGCACAAGGGCCTCTGCAAGGGCTGACTTGTACGGAATCCGAGGCGT

GAACTACAGATTGACGCAGGGTGTGCTGAAGCATATAATCCcTGCCGTGGCCTCGACCAA

CGCCATTATCGCAGgTAT

>contig00233 length=181 numreads=1

tagggaatggatcaaattcagcaagcttttgagccatgcaaagagtttgctaaagattcc

atccgtcttgttaagaggtgtacaaagcctgaccgaaaagagttctcaaagatcgccatg

gctacagctattggattttgcataatgggtttcattggtttcttcgtgaagttgatccac

a

>contig00234 length=544 numreads=28

GGCCAAAAaGAaGAAAGAGAaGAagATGTCCAGTGACAGTGacGAAGATgTGAaCTCAAA

GAGTGGCcGACGAAAGCGATTGAAGCATGGGGAGTCCCTCGTTGCAGACGGtGATTCGTT

GGATTCATCGAGAAAAAGACGGAGTCAGTCTGACCATGAAGAGGGTGGTGTTAGCCAAGG

AAGCAGAGGGGATGACAAGGCAGAcAGGAAAGATGTCAAGCGCAAAAaTGAATTtCCAAC

AAAACCCGAGGGTCGGAGAGGTGAGGAACGAGAACCTAGTAAAAGCGACGAAAAAATGGA

CGACAATAGAAAGCGTGACAAAGTTTtGGAGGGAGATTACGAAGAGGAGACTAAGAACAA

CAAGCGAATGGAGGACGGCAAGCAAAaGGAGGGCAGCAAGCCACGGGAAGACAATAAGCG

AAAGGAAGACGACAAACGAAACGAAAGCGACAGGCGAAAAGACGACGGAAAaCGAAAAGA

AAACGaGAaGCGAAAAGACGATGACAAGCGaaaagaaaaCGAGAAgacgaaaagacgacg

agaa

>contig00235 length=167 numreads=12

CCTGAAGCTGGCAATCATTACTCACCTGGACTGCGCAATaTTGCTCTGCCACAATAAAGA

AGTaGATAACTTTGTCCTGGGGAGTGGTTTTGTCTGTGACCAATGTTGgTTTCATGTACC

TGAGCCGTATTTCAGAGAATCTAGAAGCACATGCCTTGCTGGAAGCg

>contig00236 length=902 numreads=8

aagcagtggtatcaacgcagagtacgcgggggctgcagaaaggttcgatggagacgtgta

tcatgagcaaggctcggacgtggtatgaaccaccgaatgaatgggCTTGTTGACTGAAGC

GCcAaTTGCATTcAAaGATGGCGGTAGTTGTTGCGGCTCTTGCTGCGAGCGAAGaaCGCG

CTCTCGCCGtaCtAcgCGAGTCtGGCAGcGTtGGCgAAagTGTTCGgtCcATGCCGAGCA

ATGATCTAACTGCTTGCCATGTCGAAGatgtcgatgctgacaaccacacggctcagtgtg

gagcttcagatcagggtgtgagcttacgagaccagtccattcctgcGAAGTGCAGTTGGG

ATTCGCcAGACGATGCACTCGAGGGAGCTGAAAaaCGTCGAaGCGTGGATTGCACGCGGG

ATGAGTTGCTACCCGCAGCACCCGCGCCTGGAAACGGCTATTCGAAGCCGTCAAGTTCGA

TAGCTAGATCCAATAATTTTCACCGATTgCcGTCCCAGTtCGGAGACgAAcTtAaGAGGT

ATTCTGTtATttCGGtAtCAGGTAGCcGAATCAGTACGGCGGTGAAGGAATTACcGAGTt

CCTCGTCTAGAaGCTTAGAAAGAACCTATcgactctccaagctgtcaTGTCCCGAGCtgT

TGTGCAAAATTTGtCACAACGATAAcACgATCGAGgATTtCATCAGCCCGTGTTATTGCA

CAGGgaCAatGGgACaCGTTCATGAATCTtgTttGGTTCAGTGGTTGAaGAGTtCATCTC

GACAGTCGTGTGAGTTGTGTAACGCAAGGATTAAGATTGGTACTCGACTGAAGCCGATCG

GGAAGTGGACTTGGCCTTCAAGGCGACCCACATCTTTCGTCTGGGTcTAATATTTGTTGC

gg

>contig00237 length=1012 numreads=22

AgttCagAaTAaTTTATACATTtCGatACcTACAGAAATtCCGATTACCAGCGGAAAaCC

TTCACtATTTACACTAaCgTgCcTAAACGgAGATtAAATATtATAtAACCCAAGAGATAC

TACTAAaCCGTCCACATTAAAGAaGTtGGAaGTTGGGGAAGAAACAAAGTCCTATTTTGC

GTtGTCtACTtGaaCTtCcGAGAaCCgAAaTATGCCAACcGATAGCCAAAGTCCcGGCTT

CCcAcTTTTAtcGGTTCATGATCAGACTGTCTCAAGATCGGGGTCCgTAGCAAAAAACGC

GGTCCGGAGTAGCAGAGTTGTATTTCTTCCAATGATCAAGGTCGTTGTAATaCCAAGCGC

CCGTGCAATTCcGATCAACGGCAAACATGgTGGGgCCATCTACCTCCTTCATGTTCAAAA

TAACGTCGGCTGTAGCTCGCAGGTCGCCGTAAGCACAGCGGTCAAtGTTCAAGAAGTATC

GGTAgCCAtcGCCTTCTAtAGGATCGATGTAGGCTAaGCTGAGCTTTTtCAAGAGACGGT

GAAaTTCGAATtCAaTGCCGTTTACtCTCAGCAGTCcAGCGGCAGGGTCTCTCTCTCCGA

TGAAATAAAACACGACTGATTGACCGGGTACgCtCCcGCGCTTGCAAaTCATGTAGCGCA

AAGCTGAGCCcGGaCTGaaCAAcTGCCACAGTaGGATGGCCAACGCGAGAAGAAATATAA

CTCCCACGGCACAGCAaGTATTAGTATGTAAtCTTTGCTTTCTCCATTTcTTCCCCATAT

CCGTCAGTCTTGATTCGAGAATGGCATGCCCAGCACTGCTGAGAATCGATACTGCCGCTC

TTACAAGACCAACcTCCTTTGACTGTTTCATCTCAAAGGCATAGCCGATTAAAGCCGACT

GCTGCTCGCGAcGGAAGAcGTTATACAGATcGTGTTCTGGAaTaGCAaCgCCGGgCTTgT

CGGgagAGGCGTAcAAAgCCTcGCGCGgCTCaCaCgTGCCAGCTATTCTacg

>contig00238 length=1062 numreads=23

AAGCAGTGGTATCAACGCAGAGTCGCGGGGggTGAAGGTCTAGAGGTGTAGTTAGCCGGC

TGATTCGAAATTCAAAGATGATGAAAACTGTTttCTGTTCcATGCTTAtGTGTCTGATCT

CCtGCGCCAGTGTTAtGCTAGTTACAGCTgACGTatGTGgCACCGGCGtGTTCAGTGCTG

CGTGCAACGAgCTTCTTGCCATACAGACGAGAATCGACGAAAGCAAGAAaCTACTAGTcG

ACCAGATCGACGAATTTCAGAAATTTCGCTCTACAGAATCGAGCGCACGAATTGAAgATa

TCTCCAAGCTGAACACAACACTCGTAAATTtGCTCGAGGGAGAGTTGGCAACAGTGAACG

AAGAGCTTTTGAAGCATCTGAGAAATCAATCgCTTGCACTATCCGCAAGACTCGACTTAC

TGAaTAAGCAATTTACGAAGgAGCTGTCTGCAGCTCTTGACATTTCTCGCAAaGACAAcG

CTGACTTACGGGCGGAGGTATTGACGgAACTAGCTGATGAAAATACGATTATCGAGAGAA

AaGTCTCTAATCTGCAATCCTCCATCGACGTTACTAAAAAAGAGTTCGAAGCTCAAGTCG

TTAAGCAAGGGgAGAAATTTCAAAGCAGCATTTCACAGAaCTATAACAaCCTTCGACAGG

AGACCATACAATCTTCGAcATCTTTAAAAGCAGACCTGCAGAAGGAAaTCCTTGAAACTA

AGAACCTtCTTAAAGATCTGCAAAACAAATACGACCaGGTGAAAAaCAGGGCGTGGCCAG

ATGGAAGCTACTgcATATTCGCAAGTGGACCTTGCCCGTCTGGCTTCAGACTGATCGAGG

CgcaCttgaGaGCaCtATCgACATATgCcGCAGACGgtaGGTACaTTCgaCcGgACACGT

TCGGcAGCAGTAGTATCAGgtGtCatGGAAACTGCGGCCAATGGCATCCTTGGAATGGGG

AATTAAACTTAGCTGTCTGCTGTAAATGATTTGATATTGGATACAGGTACCTAAATTAGC

TTTTGTCAGCTTAAATTTAGGCGCGTATAAGAATATGAAGTa

>contig00239 length=1010 numreads=49

TTGACATATGTGAaGgtCcTTATCCAAGTtGgaCATGAGCCACTGCCGCCAAaGTTAaCT

ACCACTTGGTTTGGCAAAAAaGTTTATCGACTGCCTAATTTTTTTCAGTATGCTGGgCAT

ATCAAGAaCGTTGATGGTtGGACTGGTTTATACAGAGGACTTGGACCAAGAGTTATGCAT

AATGTGGTCAATACAGTAGTTTCGAATACAGTGTCTAAGGTGCTGTGCCcAGATGAGGAG

GGAGACGCTAAAACGAAGAAAGGTCTTCAGAAGCAGACCGTTGGCGAGTTCTCCAAGAAG

ACTGGTGGTGCCGCAGTTGCTGTTGTGGCTGGTTACGTCATTAGTTACCCGTTTCACGTA

aTCACCGTGAGGATGATGGTACAGTTTATTGGCAGAGAGACTCACTATTCGTCAaTATGG

TCTTCTGTTCGAGAAATCTACGATCAGGAAGGAATTCTCGGATTCTTCAGTGGAATCGTT

CCTCATGTGCTtGGCGAGTTGGCCTGCCTGGTGATATTTCGTTCGCTCGCTTACCTTATA

AATAACTTCGTCGTCGACGAACAGCTTGGACTCGTTtCTGATGTTAGTCAATACACCCAA

GGGATTTCGCAGTATATCGCATCGATGTTGACGTACCcGTTTTtCTTGGTTTCAAATATG

ATGGCAGTGAACAGTGCAAGACTTGCTGGTGGCAATCCGCCcTTGATGCCTTTGtACAAG

GACTGGAGGgATTGCTGGGCTGATCTGGGACGCAAgggCTTGCGCAACaGGGGcgCTGCc

GTGTTTCGAAGAACCTTGCTAGCACTTCCCAGCGTGTGAAGCATGGAAACGAaaCGAGGC

AAGACTtTcATGGCGCTAcAAAGATTTCTGATtGGCGCCATGTTTTACGGAAgTTttGTA

GGCTCCAGTAGAAGCAAaGCAAGAGGTTGTTTTTTTtaTCTCGcATGGTTTTGCTTTTtA

TTGAACGTCCTATGAACAAGAAaTGATGAATCCATGTaaaaaaaaaaaaa

>contig00240 length=680 numreads=7

CAaCGtATTtaTGTCAGGCATCaCACCGCAGCGAtCCAAGTTTCGACCAAGcGACTtCcA

GAaCTTTTTTgaCGTCACAATGAcGTACAAGgAGGgTAGTCACATTcGcATACCAtATTG

GCCGAATGaCGgAAAGTtGCCAGCCATGTATCGCaCAAAGCCGAaGGGTCGTCATTTAaC

cGAGgAtGAgCGAATGGATATTGAAGAGgATTTGCACGgTtACTTCCTgaggcatggaaa

ggaagatttcatgggatatacaataaaaaattgcTCCGATAAcgTGTTCAGCAACCTATT

TGTGCGcAAACtGCGAGAAGAAGGTTTGATTgTtACaTACGGTGTTGACtCGAAtGAGAA

ATgCTTGAATATCATTAAGGATACAGTACCACTGCCGTGCCGATCTGGCTGGTACTCAGA

CGATTGCGTTAAAACATTCGAGCAATTcAAATTCTATATAgTTCcGGaGGATGACTTATG

CGAAGACTACGTCGGGaaaaTTACTGGTATGCGATTCTTGCATGGAACGCCGTTCCAATC

GTGTACGGcgcagcagaccctgcgaaatttctcattccgggatcgtacatcaataccctg

gaagaagagtacatatcgccgacgttcaagaaagcttactcagttagcaacgacgctcta

gcttattataaggcgtttca

>contig00241 length=237 numreads=2

CTGAAGCAGGAAGGTAAGGGTTGACTTCGGCATGTTTCGCACTGGAGTACGAATCCTGTC

CATCGTCATcgCCGTGTACGTACGTATGTACGTCTATGCAAGACAGAAAATACCTCAACA

TAAATTTTtAGTATATATATAAATTTTAGTACATATATAAATTACTCTTATAAAATATAC

CCTTATGTATATATGTACATAACATGAACTACAGaccagccgtttacttgttccaaa

>contig00242 length=777 numreads=23

AAGGTtCTCTCTGATGCAACTCGTGTAATGATTACGTTGTTGCAAAGAGCTCAACcGATA

TTAaTCTGCAAATTTCGGTCGAGCGAATGTGCGAGCAGACGCACCCTTTACTGGGGAAAG

ACCGATTAGTAGCAGAGCGTCAACCACcTGTGCCGATGATATTGGAAAaCAAATAGATAT

TTCCTCTGTCTCGGCTGGTCGAAATTaGgTGACTTtGGCATAATCTTTACCCGTGGGTTT

GAGACCGATGGCTCTCGaCAaCCTCTCAACGACAGgTGGGAGTAAGTCTTGCGCGTCTCG

GTTATCGGGTAtAACTACCGATGCTGCCCCATCAAcGCACcTTAACCGTTGAGGTGGCAT

CCTGCTGGgTAAGTGTGTGGAGCCCATTACGCACATTGAGTTAGCGCGGGTGAAGCATAC

AGAGCTCCTGTCAGATTACCcTAACAGTAGAATGAGTGAATCCGACTCTGATGGATGAAT

GCGGTTGACGCTAGCATATGAGCCTAGAAATTGTCAAAGCTCGCTAGCTGGTATAaGGCA

CTCAAACCAACGCTTCTGCGTCTAATTGCTTGAGTTACGGGACTCTGGGCTGGGCACAGC

ACGGACCATGAttGgAATCTGTAATGGaagcTAGTTTATgCCcGCcAgctGtGCCTgTAA

GGGGATACTCCCGGCCgCCgAAACAtgAGggTTtGgTGCCTcTGTGacGCATTGgCCCGA

TAaGCAaGCAAatCGgAaGCGtCaTGTGCCTGTGAGaCTCTGCGTTGATACCACTGc

>contig00243 length=172 numreads=1

aagcagtggtatcaacgcagagtacgtcgggggataaggcaagagcttgtacaacgggct

tggacaggccatgtgttgaaatataataagcttggtaaaatcatggcctcggttggcctt

cgttcgcttctgaagaaatcaggccatattatcgtctgttcctggactccat

>contig00244 length=556 numreads=10

tGGAACATCAAGGACTTCAGTGAAAGAGAACACAAGTACACGCGtCAAATATTGAGTTcG

ACCATGCTACGGCTGTGTCATTCAGTCCAGATTCAAAGgCCTTCATAGTTTCCCTTGGCG

TGgAGCAGACCATTCGCGTCTTTAAGCTGAGCAaGAAAAAaGATGGAGTCAATTCATCGA

TGTTTGCTACTCCTGAATATGATTTTCCcAAAGTACACAAAACAGAAATTATTAGCGTCG

GAATATCGTCTTTGGGAAACTTCATAATGTCTGCTAGCAAAGATACCACGCTCGTTATTt

GGACGTTAAaGGGTGAAGTAcTAGAgAAAGTTGACACGCTggtcAtgTAcAATAGCCATG

CTgCCAtCTcGCCAtGTGgAAaCCTAGTTGCTGCATGCGgATTTACctCCgACgTgAAGa

TgtGGGAAGTGtCATTTAAAGGTGGCCAATTCAGCAAGGTCTCGAGAGCTCTGGAGCTtA

AaGGGCCACACAGCGGGAGTTTACTCCTTTTCTTTTACCAcGGgATCGAAGAGAGTTGCT

ACAGTCTCTAAAGATg

>contig00245 length=280 numreads=4

AaGcAGTGGTATcAAcGCAgAGTACGCGGGGaCGgAAGGGgTTCAAaCTTTCAATCAAGC

tAaCTAGCGgTTCgCCTGCGAATTCGAAAAGTTAGGTCTCACATTCAAATGGAGGCGCGA

TTCTGAATTtGGaGgCTGAaTtaTGTTATGcAAAAAGCgTagTGTAGCGGAATGCCTTTA

CCAGCAGTATTGCAAGCTCGATTGAAaGAAAAgGGAATCATCTCAGAAGATATTgCTAAA

aGAAGTCCAaGAaGGaaaatgccgactcggacgtcattcg

>contig00246 length=882 numreads=61

CAACAGTGGCGGAGGTTGCTTCTCTGGAGTGGGATTCAGCACATCGGAAAATAAGATTTC

GCTAAGTGATGGATGCTGGGACAAatCGACAGTCATTCATGAAATGGGACACAGTCTAGG

TCTGCACCACGAACAAGCCCGTCCGGACCGAGACCGCTACGTAGAAATTGTTTGGAGCAA

TATTCCCGGTGGCCAGAATCACAATTTTGATATGGAGCCTGCAAGCAAGATAGACTCTCG

CGGAACGCCATATGATTATCGGTCAGTGATGCATTATGATAAGTCTGCCTTCGGCAATGG

ACGAGTGACGATTCGTACTAAAGATCCTTATTATCAAGATTTGATTGGCAACGGTGCTGG

CTTCAGCGCAACTGACGTCCGTCAATTGAATCTGATGTACGGCTGCCCACAGACGACGCA

TACTTTCCCCCCGACAATGACTCCCGACTGCCTAGACGGAATGAGAGTATGCTCcGAAAA

TGCCGCCTCAGGACTATGCAACGACagAaGTTGGAAAGACTTCATGCATCGCACCTGCCG

GTTCTCCTGCGGTCTATGCAAAGGTTCCTACACACCTCGCCCGTATACCCAGGGgCcAAC

TCCACCAGAACCAAAGACGCCAGGTCCAAGCGGCAAGTGCCGAGACGTGCACACAAACTg

CcAAAGTTTCATAaGCATGTGCCGAGATTTtGGCTGGGTTGACCACATGAAAAAGTATTG

TGCCGGAACTTGTAAGTATGATTGCTAACTAGCTGGAGCAAGTTTGGAGCGCGCCAAAGA

CATTCTGGCAAACTGCTGGATACTGTgTGTtAGAATGCTgTAACAGAACGGATTATGAaT

AGTTtAaTAGATGCAGCCTGCTCcGCTGAAGGGATACCACCA

>contig00247 length=1468 numreads=65

tttCGGGGTTACCAGCATGACGCTGCATGTCGTGTTATTGCTCGTCTGACCAAAGAAGCT

ACAGCTGCTCGTGAAGCTTTGGCTACGTTGAAGCcACAAGCTGGAATGGCCACCCTGCaT

TTCAGGCCGCCTCACAAGCAaCTGCTCcTGCTtCTGCATCAGCTCGTCCGGACGATATGG

ATGTGACACAGGAAGCGAAGGAAATTGGAATGTCTGAGGAAATTCTGCAAAAGCtgCAAG

ACAAAGCTACCGAACtcACGACTGAAAGGCGAAAGAGAGGCAAGAAAGCGCCCGAGGAGT

TGACTTCTGCCGATGATCTCAAACAGTTTCGCCAAATCGCGTCTCACCCGGGCTTGCACA

GTGCGAGTGTGCCTGGCATTCTGGCCCTGGACTTGCAAGAAGAAGACACGTCCAAGgCCA

TCACAGGTGGTGCTGACAAGAATGcGgTTGTCTTtAACAAGGAGAATGAACAGGTCATAG

CCACTTTGAAGGGTCACACGAAGAaGgtcACCTGCGTCATTTACCACCCCcGCGAGGAGA

TCGGCATCACCGCTTCTGCTGATTCTACCGTCCGTGTCTGGAaCATTCCAAACAGCTCTT

GCGCGTACGTTATCAAGGCTCACGATGGCGGCGTGACCGGTTTAAGTTtGCACGCAACTG

GTGACTACGTCCTGACGTCTTCTTCAGATCAACACTGGGCATTCTCCGACATCCAGaCtG

GgAAAATTTTGTGCAAGAATATCTCTGATCCACAGGCCAGCCACGGcTTGACTtGCgCAC

AaTTtCATCCTGACGGTCTCATCTTTGGCACGGGTACCACTGATAGCGTGATTAaGATCT

GGGACCTGAAGGAGCGTGCCAACGTGGCGAATTtCCCTGGGCATTCTGGGCCGATCACTG

CCATCTCGTTCTCTGAGAACGGGTATTACTTAGCAACATCTGCCGATGATTCTGTGgtCA

AACTCTGGGATTTGCGAAAGTTAAGAACTTCAAGACTATTGCTTTGGACGAAAGAGACGA

GATTAAGTCGCTTTGCTTCGACAAGAGTGgCAATTACTtAGCTGTGGCTGGgTCTAACAT

CCAATTGTATgTTGTCAAGCAGTGGAGCTtaCTAAAGACcATGACAGATCACTCcGgCCT

GGTGACGAGCGTCAAaTTtGGCAAAAACGCtGgTTTcttggcgtccgcaagtaTGGAtGT

ACCCtgAAatttttgCGCgTAAGCTAAATAGACTATGCTGCGTGGATACCCGGTACACGT

Tgtggcaggtttgaagtggaggacacgtggtgctgtaacacgagtcgaccgtgccgactc

gtgaacaatttcaacttgtgaagctacctcttggagaatgtatggtgtgaaagttttgtg

tagcgatacgaaaagagtagtcgcttaactctttattactaaaaaaaaaaaaaaaaaaaa

aagtaactctgcgttgataccactgctt

>contig00248 length=177 numreads=3

gACCGaaaCTAGcgTCTTCTGATTTGCAGCAAGAATCCTCGGTTATCATTTCtCCGCcAT

TAGAgAAGgATCGGATGTCCcTcATGCTGtCGAcgtCAACGTTTTCTTCAGTCaaTAAAA

ACCCtATCAGTGTCTtGAAtGACcTaGCTCAAaGACAAGgCACGgAcgtgtcgtttg

>contig00249 length=108 numreads=1

gccaagttggacaatggactaccgaaattaaaagcgtcttcagactcgttatcatgctgt

gacgattacggcttcacctctggagaagagattcatgtgccagccagg

>contig00250 length=489 numreads=6

ggactttcgatgatactggcactttcgatggccctgatgctttgctgttccatcatcatt

caggcacttatttcggcagttatgACTTCCtCGCGGTTAAaTataaTAaCCAAAcTTCAG

GCTCGGAGACAACTACATTcAAACGCTACTTCcTCGTGGTCAAATCAATTAGGAGAATCC

AACTGACCCCACCCCGTGGGGAACACTGGGAAAGGCGTAAAAAaTTATTTGACGAaGAGC

TCaaaTAgAAAaTGtGGgAAATGACGTACATCAAACTAACCAGCTTCcAAaGAAAACCGT

TCCTGcAAAAAAGgTAAAGCATTTCAAAgTAaGTtcAAATAacaTGATTTTTCTtCCAGC

TACAGATGTTATAAGATatAGCAGtATTtAGCTGtATTtAGCTGGCAGACATAGCCCACc

TcAGaCTTCCTCCTGATGCCAGACAAATCATCaaaaaTAACATCCTGCATACAGAACATA

AAAGAATAT

>contig00251 length=605 numreads=28

CTGGAAGCTCGAGTTGGGTTGTGGAAATCCAAAGAAATCAGGGGAAaATAATTCGAGAaC

TGGCGGAGATTAAGCTTCtGCTAAAAGaTTTAATTGGAACGCGAATCGCTCATCAACCGG

ACGACGGGCTGCAGCaGaTTGcTTCCTTAGAGCAGCTGGAgCATTTTAAGATGCGTTAGT

AGaTCCTGAaGTATTCAGTGACATGCTGCGCAGTTtAGCAACCGTTGGTGgTtCATCGCc

TAGAGaCCATGTTTTCCTAGTTTTGAAAAGATTATTCTCTatGgaTcTTATGaCGaCaTT

GAaCCGTAAGGgcAaGGGGGAaaGgttgggTtggAGCTGAAaGaTGGCATcTGgCcCTTG

CTTCGCAGATCGgTGGAGAGCGTTGGAGGCAACGAATCAATATTAAAAGAAGCCgTGACG

GCGGTGCTCCGAAACGCTCCGGCGCGaGTTCCTCAAATTGAAAAaGGGCGTCCCCAATGT

CCTtAGCAgTTTCAAGTTCGCCgatgaggaattgcattcggccatagCTCTCCGTTGCTT

GAATTTATATTAATATTTAAAAAATGCTATACcGTATTAGTGGTATCAAAaTAAAAAaCT

cAaaa

>contig00252 length=199 numreads=1

tgtcacaagaatatatgtatttaaaacaatttataattacaaagttcaattctacatggg

acttaacaggaaaaacgcagcactacctagcaaacaaaaaacaacacaagaggtcgtaat

tatcacgattaagccacttcatgagccagctggctttcttgaaggcagccgcagcgagaa

gtatatcgcccttcgccag

>contig00253 length=783 numreads=12

tGCGGGCGGCAACTGTGAGGCACCATCTAGCAGTACTGGTAAGGAACCAGATGCCCCTGC

CATTAAGCTAGACAGTGATGATGTCtCGATTgATGACGCcgATaGTGTTCGtCCGgACAC

TGATGACTTAAGtaGcGAGGAAAATTCGATCGGTTTCGACCcTGGTtCTGCGGCTTCTCT

CGAATCGCTGGCTGCCCAGTTCAGCAGCCCTTACGTTGACGCCAGcGTTAGTTACAACTG

TAAAGCTTGCCGAAAGCAGCTTTCATCGCACCGATACTGGAAGaGGCATTACCAGACCCA

CCTCGAAGACCGGTCTTTTGCAtGCTCGTCGTGTAATAAGAGCTTTAAGTTTCTTAAAGA

CTTGACTCGTCACGAGAGGAATCATTCTGGAGAGCGCTtGTTtCACTGTCCAGTTTGTCT

CAAGTCTTTCAAGCGCAACGATCACCTTCAtCGACACaCGCAGACCCATCAGAAGCAAGC

CgcAGATGCACcggaCGTTCCgggCAAATCGttGCCCAAgATTCGTAAGCTGTCTGCAGA

TGAGGACCGGTCTGAGAAGGGCCCGGTTGAGAATTGGATGTTGATCGcGGcTGgTGgAGT

CTGAGCTCGTCAGCGAgTTCtGtCATTTTTATGAGAATTCTGGTTTTTGGGTATCTGAAG

gaCCGcaTGGGgCTACATTCTCAtGAGCTtCGTAAAgTTGTTTcgATTTAAAaTCcATGC

GtACCatAGACTTTGGCATTtGCGATGTTTttGtCgTttAaTtGCGCATGAGGaaagctt

act

>contig00254 length=332 numreads=2

cgtctgagaattccatgaaattgtttgaagtagaagaatagcatcctcgcaagctctagg

atacatagactttaagaccaaaatcaaatagcaaaaagtatttttgccccattatgaact

tcaacagaaTTtCTGACTTCCGTAATTCGAAATAATTAacAGGAGAGAGGAATTCTTATT

ATCAAAGtttttttttggtcgtgggtcacatgacatgctaacaggtttgcttctatcaaa

tgtatcaaatgcatgttttacatgcatggacagtgtaattaaagtcttaatttacgtcgg

ggcaattgcttatctattcggcatttctttag

>contig00255 length=227 numreads=2

tttATGTGCACGATATCGATCAAGGGGatGTTTTTAAGGTATCTCGTTGTGGACTGTAGT

GGAGGCCGTTCGTGGCGTTCGAATACGTCGAGGTGACGTTGATCGTGGATGATTTGTACG

TGGTTGGTGTGCTGTTGTGCATTTGCTATTAACTACTTATTTGTTTATCGTCTTAGTTTC

TAATGTTTTACGAAAAGGGACTTGTCTAATACACTAACATGTAAAct

>contig00256 length=955 numreads=19

tCAGTAATCAACGaCcGACCTCTAACGACTGTCAAAGATGGAATCGACGACGCACTGCCC

CTCTCTCCTTCACACCTTCTTTATGGTCGACCTATAACACCcTTCCCTACTTTAGTTTtA

GACAAagTCgAATGgAAAGACACtACTTtCGAGCcGTCATCAcAaacTGCCAACtCGCGt

ctCCAACTACTGCAGCGaTtACTagCAGAATTCTGgCGACAgTGGCGCGACGATTAtCTC

ACGTTAcTACGCGAAcgACACGAaGAAAGTCAGCGAACGAGGAACCACGACGCagAACAG

AGTAAAAGTTGGCGAtGTtGtccTCgTGCACTCGGaCACCGAAAAGCGagTCAACtGgCC

gCTTGCcATCAtCaCAGACCTTAaCATTGGTACTGATGGACTTGTCCGATCcGCGAAAAT

ACGCACAAAAaaTGGCAGAaTAACAAaTCGGCCGATAACAAAGCTCTTTCCTCTGGAACT

TtCAACTGGAGATCCGAaTGCTATAATCgaGgAAAACTCTCTTCCGCAGAAATTGACAAA

CGCTTCATACTCTCCTTCGCAAAAAGaTGAGTCAaCCACtCGAGAAGACCAACTAGGCGA

GCGGCCcAAGAAGCATtGTCGAaCATACAAcTACAGAGACTGTGGTATAATTcGTGATCT

CATTCGCCcTCCCCCGGAGTGTCATGGAAAGCCATGATGAGGAAGTGGTaCgCcTGCTCG

GGGGAATGGGGCACAGTCGAAaCCTcGCCcAcACAAGGaGAGAGAaCGATAGTCTAAAGg

TCGATGTAAATATAAcTACTTCCGCGCCAAGTCTATTGgTCCGTCCTGgAaTTATATCCA

TAaTAACTTCCTCGTGAAACTGTAtAAAAACCCTTGTAAATagATATAtAGAAGTTCAAG

TTCAAGTAGAaGCAGTTGCAGTAGCGTAAACATACAAGCAGTTTAATCTATCcAa

>contig00257 length=1993 numreads=95

AGGAGACTCCAACAATTtAAGTCTGCAAGAGATGGATGAGCTTtCAGATCGCAGTTCACA

AgTTCCACTCACACGAGATCCCGAATcTCCTAacGaTTCCCCcGATCTCCCCGcTGACAA

aGAGAGAGAGTCcTGGGGAAATAAAGCAGAATtCTTGCTGGCAACgATAGgATTGgCAGT

TGGTCTCGgTAACatCtGGaGGTTTCCCTATTTGTGCCAgAAaaaTGgAGgAGGTGCATT

CTTGATTCCGTTCTTCACGTTCATGCTCATTGAAGGCATGCcTTTGTTCTTTAtGgAACT

TGgAGCAGGGCAAaGATTTCGGAAAACTGCCATCGAAGTATGGGGTGGCATCCACAGCTC

CCTGAGGGgCATCGGCTTCTCCTGCATGATTGTTTCCACCTCCCTATGTGTATACTACGT

CGTCGTCATTTCCTGGTGTTGCTACTACTTCTTCATTTCCTTCACCGCAAACCTGCCTTG

GCAGAAGAAGTTTTGCCCCAGTTACGGTTCTTATATTGAAACGCTTGCTCGCAGAAATAa

CGCCTCGATTATGTCGAACAGGAACATAACCGGGTACGATAACCTTACAAGGACCTtGGA

CCACAAGCTTGCAAACTGGCCCGACtGTTGCGTGCACGATCCACCACAGTATTACTGGTA

TGAAAAGGCTCTTCGAATTTCCTCGCACATGACTGATTCCGGTGTTGGAATGAaTTGGCA

aCTGTtCGGATGTCTCaTCTTCTCGTGGGTGTTtGTCtACttctGCATCGTCAAAGGCAT

TAAGTCAaGCGgAAAGGCTGTGTACTTCACTGCTACCTTCCcTTACGTGGTCCTGATCGT

ACTCTTCTTtCGTGGAGTCACTTTGCCTGgAGCCGAGATAGGGATTAAGGCTTTCTTtAC

ACCAAAGTGGGAACTTCTTGCCAAGCCTGAGATCTGgAGGGATGCGGCTACACAAATTTT

CTTTtCGCTGTCTCTTGGATTCGGAGCTTTAATTGCGTTTGCAAGCTACAACCCTgTCAA

CAACAACATTATTAGGGACGcgTACATCGTCGTACTTACTGACTGCTTtACGGCATTGTT

TGGTGGCATCGTGGTTTTCTCCATCCTTGGATATCGCGAATATGTTACTGGAATTCCAGT

CACCGAGAGCGGATCTGGGCCAGGCTTAGCGTTTGTCACCTTTTCTGATGCCATGCTTCT

GATGGATATATCTCCGCTATGGGCCGTGCTGTTCTTCTTCATGTTGATCTTACTCGGCAT

AGACTCCCAGTTTGGCACTCTGGAAGCAATAATAGCTCCATTGTACGATACCAAATTGGT

TACCATGAAGCGTTGGAAATTTTTGGCAATTGTCTGTTTCGtAaTGTTCGTCGTTGGATT

GTCGATGGTGGTCGGGCCGGGATACTATGTTTTCCAGATCTTCGACGACAATGCGGTCAC

CATCCCTCTGCTAATAaTTGCCTTTGCTCAGTGTGTTGCAATCGCCTGGGTTTAcGGATC

AGACAAATTTGCTGACGACATAGAATTCATGACAGGTCGCCGTCCTTGGTCATTCTGGAT

GTTATGCTGGAAGTATTTGTCACCAGTCGCCATTCTGGTCATCCTGCTCTGGACCCTCGT

CGAGTCGGTGCAGAAAAAACCGACCTACACTGCCTACATTGGATGCCCACAGCGCAATGG

AGGAGCAGAAGAATGGACGAAGAAAGTAGACTATCCAGGCTGGGCGCAAGCGCTCGCAGC

AGTCATAGTGATAGGAACTGTAATTCCGATTCCTGTATTCATGGTAAAAAaTTGGCCCAG

CAACTGGCGTCAACGTTCTCGGAAAaGTTATGCAGCGGATGGGCGAACTACATGCCCGAT

CCAGCTGCgTGGAAGACGAAACACATtCAAAGGACAATGGTGTACGAGCCAGCCAATtCA

CAAGACGACAATGAAAAaTCCTGACCAAGAGTAACCTACTTTATACAAagCGAGCGAGAG

TTGAGTGTGTTTA

>contig00258 length=322 numreads=6

gtCTGgCatGtgCAATGAACTTGtGGATtCATATGGTCAGCAaGCGaTTTCAATgTTCGT

GGAgTATTGgCcGACCCGTCTCAACTaTGCTCGCTGAtAGGGTTGTGTCCAGGAAAAGTT

GGACTTATAGTGCCGCGTCTGTCCTCcGgTGGGATCTGGACCATTTGTCGTCACGAGAGA

GGATTTCCGAAaGGTTGgAGATGAcAcGCTCTGcTtGTCCTGCCAGATAGCGgTCGCAAC

CTTGGAAGAGCAGCTgTtGgACAACAAAacGAGggACGAGtACATAGCCGCTGcccGGgC

CATGTGCAcTtATTTGCCACCA

>contig00259 length=1810 numreads=69

AGcAGTGGTATcAACGCAGAGTACTTTTTTTTTTTTTTTtttttGTTTaCAgagAaTATT

TTACGTTTTACGAACATATGAAAATCGACTGGTGCGTaGAAAaCTTTACATACTtAACaT

cAAATAAAGGcgCAtAATgAaCGAATGGGCGCAGTCAGAggcgTgCATCCaGCGACTCTC

TACACCTCACCCACAGGCgTTCAGCAAAGCTTCAGGGCGCTGCACACTTGGTTCGGCTGC

AaCtCTTTTCGCACAAGCAGATCGATTAAGTCGCCGCCATATtGCGACACTAACTGATCA

CACTCGTtCTTGAAAGCGTCGGGgAgAaCGgCGCAGACGGACTCAACAGCTTTCTCGATA

GCAGCCTGTGTAGCCTTGTCCTGTAACACGGAGTCCAGATAGCCCATGGCATACTGGCAG

ACagAGCAGGAGATCGCGGCAGGTCGCATGAaCCCcAGCTTAAAAGtaGACCGTGGACGA

CTGCCTCGGAATTTCCTCGCATTGGAAGGACACAAACCcAAGTGTTTGCAAATCAGATCC

GGGCTGAGCTCGCTGAtAaTGAGTTGGACGATTTCGTCGCTGTACGAGTTGACGAGAGTG

GTACACTCGCCCGCAAtAACCGACGGCAACTTTGAGCACAACGTATCCAGGgCACTTCTG

ACTTCAGCAGCTGTCTtGTCTTTCAGAATGCCATACAGGTAACCCATAGCGTACTGGCAC

ACCTCACAAGTGACGACGTCATTcTCcTTCTTGCTAACTGACTTGATTGGCGTTGCGgCT

ATTGACAATCTGGCAGGGCAAACACGAACAGCCTTGCAAATGACGGTCGGATCGAGCTGC

TGTACGAGCAGGTCTAtGATCTCGGGTGTGAAGGCCTGCGTCAGAGCCGAACACTCTGAA

CTCATGGTaGATGGCAGCAATGAGCACAGGGAAGATAGCGCTGCCTCAATCTCTgCTTTA

GTGGCACCCTCCTTGAGAATCGCATCCAGGTAGCCCATCGCGTATCTGCAGACCTCGCAA

GAGACAGCACCATTCGGATGCCCTTtAGTGgCTTGTCGAACGGACTTGCCAGGACAAAGT

CCGATAaGCTtACAGACAACcTCCGGTTTTAGCTCTTCAGcAaGAAGTGCAATAATGGAT

TGCCCGTAAGTATCTACCAGAGACTTGCACTCGTCTTGAACCGATGAAGGAaGATACGAG

CACAAGGACTCAACTGCCGCTTCGATTTCGGCAACcGTGGCATTGTCCTTCAACATGCcG

TCCAgATAACCCATGGCATACTGGCATAGCTGGCAAGTCACCGGaTCcttCGGCTTCACA

AGTTTGCGAGCTTCAGCGAGTGGAGtCTTGGGTTTcGTtcGCGTCgcaCAcaaGCCGAGC

TCcTtGCAAATGACGgCcGGATCCATTtCCTTgACGAGCAGgTCGACAAtCaCTGGAagT

ACTCTTCAATAAGCTCGTTGCACTCTGACTGCAAGgCcGAAGGTAGGAaTCCACaGACAG

AACGGACAGCAGCCTCGATcTCcGCcTGCGTCGTCTTgTCCTCGAGCACCTTGTCCAGgT

AGCCCATGgCGTACGTGCACACCTCGCATGTAACCGTGCcGGGCCGCTTCAGATtCCGAT

CGATTTTcTCTTTCGCaGCGAAGCCgCTCCAGATGttaaagttagaggatttctCCTTTG

ATAAAGAACTTGGTGGAcACCAATAGAtGTACTCGCaGAACGATTGCGGTGCaGGgAaGC

GCTCCAAAAAAGCGGCGATCGCCATTGCAGCATACTGGCCCACTATGgCCTCGCAATaca

agaggtagtc

>contig00260 length=130 numreads=2

tcgaaccgcttcttcggtttgtgcacagctcgcttggctggttcgtgagaacgatgggca

gatgatcgtcgttcgccaggagatttctcaaagagtatctatcttcgcgccgaccactac

tggcctttcg

>contig00261 length=158 numreads=1

aagcagtggtatcaacgcagagtacgtcgggggtgtaaaagcttatgcgcttaatagacc

attcacatggttctcgagctcttgaatagtggtcagaaatgcgaatgaaataggaatttt

tttaagccccaagagcagattccgaagtgaagttgtgt

>contig00262 length=341 numreads=9

GaCGTTAATATAATACCGGTCTGGTTTCCCTGTTAGATCGTAaGGCGCTTGATACTGGTC

CTCTTCAAGCTGCGAAAaTTCGCTTCTAGGCCATtCCTCCGGCTTGGGGTACGTCGTGTG

ACGCAAAGAATTATCAGGGTCGTATTCAAAAGCCACTCCGGcTGTCGGgTTCCACTTGGC

ATGCTCCTTCCCAAACCCTTTcTtGGCGtACGCcGTCAGTTtCAGTTCCTGTCCCTTTCG

AAgTTTCACAaTCACGATATCTTCGCTTTCACCGCCATACTCGCTTTCGTCTTTATTCCG

CGACGTgACCGGCCGAACTTCAGGATCTGAAGACTTCAACT

>contig00263 length=341 numreads=24

TACGTTTATATAATACCGGTCCGGCTTCCCTGTTAGATCGTAAGGTGCTTGATACTGGTC

CTCTTCAAGCTGCGAAAACTCGCTTCTAGGCcATTCCTCCGGCTTGGGGTACGTCGTGTG

ACGCAAaGAATTATCAGGGtCGtATTCAAAAGCcACTCCGGCTGTCGGGTTCCACTTGGC

ATGCTCCTTCCcAAACCCTTTCTTCGCGTACGCCGTCAGCTTAAGTTCCTGTCCCTTTCG

AAGTTTCACAATCACGATATCTTCGCTTTCACCGCCATATTCGCTTTCGTCTTTATTCCG

CGACGTAACCGGCCGAACTTCAGGATCTGAAGACTTCAATT

>contig00264 length=159 numreads=2

AAGCAGTGGTATCAACGCAGAGTACGCGGGGGCGCCAGTGCGATTTCACCACAGCGAACG

TTGCCTCACGCTGAcaaatgcccagtgatccaatggcgtgtgaagtctatgtcaatataa

ctcatatagacgtctaaacagaaagactcatttcaagca

>contig00265 length=617 numreads=4

ccatcaacaatttgcctgtcactgagcagtctttcatagaacagttgcatgggctgagcc

acaaaatcaacgttgtgaaGGAGCAATCTTATAAGGGTGccgTGGCGTGCAACGATGTTC

GGGATATTCTTGACAAGCTGCGCTTGAAGGCTGTGGCAAAAaTTAgagagtttatcctgg

cgaaaatctacgcgtgtaggaagcccatgtccaactaccaagtgtcacagaaCACGTTGC

TGAAATACAGGTACTTCTTTGAATTTCTCCTGGCCCATCATCGTCAAGTTGCCCGCGAAA

TCCGCGATGAGTATGTTGATACCATgggtaaaatctacgtgtcgtatttcaagggctaca

tctccaagttgatgaaattgcaatttgaggaagttgctgaCAAAGACGATTTGATGGGAC

TCGAAGACAAtgCAAAGAAGGGCTTCTTCtCTGGGAAAACTCCTTTAAAGAATCGCTCTA

CGATTTTcagcttgggtagcagaggtcttgtcctgacgcaggagctggaggaacccatca

ttgtgcctcacgctgcacagagaagcgaaaagcgatactcatttgaagcgctgtttcgga

gtcagcacttgctcttc

>contig00266 length=202 numreads=2

TTTTATTGCATTTtGCAGCTCTAGTATttCACttCTGATATTGAGTTGCATATTGTGCTG

CTATAAGAAAATGAAATGCTCTTTCATggCCAAAGTTATAgCGTTTTtGttttcctgcga

cgcccgggcctgctgaacagcctcttctgcctgctaaagtggtttcagtacagaagttgg

aagcgtcaatgaggcgaaggtt

>contig00267 length=178 numreads=5

GTTTTGTACTGCGAggCCcGCGTCTGCTGAACGGCGTCTTTTGCCTGATGCTGAAGGGGG

TTTCAGTACAGAAGGTGGAGGCGTCAGTGAGAcAaGAGTAGAGaTtGTATCCGgaGaaTT

TAGCATGCCcTTACAGCCTATTAGCTCTATAAACGCTAAGGAtCCTTCcAACGCCTTG

>contig00268 length=721 numreads=45

ttCCATTTCAGCCCGAatGAATTTTTtACCAATGAAGCCCTAACAAAGACGTACAAAATG

AAGTCTGAACCTGATCCAGATGATCCGTTTTCTTTTGAAGGTCCTGACATTGTTGGTTGC

AGTGGGTGCAAGATTGACTGGAAGAAaGGGAAAAaTGTCACTCAAAAAGTTGTAAAGAAG

AAACAAAAGCATAAGGGACGAGGCCAGACTAGGGTCATCACCAAAACCGTGAAGACTGAT

TCGTTTTTCAATTTCTTTGATCCCCcTGAAATTCCGGAGGATGAGGATGAAATGGATGAa

GACACTGAAGAACTTCTTCGAGCAGATTTTGAGATAGGTCACTtcATGAGAGAGCGTTTG

ATCCCCAAGGCGGTACTGTTCTTCACTGGTGAAGCTATAGAAGATGATAGCGATGATGAA

GAGGAGTTTGAAGATGAAGAGGgTgATGAAGGTGACAACGATGAGGAAGAGGACGACGAG

AAAGATCCCGAATACAAGCCCTCGGAAAAGCCTCAAGAATGTAAACaGCAGTAGCAAATC

TCGTGTCATTGGCTATCCTTATCGtAAGCCGCtATTCAAAACGGGTACGCAAtGgAGAAG

CCACtGCGCAGGTTTCCCGATGACGTTTTACTaCcGCACATCTGTgATCGGACTTGATTT

tCTATCgCTGTTTGTCTTAAAATTGTCTGCAAGATTTTCCACACGATTCTGGAaGAtGta

a

>contig00269 length=205 numreads=3

GTTTTCcAGCTGGCTTTATGGACGTTaTcGCCATtGAAAAGaCAGGTGAatCgTTTcGCC

TACTGTAcGAtGTGAAAGgtAGATttgTTTGtCACGCAATTGGTGCTGACGAAGCTGGAT

ACAAGCTTGGCAAAGTGAAGCGTGTCGGTGTTGGCAGAAACTCGGTTCCTTACATTGTTA

CCCATGATGCTCGAACCATCCgata

>contig00270 length=819 numreads=23

AAGCAGTGGTaTCAACGCAGAGTACGCGGGGCTTTtCCcAAGTTTCGGTATCGGGGTTTG

CTAGACAAAATGGTGAGAGGTCCGAAGAAACACCTtAAGCGACTCAaTGCTCCTAAGCAT

TGGATGTTGGACAAgCTCAGTGGGAACTTTGCCCcACGCCcATCGCCAGGTCCTCACAaG

CTACGAGAAaGTCTTCCCATGGTGGTCTtcTtAAGAAaTCGTCTTAAGTATGCCCTtACA

TACAGCGAGgTGACAAAAATAATGAAGATGAGGTTGGTGAAGgTGGATGGCAAAGTTAGG

ACTGACCCATGTTTTCCCGCTGgCTTTATGGATGTGATTTCCATtGAAAaGaCTGGCGAA

TCTTTCCGTCTGCTCTATGATGTGAAAGGTAGATTCGTGTGCCATCCCATCGGGgCTGAC

GAGGCTGGGTACAAACTTGGCAAGGTAAAGCGCGTGGGTGTAGGTAGAAACTCAGTTCCT

TACATTGTtACGCACGACGCTCGAACAATTAGATATCCTGACCcGTCCATTGATGTAAAC

GACTCAGTTGTGATTGACATCAAGAACGGTAAAGTAACAGACTTCATCAAGTTCGATACC

GGTAACACTGTCATGGTGACTGCTGGACGCAACACGGGGCGGGTTGGAACTATAACCCAC

AGGGAGCGCCACCCGAGCTCCTACGATATCGTGCACATTAAGGATGCCGCTGGTCATACA

TTtGCCACCCGTCTAAGCAATATTTTCTTGGTGGGCCGTGgAAaCAAaTCTATGGTAACA

CtGCCCCGTGGAAAGGGAGTCCGCCTGACCATTGCCGAA

>contig00271 length=165 numreads=2

cTCGTATagATCCCCAGACACATTGAATGCGATCCTGTCGTCCTTGCAGTGGTTTATATA

ACCAGACTTGTTTTCGCCGTcTTTtATAGTCATGTCTTCTCTGcAaGAgAattttGGTCC

TTTTCTACAGTGTGTCACGACTCTAGACCAGCATCGTATTAACAC

>contig00272 length=183 numreads=3

GTGGGGACaTGTctACTAAGGaCGAAATAGATGTTTCCAGTgATGATGACATGTCTtCCc

AGgAGgAGGAAGAAGTCCCAGACTCcTTGAGCAAggaCAAtGAaGTtAaGCAaGtCAagA

ACCcTgAaTctGGGAAAaTGATTtCTTtGGTATCtAACTTTtGTGTCGaaggtgttctgg

tgc

>contig00273 length=116 numreads=4

AGCAATAGTCATTTTTGCTCCAGGAGATGTTAGACcAGGGCACATtATGTTAgCTCCgCT

AAGgATAAaCTtAATAGcTCCcTTGTcTACTTGCTcATGTGTTAGCATGGTAGGAT

>contig00274 length=599 numreads=7

aCACCAGAGCAAAAAAAGGGCTTGaaaTCAGGgAaGACtgTCATACGCTTCTATAAAAGT

CGATCGCTGGcctCGAACTATCCCGTACGCAATCGCACCAGGTCTCGCGtCCTCTCAGGA

aGCaGTTACGGCGATCcAAGCAGCCATCtATGacTACCAGGCCTTTACGTGCTTACgaTT

CGTTAGGAGGaCAAGCGAGAGAGGATACATGtACTtCTATCTAGGAGATGgATGTtcATC

TCCAGTGGGCTACACTGGATCAAGAAACGATATTTCACTTgggcacggttgctgggataa

agccacggtcatccacgaaaTGGCCCACAGCTTAGGACTGTATCACGAGCAAAGTAGACC

CGATCGCGATAACTtcgTTGAAaTTTtGTGGcAAAaCATTGAGAATGGgATGGCGTatAA

TTTTGaTAAACAGCCGGCGTCCGAGATCGATTCTCTAGGTACTCCGTACGAcTACGCTaG

CGTCATGCACTACGACTCAACGtCcTTTGGCATAAACGGACGACAAACTATTcGAacGAA

AGACGCGAGTAAGcAAAACCTCATTGGGCAAAGTCcAGgTCTCAGCGCCATCGACAAgg

>contig00275 length=232 numreads=3

AAGCAGTGGTATCAACGCAGAGTACGCGGGGACGGATAAGTCTCTTGTTTATTATCTTTT

GAGAGACTAAGgAAaCTGTtGTTGTCACcAAaTAATGGCTAcTGACCaGCcTGCgCcATA

TGGTGgTTgTGaGGGACCTGAtgCtaTGTaTGTTAAaCTTaTTTCAAGTGATGGTTaCGA

aTTTATCATCAaGCGggAatATGCTTtGAcaTCTGGAACTATTAAAGCCATg

>contig00276 length=439 numreads=8

AGGGATGAATCAGGGGGGCAAGGTCAACAACAAGggTACAtGAACCCTCAGcAATATgCT

GCTATgtACGggTaTCAaCAgACGCAgCaGCAGCAGCaGCAGCAAAGTTATGGGGGAGCT

GCCGCTCAAGGAAACGGCTaCGAaGGAacTCAAAGTAGTTATGGGCCATACTAATGACAG

TACCAGGCTCCGCAGATTgcattgcatttgAaGAAGaaGTTTCTATGCGACTtGTTTGtT

AATGTGAACCTGCCCGcTGCTAaTGGTGtGGacGCTTGGgAAGTGGATTtCTTGGTATcT

CGgAACGATCtAATTTAGCCcTGAgCTAaTATtGCcaaGGGTAATCCAGTaTCCCAtGTT

TAGATGGGGAAAGAGtGTGcTAAGACGAAGGTCTCATCGTGTATTACTTGCACTTGTAAA

ATAACCCTTGTGGTTCTaa

>contig00277 length=1059 numreads=144

TTGGGGTGTCATTCCTTGCATGTTCATAGTTTGTTGCAACTGGGGGTTCACCATGTTGAC

TCCCATGCCACCAGGACCTGGAGCTGCTGAGCTTTGCATAGGGTTGGACCCACCAGCCAT

TGAACTACCACCCCCAACGATGCCAGAACTTGGACCGTCTTtGCCCCACCAACACTTAAG

ATATGAGCCGTGCATTTCAGATCCATTCGCTTTCACGATGGCGGTAGCtGCAGCTTCGTG

GCTATCAAATTTGATGAAAGCAAATCCCTTGTCCACgAaTATGCGGgTATCGACTATGTT

TCCGAAAGTCGAGAAATGCTGCCTCACAAGATCGTTGGgTATGCCACCAGGGAGACCACC

AACATACACAGTGCAGTTGTTATCAGTTGCAGCTTGATATACTTCGTTGTAGTTCAGCTG

GTTTTGCTGCTGTGCACCAGCCTTGTTTCTCGAAGCCCAGTTCGTCTTGATACACCGTGT

TCCTAGCTGAGCACCATTCATCGATTCAATCGCATTTTGAGCGTCCATTTTATTGCTAAA

CGACACAAATCCGAATCCTTTGCTTACTCCACTCTGAGGTTCACGAACAACTTTtACACT

TAAGACATCTCCACAAGGCTGGAAAAACTTCTTCAGAGCATTGTCGTCAATTTTCTCATC

CAAGTCCCCAACATAAATAGTCACACTGGTTCCTATAACTTTGGGTGCACCAGAGCCCCC

ACTGTTTACAGCCCAGTTCACTTTTACTTTCTTGCCAAAGAACTCTCTGCCATTTATAAA

TGTCATCCCTAAATCAGCAGTGCTGTTATCAGCAAACTGTACAAAaCAGTAGCCCTCATT

TGATGAACCATGAATGTCAGTGGGAAAAACCATCTTGGCTGACAAAACCTTGTCAGAAAC

TTGGGGTAAACCTTTGCGAAATATGTCCAGCATCATATTTTCAGTAACACGCTTATCAAG

ATTGCCAATATACAGTACTGTTGCTGTTGCATCTGGTACATCCGCAGGCTCCCAGGGGTC

ACTTGCTTGCAAACCCTTCGACATGGCTGACTTGTCTAA

>contig00278 length=593 numreads=8

atcgcccgacatagactacagaatattgaagaaacactttcagcattttgggagcgATGG

CGGACCGAATATCTTGCTCTGCTACGCGAACGCCACGAGCGCGTTTCTACACGGGGAACA

GCTGCCGAGAATCGAATGAAAGTCGGGGACATTGTTCTCGTGCGATCAGATCACGAGAAA

CGaGTGTGTTGGCCACTTGCGAAAaTCATGGACGTTGTGCATGgCgCTGATGGCATGATT

CGCTCTGCGAACATTAAAACtGCGAACGGGAAgATTACAAATAGACCTATCACAAAaCTG

TACCCGCTCGAACTTgCTACATCcGGTGCTGAGcAACCTCCGAcAATCGATGAGCCGAAg

AGGGaCACTCCACGACCGAAACGACTAGCTGCaGAAACTGCACGATCACAGATCCAGCTA

CAtCAGTTTCTTGGCGTCTCTTAAATTTTTGCCGGCGGGGaTGTCATggAAACCCCTGGG

TTCCATGAGGAAGCACCTGGGTACGAGATTCAAGGTGGCTGACTTcctcgtgcttcctga

ttggacttaactgtaaatattcgaaatggcatccaaagcaagcagacagccat

>contig00279 length=218 numreads=7

acACaGTGTAGcGCAGCGTAGTGTAGTGTAaTGTAGAGTaGtAGCCtGTCATGGCGTAGC

AGCAGATtGCcAGTGCAATATGcAGTtAGGTAGATAGTTCGATAGGTGACGATCGCGTAC

ATGCAaCTGCTGCCTTCAaGTATtGTATTATGCAGATACCTtCTCTCTTTCCCTGCAGCA

GTGTGGTGCCCGCGTACTCTGCGTTGATACCACTGCTT

>contig00280 length=162 numreads=2

gAaTGGCAGTTGAAGCaaTAGTTTATTCAAGAGGAAAGCTTCAACTGCTTGATCAAGTGT

TGATGCCTCATTCAAttAaTTATGTTGAAATAAGGAATGTTGAAGATGGCTACCTAGCTA

TAAAAAACATGAATGTGCGTGGGGCTCCTGCTATCGCAGTTg

>contig00281 length=254 numreads=4

aagcagtggtatcaacgcagagtacgcgggggtgcctcatcgagcacttgCACTCGGACG

TGGTGGGTCcGCTGTtgaTAGaGAAACATcGAGgTCTTTctCTTCATAGCAGGAACCATG

TTGGCAGGATCATCTAACCTCAAGTTGGCACTTTACCTTCGCCATGCTTGCAAaGGAGCT

TtATGCTCTGCTGAAGCTCGAAGATGTGCTTCGACTATTTATGATGAAAATGGTGGTTTT

GtGCATCCAGTCAT

>contig00282 length=315 numreads=5

aCAAATGAACGTTGCTTATGGGAAAGAGGAAAAaTTTTtGGCCTGCAATGGAAGACGGCA

GCATATTTAGCAGTGGTGTCAGGTACACAGgAACCAAAgttAtCTtGTAATAGTGaTAAC

TGCCcTCTGCAGAGTAtAGACTTGACAGAATGCACATTtGATGCGGATAGTCGgTCGCAC

TGTGCTCGAAGCTGTgtCAGAAGACcTAGCAGAAAGTCTGGAtGCACaTGACGgTTtAtA

TTTTCgaaatcatcaagcgacctttcgtggattctaccagtccagtctcggcgtcgaaaa

agtcatttcgtcttc

>contig00283 length=230 numreads=4

ctaaTGTGACGCAGTtGCTCTCCtGGGTTtCCAAGAAAGGGCTtCTCAaTTCTgAgTata

TtGTTTGTcATtAgtATCATgATTGTAgCtGGtGCGTAAGTTGCAGGTCTTTAATtGCCT

TTGATTAGTTATGTTCATTCTGCTTTATGGCTAGCTTCATTCAAAGTTAGTTTTTCATGC

CAATGATGCGcAATACTACTTTCATTaTTATTAATGTTTTGTCCAAGTTT

>contig00284 length=402 numreads=7

ggTTCACTAACAAAGGCGTTATCATCGTTTCTAAGTATAGGACCCACTGTCTCACAACAA

GCACttGCCTCTAAAGAAAGCTGTGAGCTTTCCGGCAGAGCTCTGTCGAAGTATGCATTA

TCAGGATCAAGACATAGCTCGGAAAAACGACTCGCAATTTTCATACAACGCgcagcGTCG

gcTTCGgCAGGATcATGcAAACGAGGCAtGATTtCGGAcTCAtcTAaGACtGAaaCATCA

GCACGCGATTCAAGTTCTCCCATCTCGTTCgAaaCATTTACAGATCGCCACgCGATtcAg

CAAAATGCTCATCGGGGCGAtCAGACATGCTTCCTTCACGTACGGGAAGACCACCTGATG

ATTGGTGCTCGCATCATATACCACAaGGTCTCGCTAGATGTt

>contig00285 length=236 numreads=2

attcttgaagtccaggggctttggagcatgctctgttctacccctcaaagacgtactagc

tagcacttggctaggtatgacagagctagcagattgaTAGCTATAAACCTGTGAGCTTTG

TTGTGGGGACGACATTCACTGAATCGCAAGTATGTAACAATTCACCAGGAATCATCAGTT

AGTTTCAGGATATCTTCTCATAAGAGCACCCTCAATGTCTTCAATCAATAGCAggg

>contig00286 length=678 numreads=10

tAACCGTCcGGAGGgtCATAATTCTGgCACACGACAAAAGATTCTATGCTCGAATTCCGA

CTGCTACGTGGCTTGCAAACTGTAACATCCcGAAAAaaTAGcTTGAGCtGCGCgTAAAGC

AaGTCAaCGTCCTTGCCGcGAAaGaTCTtGGCaaCGAAAttGCCGCCATGCTTGAGAATA

TGGCTGGTAATGTTGAAGgCGGCGaGAagCAGCTGTCCCTGGATATACTCGTCAATATCG

TGGAGACCGGTTACatctggggctCCGTCGCAAACAACCAAGTCGGCAGGCTCTCCTTCG

AAGTGACCAATGATTTCTTTGGCAGTAGAAAGCTTAGTGATATCCCCTTGTAGCTGAAGC

ACGCCAGGCAAGGGCGCCATCGCCTGCAAATCCACGGCAACAATTTTCACGTCCCTgTtG

CCCCCcGGtGCTCTTCAATCAAttttctgctgagtacTTGACTccAGCTcTcGGGCGCAG

CACACAAGTCCACAACACGGCTTACGCCTTCAAACAGATTGAAGTCTTCATCTAGTTGCA

ACAACTTGAAGGCACTGCGGgCACGCCAGCCACCTTCcTtGGCAAGACGATAATACACGT

CCCGCTtGTCTttGgAaCtGCGTCCCATcGGAATcGAATTCCTTAGTCTCTCAaCAGATC

TTTAACAAAaGAGACcTt

>contig00287 length=106 numreads=1

gcagtggtatcaacgcagagtacgcggggctagtagatcggcatgaccttatggaagcgt

caacagcacaggagaggcgcatctactgccagcttgttgtgaaaac

>contig00288 length=917 numreads=28

TGgcACTTgTGTTAGAAGGGTGAAGGCTGTTGTTCGATTAAGACTGGTCGAGACTGAGAT

CGGATAAAAGCGAGAATGTTGTCTGTtGCTGTGcTTCTGTGTCTGTTATCGACTGGCCTC

TCCGcGTCTTGGAAAAAaGTaGTGGAGCAAGAaGTAAaGGAAgaGAAaaTAAaTTtGCGA

GGGTTTCGAATTCCTCATTtCCATGGCACTGCCAGGAAGTtCACCGGATCGACAAGGGCA

AGTAACTTCACAATGCTGGgCTTCTCTTGGTcAAACTGCGgCAgCcAATCcGACCcGAtC

ctGATAAAGCAGTTGGCGCTATCTCCAGATCCAATCAATGTTCCGGGAACTCTGCAAGCT

tCAGCTGATGTGgTAATCGCCAAGaCTGAGCCTGTTCTCTCGTCCGTGCAAGTCGTCATG

AAGAAGAAAGTTCTGGGCATGTTCATTGAAATTCCCTGCCTTGACAATGTTGGCTCGTGC

ACGTACGAAAATGCCTGCGATTTGTTGGCGAAGTTGGACTGTCCGCCTGACCTTATCAAA

TACGGTGTCACGTGCCACTGTCCGTTCGAGCAGAACGAATACAACATTCCGTCTGCAGAG

ATCAAAATTCCGTCGTTACCCCTGCCTTCAATCATCGAGAACGGAGAGTTCCAGGTGCAA

GCAACATTGCTGAATGGAAGCGAGAGGCTTGGGTGCtACCAATTCGATTTCTCGCTGCAC

AAGAACGACtAAACCGaCCATGGCTGGAAtAaTGTGGACGAAATTTtGTGCAAGTTGATT

GGTGTATGATGTTACAAGAAATTAGCAAAGCAAAGGCTTTAGAATATCGTGGAACAGAAT

TTTTGttATTGCGTTTTCTTTTTTcGATcAcAaGGaCcGCTTTTGTCtGGTgCAtATtAC

AtATGgTGTGCgTAATC

>contig00289 length=801 numreads=166

CCATGGCATGCATCATTTCGTGGAGGGCGACGCCTTTATCTTCACATCCTTGGCCAaTCG

ATATTTCTTGCCGGCCACCCGTCATACCCACGTACGACCAaCATCCCTCCGAGTTGTGAG

TGAACTCaGCGTAATCCTTTTGaTTAGTaCGAGGCACCCACTTGACGCAAGGAATGATGg

CATTCATCTCCTtAATTGCcGTTTCAACCGCCGTTCGCGCTTCTTTGCCGCTACCTTCCT

CCACACTGGTTGATGCATGAAACGaGTAaGGGATCTGTCCGTTTGTCCACTTTCCGGTCA

AACTAGCATCCAACTGGATCTTAACTCCCTCtGCGATCAGCTTCTGCAGTGCAATTTGCT

CCCTGAGcTTAGCTGTGTTTACCATGTCACCCTCGAATACAGATACGCCACCAACTTTCT

TGTCTTTATCTATTTTATTtGCAGTCAAAATCTTCGACATGgCGCTCTCTGTCGAATCTT

CCTCGTCAGCATCCCTCTTAGCTTTTACTAAATTTTCGATGACGTCCAGTTCTCGACGAA

AGGCCTCGAGTTtATCCAAGAGCTtCCCTAACTTGATGACGTAGGtGTTGTCCGAACGAG

CTTCCATCGGCGATTCTTCGTCATCGCTCTGAGCGgCCGCACCGCCGTAGTCTCTCTTCA

CCAATGCAATCGGAGGTTTACTGTTGTCTTCAGGCACACTCCTTGACCAGGCGCATAATG

CGCTGAGGGCCAAACACACCAGCTGAATGTAGTTCATCTTGATCGCGAGCCCCcGCGTAC

TCTGCGTTGATACCACTGCTT

>contig00290 length=143 numreads=3

cTAACTTAATATATTAGAGCTATTTCTGGCAATGTCGAGGCtGGGAAAAGgCACACGAAA

CAaTtaTAAATGGAaTCAatGTGCACATTTtCTTTAgTTgTGgtATTTGTTCTTTCTGAT

GGtATTCATTTTCTGCAGTTTGa

>contig00291 length=532 numreads=16

tccgcggaatCTACATTACTATCTTTCTtCGAACTTTTcccACGAATTTCtAATTTtATT

CagaGTTGCTTGccTCaCtGGCCCcGCGTTGacGTTtAaGATTTTTTTTCTCGTCACTCT

GATCGCCATCACTACCTGAATCTCGATGGCTGCGTTTCTCTTTTTTCTTCTTGTGTTTCT

TcTTTTCTTTCTTAGATTTTTtAGGCTTCACAGGCAGCAAAGTCTCAACTTCGGTAGTGT

CGGATACCTGCACAACCGGAGCACATCCAGTTTCTATTTTATCTAtATACTTTCcACTCT

CATCCTTCTCGTCACCTTTCGgTCGTCGGTACTCTTCCACATGGTCGACACGGATAGTTC

GgCCACCAAGCTTTATTCCGTTGAaGTTATCTACAGCCAAAACCGTGCTTCGCTGGTCcT

CATAaCAGAGGAAGCCATAACCCTTCGATTTCCCAGTTTTTTTtGTCACGAaCAAGaTtA

AGaTTgAcAaTCTcACCATATTGTGAAAAgCGCATAATATGTCTCCTTCTGt

>contig00292 length=1677 numreads=33

AAGCAGTGGTATCAaCgCAGAGTACGtCGGGgACTGTAAACGCCAAGGAGATTTGAGTAG

AGCCTGTGGGCTAAGGAGCGtATCTGGTTGTGAGTTTGTAaTCATGGAAAaGAAACGtCT

AGCTTTAGTGCTTTTAATTTCTGCGCTCGTCGCAGCAGGTGAAAAGgaaGAcAagAaGGA

GaaGaaagAAaaTgTTGGAACGGTAGTTGGGaTTGACCTCGGTACAACTTACTCCTGTGT

TGGTGTTTTcAAAAAtGgTCGGGTTGAaaTtATCCCCAATGATCAAGGTAACCGAATTAC

CCCTTCGTATGTTGCCTTCACCCCTGAAGGAGAAAGGCTTGTTGGAGATGCTGCcAAaGA

ATCAGCTTACAtcaaatcctgaaaatactgtatttGATGCAAAGCGTTTAATTGGCAGAA

GTTggAgTGAAAAAaCTGTGCAGCATGACATAGCACATTTCCCATTCAAAGTAATCGaGA

AAAACTCAAaGCCTCACATCCAAGTCCAAGTGGGTGATGATACTAAaGTTTTTGCTGCGg

AAgAAaTCAGTGCCATGGTGCTGTCAAAaaTGAAGGAGGTTGCTGaGGCCTACCTTGgCA

AAAAAGTTaCCCATGCTGttGTTACtGTCCCAGCCTATTttAATGaTGCCCAGCGCCAGG

CCaCAAAGGaTGCTGgTACcATCGCTGGTCTGGTTGtGaTGcGAAtcATAAaTgAACCCa

CTGCTGCTGCcaTTGCaTACGGTctGGACAAGAAAGAAGGCGAGAAGAACATCATGGTGT

TCGATCTTGGTGGTGgTACTTTTGATGTGTCATTGCTGACTATTGaCAATGGTGTATTTG

AGgTGGTAGCTACAAATGGTGACaCCCATCTTGGTGGAGAaGaTTTtGaCCTGCGAGTTA

TCGACCACTTCGTAAAGTTGATTAAAAaGAAGAAGGgTgTGGACATTCGAAAaGaCAATC

GCTCTGTCCaGAAAGCTGCGTCGTGAAGTCGAGAAGgCGAAGCGAaCaCTCAGCAACCAG

CATCaGACCAGAaTCGAGATCGAGTCCTTGTACGACGGCGAaGACTTCTCGGAGACTTTG

ACTCGAGCTCGCTtCGAGGAACTCAACaTGGATCTGTtcAAGTCAACcTTGAaGCCGCTC

AAACAaGTGCTTGATGACgCTGGATTGAAGAaGACCGaTATCgaTGAAATCGTACTTGTA

GgTGGTTCTACTCGtATtCcGAAAGTCCAGCAGTTGGTGAAGGACTTCTTTGaCGGTAAG

GAGCCTTCCCGGGGCATTAATCCTGACGAAGCCGTTGCTTATGGAGCCGCAGTGCAAGgT

GGAGTATTAAGCGGTGAAGAAGACACTGGTGAGATTGTCCTCCTCGATGTAAACCCACTC

ACTCTTGGTATTGAGACTGTTGGCGGGgTAaTGACCAAATtGATTGGCCGAAaTACGgTT

GTtCCTACAAaGAAATCGCAAATCTTCTCTACAGCTGCTGACAATCAGCAAACGgTGACC

ATCCAAGTATATGAAGGCGAGCGACCcATGACAAAAGACAATCAcTtGTTAGgAAAGTTT

gACCTAAaTGgcATTCcGCCGgCCCcTCgAGGtGTGCCGCaGATCGAGgtcACGTTCGag

ATCGaCgTAaaTGgCATtCTTAGAGTTAGCgCTgAGGACAAGGGtaCCGGCAAGAAA

>contig00293 length=568 numreads=5

CCAGCAAAAGTCGTCGGAAGGTGCAGCGTAAAGAAGTTTTCCCTTCGAGAAGGCAGTAAG

CATGAGGACTTCGCTCTTAAAGAGGCACTCGCCGAAGTCGTTAATCTTGCCGACAAGATG

AAAGATGATGTGAAGCTTCTCCTCCATTTCCTTGCTTTGCATTTTTACGAAGCGGAAGCG

GTGAAACTGCagtcgaagttttcgtccgtcttgaactttttCGAGGCCCACATGGATGAT

GTCTGGCCTAAGCCAGCCCCTGGGGtcaccgcctctctcgatatgcggtatggttcagac

tcgacggtggagggtattctgggtgaaaagaatccgcgatcgaagaatccgtcacctgcg

gacgataacactcttcccgagccgacaaaggtggtattgagaagtaatattCGATGGAAG

CTCGAGTTTGCtgCgTAaCACTCTGgCtGCaTTCgTGTaCAAgggACGaTTgTTctgaag

gtcgcggcaatgttaatgttaaggtcaagtcaatgttgctgaatttatgactacctagag

agaaaattttattttcttctaaagggaa

>contig00294 length=799 numreads=21

ccccccccctgTAGAGGAGCTTtaaTAGAAACCAAAAGCCCcACCCAAACCTTGTCTGAT

AAACTGAAACCCTATCCAGCGCTtCgtCACcTGGTgTTTaTttacAaTCtAaGAGTaTCC

cATTTGGCGCTACCGTACcAGACCACGGCcTTACTGCAACGACACGCTGTGGCCGTAgAT

ATCGTCTGTGGCTCGTGTGCGAGGTgCTCGATGCGgATTTCTtCCATACGACTCTTACTT

CGACATCATCATTTTGACGAACTCTTCATAATTTACTTGTCCATCACCATCAaTATCGGC

CTCCCTAaTCATTTCATCGACTTCTTCATCTGTtAGTTTTTCCCcGAGGTTGGTCATCAC

GTGACGAAGTTCAGCAGCACTGATATAACCATtGCCATCTTTGTCGAaCACTCGGAACGC

CtCCTTTATTTCCTCCTCAGTGTCTGTTTCcTTCATTTTgCGTGCCATCATTGTCAAAAa

CTCTGGGAAGTCAATAGTTCCATTGCCATCAGCATCAACCTCATTGATCatgtcctgcag

ttCAGCCTctGTTGGATTCTGTCCCAAACTTCTCATGActGTACCCAGCTCCTTggtGGT

GATTGTACCATCTCCAtCcTTGTCGAAGAGGgAGAATGCCTCcTTGAATTCAGCAATTTG

CTCTTCGGACAACTGATCAGCCATGATAAACTGCTAAATCCGGGCGGTAACACCACGAAG

AGATATGAaGGTATACGACGTGCTTGGATGAaGaTCGGCAGCAAGTCGTTCACCGCGAGg

CAGGTACAaGAATCcAAGC

>contig00295 length=101 numreads=1

gagcgcgatcgtgaagtcgatcgtagagatcgtcggcgaagtggagatcgagaacgcgat

aaagaaacgcgacgcagtcgtagtcgcagtcgtagtcgcag

>contig00296 length=209 numreads=3

cGAAGTTTTTtgCGGTGCATGCgCTACTTGggACGATtGaTGATTCgCTACCaTGTAAGa

TTGTTCGATCTAATCTGaGCtaCAAaCAATTGgTTTATTGCAAAGTaTTTTTGCaTGGAG

AaTgTaCGTAGTTATCGGgTttAGTCTGGGCCTGGCTCAGCCTGTATCGCAGTTTTtGAC

GCCCGATCAAATGCCATAAATAGTCGAGG

>contig00297 length=176 numreads=1

cttcaatctctagctgccggacggttctcacaaaatcatgaacacgcaaaaacatctgca

aagccatcacgaaataaaattcttgcctgaaaactctttcaatacatcttctggaagcgc

cggcagcagcattggtcattaggcttcattgattacgcgtccagtcctttgcaaca

>contig00298 length=180 numreads=15

cTGTCACCAGGTTTCAGAGGGCTGAGAAAAACACGAGCCAGAAACACGTTtGACTCGTTC

TAGAATCCGTACTTTGATtGCGTTTGACGCTTCGTCTGCcGGCTCTCTGCCCATGCTTGC

TGTAATTCAAACTCCCTTTCCTTGGCCTGAAAGGCTAGATAAGTAATTTGATGTTTCCGT

>contig00299 length=411 numreads=6

GCCAGAAATGCAAAAAGAGAAGAAGGACGTAAAGCCTCCTGTCTTTGTTGAATCGCGGGC

CTCtgCGAAGAATAGCGGAACCCCTGCACCAGACATCACaGGCAAGAAGTaTTCGCAcAA

GCcTAGtCCCGACGTGGAAAACAAaGaCTCGCCGAAGTCGGGGGgCCCTGTTGACAGGGt

ggataaggcaagaggtaaagggccgaagaaaaaggtcaaaaggcGTTAGGAAggtCgAAT

AtaGTTCCaGTgAcAGCAAGCGaGACAAGaGcAAGTCATCGGACAGCAGTGACGAAGAGA

gTtCAGCGAAAAGCGGTCAAAGCTCTGAaGAAGGCGACGtGAAaTCGAAGAaGAAGATtG

TGGATAAAgCTGgCGATAaGgTtGcGCTGACTAAGGaCGaaaaaaaaaaaa

>contig00300 length=186 numreads=2

aaggtccaTACGCGAGAAGCTTAGAAAGGACAGCACGTCCGAGGCGCGATTGACTTAGCT

AGACTTGACCAAAAATAAAAAttCACCCGTGAATAACATGATGTGCTTGTTTTGTGTTGG

GAGGTCTTCTACTGGAGCGGAAGCACAAACTGTTATAATCACTTCCGgCCGGCGCGCAGT

TGTGCg

>contig00301 length=1735 numreads=63

cACCAaCAgTAGaCGTACCGgCAGCACATCTCAGCAACGTtAGTGGTgATCGCGACCtCT

GGGTCTCGgCGGGGCCTTGATGATGTCATCCACACGcAGAACCATCTCTGCGgCTTCcGA

TGCTGCCACGAGCATGTGCCGCTTtACCTTGAACGACTCTGTAATACCAAGATCGACCAT

GCTTTCGATCGCACCGTGGGTCATGTCTAATCCTGCAGTCGAGTTtCCGGCGgTATGAGC

AGCCCGAAGTTGaGACACCAGTTCCGAACTGTCGTACCCGGCATTATCAGCGATAATAGT

CGGAAGCTGTCGAAGGGCTTGGgCgTACGACTCCATAGCCATAGCTTCTTTACCGGGAGT

CTTCGCAGCCAGTTgaCTCACAGCGTTGGCCATCAACATCTCCGAGGCGCCGCCACCGTA

TACAATTCGTGTTTCCTTCACAGTCTGCTGAAGAACACAGAGAGCATCGTGGAGAGCGCG

TTCGGCCTCGTCAAGGaTCTGCTGAGTGGCACCACGAAGCACAACACTGCATGCCTCACC

CATCTCCACGCCGGAAAAGTGAATCAACTTGTCCTCGCCAACCATGACTTcTTCGATCAG

CTTGCACcTCCCCAACTTGACCAGTtCAGGgTTGTGGAACGTAGACACGATCTCTCCACC

CGTCACTAAGGCCagACGCTCTACGCCTTCGAAGTCAGCGTGCTCAATCGCcATGACTCC

AGCATCCGCAAACAaCTGCTCCGGGTAATTGTAAATCAGTTGGCGGTTGATAAAaCAGTT

TACGTTGtGCTTCAGAaTTTGGTTAACTTTGTCTTTCATTTTTTCCTTTTCCGCGAGTTC

CAGCTCTGCAaCTTTTGCCACGgAGTCGACTCGCACTCTGGATCCAAAGACTTTtATTTT

GTCCGTATCCATGGGTGTGTTTGCGATGAGAATGCGGGCATTTTCTATTCGCTTCGGCTg

aTtCgTACctACCTTTTtATCGAGCAGGAaTCCTTcGTCCAAGTACGAGTCTtCCAGGCC

ACCCCCTAGCTtCTTGATCACTTGAATAGCATCCAGGTTTCCACTCCCCTTCAACCTTAG

AACGGCATTCACAGCAAGTTGGgAGAACATCTCGCGGTAGTGCACAAGAATCTTTGAACT

AAGGGTGGTTCGAGCGATGTTCAGCAGATCTTCGCGAAACCTTTCTGCATTGCCACTGTT

GTCCTTGGCGGACATCTCAAGAGCCTCCTCGGCGCTGCGTAAAGCTTTACGCCATCCCTG

AATGATTGTCTGCGGATGAATCTTCTGGGCTATCAATTTCTCGGCATTCCTCAATAACTC

ACTAGCCAAAACAGCAACACTAGTTGTCCCATCCCCAACTTCATCATCCTGAaCTTTGGC

TATATCAACTAATACTTTGCCAGCAGGATTATCAACCCCaaTGgATCTAAgAaTtGTGGC

ACCaTCATTTGTAACcTGTAtGcTCTGGTCACGTCCCATGGAAACTAGAATTttATCCAT

TCCTTTTtGGTtCCAAGGGTAGTCTTGACTAAGTcgCCAATGGCTATTGCACCAaCAAAA

GAAGACAATCTAGCAGTCTCAGCTCTCTCTTCCTcTACACCTtGTTCAAGTACGTTTACG

GGGTTGAATGCGCCAGCCATTTTttgCAACGTTTAAATTGGACGAGCTGCTCCCTTCCTA

CCGATCCAAAGAAAGTTCGAGAAGCCCCGCGTACTCTGCGTTGATACCACTGCTT

>contig00302 length=225 numreads=4

gTAGTAGaaGGCCtGAAAAtttCGCATGTACGTATCATGGCTAATAGCACAGTCTTtCGA

AGGTTCTTACTTTGCTGTTAAaTtGgTTtCTTTGCaGACCtGATTAGTGgAACGAAGACT

CTTTACAaGGAaGGAAgATGgCTTGGAAGCaCTtATtGTAAAATATtAATACGGTTACGC

GGGTTTAAGattCACGTTGCaCAaTACAGTTTAAaaTATtGGTTT

>contig00303 length=641 numreads=19

GCACcTGACAAATCTCCGGTGCAGAAACTtGGTCTTCTCGCCACTGCAGCGGAGAATAGT

CGACAACTAAGTCCAGATTCTGCTTGGTCCGATGGTAGAAGAAATTCATCTTCGACTTCA

ATCTGGTCCGCTCGTCCCGCTTGGGGATATCGGGAAGAAATTtCTCCAGCaCTCGAAACA

AaTTGCAGATCATCGAGCGAACGAGAAAATGGTATGACATCTTCGaagCGATCTGCTCGt

GGATGTCTTTCAGAAaGTTCcACTTGATGTCACCTACTGTTTGTTCGACTAaCTCTATGG

CTCGCAAATAGCAAGTGgCTTTCCcAGCTACaGAGGAGATTCCGCcGTGTTCACaCGAAT

AGATGTGGTTGGcGAACCTACCGACGTCTTGTTCAACTTGCCTCCACTCCGTGTAGCTTA

TTTGATAGCCTGAGATCTTTTCGGTTGATGCATaCACcAgTGGCCAAACGGACGACTCTC

GGTCTTTTGTGAAGAAATCTGCTGcAACACTTGtACATTGTCctCTCTTGTGCCGTTAAT

AAATGCGTCCATGGATTTCTTCACCACTTCTCGTAGCTGgAGGCCATtCCCGGAGAAGAC

CAGGACTCCTGCTAAAAGGGCAATCTGAAGGAGGCCCATCT

>contig00304 length=278 numreads=3

tgctacattcacagcgactgagcgaaccTCCCCttCggccctGGATCATTTCAGAAACTT

CTGGTTGTATCAGGACTGGGCATTGCACATGTATggCAGGTTGTGGGgAAACATGTACTC

ATGTGGCAGcATtgCtaTTTTAtGTGGAGtCaGTTATAAAGTTtCGTGACTCAAAAACTG

TAACAGATGaaaaGGCTTATTGGAAGGTACCATCTGTCTACAAAGGCCGGTCATATAAAA

AGCTGGGACAGATTGACTTTTCATCACCAAAGTCATTg

>contig00305 length=679 numreads=13

AaGCAGTGGTATCAACGCAGAGTCTTGAAGCAGgTTGGAAACTTAGCATCTTCAATTATT

TCTTCATCAGAAACTACACAgTTATCCAATGCaCACAAACTCCAAATAGCATTAACAACA

CAATGCCTGTAGTTTTTTtcAAaCTCACTGGAGTGtCAAATaGGGTTAGAcTGTGAAGTT

ttATGGCCGATGACAAACTTTTTACATCTtCCAcTCTTgATaCACAATTATCATGGAGAT

AGACTGTTTGTaacttcaagaaattactccaaaatgattgaccaggcagcttgtctagct

gatttgagtgtaaatccaaacaaactagattttgacaaaacttcaaagcttCAATGTTAC

AAATGAAATTGCAGCTAAGGTTACAGATTTGCAGCTGTGGACAgcAATTAACCATAGATA

TGTCTGTGACttgcttacaaggtaagcacatcaccatagcagcactcggaggaaatgagt

ctttgtcatctttggctgctccatAACAACAAAATGTcTACaCAaGtAATTGGATTGCCA

AGTGATtcGCCTTCAGCaTTAGACATGACATCATGGGCACCaTAAACCTGCGGCTTTGAC

CTTTCTACAGGCCCTGGATTTTTGCAAACTCGTCGGTACTTTGTGGCTCCCATTTCCATA

CTTCAATGTTGTTCTAGTT

>contig00306 length=184 numreads=2

cAGAATGTTTACAGTGGCATTGATCAGCGTACTACTGACTGTAGCTCAGGCGTACTATAG

TCGCCACGATGACCCTGTGGTTAAAATCCGAAAGTTTGGTTTCGACCAAGACCATACGAC

CTACTCTCACGAGCTTGTGGCGCCTTGGAAGCGACCGTTCAAACCCggTATTGCGACTCC

Tggg

>contig00307 length=172 numreads=1

ttcgcagttacctagcacctgctgccttggcattggagcatgaaatgggccttgcagtgg

tcaggagtttttgctgtgagcatagtgctggcaagctttcacccaatcctcataaacttg

gcaaagaatgatggaaagattcctttttagttctgcatctgtgctttactaa

>contig00308 length=171 numreads=1

gttgatcgtatcatggatgtaccaaaaatgaaactggtaaatggcattcgagtaccatta

tgttttccatcagagaatgtgtgcagcgctcttaattacaagccagaagatagtgacacc

tttttggtaacctacccgaaaagcggcaccacttggatgatgtacattttg

>contig00309 length=127 numreads=2

aagcagtggtatcaacgcagagttactttttttttttttttttgtAccAGCACGCAAAAT

TCACTTATTTACTTAGACAAGTTTCCATAtactgaagaactcaagcaaagaactgttcca

tcttcac

>contig00310 length=588 numreads=7

CGcAGAGTttCCTTtGTtCATTAGCAACCTCCAACTCGTCACcTtGGGTCGATCACACTC

CTGCGCCTTCTGCTTGTGGTTTTCAGGTGAGTCTTGGCCTTGGTGCTCGACTTGGCATGC

TGgATGAACGGAGCATTAGgCGTTtGCGAGATCTTATtGATAGTGTGAGtCAAAACtGTC

CCCTTCTTGTCACCGTCTTtCCTTTCCTTCGACTTCGTccaagtctttatcttcgctgtc

accactgtctgcgtcctcagtatcgtttcggcgactgcccttcctGTGCTTTTTAAGTTT

TCTGTgaCTTttttCTTTCTTCTTCTCTTCCTCTGCCTtCTCCTCTTCCGCAGCCTCcTC

TTCTTCCTCCTCcTCAGCTATCGGAGGTGGCAGCGgTgCTGGAggCaCGGgTGCCGGtGg

TAGCGgATCAGCGGGAAGcTGGGGTTGTGgTGGAGGTGGAGCTtgtGGAGGTAGAGGTAG

tAGaGATGGATCCTGAATTGGTGGTGGATAAGGCGGccGCGGAAAGCCGATAggCTGTTG

TGGATACGGGTAGGGTGGCACTggtggtagcgctaccggccctggagc

>contig00311 length=248 numreads=12

aaGACGATGTGGCGGGTTTGATCAAACAGCTCGTTGaGATTTTGCACGACATGCATCAAA

ACAACTGCGTCCACCTTGATCTTcGTCcTACCAACATCCgCATtGCAGgTGCTAAGgAAG

TCAAGCTTGTGgATTACAATAGTaGCCGCTACATTGCCAACAAGAAAGTGGGCGAGGTCG

TCGATGTCATTGGTGACACGGAaTtCTGCGCTCCAGAAATGTTGAAGTTTGAGCCAGTCT

CCGGGGGA

>contig00312 length=178 numreads=2

aggggtctgatatgtggtctgttggtgtcctcacttacattctactgagtgggatttctc

cgttctactatgaggacgaacagcaagtattggcaagtGTGGAGAAGGTTAAGTGGAGCT

TTGATGCTAACGCCTTCGCTCAAGTCACAAGCGAGGCTAAGGAATTCATCAAGGCTTg

>contig00313 length=1187 numreads=59

GGGCCCCATGATGCTTTAGATGGTCAGACTTATATGGCGAGTCGTAATaTAGTCATTCAG

GAATCTGATGAGATTGACCTCACTAACATATCAAATACCAATATTTCTATAGTGGCAGTG

TCCTCAGAaTCAGAAGAAGAaGAAgACCGATtCAGTAGATCTGAATTTagtttgaaGAAA

AAGTCATCAGTTCCATTTCTCGACTTAGCTGACACaCCAAGTCcACCTAAAaGGAaGCGT

AATGACCTCAAAGGAAGCCTCAAGTCTGCGATTCATCGTAGCCGAAATCCAGATCAAGAA

GAAGGAGAAATTTtGTCTAGTCCaGAGTTAGATGCTGCcGAGATTGAGCTCATCACAACT

GGAACACTGTCATCTAGTGAAGATTCTGAAGATGATGACGTGCTGGTGAAGCATGATGTT

CTGACTTCCGACgTTTCTAGCCATGAATCTATCAGCGATGAGGAAGGCCCCATtGATGTA

AACCCAGTTATGGAGGCTAGTATAAACCTGATAACTCCCGATGAGCCGTTACCGTTATCG

AAGGATACTGTGCTAGAAACCGTAGTAACTGATCATGACTTGTATAGCGATTTGCAGTTT

tCcGAGAAGCCCCTACCTAAGATCGATATACCGTACAAGGTGCCTGTGCGTGTGCCGGCA

TCTGGTACGGCCgaCGAAGAAGATGATGTGGACGTTGATCATTTGgAGATCTCTGCGCCC

CAGCAGTTGGAGGTGAAAaGGACGTCTGCTAAAGATCGTCCTGCCGATGtcAAGGggcTt

GTCGATAGTCctCGTGGCAGTCCCCTGTATGAGAAAGGCGACAGTAGAAAGCAAAGCCCT

AgagACGAGTCGAAGAGTAAGCGCCCATACGACGTTCAAGACGAGAGGTCAAGAGAGAAT

GATCTAGAGGCCGTAAAGAAGGTCAGAGATCTGGAGCGAAGAAaGCAGGAGGGTGAGGAC

CTCCGGAAACGAATTATGATAAAAaTGGAGGAGgAAAaTTtCAGGgAAAAGGCACGGGAA

AAGgaGAGGGAGAAGGAAAGAGAAAAAAaGGTGGAAAGAGAACGGcAAGAACGGgATCGa

GaCCGGgAGCGCTtAAAAGAAAGAGATAAACAAAGaCAAAAGGAaCGGgAaGTAAgAGAG

CGAGaTcGTgAACGGCAAAAGGATAAAGACCGGGAGCATGAAACTAT

>contig00314 length=1492 numreads=77

AAGCAGTGGTATCAACGCAGAGTACGCGGGGCTTTcTGTTCCTGCAGGCTTGACAaGGAG

GgTTCATATTAAGCATAAGCAGCCATGGTTtCTCCTCCGGGTCCACAGAGTGGCAAGCCT

AGTTTtAAAGTCGCTGATCTGTCATTGGCTGACTTAGGGGACCGGTACATCAAGCTTGCT

GAGAATGAAATGCCTGGCTTGATGATGATGCGAACGCGGTATGgACCCCTTAAGCCATTA

AAAGGTGCCAGAATTGCCGGTTGCctGCACaTGACCATTCAaaCAGCtGTtCTGAtaGAC

ActcTAAAGGAACTGGGTGCTgAaGTtCAATGgTCCTCTTGCAaCATcTTTTCCACCCAG

GATCATGCtGCTGCTGCCGTGGCAAGGAAGGGTATATCTGTGTATGCCtGGAAaGGTGAA

ACCGATGAAGAATATATTtGGTGCATAGAGCAAACTCTAATATTTCCTGACGGTCAGCCT

TTAAACATGATCCTTGATGATGGTGGAGATTTGACAAATCTTGTGCATGAAAAGTACCCT

GATTTGCTTCCAGGTATAAAaGGTCtgtCGGAAGAAACAACAACTGGTGTTCATAATCTG

TACAAAATGTTTAGTAAAGGAGCCCTCAAAGTCCCGGCAATAAATGTGAATGATTCTGTC

ACAAAAAGTAAATTTGACAATCTCTATGGTTGCCGCGAATCGCTAGTGGATGGAATCAAG

CGAGCAACTGATGTTATGATAGCTGGCAAGGTTTGCTGTGTGGCTGGTTTTGGAGATGTT

GGAAAGGGATGTGCTAATGCTTTGCATGCTTTCGGCGCTCGCGTCATTGTAACAGAAATA

GATCCGATTAACGCCCTACAaGCTGCCATGGCCGGTTATGAAGTAAGCACCGTTGATGAT

GCCGTTTCCAAGTCGCGTATAATTGTAACTGCAACTGGTTGCAAAGGAATCATACGCGCT

GAaCATTTCTCACAGATGCCGGAAGATACCATCATTTGCAATATTGGCCACTTCGACTGT

GAGATTGATGTTGCCTGGCTTGAAGGTAATTGTCAGAAGGAAAGTGTTAAGCCTCAGGTC

GATCGTTACACCCTGCCGAATGGACGTCACATCATCCTCTTAGCGGAGGGTCGCTTGGTT

AATCTTGGCTGCGCTCATGGTCATCCCAGTTTCGTGATGAGCAATTCGTTTACGAACCAA

GTGCTAGCTCAGATTGAATTGTGGCAAAGTCATGGTCATTATCAAGTTGGCGTTCATATG

CTGCCGAAAAAGCTCGATGAAGAAGTGGCGCGACTaCACTTGGATCATCTTGGTGTGAAG

CTGACGAAGTTGTCAGCGGAGCAGTCGGAGTAtCTtGGGGTGCCCGTGGAAGGTCCCTTT

AAGCCTGAATTTTATCgTtATTGAGCttCTACACTTGCGCTGGTTCTCCTCGTTGCCCGG

TGAAACTTGtaCTGAATTTTCAGTTATCGTAGAATTTTggTTAGAGATTgaa

>contig00315 length=473 numreads=5

gacaagctaaagttacgaaacagacgccggagatccgcaaacacattttaaaaaatggca

actatgaaagcagcagccttttcacacatcggcggccctgATGTTTTGCAGTATATCGAT

GTGCCCAAGCCGGAACCTGGCCAAGgCgaGgTtCtGgTtAAAATtCAAGCTACtGGTATA

AACGcTgtGGAGGTTttCAtCCGAGGtGGAaTGATACCGCTAaCGgTTTTTCCtCACATT

CTTGGAATGgAGgCTGCTGGTGAaGTaGAAAAGTtGGGGGCAGGGgTTACGAAATTcAAA

gTGGGGgACCgTGTTTGGCTGAACTTCCcTGACTTTATTCCGCATGGAACATAaCGCTGA

ATACACTGCCGCTCCAGTAAAGCATGTACATCATTTGCCAGCACGCTATAGctatgaaga

aggcgccgcccttggagttgcatatatggctgctgaaagagccctggttgtag

>contig00316 length=171 numreads=2

aagcagtggtatcaacgcagagtacgtcgggaaatcggaaatgttttacctgtgggtGTA

ATGCCAGAAGGAACTATAGgTCTGTAACTTAGAAGAGAAGTCAGGAGATCGAGGCAAGTT

GGCCCGAGCTTCTGGAGATTATGCTACAGTGGTATCTCATAACAAggaaac

>contig00317 length=196 numreads=3

TTGAGAGACTAAGCATCATATGCAATGCATGgTAAGTTgAGGcTGgCcTTGAATCGACAC

AATGTGAAGgTTTTTTAtGTTGTTtCtGGTCTTATTttGCTTGGACAATATCGGATGTAT

CTGAAAATAAaGGATCAAAAGTTACGACCCATGACTATTGAAGATATAAATGAGCATGGT

TTGTGGAGGCGAggtt

>contig00318 length=236 numreads=2

cAGTTCATTAAGCTTTTGCTGCTTTTCACGCTGAAACGCTTCCAGGTCAGTACTCGCTGC

TTTTAGCGCAGACTCGATGATCTTCACtttttCATGAGCGCGTCCAAGTCTTTCTTGGCG

atgtcatggagtttctttccttccgccgttgcttcttctaaatccagcctcttctcgcga

agctggcatacttggtacaaacaaagcaggctcgcatccagatggacataagctat

>contig00319 length=232 numreads=4

ttATCGTGTCAaCAAACCGGTCATCCTAAaTTGCGTTGCCaTgTGGTcTTcGCCGGCACG

AAAATAaTtATTTtGGGAACAGTAGTGCATATGGTAACAGTAGTACATAAGTTAGCATAG

CAGCTACAAAACCTGACTTCCAAGTGTTCATACCACAACTCATCATCCATTATTTGCATC

CAAAATATAATCGCCACAGACCTCTAACGTCATGCAGACCAGCATAATAAGa

>contig00320 length=850 numreads=50

ttGCaCTTaCAAAAGAACCtGTTaGAAATCAATgAGGCGAcTGCTTTAAGtGAACAATCT

GAAAAAGATGGCATaGAAAAAaCaGgACCAGTGCATGAATGGAAGCCAGGAGATAGATGT

AGTGCGATCCGCAAGCTTGACAAACagTATCACAATGCAGTAATCGATATGATTGCAGAC

GACCGACAaTCTTGTACAaTCAAGTTTGATAGCAGTGGATCcgTTGATGTTGTTAAGCTT

GCGTCTTTAAAGCCTGTTGAAGGGTTCACATCttcTTCAATCGCGTCATCGCTAGGCACG

GGGAAGCACAGACTCACCAGGGAGGAACTAGagCGAAAGCGTGAAATAAAGAAAAAGAAG

CTGTTGAaGAAAAAaCAagCGAATGAAGGATTTCGAAGAGGCCAGAGAAGCAGGAAAACA

AAGATGGCAGAGTTTTTACAAgagCGGTTTAAAGGGAAGGTCGAAGATCAAAGGCTTGAA

TAAAAAGAGTATATTTGCGTCTAGTGTAGATGGGAAAGGAAaGaTTGGCATCGGAACATG

TGGCAGCTCCGGAAAGGGGATGACGCAATTTCCCAGTCCAGCTCAATATATGTACAAGAA

GTAGTTGACGCTTGTCTCCCAAaCTCTCCTTTATGGCCTTCCATGCCGCTTTCTTTGCCA

TGGAATCAGCGGTAGCCATGTTGCCGCAATCCCCGGTGTGATCGTGTTTTGTGTCGTCTT

CCAGGtGAtcGGTGACCTCCATCAGCCTTTAGTAACCTATCATTGGACTAGCACGTCATA

CTCCTATACCAACAAAGTGGggTtGTTttGTAaTTTtATATATTGTTAATAAAcTGTTtA

CGTCGgAacc

>contig00321 length=647 numreads=16

ttCGGGGCGCTCACAGGCTGGGATTCgTATttCATCACGCCAAGCaGGATTACtCGGTTC

TGATGcTCTGgCTTGTGGAAAATGTGCCGAACAAGGTtCCGGCTtAcGCtAATCATACCG

TTGGCgTAGCaGGAAtCtGCGTTCATGATAACCgcGCTCTCGTTGTACAGGACAAGGACA

AGCTGCCAATGTGGTCCTTGCCAGGcGGTtATGCTGAGctAAAGGAATCCATCGGTGAAA

CCGCTGTACGAGAATGTTGGGAAGAAACCGGGGTGCTTGCAGAATTTCAGTCTGTTTTGA

CTCTGCGTCACATGCACGATTGCGATTTTGGCCAGTCAAACTtGCtCTTCGTTtGTCGGC

TGCGACCTGTAACGACAAACATTCgTCcGTGCCAAGACGAGATTTATCGCGCTgAGTGgC

tAgATCTGGACACCCTTGCGCAGAGTGCCGAaGgAACGCCCCTAaCAAaGCTTGTTTGTA

aGCTGGTaTTGCAAGGTCGAAAAtATGGATTTGACACAGTAGACATAAGTTCTTCGTCAa

TGGACAAGTGGTGgTCTCAACATAAGAGCGAAcAGTTTTTTGTATACCACAGACCTTTAA

TGAGTACTTTATAGTGATGTTCAtGGGAAGAGCTTGGAAGaaataaa

>contig00322 length=943 numreads=16

AAGCAGtGGTATCAACGcAGAGTACGCGGGggcgCTTGgCGGGCAaGGAaCATtCTGGAA

CTTCTTCCtGCaCTGtcTtCGgTTtAAcTCGACTTCCATCATGCCTACAcATCctCCCGT

TctCTGGGCACAAAGAAAAGaTtATATaCTAgTGAGCATTCAAGTTGATGACATACGCGA

TGAAAaGATACAATTAGAAGGAAaCAaGCTAGTTATGAGCTGTCGAGGTGGAAAGGAaGG

CGTAGAGTATGCTGTTGACCTTGAGTTTTACTCCGAGCTTGTGCCCCaGGAGTCCTtACA

AaGGAAAGGAGgTAGAGAATTCTACTTTCAATTTAAGAAGAAGGATACAACTGCaTCCTG

GTGgCCcAGACTCCTCAAaGACAAAATtAAGcACCCGTACGTCAAAGTGGAcTTCAGTCG

TTGGAAGGAAGAAGACGATTCAGATCAAGAGTTCGGTCGGTATGATGACGCTAATCTGGA

GGACATGATGAAAAATATGGGCGGTGGCGGTGACTTTGACCCTGGGgATATTCCAGATTC

CgAaGaTAGTGATgACGAAGACATTCCTGATCTTgAAGAGGACGTTTCAGgAAaGGTTCC

tgCACCCGCATCGACAGAGGCCGcATCGGAATCCAAGTcGCCTgaaccataatccccaag

cggaaaatatgcaacgtctgaactaattagctcaggattttcgagTTtCTGTTCTCAAGG

CTGGTCGTAATTCAaTGGGAGCcTTTtGTGGCGCgAAAaTGTGCTCgCTTttCAGCAGCa

CTTCTCTATTGTTTGGACGTGTTCCTTGTCGAGTACTCGACATTTTGTTGTAGCAATCCT

GTTTTGCCTGCAAGCACGTGCGTTCTGATTAGCAGcAAGCGAAAaTATCGGAGGCTTGGT

TTAGTAAGGATGGAGgATTAGAAGTTGatAACaaaaaaaaaaa

>contig00323 length=652 numreads=7

cTTAGAGGACGAAAAAAGCACAGTTGATGTTGCCGAAGAAGGCTTGGTCTGCTTGGCCGA

TCAGTTCCGAGCAAAGTCTGCTTGTATCCTCCGACTGGCGAAAGAACTGACGGAAGAACT

AGGCCAGGAGTCGCGGTTGCTTGTGAAAACTCTGTCGgTGTTGTGCGCTGCTACTtCCTA

CCGTGAAaTATACCCaGGCCTTTGTGGCTGTGTGGCTCTGCTGGAGGTCGCGATCGatct

tttacatggcgtatcttcggaaaccttgggcccattcttctcgcgcccGgCTgTTCcGGA

AGGAAACGTGTCGCCAAACATTGCTACTGGGTTtAAaCGCGATCTcATAAGgTTGATTGG

CAACATgTCGtACcgCAACCGTGCTAACCAAGAcAAGGTGCGTGAGCGAAACGGGATCTT

ACCGATTTTAAACTCCTGCAACaTCGACGACTGTAATCCGtACATCTTGCAATGGGCTAT

TTTTGCAGTAAGAAATCTATGCgAAGGgAATCTGGAgAATCGAcATGTCTTGGCGTCGTT

GGAAGGgCAGGgActtgcttgtaacgggtttgtaagccagtcaaatatctcggtgaggat

ggagaatggcaaagttcgtgttctacccaaagaaagatagtatttgttcaag

>contig00324 length=180 numreads=3

CTAGTTTCGATAAAGTTAGAGAAAAGCTACaCAACCAAAACTTcATTTCAGGCcAAACGA

TGATGCGaGAAAGATcAtCATtAGGTCCACAACTAgAAAaGCCAGaCAGaGCtGATGCAT

GGAAAgagttgtgacactatcgcatatcaagaaacagaaataaaagcaatgcatccctac

>contig00325 length=118 numreads=1

tgcttgtggaagccagcaacagttcagcttttgtaagattccattctcgcacttacacca

aatagtttgtgaattatatagcttttttttctatccttgctgattttgaagaccctag

>contig00326 length=485 numreads=6

caataacatggaaaaatacttctagtaacaaaacccgcggaaataatagcggctcAACTT

TAAATACACTTTTTaaCcTATTTCACAAAATAAGAGaaaCAAGAATTATCAAACGAGACA

ACGATAAGCGGGATTCCGCCTAAGCCACATAACGTaaaataaagcaagcgtttcagttgc

ctggatactggctcaaaaagaaagcccttgccatgggcgaacacctgcaatcacatagct

gcacgcacttaaGAAAAAGAGTCACGCGCATGATCGTACTTGATCAGCTTGAAGTCGATA

TGCAAGGAGTCAAGGAAAACaGGCATAGAATTCTTGTTTTCtGCGTCTAGTAGCTCACGC

aGCtCcACAaCtGCCTGCTgCGcTcGcccctcTCCACTTGGCGATCAGAGACGGCAAGTT

CGTCAGCTCATgCTTTTTCCGATACATGCGGATAAGCTTGAgCTTtCCTTAACTTGTCTT

CTTTg

>contig00327 length=142 numreads=1

taaggcataaagtttggtgctttccagctgatagaagtattttgttgagctaatgatacc

tattcgctatggaccctgtggataaagattcttctgcattgcaaaatcttttatctcctt

ctgggggtgtccagcagttctt

>contig00328 length=165 numreads=1

tataggcatgtcagttcagcgaaatggaagaggatcaacttcttgacccagttgagccag

tgccaactaaagttgtggcttcatgtataggaagtgataatcaacgttactacaaaatcc

aatggagcgagagctgggcatctgaagatcttctagctcttaaat

>contig00329 length=1160 numreads=59

gagagcttaagTCTAGAGCTTACCTTAGTCAGTGAGTTTCgTCGCAAAaGTCGTCATGAG

AACTAAGAAAAGAAAGCATTCTAAGAAATATTACATCGGAGGAAaGAaGGCAaGgAAGgA

TTGCTTGGCGGCTGGCATGAATGGATTCTTCATCACTTGCATGTCTCGCGAGCGAGACTC

TgTGCTTgAGGCTTACaGGATACTTAACGAGCATGCTGATAAGCTGTATGGCCCAGAGTG

TTTGCCTTCGCAACCAAGCAACGATGATAGTGTTGATGACGATGTGGAGGCTAGCATCAG

GAAGGAAGTAGCGGGgATTAAAGAAaGCAGTCTCGTTGAACGGCGGTTTTTGgCGGTTTC

GACTAGAGTCGGTAATAGCTTGTTCATACGAACGACGTTAGAGGATCCCACTGCTCTAGC

TAATAGTATCTTCGAAAAGACGGAGGAAGGTGTTCAGAATGTTCGACACTGCTACCGGTT

CGCACCAGTGCTAGCGACCTGTTATGCTTCGACGGATGAGATCATGAAGTGTGCGAGCAA

GATCATCCCCAAGCATTTTCACGGAGAGAACGTCGCTCAATTGTCGTTTTGTATCACATG

GAGAGTGCGTTGTAACGATACGGTGAAGCAGGATGATGTCGTGTTGCCATTGAGCGAGCT

AATTGAGAATGGTGGAAGCGTCAAACACCGTGTCGAGTATCTTGATCCCGACTTGGTGCT

CAACCTTGATGTTTTAGCGAACGTCTGCTGTATCGGGGTCTTGCGAAACTTCAAGCGTTT

CATGAAGTATAACCTGTTTGCTGTAGCGTCTAAGAAAGCTGGTGATAGTTCTGCTAAAAC

CGGACCCGACGAGAAGGATATCACGCCGGTGGACCAAGCAGGAGCTTCGGTAGACTCAGT

GGGTTCTGATGAAACTAGTGACAGCCCCAATAGTCGGATTAATGATTGTCCAAAAAaCCG

ATGTTGGTCACAAGGCTGAATTGGAAGgCGATGATGTCAAaGACATGCCGTGTCAgagTG

ACgCATTGAATTGACGATCTATCGGTTATAGGTTGTGGCGAACTCGGTTGCGCTTTtgTA

CGTCTAGCTtCACCATTTATCTCAAGTATTTTCGTGCCTAGTCATGGGAAGATATCATAT

TtCGAaTtaaaaaaaaaaaa

>contig00330 length=802 numreads=11

AGAACCAGAATTTtGTTATGAGAGATAAATTCAGTGTTGAAAGTTTGAAAACGTTTGCTT

CGGAATTCCTTGAAGGGAAGCTGAAGCCATATATCAAGTCTGAGGCAGTCCCTGAATCTA

ATGATGGACCTGTTAAGgTtGTTGTTGGTACAaCTTTCGACCAAATCGTTAATGACGACT

CTAAAgATGTGCTCATTGAATtCTATgCGCCGTGGTGTGGTCATTGCaaGaCTTtGGAAC

CGAAGTACGCGGAaTTGGGAGAAaaGCTTAAAGACGTGAAAGACGTAGTTATTGCTAAAA

TGGACGCAACTGCAAaTGATGTACCTCCGCCGTTTTCAGTTTCGGGATTTCCAACACTAT

ACTGGgCACCTATGGGTCGAAAAaGCAATCCGAaGAaGTACGAAGGAGGACGGGAGGTGA

AAGATTTCCTAGACTTCATCAAGCGAGAAGCTACTAATCCGATtGAACTACCTAgTgAAG

GGAAGAAGAAGAaGAAGCAGGAAGAACTGTAGACTGATAGgATTtATAAATtGgaCATTT

TGTACTGTTAGCCGGTTGCTTCCTTGTgttctatttATTTTTTtCCccttttCTAcAGGG

TTtGTAAGCcGaTTTTGttAAaacTTtaTTTAaCGTGGAAaCTCGTTGAATCCGaTCGGG

AATGGACTTTTTTCAATTCTTTCATGTGTGTCGTAACcATAGCTTGACTtGtCTCATCGC

TGGtGaTGgTtCATACTTTAGTtGGctGTaGTGTAGggAATttCttttttttttGCAAAA

GAAAACAACCtGCTTATCTaaa

>contig00331 length=919 numreads=10

cggaatgagaatggggcaTCCATTTccccaCTTTTAAGTtGATATGTCTGTTTCTGTGaG

gATGGGGGACGgTATCGATGCGCTTAGGTGATTTTTCTGGgAaTGCTGCCTCGAAACTTG

CCGCCTCCTCCCGTGCCGCcGCAGATGGCTCTTCcTCCTATGCcACGTTtAaTGgTATCG

GCACCAATGCCACCATTACTGCCAaTgATGCCGACAGCGTCTACTTCGGgTGGTAATAAt

CcTGCACTCGTTATAAAGCCaCccaCGGTaCCGTTAGATAGCTACGAGCCCCTTAGTGTT

GCACCTTCCGATgCGACATTTGGAGAaGCtGgAGATGAtgTTACTGAAgAAAGCAAAAAA

GCTGgAAAAGGCAAGAAAAAGTTGCTCCGGATCGCCGGCGGGCAGGTGTGGGAGGATTCC

aaCCTCGTAGACTGGGATCCAAACGAtttccggctatttattggtgacattggcaatgaa

gttacggaggaagccttgttgcgagccttcagtagatacccATCCTTGCAAAaGaTAAAA

gTTGTTCGtgATAAGCGTACGAAGAAGACCAAGGGTTTTGgATTCatcagttttaaagac

cctcaagactatctaaaggctatgagagaaatgaacgggaaatacgttggtaaccgaccc

ataaagcttcgcaagagtacctggagggaccgtgatattGGCGTCGCTAGGCGGAAaGAA

AAGGAAAAGAAGAAGCTGGGTATCAGATGAGCAGTTGTTGGAATCGTACTTTGGCGACTG

TTTTTcATTCGCAGccatctactctccgtacgtttgtgcagttaccagcgttcgggtgtg

ctaggaactgtgtgactaccatagcgcttgtacaattgtattccattatttgccggagaa

gagttcgtaggtggacaaa

>contig00332 length=153 numreads=3

AGAGCTGgtCGGACGcccGAAAaGACAACCTGaCGAATCAgCAACACAAACAtCTCTaCA

ATATGAAaCAAAGCaGCGAGCGAaGTaCGTCATTTGCTTTtCTCATCGATAGGTCAAAAA

CCTcaGTTCCGATAGGCgATAACaCGCAGACGc

>contig00333 length=781 numreads=12

cTTTGCTTTAAtGGGgAGGATTCACAGTTTCTTGTTcTtGCtGATCAaGCTGGAGATATG

TTTGCTTACAGCATaTtCGACGGGATTTGTGAACGAGAAaTGTTGTTGCCcAGCATTCCA

AACAAATCACTGCCTTACAAGtGGTTAGTTGGTAAaGGTGATCTAGTCGCTGTTTTCGAT

GCTCAGAACCGTGGACACGTCATTGATGTCAGTGGCTGCGCATACAGATGCTGTATCCCT

TCgTTAAGCAGCCACgTAAGTttACGCTACTTTCCGACCAAaTGCTACcTCGATTATGAT

GTTggCGGCTGCCCATGATATGTATGAGTTCGATTACACCGAAGAGACTTTCCTTCCGTG

GTGCTTGTCGGTTAACGGTTCgcgtttacttGTTAGAGCcGCACCTATGaGTGACCccAT

TTCTAAAATTGTCTTTAATCcTTGCAATCCAGAAGAGATGTTTTtGGTGAaCACTAAGAA

GATTGGAAaGATGATGTATGGGGCAAATATGGAAGATGTTGACCCTGATCAAAAGTATCT

TCAaGCcAAGTGGGAATCTGATTCAATGGGaCcGGTAAAGTTTTCCCaTAGgTaCTCGGa

TATATTGTTTTTTGaCTtCaCGtCCGCAGgTGaTaTGGTAGTTGTTGAGCGATTGATGGg

AGGATATCCTAAGGAAGCTGCCtcccagactgaaagtcaagcagtttggatactaagttg

tggtaaagtgccgtgcgtgtggcaagccaaccttcgtttcgtggattgtatacttgcctt

g

>contig00334 length=135 numreads=2

AAGCAGTGGTATCAACGCAGAGTCGACGAAATATACTAATGTTTAGAAATAATCAACACC

CTGTGACGCAACTGCAGCGAAACTCTTGTGATAATACCAAATAAaCaaagcttaaagtaa

aaaactgccctctca

>contig00335 length=355 numreads=11

cGAGCAGCGTCAAAAAttGCAGAAACGGTTGCGAAAaTGGCGTCGGCGGGTTTGTCGGCA

tCAaCACGCGAATGCAAATTTCgCTtCCGATAATAATTGATCAATGGCTCCGTcTGTTtG

TGgTAGgCGGTTAGTCGCTTGCGAAGaGTTtCTTCATTGTCGTCTGAGCGACgAACCAaC

GGTTCACCAGTCACATCATCGGTCATCGCAACTTtAGgTGGGTTGAATGTCGTATGGTAT

GAGCGTCCACTTGCCAAGTGAAACAGTcTCCCAGTGATTCGCcTAACCAGCAAGTCATCG

TCAATACCGAACTCGATGACTCGATCTAAACTGGTAGCCCGCTTTTCCAGCAGCT

>contig00336 length=154 numreads=39

TGGTACTGTTAATGCTCGGTATTGTTGCGATCCACGGCTCGTGAGAGGAGCAAAGCCCGG

GATAAAGAAGTGGAGACGCGGGAACGGTACCATGTTTACGGCCAGTTTTCTTAGGTCAGC

ATTTAACTGACCAGGAAACCTGAGACAGGTGGTC

>contig00337 length=565 numreads=15

TGTGCTCCATTGAAgCATATCCGTAGTGCTCAACGATGTCGCTTTTCTCAACCGCGTAGT

GTATGTTATGCCGTTTGTCCTTGTTCGCCATGTTTACGAGCTCGACGTCGTTCTCAGCCG

TCCACCACGAaGGCAGAACACCACGCCGATGCGCACCGTTGAGGAACTTTcGAAAaTcAA

agTtAGGgTCcTCTtGAGCATAGAGACCACGAAGGTCGCCTtGGAAAaCATATTCATCTT

CTATCCGAaGCCTGTAGGCATCAaTCAGTCTCTCATAGCATcTtCTTTTATTtAaCCCAC

TGCTCCATCcTCTTTtATCaGAaGGATCCCCAAAATTTTCCaaaaaCCTCTccGGTGTGG

TAaTAGAAaTGTTagCGAAgTCATTTAtCACATTTGAaTTTCTCATCATATTTtCtGTGG

TCACGTTCTTGCAtGTTTGACGATGAGCCTTCCAGTCTGCTTTTTGGCAATGCCTGGAAC

AATATTGCACACTTTTACAAGATGCACACcTTTTtGtAGCATCTTCCTTCTTGCATGTGT

TGCATGTTCCAACCATTtGTGTGGC

>contig00338 length=855 numreads=49

GGGACAGTGAACTAACTACTGCCAAGATGAACATGCGTGTGTATGCTGTTCTAATTCTGG

GCGCCTGCCTCCTGGTACTCAAGGTGTCTGCCAAAGATACCGAGGAGGCAGCCCcTGAGG

ATGACCACAAGGAAGAAGAAAAGGAaGTtAAGGAAAAGAGTGCGTCCGAGGCTGCGGAAa

CCCcTGCCACCGAGCCTGAGAGCCCGAAGGAAGAATCTAGCGCTAAGGCCAAAGATGTCA

CTGAGGAaCCAAGCGACGCGCCCGcTGATGaGaGCAAAGGTGAAGAAAaGAAGAGCGATG

TTGCTGATAAGAAGGATGAAGATGAAGAGGAGGAATCCGAAGATTCTCTCCCTTtCGCCT

GCAACCTGAAGTTCAAGCGAGTCGGTTGCTACGCTGACAAAGGAAACAAAGAAAGGCCAC

TGCGCtCTTTCATTATGAATGATGCTGACATGGGCACGATTTCGAAGAAAGGAAAACtCC

cAGAAGGTGACAAGTTCAACATGGAACTACCCAAGTTTGCCTGCAAATGCGCCAATGAGG

CTATAAGTTCCGGAAGCGCAGTGTTTGGCTTGCAGAACCTAGCCGAATGCTGGTCAGGAC

CTGATGACAGCAAGTATGACAGGGATGGTGAATCTGAGGATTGCGTAACCTTCGACTACG

CTCCATGTGAGGAAAATGCTGAGCTCTGTGCTGGAAAGAAGCATGCTAACTTCGTTTACT

ACGTGGACACCCCGGAACACACCAAGAGCAAGGCAGAGAtAAAGAAGGAgTACGCTGAAT

ACAAGAAGAAAGTCGCAGCCTGGAgAAAGAaGCAAGCGCTGAAGAaGAaGGCtGCAAAGa

aggACAAGaagAAgg

>contig00339 length=504 numreads=5

cTGACGTTGTATGCTGTGCCGTACTACTTGTTCATCATTACCTGATGAGAACACATGCTC

ATTGGCATAATCAAATGTGGTGCAGAAAATATTCGAATGATGCTCTTGCTTCATAGCAAC

TGGTGCAGCATGTTCATAGTGAACTGCCTCCTCAACATTCCATATAAGAAcACGTCtaTc

GTCTCCACCTGAAGCAAGCAGCTGCCCATCatttgaaaaatcaattgcattaacacatcc

atagtgcccaatcagatcttttcgatacaaattattacagattaacatttgatcacgcat

cagcaacttaggggataatgaacaaccAGTTACAGAAGCTGATCGcAGtAgCTTcATCaT

AtGTTGATTTGAtCCCATAGCTTTGCGTCTTTTTCAACTTTAAACTTTGAGCCAAGTTGC

ATTCCTTAGACACAAATTCATAAATTCTTCTTCGGTTTCATAGTTGCTTGTACTTCACTA

AACTAGGTTATATACTACAAAGTT

>contig00340 length=166 numreads=1

gattagtttcgtctgttccgctatggatgattgatcagagatagaggtatggatgaggac

ctggaggagaccggcgtggttgccaaagctccattcacgattattcgtgagccctacgac

gccgaatttgcagccgaaattctcgaggagcagttagagaacgccc

>contig00341 length=434 numreads=19

AAGCAGTGGTATCAACGCAGAGTTAACCTGGGAAAAaCGAGATAaCAAaCCGCACGAAAC

GGGTTtCGTCATTtGTCCATGATGCGGAGAGTGAGCACGCAACACTaTGgCTtGCAAGCC

gCATCCACGAATACGTAGCTGTACAACCTTTCcAGTGCCAaCAAGaaCAAATaTTTTAGC

GTGAATCAAGTCACGTGCGCAAACACACGaGAACCGTGCGCAGGAaTCGCAAAAACGACA

AGAAGCAGCAATAATGCAATACCcAAAAGTGACCAGAAATGTAAGCGAAAATAACGATTA

CGGAAGCTGACCTGATAAAAAGAAAAaGATAAAAAACGCGATAGCGATAGGAGAGAGGTG

CGGCTTGATGGCATATGTTGACCATACAAGTTtCAGACATGCAAGGCGTGATGATAGCGT

TGTGGCGTTTATAA

>contig00342 length=597 numreads=24

aaacaggctgtAGcATATCGTCAGATGTCcTTGCTGTTGCGTCGTCCACCcGGTCGTGAA

GCTTATCCGGGAGATGTGTTCTACTTGCACTCCAGaTTGCTGGAACGTGCTGCTAAAaTG

AATGATCAGTTTGGAGGTGGATCTCTCACAGCGCTGCCAGTGATTGAAACCCAGGCTGGT

GATGTATCTGCCTATATTCCCACTAATGTGATATCCATTACTGATGGGCAAAtATTTTtG

GAAACTGAGCTGTTCTACAAAGGTATTCGGCCTGCCATTAATGTCGGGCTTTCTGTGAGC

CGCGTTGGTTCTGCTGCTCAAACTAAAGCCATGAAACAaGTTGCTGGTTCCATGAAGCTT

GAACTTGCCCAATATCGTGAAGTGGCGgCTTTtGCCCAGTTTGGCTCCGATTTGGaTGCC

GCCACCCAAGCTCTGTTAAACCGCGGGGTACGCTTGActGAGCTGCTAAAACAGGGACAG

TATGTTCCTATGGATATTGCGGAGCAAGTGGCTACAATATATGCTGGTGTGCGAGGgCAT

TTGGATAAGCTTGATCCTGCCCGAGTTACAGAGTTTGAAGATTCCTTTAAGAAGCAT

>contig00343 length=1141 numreads=28

ttatcaatcagaaacgCTTTAATGAAGGCCAAGATGAAAaGAAGAAGTTGTACTGTGTTT

ATGTTGCTATTGGTCAGAAGAGGAGTACTGTTGCCCAGCTAGTGAAGCGCCTAAGTGATG

CTGATGCTCTGAAGTATACAACTATTGTGAGTGCTACGGCTTCCGATGCTGCTCCTTTGC

AGTATTTGgCACCATATTCTGGgTGTGCCATGGGAGAGTACTTCCGGGACAACGGGAAGC

ATGCACTTATTATATACGACGATCTTtCAAAGCAGgCTGTGGCTTATCGTCAGATGTCTT

TGCTTTTGCGTCGTCCTCCCGGCCGTGAAGCCTATCCTGGAGATGTGTTCTACCTGCACT

CAAGATTGCTAGAGCGAGCAGCTAAAATGAATGATCAGTTTGGAGGCGGATCTCTTACAG

CGTTACCaGTGATTGAAACTCaGGCTGGTGATGTATCGGCCTATATTCCCACcAACGTGA

TCTCCATCACCGATGgACAAATATTCTTGGAAaCTGAGTTGTtCtacAAaGGTaTCCGGC

cGgCcATTAaTGTGGGTCTTTCTGTGAGCcGCGTtGGTTCTGCTGCGCAAaCAAAaGCcA

tGAAaCAGGTTGCTGGTtCGATGAAaCTTGAGCTCGCCCAGTACCGTGAAGTGGCTGCTT

TTGCGCAGTTtGGCTCCGATCTGGATGCTGCCACCCAAGCTCTGTTGAACCGCGGTGTGC

GCTTGACCGAGCTTTtAAAACAAGGACAGTATGTTCCTATGGATATTGCGGAgCAAGTGg

CGACGATATATGCTGGTGTGCGAGGgCATTTGGATAAGCTTGATCCTGCGCGAGTTACGG

AATTTGAGGATtCCTTTAAAAaGCACGTtCGTGCCAGTCACcAAggTTtgctggacacca

tccgaaccgaaggaaagatCAGTGAAGCTACAGAAGCTAAAttAAAGcATTGTCGTGGCT

TtCcGTGCAGTCGTTTGCTTGAGACTAACTACCAAGGGACAATAATGGTTCGTGTACAGC

CGGTTGTTAAAGTCTTTCttAAGTCTTTaaTAAAtCGgCctGgTTTTGTTCGGCcGAAAT

GTTCATGTGCCACTGTAAGCGCTTCAaTCAGAGCTAAATTTTAtACAGtttttttttttt

t

>contig00344 length=171 numreads=4

GTAAgcAAtGGAaTATTTATaTcTTGAGAACGAGTAAAGTaTATCTCtGCcGTTGCTTGT

ATTCTTCCCATTCCAATAaTGTTTGCTATTTTGATAAAATGAATCAAaGAGTTTTaTcTT

caCATAaGGACAAcACTAAGtGAACAtaTTTAtACGCGATTaCAtATtATT

>contig00345 length=254 numreads=6

CCACACACACTTGCTGTGCCACCTGCAagAAAGGgCTCTTCGGAAAGGAACGAAGATGCT

GGCAGTGCCGgTTTGgAaGTGGCTGTGaCTACGACAAGTGCGGTTGCACCGGCTtCTAGG

GAaGACaCtAaCACCCTCAAAGTTGATGgAaTTGCGACCGGcAAAGACACTAAATGGCCA

aCATGTTCAAAGAACATAAAATATTACTCTGTTtATTATTTTGGACTCCAAAaTAAAAAA

TGTCTGGTtaaacg

>contig00346 length=173 numreads=2

TGCCTATCGCGTCTAGGTACGTTTGTTTTATATAGACGACCAGCTGGATGTGCATAtgtt

tccttacgagtctttcgagtctttcaatcgagcattaagtgaaattcgaacgggagattg

tagaaaggggtccaagtttgttgcgtatctcgtcgaggttttttgtttatata

>contig00347 length=138 numreads=4

AAGCAGTGGTATCAACGCAGAGTACgCggggggCTACAGGAATTgCGATTtGGTTACtgC

ACATAagAGCAGtGTTaGAAAaaGCgATGCcAGTTTCgAGAGGACGTGTTCTAATTTTGA

CTTCGACTAgTTAAGCcG

>contig00348 length=211 numreads=3

AAGCAGTGGTATCAACGCAGAGTtACTTTTTTTTTTTTTTTTTTGTtCCGACATTTAaGA

GAcGCAGCcAAtACATCGAgATAAACAaaCTGTCTAaTACAAAacGacAGCAAgTGTCAA

CcgcaGTCTtAAtCTAGTTCCGCaGCgCGACGTGCCAaCcGGAaTCGTCaGTaGGGTtCC

ATAGTctGacTGAgCACCCAAGCGGCATGGc

>contig00349 length=768 numreads=30

AAAGTGGCCATCTTAGGGTTTGGGATTCcAACATGGAGCTGCTgAATGAgCGAAAGCTTG

gTGaTAACATACAAGCGTTGAGCGTTtCGACCACGGCCAAGCTGCTTGCGTCCGGTGGGA

AGGAAAaCGatGTGAAaGTGTGGGATTGCgAaGACTTacagAAACCGaTCTTTGCAGCTA

AAAACGTTAGCAACGATTTTCTAAaTTTGCGCGTACCTGTGTGGGTCACCGGTGTTTGCT

TCTTGCACGAAGACGACAATAAGTTATTGGCGACCAGcGGCTACAAGCATGTGCGTCTCT

ACGACCGCCTCGTTCAGCGCAGGCCAGTCCTCGATGTATCGTGGCACGAGCATCCTATCA

CAGCAGCAACCGTGCTTCCAGGAAATACTTGCGCCATTGTCGGTAACAGTGCCGGATACA

TGTCGTCGATAGATCTTCgAaCCGGCAAGGTGGTGGGAGTTTTTAAGGGTAATGCGGGCA

GTATTCGAAGTGTCTTGTGTCATCCTAGGCAGCCGATTGTAGCTTGCTGTGGATTAGACC

GTTtCGTAAAGGTCTATGACCTAACATCAAGACATATCCaGAAAAAGGTgtACCTGAAGT

CtAGTTtGAATTGTAtGTTAAtgACAGAGAGTgACATCATGCTCAATCCCAATGATACTa

tcggctgcaaacgtcagggcgatctgaacgggtcccacgccgataatgaactccgcgata

gtcaagacggtgatgatgtgtgggatgagatgcagaaagttgatgacg

>contig00350 length=463 numreads=9

cGTATcgACCCACGGGGgCATTCAAGTAGcccGTCTCTTGATTCACTATGGTGCTGACGT

GAACGCCAGTGACAACGACCACAAGACCcGTTCTAATGATGGCGGCGCtcaatggtcaca

cgagtttagtccagctgctcatctctaaaggcgcgaatcctaggtccgagtccAGCCACA

GTAAGACTGCTCTCGACTTCGcAaGGTCGTTCGACCATAGGCCGATtGtGAaGATTCTCG

AGGAAgAGGCCGAGAGGATGCcTTCAGCAGCCGCGCGATTTCGAAAAGAAGGGCGAATGT

TGCTCAGATGACATGAGAAGCGTCTtCcTTCCAATGTTATTAAGGTAGTGCCTTGAATCG

CCTTCACCACCTTGGAACTtGATTTCTTGCCgCACCCCATACCCCTTTTTTtCTtCTTTt

AAGAGCAATTTTAAAAGAAAaTCCTAGCTtGAAAATATTATAA

>contig00351 length=176 numreads=6

AAGCGTGCATGTAAACGGACTAGCTGGTCGGTCATTTTCTCCAGCATTTGTTTTGTTTCC

AGGTATTATTAACTTCCTGAACTGGCAGTTGAGAgCTCGCTCGTCCcTTTCTTCCTGTtA

TTCCTGACCTGATAtACAcaCGCATtACGTTGGTAACgaaaaaatttccaaagaaa

>contig00352 length=840 numreads=37

GGAGTTGCTTAACTCTTTTGAACTTCAGTGCACATTGGAGGCAACTAACTTGGTTGTGAA

CATGGAGtATTTTCGAAGTAAGgAGGAGCAATTTAGGCAAGTTTTCAAAGAGTTGGACTT

tCTTGGATGGTATACTGCGGGtGGCACTAGTAGTGCCGTTGCAAATGAGAGGGACGCTAC

TATCCACCAGCAATtGATAGAAGTCAACGAAAATTCCcTGATGTTGAAGTTGGATCCCTT

ATCAAAATCTGCGAATTTGCCCATTTTtGTTTACGAATCCATGATTGATATCGTTGACCA

AAAGCCAAGAATGCTATTTATAGAAGTGCCATACGTGCTTGTGACTGAAGAGGCAGAGCG

CATTGGAGTAGATCATGTAGCCAGACTGTCCAATGCGGGACACTCTCAGGTTTCAGAAGT

TGCTGAGCATAcGcAAGTGCAGAAAAGTGCTGTCATGATGCTGCAGTCTAGAATCGATTT

AATTGTTGAATACATTAAATCTgTTAAGTCTGGTGAAATCCCcATGAACCAcGAGGTTAT

GAGAGAATGTTTAAGTTTATGTCAAAGGaTtCCCGTCTTACAGACAAGTGAATTTAGAGA

TTGGTTTTTACGgtCAGTGCAACGAGTTtCACTAATGGCCTTGCTGGCAAACATAATgAA

AGGTTgCAATGCTGCTAATGAGCTCATCAACAAATTtAATTTGACTTTTGATAGGCAAGG

TGCTGGACGCAgAATGAGAGGgTTGTTtATCTAGTTTCGGTTTGTAACCTCAGCAGCTTA

TCATtGTTTATTCGCCAACTtCGTTCTGCGGTTTAAGATTAATGCCAGGTGTGTCTAAaC

>contig00353 length=135 numreads=4

agaacctgacgagttcgagaccacatgagggagcatacttttttacggagaaattGGaCA

TGTCAgAATCAcTTTcTcTTTGATttCtttttgtgtggaggtacaatgttaaacatttac

cctcgcgaattagag

>contig00354 length=1350 numreads=52

AAGCAGTGGTATCAACGCAGAGTACGTCGGGAACCCTCCAAGACCTTAAATTCggTGAAG

TATCCACAACTCAGGCAGGGAAATGGTGCGCCggaTGCGtGACGTGgaGAaCGTAACTGA

ATGGGACATcGCTTGGATgaaaCTGGTtGgTCcTGTATGCCGCtGCGAACCCAACTAGCC

CTGTCGGCGGGCCAGATGCTTTAAAACCGCAGGGCTCTCTTGAGCTGGACAAGCTCGTGC

CCGCCTCTGCCTGCAGTAAGAGgATCTTCATCCTGTATCAAGTGATACTGAAGCGAAAAC

CGACCATAGACGACCTGCAGGCTGACCTCAAGCAAGTGGCACACCTCGCTGACGAAGCGA

AATGCGACCACGCCCTCGTCCAACTTACGAAGATCATTAAAAGCAAGTACGAATTCAAGC

ATGCGTACGCCATGAAAGCTTTCACGGATATCCAACACGAAaCTCCAaCATCAGGTCTGC

TGGAAGAAaTCCACACGATAGCGAAAaTGGCCGATACTGAGGAGGAAATCCTAAAGAaGG

TCATCAGAATGAAACcGATCGAGgACCCGACACAAAGAaCTTCAACACTACTACCTCGTC

GaTAGTGAAACCTGGCGATGATGCCAAAGTCCcGGCAACCGATGGCCCGAAGCTAaCAAT

TCTGCCAACTACTGTTGCCAAACGAGAGCCCGCGGATCGATCCTGTCcACCAACTGCAGA

AATCACCATCAAATTGTCTTCCAAGATATGCCCACCcTGGACTGAAGGCAGGCAAGCTTG

CGAGAGTGTAATTGAAGTAAACGgTATCAATGCCACGCCACCGACCTCGAAGAACaTCGA

CAGCGGGCACGCGTGCATTACCATGGACTTTAAAACGGGTAAAaTCTTGgAAAAGgTATC

CTGGGATACATATTCCGATCCGCAATCTCCGCTTAAAaTGAGGCGATGGCTGAAAaCGCT

TCAGGAGGAGACCGTGGTGGCATGCGTGGTGCGAGGAtACGGCTCCGCGTATCCCGTGAC

CTACTTAAAGGAGTTGGGAGCTGATTCTGCTGACCGAACGTTCGGCACGCcGTACGTCTT

TCTTGGGTTCAAGTCCACGAAGACGGGAGTTGTGAAGTACGGCTACCTGCCATGGATGGT

GTCACAGGGTGCGGCCTACTTGACACCGGATGTCACGGTGACGCGCTCGATCAAGTTGAA

CCAGTACATGTGTGACCTGGAGAAGCCGCCCAGCGACTGCGATCGCTCACTCGTACCAAA

TGGgATCAACTATCCGCcGTgCTTCTACAAGTtCTGTCACAAGAGAGaCATCtCGATCTG

TACGTGcGACTGCATCCCTTGTCCAAGGAg

>contig00355 length=1730 numreads=65

AAGCAGTGGTATCAACGCAGAGtACGCGGGGTCTTtCCTTGGTGGTGGCAAAaTGGCGCT

GCTTGACACGTGTCGTGCCAGGGCTGCATTTGTAATCGCAGTTTGTATTTGGCAACTGAT

AGTCGTTGCTTTTGcaGGAGAAAAGaCATTGGTGCTGTTAGATAaTGCAATTGTCAAGgA

GACACACTCGtATTTTTTTTAAtAGCTTGAAAGAGCAAGGATTTGACTTGaCGTTTAAAA

ATgCAGATGATCCcGCTTTGGCACTGACAAAATATGGAGACTATCTTTACCAGCATCTTG

TAATCTTCTCTCCGTCAGTTGAAGAGTTtGGTGGATCCATTGATGTtGCTGCAATTGCCG

CcTTTGTTGATAATGGTGGAAaCGTTTTGGTTGCTGGCAGCTCCTCGATTGGTGCTCCTA

TTCGTGAGCTTGGTAGCGAATGTGGAATAGAGTTTGATGAAGAGAAGACTGCTGTTATAG

ACCACCACAACTTTGATATAAGTGATGATGGGCAGCACACTGCAGTTCTCAGCTATCCAT

CAAACATAATCAAGGGAGCAAAGAAGATTTCGCCATCTGTTATTGACTCGCCAATCTTGT

TtAAGGGCGTCGGAATGACCGCGGATCCGGACAATCCACTtGTCTTGGAAATTCTGACTG

CTGCGTCTACCTCTTACTCGTACTATCCGGATGAAAAGATTTCTGaaTATCCTCTGGCAG

TGGgCAagAACACTCTGCTGATTGgTGgCCAGCAGGCACgAAACaaCgCAcGAaTCGTCT

TcTGTGGTtCgCTCgACTTtttCAGCGATCGAtACTtCATGTCtGGAGTTAAGAACGCTG

CGAGACCAGACTCCAAGTACTTTGCTCAGTCAGgCAaCATGTtGTtGGCAATGGATGTTG

CcTtGTGGACGTtCAAGCAGaGcGGCGTACTTCGTGTTCAAGAAGTCAAGCACCACCTGG

CCAACTCagTGGAAaCGCCcGCTGCTTACACTGTTGAGGACCAGGTgACTTACAGTATTG

AAATTCAGgAACTCGTGGgTGgCCAGTGGGTTCCGTACTCGGAGAATGACGTTCAAATGG

AATTCTTCAGGATTGATCCATTCGTGCGCGTATATTTAAaGGCTTCACCTCGCGGTGTAT

ATAGCACCACGTTCACGCTCCCcGACGTGTATGGCGTTTtCAAGTTTAAAGTCGACTACG

ATCGGATCGGCTATACGCACCtGTTTAGCTCCACACAAGTGTCCGTCCGTCCGCGCACGC

ACACGCAGTACGAGCGATTCATCGCCTCTGCCTACCCGTACTACGCGTcGGCGTTCAGCA

TGATGGTGGGCATCTGCATTTtCAGCCTCGTCTTCCTCCACCACAAGGACGACAAGAAAG

TCAAGGACGAATAGGTGTCCGCCGATGGATAGGAATATTAGGGACTTTGTAAATTATTTt

GCAaTTTTtACTGGTCGGCGCTATGTTGTTTTGTTAAACTTTTGGCGtGTTTGCCCATGA

TGgCCGATAATtAtGAATCAGGCGTCGAGTGAGATTAGAGGCAGGGTAGCGACGATTTGT

TATgtGGgCGGTTTAGTTTaGTGTGGGTGTGTTTGGAACTGATAGAgaTGCTaCTGTTGT

GATCCcTtGTAGCCAGGAACGCGTGGGTTAtCCAGGTGAGTAGTCGCTCTGCTCTTGTCA

ATgTTTtAtGTAAGAGgAAACAAAGACCTTGTTGCTCGAAggaaaaaaaa

>contig00356 length=1060 numreads=45

GAAGTTTAGTCCAAGTGGTGAGTGGCTAGCAACAGCATCtGCTGATAAGCTGATtAAAAT

ATGGGGAGCCTTTGATGgAAAATTTGAGAAAaCTATTGCTGGTCATAAaTTGGGTATATC

TGATGTAGCATGGTCATCAGACTCAAAGCtGCTTGTATCAGCATCTGATGATAAAaCATT

AAAGATTTGGGCTTTGATTACTGGAAAGTGCTTGAAGACGCTGAAAGGGCACAGCAACTA

CGTGTTCTGCTGTAACTTCAATCCTCAGTCGAACTTGATCGTCTCGGGATCGTTCGATGA

AAGTGTGAGGATCTGGGATGTTAAGACTGGCAAATGCTTAAAGACGCTACCTGCTCACTC

GGATCCGGTTTCGGCCGTTGACTTCAATCGCGATGGTGCCCTCATCGTATCGAGCAGCTA

CGATGGTCTCTGTAGGATATGGGACACTGCTTCTGGgCAGTGCTTGAAGACTCTCATAGA

CGATGAAAACCCGCCAGTATCGTTtGTCCGTTTCTCGCCcAACGGCAAGTACATCCTGGC

TGCTACTTTAGACAACACCTTGAAGCTATGGGACTACAGCAAAGGAAaGTGcTtGAAGAC

GTACACaGgTCATAAGAACGAGAAGTACTGCGTGTTTGCCAACTTTTCAGTTACTGGCGG

cAAGTGGATCATTTCaGGATCTGAAGATAACCTCGTTTACATCTGGAATTTGCAAACTAA

AGAGATTGTTCAAACCCTTGAAGGGCATTCCGATGTGGTTCTCTGTTGTGCCTGTCACCC

AACCGAGAaTATAATCGCgTCGGGGGCCTGGAAaacgaCAAGaCAGTCAAGATCTGGAaG

TCTGACacGtGaacTAAcaCgaTCGCAaTCATGGAAGCTtGTCgAGGCGAGAAGCTGATG

ACGTTTTGGCCtCGTTTTGTCGTGTCTATCATGATCTTCCCGCTTCGTATGCACCTGGCC

TTACTTCTAACCATAGGATGAGACCTTACGAAGTGTTTCAGTCCGCGCTTATTCcAGATA

GTTAGGCATTGTAAAATATTTTCTGCAAaGACaaaaaaaa

>contig00357 length=213 numreads=3

ctgcagtGGGCTATTAGTGAATaCCGAAGTTGTTTGCGATCAAGCATGgACTAgCCGTtA

CGaAGTTTTTTGCTtgCCAaGTAGCTACtGTGAaTATGTGGATTACACCGGAGGCaGgCc

AGTTCGAGCGCGCACcAAaGAAaGCATCCAGCGATTTGTGTGTGCTTtGCTTCGATTtCT

TCGAAAAAGATTttCAATTTCTTtACTCGAcAA

>contig00358 length=1533 numreads=43

AAGCAGTGGTATCAACGCAGAGTAcGCGGGGACACCtCTTTTTCCAGACAAGTTCGAGAG

AGGAAGGTGAACATCGGGAGTGAACAATGCAGGCTTTTATTGCGCTTGTATtAGgCCTCG

TCGtGGCCTCcGaGGCcACGAACTACAAGAGGGTTTGCTACTATACCAACTGGGCTCAGT

ACAGACCCGCaGGTGGAAAGTTCTTCCCCGAaGACATCGACCCTTTCCTCTGCACGCACG

TGATATACTCCTTtGCCAAGATCGGAAaCGgCGGCAAACTGGAAAtGTATGAATGGAATG

ATGACGTGATATACCcGAGAATGATGGACTTGAAGAAgAAGAATCCAAACTTAAAAATTC

TCCTCGCAGTTGGTGGCTGGAACCACGAGAATGGAAACCcTAGTAAATTCTCGGTGATGG

TCCGAACCCCTGAATCtcGTCGGCTCTTCATCGATTCCTCAATTGCCATGCTTCGTCAGT

GGGGCTTTGACGGCTTCGACCTGGACTGGGAGTACCCTGCCAATCGTGATAACTCCCCTC

CcGAAGACAAGCAGAGGTTTAcTACCCTCTGCAAGGAGTTGCTGgAGgCTTTCCAGAAGG

AGGCGGCGAAATCGAAGAAGGAaCGCATGCTTCTTACTGCAGCAGTGgCAGCCGGCGTTA

AGACCATCGACGCGGCATACGAGATTGATAAGCTGGCTTTCTACCTGGACTGGATCAACC

TGATGGCGTACGATCTGCACGGAAGCTGGGAGAAGGTGACTGGACATCACACAGCCATGA

CTGgTGATGACAAGTtGACAGTTCCGTACTCAGTGGAACACTGGATGAAAGGTGGgATGC

CCGCGAaCAAAaTTGCGCTAGGTATGGGAACGTACGGCCGCGCATTCAAgTTAaCCAATG

CTAACGTAAACGGACTGGGCGCACCAACCCACGAATGGAGTAAGGCCACACCcGGTCCAT

ACACTCGCGAAGGTGGTTTCCTTGCCTACTACGAGATCTGCAAGATGCcGCTAAAGATTC

AGAAAACcAACTCTGTGTTGGCGCCTTtCGGACACCACGGGGAGGAGTGGGTTGGATTCG

ATGACGTTGACAGCTTGATTTATAAAGTCAACACCTTGATAAAAGGAAAGAAtctGGCTG

GTGCCATGTTTtGGgCTCTTGATCTAGACGATTTCACTGGGAAGTtCTGTGGCCAGGGAA

aGTACCCACTCATGAGCGCCGTGGCAAAaGCTCTGGGaGgAtaTACGCCCCCTAaGCCGA

aCACACCTGGTCCcTTACCACcTACTCAAAAGCcAgTAAtCACCCagAaGCCCgCGACGA

ATCCTCCTCGACCTGGCACATCCGGGCCAAACGGCGgTGCTTGTAAAGCAACCGGCGTCT

GGgACGGGTAACGgTCAACAtggacagctggtgtgaggcgaattgcaagcatggaaactg

ccctgctgaagtgtgcagatgctaaacgcctcgaatgaatgtaattaatatatgggtttc

ttttaaccagaaataataaaactaaggataatg

>contig00359 length=178 numreads=2

CCAGTGAACTGAATGATATTTGGCGAAATAAAAGCATTACAGATAATGCAAATGTGCAAA

AAATGGGATCGTCAAGTAGTATTCCGGTTGAAGAAAAGCAGAGTGTCAGCAGAGAGTTGA

ACGCAAGAAGTGTGAAGAATGATTTCAATAAGGAGTTTGGCAACGTCAGAgaactcaa

>contig00360 length=217 numreads=1

aagcagtggtatcaacgcagagtcgtgcacaagccatgccctgaccctactaagtactgt

cacatgtttctctgcgttgactgcctaaaggagaacgtagcctgtacccagaacgggcag

tgttgcccaggaacggagtgcacatacggaaggtgcaagaaaggatcttccaaaaggagt

tgctggtatcgtaaaacactatgcaacccgctttcaa

>contig00361 length=1717 numreads=97

AaGCAGTGGTATCAACGCAGAGTACGCGGGGaGTGCCAATCAGGCAGTGCCAATTGTcAC

ACTtCAGCCGTGTGCAAGAATCTCgaCGGaTCGTTTGTTTGCtCATGCAAaTcAGGTTTC

TCTGGCaatGGTACATACTGCGAAGaTGTCGATGAGTGCcgATCAGGCATTGCCAACTGT

CACAAGTCAGCcGTGTGCAGAAATCTGgAtGGATCATTTGCTTGCTtATGcACtCCTGGg

TtCTCaGgAAACGgCACTAGTTGTGAAGATATTGATCACTGCTTGACGACGgCATGCCAT

CGCGATGCAACTTGCGTCGATGGCCTTCTCTTTGCAaCGTGCGCTTGTAAaCCCGGGTAT

TCGGgTAACGGGgTTACCTGTGTTGACATCGACGAGTGCTCTACTCCcAGCAAGTGCGGA

AACTtCTCGACTTGCTCAAACTCTATTGGCAGTtACTCGTGCACGTGTCCCTCCGGgTAC

GTGTTTGGCAGCCAGAATCAATGCGTGAaTAAAGACGAGTGTCAGGAAGCGCCGTGCTCG

CCCTATGCAAaCTGCCTGgACACGGTCGgCTCGTtCCAGTGCTCaTGCAAGCCTGGATTT

AcTGGCAaCGGACAGATGTGTGCCAACATAGACGAGTGCTTACTGTCGCCTtGtAGTGCA

AGcGCAGCATGCATCGATACAGTTGGCTCGTACGACTGCCGATGCGGCAACGGTCTTGGA

GGGGATGGTAGATCTTGCTTTGACTTTTGTGCCAACAACAGTTGTCCCGCGAATGCACGC

TGCCGCCATACGTTAAGTGGcCACATCTGCGTGTGTGACTCGGgTTATGCGTACGACCCT

AGTAAGGCGCGTGGAACTTCCTGCCTCAAAGGAGTAAAGATACTTATGACGAGGATGAGA

TTTAATCACAGTTGGAGCCCcTACTTTGCTGATGCCCAATCAAGAGAGTTCGCTTCTAAG

TCAGAACAGATCAGCTTCGCTCTGGACGAGGtCctAAAGCAGGCTGGCGTGTTGGGTGGC

GCCCAAGTCAGTAGAATGACACCAGGAAGCATCATCGCTgaTCTGAACATCGTGCtgTtG

AGCAGCAGTCGCAATGTGACTGCCGACCAGCTGGCTTTTATCCTCAAGAATCTAGCGGGT

AGTAACAATGCCAGTTCGATtAATTTGGATCCTATGTTTCAGTTGCTTGTGTCAGATGGC

GATATGTGCACGGTTGATCCTGGCAGATGTGGTTCTAATGCTAAATGCACGCCCTtCGGT

GCTGCCGACTTCTTTTGCTTTTGTCACGATGGATATGAAGTCAAGAACGGCGTTTGCGCT

AAAGAaGATGACGACGATCGTGTTAAAATTATCGTGCCGGTGGTCGTTCTCGGTGTAACG

TTTCTCGTGGTCCTGCTCGTCATTGTCGCATACGTCACACGTCGCAAGTCATCAGGCAAG

GTCAAAGTCAGCAGTGGACAGGAGAAAaaGGGTTTCGAaCTTAAGGATGACTTCCAGTAC

AAAGGAAACGTATAATtCGAATAaTGACTGGATTCTTTCTTTCATTTTTTTtAAaTTGTG

TAGCGACAGATAGTTAGACATTtGTTGTTAAagCAGCTATATGGCTTATATAAATTTTGT

AGTCATACGTCTGTCGCTGCGAGtAtAaTTTTgTGTTGATAACTGTCGtCCTTTTGAGCT

GTttATTTTTCTTGTTTTTATAAACTGGTtCaaCGaa

>contig00362 length=288 numreads=6

TCCTACGtATGACGTGTCATCCAtCCTGACCTCtATTcAATCGCTATTGgACGAACCGAA

TCCGAACAGTCCTGCCAACAGCCTGGCTGCGCAGCTCTTTCAAGAAAACCGTCGGGAGTA

CGAGAAGAAGGTAATCGCcACGGTGGAAGCTAGCTGGCAGAGTCAGTGATATTTGCGATT

TTCTACGGTGCACACAAGCTTCTTTCATGATCTCATGAATTTTCTTAGAAGTTGCTTTCC

AccaTGATCTTCCttGCaGTCaacatgctgcagatcaagcccattccg

>contig00363 length=100 numreads=2

aagcagtggtatcaacgcagagtaCGCGGGACTGTTTGGATTCGGCTCGTCCAGCAGAGA

TTGAATCGAAGTCAGTATGGAAGATACGTCGTATGTTGGG

>contig00364 length=607 numreads=20

GTTTCCTAGCTTATAGACTTCGTTGGCCTGCAAATCAGAGAAAaGTTGGTTCAATGTCAA

AAAATGTCTACTGACTTACAaCAGACTTTGGTAGAAGCATGTCCGAAACTTTCTCCTTAC

TTTTGCGAGTTGGATTTCGGTGCAAGAATacACCGCAGCAAATCcAACAGAGCAAGGGAA

AaTGGAAAGCACCTGATTCGAGCAAGAGCTCTGTCTGTGAAaTTtGCGGATTTTGCGTtG

AGGAGACAGCTGAAATTTCAGGAAAGTGGAGAGTGGACCTGGCAaGCTCTtCAGTTAaGC

TTGAAGCCGTtCAGCACCTGTGTAGTAAGTGCAGCAAACTTTCGCGAGGTGAAGAATTGC

TGGGCgCCATATGCCGAGGGaGtCATACTTtGgAAGAGAAGGCAGAGTTGCTTCTCCTAT

CTACTCACTTCAATGACGTTAACgATgAGgTGGCCCACGGCGAAGATGACTTGCACTTCT

TGCGTCAGTGTTTATCGCTCTCCTATTGTATGTGGTTACAACTGAGGTCGCTGAAGAAGA

GATActtAaCTGCCCAACGGAcgCGATTTGACCGAAgCcTCTTTGGCAGATTACTTGGCG

AAATTGG

>contig00365 length=197 numreads=2

ATCGACGCTACATGCACATTCACTTCAAAAACAAAaCTCAAGTTTaCAAGTCAAGCGTCA

Caatctagccaatggatctaaaaatcaatgcgatgacacgaagccacatatacgatccag

cgacaacgaacggtctcatattgtctgtctttaaacttttgttgcgcgatctgtcgccga

acaggaaaccttcgtcc

>contig00366 length=297 numreads=5

aGACAGGTTcAGGgAGTcATGAACAATGAGCATGTACgCgAAGgATTTGTtCACCtCcAG

CCGTACTATTCTCGACaCGGCATtGACGCACCGTTGCCGAAACGAAGTCTAACGGATCTC

GCTCTAGTCGGgACTGGTGGTAATGTCAGCACGCCTCAAGCGGCTGCCACATTGAGCAAA

CTTGAGGATTGAGTTcTGTGAACCTTGCcGTTGAggAgTCAAAGAAaTCGGACGGTATCT

ggCTCATTTTGGAATGGCGCGCTTGAGTTAAATATTGTTAaGgaaaaaaaaaaaaaa

>contig00367 length=369 numreads=9

AGGAAGTGCAGACGgAATTTtcGGTTAAGTTGATTAaGTTCgACgAGAaGgCGAAaGTGA

AGCTAATtAAGGAAATCAaGGCCATAATGACTGATATGAATTtAGTACAGGCCAAGAAaT

TtgTGgAAAGcGTACCACAGgTTGTCAAGgAGAAGCTgACGAaggAaaaTGCGGAGAagT

tGAAAGTACAATTAGAGAcTGCAGgCGCTgTGGTTGAGGTGGAGTaGtATCCATGCTCAA

gTGGCAGAGATCCTCCTTCCCTACGGTGTcGCTTGTCATGAAAACTTtGTtATGGAAACT

TCGcGAACGACGAAGCCcTGCAGTCTTCTCTGAATGgAaGATGTtATTtGCAAaTATTAC

GAAgTtAAA

>contig00368 length=295 numreads=9

AAGCAGTGGTATCAACGCAGAGTACGCGGGgACCTTTTTCCGGTTACAAATACAAGTCAA

CATCCTCTATGACAATGTTATTAATCATTAATAAGAACTTTTGTACGTTACAGTTGAGCG

AAAATTTTTCACcGGTATCGGCAGTAGCTGCAAaTCTCAACGATCCAAAGTTTGAAATTA

GATTTAAGGATGTTGTACATACTTGAAATTAAAGTAAAACgCTACAAAATGCGTAcTTTG

CAGGTACATCCtAGGAgATTTCtttGAAAAAAATTAaTTTtGaaattaaggtacg

>contig00369 length=137 numreads=2

ttCCATGACGATTGCGACTTGGATCCCCTGGTGTATAAAAATGGTTAGGCAgCAAGTCCT

GCTCTCGCGCTCTCgagaacttctgaaaggaaaaacttcacgaatattttcgagtgggct

gcggcgtcttgtatgta

>contig00370 length=132 numreads=1

actgacctcgctcaactcaaccgtacatttcattggggttgctacctcgaccacggaatt

gcgggtacattaacctgcatcacttacaacatgctagctgaccggtggggataattctag

cccacttgttca

>contig00371 length=182 numreads=1

aagcagtggtatcaacgcagagtacgcgggtggagggatttgttgaacgagatctacata

gactggaggtacgttcagatgaggacattgcgcgcgtacatgcgtgtttgtacattgcag

cgcaaggagtgcgaggggctagttttaccacgtgcaaacacatctacaccgctgctagta

tt

>contig00372 length=227 numreads=6

TTTTATTTtGTAAAGCTTCAGACTAAaTGCACTTAATCTTAAGTCATAACAATGATCACG

CCcAATGGCAGTCAGAGAGAATACAGTTTTaGaGCTCTTAACAAATATGACTtGAGAAAA

GAGTGCGTAtAGTACTGGAgCTTGTAACCCTGCcGTATGgTTTATGGCTGGTAAAAGtGT

AAAaGTTTTCAGATtATAACTTtACAAAcgACATACATAcatacata

>contig00373 length=223 numreads=1

caacagattcctggtccccagttttctctgcaattttgacagcgtgactaataagatgct

tatcaaggagagatagaacctcggaacagacttcgtttagctcgtcctctattttcttct

tgtaatccaacgctagatccttgtgtgatgtcttgtcaacaatcgaagaaatgacacgcc

cggaggacctcttggctccaacaatgtttttgaagccacagac

>contig00374 length=588 numreads=18

aatcggggTGGgcAAAGAAAGATGCCTCTGTGACATCAGATGCAACAAACGCGCATTCGA

CGACACCCTTCTTTCCGCTTAGCGCCTCAAGGATGgAGAAGgCGAATCGAGCCCCAGCAT

AGGCCATTGATAACGTTGCTGAACCCGCACCCGCTTTCGCCTCCACCACCTCGGTTCCGG

CATTCTGGATACGAACAGTAAGCGTCTCCAGCTCTTCCTTCGTGAAAGCGGTCTTGGGCA

CGGCTTGAGAGAGGAGAGGAAGAATAGTCACGCCCGAGTGACCACCAATCACTGGAACAT

TCGTCTTTGTTACATCGAGACCCTTCTTCTCAGCAACAAaCTGGTTTgCTCGCACAACGT

CCAAcgTGGTGACTCCAAATAACTTGTTGGGATTATACACGCCATGCTTCTTGTACACTT

CAGCAGCTATCGGTACCGTGGAATTCACTGGgTTGGTAATAATGCCAACAACAGCATTTG

GGCAGTTTTTTGCACAAGCTTCTGCAAGTTTCAGAGCTATGCTGGCATTTGTGTTGAAGA

GATCATCTCTGGTCATTCCAGGCTTtCGCGgCAAACCaGCTGGTATtA

>contig00375 length=384 numreads=3

ggtgactagggttccatggaacatggcatggaaaatgaggtaaatgctttgcagcataag

ttcatgtactttaacatggcgttttgagagctaacacccttgagcttcgatgctggcttt

acaagtgcctgcagctgagcaggtgtaattgagcatggaagcttctcaacaaCTGCTTGA

AGAGCTCTTGCCAAGGAATTGTCATTGTTCATGAAGACAAAGAACatgtgacaatttttc

aaacttatcaatttctcctctggcaaaattctgaaattatcatgaacatacTGTCGCTTC

TTGCCCATTTTCTCCGGCAAGGCCATATGTCTCTTCTTGGAGGGCATCCTCTATAACTAC

AACTAAAATCTACTATaaaaatct

>contig00376 length=168 numreads=1

tgtgcatgcgagcccaactaagcattgatctgtacaagaaaaagaactcaccttggtata

gatataaaaaaatgcctagccagagatgcagtcatcaacatcgatgagcagatgggtact

gcggcggcagaggcgattcgctcttatttcctgcataataatcgtttg

>contig00377 length=208 numreads=1

caaaacatacaataatagaaaatcggtgcaaagagacatacgttaagtacggacagacgc

ccacagaaacattccaccacaaacaaacacacgcgcatctataaatttgctaaccacgtc

gctttcgcaggggcgtgtacactacccttaaaacgaccaaccaagatttaccaaaccaaa

ccctacgactatcttcaatgtctgatac

>contig00378 length=535 numreads=17

TttAAAAGTTCAaGATtCTTtCcGTCGAAAaTGCCCGTCTCGTTGGCAAACATGATCCCC

CAAcTTCATTAGAACTTGCCAGTCTTtCtGCTTGTCGTACAGCTGGTGAGTGACGTCAGG

TAACCGAAGCAGCAAGTCACCGAAGAAAGCGACGTTCTCTAGTACATGACCCAAAGCATC

TAAATATTTTtGCTCcGAAGGAAACTCCTCCCCCGgCACGTAaCCcACTTTCCTTCACAa

TCAGATGAGCAGCACCAAGAACCTGAAaGATCTTTTCCAACACTATTTTTAGGAGTTGTT

TCTGCTTAGCCAAGTCATTTAAAaGCCAATAttctcttcacactttctatctgctctgct

cgCttACCTtGAaCCcaTAaTGATACAGGTcACgTcGGGAAGgCCCAAaGAGCTTGcAaC

AACGgaTTtGCTTCTTGTAGTGGCtCTCCTGCACGGCCATCTGTGGTCTCTTGACTTTTA

CcGATCCTCACCAGgAGCACTAATGATACTACAATTGTGACATGAATGTACATGC

>contig00379 length=150 numreads=7

catttgagcaattgctcGCTGtCCTGCcTGCTTCCAGCAGAAAaCTTCTGCCaGAAGCTT

TCcAGAGCCtCATGATCATGGATAaCTCGCCTATTATTGATTACTATCCTGCGGAGTTCT

CCACTGACTTGAATGGCAAGCAGCATGCAT

>contig00380 length=708 numreads=15

CAaGGcACCCATTCTACTACTGTTATGATTTAATTGGTGGCGAGGTTTTGAAAATCCCcT

TAACTATCGGGAAAGTGATGCAACGcTgTTCCCGTTTtGTTATATCACCTgACAGTGGAA

TATtGGcTTTtCTtGgAGaCAACGGCTATTtGAatCTAGTGTCAGCAAAGAGCCATCaGT

TtATCGCAAGCTtGAAAATGAATGGCAGCATAGAGGCAGCTGCCTTTtCTTCAGAAgCGT

CAAAaGTGTATACTACTGGTGGTGATGGAGAAGTTTACTTATGGgATATCGGAACCCGCa

GATGCATTCATAAGTTTACaGACGATGGTTCGTTGAAAtCAACTTGCCTaGACGTTtCTC

CAGACGGAaGTCATTTAGCTGTtGGgtCAAGTAGTGGTGTGGTAAACGTGTATGACGAAG

CATGTCTTTCTAAGAGACGTCCTTCTCCAGtGAAAAaaCAGTGCTCAaTcTAACgACGCA

CATTAAGACTGTGAAATTTAACTCCACGAGtGAgATGCTGGCGATTTCTTCTCCTGTTGT

GAAGGATGCTTTGAAGCTGATGCATATTCCTTCAATGACGGTGTTCTCAAATTGGCCGAG

ACAGACCACACCACTTGGCTACGTtGAATGCCTTGATTTTtCGCCAAGGAGCGGgTATTt

GGCCATCGGCAATACCAAGGGCAAaGCTCTTTTAtACAGGCTTACCCa

>contig00381 length=243 numreads=1

aagcagtggtatcaacgcagagtacgtttcggggcgaatttagcgaagaagaaaaccgtc

tcgtacactggcggtagcttcctacggaagcagaaacaagctacgactgatgacagctcc

acacgagtgtacaagcccgttacgaatggtagccctactgaagtccttgatggcatgttc

ccgcttcccgatgcggccttggagaggctgacacggtgcattatgctaccacggactttg

atg

>contig00382 length=156 numreads=3

aagcagtggtatcaacgcagagtacgcGggCCGAGAAAAGgTGGAaGATTCCCTACGaGG

AaTATCTTTGGggaGgTAaaGaTgCcGAgAAAAAGGTGAAGATtCCCcTACGAGGaatat

ctttggggaggtaaagataccgagaaaagggaagat

>contig00383 length=1130 numreads=66

aagcagtggtatcaacgcagagtacgcggggacacgctcctttgattcaatgaagttgag

agaagatcttgtgcgagggaTATATTCATATGgATTTGAAAGACcGTCTGCTATACAGcA

aCGAGCTATtCtGCCTATAATTAAAGGCCGTGATGTTATCGCTCAAGCTCAATCTGGCAC

TGGAAAAACaGCAACATTTTCCATCGCTGCTTTGCAGCAAGTGGATACAAGGTTGCGTGA

AACTCAAGTCCTTGTGCTTTCACCTACGAGGGAGCTCGCTGGGCaGATTCAGAAGGTGAT

GCTTGCCTTGGgAGATTTCATGAAtGTCCAATGTCATGCTTGCATAGGCGGTACGAATAt

cGGtGAGGAcATTCGGAAACTGGACTATGGGCAGCACATCGTCTCTGGAACGCCtGGCAG

AGtcTtCGACATGATCAAGCGTAGAAATtTAAGAACAAGATCAATTAAGCTGTTTATTCT

GGACGAAGCTGACGAAATGCTGAACAAAGGCTTCAAAGAACAGATTTATGATGTTTACCG

ATTCCTTCCTCCGGCAACGCAGGTTGTTCTTGTGAGTGCTACTCTACCACATGAAATCTT

GGAAATGACGCAAAaGTTTATGACAGACCCTATTCGAATCCTCGTCAAGCGTGACGAGTT

GACACTAGAAGGCATCAAGCAATTTTTCGTTGCTGTCGAGCGCGAAGAATGGAAGTTTGA

CACACTTTGCGATCTTTATGACACGTTGACGATAACCCAAGCGGTGATCTTTtGTAACAC

GAAGCGAAAGGTCGACTGGCTCACCGAGAAAATGCGAGAAGCGAACTTCACTGTtACTTC

TATGCATGGTGACATGCCgCAGAAAGAGCGCGAGGCTATCATGAAGGAATTCCGCTCTGG

AACAaGCCGTGTGCTCATTTCAACGGACGTTTGGTCACGAGGTATTGACGTTCCTCAAGT

GTCGTTGGTTATCAACTACGACCTGCCCAACAATCGTGAGTTGTATATACACAGGATCGG

TCGTTCTGGTCGATATGGTCGTAAAGGTGTTGCCATTAACTTCGTcAAGTCCGACGACAT

TCGTGTTCTTCGCGATATTGAACAATACTACAGCACACAGATAGACGAGA

>contig00384 length=722 numreads=20

tATATATGTAATGGGTGTCGGGTAcgCTgTGGaTCTCgTTGTGTGTTCAGcgCTCGGTGC

GGatATGTTCGATTGCgTGTTtCCAACAAGAACtGCGAGGTTTGGTTCGGCACTCGTACC

GCAAGGCCAGTTgCACTTAAAGACGCAGCAGTTTGCTGACGACTTCACACCGATCGACAA

AGAGTGCAAGTGTCCAACTTGTGCGCGATACACGCGggCTtATCTGCATAGCATCGCTGG

CAAGGAAACTGTTGCGTCGCATCTCATtACCATCCACAACATTACTTATCAGATGAACTT

GATGACCTCGATTCGAAGAaGCATATTGGATGGGCGTTTCCCGGAATTTGTTCAAGAATT

TATGTCAAATATGTATCCGAGTAAGTCTTATCCGGAATGGGTcGTCGAGGCTCTCGCAGC

CGTTAATATTGTTCTCACaTGATTGCTGTCACATTGCGTCGGCAAGAAAATTTTAGAAAT

CTtAAaGTGGAACTTAGTGGTCTTACTGCtGCaGAGAATACACCATAGCGAGCCAAGAGC

TTGCACAGGGACGAGAGCATGCAGCATCGGGAGACAAGAAGACACAACATCGGGAGAGAa

GAGCATGTAAcTtGtCTAaGCAAGAGCTGgCaatGGTgggAGAAAGGaaCATGGATCATT

GGGAGACGgAAGgCATACAGTATCGGGAGAcAaGAtcaagcaacatcgggatataacggc

ag

>contig00385 length=234 numreads=3

CTTCGCTGTCCTCCTCCTCAGCCTTCAGTCCGTACTGGCGCGAAGCCAGGACACTTCTCA

ACTCAGAATACACGAGATGGCTCGCTAAGTACTGGCTCCTTGCTTCTGCCAAGCTCGACT

TCGCTCGCTGCAGTTgTtcACTcAGCTGCTtAGgTACCAACcGaTCGTCGAGCACGTGCg

TtATtCGCGcACAAAgCGAGCtGAaGCCaGGGTaCTtCTCGaGAAGaGCGTCAg

>contig00386 length=610 numreads=16

cATGCtGcAgtAGgAGTGaGCTCTtCTCAAaGAatAaCCaCAAACGTGGAGTTGCTATTA

AAaTGACTCAGCGTATCTATCCACTTCCAAGTTTTACGCATCTGGACCGGCAGCtATTTT

TtCCACAGAACTTTCCCTCTATTGCTGCTGGAAaTTCTTTCCCACTTGACCcAGATGCCA

TTGTGCTTGACATGTGCTCATCTCcAGGTGGAAAAGCTTCTCATATTGCTTCTCGTCTCA

ACAACAAGGGATTGGTGATTTGCATAGATAAGACGAAATCGAAAATTTCAAaGGTGGATG

AAAaTGCAAAAGTGCAGATGCTGCGgAACATTAAAACaTTtGTATTTGATTGCACtATGC

TTTGCTCGGAAAATTCAGAGAAcTTTTCAGTTGATCGgAtGGTTTTGCATGGGCCTCCAT

TTCCTCGCGAGTCATTCAAGTACATtCTTCTGgACccTCCGTGCAGCGGGCTtGGCCAAa

GaCcTCAGCTTAGGtGTGAAATGACTGATGTGGAACTCCAGTCATATCATTTATACCAAA

GAAAAATGCTTGCACAGGCTTTTCTCCTGTTGAAAACCCACGGTTACCTCGTGTATTCAA

CCTGTACCAt

>contig00387 length=980 numreads=14

aagcagtggtatcaacgcagagtacgcggggagagcgcagaaaaccaactttcACGAACG

AGACGGTGCTGGACAGGAAGCCAGATTTGCgATgTCAGGTTCGGAGCTtCCcGcGACATt

cGtCGACGGcTTTCATGATGTCAATACCGTTCGGCGAATGGAGTACCGaCACCTGGGGAG

CACAGGCATTCAAGTTTCCAAGTTGAGTTTCGGCGTTTCGTCGATAGGGAgCGtGTTTcG

CCCGAcTGACGACGTAGAGTCTTaCgACGTGATTGAGACTGCACTGAAGAGTGGCGTAAA

CTGGATtGgATGCAGCTCCATGGTATGGTTTTTGgCAAAGGGGAATCGGTTTTCGGTGCT

GCTATCGAGaaCaaGATGATTCCCCGAAACGCATACTtCTTGTCAaCGAAGGTTGGTCGT

TACAATCCCGAGAGACGGAAAATGTTtGATTTCTCAGCCGAAAGAACGaTACGTAGTGtc

GACGAGAGCTTgCAGAGAATGCGATGtGACTATGTCGATTTGATCCAGGTGCACGACATG

GaGTTtGCTCcTtCGCTtGACGTAATCGTGAATGaGACTcTTCcTGCGTTGCAGAAGgtG

AGGGAGTCGGGCAAGGCGCGTTTCATTGGCATAACaGGgTaTCCCCcTCgggAATTTCGT

GCAGGTGCTGGAGCGAAGCACGGTGAAGGTAGACACCGTAATGAGCTACtgccgctattg

ccTCTTTGACACTGGCCtGgATGACATACTTCCCTTGCTAAAttctaagggcgttggcgt

cttcagtgcttcGGCTAACGGCATGGGATTGCtgAccGGGAATGCGCCACCGAaGTGGCA

tCCAGCCAGCGAACGcACcAAGAAGGCGTGTgAAAGTGCaGTTGAATACTGCGCGaCGCA

GCACgTCgACAtCaGTCGtCTTGGCGTCaGCTTTTCATgCCAAAACCCCACATACACtCt

aCCATGATCAGCACGGCCAg

>contig00388 length=136 numreads=1

gtcacaataacatgatggccttgaagccggttttatcagcgtggcttgacgaagccgagc

gagctaacagaatcaaggccaaagacagtgctttcttaccaaatgctgacaaaaaaagaa

aacgcacctctatagg

>contig00389 length=233 numreads=3

AAGCAGTGGTATCAACGCAGAGTAAgCTCAATGTccGAACCAGGTTTTACTTGTGGCGTA

GAGAAGCTCAGCGACACATGGTTTTGCAGCTCTTCCTCAAACACAATTTCCTTGGCATCA

CCAATAAACTCTCCATCTTTGCGGATGTAGTATACCAGTAGTCGACAaGAAGGGACCATG

TTCTGTGTAGCACGGAACGTCACAGCGACGGagCCGCGGCCTtATTTtGTTAt

>contig00390 length=255 numreads=16

AGCTTATGCGCTTGTGATGAAGAACTGTCGGAAACCGTTTGATTTTATCAGGTAAAAaCT

GAAATGGGATCCTCCTGCTGTCTACAGGAAAGTGCATAGAAACGTGCTACAATTTTTTTC

CcTATCAAAATTtCATTGGGTTgTTtAACTTGATGTTGTGTACTTGTGTTGTCCATCACC

cTTTATATTTGGTACATACTCATCGTTAatCTCATCAaTaTCATCAATCTCATCTTTTAC

TTtacatacatatta

>contig00391 length=1218 numreads=52

ATTTTtCTCACACAAGATTTAAGTTTCtCGATTTTCtttttACCtACGCAGATTcTtCAT

CAAGAGAAaGTCTCTCTTATTGGcATGTAAGCGTCGCTTCGCTCGTCGAAACTGGCGTCC

AGTATACAGcGTCCTcAaTGCTCTCcACAAGAGGCGTCACCACTTCGGGGCACCACCAAG

TATCAGACCACGGCTTCCAcTTAGCTCAGCGCGACcGAGTCTTGCGCACCTTGCACTTCT

TCGATTtAGaGTtCACCActGGAgAaGGGGGAaCaGGGATTGACGCCCcAGTCAAaGTGT

CTGATCcGGTGGAgAGATTtGACTCCTGctCCCACGCAATGTGGTTTAGATCTCTCAGCA

GTGgCccGCCGTCCATATCCTCGTATCCATCCACGTCAACATGAATATCGTCCTGATCAC

CGAGCGCAGATCcTTCAGGTCTCATGCTGACAGGCAGCGGTACGTcATCAGTGTCGCCcA

GCAGCAGCTCCCTAACTGCACCCCTCTGCTCGTcAGAGTGCTGCGCGCAcTTACCcGTCC

TCGTACACATTTTCCCcGTGTGTTCTGATATAGCGCCACAAGTTTGCGTCAGCAAAGCTA

TCTTTTCCTCTTGGCTTAGCGATTCGAGGGCTTGCAGAGTTGGGCCAACCTTGGCATTGG

CATTAAGCTCGCCACTTGCTGTAGAACACGGCGTTCCGGGCCTGGATCCACCACCGTTCG

CCTGCACCGACGGGAAAGGGTGTGGCGGATTGCCGTCAGGATGGATtGCGATTCTGTCGC

CAAAAACGCCCTTTCGGATCTTCGTTCGTCTTGGTGAaTTATTGATCCTTTCCcTGTCTT

TCTTGAATTTtCTACTCTTCTTGTCGAAGTCGTAAGTCCAGTCATAATCGATCTCGCTTG

CAACATCATCAAATTGATCGACTTgCCcAGAAGCTTGCAGCCTTCTGCTTGCGATTCTGC

TGCTGTTCcTACCCATTCCCATGCACTTCTCCAGGTGGGGCGCGAATCGGTTGgCCGCCA

GATTTCTCTGGCATTTCGGACAAATGCACTCCTGCAGCTTTTTGAGCGGCTGCTGCCCGA

ACACGTCTAGACCAGGCTGGTTGACAATTTGGAATTCTTTCTGACCTTCATCATCGATTT

cTtCTagCTCCCAATGtCCcAAgCGAATAGAGCGATGTATTTCAAAGCAAATTCCTAATG

TTTCTATGTCAATGAGtt

>contig00392 length=241 numreads=1

aagcagtggtatcaacgcagagtacgcggggttagtgatagctggaacggctgatatcga

cataaaggattggcgaaagaacacggaatatcgctcaggctaccacgacaagcatcaagt

ggttgaatggttttggagagctgtcgattccttttcgaacgaaagaaagttacgtcttat

acagtttgtaacgggaactccgagcatcccttacgaaggcttctctgccttgcgaggaag

c

>contig00393 length=271 numreads=12

ggAAAAGGATTtGCAAGGCCTGGGCAGCTTAAAAaTCACATGCAAAAGCATATGAAGCTT

GAGGAGTCTTTCACGgCCGaGTAAGGACATAACTGCCTTGGgATAgCCACGACcTTCTTA

CTGgTtGTgATAAgTGATAGgATTCTTTGTTTTCAGTGAAAAAGTTAAAaGTTtGAGTTA

AGCAAaCTGGcATGTCATCTGTAGCATTtCcATCAAAATGCTGCCACCATTTGTCGCAGT

TTGTTTGGGtACgTTTCGGCgACGCATGTTg

>contig00394 length=1141 numreads=44

tCGGAccGGCTTTCGAATCGCCCGGGAAATGCAGCAGTTTGGCGGTGGTCTTGAATgTGC

AAGCACAaGAGAaTTtATAATCAGAAAAGCcACATTCATGCGCAACTATTTGCCTGAGGT

GTTGGAGTCGgTTGTTCCcGATGTAATTTGCCCTCAGTTTCACTCTTGGGACTTGgAGGA

AGCAAAAaGTGCTGCCCGTGCTGAAAAcGCCGCAGTTGCTGCTGATATCTGTGCAGGCAA

TCTTGAACTGGCGCACAAGGCCGCCTACAGGTCTGGTCTGGGCAACTCGTTGTACTGtGA

CAATCTTAGCCTGGATGGAATTTCTCAAAGTCAGCTCCTTGCGTATACTCAGCATCATCA

TGTTGGAGAAAAGATTACGATCGCCGCAGCTGATGTTGACCATGATGAGCTCGTTCGCTA

CGCTCGCGATCTTCTCAATGGACTACCACGAGGCAAGCCTTCTGCTGGAAGTGtGCAAAa

GTATCATGGTGGAGAGTACCACGTAAAGACaGgCACTAAACTCGCGCATGTCTCCTTGTt

GGCTGAAGGTGTTAGCTGGACGTCGCAAGATCTAGCTGCTTTCGCTGTTATGCagCGGAT

CTtGGGagCATCCTCTGCTTTGAAaTGGGGAAGCAATGCTGCCTCGAGATTGAATAAGGC

CGCTGTCAAAGTCACTGACGGTCCTCTTCTGATCcACGCACTGAATTTCAACTACGCTGA

CTCGGGTCTCTTCGGAaTTTATGCGATCGCGTCTTCTAACGCCATCCATCCTGTTATGAA

AGCCGCTGTCGCGCAAGTTGTCAGTCTGGGTAAGGGAGAAGTCTCCGCGGAGGAATTAGA

GCGTGCAAAAAaCCaGGcACGCTCGAGTGTGATGATTGCctACGAAAaCAAAGACGACTC

CGTTGAGGATTTAGCAAAACAGGTGGCCTTGACAGGCTtGTACGTGCCGGTAGATAGCGC

TGTAGCCAaGATTGACAGcGTGACGAAGGAGCACATCGTCAAGATTggaCAGAAATTGTT

GACTGGACCCgcAACACTTGTTGCAACTGGCGAAACTGCAAGCAGTCCTTACATCGACGA

GCTgttcccctaatgttttttatctgattcggctgtaagggcttgttgcgaggttggatt

t

>contig00395 length=241 numreads=2

CAGGATACAATCCAGTGAAGAACGTCCATGTCATCGGCCATGTCAACGTTGGATAGACTG

GTATGAGTCGCTCCACTCTTGAGCCACGTAAGCTAAAGTGGACCAAGTTTGGCATTTCTG

ACTTGGCGAAGACGTCGTGTGGCactccatcaagaaataggaagaaaactttcggtcttg

gagctgcaggttcagttaagacaggaagggtactagcgatatgaagaattgctagcacgt

a

>contig00396 length=245 numreads=2

aaaTTAAAATGACTCTTCTACATAGTTGcACCCCCcAGTTTTTTTTTtAGTTTTTTTtGT

TGCCAGGACCTTCGAATTAGATCCAAaCtCTTcaactctaaactttcaaagtctagccgg

atctagcgtcgtcgatttgattggaatgggagtttgtggactgcaatcgggctctctcaa

gtggaagtgagaacttcctggtcaaagacaaggttaaaatagccttcgcgtagcccgttt

caaag

>contig00397 length=319 numreads=4

gCTTATgtGGGTTCCAACCGAGAAGATGTATTGAGCTTAAttttGCCCATAATGTCTGAC

TCCAAGTCGAGCATGGAGGTGGCTGGGGTAGcTtCGCTGGCcTtGCGGTCTGATATCTAT

CggCTtCTGCAATAGCGAGGtAACAAGtgTAgTaTTGCAGACTCTTATGGAGCgATCGGA

AATAgAACTGAAAggTCATTTTGGCAGATTGATAGCTCTTGGCCTGGGCCTGACTTATCT

CGGTAAGCAAGATGCAGTATCtGCCACGTTAGAGACACTAAAaGTGGTTCAGCagcctct

cgggctttgggccagcgta

>contig00398 length=783 numreads=24

aaCCACATCgAcAGCctGACCGACCCGCACTTGAaCGGGAATAGTTTGTCGCTGGGCATC

AAGAGTGACTAACATGCGCGGTTGAATCGCAGATACCAACGAGTACAACAGGTAATGGGA

CTTGCCTAGAATTGTCGACTTGACATCCAGGCAGGCAGTAAGGGCAGCAAGCAAACCACC

CACGGCGCACGGCGACATAAACTGGCGGTCGTACTGATACGGGCTGACGGTCAAAGTtCC

CTTACCAAGGTGAACAAGACCCTGCGCCAaGCGAACcATGAACAaGttGCTTGCAtCTTt

gTGAtAGTACAAAGCTAGCTGTCGAAGCATACCtGCCAAGCGAGCATTGTTCGTACCCGC

TCCAACTATTCCCATGGCAAATATAGAATTATGAGCAACTTCAGCATCATGGTCATGTGA

AAACTTGCTCAAAGTGTCGATCACGCTCAAGCGGGgATTTGAAGCCGACAACAAAGCTAG

GCCCAAAGGCACAGCCCTTTTTATCAAGGGCTCCCCATATTGCAACAAATGATTGTAAGC

CCGCAGGGCCATTTCAATGCCGATATCTTCACCCATGGCcAcAGCAGCTATTCCCATAGC

TGCAAcaCCTTGATGAgAACCATCTTTCTCTGAAATAGcAccActAcTATCAGCCTTCTT

ATCATCCCTGAGTTTCTTATCGTCCTTACGATTTCTGTCTTCTTCTCGCTCTTTGTCTCC

TTCAAAATGTTCGCTGCATATATgAaGCAGCTTTTGCACTGTCAGCAcgTTGCCCGTTCC

ctt

>contig00399 length=241 numreads=1

caagagcgccagcgtagtaatagcagccgcctggaagaaatgcggagctacgagcataca

aggcagacgtcagtagccgctgaaatacgggacagaaatgataagcctcaaacatcgcat

gtttcgcaaaatagaaatcccatctcgggaaggatcttccctaccgaagccaaaaaactt

tgcacgatatttcaagcgacgatagcgttctgatatctggcaagtttcaaagtccacttc

t

>contig00400 length=195 numreads=1

atttgctgacgcgaatgcgtgtgtggcagcatgttccgacaggcgtaagggagatccctc

gattaacggcgtgacggtggactctgctacggaaaagaaatgcttctgcgaaagaggaat

gaccgggtcgagtcctacacgagcttggaagacttgcgagttttcgtaagagcactctcg

ggcctactcgtataa

>contig00401 length=518 numreads=11

aGTCTAGCTTtCTTCTCAGCCAAAGCCTTctCCCTTTTGgCTAGATCAGCATCTGACAGA

CCAaCAGCGCCTGGAGAGTtCTTTGTGATGAcACcTTCGTAGGCCAAATACAAATTCcGC

TtGTCAACTtCTTTTTtGCTGTCaCTCTCCTTGCGCTTTATTTCGGACAGCTTTCCTCGA

GTAACGGCCAGCGTGACACTGACTTTTCGGCCATCGATGTGCAAAGGGCGGCCTTtCCTT

tCGAGATTTtCGATCTCCTTCAAACAGCTGTCAGccGCAGAcTTCTCTTtAAaCTTtAAG

AaCgCCGTTCCCTTGCtGTGACCAGTctCAGGATCATGAACGATCTTACAGTCGTCgACT

TCCCCGTACTTgCTAAAaTGTTCCGTGATTTCATCTTCcTCAGTGTTGTACGAAaGGTTC

cTTAGgAAaaCCGtATGGCCGGCCTTAgCATCCTTCATTAGAGTCTTCTTCcGAGCCTCC

CTCTGCCGGATGGaCTCCTCGTCATGCGaCTCTTCATC

>contig00402 length=757 numreads=11

aagcagtggtatcaacgcagagtacgcgggcaactaagccgagtactacctaagtatacc

attctatctggctgggaaaacgtttgccctaaatacaggcctatgaataatactcttaga

agtaaccttgtaaaatgtaggaatgtggaccgcAGGACaTGtAcATATGTTGCCCccTaT

TaTGCTaTgAaGAcGCTtgCCGAGCGTCGCCTgTCACTACCTCGtGACTGTGACTTAGAG

GAACTGGAGTCCCTTACGTCacagttgaatgggttacatgcaaCAAGTCCAGAAAGgTCG

CTCGCGCCAGGCAATTGGTTGCTTGTTAACAAGCTAACAGATCTTTTGTGTCGGTATCGC

TATTATATTtGCAAGAATGTGAGCCATGgcTTGGgCGAGGCTGAaTtCAAGGTaCAcTCT

gAAAaTaTTCGCTTGCTATTTTGCAAGCTTGACCAGGACATGGGCTGTTCCAAGTGGgAa

GCGgTGCAGgAggaCAAAGaTCGCGAAgAaCTcaTAaTTATtGTCTATCAAATAaaaaaC

GTTTTACGcGAAaTGAGTCGCTTGCTGTCAGTCGGAAATgAaGGagCAGAAGACcAaGCG

AGTGCTTTGCGgCTCTTAAAGAGGTACAACAAACGGGGCCAGTaCcAACTCGCGCTTGAT

ATGCTGCGAGTGGAACCTGATTTTTCCAGGAACgACGATTTtGAATTATTttaCCAaTGC

GCGaTgagCcACTTtAaCcTTGCACGACccAaaGAAg

>contig00403 length=226 numreads=13

CAGTGTAGGATCGAGGTATGGCCCAACTCTTAACGAAGTGAAGGTGTCGACATATTCAGG

TCATGTCAACGTGCCTTCTCTAACGTCCTGATTTGCCGATGTCGTATTCGCCCAGGAAAA

GTTGGGGATATCGATGCACTGCATATATTGGCATTCGCATGTGGTgCACATAGATTCCAG

CAATGTGGCATtGCCATTGAAGCTTTCtGTACatCTATTtAAGGTC

>contig00404 length=253 numreads=11

GGGGGATGAATTCTCGAAGAATAAGAACTGCAGGCTTGTAAGGCAGCCTTGACACTTCAT

TTAAAATTTGTTGATAAGTCCTTAGAATCACCATATCGCTTCGTGAGTGGTTAAGGGCGC

TGTGCACAAAATCTGGTGGATGCAGGTTCAACCCTTGCTGAGGCTACACGTTTCATAGGT

CCATTTAGCCTtAGACTCTTCTGGGTCAGgACGTGAGTATAaGTCACTTAcaGGgAaTCA

ACtttatgatctc

>contig00405 length=239 numreads=2

aGAAAGTAAAAaTAACCACATCTAAAATATTCTTGGACTACTACAAAACCTCGCTCTCAt

CcTTTCTTTAGTCAGCATATTAAGtCaTAAAGgACAAggataagaggtctcactaaagga

caattgacgctgactctgccacagaccaagcggcaatatccgcatccagaagttaggaat

ggccagattggacgattgtcacgggcgaaggtagcagattttgcgcctaggtacggagg

>contig00406 length=326 numreads=5

ACACTGATAACTGCaaTATGCCTTATTGTTTGCGCACTCGTTCACGTCTACGcAtttttt

CGTGTTACTGTCAAGTTGGAAGCCAGTTTTGCAGTTGCAGTAATATCGGCcGTAGGAaTT

gaaacacgttcctccaTCACCGCAAatGTTTGGGTCGTCAGAACACTCATCGACATCGAT

ACATTGCTTCTTgACAaGTCgATAGCCTTCGtCacaTTtCACACGCtCGCATGAGTAaCT

GCCAACATGATTGATGcATTGAAAGTTTTTCGGACAGGCTGCTTGTGATACAGTGCACTC

ATTCACATCTACGCAAACGTTGTTAg

>contig00407 length=241 numreads=1

gaactagaagaattactctgcggaatagcttcgcactttgctccattttgacacaggtta

ccacatctatctacacacttagttatttctcttggactccattctccattctcgtcacaa

acaattttgtggaactcagatccatctatgaatgcatagccttgcgcacaagagtatctt

attatttcaccactcttgtactggtcttgggaaggagatagtgcaatagcatttagcggc

t

>contig00408 length=661 numreads=34

TTTTTcATTTCTTTGCtGCTGCCTTcTGCTTCtcAGCCTCCTCGGCCTTTATTtCGGCAT

CGTGAAATTCCAGAAGTTTGTCCACAGCGTCcGACTTGAGAATGCTAaGCAACGTGgTAT

tACCcTTTCTCGTGAGAGTGGCGATCTCCACTTTCTCGgACGTCAGCTTCTGAACGTCTA

GAGTTTTGCTAAGCACTTTGATCGCCAGTTTCAAAGCCTCGTCGAGGGAGATGCCGTCCT

CGTTGTAATCCTGTTTTAGCATCGATATAGCAGCAGCGCTGTTGTTTCCTATGCATGTGG

CTTTCCAACCagAGTAATTGCCACTGGgATCGCTCTGATAAAGCTGGAATCCTAGCcGCC

TATCCCAtCCCATATAAAGCAGgGAAACACCGAAAGGCCGGTAgCcTCcAATCTGAGTGT

ACCTCTGCTTTAGATCGCAAAGACTGCTAATCAGCTGCTCCACGGGCATCGGCTCCTGGT

ACGTtAACAAATaTCTCTGAGCAGTTAGTCGCAGCTGTTGTGTTAATaCGTTGGCGTCTG

ACGTAATTCcAGCTACACTGCACGCAGTGTTCTCATCAAGCTTGTATATTTTATCaGAAA

aCACATCATCCAACAGCTtGTTTGTGTTGCgCCTTTCAGCAgcaagaagtacaccatctt

t

>contig00409 length=557 numreads=10

AGACGCTtATGGTAATTTGGGTTGCTGCGGTGGTTTCGACAACTGCTACAGTTGACTCTA

AATCTGAAGCgcttattatttcactggtatttatagattcagttttggatggaccaacgg

tgccttcaaccgttttcgcTGTACTTACGTCAACAGATGAAACCGTAGACAAAGGCTCTA

GAGTCGTACCAACTCTCATCaCTAGGCTTGTTTCTTGAGATCCGGTcGTGGTGATGTCAA

TTACTTCGGGCGATTCAGATTGTACATTGCCTAAAGTTGGCTGGGCAGTAATTTCGACCG

CATCTTTTGTTGACGCCTCGGCAGCAAAAGTGCTTACTTCCATTGGAGTCACCGAaTCAA

CCAACTTCGCAACAGCAGTGCTAAACAATATGGGATCTGTCACTATGTCAGCACCAGTGG

ACAACAAGTCTACAGCCGGATCACTAATAACCGGAGTTGCcGTAACGTCACcATCTTTGg

AAAAgCCGTAGCTTCCAAGCCAGATGTAAcatcaccagcagctgtagtcaatagtgacgt

cggtctttccttcaccg

>contig00410 length=241 numreads=1

agcagtggtatcaacgcagagtacgcggggacgtcaggacgtgagattgaagagaaagtc

atccaaggctttcaagacagctgggagccagctggcttccgatcgggaactgggaaaggg

catatcctgaatgcacagccagtgatttctaattacgttacagagcagatcgtgttggat

acccccgagatgccaaacggaaaagccgggcacaaagacctgaagaacgcgagaagtgat

g

>contig00411 length=209 numreads=6

ggggggCAACGTTCATCAAGCTGGATCATGTTGGAGAGGAAGGAGTAGCCTGTTTCTGCT

CGACtATCTCCCTTtGTTAACGTATTGCGAGTGAGGGgTtGGGAAGgAAGTtCACGGTGG

GTCGTTCATCGGTAAaGTGCAaTCTtCTCCAGCTTACCGTAGTCCTCGCGgtCaTTCAAG

AATAAtGCCTGGctttttcgaatttgtca

>contig00412 length=356 numreads=2

aagcagtggtatcaacgcagagtacgcggggagcacacagctcttcgcttagacactggt

aagagttgacgagccaagggcaggaaaagttttgtattcataccgaatcatCAGAACAGG

GTCGGAATATAAGCGAGAAAGAGATACGGTGACAAAATGATTACCATTCATTCGCTTATC

GCAATTGCATTCGTCGCAAGCTGCTggCAAAaCTCGCTgCAAAAtGACCttgAAACGTTT

AaGTTGTTTGTgcgaagagctcaggcgatattggccgaagataatccattcgtatccatg

aaagtcctagagatgctattcaaaacggaggcaaaccgggcagaactcgagcgccg

>contig00413 length=169 numreads=3

ctgagcgtgaatacatgtactatcgatgaaaggagtatggttgcttcctgtccttacctt

acgctgtcaGAAGTGAGaaaggggTTGTGCCTGATTTGCATATGGTTCTCATCAGAGCCT

TCAAAAATATGACCACGTTGGACTACGTTCTTTTCACgTATctcccTTT

>contig00414 length=662 numreads=27

AaaaTAAAAAAAATCGTCGCCCTAGACATCTGTtGCTtCATCAGGCATGGgCGCcGGtAT

GGTTTCGTAGGCGTTtGCCGAAGAAGAGACATCtCCAGACTCCGGCGCCACAAACACGGC

GCTAACACTTGAATCTAACTCCTGCTCGTCAaTGACATTGCCCTCAGAGGAATCAGTACT

TTTTCGAAGGCGCTTCCGGGTGATATAAaCCAGCaGagCAGCGATAAGCACAGCAGCAAG

ACAGCAAAaTATGACAACAACGGCAATGAGACTCGCAGATAAACTTGCtGCTCCTTGTAC

AGCATACTGCTCTTCACTGTTAGGCAAAAAACaTTCAGTCTGTACCCCGGCGCTGCACAC

GCACGGCAAGCAGCGAGATACAGTCACTCCCACTGATtGCATTTttGTCAGGTCTTCAAC

ATGGCTGCGCAAAGACATCTGCACAGTCTTTAAGtCcGTCTtATTCTCGTACAAGACAGG

GCcACGTGCGGTTACCAGAACGCCATATCGATAATAGATACCCcGgTACTGGACATCAGA

TGAGTGGTCCtGCCATGAGAAGTTCAACTGATGCGTGTGTCTtttGTAGgAGCAAGTAAT

CTGCtGAGACAACGCcTGAAGAGACACTTCAACTGATAAATTATGAaGACAAGAGACTCC

AA

>contig00415 length=216 numreads=2

aagcagtggtatcaacgcagagtacttttttttttttttttttttgtcggaattaaccat

tatattacatcaattcatctacatacactgtacagtcatctaacaagtccttataaaatc

atcgctaaaaaaaacgacattcacaaatgttcacatgctaattaaaaattATATGCGCAT

TTCAGGAaCcGGATATCGGAaGTAAACCAAaTCCTT

>contig00416 length=240 numreads=3

aGGACAGAGAAGAGCTgAAGAAGCTtCAAAaGCTCCAGGAGGAGAGCCaGGAAgCCAAGA

ACGaCGATAACTATGACGACAACGTCGcGAAACCGAGAGAGAGGAAACGAAGAAGCTGAA

AGGGAGCaaagggaacgaaagaaatgaaggcaaagactttgaagactttaaagccggtaa

taaggcaggtggcaaggaatcgactaagttaaataaagaaagtctcaaaaagactcaagg

>contig00417 length=437 numreads=12

ccTTTTCTTcggCGGCACTACAATAgAGGCATTGTGGCAAAaCTTGCTCAGGTTGTACAa

GTCGTGCtCTTtGCGCTGGCGAAATTCCATGTGATCGTAGTTTGGATCGTCAGCCACTGG

AAAAGCTGTTTCCCCACCTTCCTCAACGTCGTTCAAATAATAGACGATAGTAATAAATCT

GCAAAGCTTGCAGTTCACGGGTTGTGAGGCCAGGTCTAAGTGGCAGCACTTAAATCCGGG

GTATtCCTTTCCTTCTTGACCATCAAAaTGAGCGTGGTAgTgACCGTgtGggTCGTAGTG

tAGcACTTGAAGTGGcTCCccTCCTTCGACGATCTGCCGAGGAAGACGGGTCAGGCGAAT

GACTCTTTCTCTTATGCGTTTCATAATGGAATCTGCAGCGCTCCCTTGCCGCAACCACGT

TTGCTCGCTGTACCGAt

>contig00418 length=464 numreads=6

aaagaaactgaggatgaagaggagcgcattaggaatcgcgatcaacacccaggcggctct

cggaaacgcaagtatctgcaagatttgttccggccgccagttgatatcttgcaccacgga

aactttgagtcggcaaagttcgttggtaagaaaagcaagaaGTGGCTACTTGTAAACGTG

CAAAAGTCGACGGAATTCGCTTGCCAGGTGCTCAACCGCGATGTTTGgTCCGaCTCTTTC

GTACGAGAGATACTTCACGAAAaTTtCCTCCTcTGGCaGaTttATATCGATAgTGaTGAA

GgTcAGCACtATAATAtGTTTTACCCTGTtCGACTGTGTCCTCATaTAGCGGTCATTGAT

CCCAGGACAGGTGAGCGTATGGCAGTATGGGAAAATCTTGgcACTAGGCcTTCTGCCGCG

CAAGTGTGCGAACTGATGACTCACTTCCTTGGAAATCATTTTGA

>contig00419 length=232 numreads=1

aagcagtggtatcaacgcagagtacgttcggggttcgccaaggcccatgaagccatcaag

agtgcttgtgatggcctgctgaaccctaacgccaatcaacaacagatcttgtcagctgcg

acggtaatagctaagcacacggctggcctgtgcaacgcctgcaagactgcatctactaag

acggacaaccctgtagcgaagcgccatttcgtgcagtctgcgaaggatgttg

>contig00420 length=524 numreads=5

cGAggAgACAATGACGAGAAGTTTCGTTTGTTTCCTGGACCCACTTTCCATTGCTCTGCT

CCTTTGAAACCACCATGCCAAATACGACTTCTCTGCCTGGGATCCAGGAAATAGAGCCTC

TGCcGCTGGAGGCGTcgtcgagggagctgtcaactttcatgcgcgaggcgtggcgACAGT

CGAGGCACGACGACTGCGAGAAGTATGCGATGAACGGTAGCTTCAGGTGTGACGTACTGC

TTAACatgtaaaccagcttcagaacgaccaaaaccgatgcagccagcAAACAAGCGATTC

TACACCTGAACTGCTGATTCCCcATGACCATGgCtCCTTcTTCGGAGATgAGTATCAAaG

GggggtgAGCATtCTtCCCTTACGGTATATCATTTTAAGCCATCTGTCCAGTATCCGGCG

CAGCCTCAAACTAGACACCACTTGGCCCCcTCCGTAACTCCGGACTCGCTTGTCTTTCTT

CTTGATTCGAAGGTAACGGCCACCCTACACGCTAGTCCTTAAGT

>contig00421 length=150 numreads=2

GGAGCAGTCGCAACGCACCTATTGGAAGGTGGCTTACCATCGTTGAATTCGCCTTATGCG

TTGCCTGCGCGACTTGGTTTATAAAATATTTGTCGGTGGTTGCGACGAAAATTGATAAGC

TGGTGCTGGTtACTTtATtGAAGGcagcag

>contig00422 length=1147 numreads=18

GTTTTCAAGTCCACCAGAAGACGCATCGACGATGGCCTTTCCTGGTTCACCGACCTCGCT

TGGACTACAAccGACTACACTTGCCGACAAGACAATGCTGTCCTTTGTATCCGGCTTCAC

GGCATTCGACAAAGTACGTTCCAACTCAGCCAACTCCTTGCTCGCGTCACTTCCGGCGGG

CTGTATGGCGCCTTTAGCAGCCATAGCGAAAgCGATGTCAAACGCACCGAGCACCTTAAT

catctcacactgcattcgcATTTTCTCTCTCAGGATTTTCTGCAGCGCACGCAgTTTCcT

GGCAAACcGtGGacAGtCCTCGATCATGGCAACGTACTTATCCAGAATGAGTTCGGCAAC

TGCGATCAACACGGGAGCGTAACGACTATCTgTGaTAAACTTGAaGACAAATTtCAAAAC

CGGcTttAACTTGGgaTAATCGCGGCGTGACAGCGCGATTTCCAACGCATTTCGCCGCGC

cAaCTCGGCGAAAATACTTATTACGAACGGCGCCCGgTGATCACCaCTCAaTGCCCTGTC

CAGCGCCTCGTGATaTTtAAACTTTCGCAGACACTTATCGACTTGATTCATTCTTTTGCT

GCTTTTACgtgCCTCCACTACTACGTCATCCTGGTCCGgCTGgTTATCCTCGCCACGCTG

AATGTACTGGTAGGTGCCTGGACGTGGCTTCTTCGTGCGCTGCtCCTTCACAGACTCTTT

TGCGgCCGgCcTGGCTTCGGACGGATATTGCACCGTCGGACATtCCGACCACAAGGTTTG

CGCCATCcGGAGAAAGACCGACAGCGAGAaCTGGTGATGGGTAGTCCAAACTCGCGACCA

CAGAaTActCcATGACATCGTAAaCCTtCAcaTGCCTGtCCAGTCcACCaGTCAAAAgcc

gcttttgttcgccgtcaaagcacatgcatgttattgtcttCTGATGATTAGAGAaCTGAc

gCTGCAGcgAaCCagAACCaCgAACATCCCATACACGAACATAGTTgccacctgaggagt

agcagatcgcgccagtttggatacgagcacactctccactggctgaccatgatccatgct

atagacttccttccagatcgggtgtcccaaatcttcacacagtgatcgtatgaaccggta

aaaaacg

>contig00423 length=241 numreads=2

gggAAGGGGACTTTACTTGAAACTTTCAGAAAGCGTTGTAGCTGCCATATGCTGGGCTGA

TACAAAtcgcccattccaacctgattgttattttcagttataagttctatccaaagatac

gttcacgaagaaacactagaacggctcattgatctgcaagcagacatctaattaaaaatt

acagtgccaatacctccataacgagccctttgttttcgagtcgagtttatagaatatcgg

a

>contig00424 length=242 numreads=3

aagcagtggtatcaacgcagagtacgcGGgggTTGCAGCCAGGCAAGGCTCTGCAAAGCG

TGTTCCTCCTCCAACTCAGGGAGATTATTtGGCAgCTAAAAAaCGaTtGTCTaCTCgTGa

TGgAGCTAATgTTAATAAGCTgCCTgTTtCTCGTGGGTgTGAGCCTAACCAAGCTCACAT

GAAGCAAGTAGATGAGAAGTGTATGGAGCAATCTTGTGGTGGCCAAAACGCTCAACTTTG

GC

>contig00425 length=241 numreads=1

tctactctggagaaagtcccaaagtggccaactctcaatatttggatacgtcatggggat

tgggatcaagcatgtacgagttgacgcgtggtgttgattgtcccgaaaacgcggtcttcc

ttgatttccacattttatggggctcgtcaaacccggccctaaagaagaattctatctgcg

tatttgagtggaacgccggaataccgtcgcgacggcatatagaaaacttgcctggatctt

t

>contig00426 length=278 numreads=17

aatctactgtgcacaagagatttagccctaccaccggacgACTGATGTTGCTGTTGACCA

TCACGCAGTTTCACTTCCTATACTATGCTTCGAGGACTCTTGGCAACGTATTTGCTCTTA

TTCTAGTTTTGAATGCTTTGAAaTCGTGGTTGGACGATCACCACTCGAAGTTTATATGGC

TATCCGCTTTtGCCATCGTGATCTTCAGATTTGAGGTCGcAGTCTTCCTAGGTTTTCGCT

TtCTAAtGAAGCTAatcAAGCGAAGgATAGGCATCTgg

>contig00427 length=1977 numreads=83

AAGCAGTGGTATCAaCgCAGAGTaCGCGGGGcTTCTGgAAGGTTcGCAGACAGCAGGCGG

TTCTTTGTGGCAGTATCAGGCAATCCTTtACGTCGGAGAAATAAATTGCACTCACTATGG

CTtCTTTAGTTTTAGGGGgCGAAAGGACCACTGgAAaaGATATCAGGgCGCAAAaTGTtA

TGGCTGCTGCTTCCATTGCAAATATCGTAAAAaGcTCCCTtGGCCCcGTTGGACTTGACA

AAATGCTGGTTGATGATATTGGtGATGTCAcGaTAACAAaTGATGGAGCCaCTatCCTCA

AACtATTAGATGTtGAAcACCCCGcTGCAAaaTTTTGTGTGaGTTGGCTGAGCTGCAGgA

TCAAGAAGTTGgAGACGGgACAACATCAGTAGTCATCATAGCAGCTGAACTACTCAAGAA

TGCTAGCAAGCTGATCAAGTaCAAACTTCATCcAACTAGCATCATATCTGGGtATCGCCT

TGCTTGCAGGGAAGCGTGCAAGTATATTCAAGATCAATTGTCTATTAGCACAACTGATCT

TGATAGAGAGGCCCTTGTTAACTGTGCAAAAACATCAATGTCATCTAAGCTAGTTGGTGT

GGATTCCGATTTCTTTTCAAATATGGTAGTTGAAGCTGCTTTATCAATTAAAAGGACTGG

ACTTAAGGGTGAGACAAAGGTACCAATCAAGTCGGTAAACATCTTAAAAGCCCATGGTGG

TAACATGAAGGAGAGTGTTCTTgTTCCTGGCTATGCTCTGAATTGCACCGTGGCTGCTGA

AGGAATGCCAAAGCGAATAGAGGGCGCCAAAATTGCGTTCCTTGACTTCAGCCTGCAAAa

GGCAAaGATGCATCTTGGTGTGCAGGTCTTGATCGATGACCCAGAAAAGCTTTCCGGAAT

CAGAGAAAGAGAATCCGATATCACCAAAGAGCGTATTCAAAAGGTTTTGGCGAGCGGGGC

GAATGTAATCTTGACGTCAGGGGgCATTGATGATTtGTGTCTTAAGTACTTCGTCGAAGG

TGGAGCTATGGCTGTTCGAAGAGTGAAAAAGATCGATTTGAAGAGAATAGCCCGTTCgTG

CGGtGGGAATGTGGTCTTGTCGCTGGCCAACTTGGAGGgCGAGGAAAGCTTTGATGCTTC

ACAGCTGGGTCATGCGGAGgAGGTCGCTcAGGAGCGAATATGCGACGATGAGCTGATCCT

TATTAAAGGGCCAAAaTCTAAGGCTGCATCCTCGATCATCTTGCGCGgCGCAAACGACTT

tATGGTaGgATGAAaTGgACcGGTCGATTCATgACGcTTtgTGTGTTGTTAaGCGAGTTC

TCGAGTCgAAGGCTAtCGTTCCTGGAGGTGGTGCTGTGGAAGCTGCCCTCtCAaTTTATC

TCGaGAATTTTGCCACCTCCCTGGCCTCTCGGGAGCAAATCGCTATCGCcGAGTTtGCCA

ACGCCCTaCTAGTCATTCCGAAAGTACTTTCTGTGAACGCAGCCAAGGATTCCGCTGACC

TTGTTGCCAAGCTGCGAGCATACCACAACGCTTCCCAAACACAAGCGGATAAAGCGCACC

TGAAATGGGTTGGTTTGGACTTGCTCAACGGTGTCGTGCGCGACAACAAGAAaGCTGGAG

TGCTTGAGCCGGCAATTAGTAAAATCAAGTGTCTTAAGTTTGCCACTGAAGCTGCGATAA

CCATCCTTCGCATAGACGATATGATTAAACTGgAGCCGGAGCAGAAAGATGATCCTGATT

CGTATGAGAACGCCAGAAGGAGCGGCCGGATATGAGCCAATCCTGTGGTTCGttCtGGAA

ATGACcAagTAAGCTTGATCCATTCCTCGGAaGTCGATtGTCTGCGTTTTTGGGTTCGaG

tGCTgTTTCccgAtgCcGATGCCGtCGAAGaTGcTgCGTGGCTGTACttACGAGCATGCC

tGAAAAAAAtCtGATCTATtCATTCCTAgCtgaCTATGAGATGgTTtGCTtatagaa

>contig00428 length=169 numreads=2

ACAAGAGACGTGCTGCTGTGTACGTCGACTTTGGACTCGTCTATGTTGATGCAAGAGACG

TGCTGCTGTGCCTGTGGACGTTTTACGATGTATATATAAAAGGGAGTTGGTTTTTagtac

gagttagagtcgaacgagaagagacatgtaaatattaatacgatcggtt

>contig00429 length=241 numreads=6

AAGCAGTGGTATCAACGCAGAGTATTTtCTTGGACAGCACGTTCTTCCTCATCTGCAGGA

GCGATTTGTTGCCCcGGCATAATAACCACGCCTGTTGaTCTCcTCTTttCACGCAGATGC

TTTCtAGCaGCTCGCTTTCGcGACGAAGTTCcAcTAGcTCcACcGtGtCtGtCCTTtttG

AaGaCTTTTtGAgtCAtCTCCTTCATCGTCATCTTCATTGTCTGACCCATCTGccctttg

t

>contig00430 length=431 numreads=14

ggaagtagaaactttaaacgacaaaatagtcgacactgcagacacacgcGCCGACACTGA

TTAAccgCTATCATCATCACTCTCCTCGATAATCTTCTTGCTTGATTTCTTTGAAGCCTT

CTTACCACTATCCCCTTCATcGTCGCTGGCGATATCAaGCTTtGCATTACGAAGCTTGTT

CTCGCTGTCACTATCTTCATCGCTATAGATTCCTCGGTTACGTTTAACGTCCTTCTTGTA

TTtGTTTTTtATGGCAGCGATACTTTCTTCGAAGTCTTGCTCTTCATCCATTCCAGCATG

TCCCTTGCCACCCTCCTCGTCCATATCTTGCTCCAGGTAGTTtGCGGTTAAGCCcTTGGA

ATGTCGCTTCTCTCGAATCCGTCGCTGTTGGCTCTCCCGCCGCATTCGCGCACGTTCGCG

CTCAtCcTcTg

>contig00431 length=240 numreads=2

agtgggtcgcGTAGATTgCGAaGAAGAGATCGCCGCTATCTTGTGAAAGGATGTTAGCAA

CACCCAATTACAGCTGCCAATCGTGTGACCAGAGCAAACCGGGATAACCTGGACCAGTCC

AAGGACGTCTAGTTTCTCGTCAAAGCCAACAGCTTGGACTTTGGAAATCGAATTTTTAAC

ATCCTGTGCTGaataaatggccttccatgaaactgaattagactggtcacacagagggta

>contig00432 length=223 numreads=1

gcaatcattacctcaagctcagcctcggcattgtcatcaaagttatttaagaacagactg

acatcaccagccattgcggcaacttcatctccactagcaagacgttctcgatcaagcaca

ataaatgtgcacttatcctcgtcttgccaccagctttgctgcatgcggtactcctcctca

agactcaatcgatacgaagcagtcttctgcaaagctcctcgct

>contig00433 length=241 numreads=1

atcagaaaccgtaaggatttcgtcgatattcaagattccctcgtggttttcaatctttgc

aataatcttgatgttctttccttgctcgccaagaacctggcgcatttccaggacgtctgc

ccgcttccgaataaaggacgcaaagatcatgtcaacattgttcttgacaccaaaaagaag

gtcttctctatctttttctgacactgctggcaagtcggcgacagctccaggcaagttgca

a

>contig00434 length=221 numreads=10

AAGCAGTGGTATCAACGCAGAGTgCATTCATTCACATCATAGCAGCTAACCCCATTCCCC

GTGAAGCCAGCATTACACAAACATGCATAGCTGGATAGCGTGTTCAAGCAGCGAGCATTT

ACGtCACAGACCGCCAGCCGCTCATCGCATTCATTGATATCTGAaCATCGCTTCCcATCT

CCTGAGAaGCCAGTCTTAcAACGGcacGCAAAggagccaag

>contig00435 length=293 numreads=4

tttCCAAAATTACTTGGAATAAATAATCTTCCGTGTTCTGGTCTGGGTTCGAAGTTGCCG

TAACGATGTCGAaCGGCGACCGGCCAGCCAGCATctCGTAGAGCAATACACCAAgTGCCC

aCCAGTCAACGCTGGgCCCgtaatcttcgccgcgcaggatttcgggggcgatgtagttgg

gcgtgccacagaacgtgcttgtcgtctctccaggtccgaggccctCCTTGCACATTCCGT

AATCCGTTAATTTGACATGACCGTCATTGTCCAGCAAGACATTGTCAAGCTTC

>contig00436 length=721 numreads=14

CAAGCcAGTtGTGAAAAAGGCAACCTCATCTACTCCAGgTGATCCAGATGAACCTGCTGC

AAAGCGGGAAAAGAGAGTCCCTAGCACTCGTACTAGAACAACTGCTGCTGATACTACCCA

GCCATCTCTCACATCCCCACCTCCAGTTGACaCTCCATTTTCTCCTTTGATATCACCACT

GgCTATCAAGCCAAAGGCTATAAGTCCAGCATCAGCTGTTGTAACcTCAACTGTGTCTGC

TCCAGCTACTCTTCTGCACTCCCCTATGCGAATGGTGAAATCAGGTGTCAaGCGAAAAAa

GGCTGATACAACTACACcTGGAATGACAGAGCCTTTGCTTCTGTCTCcTCTACCGgTAgC

TGCTCAGATTCCTGGAAGAaGAGAGTCATCaCATCGCACAATAAAAAAgCCCCTGAAAgA

TCTtCCtGttGACTCAaCtaCAAAaGTGATTATACATGGGAAGAAGAAAGGCAGACTCAA

TGAGcAACTTAAGTACTGTCaCAaCTtGCTgaaGGAgAtGTTCAACAAAAaGCAtCAGAg

TTATTCATGGCCTTTCTACATGCCCGTTGAtgCtgAAGGGCTtGgTTTGCATGACTATTA

TgATATAaTCAAGaCACCGATGGATCTGACTACCATTAAGAACAAAATGGAGGCACGAGA

AtACagTtCACCACAGGAGTTTGCATCAGATGTTAGGCTGATTTTtACAAACTGTTACAA

g

>contig00437 length=255 numreads=8

AAGCAGTGGTATCAACGCAGAGTGTGGTCTAAAAGCATGTCTTAAAAGTTCCGAATACTC

ATCAGAAATTTTTAATGGTCATATAATGAaCGTGTCGTGGAAGGTACCATGTGCTTGCCA

TGTAATTATTTGTTAATACCGTAAGTGGCAATGAGCGTGCtAAAATTCAGCTGCAATGGC

AATGTTTATTCATGCTTTTGATCATATATtCGACATTGTTAATTcGTTGAaCCCCtGTGC

CAGGGGgtttgaagt

>contig00438 length=1881 numreads=135

AAGCAGTGGTATCAACGCAGAGTACgCGGGGCTTTTGCGCAAGTTgCgTTtGGCCTGaCG

ACGTATtCacGAgCTGTGCTCAAGCgAaGCAAAAAGTaTTcTGtATGAGAAaTACAGTCA

AGTCTTCGAAATGAGTtCCGTGCtCGTGACCAGCCCCAATGTtCATTACACTGACcATGA

AATTCTCAGCAAGTATGTCTATCAGTCTACAAGAGTAAGCCGTGGTAGCCATGGGCAGCT

TCATGTGAAaCCTGTCGAAGTCGACGTGACCTTCAAAaCTGCCCGTGACTTGCCCAAaCT

CGGCGTCATGTTGGTtGgATGGGgAGGCAACAaCGgCACGACATTCACTGGGGCGCATCT

GGCGAACAAGCATAACaTCTCGTGGCAGACCAaGTCAGGCACCAAGCAACCCAACTGgTT

TGGCTCGATTCTCCAAGCATCAACTGTCAGCCTTGGCTTTGGTGAGCAAGGCGAGgagTT

CGTGCGCATGCGCGATGTGCTTCCGATGGTGCAACCCGACAACTTAGTCATCGATGGGTG

GGATATTTCCTCGCTTAATCTGGCCGACGCAATGCGCGAGGCGCAAGTTTTtGAGTTCGA

TCTACAGGAGAAACTGAAGCCTtACATGGCCGAAATGAAGCCTAGAAAaGCTATCTACAG

GAAAGACTTCATTGCGGCCAACCAGGAGGAGCGTGCAAACCACGTCATTGGCGGGACTGC

GGAGGCGCAGCTCAAGGCCATCCGACGCGATATCAaGgACTTCAAAGTCAAGCACTCCTT

GGACACGGTCATCGTTCTGTGGACGGCAAACACAGAGCGATTTTGcGAGGTCATCAAGGG

CATTCATGATACATCGGAGAACCTGCTGCAAGCCATTGCTAACGACGAGAGCGAGATATC

CGCCTCTACGCTGTACGCTGTTGCAAGCATTTTGGAAGGCTGCTCTTACGTTAACGGCTC

GCCTCAAAACACGTTCGTACCTGGAGTGATCGAGCTGGCTGCGCAGAACCGTGTTTTCTt

GGCAGGCGACGACTTCAAGTCAGGTCAAACGAAAaTCAAGTCCGTGCTCGTAGACTTTCT

CGTGAGCGCAGGCATCAAGCCTGTATCCATCGTTAGCTACAATCACCTTGGCAACAACGA

CGGGAAGAATCTGTCTGCCCCGCAACAGTTCCGTTCGAAAGAGATCTCCAAGAGCAACGT

CGTTGACGATATGGTGGCTTCCAACAACATTCTATACGCCCCGGGTGAGAAGCCAGACCA

TGTCGTCGTCATTAAGTACGTGCCGTACGTCGCCGACTCAAAGCGAGCTTTGGACGAGTA

CACGTCAGAGATCATGATGAACGGTGTTAACACGATTGTCATGCATAACACCTGTGAAGA

TTCCCTTCTCGCCACGCCCATAATTCTCGATCTCATCCTGCTTACCGAGTTGTGCCAAAG

AATAAAGATGAAGGTGGGAGAGCACGGCGAATTTCAGTCGTTCCATACGGTCCTGTCGAT

TCTTAGCTACCTACTCAAGGCTCCACTTGTTCCGGAAAACACTCCCCTGGTGAACGCTCT

GTTCAAGCAGCGCATGTGCATAGAGAACATCCTTCGGGCTTGTATCGGCTTGCCTCCGCA

GAACAACaTGTTGCTGGAgTACAAACTAGAAGACATGCAGGCAaTGCgcAGgAGGGAtgA

AGgCGgTGCATTcAaGCTCAATGCACGTGGaGAATGGCGAGGATTTGCACCAGAATGGAT

TTTGTCATAACGGCTATGTCATTtCcAAGAAGCACATGAACGGCTATGTCCACAGTTGAC

GCTCTAGACAATGTAGGAATTtCAGATGGTAATTTATATATGTGCGATGCAATTATAAAT

ATGAGCTTCAATTtGTAACta

>contig00439 length=826 numreads=18

ttCGAGGCCTCAAgATtCaGCTGCTTAAAAGGAAGATAGGCGGGACCCACCCCTAATTAG

AAGGGACTtATtATAGAATTTGCGAAAGATCGGCGTGCgTTGCCgTTAACTTGGACATTT

CGTACcAaCGAAATTtATAAGATTAGGACGGTGAGGGTTTATtGAATCTTCTTTTTCTta

TTTTAAAaCATTTGAGaCTtACGCCAATGGCTGAaTTCCTTCCcTCCAAATGCAATCCTA

CAGAATCCACAAACCCCTGCAATCCCTTGCTTGGAATCATCTGAGAACcTCTGAGGACGC

ATCTGAAATtCCTGGCGgTAAACATCATGATCCCTTcATTTCTAGCAAaCTTtCATCAGA

AAGGACAGCCACCTTCCcTGGGGCAAAAGCATGACTCCGAAGAAAGGCGTATTCCTGATC

ACTAGCAGGATTCCCGGCTCATGGgACTTGAACTTGCTATCATCGAGgTACTTAaCAGCC

cACTTCATATCATCATGCTTCGAGAACTCAACGATAGCAGTGCCATCTTTGTAAAcATCT

GCATAAAGAACCTCACCAGCctCTCGCATATGgTCTTTTAAATCTTGCCAGCTACCcGTT

GgAGGCAGGCCTGAAATGaGAACCCTGTTCTCAGATCTTCGTATAGGGGCACCTCGACCT

CGACCAGGAGTATAATTGCCACCACCACGAGGACCACCACCGAATCCTCTATCACGGTCG

CCTCTGTCTCCACGACTACCGCCACGTGGGAACTGAACTTTCAAGCGCTGACCATCAAAT

TTCTCCCCATCTTTTTCATACACAGCATCTTCTGCATCTCTTTCAt

>contig00440 length=1876 numreads=132

AAGCAGTGGTATCAACGCAGAGTACGCGGGGGAACATAAAaTGGCAGCGCCACGAGCGGG

AGGCTTCcGAATGCAGCCACAAATAATTCTACTCAAAGATGGcACAGAATCTTCTCAAGG

AATTCCACAGCTAATCAGCAACATAAAtGCATGtGAGGTCATTGCAGaTGCtaTCCGAaG

CAcTTGGGCCAcGTGGTAtGGACAAGCTCATtGTTGgAGAAggAGGtgCCACAACCATAT

CCAATGATGGAGCAACTATTGTCAaGCAGTTAGATGTtgTGcATCCCGCTGcTAGAGCCT

TGGTAGACATAGCTAAATCTCAaGATGCTGAGGTTGGTGATGGGACAACTAGTGTTGTAC

TTCTGGCTGTCGAATTGCTCACACAGATCAAGAGTCTGGTGGAAGAAGATGTGCATCCGC

AAGTGCTTATTAAAGGTTACAGAAAGGCTACCAATCTAGCGCTGCAGAAGATCGCTGAAC

TAGCTGTACACATCAAGCGAGACGATGCTGGCGAAATGAAGAAACTACTACAAAGGTGCG

CGGAAACGGCTTtATCCTCCAAGCTCGTTGCGAGGCATAAGGAGTTTTtCGGCAAAATGG

TGGTGGAAGCTGTTGCACATTTGGACGAACTGCTGCCCCTCAACATGATTGGAGTGAAAA

AAGTGCAAGGAGGCTCCCTAGAGGATTCACAGCTGGTTCTTGGAGTCGCTTTCAAGAAGA

CCTTCTCTTACGCTGGTTTTGAAATGCAGCCAAAGAAGTACAGCAAGCCGAAGATTGCCT

TACTTAACGTAGAGCTGGAACTGAAAGCTGAGAAAGACAACGCTGAAGTTCGGGTGAACA

GTGTTGAGGAATATCAGAAGATTGTTGACGCAGAGTGGAGTATTCTCTACGACAAGCTAG

ACAAGATCGTCAAGAGTGGTGCCAAGGTCGTTCTTTCCAAGCTTCCTATCGGAGATGTGG

CCACGCAATACTTTGCGGATCGTGACGTCTTCTGTGCTGGTCGAGTTGTGGAGGAAGACC

TTAAGCGAACTATGAAAGCTTGCGGTGGCTCTATTCAGACGAGCGTCCAGTCACTCACGG

AGGACGTCCTGGGAATATGCGACGACTTCGAAGAAGTGCAGATAGGGGGCGAAAGATATA

ACTTATTCCGGGGTTGCCcGAACGCGAAGACTGTCACACTAATCTTAAGAGGTGGCGCTG

AGCAATTCATCGAAGAAACCGAGAGGTCTCTGCATGATGCCATTATGATTGTGCGAAGAG

CTATGAAGAACGATGCTGTGGTCGCTGGAGGAGGCGCTATTGAAATGGAGCTAAGCAAGC

ATTTACGAGAATATTCGCGAACGATAGCTGGCAAAGAGCAGTTGATCATAGCTGCTTTCG

CCAAAGCGTTGGAGGTGATTCCCCGCCAGCTCTGCGACAATGCAGGATTTGACTCCACTA

GTATCCTAAATAAACTCCGACAGAAGCACGCGTCTGATGGTAAGTGGTTCGGAGTGGACA

TAAACAGCGAAGACATTGCCGATAACTTCAACGCGTTCGTTTGGGAGCCTGCCGTAGTGA

AGATTAACGCTCTTACCGCTGCCTCTGAAGCTGcgTGCCTCATACTGTCCATAGACGAGA

CCGTGCAGAATCCGAAGTCTGACACAAGCGGTGCGGACAATCCAATAaCaGGTAAACGCG

GAATGGgTGCCGGTGGTCGGTAGTGTCCTAAGTAGCTCGATTGCTCCCGCTAGCCAACCT

CAGCcGCTCATGAAGGCGTTTCGTAACGCAAAGCTTCGTCTCCAGAAGAACATTGGCcTG

TATTTGTTTGTTTGTTtCTTtGATTTCTTGTCTTAGAGAGCATATTTGGGTCAGTCcGTA

Acaaaaaaaaaaaaaa

>contig00441 length=415 numreads=4

AAGCAGTGGTATCAACGCAGAGTACgCGGGGACGGATAAGTCTCTTGTTTATTATCTTTT

GAGAGAaCTAAGGATCTGCAAGAATATAATAAGCCAGAAATGCAATGCATACGATGACAg

atgtcacaatcttggttaggtcaaagcgaatataaaaatcgctgtcgagaagtcttcgtg

cGGGTCgTTTGACAGAAGTCATCcTgCAACtCTTCAATTGGAcTTtGTGAAAGAAGGTCT

TGCTCTACATAAAGATGAAGGTCTTCtttGCCATCCTTTCATTGGCTGCGTACGTAAAAG

CGATCTCACAAGACCAGGTTCGATGCACTGAAACGTCTTTGGTGTTTACTGGATCtcaag

cagactttcccaggtatacaccaaacttaataactcttggaacgtgtcccctggg

>contig00442 length=229 numreads=6

AGGCcTACACGTATGTTCATAGCGGCCAGCTCTGCAACTAGATACTTAAACACGTAGGGA

aCTAAAaTTTCTTTAACTTCGCGGCCATGTCCGCAAGTTTTACAAAGCCACTGGCGCTTC

TGCGAAACTGATAAAACGTGCAACTCAAGCGGCTGCTTCTCGAAAACGGCGATATAAGGC

TACCACACTTTGGCACACACGGCcAGTGgTTgCATCTGAgcAATGAAAG

>contig00443 length=241 numreads=1

aattatagtagctccattttagagcgagccgcgtcttcagtgttaagttcgagtagcttt

ccaacaagtctcctactattatgtcattgtatttgctgctctccgatctaataatctggt

tgtccgcctggtcagatgaatgaccgaggacaaatacagtcttcacgacatcacgcgcgc

tcacgcggatatccttttgaaatgcggtctgcctcttcaggtgcaccgcgtcatctaggt

a

>contig00444 length=425 numreads=4

cttgagaaggtcggaataatgctgaagccgttcttgccgcagttacagacaactttcatt

aaagctttaaatgacccaacccaagcggtgcggcaaaaagcagcttgggcccttggcttg

ctgactgtacttcatACGAGGGTAGACTCGCTTTTTACTGAGCTGAAAAATGGTGTATGC

AGTAGCGAGGATTCGGCTGTTAGGGAAACTATTTTGCAAGCACTtcGGCGAATAACTTTA

AATGCTGGCAGTAAAATGAGTGAGCCAATTCGCAAAAACTTGCTGGAGACACTTCTTCAG

TTTATTACATCACCCGAGgATTGCTTGCGTGtCaGTGCTGGCGGTGCTCTGGGCGCCCTA

TgTCAAGTCCTTtCTGAAGTAGAGCTAAAAGACCTCCTTCAAGAACAGCTGCTTTATTCT

GATTc

>contig00445 length=645 numreads=40

ctCTCCGAAGAATcTCCCATGAAGGAAATGCGTGACgACGGTCACGATGGCGATGACGGT

GACCTgATTGATTCCGGTGCGCGTGACATGGAGACTAGTGGAGGGgCAGCTGTCATGTCC

AAACGCAGCCGTGACTCGATCCGAAACCATCGTAAAGCCGAcAAaCGAAaCGTCGGAGGT

GATGACGGTGACGATGGTGACGTgATCGAATctGGCGCAAGGGACGAGCTCAaGAAAGGT

GCAGGTGGAGACAaTGGCGGTGCGGCTGGCATGAcAAaGCGTgCCCGTGACCGAGTCgaG

GATTTCGGCATGCGAAGCAGCcGTGAcgAGGGGGAGAATGGAGAAGTTACCGAGACCATt

GACCCTGAACCcATTGACAACGAAagTCCCGAGTCCGACTCGGGCATgATGAGGATGTCC

AAGCTAaGCCGTGATGATGGGGACGGTGAGAATGGAGGCGTTACGGAGCCcATTGACCCT

GAACCCATTGACAACGAAGGTCCTGTCTCCGATGACGGGGCGGgAATTAATATTCCAAAG

ACAGATGGAAATGCACTGGACAATATCATTTTGATTAACAACAAGGCTGGTGTTGACGAC

AAAAAAGTCGACGAGGGCGACATCAGGATACCAGACGGCGGTGTg

>contig00446 length=270 numreads=6

cTGGCAGAAAGCGGTGGTCAtCCACGAAATCGCTCACAGCTTAGGTCTATATCACGAGCA

GAGCAgACCTGATCGCGACGCTCACGTGACcATCATATGGCAAAATATTCAACAAGgAAT

GTCCTaCAATTTCGACAAGcagCCAGCGTCCTCGATTGATTCcAAGGgTACGCcGtACGA

CTACAgCAGCGTCATGCACTACGACTCGACGGCCTTtGgAGGCGGTAGGCAAaCTATTcg

aacgaacgacccgagcaaacaaggtctaat

>contig00447 length=476 numreads=6

ggttaatatcaagtactagttctccttgttgtcaaaggagaagacagaccttgttgaGAA

CACAAGCTCTCTGCCTGGGCCGGTCACaaTAGGAGAAAAaGTTAAACAAGCTGGCAAAGA

TATCAGCTATACTGGTGTTCTCCTGTTAGGATTTGCCGTCACAGGTGTCCTCGCATGGTA

CGTTGCCAGTGAACTGCTCTTTGGCTTTAGTCcGAATGCAGTTtACACGAaGGCGTTGCA

GCTTGTTAAGgATAGCAAAAAGGCTCACCAaCTGATTGGTAGTGGTttGAAaGGTTACGG

GGAGGAAaCGTCGCGAGGCCgAAGGAGGCATGTgATTTCCCAaGaGTACGTCGTtGACGG

CGTGAATCACATGCGCGTTCAATTCTACGTTtCTGGAAGTGAAAGAAAaGGAACGGTTCA

CTGCGAAGCTAAGGAGaTTGgACGGGGGAAATTTGAATTCCGTTACGTGTTCGTCg

>contig00448 length=186 numreads=3

cgCGCTCcTCgatcTGCTGgTGGAGgAcTTGCGCTCcACgCaGTCTgTCgtGACGCTtAg

CATTCTCCCGTTCTTCGTACtCcTGCaGAGCTCGCAaTcGATCGCTCTCCaTCATTtCAt

CAAGCCTTtGtCTTCTtCGAGCATCTCTctCTTGATCTCcGATTtCTCCGCaagctgtgc

gtcacg

>contig00449 length=241 numreads=1

acgacgtcttatagatgatccagagacctcatccgtagaagacagatctttgatttgtgt

cgatgcattaatcggcaacgcctcggggccaggttttacatcacgtttatcctccttgtt

atcaacatcgcctaaacccttctcgcgttcgctcgttttctctctctgatttctaagtct

tcgttttgatccctcgtcgctttcgcgtttatcgagcttggttggccggtcctcggattt

c

>contig00450 length=1379 numreads=40

gCAAGCCAgTTTTTTTGGGCGAGgAATCCTAtCTTgAgCGATTAAAGCGtAAATGGATAT

CTGCTGgCCTTGAAGGAGTTCTTGGAGAAGTTTCCGAaTGGAATGAGCTGCCCCGAaTGG

TtCGCTCAGCCAGCAAGTTGTCGgCGAACaGTGTgTCTGCCCTGAAGACGTTGCTAAGCA

AAATCGGAACGACgTTtGACATGTCcAAAagCGTTGTGTTGAcATCAAATGAGTCGCGTC

CgaGGAAACTGGCTaGCCATCGACATATTCCTAAAATAGCCTTCGcGGgACAGgACGCTA

CTGTGACGATCATCTCTCTGCAAGGATTAGACCATGGCGATGTTGTTCTGAAAGATAAGC

GGCAGAGGGGGATTTCATGTCTAGAGTGGAGGCCCCTCTCTTCCTTGAGTATCGCAGTTG

GCGGTCaGgATGGAGTTCTTGTTTGGCTCctAGATCCGAAGTCCACTGCTGCTAGACCAT

CCACTACTATGACGCGGTTTCTGCCAACGCCGTACCCCGTGATCTCGATATCGTGGAAaC

CCGaTGGgCACCAGCTTGTTTGCGCTTGTGAGAACTCATCTTCCTTCATTGTGTGGGACA

TCTTAACCGAAGAATCAACTTCGgTTCATAGACTTGGAGCTAACcTCGTTGAtGTATCCT

GTtCCCCCtGcGgcTCCAGGgTtCTCACGACCTCTGCATCGAACATGTTCAGGGTGTGGG

AAACTGAGAGTTGGCAATGCGAGAAGTGGGGAAATTTGAACGGCTATtGCACGTGTTCGT

GTTGGAGTCCATGCGGAAATGTCCTCTTGTTTGCCGTCGCTGAAGAAAGCTCCATATACT

ATGCGCAATTCTTCCCTCGTAGAGATGTTAAATCTACCATTGACCTGGTGGGTAATGGAG

TTGCAAGCAAGTGCGCTGACACTTTGCCCcACACTTGGCAAACGGATCAGGGAGAaGTGC

GAGTTGGCGGTCGCATATCTGAAATGATTtGggATCCGACGGGGTGCAGGCTTGCAGCCA

TGTTCTCAGAACCACAGCcGTATGTTACGTTAtACAACACAAGGgTATCGCCCGTTCTCC

AGCTTATTCCGTGTGGCTTCGTCTATGGGGgATCGAGCGAAATTCCAaGgAGCATcGCcT

TCGTGCACGGCTATGAAAAGGGAGCGCTTTTGGTCGTGATCTGGTCTAGTGAGAACCTGT

CCGCTGTACCTCTCGTCTTTAAGGAGGTGTCTTTTTCGGAATTCGGGAgTTTaGTtCCCg

CGATGAAGCAAAATCCtcAcTCCATTTGCcAAGGCaTCTCGGAGTCAGCGAGACTTACTG

TATAGCACTGTTGATAGTTAGCAACACTATATAGAAGCAACGTTTTGTAaCaaaaaaaa

>contig00451 length=1259 numreads=58

cACTACTGCTTTCGAGGAcccAAaTTTCCAGGCCGAAGCTGCAAAAGCCAGAGACGGAAG

CGTGCTTTTCAAaTATCTTAACGATCATTTCCAAATCACTTCAgACGACCTCGCGACTTt

GTACCGTTTCGCcgAAGTTCCAGTACGAGTGTGGCAACTACcTGGGTGCTTCGCAGTACC

TCTACTTCTACCGTGTCCTTGCCGGTGACAATGACTCGAACGTCACCAGCGCGATCTGGG

GCAAGCTCGCCTGCGAGATCCTCAACCAGAACTGGGACGCGGCTCTGGAGGACATCAATC

GACTAAAAGACATCATCGAGAGCGACCACTACTCTCCGCCgTTGCAGCTGCTCCAGCAGC

GAACGTGGCTCATCCACTGGAGTCTGTTTGTTTACTTCAACCATCCGAAGGGCCGCGATC

TGCTCATTGATATGTTTCTCTATCAGCCGAACTACTTGAACGCCATCCAGACGACGTGTC

CGCACATACTGCGCTATCTGACGACTTCGGTGATCACGAACAAGCGCAGGAAGCAGATCC

TGAAGGACCTCGTACGCGTCATCCAGCAGGAGGCGTACACCTACCGCGATCCGATCACCG

AGTTCCTGGAATGCCTCTACGTCAACTTCGATTTCAACGGAGCTCAGCAAAAGCTCGTCG

AATGCGAGACGGTTCTCTTGAACGACTTCTTCCTCGTCGCCTGCCTGGACGACTTCATCG

AGAAcGCGCGCTTGTTCATCTTCGAGACgTTCTGCAGAATACACCAGTGCATCTCCATAA

CCATGCTGGCCGAGAAGTTGAACATGACGGCCGAGGAGGCCGAGCGATGGATCGTCGACC

TCATCCGCAACGCCAACCTGGACGCTAAGATCGACTCAAAACaGGGTCACGTGGTGaTGG

GCACGCAAGCCGCGCAGGTTCACGAGCAGGTGATCGAGAGGACGAAAGGCTTCGTCAGTC

GgACGCAGTTgCTgcTCTCAAAGaTCGACcAGAAGCTCGCtGCtAGGCAAcaacagcctt

acacagagccTTTCGCCATGGCTTGGGGAaGTTAAcGCGATGCTGTCATTGACGGCCGAG

gTCGACGCGCAGACTCGAAGCGCATtCTCCGCAAGTTGAGCGACCGTCCAACCGGGTATG

GCGAACGTCAACTCCGTGGTATTCCTCCcGTAGTGGAGAGTTACGGAGGCTGGCGAaGAT

TAATGAAAAATGTCGGCAAATACgTGTTCGTgTAaGAAaCATAaTgTATGTGTCAgCCA

>contig00452 length=241 numreads=1

gcaagcctcgcacaccgttctcgactacccagctgctgacactggagcagaaatttaaaa

ggaagcagtacctgtccatatctgaacggtctgaattaagcgaacagcttaagttaactg

agacacaaataaaaatttggtttcaaaatcggcgcgctaaagagaagaggctgaaggaag

ctgagatagagaaggaaacgcgggtatatcctcttccacagccgggcagtgcaacttgcg

g

>contig00453 length=249 numreads=2

CAACACGATGAAGATGCGGACGTTGATATCGCGCGAGCGAGTGGAGACCGTCGACCAAAG

AGACGAGCGGCTCTAGAGGCTGATCTCAAGAGAATAGCTTACGAACAGTTGTAGACGCTA

TCGATGTGAAATCTGATCCAGGGCGGCCGTGTGTCCGGAAAACTAGTTAAGCTGGATCAG

CTCAGCGGCCGCGCTTGTTTTTAATTGGTTCCTTGCGGGGGCCTGCCTGATTGaaacttg

gagacaccc

>contig00454 length=1168 numreads=19

aaGAAAGGagCTGATtGCAAACCAGCTGGAGAGACGAAGAAACCCAGGCTTAACAGTGAA

TCATCTTCTGCGCAGCCTTtGAGAGTTGCGTACAACATACAAATGGACGATGAAAAGGTG

CaGAAAAaGTTGACTAAGAAGTTGAAGAGGCAGCAGGTGCCCCAGAGGAGCCTCGTTCAG

AAGAAGGTTCATTTGTTTTCCCATCTTCACcAGTACGAGAAGGATGCcTCTCTTACGAAA

GATACCAACcTGTGTGCGACTATTcATCCTACGATCTTGAAGCTGGGCTTGCAGtatgca

gagggcactgtgaccggctctaatgCCAGATGCaCcGCGCTCATTTGCGCATTCAAGAAG

GTTATTGCAGactataccacgccgccgtctaaagagctgtcaagagatcttgaagctcga

atcaagccttatattagttttctaaatcagtgccgccatttgtcggtcagtatggggaat

ttcatcaagtttttgaagcttcaaaTTAGCCAAAGTTACCCTGGCTTGCCAGAAAATtGA

GGCAAAAGATGCTCTATTGAACAGcGTGGgAaGCCTACCTGGAGGAGCGAATAAAGATTG

CTGGCGAGGCCATTTCGAAAACTGCAGGCACTAAAATCCGCGATGGGGACAAAATATTAA

TCTATGGATTTTCATCGCTCATCATAAAGATTTTAACAGACGCTTTTAAAGAtggaaaga

agtttcaagtaattgttgttgattctcgaccacgcttcgaaggcaaagagtgcgTTCGTC

GTCTTGCAtCGCTTAaCATAACGTGCAGCTATGTGCTGACGAATGcTGTTTCGTACATTA

TGAAGGAGGTGACCAGTGTCTtCCTGGGCGCTCATTCGCTGCTGGCTAATGGTTACGTCA

TGgCTCGTGCcGGGACTGCTCTCGTCGCTATGGTTGCAAaGGCCcACAACGTTCCTGTGC

TAGTCTGCTGCGAAACATATAAaTtCTGTGAGCGCGTtCAGACCGATGCATTtGTCTtcA

ATGAACgtgggcgatccagacgctctcgtggatcttgattcggttcgggggccgaactcg

acgcagctgagcgactggcgcgacgttaagtccctgtacgtcttgaatttgatgtacgac

gttacccggtacttcggtcgacttggtc

>contig00455 length=203 numreads=3

AAGCAGTGgTATcAACGCaGAGTACCCtGTTGTTtCCCCCTGCGTTCTCTGCtACATGCC

cTGTGTaCCcAGCTAGTtaCCCtATGTACCcAGcTAGTTACCCtGTGGaCCcTGCTAGTt

GCCctGTGTACTCTGGCTAgTAGCCCTATGTACCCTGTTAGCgCTCcTGTGTACCCTGTT

ATTtGCCCTGCATAcccagctag

>contig00456 length=183 numreads=3

cTAAGCAAAaTGAAAGtGAAAGTAATAGTtCGAAaTCCTTCTGAcTATGtCaGAGGaTCA

ACAAaaGAAaTAcATAGAGTTGCACGTAATGTaGACCCagAGATCCACCCTTttGAGgCT

gCTaGAGAaTACACACGGGCCCTCAATGCTGCCAAGCTTGAGAGAGTTTTTgccaagcca

ttt

>contig00457 length=2066 numreads=38

AAGCAGTGGTATCAACGCAGAGTTACTTTTTTTTTTTTTTTTTTTTTGTTaTCAATTTGG

ATCTTCGCTGTTAATCTCTGAGCAATTAAAAAAAAAAgCAGGaCtgAACCTgTTAAaCAA

AACATTGCATCGATTAGCCTGAAAAGAAATTATCCATACAGACATACATtCGCTAAATAC

TGCAAATTTGAaCCGTCcACAGAAAGCAGGCGATTCTTGCTCTTCATCAGaCaGTCAtAT

TTCGACATCTTGCTCAATGAaCACAcATCGGTCGCATCAaCcAAaCCGTCTCATGATTTT

CcTTCCAGATACTATTCGACACGCTGTCCGCCCATGAATCTTtCTCCAACGCGATCGCAA

TGACAAGATTtACCcACCCCCGATCAGACGCTCGATGGCAGCATTGACGTCACCCCCGGT

CGCCATTAAAGCTTGAATGTTTGCTGCTCGGTCAAGAAAGCCCATCGAAGCCAACTGGTC

AAGCTGGACTTGAAAACGTTGCTCAGGGGgcAAaGTCGACTGgCCaCCCGACTGGCCAGT

CATGGAGGACAtgaagttgcccaagAGCTGGTTTAACGGAtACCCCCGGCAGGAGAGCTG

GCAGTGGGCGTGCTGGTAGTAACGgCAgCcGAaGatGgaaCaCGGggAAATCcAGACAAA

CCAAACGTAGGCAAGaGCTCCGGCGCCTCAGTTTGCAATGTTtGAACGCCTCGCATGATC

TGATCTATGgCCTGgATtACACGAGGATTCGAGGACGCcGACATAAGTTGGGgCATGTTt

ACTCTTTCCATTAGAGCAGGCATTTCTTGCGCCAGCTGCTCTGCCAGCTGTGGATTGTTC

GCGAACATGGGATGGTTGCGAAGAATTGATGTCATCAATGCAGGATTTCTGGCGAACATT

TGCATCATTGGCTGCATAGACGCTACATcgtccAcGTTCGGCaCTTGTCCCCGaCCTCcT

tGCAtCTGAGACAAGAGGTCCCGgAATGcGGCAGGTCCcgCcTGACCAGAACCAGAaGaG

GTGCCACTAGTTGCACCGCTGCTAGCcGATGCTGGAGTGCTTAAGCCAGGAATCGAACTG

AATAAACTGAATGGGTTCTGAGCGGAAGGAGTAGCCCTGGCGGAAGACGCAGccGAGGTg

ttAGAGGAAGAAGGCCTGGTTGGGGTgCCACCCcAAGGATTGGgCAgAGGATCGACATTC

TCGgCGCCtcTTTgCGgATTcTCCggcGTAaCcaCAGCGTCTGGTCCTCCTGCTTGCCTA

TTtCCAAaTTGaTTtCGAATTTGCTCCTCTGCGGCATTCATCATTggCTCCTGGATATCC

GTGTATAATCGCCGCAAaGCGTtGAAGCCACCCGGCAAaCTCTCTACATTGCTgAGGGCC

CgATCTTGAGTTCGCATCATTTCCTGCATCATGGCTGGGTTTCTGATCATTTCCAAaGTC

TGGCGGAGAGTTtCCGGGTTGTTTAGAAGATGCCCAATTTCGGGATtCCTCTGAATAACC

TCTTGCATTTGCGGGTTCGAGGTCAAAATACCCCGCATGACCTCAGGATCGTTCATGATG

GCTtGGACAaGCGGGTTGTCCAGCGCCTGGCGCAACATcTCCGGGTTCTCCATCATCTGC

TGACGCATTTCCGACATCACAGGGTCACTtAGCATTTCACCAAACCCACTGAAATCCAGC

CCACCACCTCCACCTaaGCCACCTAAaCCTCCTGCAGTCGAGGaTGGAGCCGAAgCTGCA

GCATTCGAAGGTGCAGCAGAaGTCTGGggtAcAACCGTGCTATGGCGAGCGGCCTGCTCT

TGAGCCTTGTTCTCTGACTTaatGACAAGaTGTATGgTGATACCATCTTTAATACCTTCT

GCCTTGaCAGtATCtGGaTCTTtCAAAaTTCGgCCGGCAAAAatCAGACAGaGCTGTTCC

AAAGATGCATGACTAAATTTCTTTTGAATTTCCTGACGAAGCTCTCGAATGGTGGCATTG

TGTGCCACTTCAACTGCTTCCTTGCCGGTCGCAGTTTTGACTGTGATATTTATACGACCA

GGCTTTTCTtCCTCCGCCATATTGCG

>contig00458 length=236 numreads=10

AAGCAGTGGTATCAACGCAGAGTGACAAAGTATGTAGTGCGCACTTTGCAGAGTTTGACT

TCACAAAAGGACTTGAGAAACCcATGCTGCGGGAAgATGCAGTACCATCAGTCTTCCcAG

AATACCCTCAAaCTAAAAAGCATCTGCCACTTAAAaGGCGCTCCTCACCTATGAaGCACA

GATTGACCAGCAATATCCTACCATCTGCTAATAaGCCAGAAgCAaGAGTCGCtcta

>contig00459 length=243 numreads=1

atgttaattttctgcagatagacatatgctctaatagtggattaatttcttatcttcgaa

acaagaaaattattgatactgtggtaatgaatccacctttcggaacgaagcataatcaag

gaatcgacttgtgctttgttcagcaagcattaaaggttgcgagtggtgttgtgtattccc

ttcacaaaactgctaccagggaccatatattaaagaaagctaggaaaaagattggaatgc

aac

>contig00460 length=244 numreads=2

CAGAAGTGCAAATCCTTCAATCTTAAGCGCTTATACTTTTCGTGTCGTCCCATGAGCTTG

TCGTTGCATTTAATTTTTTCTTtCAATCTTTtgACGTCATGAACGAATCGGaagtcttcc

gatttaccccacaatatgactcggaggacatttcatagcctatgtgcgtgggcatcatca

tcatcgcgaaactttcctttgcgacgctgggtttgcggtatcggcgaggatcaacatcag

aatt

>contig00461 length=241 numreads=2

AAGCAGTGGTATCAACGCAGAGTAATACCGGACCACATGCTGGTGATGGCCTAGAACAGC

ATGTGCCcATACCTCCCGAAgagccatttgctcatcaaccgaacctgccaacggctttaa

cgactttttaagggcataaatgcaaccatctagcctattaaggcacttgtggacagaacc

aaattgccctgagccaattcttttgagctcaagaaattctgcatcaaagcgagatatgct

a

>contig00462 length=229 numreads=2

GACAGCAGAGCGAGATACATTGATTGTACGAGGGAATTCGCCGGTGAAGGTCTTAAATTC

CCTTGTCGGAAGAATACCGGACTGCTCGGGACCGATGCATGCGGTTGTTTAGTAGCTTTT

CTCCTACACAGTCGAAAGAGCGGATCTCTGTTGAGGCTATACTGCCCACTGCGGACAccc

caaactcgtcaccgctttcaggaccgagtcatcgtatgcccatagcacc

>contig00463 length=801 numreads=15

gTGAAAGTACCACACGTAGAACTGGTAGTGAAGCGATCGGGCAAAGCACATCCCAATGAA

aTtCGACgAAAAcAACaCGGCCAcAAtGGTCcTtGgtgAAACGtcGCcGTTTTGAgcTTT

CAGACTAAGCAAAGACGTCACACCACcACgTAACCTtGGCcATTTcTTGATCAgaaataa

gcccaagacggcaaggtgacacacgaggaGGCAAaCcGTGAAATGCCCGACTGAGAAAAA

GCCACTCggggACGAAACGCCAGTTCACTGTCCAGACGTAGAAGAACTGCCGAaCCTAAG

TTGAAGGcTCTGATCAGAtAACCGACAGGATTGATCAGAAGAAAGGGCACAGCCACGAGA

ATCTGAaTGCTGCcAcAGATGACGAGATGCTTtAAAGTACCCAATACGCCAAACTTCAgC

AGCaGCAGGTaTAGAAGCCCGGgAGCAAACAAGAGGATGTTCATCTtGATGCTGACGGCT

AaGCTAAAAAaCaCACATCCAGCACTCcACCGCtCCGAGATAAACAaTGCGATGcTtACG

TAAACAaGCAaCATGGCCACAGGGTCGTTGAAGAGTCGTAGCACGTAGATGGAATGGATG

CGGTAGGATGCACAGCACATGAAGAATAAGACGTACGGCtGTACCAGCTTGGTTTtAtGG

TAGATGTAGAAGACTACCACTATGCTCAGCATGTAGAGAGCAGCAAAGaTGTACTGGgCC

ACTCGAaTGTTTTTCCCATGATCAGTgACATAATACAGACcAaGAAAAAGcGTAAaCAAA

ACCTGCTGGATACACTAACgg

>contig00464 length=268 numreads=7

AAGCAGTGGTATCAACGCAGAGTACGCGGGTTCCTTATATACAATTCTTAATAAATAAAG

CTTtCATCCATACATAACACCACTAATTATACTTGCCAACTGAACTAAAGGGGGCCTTAG

TTtAAtCATAAACcAGACCcAATAAGAAGTCCATTATATAAAGATTGCGCAaGGTACATG

CGCTTGCGCAACCATtGTCCTTTCAGTTAAATaTtATCATtCtACAAAAAGGTCcTTAtt

cAatcGTTGGAATTCACgattttgcaca

>contig00465 length=240 numreads=1

ttccacgtagttaacagggaagaagccacgcctgccatgcacctctccctccaaccagtt

atcgtcgatgcggttcgttaactttatgatatcaccttcactgaaacccaactctccctc

gtttctggttcgaaatcatacaatcctcggcaatgtgggcttcgcaacgccgcagccgaa

aacgcgggctgcgacacagcagcaggtgctgacgagaaggcagaaacctcttcgtatgaa

>contig00466 length=236 numreads=1

tctcgaaatgtagaacccgtgacaccaatggggcattcgttggcattttgctgcatctca

ccttctcaagcaactggttcaaatcgctcaagtcttcatagattacttttacaagaaact

ccctcctgtcttctttctccgtcagcaaatattcacgcaagtgaaagagaagtgctatga

tcttattgctggctgtccgcatccatgcatcttcaaatccacatgagtctctccaa

>contig00467 length=536 numreads=7

ttttttttgttctgacTTGACTCCATTCCAATTCAGTTCACATTCAAAAGTCTTGCAACT

TGGAACAAGATCTTCATGTTCcTTTtGGTCGCCCTCTCCCTCTCATCGGATTTCGAAAAC

AATATGGAAACCTtGGgaaGgAAGACAAGACACAAaGtAaCCGtGGTgCAAGTCcaCacG

CACATGGACAGCAaCAAaCAAAAgCGCCTCGAATCGATgcgcgccgttgagggctggaac

ggtgatgacagcgagCAAGCTGGTGATGCTGACTAAGTtAGACGGAAAATCCAATCTGCT

TCGAGTCGTTCAGAACCTCGATCTCAaCCTCGCGCgTCTGCCACGCAaGgAaGATACCGT

AGATCAGAAAAATGCCCTTGAACACCGCTATAGTGATCAGCAAAGCTTCCTGATTGTCAC

AGGCACAATCCTCAGTTAACGTTATGAACTCCTTGTCAGCAGTGATaTTGATCGTTctGC

TTTATTTCCTTctGAaCGCGgCcAGcGgcAaGagCTGACCcAGaCTGTCAAGAATa

>contig00468 length=241 numreads=3

aagcagtggtatcaacgcagagtAGGCTACTTTATcccTTCCTTCAGCCGAACACTTTTC

GGAAAGTGActacatccctgctgtttatagctattactctcgcaaccggaattgtttgga

cgggtacacgtctcagcgcagttctgatccgcccggcggtgtagtaggtcaatacggtag

cgacaacgagttgtcgcatGACGACAGTCTCGAAGAAGCCGACGTCGGATTTTGCAAGCG

T

>contig00469 length=240 numreads=2

agacaagggaaaagaaagagcagagaagatgcagggctttttcgacaatctggaggcaaa

gtactgtcagtcaaagaagaaggatgtcaagaaagcactggaagaaatacagagaacaaa

ggCAAtCCTAAAATGAGAAAGCGTTAGTCTAATATGGCAGACAAAATCGTTAGCTGTGCG

GTCTGTTGCTTGTGATATATTTGTGATCGTTGCGTCTACGGGAATATGAATGTGTGCATG

>contig00470 length=239 numreads=3

AGTGAAACGGCTTtGCCATCcTGAGCGtCGCAAAGACTTTaTTTCCGAGACCCATTTGTA

TGCTCTGGGAAaGTgTaTCAACATGTTTGCAgTTttAagCTCGCTGAAGAATATGAAAGC

TtGTTtGAATAATGATtATGCATTTATAAAAGGGCTGAGACATTCCTAAAgCAGACAGCC

GGTGATGCTCAGGCTCTTCAAGAATCTCAGACCTTGACTATGTTTCtcgctactcatga

>contig00471 length=241 numreads=1

cgcacatgaatcttcagcaggctccaagtaagctataaagctttcattgtgaaggacata

atgcaacaaagacttaacgttcttgaacgtcagcttatgcttagaggcaaacttctcaag

atcttcttctaatttttcaaattaattttgtcttcaattggcattgtgtcctccgaatca

ataggaaccaccacaggttcatcatcttcataatcactgtcactccccgactcagaacct

a

>contig00472 length=946 numreads=29

gtcaagcaattgtttatgtcatgtacTtGGTATGTATGGAGATCCATCTGATCTGGGTGC

tGGTGTCtGCTTGTtGATCaTAaTCcAAcTGTTCTGTGCTGGTTTGATTGTCTTGTTGCT

GGaTGAGCtACTCCAAAAaGGgTATGGATTAGGATCTGGTATTTCCTtGTTCATTGCTAC

AAACATAtGCGAGACTATTGTTTGGAAGGCcTTCAGCCCAGCAACGGTgAacACTGGACG

AGGTACcGAGTTTGAGGGCGCTGTCATTGCACTCTTCCATCTCTTGGCCACACGCCAAGA

TAAAGTTCGTGGTCTgcGGgAGGCATTCTACAGACAAAACCTGCCAAACTTGATGAaCTT

GCTAGCAACtGTTTTGGTGTTTGCCATtGTCATTTACTTTCAGGGTTTCCGTGTCGATCT

TCCAATCAAGAGTGCTCGCTACCGGGgACAGTACAGCTCGTATCCCATCAAGTTGTTTtA

CACTTCGAACATCCcTATCATTTTGCAAAGTGCACTTGTTTCCAATGTTTACTTCATCTC

CCAGATGTTGTCAGCAAAGTTTTCTGGCAACTTTTTTGTTCGCATTCTCGgAATTTGGAA

TGAGGCTGGCGGTCCTTCTAGGTCTTATCCTATAGGTGGGCTATGTTACTACCTTtCTCC

ACCTGAGTCGCTCTCGCAAaTCACAGAGgATCCCATTCATGCATTTATGTACATAGTATT

CATGCTGGGCTCATGTGCCTTCTTCTCAAAAACCTGGATTGATGTCTCGGGCTCCTCTGC

TAAGGACGTGGCTAAGCAGTTGAaGGAGCAGCAAATGGTAaTGCGTGGTCATCGTGAGAA

GTCTATGATCCACGAATTAAATAGGTACATTCCTACTGCTGCAGCATTTGGTGGCCTATG

TATTGGAGCTCTATCAGTGCTAGCCGATTtCATGGGGGCAaTCGGG

>contig00473 length=240 numreads=1

aagcagtggtatcaacgcagagacttttttttttttttttttttttttcaagaagtaaat

tttcttcgtccaagcgaacaacgagcaagtagaacggtaagaaagtgtaaatacaaacat

ctagaccgaccgggagtgaaacggggtggtaggatttccaataagttgtctacccattgc

acgggttcctctatgtagcttgtgtggatggtgtgactgttgtagccatccatgccaaac

>contig00474 length=221 numreads=2

ATGGCTTGCATACCCTACTTATTAACATCCTGCATGCCATACTTACACACGGcttgcata

ccatacttattcacatcctgcatgccatacttattcacatattgcatgctatacttactc

acgccatgcataccctacttattaacatcctgcataccatacttacacacagcttgcata

ccctacttattcacatcctgcataccatacttacacacggt

>contig00475 length=259 numreads=3

atgaggtgcttaacagctacgagttgatcttacgttagcacgcatacgagaactggcgag

agtggtcctgatttcgaaggttcttcaagagctgcttgcctacaaacatctacaaatcac

cacacagagcatttttcgttaggattcattctcgtaCCAGGACGGCAGTTGAATGttttG

CCAAAATcATGGCTGTTAGACAGCATGCCAACTAcccGTAAATTCCcGGCGAGTGAGCCT

CTGTATTCggggattggac

>contig00476 length=190 numreads=1

agctggaaattgtgatgggcgatgaacacatttcgtttacaacatcgaagataggctctc

tgattgatgtaaataactcgaaggatgccgagggacttaagtgtttctactacctcgtac

aagatctgaagtgcttagtattctcccttattgcccttcactttaaaaatcaaacccatc

taaacgctat

>contig00477 length=241 numreads=2

aagcagtggtatcaacgcagagtacgcggggtcgccacgtcggccattcatagtgcaagg

gttGCACAGGAGTTGAGGTTCCTATACTGTATTCATCGACATTGGCTGCATCTTCCAAGC

AAAGACCCAGAAGATGGAGTGTGGATGGAGATTAATAGCATTCTGTGCTCTTGGAGCTGT

TATTTCAGTACCCCTTTCTTATGGTGACGTGGTTTtAACCCATGTAGCAAGAAAtattga

t

>contig00478 length=1093 numreads=20

AAGCAGTGGTATCAACGCAGAGTACGCGGGGAaGTCTTAAaCTTTGCAGAAaTTTTGAAC

ATAAAAAGTTCCAGTATTATATCATAaGCTGCTACAGACTTTAGCCAGTCACAGTGGATA

CCTTACCAAGAGAAATGAATACCAATTACCCACCTGCTCTGCTAAaGCCAGTAAAACTTc

ATCTTCATCATAtATTGTATCTAAGGCCAAGCACAACTGAGTCAGATTACCTaTGCATaa

GAAACGcAtAAATTTGCTTACTAAATttGtAGgCAGTACAAGGTTAGTGAAGCTACCCTA

CACCATCACAAGATGATATATTTtAGtCATCAAgCGTACAGGTTCACTCTGAATGCAAGA

TCAAAaCTAAAGGACATCAaCTTTAACTAGCTTGACCGCCATCTtACCAATTATTGCATA

AAAACTTAAGTTTAAAACCAGAAAATACTATTATTGCAAGAATCACTGACCATGCAGCCA

GGAAGAATAAAaGATGTTTGCAAAAGGCTTAGCCAATGACGCTACCAAAGGATGGTTTtt

CCAGCAAACTGCTACCGAACTTGCAAACAATATCTTCTCTTTACAAGCTTGGCTCTCCCT

TaCTaTTTtGATTAGGTAAACTTaCCAGTAAGAAAAggAaTGAGTTCacTTCTGGTTCTT

TCtACACCCAGTGCCAAAGCGATCGTAGACAGCTTTTTaaAGAGTTGAGTCTCAACTAAA

GCCAGATGCAAAGAGAGTTATGTATGTGATGCTTccaacaagctgatataagtgatttga

caaactagcaacttctcatgaaaacaacccaatacaaaaacactacatGGGGCTTTGCAA

TATTTTACAAAGAGACAAGTGATTAATTCTATCTATGAACAGCTTAATCCTTAAAACTTA

TATCacaattgctgagatgtatacaaactacaacgcagacttggacggttgccgtctctt

tgataaaacaagcaacAACAACaaCAAAAAAGATTTGGGAGTTGACCTGAACGACTCCAC

ATACTTCCCAAAGCGAAGTATTAAAATATAATCGGTCTTCTCAAATATCCACTCTGCGTT

GATACCACTGCTT

>contig00479 length=242 numreads=4

gggTTTtCGGGGTGCAAATTGACCTTTAAAATTTCTGCGATtAGCTTTGCCAAATGAACG

CGTCAGCATCGCTACCAGGCCTGGAAGCACCTCGTTCCATGCCTTtACCTTGAAGGGCGT

TTTtGaGAAgCGACATGGCATGTTGTACTTGCTTCTGGTTTCCAGCAATtAAGAATTGCT

TCGTATCAGAGGAGGTAGGCTGGTTCTGCTTCAGCCTGACTACTGCACCTGAAAAGCGCT

GG

>contig00480 length=241 numreads=1

atttgtaactatttaaaagtttctctctgttagtgactgaacagttttatttctgtatta

tcactagctcttctccgtcttcatgaatgctgattcatttaaaacctgcttattgtttgc

aggtaaatgttacaagatgtggagtgcaaactctgcatgacgggtttctatggcttcact

atgtattttgggttagatcttatgaataaatcttttccaaggcttttactaagtatcttc

a

>contig00481 length=241 numreads=1

actgtgtaccatcgtcggcattagcatggcgtcgcctgctcaggtacttgccgatgtgtg

cacgtttcgtttactgagaaaccaagatcagtcgttcggaaaacaggtgctttggggagc

cgttgggtttggaacgtgctccttcctggtcgggagtttcgtgagcttctcgacgtacga

aaatccgtgcacaaaacaagtgataatcaactatcagccttgtttcttcgtcttcgcgtt

c

>contig00482 length=443 numreads=15

aGCCGAGAGCAGGGAGCACTTCTCGTCTTTGCAAtaGGATGGCAAaGcAAAAaCTaGTCT

cGGgATCATCAATTGTCAGCTTtcgaCTGCTTGGATCGAAaTACTGTGTGTCATTGGGCT

GTTGGTCGATCTCCAACATGACGCGGTCGGTtGCAGTCTCGCGATAAATATTGGACTGCA

ACCcTTTCTGATCCcAGCTGTAGgCCTGCACGCCCTGGTATAAGCTGGAACCAAGATACT

TCGCACCAGTCAACCAaGTAGGCTTTAGCACTCCACAaCCAtGAGCGGCGTCACAGCACT

tGCAGCACGAGTTCTTCTCcGGATAAACAAGCCATCTCATGCcGTTTATGACCAAGTGTG

TGCACGGCGTATCCTGGAACGCTCgCACACCATTAAACCCGCAGTaTCTGTCGTAGCGGC

CATTTtCACGGTCGACTCTCCCc

>contig00483 length=621 numreads=18

TAGAACTCTTTATCGAGGCGTTCATGTATACgAAGgAAGTTTCCGATtAGTATGCCATTC

TTGTAtGCTTGCAAAGTGGGAAGGGCATTAATTTTAAAATTCAAACTGACACCAGCTTCT

ACCGCCTTGAGTTTGCAAAACTTTACGTAGGGgTACTGCTTGGCTAGGCAAGCCAAGCAC

TTGTTCATCTGTACGCATGCTGCATTATtCTCGTCGTAAATaTGCACCAAAACcACAACT

TTAGTGTCTTCTTTGTCAATACTTTCCACGaaGTCCATCCCgcTTACTTCTATtAAGTCA

CCAAACTTtGgtCtgCTCTGTTTAAGAtAaGCTTCTGCTTCGGcTTTCATTTGCTGCAAG

CGCTTTTGGTGGTACGCTtGGAGGAAGTGATCATCCTCCTCGAGATCATCGTCCGACTCG

CGATCTGGGAaTTtATTCGGGTCAGCAGTGAAGGCCtGCTTGCtGTGAaTAGCGATCtGT

TCAAtAtCTTGcTCTCTTCGTTCGATACGCGCGaGTTGCTTgTACcGCTTgTAgTCTGAC

AAgaCtCCCTTTGGTCCAGTCTTCGGGCCAGGATAATAAGACTCTTcgtccagggcccag

gacattcctcgactgcagcag

>contig00484 length=346 numreads=2

aaggagtaccctaaaacccagatgaagtcgcctggaattacaacgatgcttcacggaaga

agcaaaactctttatatcaagaatatcccaagtttagagatagcgaCCCGACCcGAATCT

AAGCAAGCGCCTGCTCGACTTGGAGCTGGTCGACGGCCAAGAATTAATCGTTACAGATAG

TACTAGCCCTAAGCCAGTAGCCTGTCGGTTACACTATAAAATCGATTAGtGgAATTGGGG

CGTTCATGACTAAAGGCGATTGTGCTTTCGACattcgacgtttaggttcaattttctaga

agctatgtaaactcgtaatcagttctgttaaactcttgttcactaa

>contig00485 length=241 numreads=1

gttttatgaagtgcaaaagaagttgagctatttcaacgatttccgcctaataaaaacaga

gccaagtgagagcttgaatcaacagatagcttcaagacgactggttcttgcaacatcgac

agctttagaaaaaaggataccgtttatgtaaggcaaattacgtgataaggcaattgtttt

tcgtaaagaaagaaatgacttcatcaagcaaatataagaaaacaaggaaagcaatttcca

c

>contig00486 length=516 numreads=6

ggTGACGTCAACTAAGTTGAAAGCATCTGAAGTTTCCACGAGATGTGTGAAGATGCTCAG

AGCTGCGATTCCAGTTCCAACCGaaCCAGATACAGAGATGGCTGCTAGGAGCGTCAGTAA

GATGTTCTCTCCCTGCATTGACAACACACcGAtAGCGAtGATCGaTGGCAGGTaGaGACA

AaGGgcAATcaCACTCAgtaGTcGCAcgTTCTGACTGTAAATgctgtcaaatgggcttcc

ttgacctctgttatccgagcacacagtatctgtacctttaacaccaacgcatactttcca

cacaccataatgtgcgctgtagatgacagacaccccGTTtACCTTTtCTTCATACTCACA

GTCAATCAgATgCGTaGtAaGGAAAgCgATGAAAAgAAGAGCaaGGGAAATCAAAaGGAA

AgCcAgTAGCaGCAAAACCCATTTTTTGATGgAGCCTCgCTtAATCGgCtGCTTTTCCaT

TCccGTCGgCTATTtaGCTaCTCCAaTTCCACGgAT

>contig00487 length=219 numreads=2

aGTTGTAACGAACAAGTTGCAGCGTCCGCTTGAGGTAGTTTGACGATACTTGGTACgtGC

TTCCGTcTtGCTCGTAATGTGGATGTGCTGTCCAGGACCGCAACTCTCCTAAGACCTTTC

CGAAAttAAaCTCcTtCCAGGGTTTGCAAAGACTGCGGATCAACTTGCCACAACTTTGTT

GCCTCGGTGGTGGCGTAAAAGCGATCATGGAgcttcacg

>contig00488 length=242 numreads=1

tcgtccgttacaaccccgtggctccgtttgttgagatttggagcagtcgtcctcatgtaa

cttaccgcttttcactttgctcgggaagatattggctttaacgttcgtgagctccgataa

attccccaaactcgaaaaggctgcctagagacccgtaacatacatgctgctataggtggc

tacttcttgtgtgggctaaagatagctgatgtcaatctgcttttgtgaaaatgcgtctct

ac

>contig00489 length=224 numreads=1

caaagcagtcgtggctaatttagcttcggaatagcttaggtctcgaaatggtgagtcaat

gctatcagcttctgcatggttccggcttggctttgcctcgtcggcagagatgtcttggag

cgtctgtttagcctcgcgagaacgaatgttacgtttcaaaccctgtttatagcgatgcaa

tttagaaaaagttttcgagccacctgattgcgacttttttttcc

>contig00490 length=913 numreads=11

ctactccagaagcagcggttcctcaatctcaaccatcatctacctctacctcggaaactg

acagacctgtggccaaagactctaagcccgccaagcaaattttgatctacacgaccttct

ttggcgatgagtggaactcgggttacGgggAGAAGGAGTGGAGTTcGgAATGTCCTGCGT

GCTTAGTAAGCCGAGACAGGCGTGACTTTTCCGTATCcAaTGCGGTGgTGTTCCTGTCTG

GCAATGTTAAGGACAtACcGCCCCCTGAGGAGCTTTCCTTGTTGAGCAGGCCTCcAAGgC

CAGAAGTGGATAtttttGTGCATGGAAGccccAATTGCAAAAACGaTACACCCCGGATAc

TCTCATCtttttAACTGGTcAaGCACgTaCGATACTCGCTCGGATATTCCTGCTCCATAT

GGAAATTATAGCCTTCATcAaCCtGgTAACCCCAtCTTTCAtGTTGACTTtGACATAAaa

CAGCGaGACGCTATtCTGTGGAtGgTTaGTAATCCGAaaTcTctATACaGAAATAGTATG

TACTTGgCATtGCGTGAaTTtGTCCCAATCGATGTGTATGGTGAGTTTTCACCTTTGtCc

TtGCCcAGGggAAAaTtCAGTACGGAGTtCCGTCGAAGATACAAGTTCTATTTTGCACTT

GAAAACGCCGTGTGCaGTGACTACATAaCAGAGAAATATTGGGATGCCATAAATGATGGT

TTGATTCCGATCGTTCttGATTTCGGTAATTACAGGAAACTTGCCATTCCAAACTCCTAC

ATAGATCtCAACCcaGTTCAAGTCTTTtAAAAGAAGCTGgTCTGTTCATCACGAAGCTGT

CTTGGAATATGACAGCGtATGAAgAGTTtttCAaGTGGAGACACACTTATGATCCTTaTC

GAAAGATGGTTCg

>contig00491 length=242 numreads=2

GCACTGCGAAAAGAAGTTTTCAGGACGATTAAAGGTTTttAATGGCACACGTTCGTAGGA

AACCGAACGATGATCACTGTCAGCACACCTTGATGACGGATATAAATGCAAACTAGCTGT

CAGCAGGACAAAACCATCTCTTATGACACATGTCGCGTTTTCTCTTTtGCTTGCTTATAT

GTATAATATCTTTGCTGACACATTTGTTAGCATGTGTACTAaCTTGATGTagtaccgcta

aa

>contig00492 length=1024 numreads=14

ttcttcatctttttagcctgtcgctgctctttCGTGAGCTTTCGCTCTTCGTTTGCAGCC

TCGTGAGCTTtCtGCCGGgcAGCCATTTGCGCCCGAAcGTgtGCTTCcACTTTCGTtGgA

TCTTGCACCGcTTCGGTCGCCAgAACaCGCAtCAAGTTGGAGATTTTCACTTTCGgaGGa

GGtGgCGcTTCCAGGCCcAGACGAATTTTTTCCTGTTTttCTTTTtCCTGCTCTCGCCGC

CGCTGCGAACGAATCTTTTTtCGCTCCTTCTTCGTTAAGAATATCGGcGgCGAAGgAaGC

CGACTTGACGTCACTGGGTGCATCTTTCTGTATAgggtgctcaactaggtttgtaatgcc

cgttaaccgcacctccccgtcaaccacgggcttggccgcatcgctgtacgatcgactagg

acaaatgcAACATCCCACCACTCCATTTCGGGTACTCTCTTCGTTTCGAATTCTTCATCC

ttAACTTTTcgCcGCCAgCTTGGCtGCcGaTtGAATTCCCGTCTtCTTGGCAGTCTCTTG

AaTTtGCTTCTGcAaCTTCTcTAacTGTGCcTTGgCCCGAAtACGTtGCGCTAgcTGCTG

gAACTTGCcTGGTTCGTGAAACCTGAAGttCCGTTTTTGaCGAACGACAGgCAGATTCCC

cAAACGGGGATCAAAaTGAGAGAGGTCTTGAGGAGATTCGCGAGGTTtATTGCCcTCTAA

CTTTAGCTGCTCTCGTTTCTTTTCACGGATGTTCACCTTTAaCGTGGgCATGCGCTGAAT

CATTTGGATTTGCcGGCCCGTGGTTTGATCTATGGCCACACCATGCGAGTCAaCGACGAC

TGcGCCAGGCTTCACAGCCGTGATCGGAATGGTGTCATCGTCGGCAAGGCCAGTCTTAAT

AGGTAAGCTTCcGAGaGAGgCgAgCAGCCcAGCTTtCCCCtGCATcTTCTCGGCTATCTT

AGCCTTAAGGTCGGCAGCTCGTTTGGCTTTCGCAGCTGCcTtCGtGTAAAAaTCTCTGCT

CCTC

>contig00493 length=244 numreads=1

tctgaaaacgactgcgaatatgcttccttgtctgcttccaacgagatacctctagtcatg

tccaccgatgattcaccatttccgtccttacacaaccctgcagtattacactggcctgat

ccgtcagcagacatggaatcggcaagcaaggctgattgatccctgctagttttaccttga

tactcgtcccgactggttcttctagcgtgcacatccagttttgacgggtcgacgcacgcg

gtgg

>contig00494 length=773 numreads=43

tCTTTTggAAGAATCATCTTTTGCTACATTATTTCCTAAATATCGTGAAACCTATCTGAG

AGAAGTGTGGCCAcTGGTGAAGGAGCGCCtGAAGGAAGTGGGcAtaCGTtGCACGCTTGA

TGTCATAGAAGGCAGCATGACGGTGGCAACAaCAaGAAAAaCCTTTGACCCGTATATAGT

AGTCAAAGCTAgagATATGATCAAGTTATTGTCCCGAGGAGTGCCATTTGAGCAAGCTTG

CAGAGTGTtGGAGGATGAAACAACGTGCGATATAATCAAAATCAGAAATCtcGTTCGCAA

CCGAGAGAGGTTTGTCAAGCGAAGACAGAGGCTCATAGGTCCGAACGGTGCGACTTTGAA

GGCATTGGAGATTTTAACAGAGTGTTACATACTTGTtCAAGGCGGGACCGTGTCTGCGAT

AGGTGGCTTTAAAGGCCTCAAACAAGTGAGGAAAGTAGTTGAAGAAACAATGAACAACGT

TCACCCTATATATAACATAAAAATCATGATGATCAAAAGGGAGCTAGCCAAGGACCCGGT

GTTGAAAGACCAGAATTGGGATAGGTTTTTGCCGAATTTTAAGACGAAAAATGTTCAGAG

GAAAAAGCCTAAGATTAAAGAGAAGAAGCCATATACGCCTTTCCCTCCTCCACAGCAGGA

GAGCAAGGTTGACAAGCAGTTAGCCAGCGGCGAGTACTTCCTCAAGAAGGAAGAAAGAGC

TCTCAAGGAGACTCAaCAAAAAAaTTGAAGCAAGACGAGGCTGGCGAGAAGCG

>contig00495 length=356 numreads=4

TGTAGAAAGGTGCCTTCTTGaCCCcGCAGCCCTGATATCATTCGCGCaTTTATTCcGCtg

TCGAAGCGGaTCGgCGTtGTTGTACGCTGAAGGTtcACCGtGgTTtACGTGGCTtCGATT

CATTCcACGGCGAttACTTcGTGAGGTtACTAAATGCTTCGGaCGCTTCGGCCATCTTGC

ACCCcGGTCTTCTTTGCATCAGTTACAGATGGCTAGaGCGGGGCAAaTTTCATTGTATGA

AAGCCTGCAACTGAAAAGATCCCTCTGCGaaTGCAACAGCCtctgctaaaagatgatggg

cttggggaacagagctgcttgtttattttgttctttttaaagagatacttgtttat

>contig00496 length=393 numreads=4

cGATTCTAGACGCGTATTCTTTACCACGTTGTACTTTGGAGACCGCTGGTTGTCGCTTTG

AGGTGATGGTCCGTAGTTGCTGACAAAGCTGTCGCTCATTGACGTGTACCGCGTTGGCAA

GGGAGTTACTGGCGCTTGTGTTGGAGCAAAGCGAGGAAGTAGTCTATTGGAGTAACTGct

tCCTTTttCcggCCACGACTTGTTTAGTCGACTCTCGGTACTGCTTGGTTTTgttggcgc

cgttaggacagggatcccggtcacacttccgccacttctaccgtaggtggccattacgtc

ttccaaggaacgaaacttctcgttgaagttcgtctcgagttcgcgtagcttgtttgccag

atcctggttagtgacgctgcagctttcgtgctg

>contig00497 length=230 numreads=4

tGTCGgtaCAgCCAAaCTCCGgAaTTGGAGACCAGTGAACATGCcTGTTTTgAaTGTGGA

TCGGCCGAGAGCcTATGGATATGCTtAATATGCGGTCATATtGGATgCGGAaGgTaCCAA

GgCcAGCATGCATATCAGCATtAtCAGCAAACTGCCcAcACaTTTACAATgCAATTGGGT

AATCAACgggtgtgggacTATGCTGGTGACAATTATGTTCACCGgACTTA

>contig00498 length=230 numreads=1

cggcaaaggctgagcaaatcatttccagtatctgtacagaagactttatttgcatacatt

ccaactaaaatactactcatgccaaggccacagcctaattcaatgcaagttttgcctctg

aagtctttggcattagagacaatatagtcacccattagcaatgaacctttccatatttgc

atcccaacatcctgaagtgatgtagacattttatgcttcaatgttaaaac

>contig00499 length=222 numreads=4

AGTGAATCACGATCCTCATGCTTTAAGtAAGgCCTAATGATGTAAGATTTTGAAAGCAGA

ATAGTAGAATCCATGTCGCTTCCAGTTGAGAAAGGCaGCAATCGCTCTAaTTCTGCcGCC

ATGCCTCCACGAAAAACCATGGCTCAGgATCATCCaTTAAAAGCGCATTTTtCTTCGATC

TTTtGAGCCTGCCCATGTAACATATGaGCCAAGCAAATCAAC

>contig00500 length=230 numreads=4

AAGCAGTGGTATCAACGCAGAGtACCGGgAGAGCcAGCGAGGATCGAGCAGTaGCCATCG

ATGAGGtCACCAAGCTCCGTAgCCTTGTCGATCGAGTTGCTGGCAAACACAAGTGgCTCc

TGGTTTCCATCTACTTGCaGGgAGAGGgtGGCCTTGCCAgAGACGGTCgAAcTCCgCGCG

ACAtAGaTGGATcGCACCTgaGCGAAGTCTGCCATGTGATTGATaactga

>contig00501 length=795 numreads=10

cAGCTAACTGCACTTGCAGTTTCgTTtCCTTTGTTTTGGCACGTTCTTTGAAAATCTCTA

GAACAACGCGATATCTATCAAACACTTTACATCCAAAAcGagACTCTAACTCTTTGATCT

GTGTTAAACGGAGGCAACCAGTATTAAGGAAAACCATTGTAACGCCATTCACGTCAATCA

tGGTTtGAATATGAaCAGACAAGTCTTCCATTTtATTGGgTCcGAAAAACGTTTTGCTAT

GATGATCATGAaCtgACTCTATTTGaCCACCAGCAaCAGtCCAGTTTTGTAcACTGCTAA

TCAGCCCTTTAGCTTCGtCTAgtCGGTGTTCCGCAAGTCTGGTAAGAaaCCtGtcTTTCC

CCcATTTGAAAttcGGCTGCAAAATAAgCACACAttcTTtCTCAgcAaCAGATCTTTTAA

GCTCTGCAAATACATCAGTTGTGTCGTCATCAAGGTCAGCAGaaCGCTCCGGTTTAAACA

AATATGGAAGCTCAAACTGCAGCTTtCCATGCCCACGCTTTCCGTGCTTAGTAGCCTTGA

AGATATAGGGCAACTCAAAATgCTTTTTGGGAGAGCTActttcaaccaaagtcttttctt

ttgcTTTTTTCGGTACGAAGCAAGTaCCAACGgTtAAAaaGTTTTGTCTACGTGGCTTAA

AaCATATTCTGCATGACaCAAGAATTtCACGTATTGgCGTGCATGACATAGAGGTtCGTG

CACtGAaTTGCATCATCGATAGCATTCTTTTCAGCTCCAAAAATCATACGGCCACATAAA

ATCTTCACAAAATCt

>contig00502 length=240 numreads=3

aTTTAACCGTTTGCTGGTGCTCCAGGAGATGACGGCAAGCCTGCTCACTGTGTTTGAAAT

GGCCGACTATTTTTCGaaCTTTCTTGACGACaatttctacagacgtctgtgttaacagag

catcatgctggaCCAACtGCAAAGtGTGAGCCATGCAGCCGATGTCGGTGACGCCAGCGA

GTTTCATGGCTAATTTCATATTTGCGGCATTGTCCCGTATTCCAACGTGAATTTTATCCG

>contig00503 length=172 numreads=1

cgcagacctgtgacaaatactgatcactttcaaatttattcgaatccttcacaggcatca

accaagtcaacatatataaagtttatatagtctatgtagcactaggaataaaaccgtcac

ccacaatctttcaatcgcagggaaaatattacgcaacttcattcaaaaagga

>contig00504 length=1004 numreads=39

cTCGCCATGAaGGaCGACGGCAATCATCATTTCCAGAAGAAGTTGTACAAGTCTGCCATt

CTCGCCTACACCGAGGCGTTGAAATGTAAaCACGACGACAGCACACTCAAGGCTGTCTTG

TATACCAACCGTGCAGCCGCGCATTTCCATCTAGGAAACAATAGAAGCGCCCTGACTGAT

GCGGCTTTGGCGGTCAAGGCAAAGCCAGACCATATGAAAGCTCTTGTTAGAGCCGCGTTG

TGCTGCTTTGATATGGAAAAGTATGAGGACTGTATTGTTTGGTGTGACCAGGGACTGGAG

ATAGATGCAAAGGAGAAGAAGCtCCTCGAACTCAaGAAGAAaTCtCTtCACCAAAAaAaG

CAACATCAGCGCGATGAACGCAAGCGCGAAATGAGGAATCTGAAAAACTTGAAAGAACGC

GAAGAGCTTGTCAATGCCATTAAGAGTCGCAACATAACGTTGGCGGTGCAAGATCGTCTG

GAGGACGGAACTTCGAGTTCAGACCCCcTCGTTAACCTTTCCAACAGGGATGCGAAGGTT

TTCTtGGATAAGGACAAAATTCTTCACTGGCCTGTCTATTTTCTTTATCCTGAATACAAC

CAGTCGGATTTTGTTGAGAATTTCGAAGAGACTGCAAGGATTATTGATCATTTGTCTCAC

ATGTTTGCCGCCGAAAACTTGCCCGACTGGGATTCCGGGAAGAGCTATTTtCAGGGAACT

TACAGGTGCTCTtCGAGAaCCGTGACCAAGGCGTGCTTGtGCTCGTGCCGCAAgaGTTGA

CACTACGggAGGTGCTGTCTGATTCCAGATACGTcGTCCGcGGTGGGTgCCCTGCTTTTA

TCGTCGTTTCGCACGTATCGCCGTTCTACACCGAACTGACCACAAAGACGCCATTACAGC

CTTTCAACTAATGGGCTCCGTTtACCTtCATGGCGATTGAAAGATGTTCGtagaaaaaaa

aaaaaaaaaaaaaaaaaaGTAaCTCTGCGTTGATACCACTGCTT

>contig00505 length=242 numreads=2

atttctgggcgaggcttcttccgagactttgaggggctcaatttctccccttgctgccga

tcatcagtgccttcctctggcttcagatcaagatgatccagagcctgaCGGCAAGTGATT

ATGCTAGCATCTTCTTCTTCGACGGAAGCCCTACgGTTTCCAGCTCACCCTGAGAGACCT

TCGCAATtAGGTTTTTTTggCTCCTTCTTCAGCGGTCGCCGCATCAGCTTGCTTGAAGac

ct

>contig00506 length=241 numreads=2

TCATGTCCCACCGGACGTAGAAGCAGcAAATTTTtCTTtCTTAGATCCGCCTGGGTCAGC

AATTAGCGTCAACCTTCTCGCGTGGAATCGGATTTCAAGCAGAATAACGCTCGTCACCAT

GTTGGAAACTAACCTCAGTGCTCTTAAAGGTTtCCCTTCGAATAACTGATGCATAccttt

gttttactaccagatttgatcgaatttaagcctgtgtgtaacgtaaaagttgtaaaattt

g

>contig00507 length=238 numreads=2

agcacccgaggaagatcagcgcagtccgaaacggattcagctttcagacttctactgctc

acaaaacttgtcagggtggaaatctctaggtcgtccgcacgacgcttctcctcaatgtcc

gacttttcacaaccgctatccgacgtcgcgtcgtcgtcgtttacatgattcaccgcgcga

attgccggcgacatcTTGACTCGCGTGGTCGGGCTCGGAggACAATTCTTGCCCGAAT

>contig00508 length=614 numreads=10

gcaaatgagaatggccccttgatagactcgaaaGGACGAGAGACTATTTtCGtgCATGAG

TATAATTCCGtATGGGGGAagTTTCTAAGCACAGATCGCCGAAAGTCcTCGCTCTAgaGT

GCTGGTTTtCAAGAGTCGCAAAAAaatAAGgctGGcGTTTtCtCttGCGGGCATTTtGTT

CTGTTGTTTATCTTCTAAAGCAGAGAATTtATGgTtAACCGTTGTATCCggtgtttaaat

gaccaTCTGATACTTAGCGCTTCTGGTtATTTGCGGCTtCTAGCTGCTTGaGCTCCTTGG

ATTTGGATTAGTTGCTTCCTTGTATaTCTtCTCGCTagTCTTTTtCTACAGTAGGGATCG

CTTAAaGTGgCATGCGTTTATGCTTCAGTTTAGATTTATTTCAGCTTGTCCATGACGCTG

GTTGCGACTCTCTtAAAGTGGCTGGTAATCAGGATGCGGCTCTTGTTATTTATGAGCGGA

ACaCAaCaGAACCGTCCAGACCAGATTTTTtCCCGATAAGTGGAGCGCAtCTAAAAAAGA

ATCTCGAAAAGCAATATGCATTGGATCTCATAAATTatagtaactgtcgagatgtattat

gtagtatctttcat

>contig00509 length=1146 numreads=62

cTATGCTCGACGGTTGCCTCCTtACAGAGGAGGAATGTAGCAGTCAGTTCcTCGCACCGT

ACGATGCTGTCGGAAAGCCGAAAGCGACCGCATGCCTGACGCGAGCCAGAGAACTCAACC

CAATGGTCGATATCGTGGCTGACACTGGGGACGTATCCAGCAAGGGAGAGGGATACTTCG

CAAACTTTGATGTtgTgTGCGTGACAGGTTGCAGCGCGGAGCTTCAGTTGAGAGTGAATG

ACATCTGCCACCGTCTGGGATTGAAGTTTCTCTCGGGgAATGTGTTtGGCTACTACGGTT

ACATGTTCGCTGATCTTGGGACGCACGAGTATGTCGAaGAAAAGGCTAAAATAGCCGTAC

ACAAGACTACGGAGCCGCCCTGCAAGAAAGCAAAAATGGATGAAGGCAAGGATCAGGAAA

TGGAGCAGAAGGTGTCGGAGTTCTGCAGTCTGAGAGAGGCCCTCGATCGCAACaTTATGG

AAGGCATGACTgcATCGAAAATCAAGCAGACCAGTAAGACGTACCTGGTTATGCATGTTC

TCCACGCTTATTGTCAAAGGTACGGTGAGTATCCGAAGGAAATCTCCAGCAACGAACAGT

TGAGACAACTGCTAGACGTGAGAGGCGATGTGTTGGAGCGCCTTCGACTCGACGACGACA

TGTTGCCGGATGAGTTTTCCAGCCtGTGCATCGGGGAAGTCTATCCAGTCAACGCTATTG

TCGGAGGGGTGTTTGCTCAGGAGGTCATCAAGGCCGTGTCTGGTAaGGATCCGCCTCACA

ACAATTTCTTCTTCTACGATGGCGACGCAACGAGCGGTGTCGTTCTGCGAGTTGCACCGA

GTATAGCTGCTGgCGCcGATCGTTCCGAAGCTGAAACAaTtGCTtAACCTGTACCATGAG

AAGTCCTCGCTCGCTTGGTGTGTAGCGCTGGTGCTACcTGGAACGTGAATtGGCACATGT

TTGAgTGCGAACAtCGCTTTGCTgCTgggtccattcgtcaccgtcccaggcaattgtcga

gtcataagacgttttcgctactggtctttaggagactgTTGCGATGCACATACTTGaaGT

TTGTTAGCTTtCCCTTGCGTGACGTACAGGTTTGCATGATATGATGGCGATGTGCTTTCT

GTGGtt

>contig00510 length=292 numreads=3

aaaggtgatgccgcagaacgaacatggatgaggccgctcccccaaatgtgaccgcatgtg

gatcttgagaTTATACGcgCgTTtAAATGTCTTCGAGCAAAACTTGCAAGTCACTGGCAG

AATGCCGAAAATCCGAGGCAACaTTtCTTCTCTCTCTTGCCACATCGccGGCAGAcactc

ctccgtgagcaggctcaccgctccgggAGCGTCGAACGAATGAGCAGCGTAAGTGTGCTC

CGAATCGTCGAAAGCAATACATGGAttAGCGTCACCAGGCCTGCCctCCggg

>contig00511 length=258 numreads=6

ACAAATATTGGAGACAATAGTACTGATGCAGAATGCTGCGTTTGCAATGAAGCTTTACCA

TACACGAGTAAGTAAATCTGcAAAAAAaGgTTTAAATtATAACTATCTGACGATAGAGTt

ATTTAATGATACCCAGCTTGGAACAGTGCAATGCAAAaTTAATTAtCAGAGAGAAGGAAC

AATCtAGTAAAAgCAATCGAATtCAATTAAATAAATCAACTaatttcacaagaaacaatc

cataattttcggcgaaga

>contig00512 length=1929 numreads=85

AAGCAGTGGTATCAACGCAGAGTACGCGGGGATGGACACAAAGATCATTCATTCAAACTT

AAAAAATCTGAGCTACGAAAAAATCAAAGAATCCCTTACCCAATTCAAGAAATCGAGCGA

AGATTTCGTCCTACTCTCAATATACATCCTGACTCCAACGCATGCGCGTTTtCAAGTaTA

TtCTAGCAATTTGaTCAAATTGACTCATAAaCAGaTTAAAATCCTCGTTCCTcGAATTCA

CCAAAAgTTCGAAAAAaCGCtAAGAtcTTaCCAAATTCAAcTACCCATAGGgAacTTGAC

CTTCGAGCTAaTAAACGTACCAAAGAAaTACCATGCTACGCAAAGAAAGAATAATGACGC

TAgACATTGAAACTCACATAATTAAAGGAATCCATTTACCcTTCGCGGCATCCATATGTG

AAATCGAAGGTGTAAaCAAAAAAACTCTAACTCAAACTTGTCATCGATCCTCCCATCTTT

TACTCGAATCTCTTTTtCTTAGATGTTTCTTtCATTTCAGAGGCTCACACTTCGTTTTTT

ATTGTCATAATCTAGGTAGATTtGAAGGACCACTTTtGGCCAATTTCTTCATCGTCCATG

AAGAATGTATGATAGAGAACATCTTAATCCATGAAAaTAAAATCATCATGTTACATGTGA

AACTAAGAGGAAAACATTTAGTGTTTAAAGACAGTCTAAGCTTCCTACCCTTCAAACTAT

ACGAACTCAACGCCATGATCGCGCCAAAAAAGATCAAGAGTTCTTTACCCTTTAACCCAT

TCGTGAACAAATCCcAAAAACACCGAGTTCTCCCcTACGTAAAaCACGACGCACAAGTCT

TAGCCTTCCTCTTATTCATTTTTAAAAAACAACTTCGTCACAATTTTCTTCAAGACCCCT

TGATTTCGTTTGGAACCCCAGGGATGGCCTTAAAGATTTACTCTAAATTTTTCCACGAAA

AGAACATTTTCACTAAAGAACAAGAAACCATAAGAAAGAGTTTCAAAGGGGGATTAAACT

TTATCAAaCAACAATCTCGAGTGAAAGGGGTTCTAGGTTTCGACATCAACTCCcTATACC

CTTtCTGCATGCTAAATAAACTACCTGTAGGTGAACCTAAGTTCAAAAAAAaTGTCATCT

TAGAGGATTTCTTCGGGTTCGTATACGTAAAATCATTAAAGAAACTGAGCAGGGGGgCAT

CCGTCATTGGAAATAATTCTAACCACTCTCTCCCCCCcTCTGCTCCATACGAAGAAAAAC

TCCTCTCCTCGACCCCcATGATCTTATTTAGCGAAGAAGCTAAATACGCCCAAAAACTTG

GATATCTGATAGATATAAGATGAGGAATTGAATACGAATCTTCTTTTTCCGTCTTTCACA

AATTTATAAACTATTTCTATAACTTAAAGAAATCTCACCACTCCACAAATTCCATTTCAG

CCAAGCTGATCATGAATTCTCTCTACGGAAGATTGGGAATGAGAAAAAACTACCCTTCTT

ATCAAATCGTTGATCGATTCGACTTACACGAACTAAGCTCCCAAGGAAAAAaCGTTAAAC

TGATTAGATCTTTCACTCCAAATGCCTATCTGATAGAAAACAAGGAAGAATCGAAATACA

ATCGAACCCTAGCGTCAACCCCGATAGCTTCCGCCATAACCAGCTACGGAAGGATAACGA

TTCATCAATGAATCACGAGAAAAGAATTAGAAATCCACTATTCCGATACGGATTCGCTAT

ACACTCTcTCCCCCCcTCcGTAAACTTCATTAACGACAACTTGGGgagCTTTAAAAaaaT

CAACCCTtCTCCCTgaGAtgaagCAATaTTCAtCAAATCTAaGATGtATGGCTtAAAGAG

aGGAAaCTTTTCCATAAACAAGATCAAGAGCATTAGATCTCCTAAATGAGAACATCTGAC

GGAATtaaa

>contig00513 length=241 numreads=2

atgtagtaagtgtgCTtGGCGTCAgCGGAGCGAGATCGTCGTGGCTacAAGAAAGGTAGG

TTAAGGGTCGACTGTTCAATACAGCTTCCGTTTCAACTAGGACGGTCTGTAGTTCGTCGT

AGGTGATAGTCGAcctGCCAAGAATTTTTCTTAACACCGATTTTGTCATTCCAATTAGTC

TTTCGTAAAAAccCCGAACCAGGGTGCACGCTTTGTTATGAAGGACCACTTTATCCGATG

A

>contig00514 length=447 numreads=6

AAGCAGTGGTATCAACGCAGAGTtggcACGGAATTGCTGCGGCGTGAAAATCGAAGCGCT

TtGCCTTTAAGCGTGAAATTTTTTGTTAGTTGGGGgCCGTTTTTTTGTTTAGTCAGTTTG

GgTTTtGTCATTTTGGTTTTGTGTgaggatgactgctttcagattttaggatgcgacgct

catgctgttcctgaTGGAtACTTGAAACGCAAACTGAGAAGATTTtATGACATATTTGCC

TTCTACCACGGTtCTATCGATCgCCTGtgcAAGgTAGGTTtGTCTCcTACGGCAATGTAg

TTTCCTAAAGCATGGTAAGATGCGCGCACGGTTGCAGCTTGACCAAGGTTGGTAAATGCG

AAGGTTGCGGAAGGTTGCCCATGGCGCTTTTtACTtGTGGAGTGTGATTGATGTGGACCC

GCGTACTCTGCGTTGATACCACTGCTT

>contig00515 length=720 numreads=17

cgctggctcgacagaatgctgattcggctcgtgcaaaaattCGGCGAGTACAACAAGGAC

GATCCGAACTCGTTCCGCTTAGCCGATAACTTCACGCTATACCCGCAGTTCATGTTCCAc

ttgaGACgATCGCAGTtCTtGcAGTACTtCAATAaCTCGCCTGACGAAACGTCGTTTtAT

AGGAATTGTTtGgATAAAGAGGACACAACAAaCTCGCTTATTATGATTCAGCCAaTACTC

TATTCTTATTCCTTTCACGgACcGCCAGAGCCAGTGCTTTtGGACTCCAGTAGCATtCTG

CCAGATAGGATTCTGTTGCTGGACACGTACTTtCTGATTCTCATATACCATGGCGAGACT

GTAGCGCAGTGGAAAAAGGCCGGCTACCAAGACGATCCTGCGCATGAAAaTTTCCGCCAG

CTTCTGCAAGCTCCTTtGGATGATGCTCAGGAAaTTTtGCAGACCAGATTCCCAGTTCCG

CGCTACGTTGAGACGGAACATATGGGTAGCCAGGCTCGTTtCTtGCTGTCTcGTGTGAAT

CCGAGCCAGaCTCACAACAACATGTACTGGGGTGGCTCTGATGCTGGATCTGCCATTTTG

ACTGACGATGTTAGCTTGCAAGTTTtcACGGATCACTTGAAGAAGCTCTCTGTGTCTTCG

GCAACGTGgAGGGgTCTATGCTACATTCATCTCTTGCCTAAcgcggatgacctttgtcga

>contig00516 length=243 numreads=2

AAGCAGTGGTATCAACGCAGAGTCGCGGGAATAATAATAATAATTTATTTTtAAACatca

agtgtatttctaaatcacatagcatcgtacaaggaatacagctagttatctgaatctggc

actcgctttacaagtatgccaggtccgcgttgctcgagctaccaatgtaggagttttggc

tgagtccaaggttggtcactcctgcaaaagatgtagtaggtcggatcgcatggtaatgtt

tat

>contig00517 length=251 numreads=3

aGgaGATACTCATAaCcTCcAaCACGtgTTctaCGtGGAAGAAATCTAcACATaCGAGtG

cAagCGGtgACGATGTGAcAaTTTtACCCcTAGGGGCATTGATGACATGTAGTATCCTCT

GTTTTAAGCAGGGACACACCTTTtGTTACATGATGCTCTCTTTCATTTTGAATGTTTTGC

CAGAATACCTGGTCATGTACTAGCTGGTATACCCTCGCGTGGCATAAGTAccccatattt

tcatgtagctt

>contig00518 length=870 numreads=24

ccccaaggacgataccagcgaaacctctcagctgAGAACGAAaCAGCTTTTTGTATCGAa

CGTGACTCCTGGTCTCACAGAGGAAAAGATACGGGATTATcTtGAAAGCCGCcATCCTGC

TAGATTtGGACAAaTTGAGAAGATAATTTtAATGAAGACGAGAGACCAATCGACCGGCAA

GCTTACGGAGAAAAATCGTGGTTTtGGTTTCATAACCCTCGATTCCGAaGgATTTGGCGG

ACCGAATAGCAATCGGAGATGCTGTGTTtAACATCGGTGGTATGAGCAGACCTCAAGGGA

TACAGAAAGCCCTTCCTAGGGACGATCCGCACGCGGCTCCGCTGCCGATATCGCGAGGGC

GAGGTGGAaGAaCAATGCGCAGCGGTGGTGgTGGTGGCGGTAGCTCGCGTGGTCGAGATC

ACGGACACGACTGGGACgATTACGGATATGATTCCTATGCGGGAGgCTACGATGATAGGT

ACGGTACCTCCAGCGGCCGATATCAGGATGAGTATGACAGCTACTaTAGCGAtAGCCGTG

GtGgCGGtGATTatGgAaGAAaTAGCGGGTACTCcTCAAGGTACGGCGAGCCACAACCGA

GTCGTGGAAGAGGTGGCGGTGGCTCTTCTtATCgAGGGggTGAAgCgTCACGAGTtGGTG

GTGGTAGCCGTGgAcGCAGTGATCGGTTTAGTCGTGACGCtAGCCCcGATtACCGAGCTG

GTcAGCGCTATTCACCGTACTAAGgATACAAGTTGGGCGATCCTGATCGAATTCGTGGTG

TAGTGCTCTGTAGCTTTTGAAGCATCTGCTTGgAGGTTCGTCGTGGTGTCCCTAACGGCA

TAGTCAGCTTTATTTCCACGCCAAtcatcg

>contig00519 length=352 numreads=4

tAAGAAAGTTGACAGTAGTGATGATGATAGTGAGGAAGTTGAAGGACGGATAAGGaatcg

tcacacggcgcaagcgacgAAGCCCCCTCCACCTCTGAAGGAAAAGCCGAAGAAAGGAAA

CGTTGGAGAGACAGTGTCTGTCGTTTCACTTTCtGTCCCTGAAGTAGTgCCACCGGTgAA

ACCGGCTCATGTTGCTCAAGCTGCCGTTGCTAAGACTACTGCCGCTGCTTCTGCTGAAAA

aGGAGGAAACAGAAGAAGAAAGGAAGGCCCGAAGGCGCAAAGAGCGCGAGGAGAGGTGGA

GAAAaGGAAGAGGAGGCCGAGAAATCTGCGGTGGCCAAGAAAGATTCTGCCg

>contig00520 length=189 numreads=3

ttAAACCGTCTAACCATAAGttGAAACAACTTTtCGGCAgCCTtCCTCCTTGTTCGATCG

TATGTAAATGTccACCgtcttgagaaacatttttttggggtcattaactttgagcgcctc

ttgaaaagcagcctccagggtttcttgggATCCATACAAaTTTTCAAGGTTTAACAAGGC

GACCCAAAc

>contig00521 length=241 numreads=1

aagcagtggtatcaacgcagagtacgcggggctctctgcgcggccatcttgttaaggaaa

ggagggtctaagagcagttgtggcaaaatccagccgcgatctactaggtgaagggctgtt

tttgtttcttctggtttagaggtctgttgggaaaatggacgcatctggtggtggcaaact

tgctcagctgaaggccaagttagctagtataaatactcaaattgatgaagctgatgctcg

c

>contig00522 length=241 numreads=3

ACTTGCCTTTACCAGCCAGATAGTGagAACCacTTTGGCcACcAATtAGCTGCTCTTCaG

GgTtGCTAATTAACgAGTCcTcGtCGTCTtCGTTTACTACATCGAttGTGAACTCAGCTT

CGACACCTTGTGATGTATTGCCAAGGTCTCCCTTACCGTTGCCGTAAGATTTGCCACCTG

TTCGAGCAAGGCTGTCTTTTTCAGCAGGAACTCTTTCAAGAATAGGTGGTTCATTGCTAC

T

>contig00523 length=333 numreads=4

ggctagctcccggagcaggaagatttttcgcaggaggaggaagaggagcatcagatccag

tttgcttacatttttccgaaGGAAGTGAAAGACTTTTCGACAcAGCCAGCtCATATttCG

CaTGGTCGACATGCGAAGGGTCGTATCTATCAAAGGCtGCTTTCGTTATGGAACTATTGA

CGTCATTTCGTTTTccGATTGTCTTCCCAAGAACTTTCTCAaGGATAGCCAGGGAGTTCT

CCTTTtCCTTCTGCAAATCGAGATCAAtaTTCTTGATGaCATCCATCTGACTGgTtCcTg

TAGGgATGgAGAAGGTcATgcGGGGCgAGTCtt

>contig00524 length=241 numreads=1

aagcagtggtatcaacgcagagtacgcgggaaggtgaaacaggctgcgaaaatgattcag

aggaagtcgtcaaatgttgaaaagcagctcaaatttctgcgcactgccttctcgtacacg

aacgattttgtaaatgcgtttttagaagtagacggggctttgtacgccttgatcggttac

ctgacgggtagtgtttcggacctgcaattagaggcggcgtggtgtgtcacaaacattgct

g

>contig00525 length=492 numreads=5

ggTGTTTGACAAAGAGGAAAGTGGATTCATAAACTCCAGCGAGTTGCGTCGCGTCATGAC

GAACCTGGGAGACAAGTTGAGCAATTTGGAAGTAGACGAAATGATTCGGTGTTCGGACAT

TGATGGTGACGGACAGGTTAATTACGAAGAGTTTACCAAAATcATGATGAAAaTGAAGtG

ACTCTCAGCAGCTGTTTGCCCCcTgCTGGGCGAGCCAAcTTTTTCgaGGgAAgaTTTGaa

actgtgcactaggcagtttcgaccatgttttatttatttatttgagctggtgagagaatg

aaaacaagtatgTTGGGAATAaTCTTAAACGTGTGGACTATGCAAACGCcttaGAGGATC

AGGGTCTATggacttgacgaaagcgtttgattataatatgaagaaaaacaaccagatgtc

gacttcgctacttgcatagcaagactatgagaacttaggcacttgtaaatagctttgtca

ataaaaccaaaa

>contig00526 length=239 numreads=1

gacggttgtgtggattagtcataagttaaacaagaggtcgctgaacttattgcggctgac

taaacatcctcgcacctcggtaacttggaaaactttatttgaaagacctacaggcctgct

ttgtttctaacatcggagatgcgagcagcgggttacgtcattggcaagtcaggctagaca

cgttgaggagacgtatcatcaaaatttttcacttccaaagcatgtagatccactcaagg

>contig00527 length=491 numreads=4

aagcagtggtatcaacgcagagtacgcggggaagtacagaagcgctaccgtaccatgctg

tcgttaattcggtccttgtcgtggaaaagcaatggacaggttgcaccatgcagttcccga

ttcttccatttttccatgcaaggtaccagcagtaaatcctccaaggctgagatttcACGA

GAATATCCGGTAATCGACCACACCTATGACTGCGTTGTAGTTGGTGccggtGGtgCTGGC

TTACGAGCTGctttcggcTTGGCAAaTGCTGGATTTCGTACGGCATGCGTgTCAAaaCtG

TTTCCCACCCGTTcGCACACAGTTGCTGCGCAGGgTGgTATCAATGcTGCTCTTGGCAAT

ATGGAAAGTGaCAaCTGGCGCtGgCACATGTATGATACAGTAAAAGGTTCAGATTGGCTC

GGAGACCAAGATGCAATCCATTACATGACCAGAGAAGCTCCGCAAGCCATTTACGAATTG

GAGAACTATGG

>contig00528 length=240 numreads=2

TCATCTAACTTCAATCAAGTAGCCTGAACAGCTACATCTATATTTGAACATAAGCTGCAA

GGATATCCATTCACCAAGTAAATAACTGCACCGGTGTCTACTCCAGAGAAGACATCACAT

TCTTGCATACAAAGTGCATATGCCGTATCCAAAAACCTTGAAAGAACTTTCCTCGGTACC

AGACATTAATGTATGAACTACAAAAACAACAATCCATTCACACTGTCACAGAttcctgat

>contig00529 length=241 numreads=1

tgtcgctttaattctttagcaatcttgtcgtcgcactaaattgtggcgacggctgaccgc

atcgcgagttagggcacagctagacggcacatggaggtccgtttctacaaggcttagcgt

taaggtctacttctcctcgcgttgttcgcacgttggttggcagggtgcctgacaggatct

ttatcgcagtgacaagctgactactgccgaatcgaccgtccaaagccatggtctttggac

t

>contig00530 length=241 numreads=3

AGAGgaGCTCCGACAGAAGGTtCGCcAAagTTTTCCctCTtgAaCTTGAAAgCcGACATT

ttGGACGATCAAaGTCGATgAAgCTACCCCGTCGCCATTTGCTTtcGAGCgaGGACGACA

ATtGCCCGtcTGATtCGGAaGTGTCGCCcGAcAAaCGaCgACCTGTGAaGAaGCATCACA

GCGACACTGAAGCCATGCGAAAgCaGCGTCATTGGAAGTtGAGtGgTATGGTAAgcgccc

c

>contig00531 length=849 numreads=25

tCAGTCACGCAAGCCTTAAAAGATATTTATGTGCTGAAAaaaCCTAaTGCcTGTATGTTt

CaGAgAAAAAATAtCCTGCGACCATTTGAAGATCAAACGTCTCTTGAGTTCTTTtCTCAA

AAAAaTGATGCGTCCcTTATGGTATTTGGTTCCCATTCGAAAAaGCGACCCAGCAACATC

GTGTTTGGATGTTTTTATAATGGTCATATTCTGGATTTGATCGAGCTTGGAATGGATAAG

TATGTGTCAATGAATGAATTCAAGGTTGACAAAATCAGCTTTGGAACTAAGCCTTGTCTG

TTTTTCTCTGGTGAAGAGTTTGAAAGCAAGCTTGAATACCAAAGACTGAaGTCTTTCTTG

ATAGACTTCTGGCGTGGCGAGAAAGCTTCAAaCGTTCGGCTGCAAGGTTTGGAACACGTC

ATACAAGTAACTGCGACTGACGGGAAGATATTTTTCAGAAGTTACCGCGTGCTTCTGAAA

AAGTCTGGCTtGAAAACACcTCGAGTTGAAGTGGAaGAAaTGGgCCCTTCTTTCGAcTTT

ACCCTCAGGCGAACcAGGcTtgCCTCtGaCGatCTCTTCAAGgAGGCcTtGcgTCAaCCT

AAGaCGcTCAAGgCAaGgAAAATAAaaaaTGTagCtcaCgaTGtATTCGgCaCgAAAACG

GgTCgCATCcaCATGCAAAGGCAAGACCTGGAtAAGTtGCAGACcAGAAAAaCGAAAGCT

CTCAAGCGGTCTGCTCCCACCAGCAGTGCTAAGCTGAATCGTCCGGCAAAGCAGGgAAAG

gCTGACAGCGAGTAAGACTGCcTACAGGCATCGCTGCAGAGTACGTCTTTGTGCTTTTCG

AAGCCGGTA

>contig00532 length=241 numreads=6

GAAGGtCCAGTTAGAGCAGCTGGGTGTTGAAAAtATGATAGCAAAGACCGCGCTTTCTGA

GTCCCAGCTTAAGCAGTTTATCAAAGACCCGCCAATAGGTATTGACGCTATTCTCTGGGA

GCAGGCAAAAAAAGaCAACCCAGACCCTAGAAGCCTCATTCCGGTGCCCATGATGGgATT

tGATGAACTCCTTACTCGTATCAaGCACCAGCAgTTtCAGAcACAGCaGCATCAACGCcg

A

>contig00533 length=977 numreads=24

cACTGGATggTtGCTTACAATTGCTCGAGAGGCATACTGGCGGAGCCgCcTTgAcGGCcT

CAGATAAAGTTActCTCAAGCTtATTACGCAAAGTCTGAACGaGAaCAAGATTCCGTCAC

TCAGTTtGTCTGAGgAGCAAAAGCGgCAAGTGAGCGAGGAAGTCAGCGcTAGAAAGGAAA

GCGTCAACATGAAGTCTGTCcGACTGTGTTtCCaGGCAATCCTGTtCGATCCAAACAaGC

AaGTAaCGAAaGTCTTGCCAAAGGTTtACTCTGACTCCATAATCGATGCAAaGACCCCTG

ATGGTGCAGTTCTtAAGATAtCGAAGTTGAGCAGGgTTAGCAGCAAaGCTGAAGGTGGAC

AaGAagTAATTATTCTtAGCGATAaGCTTTCAAAaGAcGATAtCCGTGTGCGATTTTtCc

AAGaGGAAAaTGGAAAAATTATTTGGGAAGACTTTGGTGtATTTGCGAAAaaTGACGTAT

ATAAGCAAGTGGcGATAATATTGCGAACACCTCCGTATAAaGACAAGTACTTGACGCATG

ACGTAACTGTGcAAATGCAgCTCCAACGCAGTACTGATTtGAACTGTGTCTCGGCACCtA

TGAACTtCACGTACAAGgCGACCGACTTTGATCCACACAAAATTTttGCCAAGAAGCGCA

AGATGACCTCGCATCAAGATGACcaCGAGCTTCATATGATGAGTGGAGCTGATACtGCcG

GACTGGCGAGTAACGTAGCcATGGaGCCcaGAGAGAACTTCTATgacccagatttttttt

ggttcagcttggtaacttgcccccttttcgagagtccccttacgaactcgttgagccgct

tgacttcgcgacgcggaatgaggttaccgatttcttgcagctgcaagggttgaacttcaa

cacggaccagacctctgatgaactgtttgcctccagacctgtccaatagttgaagaggta

gctcaccctatgccaaa

>contig00534 length=505 numreads=7

AATGTTCTCATCTGcATCTGCAAGCCTCTTTTGCAAaTGAGCTtCATCCTTGAGCTCAGT

TGATGCTGGGCCAGCCTGTTTCCTCATGTAGGAAaCAATACCTCCAGCTTCACGGGGTCC

CTCATAGTCACTAGGAGTTCCACTGCGAAACACCTTTAGGGTAGGATAACCACTAACCCC

GTACTTACTGCATGTTTCCTTGCcACcTtCTGtACAGTCCACCTTTGCTAAaaGAACTGG

AGGCTCATTGGACTTCAAAGTAGTTGCGGCAGTTTCATACTCTGGAGCTAATcgtttaca

gtggccacaccaaggtgcaaagaactccACCaaaaTtAaTGTCCTTGTCCTcAaCTTCGT

CTTTGAAAGTGCTGTCAGTTAGATCCAACACATCGGAAGACAAAGCGACTCCGACGAGGA

GCGAAATaaTCccAAAGtaCTTCATGGTAAATGTTGGAGCAGTAGTgggggatcgagacg

tgcgcagggctacatcaacaaaacc

>contig00535 length=576 numreads=7

ACCAGaTGGGTGAAGTTCATgAAGcAGCtGTTCGGTGGCTtCTCTaTGCTGCTGTGGaTa

ggggCggTGCTGTGcTtCTTCGCgTTCGGCaTCCGcTCAATcAAcgAGAAGGACCCaGCC

CAGGACGAGCTGTATCTTGGAATCGTTCTCACTTTCGTTgttgttgtgacaggcatcttc

tCTTACTACCAGGAaGCCAAAaGCtCGAaGAtCATGGAGAGCTTCAAGAAGTTGGTGCCT

CAAGAAGCCGTGGTGTTGCGAGATGGCGAGAAGACGAATATTAATGCCACTCAATGTGTG

GTCGGAGACATCATATTCGTTAAGTTCGGcACAGGGTACCAGCTGATATGCGAGTCATCG

AGTCACGAggCTTcAGGGTTgAcAActCCTCcTTGaCGGgAGAATCTgaGCCTCAGGCTA

GATCACCGGAATTTTCCAgcgaaaatcccttggaaaccaaaaacctagcatttttctcca

ccaatgctgtggaaggaacggctaagggtatcatcgttagaatcggcgacaacaccgtca

tgggtcgaatcgctaatctggcttcgggacttggct

>contig00536 length=242 numreads=3

ctcgtacgggaatcgttcaagaggcgcagggaattgatgaagacgtcattcaaggagatg

ctcggggagtttgtttatgcgacttcaaaaGAaTTCGTGCGATACGGATGCGATATCaGA

ATtCcTGgaTGAgTCGATtAAAGgTAaCTGCgAAGGGCTCATGGTCAAGACGCTGgAaGT

CGATGCcaCATaCgAAaTCGCAAAGCGATCGCaTAaCTGGTTGAAgctGAaGAAGgACTa

Cc

>contig00537 length=242 numreads=3

AAGCAGTGGTATCAACGCAGAGTCTAGCGATGTCGGACGTATCGAAAGCGCGCGAAGCCA

CGACGCTTCCGATTTGATCCAGGACAACCAAGTTGTAACCACGCTTCTTAGAGCAATACT

CAACGCCGTTGACTACGATTGACGCAccgCCTCCATAGCCTGGATCGTCTGCGGCCTCAC

TATGCACCTCAAACTGAACCTTGCCTAAGAaGGTGaGAaCGaCTgttGGGgTAGCAAAaC

AA

>contig00538 length=838 numreads=15

aaCAGATCGCGAATTCCCTGTGAATAGTTTAACTCGATGTCAGGCGAGTGCGATAGACCG

GCGCTTATTATATTTTCCAGAATTTGCTtCGCCAGTaGGCACGTAGgTGAGTTTTCCAGC

GTTATCAAATCTCATCAAaGaCCGGAAATCTTGCAGTTCCAGTCTTGCCAAAaGTGAGAC

CTTTCGACCTCTCAAaGATGTCGGCAACGCGAACACGTACTtGACGcTCGCTAACATCCA

cGTCACCCATAATAAACaGCAGATCCTCCTTGTGCTCACCATCCAGTAAAACTTTGATAC

Agcattctttcagcttctcctcaagcaaaggatccacattgtagccatcgtctccaaggg

aatacacgagcacggctagcattagaacatcTACCACGCTAAACCTTGAGTGGCTCCCTG

ATACGAGAGATAGAATCTCCGAGAACGGATTGACTTCCTGGTTACCAaGaTTCAAAATCA

TTATCTTCTGCACGCCAATCATTTCTTCAATCTCTTTTATTTCGTCGCCATTAAAAGTGT

TAACGAACGCAACCACGCCCTGAAGGAAACCGCTGTACTTTTCGAAAATCTTCTGGTTAC

TCCGAATTTTTAAaaGtAAATCGCACAGAACTGGCAAAGATGGCTTCTCcTcGCTtAAGG

AAGCGCcTGGAACCTCCGcTtGCATTATAGATTTTATcTtCCCACAGaGaTAAACGATAC

AATCCTTTTTGGACTTTGAAGAAAATACCttGAGGAGCTCCTGAACTAGCCTATCGTTAT

GGTGAGCAAGGCAACCCGGGgAACACGGTTGCTGGGGTAACGTTTCCGCTGCTGAAGa

>contig00539 length=340 numreads=7

CTGCCcACGAAAaGGCCACGCTAAaCCAACGTCATATAGACCACGCATCAGCCGAATAGC

GAAATATGCGTCGCCGTGTAACCTATCGATTTCTGCTTtGATCTGCATGCGGTAGCCGCG

CATGGATGTGTAGAATACCGAGCTGTAATATAGCAAGGAtGTACATTCAGGGCTACGCTT

GCGGATAaCCCATTCGAATTCGTCACTgACCACGATATCGTCCGGATGcgctgagacaat

taactgcagaggcgctgacacctctttacttctgttgTAATATGCACCTACACTTTGAAA

CTGCTCATTTCTAGATGGAGGAGGTTGCATGCACTCAGCa

>contig00540 length=1334 numreads=48

TCATCAGCAGACGATCCATTCcAACACAAATtATCTCGGACAATtCGTCTACTTTTtCgT

CCGCCGCACAGCACAGCTTCCTGCTACAGCACAAGGTCAaGTGGACATTCATCCcTAaGC

GTgCGCCTTGgTATGGCGGGTTTTATGAAcGCATGATTGGAATGACgAaaTCGCTGTGGA

GAGCAACGgTtACACTAGACGAGCTTTCcACCGTCGAaGCAGAAGCAGAAGCCACCATCA

ATGAtCGTCcTTTGACCGCCATAACcAaCGATGCCGATGAACCGACACCACTCACCCCTt

CGCATCTTATCCATGGCCGACCCTTGAAAATCCTGCCGgATGCCGAACTGAaCACGGATG

TTTCaCAAGATCCTCCATACGAACCTTCGCACTCTAGCATCATCGTAAAACTCAaTAATT

TTAAGCTtCTCGTTTCAGCTTTCtGGCAGCGGTGGCAACACGAATACCTTCCGCTGCTTC

GCGACTATCATGGACGGACAACGATCCGGGGAACGACACGGAAAAACATCGTAAAaGTtG

GCGATGTGGTGATCGTGCACACTGACAACGAAAAGCGATtACGGTGGCCACTAGCGAAAG

TGGTCGAGCTACATACAGGCGCAGATGGGTTCGTCCGGTCAGCTACAATACGAACGTCTA

AAGGTCAGCACACTACACGCCCGATAGCCAAGCTGTACCCTcTTGAAATACCAATCGCAA

CGAACGAAGCGCAACACTCTCCTTtGAATACAACAGCCGAATCAACGAGCAGACCGGTGA

GAGAAGCTGCAGACCGAGCGTTGgCCAACATATATCAACAACAGGCGATATGATCGCCTc

GCACTCTCTTTcGTtGgCcGgCgggAgtgTcATGGATtgCCTCCATGATGGGACCTCCGA

GACcTCGGCACGAGGAAGCACACTGAGAAAGCCAGTTTGAACAGGCTTTCGAGGCCCCAC

GTGACCAAGACTAAAATCCAATGCTATTGGTGGACTTCTTACCATAaTTACTtCCTTGTT

CTAGTACAGTCTTGGAGCTAGAGTAAATGTATTGCAATTGGAGGACTTCCTAACGATAAT

CACCTACTCCTAAACGTGCAGTACTGAAGTTGACCGAAaTGGTTTCGAGTtCTGACGACC

TTtCtCTTCCATTGGACAGAaaCcGTTATTGGAATGTGATTGgTGgAaCAGCAAATGAAA

CTCATTAACTCGTACAGAATTTGTATATAAACCTTGGACTTCAGTAAATTTAGATAGATC

GCATTTAGTGGAaCATATCGCAATCAATAACcAAAAAAAAAAAAAaaaGTaaCTCTGCGT

TGaTACCACTGCTT

>contig00541 length=461 numreads=5

AAGCAGTGGTATCAACGCAGAGTACGCGGGCACCTGCAAATGAACCGATTCAAAGCCAAG

AGCGGGAATCCCGGAGCCGCGGAACAAGCAGCCAGCACCAATCAGCGgAAGCGACGCGCc

GTACGTcACAGCTGcTCATGCgACGacAgTTGCAACCGTctCCCCAAgaGCTCATTCGCC

AAAGACCTTTTCGCACTCGCGCACAGCTACCATCATTTTCATTGGTTCCTGGTCAAAGTA

aCGTTGGCCCTTTTgATGAACCTGAAGACTGCACTGTGCCAAGTACTCCTACcTtGTTtG

TTCCGAAAaGAaCAGATGGATTtGCAGAGgCTGTtAGCTCACCCCAaGTTCGGTATCCTA

TGTTTTCGTTTGGTTCAGGGGACCAGGTTGCCTCGCAAGTATCTGGCTTGGagCAGCAaG

gAAATGCGACTTGATGAAGCTCGAGTTGATCTGATGGGCca

>contig00542 length=1167 numreads=18

TGCCTCcATCGCaCGGTGGTAGTGCGCCATGGCAGcACTGAaCTTGTcGGAAaCCGAGCT

ACAGTTCCGGTCAGCCAAGCCCGTTaTTTGcTTGTAGTCGATATACTCGgCCGaCTGgtG

TGTGTCGATGAATCGAAAAGaCTGGAaGCGCCGCTcGAAaCgAaTTTGCTCATtGTCGAA

AAACGGCTCGGGGTGTTTGACTTTtCCTTCACGgtCGAAGGCTTCGTAGGCCTGAaGATt

GCCCAAGCAGATTTCcTTCATGCCGGTAAAGAGGATACGGAGACTTTTGTGTTCGTTCAC

CATGTCCtggCAAGACGTAGAAATTTCTTTCTTTTTCttcttgtccttcttgctgaattt

cttatctgaataagctttgtagtcaagcaacagtatgctcgcacgagaaagacAATTATT

CGCCCATCCGAGCAGGTAATCAAGATACCAGTAgacgtagtgaaactcgtgaggattgaa

caagtctaattcgaagccaagaaaaatgtggtgaatcatgaTGGTCAGTGAGTGATACAG

AACCCACGaTTCAAAacATGCTAAAtGTTGTTTTTGCTGTCgACAGTTTTAACTAACTTG

TCCAGTtCTTGATCAGCCTGATcTGCGTCTTCTTGGAGGTTTCCCATTtCTTCGAGAAGC

GATGCTAACTTTTCGCGCTGCCGAGCACGGTTGTGTCCGTAAGCTTGGATGAGGgATACC

ATAGCCTGCGAGGCCTTGGCTATGAAATTGCTGGTAATCACTTTAGCTTCTGgCGCTGTC

AAAGGTGTTACGTCacacactgccagagaagcattgaacaaCTTTATAGCCTCTTTAACC

ATGTCCGGCAAGGCGCACCGACcGGAAAACgCGGATTGTTCTGGTAGACAATCAGCTGAA

GTAGCGACCTGGACAACACACAAGGCACGCGACGGCTGAAATCGCAGAGGAACTCCAGAa

TATGATGCAATGTGATGCATTTCGTCACTGTGCAGATGTGGCCCAATCTATCCAGCAGAC

CTTCCATGTGTGAAACTGCCTTGTTTCTGTCGCaaatttccgactgACGCGGGAAAGACG

GAGGCAAAAGCCTTTGGTTGACAAGGGAATCAAATCCGATTACATAATATTCTTTGCTGT

AATCTGGCTTGTCGACAAGATCACggg

>contig00543 length=261 numreads=9

tCGTTGCTGTAGGCTtCTTCATtCgCTGTTCAaCAAGGCGACTGAGGTGGTCCGACCAAA

TGCTAGTCACCTGCGCAAATAGAGAAAATCCTGGATCAGGCTTCTTCTGCTGGAAGAATA

CATTTGCCATATGGAAGTATCCACCAGCCGTTTtGATATGATCCGTACCAAAGGCGAGCG

AAGAATAGTAGATATCATCAGCAAAATGTCGTAGTGAATCAGCGTAGTTCTcTtGCGTGG

CATACAtCGTTCCCGGGTTac

>contig00544 length=238 numreads=3

GAGCATCTTAAGgAGAGGAGTtGTATTGTTGTCTCTGTCTtACTCTtaTaCTTtAtAcAc

GAGTCTCTtCgTCGAAAATTTcTCAGATTTCGGTTGTtATAGCATGTACAaGTAAaCagA

AATTTACAAgACACCAACGGGAaCTTCAGGCAAAGTTTCGGAAGTCGTTCGGAACATTAA

Atgaagacccttgaatatcgcctacaacagtgcaaacaggagctcaaagcgtgctcag

>contig00545 length=494 numreads=17

CGTCGTtGGCTCCCAAGCGAAACCACCTGTGATAGTATCCAGCGTTCTTTCTAGTTCCAT

CCACGTTAAGAGTTtCGCtCTCAATGCCGTCGTCCGTCAAaTTAGCGCATAGTTATCTTT

TCCTGGAATCTCTTCCATCAACTCGTCCAGgAaGTTtcTTCCCATGGgCTTCTCTTGAAA

TACACTtGAGTCCATCGTATTTATTTGAAACCTGGCTGCTCTGGACAACAAGTGTCGGTA

CATACTTTTGTCACCCATCATTCtAGATGAAAGATCCTCGACCACACGTAAACGTGAAGT

TGGTAACAACTTTTGAGTCGGATGACAAAGGATTCTGTAATTCCACTGGGCGTACAAGGG

AATGGAGTCGTTCAAGAAACCCATGATAGCAGTGGGTAGAAAAGCTAGATTTTCCAACCG

GCTCTTGCCgCCGGACATTTCAGCGAACATGACCTTcTCTTGGaGTTCTtGCCAAGttCg

CGCGTCtATGAaaT

>contig00546 length=240 numreads=1

agcatggtaacgtgcgttctcttttcgttgtgtggctttagttttttattttctgcgctt

tcttgtaatttcagcatggttttgttctgtctattttcttgcacgatgccgccaaagttt

cacttactagtattcggcaaatttttgccgcgtagatctggcgttggtttggtatcggcc

gattgttctctctcttttgcgttgaaattgttgggattggcgccttggcatttcgtctta

>contig00547 length=240 numreads=3

ATCGGCACGACGGCAAAACCAAGCCCAACGGAGaaTTCTAGACCGCTCAAAAGACACGCA

ATAAGATGATCCcagCTCACGAGAGAACAaCaGGGAgAAGCAAGGCACGGGGGATGTTGC

TTCACAAaCAATGCCTtCCCGTTTGtACGaTCCCCATCcGcATGTCCTTTCTtctCTTCT

TTCACATTctCGCGACcTcTCCacTAaaTGTtAATTAGTGTTCGCGTGTGACATCCTTGA

>contig00548 length=241 numreads=1

aagcagtggtatcaacgcagagtacgcggggtagagatctcttagtttattatccttcga

gagatcaagatttttaagagtctctgacatcggccagaatccgttctgatacatctagta

tttgaccaggttcacatcatgtgatggtatttgtctggaaaaagttattcactgacagtg

gtttatttccaaattttgtctcctttctttgcatagaccagtttatgtgtgatggatagc

t

>contig00549 length=240 numreads=1

aagcagtggtatcaacgcagagttacgcggggatgtggccgatgtctggtttggtcggac

gatcattaggtcacgatacaaattgttttatgaggccgcgcaagatattattgatgggag

gataaaggcggaagaagcctccaaaagtgttcctgaattacaaaatcttgataagattgc

attgcatgaaagatttgagagctgaagaaatccctggttgaaatgatggcgatcgcaagg

>contig00550 length=209 numreads=2

cGAAACCGGATTACCACGGGTCCGCGCAAGGGCAGGACACATCTTCAACTTCATGGTCCA

AGTTGAAACCCATGTCAGCTACAGGGATGACGTCCGCACTCCTTaaCCGAGCGCTTACCG

AAACTTACGTAAAGAATGAAAGGCAACTTCTtCAGAAGGGAGTAAGCTATCTTCAGGACC

CACTTTTTGGCCTCAGCTTACCTGCCAAt

>contig00551 length=795 numreads=41

ttAAggTcGGCCaTCTtGtCcTTCGGCGTCcACTGCAaGTTAaCAATgCAGAGTTTCGGT

CGGCGCGGCTTTttCTTGTCCATCGACCACAGGCCATGATAAGATTTtAGAACCTTCAAa

CTAGAACCAAAGCAGATCACCAGATCGGCCATCTTTGCATTGTCAAAAGCAGCTtGCCAG

TTTtGCGGCTGCTCGAGAGAACGCTTCTCCCCAAAATGGACGATGCTGTCAACAAGGGCT

GAGCTACACGCGTCACATGTCCTGCCAGTCAGGTGACGCCGCAAGTTtGTGCGCTCCGTT

ACATCAAACAGTCTCCGGTAAGTTCTCTGCGGCTGGcAaCtGGTaCaCACcTCGATGAaC

ATATtCCcGTGgATTTCAGAtAGGAGTtCACAaGGgATACcACTCCGCAgATGAAGgCcG

TCACAATTCTGAGATACCACATGCCGCACTATGCCAGCTTGGACAAGTTTTGCTATGCAC

ATGTGTGAGTACGTTGGCTCAGCTCCAGCAATATTACAAGcGCTAATGGTTTCGCcTTGC

TGCAATTtCGTCCACACACCTTTtGATCCTCTGTAATCTGGAATtGCtGCGGCAGTGCTG

ATTCCTGCACCAGTATAAGCAAcAACATGGGACGCAGAACtGAGCATTGATTtCAaCTGT

CTACACTTcGTCCTCAGCTCTTCCTCCGAaTCTTCAGACTCTAGTAACTGTGATCGCAAA

GCCTCATTCTTGGTTTTcTTCTGGAGACATtGCTCgTATTCTTGCTCgtGTTTCTGATGA

ATGCCTTGTCCACtt

>contig00552 length=318 numreads=3

tccaatccgctttactgatatacaattagtttgaagttcgccaaaatagaaataccagtg

gtcatcttgcgcaCAAcGACcTATGactGTTGCgATtGgCACTGGgAAaGGgCTCTTTCG

AGtATGGCCCtGAGCTTtcTTcTTTTTTCTTGGTTGTCTCAaTAGTTGATCTtCCaTgct

acaaaatgatcaaatagcatcgttaaattaatcctagctattccattgaatcacataaca

ttgaaccctcaagagttgcaattgtgcctaaaaacatgcgttctggagatttgatgaata

atgattgacaacatacaa

>contig00553 length=192 numreads=2

aagcagtggtatcaacgcagagtacgcgggcagaaagtagacatggatacccgagccggc

agctttgaagttagcttcggtgctcttgtctctctgtcgttagttggaaatctcattgac

cagtggcattctctgaaccagcttaacaagattcagaacttaactgacctcgTTTtAAAa

GTtAAtCCTTTa

>contig00554 length=1262 numreads=43

GAATATCGTTATAGAACTGAAGGTGTACGATTTattatggagcttgggaataagcttggc

ttgcgataccttacaatggctactggcagcgtttatttcatcgcttctacatGTTTCATA

GCTTCAGGGAGTTTTCTCGGTGGGTAGTGGGTActGCATGCTTATTTTtGGccggcaagg

ttgaagaGACccAAAAAAATaGTGGTGACATCTTGAAaGTTACCcaGAAGACtGTTACTG

ACAACCAGTTTCAAaGTTTtGGCGAGAATCCACGAGAAGAAGTTATGATATGTGAACGTG

TACTTCTTCAAACAATCAGATTTGAtctGCaGgTGGATCATCCTTACCAATACTTAATTA

AATATGGAAAGCGTCTGAAAGGAAACAGAGACAAAATCAATGAACTTGTTCAAAAGGCCT

GGATTTTtATCAATGATAGTTTGGCAACACCGCTGTGCTTGCTATACAAGCCTCAAGTAG

TTGCTCTTGGCGTGCTTTTGATGGCAGTTAGACTTTCTAATCAAGATATTCGTGACCTTA

TGCATCAACCTAAGGTTGATTGgTGGgATGTTTTTTTCcTGgTGCAACAGAAGCTGATCT

AGAaCATATATGCAAaGAATTGCTGgAGATGTATGATGGTCGTATCCCTAaGCGACGATA

TGAGCTAGCTGACAAaGGGCAACTTTCGTCTCCTGCAAGTAGTGATCCtAAGTGTTCAGC

AAGCCCTAAATCAGAGAGTCCAaCAGCTATTGAAAGTCCAGCACCAAAGAGACTtAAAAT

tCTCACACCTACGTCAACGgCTGCAACTTTGGgCAGTAAAGTAGAAGGTAGCGCATCAAG

TGCATCTGTATTGCCTGGCCACCTATTGAATATTGCCAGCgCTGTTGCTTCCAATTCAtg

TGCCATGCAGGTGCCAGTAGCTGTTTCATCTTCATCGGCTTTGTTAGAGCTGCCCTTGTC

ATTGCCAAATCAGgTTGCAAGTgCcTtCGTTAACAGTTTCCACAACTGCTGTGCAAGTAC

ATAACCCACAGCCagTACTTCATTCAAATATGGAGCTTTTAAATTCAAATGCTGTGTCcA

TGGGTATGCCcTTACCAGACAGAGgCTTtCCTGTGTTAaGTAACAATTCTGTGGCTAGCT

CATATTATCAGTCGgCACAAATGAATGGATATGGGTATATgCCTGCAGGACAGCAAATGA

TGCACTCATACAGCTATGGTCAACAGCCAATGCAATATCCTtCcGCAACaatctatgttt

ag

>contig00555 length=218 numreads=3

tACTTCCAAGATTATTTGAAATCTTTCTTCTGTTTCGCAGAACTACCGCAAAAACGTCCC

GaTCACAcTtGGCGcAGACAGAAGCCTTACAGCAACcTCTTCCaTGGAgATtCTCcTGTC

TGCTTGTAGaGTtGTCgCCTGTGCTGATCTTCGTGCCAACAACACTTGACAATTTGTGTT

TCTTGGACAGAACAAATATGTCATGACCATTAGGGGCA

>contig00556 length=641 numreads=13

TggCGATTTtGACAATTTTCTTTGATTTCCGCATTGTCGCAGATAATGCTATTCTGAAtG

TTGCAACCGCTTTTATCGTGACATGATTCATTATGACACAGTTGCCTATCTTtACTTTTG

AGCcGATGCTACAGTGATTtCCAaTGACGGACTTCTTCAcTGAAGaCAGCTCGCCTATCG

TCGTCGACTCTCCAACAAGACTATCAGGTCCgATCTGCGATTTCTCCTGCACCGAAACCT

TcGaGTgCACTCTaGAGATCTCAACACCAGGAGCcAGCGTATCAAAAAGCTTTATAATCT

GTCTATTTATATGaCcGTAaGCGACAaGATTATTGACTCGCAGgCAAaGaCCATCaaaCA

ACAAGCgcATGACATCGGATGCtGTtATCACtATTGTCTTGCATATAGCCTGACCATTtC

TTAGTTTGTGTACAAATTTCATCCGAAGACGCAAAGTCAAATATATCAACACCACTGGCA

CTTCTGCTTTTCGGCTTCGAGAACTGCTTCCTGATAATATGCGGTATTAGATCGGATTtC

ACACTTTCAAaCGCGTTTGATTTACACAGATAGTCAATTATCCATTTCTTAATGATGTAC

AAGTGAGAaTCCACAAGATCGGTTTGCAAACGCACTTTGGG

>contig00557 length=274 numreads=11

AAGgCAGTGGTATaCAACGCAGAGTCGCCCAACTTGAAACCTTtGCTCAACAATACAAAG

GAAGAGTTGTCCGTTttCGAAACGACCGCTTTGGTTTTCCTGTTTATTTACGACAGCGAA

GAGTTTGCCAGAAGAGCATTTTTGATTACCCATTCAAATGACGTCCAAGACTTCGAAAAG

GTTAAaGaCAGaTTgAAagAAaaCGAAGAAAaGaCAGTTttATtAAAtCaGAAATGCAAT

tGtgAAaaGAAtCCTTGTGaatgcgacaacccgg

>contig00558 length=274 numreads=2

aaggggttttgcacgttctgttcttttttaggttcgtcaagaatctcatcttttccacaa

gggttcgagttctgatcaaagtagggctcttggtacggtggcgaatacgtcaagacgcat

ttttcctctGGACTGCTCACTGAAGTTCCTTCTTTTGTGCAGTCCACGGAAGGGGACatG

ATCTCAAGAATCTCTGCACGCTTTCGTAGCCGCTTGTACAAGAAGAAGGCCAAAATTCCC

ACAACAAGGATCCCAACTGCCGCACCGACTGCCg

>contig00559 length=243 numreads=1

ataaatcgggccagaaagagcgatgaaactgtgcaccccatgtggcatatttcgcttgta

gtcagacggtacccatggcccaaggaacacccaagcgcctttaaatagcccctctcaacc

actttcactgccaataagtgagactgagctagacgacaacttcttgttgaaaagcatacg

aatctcttacccttgcagcccattaattggaaacttaaaatattaacagtcttagaaaac

aaa

>contig00560 length=1231 numreads=33

GTTGGAGGAAAGCGAAGAGTCCGATAATGAAGGTGAGCAAACGGAGGAGGGTGACAaGGA

AGACAAAAAGCGAAGGAAGCACACAAAaGAGAAAGAGAaGgCTCGACTTGAAGACCATCG

ACGaTCTCTtATAAGAaGGCACCcGCTGCATGTGGCCGTCGGGATAAaGTGCAAAGACTC

TAACGAAGTAAACTTGAAATTTTACCACCTCCCATCTATTCATATCTGCACGGTGGAGAT

TGATCTGAAAATTTGTGACTCCGCGCAGCAAGCTCCcaTTTTGGCTTCGAGCTCACTGaT

GTCTCCAGCTATGATCTTGCGTGACCTGATGTGCGGTGACCCGGGGTTAGAGTCGCCGAA

TCCTgCcAATAaGTATCAGTTGGAGCGACTGCGCATGGgCGCCTTTTCCGAGTACATAGG

TCAAGTTGGACATCCATATtACTGGGTGCAGTGGATTtGTGGCCTtCACTATCTCCCGAA

CGACCCGATTGCGGCGTACGAAGCCAATCCGCTGGTCAGCTATAATCACATgCAACGCGT

AGTCAAAGCAATTAAGGCCAGAATATCTGCACGATTGGCTCTGCAATCTCAGTTAGCCCA

GTTAGAGAATCTGGTCATCCCCCCGCCTAGTCGGAAGAGCGTGCCTAACAGTTTCCCGTC

TAAGACCGTCTGCCAGTTGTCGTCCTGGAAAaGCAtAACGTTTGAAGACTtCAGGGTGTT

CCCATTTGCTGAAGGAAGCCTGGAGATGCAGATGGCTGACGAAAGTTGTGCTTTCTATCA

GGCACGgTTtACGCGGGAAGAGTCACTCGATGTTGTCGTTGTGATCTACGATAGCTATCc

GTCCAATCcGCCCcTGTtGCTGGTTGGATCTGTGGATAAGAGTTGCAAGTTtGCGGACGA

CACTTTGCGGTGTTTAGAATGTGAAGTAaGTGCCTATGCTGACGAaCTTCTTAAACtCGA

TCATCcGAaCaTGGTCTTGTCTACTCAACTGAGAAAGCTTCAGGTTTCGTTTGACATGTT

GGTGGAaaCTGggAaCcAaTCTCTCGCCAaGGAGAaGcTTtACACTCGgCGAGTTAGGGG

ACGAGATCGAAATCGCCCAtAtCGTTTCCATCaGGACGGTTATTTcAcTCATCGTTgAAC

TGCTGTTGGgTGGAGTCATCACTCATATGTGCTGTTGCACTAATACGTGCTATGCACTGG

TtGTTTACAaGAAAATATCGCCCGCACACAC

>contig00561 length=195 numreads=3

GGGtATGTGGGTGGATtCTATGGAGCctGcAGCGAAgCGTTGATGTTATCcGCGATCgTt

AAGCATGGaCCgCTaGcGGTATCTTTTgaGgTCTAtGAAGATTTcATACATTACgCTGgC

GgAATCTATcACCAcAgTGgTCtCTCGAatAAATTcgaCCCCTtCCAgCTGAccAACCAC

GTTGTAGTTATTGtt

>contig00562 length=201 numreads=3

gCTCGAAGAAGAGTCTAGAGAGCTTGAGGCCGAGCTTGATGAACGCGAGCAAGTCTTGGC

TGATAAGAAAGAACAACACAcgaaggtcaagccggagctcgagtcttgtatgaacgaaat

cgccgagatgtgagACCACAGCCTCGGACGCCATGATGAACTCACATTTGATGAGACGCG

ATGTCttCCATTTTgCGGACA

>contig00563 length=647 numreads=11

GTCTGgAGGAAACACACACTAGCATACCGTAAaCCTaCCATTtACTCcTCAAGCcGCGAC

GgTTAATATGGGCTGcGgCTGCCCGTCaGCGTACAaCGCcAGTAgcTAaGTTAaTTGCCT

GTGAATAAATCTTtCTCATTTTATAGAAAATTTCAAATGATTGAGGATTGCTCGAGCTTG

TTAaGGATAGGgATTGTGATAGGGTAGGGTAAGGACTAGTATTATGGTTAAAGTTTGAGT

TTGGGTTACATTCTGACTTAGCCAACAAATAAATAAAAAAAGTAAAaTGCAAGCCAAaGC

TTCTCcATcaCTAACGACTGCATAGCAGTtGCCGTCTTGtAaGCATAACGGCCGCGAGGT

AaGTCACATCAGATCAaTCGATaTCTGAGgagTAAACATGAGATTTATACCCTCTTAGCG

CCGACAAATCcAGCCTCGTTGCGCTGACTAATTTTTTGGATTTCAtCAGCTATCTTGTTA

ATCCCAAaCAtGGAagAGCAACGtAtgTAAAAAACGAAGgCTCACcGATGTTGCAGAGgA

CTtGTCGACAGaTCACTGTGATTtAATTAACGTGgTCAGCTACTCCATCGCAAATGTAAA

CAAACCACCTAGTAAACCGTgCCCACTCTGCGTTGATACCACTGCTT

>contig00564 length=422 numreads=7

tactggaaacttatgaatgctggagggaagggaatcaagtgatggttatatttcttatct

gttcttatttttcggatacctctttatggcattgcagcttcaagggtgtaagtcacagtg

aaccatttccattttttgCGTATAAAAACtGGTCAGATGGCAGGGATTTTTtGTGAAGAG

GTAGGGCAAAAAaTTTCCGTCGTGTGCCTTTAAGAACCAATTGCAAACATTGTTCTGCGC

TATTTtCCGTGTATTCTTAAGGAATTGATAGGCTAACACTCcATTTtGCATtGATCTGTt

GATTTTTTTaTTTGAATGGTTtATTGCgTTtCTTGTGTTtATTTTTACAGGGTTTATTTT

gCTTTGtAGTGTGATGAGGATGTTTTGCCTTGTAATtAGAGGATGTTTTGACCGCAAATA

TG

>contig00565 length=241 numreads=1

gtggtgtacattcatgcttacaggattgttgtggcgcgctctggcgttctcacgggtaaa

aatggatcggtgtggaaggctctagggaagacccttagattcttgtcgcaaggtctcgtc

cggacaagctaggctccatctgtcatcacttgtggtaaacgatgcagtacgcacgcgcga

aacttaggttttttaacccactgtcattgtttcgggttaacatttacaattaccgtatat

t

>contig00566 length=931 numreads=26

CGAGGGAATTGAACTGCTCGCAAAGGTCACCATCCACAACATTTTTGACTGGTGCATATG

ATGAACGATACcATAGATGGTCTCGGCCACACAAGGgTGGATATTCCTGgCGCATATGCA

TTtCCAAGTGCTGgAAAAAGTCGATGTCcTCTCGGgACGTGAATGGAACGAGCATCCCTA

CACTGCCGGACACTGTtGTATAGACAAGCGATTCTGCGCcAGCAGGAATAAGGGTCGCcT

TTTGcAGTGACAAAACaGTCTCTCCAATGTGATACATATTTATGgTgTCcGCCTtCTGAG

AAgCACCGTTGaGCAGACCTcGGtCCCACAAAGCCTTCACACCAGTCGGGTCTTCCTCCA

CATCGTCTTTAACATcgggtggcagcctcacaacaaacacattgccgaacttatcagctc

ccgcaactgagtcgtaatccaaaagacacgCACATGTTAAGAATCGGGTGTTTACGTCAT

CCGCAAAAaGTACCAGCTGGTTGTCTTTGCGTTTATACTTCAAGAAATGAAACGAGTCCT

GGATATCACTAACTACTATGCGGTAACCGGTGGTATTAATCTGTGTGATGAACGTTGATA

TCTTCTTGTTCTCGCATTTCCTCAGCAATTTCTTCTTGCCGAGATCGTAAaTTCGAAGgA

TCCGACCAACACCAACTAGAAGACGCCCCTGAAAaGCGCAAACAGCAGCTGGGATGTCAT

CAAGTGGAGTTTTGTGAAGCAGCTCCAGCGTGgTCCTCCCGTAGTCGGACTGCGCTATTC

TGTATGTGTGAATGGAACCACCTTGACACTTTCTGGGgCTGAGCATCAtcTtGAaTGCAG

TGCCGACAaTTAAgAaGcGCTCTTCGGGACGACTCGAAAATTCGGCAATGCAAATGGAGA

AGGCGGCTTCATCCTGCTCCAGCTggATTTT

>contig00567 length=241 numreads=2

aagCAGTGGTATCAACGCAGAGTACgCGGGGTCGACACCGGAGGAAACATTCGCGTCACC

AAACCTCTCGACCGCGAGTCTAACCCGCACGGATTCTCGTTGGCGATCGTAGCAGCCGAT

AAGTCGCCGACTAATCAACTGATGGACAGCATTTCTTTACTCGTTTCGTTAACCGATGTT

AACGACAACGCACCGTCGTTTAGTGAAGTCTCCTACGATTTCTTTGTGTACGATGATGCG

C

>contig00568 length=238 numreads=3

AAGCAGTGGTAtcAaCGCAGAGTACGCGGGgCTTtCCgCCAACcGAGTCCACGCcAGtAC

ACGtGcGTGCTTGatGgcTcAGAGTCCtGCTGcTAAAGTtAGAAAgaTCTCAGATAATAa

GGAAgAAGCCaTGGcTACAGcTGaTTTCATGCTAAACggGATGTGTcggatttaaagaaa

atagtactggatgaaaagccatgtaaagtcgattcagggaatatcggcactgcacttg

>contig00569 length=203 numreads=4

aaatGggtCAACGATATGTCATCAAGAATTAATCCAAACGCAGATGCCCTTTCCACAAGA

GCcTCCTGAACCTtGTGaGAAACCATATCTCTTTGTGTGATTAACTCACTGGCATCAAAC

TGGGCCACCACAGCTTTTaataCTTCaGtAGTTATCGAAGgAAGCACCCGCTCATCGTAa

TCTtCACCTAAGccataatttaa

>contig00570 length=443 numreads=10

ACTCCTAAATCGGAGGCGGAGCTACATGCGGAGGAGcGtCTGGCGCACGAGACGACGCAC

GTGCAGTTTTTGACcACcAATCGCAAGGgACAGAAaCTCGTAAACTACGACAACCAGCAG

CTGGCCATCAAGCCGGAGGTATTCTtGTCAAAAGACGAGCAGACGATCATTTGCTACCAC

CCACCACCCAAGCTTTACCcTGTATCTTTCACCAAGCCTGCAGTTGGCGAAGCATGGTGg

CGGCCGGGAAAGGAAGAtCTCGTGAAGTATGGCGGCGAACTTACTTCGGAACAGATTGAA

GAGGTTAAAaTTTtGCGGCGGgAAAATCcGAAGTTATGGACTATCACCGCCCTCTCGAAT

TTGttcGAGACGAAGCcAGAAGCAATcAGgCcTcACGCCCGTTTGAGTGACCAGCAAATC

ATGGAGCTCGATGTTGAGCGAGa

>contig00571 length=381 numreads=7

aaCCaTtGCTCCTAGATCTTTTCGTATGTCAAACAGCTGCATCAAGACtttttCTACGCG

ATCTtCCGCCAaCAGaCTCtgTACGTTcGCTtGCCaGGACTTCCTGtACGCCACGCATCC

ACATGAATCAGGAaGGGAAATATACTTTCTGgCGAACTCTTTGTATTGAGCAAaCGcATA

AGTCGGGAaCAAAAGAATGGAGCGATGAGAGTATCTCGACTTCACTCGCTTTTCAAGAaG

cTCcaCCaCGTCAaGTCTGCACgTTAGGCCGACTACGCAaaGtGgCGTCtGAcGgCTTtG

TGCGaTGTCAAACAAATtATAGAGCAGAGCCTGGTtCTTATGTGCGTGAaaCcaGTCGaa

ttCGTCAAGGaCAACTACcAC

>contig00572 length=1602 numreads=87

tcgagaaGTCCGTTCTGTCTAAAgAAGCAAGATTTATgACGAGAGtcTTgCGGTCTCTGG

gCTCGACCAGGAAGAAGCTGAGTCAGAGCACCCTCTGCAAAATAGTCCATGTCTACGGAT

CGCCAGGAAGTAAAGCTACACTTTTTGGATTTCTTGACGCTGCCGTTGAAAATGGTgACG

AGTTGCAAACTCCGAGTAAGCCCAAGGgCCCGAAAATTCTTGCATTGCAACAATTGCCAG

AAGTTGATGTCTATTTGCATTTACTTGTACTGGTGTATCTTCTTGACAGCAAGTCAACTT

CCAAGCTGATTGATTGTGCAGACAAGCTGGTGGAAAAGGTTGCCTCGCACAATCGCCGAA

CGTTAGATCCCcTgaCTTCAGTCTGCTACTTCTACCAGTCTCGCGTATATGAGCTTGCTG

ATAGACTGGCAGAAATTCGGGgCTTCCTGCATGCAAGGCTTCGAACCAGCACTCTTCGTC

ATGATAACGATGGACAGGCGATGTTGCTAAATTTGCTGCTTCGAAACTACATTCACTACA

ACTTGTTTGACCAGGCCGATAAGTTGATTTCGAAGACATCTTTtCCTGACTCGgCGTCGA

ACAACGACTGGGcgcGCTACCTGTGCTAcTtAGGCTTTGTGAAGGCTATCCAGCTTGACT

ACTCGGACGCTGAGAAGCATTTGATgAATGCGATCCGTAAGGCACCTCAGCAGGCGGCCA

TTGGATTCAGGCAACATGTATCGCGGTTAGCTATTGTCGTACAGCTACTTCTCGGGGAAA

TCCCTGATCGGAAGATTTTCCGAGAAAAGACTCTGAAAAaGACCCTGGCTCCGTACCTTC

AATTGAcAcAAGCTGTGCGCTTAGGGAACTTGAAGCGTTTCAATGACGTTGTTGAGAAGT

ACAAGGATAAGTTCCAATCCGAGAAGACCTACACGCTGATTATAAGACTTCGCCACAGCG

TCATAAAGACTGGTATGAGAATGATTAGTTTATCGTATTCGAGAATTTCTCTGGCTGATA

TCGCTAAGAAGCTGGCTCTAGATAGCGCTGAAGATGCCGAGTTCATTATTTCCAAGGCTG

TCCGCGACGGTGTCATCGATGCCACTATCGATCACAGTCAGAAATGCGTGCAGTCCAAGG

AAAaCATCGACGTGTATTCTACGACAGAGCCTCAGAACGCCTTCCACCAGAGGATTAGCT

TTTCGTTGGATATTTACAATCAGTCTATTAAGGCTATGCGTTATCCTCCAAAATCATACA

ATAAAGATTTGGAGTCGCTTAAGGAGCAGATTGAGCGAGAAAGGCAGGACTTGGAGTACG

CAAAaGAAaTaGCTGAGAACGACGACGAAGaCTTCATATAGGTTTCTTCTGCtGgTtACT

GAGATGATTAGAATTTCGGAAAGTTATTCTTTGGGATTtGACTCTCTGTTcACCcTACAT

CATTTGCtATTGCGTGAcGTGGtAACACGACAaTATGACGTAAACCTAAcTTTTCCTTGT

CAGCCATAATTAaCATtCCGCTGTTTGTTTTTtCGGAATtcAGAaTTCGGAAacTaTACA

AAAAAAAAAAAAAAAAGTAACTCTGCGTTGATACCACTGCTT

>contig00573 length=384 numreads=6

AGGTGAAGAGGCTCTcAcgCCAGACGACTTGCTGTATCTGGAGTTCCTGCAGAAGTTTGA

GAAAAACTTTATCGCGCAAGGGCCATACGAAAaCCGTaCTgTCTTCGAGTCGCTCGACAT

CGGATGGTCGCTGCTGCGTAtCTTCCCTAAGGaGATGTTAAAGCGaaTtCCACAGTCTAT

TCTCGCTTCTtATTATCcTCGAGACGGGAAGAAGGGTCAGACGGACAGtGCTGCGTAAaC

TGgCACATTCGTGGGTCACGCTCGTATCCATTTACCGGGGAATGCATGAGAATTTCTTGA

aTTtGTAGAAACTTtATGACGTCTTCAATTTTtCCcGACATCTTTtGCtAcaTCAGTCGG

GtAATTTAtGtCTTACATaGAAAA

>contig00574 length=115 numreads=1

aagcagtggtatcaacgcagagtacgcgggtatccgcggtttcacataaaaacgatggca

aatcgatttttgttcgaaatttgcaaattcctatcaagtcgcaaataaagtttgg

>contig00575 length=250 numreads=7

cATTCTcTATATCCGGACAATCTGTTTACTtGCtCGgAaGATGGCTCTTGTTGGttctGG

gACGGAGCTTCCATGATTTCcGAGGCATCGTACACGAAGCACAgCCACAACTTGTTTGGC

GAGGATGTGCAtGAGATGCAGAGCGCCGTTTCTtCGAGtGTCTGGCTCTACGTTGACGCA

AACAaGCATCGAATGCAGACATTTTCACTAGTTCCGTTCAACAGGCTGCCGGTGAACACG

TTCGACGTAg

>contig00576 length=241 numreads=1

aagcagtggtatcaacgcagagtacttttttttttttttttttttttcgtatattgatcg

tgttttgtattccgcgcgctatatttccaggtagctaggacctgtttgaattgacagtgt

tgagaaactatttctgaggcaactgacctgtcgctggttatgcggcatctgtcatacggc

atccgtcaagcgtctataaaagcttctcggttctcggttgtgcagcgtcttcagagttct

g

>contig00577 length=407 numreads=7

ccGAAGATTGAgAGTCATCAcTaCTtGTtgAaGgTCATGCAGCctAGTTCcGTATGGaTT

GCCaTAAAagCCTCTGCtCCAAAGTAcGGTAAATACAAGGAAGAtCTTCTGGACCTGGAC

ATGACCATTTTtttAGTAGAGTCTCAGTCTGGgaGAGTAGTAGCCGTCAGCAACTCTGTA

ATtGACAAGAagTTCTGCCTtCAAGCTGACCTTGATATCGGAATTTACCACCTCTTCACA

TTTTCTTCTGGTTGCAAACTGGCCGCGGAAGACTCGACGTCCGGCCGACTTGGTTtGGCt

AAGGGAACTGGCGACGAGATGCGGCTGACCAAGGTGTGCCAAGACGCGCTGACGGAgATT

TTCTATCGTTgCGATCTTGACGgAAaCGgCTTCctcAGTCGGGACGA

>contig00578 length=213 numreads=7

TTAAaGGACTGGAAGGAAATGGATGATAGAAATtCGTtGATAGAGACTACCAAGACAATG

TTCAGAGAAAGTTGAAGTCAAAaTTGAAGGAAGGAAAaGATtCATCGCCTACAAGGCAGT

CCTCTGGTGATTCTTCATTAaGCATTGAAGAAACAAACAAAATtCGAGCCAAGCTtGGTC

TtAAGCCTTTGCAGCATGATaGTGtGCAAggTT

>contig00579 length=223 numreads=2

GATGTGCAGTTAATTCGACTtAGAGACATGAAGATGGTGCAGGATGAAGGAAGGAAGAAC

ACTCGACGCATCCTCATAACCATGCGGAGCGCCGACACAGTTGACGGCTGTGCTACttCC

GAAAaGATCATACGTcaggccattgagacagagctgccttcgcccatctttaactgtgga

gctcaggtcgagttcgataattttgatagcgcctcgcttacta

>contig00580 length=237 numreads=1

aagcagtggtatcaacgcagagtacgcgggcatgttaaggatccaaacttcaatattgtt

cagcgactccagaagcgcaattcgagagaccgggccggcttggcgatttctgacaagaaa

attgctggtgacatacttgtgacgaaaggctttgatgtcgcccaccagcctgccagtggc

gagagtaagcctgctttgaatgagcgatacgcctctgaaactgaactctacaatggt

>contig00581 length=228 numreads=5

GCAGTGGTATCAACGCAGAGTACGCGGGCGGCGTACCtGCGCcAATCAACAAGAGTGGAC

ACGAACGATGGGAATACGAGCGAGCCAGTAaGTTAACGACGGgACCTGATGAAAAGCTCG

CGGTCTGAACTCCTGCATGACCAcAGAAATGATGTATGACTCTGAAATTCtAACTCCGGA

TGCGTcGGCTGCAAaGTCACCGGTACATCTGTCTCTGAGtcacaagat

>contig00582 length=112 numreads=17

AAGCAGTGGTATCAACGCAGAGTtGGATATCAGCAGAGTGGATATCAGCAGAGTGGATAT

CAGCAGAGTGGATATCAGCAGAGTGGATATCAGCAGAGTGGATATCAGCAGA

>contig00583 length=719 numreads=22

aagcagtggtatcaacgcagagtacgcgggtttggccgcaagcctaatgtcgaccctgtc

atctttgaaactataaactcacaggatgcaaaagatggctcacatgtgaagtggaaacga

aatcaaattcttatgcgcgacAGAGcAaGAGCTTTTTCTAAACGGGCTTCAGAAAAAATG

ATAaCGCGGCATCGACGAAAGTATCCTCCAGCCATTTATCAAACTGGGgATGAAGTTATG

AtAAAACCcTTCATTAGCGGAAAAAtCATtCtGAAAGgTAAAAgCcGCCTAgCCTTtCcA

AgAgTAGtCGTGAAAGTAAAaGGGgAaTCGTaCTGGATCCGCTATGTAAACGACAAAGGA

TGCAGCAAAGAATCGTGGTTTCCTGTGAGTTCCGTAACATCGCGTACACGCAGCGCAGAA

GTGAAGCGTCGCTGTGATATtGAAAGCACGGACTCTGCAaAGAAAATATCTTCTCGagaT

AACCAGGGagCGTCCCGgACTCAATCCAGACCTAATACGCgAaCAGAATCCCAgTTGAAG

CGCGACCTCGAGGgAAGCCACTCGACAGAGCTTGCAaAGAGCAGGCAaCACAAATACCAC

TGGAGTTGGTAAGAGTATTGTCGGAGCGAAATCTCGACTATGTGGACGTAGGGAGGGACG

GgAaTTGCTTTTTCCGCGcAGTTGCCCACCAACTTTTTtGGAACGGAGctAAGGCACga

>contig00584 length=239 numreads=7

CcACAAATTCAAATTCTTGTCCcTTtGGTGTTCGGGtCCAGCAATCATCAATGTTAACAT

ATTCGTACCCAGCCTTCAGGAACCCATCTGtCTACCATATGGTCtGCCATTtGCTGgAAC

AAaTTTtCACTAaTaCACTGATCaGGGTAAgtCTGACAATCGGtAATGCAgCGgAATCTc

tCCCATTGAagCCAGCCCATGGGAGgtTCCTGGCAAcccattgtttaaagcagttccaa

>contig00585 length=350 numreads=5

CTGCTACAAAGGATAAGTATGAGGCGTCGATAACTAAATGGGCTCAGTACTGTAGTaTGC

AGGgCATTtGCcACACTACACCTCAAATTgAGCACATTATTtCCTTCTTgACAGAacTTT

TTGTCTCTGGTAAGAGTTATCGCACtATTTTGGGTTATCGATCAGCCATTAATGCAGTTG

CTAAGGTtGCTTACTATCcTGACATATGTCAacACCcACTcATGcaGCGATTtGTtaGAG

GGGTgTATAATATACgACCTCCCAtCCCAAgATTCtCAAGAATTtGGGATGTAAATActG

TTTTtAccACATTAAACAAaTGGGAGACAATGACACTATGCCCATGGTAc

>contig00586 length=773 numreads=12

AACTTTACTAAGTCTCTGCATCAAGAAGGACCAGGCCTTTTCAACTTGCTTCTGCGTAAA

TTCTGGTTCCCACAACGTGCTTAGTACGACATGAGCACCATTAACCCTTTCCTGGTTAGT

CATTTGAGAGTTGATCCTCCCGAAAGCCTGCGCTTCATTTAAATGATCTCGCTCAAGCaT

tCTTCGcACaGaTTCATTCGGGGGAacAATGCTCACCCaTAccTCATGGACAAGTTTGtC

CcAACCaGcctCcaGAAGCACTGCCGCATCTAACACGACAACATCATAAACACCTTCAtC

gCGAaCGCgCCCTaCAaTATTGGCAATCATtGTCGCAATTTCAGGCCACACTATGCTGTT

CAGCAGCATTAGCTTCGACTTATCGGAAAaTACAaTGGGACCCAGcTTTTTGCGATtGAC

AGTTTTGTCTTCGCTTAGGATAGcTtCGCCAAAGTTTTGAACTATAAGGTCATAAGCcTT

tgTGCCAGGCAAGTAAGCATCGTGACCAaGTTTGTCACAGTTTATCGTATATGCCCCTAA

ACCcTCCAGTCGCTTCGCTATGGCAGTTTTtCCAGTAGCAACGCcGCCAGTCAGCCCTAT

TAAATACGGCcGGGAACATtGAAGAAAATATGGAGGTTTTATGAGAGAACCCAGGCTGCG

CACTCTTGAAGCTgatgagctaaattttccttccttacgcaactcttcgggcaaatccaa

ggattcggaactaaccaactcaattgacactacatctagctcatgtagaccat

>contig00587 length=231 numreads=3

TATGTCTCcGTGCAATGCCCaaGCCatAAAGACAGAATCAGgTTCGCGGAGATTTTGTCA

GCTAAAGCATGCGACTGcGTtCCCTGCGgCGgCCATCTTGAAGCGAAATGAGAAGACAAT

AATTCGACTGCAGGGATGCGCCGATAGTCTAATCAgtGGCGTGACATACGCAGCTGATGT

AGTGGAGGACAGTTCCCCGATGGtcttccaagccattcatcacattcggtt

>contig00588 length=227 numreads=4

ATGGAGTTTCGCCATGACGTAATCATACCCATATTCCGATTCTGGTGTGAGGAATCAGGA

GTTATAATGATGAGAAGaCCATTTATTCGtAGAAGCTGATGAGCACGCACACAGCATCTG

tAGCGCTCCAAAGGAGTtGGAAAgTAaCaTAACAaCAatGaGAAAACAaCAaCATCAAAG

CTTTCGCTAAAAaCgTTTtATCttcagtctagagcagcacatgatgt

>contig00589 length=405 numreads=5

aTCCATTGAGAGTCTTGTATGGATTGATATGAGGTTATACTCAGCCGGGCTCACTGGTGA

AATTTTGGAGTGGAACTTGGCGAAATGTGAACCGCAAAATTCGTATGACTCATGTGGCGG

ACCTGTTTGGTGCTTGCGCGCAAGTCATTCGCAGTTGTTTTTGGCTGCAGGTTGTGAAGa

TGGCAGTGTCAGACTCTTTGAAaTtGTCGgTATGgAGCTGTGCTACGCAAAGCACCTagA

TAAACAGGAAAACCGaGtCcTCTCAGTTGCGTGGAGTGCaGATGaCTCTGAAATTGTGAC

TGggaGTGCtGATGGTAAGaTttGtCTATatAaTGtGcAGACaGGCCACGTGATATCGAG

AatATCGgCtGTTCGTAAAAAGtCGcGGCCGGcAATAaTTTGgTC

>contig00590 length=242 numreads=2

acgattccgccatgagctccgcccagtagggaaattCAGCTGGCGaaaaaCCAGTtGAAG

CAAGCAGAAATGGCCACTTtGCAAGGAAAGGTGAACCTGATGGACGGGAAGAGTCAATTG

CAGATGCAATGGAAACAGCTGGACGCGTTGCAGGTCAAGCACAAGGGAATTCAGGACGAA

ATCCAGGGgCAAGATAAGCGAAGTCCGTGCCCatCTGGAAGCTGCGGAGAAGACGGAAGA

GG

>contig00591 length=384 numreads=5

aaaGTTTTCGGCGGCACGTAACTTGAAGAGGGAACCTCCAATTTCaaaaacggctgaagc

actttttttctgtgcaagaaattttggcacaggcaccgactgaagcatctttttgatgct

cttgcttccgagatctttCTCGGCCAGCTTTAcTTTTTCcTCCgCAtCATCTGCACAAGc

tCTGAcAaCAGCtAAAAATTTCTCAAGTTGGTCAATCTTTCGAAATATTGGCTGTAGATC

TTTGCATCTTTCATAAAGCACAGGTAGAGTGACGAAAAGAACATCCTTTGATTCTGAGCG

AATCAaGTcAATAaCaGTACaGAACTCCTcTAATTTtGCCAGAAGCCcATCAACGATgTT

ttCAACCTCTGCAaTCTGTTTtGA

>contig00592 length=243 numreads=2

aagcagtggtatcaacgcagagtatgtggggtCTGTTGGAAGACAGACGTGTCGACcTtG

GTCCGTGTTAATTTGAAAaCCATGCAggTATTTGTGCAGTCGGACGTTTcgACAACGTAT

GACGTTAGTCCTTCGATTACCGTACAGGACTTGAAGGAGCTAATTTCATTTAGgAAaTGG

CGTTGCTGTAGAGGACCAAGTTCTTACTCTTGGGGGTcTCCTCTccACGGCGAGCTAAca

TTA

>contig00593 length=912 numreads=48

CAaTCCGAAAATGgTTTCGAAGCCGGGAGATGTGGAAATGCGTAGCTCTGAACCGCAAGC

GAGAGACCTTGgTGTGGCTGAATGGATTCTGACAATATTTTCGATATTTTTGGTCATTTG

CACTTTTCCTTTTTCACTCATGGTGGTCATGAAGATtGTGCAGGaGTAtGAGCGGGctGT

GAtATTCCGTGTtGGgTCgACtccTcTCTGgCGAGGcGAaGGgCCcTGGAaTCTTCTTCA

tCCTCCCATGCATCGACGTTtACCAGAAAGTTGACcTTCGAGTAATTtCCTTCGATGTtC

CACCaCAaGAGATACTTACTCGTGaCAGTGTCACCGTCACCGTTGACGCAGTCGTCTACT

TCCGCGTttgcAaCCCTGTCTCCTCAGTGACCAACGTCGAAGATGCGCAGAGCTCTACGA

AGCTTCTTGCGCAGTCAACACTACGCAACGAGTTAGGAACGAAAAATCTGTCCCAGCTCC

TGACCGAACGGgATCTAATCAGCAGCGAAATCCACAAGATTCTTCATCAAGCTACCGAAC

CATGGGGTATCAAGGTAGAGAGGGTGGAGATAAAGGACACTCGACTGCCACAGCAACTGC

AGCGAGCAATGGCTGCAGAAGCAGAGGCGTCAAGAGAGGCTCGTGCAAAGGTGATCGCTG

CTGAAGGAGAGAAAAACGCTGCCAAATGCTTGAAGGAAGCTGCCGAAGTCATCAGCGAGT

CACCCCAAGCCATTCAGCTTCGCTACCTGCAAACTTTGCAGACGATTTCAGCCGAAAAGA

ATTCGACGATCATATTTCCTGTTCCGaTCAACTTCATGCAGCGTCTtGCTAACaTGTAGC

GAGTGCGATcTCctCAATTTGGAACTCATTCTCGACACCATGCGTCCCGccaattttcat

gggatcgagttt

>contig00594 length=231 numreads=1

atgatgattttgtattttcatgctgcttcatgcttcttgttgttgatctgttttagaata

ccaaatgagctggtctgaggttgttttaaattatgctaatgccgcagatgcttgtcttgg

cagcatcagtaacatgccgtgggtatttcatgactgggatgctcagaataaacccagact

tgttggttatagtcccttgtgttgctattcaatctaaaatgcacaggagag

>contig00595 length=366 numreads=8

CGGAATCACATTcTACAAAGTCTATCATAAGGCTTGAGGAACTATTTTTCAGCGATCCAG

TGTATTCAAATCAaTCCCGCTCTGgTGTAGTTGGgTGGAGTAaTACTAATCTCGCTTGcT

CAAGGCGGTATATCTGGTGTTTCTAGGGGgAGATAATGATATGCTACTACAGGATGGACG

ATGGCTTCTTCCAGGCGGGTGCGTCAGTGTCCGATGTCTTGACTtAACAACGTCCCtACT

GGAAAGTCGAGAaTCAGCAGCGCAGCAGATCTTGCAGACGCTCCCGCGAGATGCACCCTT

CAACAGCAAGTCCTGGCGATAAACTCTGATAAACTCgCGATATTTTCACCAACACGTCTA

GTGttt

>contig00596 length=168 numreads=2

aGCTGAGCACTCTTGAGGCGGTAttcGCGAGAATATGTACCCAACGTCCAAGAAGAAAGA

ACaaCTTGCCGAGACCTTGGGTACTCCCTACGGGgAAGGTTGTGACCTGGTTTCAAAATC

GTCGTGCCAAGAATCGGCGTGTGCAGAAGGATAACGAGCTtAaCGAAt

>contig00597 length=189 numreads=4

AAGCAGTGGTATCAACGCAGAGTACGCGGGGAAGGAGAAACgAGACACCACGGCAGGAGC

AGAGTCACTCCAAATTGCCAAGaTATCATGGAGGACGGcGCAGtCGgATTCaacggagtc

gtggactacgtcacgttcagtgacgaggacatcgcaaacgtgaccagagaaatgcaagag

caaggggct

>contig00598 length=243 numreads=5

AaGCAGTGGTATCAACGCAGAGtCCCTTtAAAAaGTCGATCGCTTTtgTTAGGTCAACAA

AAAACAGCGTAGAGTTAGCGTTGCTGCCCTTTACATTTTTCTTGTATCTGGCCTAGTGTA

AaTGTCATATCAGCAGtaCCCCTGCCAGAGcGAAAACcACACTGgcTCTCaGgTAGGATG

CCCACTGTCTTCATGCTTCATCAGTCTGTTTAGGATGAttctggcgaggatctttcccgg

tat

>contig00599 length=1705 numreads=41

tcctttttcgactccttcagtacagaaagacgcttcaaaagaTCGTCTAGCGATTTCTCA

CCGTGCACAACGTTGTCTCTAGTtCGCACGTTCACCGTgCCcGCCTCACGCTCCTTGTCA

CcGACCACCAAAATGAAATTATACTGATCCAACTGTGCATTTCTGATCTTCTtGTTGAGG

GTTTCTCCCGCATCAACGTTGACGTCAACCTTGTACGCCTCCAACCGTCGCTTGaCCTCA

TTAGCATATtCGTCTAACTTCGGGTTGACAGGAaTAaCAATTGcTTGGCGTGGtgACAAC

CAGAAAGGCcATTTtccaCCATAACTTTcTGCCAAAACGGCGATCaTCcGTtCTaCaGAT

CCcaGAATCGCTCTGTGTATTATGACAGGCCTCTTCCTCTCATCAGCCTCTCCGCTTGTT

ACGTAGGTGAGGTTGAATCTTTCAGGCAGCTGGAAGTCGAGTTGTATGGTGGCGCATTGG

TGGTACcTACGCaAAGCGTCCCGAATTTGAATGTCGATCTTTGGACCGTAGAAcGCTCCa

TCCGCAGGATTCAGTtCCCAAGGatGACCGAATTTATTAAGAGAATTCTCTAGTTGCTTT

TCTGCCcTGTCCCACAACGAAATATCCCCCATGAACTTTtCAGGtCGAGTCGACAAACAC

AGTTtGAACGTGTAGCCAAaGaTATCaTAAACATCTCTCAAGAAGCCCAAACAGGACTCA

ATCTCTCCCTCCACCTGCTCcAACGTGCAGAAAATGTGTGCATCATCTtGCTGAAaTCTT

CGCACTCTGGtCAaCCCcGTCAAAGTCCCAGACAGCTCGTTtCGGTGCACCACGCcAAAa

TCAGCCATTCTAaGGGGAaGCTCCCTccATgAtCGTGgACgCtGAtCAAaCTCACACAGt

GACCAGgACAGTTCATCGGCTTCAGAGcGAacATTtCCTtCTCCACTtCAAAcgCGTACA

TGTTGCtAGAGTAaTGCTGCCAaTGGCCAGACGTCtgCcACaGTTTAGTGCTGTACACGT

TGGGCGtGATCACTTCCTGGAaGCcGCGCTTCCGGTATTGACTGCGCATgAACTCGATGA

GGGTGTTGAaGATGTGAGCACCGTTTGgAAGgAAGAAGCAAGAaCCAGGACTGATtTCGT

TGAAGAAAAATAGCTCTTGCTCCTtGCCAATCTTtCGGTGGTCCcTCTTGGCAGCCTCCT

CTTGGAaGTGCTTCCATTCTTTCATCTGCTTTtCGTtGGgAAATGAaaTGCCGTAAATCC

TCTGCAAAGACTCCGCATCCGCTCGGCCCTCCCAATATGtagcagagttcttggtaacag

ccatagaCTTTATTTTTCCAGTGTGCcGCACATGCGGGCCACGACAAaGGtCAATTAgCG

GGCCACACCTGTACACAGTAGTAgTCGGGGTGTCCACcTTTTCGTTGAGgATtCGgAcTT

tAAAAGGATTGTAAGCAAACATCTTCAACAGATCTTCCTTTTtCATCtCTAATCGaaCAA

AAGGCTGCTtCtCTTTTAaGACCTTCttAATAATGGTATCtAGActCCCGAaGTCACTcG

TTGAAACTTGGCGgTTATCCAGcGCcAtGTCGTAGTAGAAACCTtCCTCAATgggcggac

cgTAGCAGAGACATCCACCGTAGTGCCGCTCCATGGCTTCACCGAGGACATGGGCACTGG

AGTGCCAAAACACTTGCTGACCATC

>contig00600 length=461 numreads=20

ccacTCGCTCTCCACAGAACGATGGAGACCTCTGTTGCTCCAAAATGTtCTTGATTGGCA

TCTCCGCATAGCcGAATGGGCTCcGTCTcGAAAaCGAGTCCACCGtCCATAGGTTGAATT

TGACTTTGGTGTCATCTGAGCGCGCCTCTtCCTTGCTCAGCGAAAAGCGGAACTTCTCGT

TGAATATCGGATCTGGCCCTCTTTGGCGCGCCGACTGGTAAATTGCCGAACTGCTTGACA

GCAGGGCGCTTACTATCAGATCCTCGTTTACGTAGTCGGGTAAATGAAAGCCTTGCCGCA

AGAAAACATCAAGCACTTGAGGCTTATTGTaGTAGACAAGTTCGAATGTCAGTGTAGGTA

TGGCTGACAGTTTCCTCTGCTGCGGAGTCATATATAGTTCATCATTGTCAAAGTCGCTTA

TGTTCGATATTATactCaTTTTCTTCGTTtGTGACGCGATg

>contig00601 length=404 numreads=3

AAGCAGTGGTATCAACGCAGAGTACGCGGGTTCGCTAAGAAGCTgtCCTTGATGGCTGCA

GACGACTTCAACTGATCTATTTCCTCGCCAAACTGCAAAACAAGTTGCTGATACTCTCTG

ACCTTCTCCTCGCTGCTGCTGAGTTGCTCGGCGAGACTAGCCTTCTCAAGAGCAAGCtcG

CTGCTGCTTGCCAGCAGACTCTCATTCTCAAGCAAgagcctgaattctctgagcttagct

cttgcaactcgtcagacgcgtttcccacgtattccaagtaggcggcatccttattctcca

gggcaacccagagcaacttcagctgctcctcataattatcttgcactttcaagccagaga

gtgagcccagcttcgtatcccactctgcgttgataccactgctt

>contig00602 length=1547 numreads=61

AAGCAGTGGTATCAACGCAGAGTACGCGGGTGGTAAGGAGATGATACACAAGGAGATGAT

AAaCAaGGAGATGTAAAAaGGGGCACAGACCAGTCAACCGCAGCTATAACTCTGCATCAT

TGAATAGTTTCTAGAATTTAAATTGTGAAAATCGGTCCTAAACCTAGCACAaGATTTCAA

AGCTCAtCTTTTGGTGTCGAATCCTCGCCTTCGGCTTCGTCTtCTACCTTCGGATCAGCG

GCTTCCCCGGTCTCAGCTGACTTGTCTTCCTCGTCGTCGTCcTCATCCTCcTCTTtCTCC

TCCTTTTCATCATCTTTCTTCTTTtCGTCTTCTGCTTtCTTCTTAGCTTCCTCCTCTtCT

CGTTTCTTtCTGTCCTCTTCGTCTtGCTGGTCTTTCATTTTCTTTtCCTCAGTTTtCGTT

TTCTGGAAGGTCTCTTTGCtGAAaTCTtCAgCTtCTTTGATGTCATcAccAACAAATATA

TTGtCGAAGATAGTGCCACTCTTTACCTGCCAGATTTCGAATCCAACATGTCCTATGTTG

TCatATTTGTACAGGTTGCTGTCGGgTGAATAATCcGGATTGTCAATCTCAGGgTGAACC

caCTTTCCCTTAtATTTAGGgTTGTCGATTtGCTTTGGCTTCCATTCACCCTTGTACTCt

GGGTTATTAATCATTGGAGGTTCCCAGTCACCATCTTCAGCATCGTCCCAATCTTCTGGT

TTCTTAGCATCAGGgTCGGGGATTAACTCTGCCTTGTCCCAATCAGCAGGCTTGGTGTCC

TCAGGGTCATCAATTTTTTCCCTTTCATCCCAATCATCAGGCTTCTTTGCTTCTGGGTCC

TTGATTTTCTTTGGTGCCAGAAAGTCCCAATCATTTTCTAACTGACCAGACTCAACCTTT

TCCCCATCAATGCGAACTTCATACGTGTTATCACTGTTTAAAATCAGGGTATAAAGgTGG

gTAAACTCGTCgTCTTTGCAGCGAATATCTTTCTTAGTTAGAAGATTCTTGCCTTTGTAG

TTAAATATGACATGCACCTTCTTTGTTCCTGGGCCACAAATATCAGGACCAAACATTATG

TGGTATGGTGTGTCACCATGCATTTTACTAGGTTGGATATCACCAGGAAATAGCTTGATG

TATCCACCTCCACAATCGATATTCTGCTCGTGCTTGACCTGGTACTGAAGAACAaGCTGC

TTGCCTtcGTtGGTAAAAGgCTtGTCAATTTtGGCTGAGaTTTGGTAAAACTTCGCatct

tgggaagtttgcagtCCCtGGTCACGCGTTTTGTCACcATAAaacTTCCcAGCCGTTAGC

TTGAaCTTTCcAGAaTcACtCcTTtAtGAGTtGAaTAAaCCCATCGTTTTCCcACGAGTC

AtCTTCAAaTGTATCTTTGAAgTAAACGGTACAGCTAGCAACAGCCGCTAATCCAAGAat

AAGTAGtGTTTTCAGACCcATCTCGAAAGGTCaGATGAAAAaCACTCGTGCACTGGCTCG

AACCTCAaTCCGAaGgCCCCGCGTACTCTGCGTTGATACCACTGCTT

>contig00603 length=827 numreads=22

GTGGTAGgCAAGCTGATGGTCTTTAATCGCTTGCtGGGTAATCTCATCACGTGcTCGTTT

AAGAGCATCACTAGCCATGTCTTCAGGTGCCATGGTCGCAAATCTTTCAGGAGATATAAA

ACCATCAATTACACGCAGACGCAGCTCTGGATTCTTCTTGTCCCTCAAGTTCATGACACG

ACTTTTAATGCGGTTTCTATACTTTGGgCCAGTATCATTAAATTGCTTGTAAATAGCACC

CTCAATTtCGAAAGCAACTGACATGCAGTtAACTTTTTCtCGCTCGtcACATGTtAaGgA

ATTTAGAACCATTTCACGACATTtCTCCCTTATATCCCcATCACTACGTGATGATGGAAA

AGAAATGCCAGAGTTTCCATTGTtGgATTTCTGAGAACTTGACATGTTAGACAATGAAGA

ACTGCTGTCACTGCGACACAAACCAGatGCGTcaGGCTGCTTTtCTGCAGATCCCTGGCG

TTGAAtcttAGTTTtGTCACTCTGGCCaTCAAGAAGCTTTTTCCATGACTtGaTTAGACC

TTTTGCTAGcGACTGGATTTCTtCcTTCCCTGTAGCTTtCctCACAACGTTCACAGACAT

tCcAaTGcGtGTTTtctGCaGtATATCCAGGGTTATCGGCATATCTTTCaaCcTTTgCAa

CAAATCTCTAGCAGTACTTTTATCAGCACTAGCCTCAGAAaCCAGCTTTTCAAGCTGACG

ACCGATTTGCAGCACATCTTGCTCTAAaGGCATTCTGACAcGAGGTAATTGTGATCACTC

ACcAaGCATAAGTAGATGCCAACCAGCAAATCTTATCTGCTGCCGAA

>contig00604 length=176 numreads=2

aTTTATGAAAaGTTGGATTCTAATCATGATAGCACGCATaGCCAACTAtgataactattg

aaggtctatataactattgaaggtctctctttgatccctgcgcgtctgcgagtcttcttt

cgatgtcaactgtctacgggttcaactatgatgtaccgcaagtctaatcttcgatt

>contig00605 length=380 numreads=6

cTTTCAATGACTTCGCCATCTCGGGTTCGCCgAGGTTTTGCGAGGAAAaGGTGGAGGAAA

gAGTGAacGCAGCGGtAGGGTtgCCAtGgTGATTAGCGACATAaTTGTAAAcGGCCCGcA

GgCTGTCGTCTGCCTTGAACGTAGCAGTCAGGGACGATCCATTCTCAAGTCGGAACTGTA

TGCGACAGTCCGTGTAGTCCTtATCTTTTTtCGCCTGACCATCTACTACCACACTGgCAG

TGGCtACAgCACtcATCGGATGGCCCAAAGAGGGCCTTGGCGTAGGCTCTGGTTTGTCAA

GAGATGACTTAAACGAAGCGCGGTCTTTGGCGATCTCATCTTTAATCTTTTGTTtCAAAC

GCTTCTCTTCAAgCTTATcc

>contig00606 length=215 numreads=1

aagcagtggtatcaacgcagagtgggagagattgcgagacttgagagactgcatggtgtc

gagtgggaataacgtgcacgcattatgctttaaccggttaaaccgggtgaccagagctcg

ctcgccttggtaggcagtctgcttaggggaaggaactctgaatgtacactggggctgatg

gagcccgtaaaactgatacaaaaaatgaaaacggt

>contig00607 length=241 numreads=1

tcatgcacacggtcgtcgtccctgctcaccgggctgcggttgcctacgtccagcctgtct

cgtacggtgaggttgtagcggcttcccagacgctcctgcatttcatgtatatccgcatgc

atggtgcgctctgggtgacttgcctcatttaccatattcagtctcttgacaactggttcg

ccttcgtcaacttccatcttttgaacgattccttcccgcttaaggtcagcaaggtctacg

a

>contig00608 length=239 numreads=1

cgaaccaattcttcgtgcacgtgcaaacgccacagagggctcacgtggacccgtcctctt

taatatcaacaatattctgagaactcaatcaacattgacctgggtgagaggcgtttttat

atcgttattttatcgtctttctttccgtagtttgtgtctgtttgtgttcttttttcattt

tgctggtggattcgttttgcatttgcgttctacagttagcgacacgcctgtcaaaatta

>contig00609 length=241 numreads=1

actaggcacaagttaatgtagcagttgaccgtgccagcagggtccttgtcgcgggaaggc

aaatcaagagcttgcctgactgtaattcgaagttcggacgcttcataatgtagggagaac

ttcagtgctccggggtacgagccatcatcctggtgacttgcattctggtcataactaatc

acaggatcattgcttgcaatgtcttgtatatcaggaagaaacaaaccgtggagatacacg

t

>contig00610 length=241 numreads=2

tactggctagaacatcaaggcgaccagccgacatacaaaacagccgatatttcaggcctc

gtgcaggaaagcgctattgccaaatccacagcaaagcaaaaagcctgacaacttttctcg

cattcattccttcacggcgtaaacaagtaagactaggatccgagatgccaaaaaGAGCGT

GAGCTGGAGGCACACATGCAATTTATCACAGGCTAAAGTTTCAGTTTCTTCTCTGCAGGC

G

>contig00611 length=240 numreads=1

aagcagtggtatcaacgcagagtacgcggggacggataagtctcttgtttattatctttt

gagagactaagccgtaagcatacagttttctcaagatgacgatatttaacatgtatatat

tcgacagaaatggggcatgtctctattacatggaatggcaaagacggaaaccttgtacga

tgttgaaggaggaggaacagaagcttatgtatggcatgctttttccctaagtcgtttgtt

>contig00612 length=223 numreads=4

aGCCGAATCAGCATTCTGTCGAGCcAGCGCAGAACATCCTGGGAGCTATCCTCCGTGTCG

CACCGATGAACCGCAACGCGCGCCATGAGAACAGCGCTGgcTTCTTGATcAAAACTaGCT

GCAATGTGGTGCAaGTTGGCCGAGTCTACCCAGTTGCGAGCACACGTTGTGACACGGACG

CGGCGCtGACCACTCGAGTGTTGGTACTGaGTGATGAACTGTA

>contig00613 length=241 numreads=3

AaGTCCCcTATTTTTAGCtCAtAAaCGTTTACCAAAtAGTCAaTcAACTtgTGGgCcGAT

CCGTtAaTAAGATTCcGCcTtCCGGTATTTTATCTGTCGCTTGTGTAAGCTTCCCTGCCC

AGTTACCTTGGCATCACGCAAACGCGATTTGCTGAACCAAGTTACCGAATCTAAGAAGGC

GCTGCTTTCACCTGCCGTAATCGAAGTCGAAGTAAGTTCCACTTGAGATggacaagcacc

g

>contig00614 length=207 numreads=1

taggcggaagtcgtaccgcaaggcatttgaacgtcacgaacgttgcggatctgctctcgg

acagtgttagttctgatcgaggtggtccactgaaggtgtctcgcataattgaaccgttac

atgctgatgatagtcctgctctatatcagagcaatttcgattctgacagcagtccagagt

cagcagtacagcctagtcatagcatca

>contig00615 length=340 numreads=5

TGTTTGCcgAAGAACTCTGGTTAAAaGCgcGCGtACTTTGATCTGTAGCGGAACGTACTC

GATCGGCAAaGAGCGgcTTTTTATAGCTaTAGCAAAaGAGTTtGGCTTAAaGGTTGGAGT

CTCTGCGTCGAAGTTGAAAATtCTTTCGTgCTTGgAaGACGAGgAaCTGAACAAATTGAT

GACGCGGGACTTgCGGGCATCGCAAATTCACGtCCTCCCTATGGCAGCTCTGTcTTTCAA

GGCTCTGAATGAGCATCTGTCCGTCAAATGCAAATCTTCGGGATACGAGCACTTGCTGGC

TTTCAAGCCGACAGGCTGGACTCACTCGGCTAAAGTACCa

>contig00616 length=397 numreads=3

AAGCAGTGGTATCAACGCAGAGTACGCGGGGCACTAAGTCAAACGACAAGCTCTTGTGTT

CTAGCGACAAAGTAGCAGTAGCAGCAGCTACCTCAAACAGGCAATTAATAGTCAACTTAC

AGTCCCAGCTTGGAGTGTGTGACAAGACACCTCAACAAATGAAaaCGTGCTttGaCGAaa

GgCGAaGAGACAGGCTtCGTGcTGAaTACAATGGTGTTGCAcaTCTAACCATGGCAGACT

Cagtcctacctcgaaaatggctatgggatggacgttttgctagcagtcagatggttaccg

gaataaaaatcttgtctggtaccctacccacgcgccttcggacatccagggtgtagccga

acgtcacttgaagctatgccgccatgggtgccaggca

>contig00617 length=436 numreads=27

TTTTTTTTTTTtttgTACATCAGGAGAACAAGATATTTCTTTtCCAAGATGGCGCCAGCT

ACAGAGGACCCAGAGGACGACGGCATGAGCTTCATCGAGTTATtGCGGTTCTCCGATCAA

CTGCTGAGGCGCTACCGCcAGCTCtGCTTTGCCTCCTTtCTGCTCAGaCGACGGAGCCTg

TGGAATGAcctCttCTTTAGGCTCGACTATGGAAACATGGTCCGgAAGTGGCTTCTtGGG

TCCGgtCTTGCcAGTAGGATCCCaGGGAAGCATGATCTTGACCTTTATACCAAGCACACC

TTGTCTTAAATATACATGGCGGACAGCCATATCAATGTATTCCTGGATTGGGTAaCCACT

GTGAATCATAAAACCATCAGTGAACTTCATGGACTTTGCTCGCTGGCCTCGAAGTTTTCC

GGACACAACAACTTCA

>contig00618 length=1678 numreads=50

GATGATGTGGGTGGGTGCAGAAAGCAACTAGCCCAAATTAaGgAAAtGGTaGAACTGCCA

CTGcGCcAtCcTCAaCTTTTTAAGGctcTaGGtATCAAGCCTCCTCGAGGAAttttGCTC

TATGGGCCACCTGGTACAGGAAAGACATTaaTtgtAAGAGCCgTtGcAAATGagaCGGGG

gCaTtCTTTTtCtAaTCAATGGTCCTGAAATAaTGAGCAAGTTAGCTGGCGAgTCGGAAA

GCAATCTGAGAAAAGCTTTCGAGGAAgCCGAAaaGAATTcGCCcgCGAtAaTTTtCAtCG

AtGAAaTCGAcGCGATTGCGCCAAAACGCGAGAAAACGCACGGGGAAGTAGAGCGACGAA

TCGTATCTCAGCTTCTCACGCTCATGGATGGTCTGAAGCAAAGAGCTCATGTCATCGTTA

TGGCAGCCACAAaCCGCCCAAATAGCATCGACCCGGCATtAAGGCGTTTtGGCCGCTTCG

ACAGAGAAGTAGACATTGGAaTTCCTGATGCtgTGGGAAGACTCGAAaTtCTGCGCATTC

ACACAAaGAATATGAAGCTTGACGATAACGTTGATTtGGAGCAAGTTgCtGCcgAGACTC

ATGgTTACGTTGGCTCAGATGTTGCTTCGCTTtGCTCTGAAGcTGCCCTtCAgcAGATTC

GCgAAAAAATGGATCTGATCGAtcTtGaGgAAGATACGATTGATGCCGAGGTTCTAGACT

CTCTAGCCGTAACTATggACAATTTCAGGTTtGCCATGGgAGCAaCtAaTCcGTCTGcTt

taCGaGAaaCTatCGTTGAAGTtCCAAAtgTCACATGGGAagACATAGGtGGTTTGgAAa

aTGTTAAGcGAGAGTTgCAAGAGTTAGTgCAGTACCCGGTCGAACATCCAGAAAAGTTCT

TGAAGTTTGGAATGACTCCGTCAAAAGGCGTCCTTTtctATGGTCCTCCTGGTTGCGGTA

AAACTCTTCTTGCTAAAGCAATTGCAAATGAATGCCAGGCCAACTTCATATCGATAAAAG

GGCCTGAGCTGCTGACGATGTGGTTTGGTGAATCCGAAGCGAATGTTAGAGACGTGTTTG

ACAAGGCTCGCATGGCCGCCCCCTGTGTCTtGTTTTTTGATGAGTTGGACTCAATCGCtA

aGTCTCGTGgTGgCAGTGTTGGTGACGGAGGCGGTGCTGCAGATCGCGTAATCAATCAAA

TTCTGACTGAGATGGATGGAATGGGTGCCAAGAAGAACGTCTTTATCATTGGCGCTACTA

AcaGGCCagATATAATCGATCCAGCTATtcTTCGACCcGGTcGACtGGATCAGCTGaTAT

ACATCCCTTTGccTGATGATGGAtCTCGACTAGCCAtCTTAAAagCTGCTCTgCGAAAAa

CTCcTATTtCGAaGgATGTCGACTtAAACTATCTCAGTAGTGTGACTAAGGGCTTCAGTG

gAGCAGACTTGACGGAAATCTGCcAACGGGCTTGCAaGCTGGCCATCAGAGAATCCATTG

AGAAGGAAATCCGGCGGGAaCGCGAAAAGGAaGCCAACCCTGATGCTGATATGGAGATCG

CAGaCGAGGAGGATCCTGTTCCAGAAGTACGACGGGATCACTTCGAGGAGgCCATGCGAT

TTGCTCgACGTTCAGTATCtgACAATGATAGCaaGaaGTACGAGaTGTTTgcTcAGac

>contig00619 length=1338 numreads=192

TgTtcAAGAATAACTCATGTATTTTAATGTGGgCCGCAATGGCCTCTCTTAATGACATTC

CTAATTGACAGATTTCTACGGAAGGTACAGCTACTTCCTACCGCTCGTCGGACTAGGAAA

GCGCATCGCATTGGCGATACTTCCCCTTTCCGCAAATAGCCAAGCAGCAGGCTAAATACG

ATACGTGCCAAGACACTTAGACGAAACCAGACCTTCAATGACAACAATGATCTACATGTG

TGCCTTCAGAGACGTGGGCAACATCTTtCTTCTTCCCCTCACAGGCCTGGAAGACTTCTT

GCTGTTCTTCCGCAGTTGTGGCTTCCGCATGCTGCTTCAGCAGCTTGTCGCCTGCAGTCA

CTCCCCACTGGTCAGGCTTCGATAGGGCAGGGCAGAACGGACGACCGCTTGCTGGCCGAA

TGTTTtCCCAGTAATCTCGTCTAGCGCGAGCACGAACCCAAGGTTTtGTATGCAGCTGAA

GGCCAGCGCCCCACAAATCGTGCTTCTCGGTGCCGGCAGGTTTCTTCCCTCGCTCTTGAC

AAAGCTCTTCAGAAATCGCTCGGATGTGATACGGCTCAAAGCCACAACAGCCACCAATGT

ATCGGATTCCCATGTTGTACGCTTCCCGCGCATATGCCTGTATATCCCATCGAGTGCAGA

TGCGAGGCTCGAGAGCAAAGGGAAATTCAGGAAGGTCAATGAAACCTTGCAGACCAGCAT

CAGGAGTTTGGAAGCCAAGGGGCTGAATAATCATGTGCTTCTTTATCCCGGCAGCCTCGA

GTCCTTCTTTCATTTTTCTCATTCCAGCAAGGGCGCTGAATGGCCCGAAGTGGCAGTTGA

TACCCACAACATCAGCACCAGCTTTGGCCATACGCACAGCACACTCACCAGTGGAGACAC

CATGCATGTCGCCCTCGTGACCAATGCACATGGTTGAACAGACTGGCAAGCCTCCTTCTT

TCAAAACTTCAATAGCCCATTCCATTTCTTCTATATGCTCAAAATACTCTGCAATAAGAA

AGTCAACACCCATCTCAATGAAGCACTTCAGCTGCTTACGAAACTGCTCCTGCACTACAA

CTTTGCCAGATCCTGACAAGTACGCTGGGGTCTGTGAAACGCCACCTGCAACCAACGCAT

CTCCTTCGTTTGCAACTTCACGTGCAATAGTGCAGGCTGCCTTGTTGATTTCAAAGaCTC

CAAACTTTCTTGCTGCTTCATTGCCTCGGTTgTCTAGTTtATCTTCACTGgCATaGAAAG

TGAAGGTTtGCATCACATCAGAGCCAGcACGTaGAAACTCACGATGCAGCTGTTTCACTG

CaTATGGgTgCTCCACAA

>contig00620 length=242 numreads=4

gCTCTAACTCTCTCAAAGAAAACACAGTCGCAACGTTCGGTAGTGTTTCTAACAGtGGAG

CTTGCGGAAGTGGACTCGATTTCCTGCATGCAGTtATTTCTGTGAAATCCTGGTCACACT

ATCTGGAGgTTAGTCGTGACTCGCATTACTCGGAAGTGTTTATTAGtGAGCTTCAGGACT

CCCTTCGATCTGTGAGCAAGAGTTTGCTTGTCGTAGATTTGAAATtCTTcgagcttagat

tt

>contig00621 length=188 numreads=1

ccgctaaaaaactcgttgcctgtagaaagcaattaaaaccgctattatttataagtttat

ggaaaggaagcgtttctttcacatcttttcttgagaaaagtgcatgaattgcgatgtctg

tgacttcgacgaacatgcgatagagttctgttcttgtggcaaggtccattaggccgtaca

tcaaggtg

>contig00622 length=241 numreads=1

agttcaacgagaacgaggaggtgcgcaatcgcgccgtctttcttatggagacgtattacg

agaacgacgacttagagatgaaggttggtgagcatgacgaggtcgcggtgaatgacgata

gagaggtccagcgtagtggcgaagagaaaccgccggagcgttagcgaagggttgtggaga

taaatgaaattgcggcgggctctgagtgagcttgttgtaaaacgttttcggagcgaggcg

g

>contig00623 length=240 numreads=2

aaGTGGCCTTAATTCTTGGTCCGCTGATGGTGTCGATTTATTAGGTGTGTGTGACACTAA

AGAGGATACATCTGTCACGAAAACGGACATGCAACCTCCATCTGTAGATGCGACGTCGGC

ATCTGACGCTTTGAAGTTTACTGCTGCTGATCCTTTTGCCGGAGGAATGTTAGCTGtcca

gcatttgcatgtgttcaccgagagctgtcctgaccgacaggtaccttcattcggtcagaa

>contig00624 length=353 numreads=6

ccATAAAATAATTCTTCCTTAACCTTGGAGTGAGACGAGTTTGAAACTTTTGCAGCTGAA

ACCAGCGACGACATGCCACGGACTTTTACTAGTGACATAAGACCGGCGCTCATAAAaCGC

CATTTCAATAtACGTAACTGCTAaCGGAaCAAgCGGgCAGGCATATACTtCcAGTTCACC

TAAGGAAaCCCATCTTCGGAGCGTCGGGTGAGGTTTCAGCCCTTtGCAGCGTCTTCGACA

GCTTAGCaTTAaGgTCCTTGCCGATTTGGCGCCCGACCtCcAGCaTCTCAAACATGAGCT

CTGGATTGATTCCaCCGTGACCGTGCTTCTGaaCACTGCAAATCTTtCCCGTc

>contig00625 length=218 numreads=5

AAGCAGTGGTATCAaCGCAGAGTtACTTTTTTTTTTTTTTTTTtCTATCTGCAAaTTGCC

TtCACAAAaTAATCACTCAATCGCACCACTAAATGAGCTAGTGCATACaGCTAAAATTGC

AGgAATCAGTGACAAAAAGGCTATAGTAAAAAGtCATCCATCGTCTGTGAGCCAGAGCGT

AATACCAGAACaGGcccAGGTTACTCCCTTGCTCGTCa

>contig00626 length=224 numreads=2

AAGCAGTGGTATCAACGCAGAGTACgCggggACCTGACTGAGCTCCGACGAGGAAGGTCG

CTTGCTCTGGATGCTGCATCTCTCTTTTGCAATTATCAAGAAATCTGAtCATGGCTACGG

CACCTTGTGCTCGATGTGACAAGCCTGTTTATCCGACGGAAAAGCTAAGTTGTCTGGATA

AAACCTGGCACAAAGGTTGCTTCAGCTGCGAAACATGCAGCTTg

>contig00627 length=437 numreads=15

caCAGGAATGTAGTTCGaCTTAAAGAAgTGGTTGTtGGACGATTGTTGCAGACTGTGTTT

CtGGTTATGGAGTACTGTgAGCAGGATTTAGCTAGTTTGCTGGACAaTaTGACAACTCCA

TTTACGGAAGCACAGgTGAAATGCTTGGTGCTACAATTATTGTCTGgTGTATCGTATCTT

CACAAAAaCTTTGTTATTCATCGGGACTTGAAGCTTTCCAATCTGCTGTTGAGGCATAAT

GGGGATTTGAAAATAGCTGACTTTGGACTTGCGCGCACTTGCGGTGTTCCTGCGAAACCC

ATGACACCCGTTGTGGTGACCTTATGGTATCGGGCGCCGGAGTTGCTTCTtGGATCTCGG

ACTCACTCTGCTGCCgttgatatGTGGGCTGTTGGATGCATtCTTGgCGAGCTGcctctg

aacagcctttgtgaacc

>contig00628 length=865 numreads=26

aagcagtggtatcaacgcagagtacgcGGgggAGATGTTGTAAACGGGGGTTGGACAGAT

TTCAGTGCCTGGTCAACATGCTCACAGACATGTAATGGTGGCACTCAAACgAGAGCCCgA

TCGTGTACCAACCCTTCACCAAATCAgTTTGgTTACCCgTGCAAaGGggTagCCGTGGAG

TCAAGAACTTGCAACgAGTGGATtATTTgCGCAGACCCCACCTCGACttttcccggttgc

aagtttattcttggcgacggcagtggAGGAAGCGAAaTAAACATAGGTAcAGTTGCCAAC

CCGAAGGAATGCGTTGAAGCTtGTTTTCAAAGACACCAAACAGATCTGAACATAAATGGT

GTAACAGTTGATGCGgCTACTGGAACAAACTGCTACTGTGAAAAGGGCTTGAATGGATCT

AACTCCTCACCTGCATGGATGaCCTGCAAGTtCGGAGACaTTATtAACGGAGGTTGGACA

GAGTTCAGCGAATGGTCAACATGTTCACAGTCGTGTAATGGTGGAACGCAGTCGAGAACA

CGATCAtGTACTAACCCGTCACcAAATCAGTTCGgCACGTCATGCATTGgCGAAGCAaTG

gAGtCAAGGACTTGTAaCcAGTGGATTATTTGtGCAGATCGTTCTACATCATTTTCCGGA

TGCGAGCTTGTCGTTGGTGATGGAATTGGAGGAAGCGAAATTAACATAGGCACATTTACC

AATCCAAGCGAGTGCGTGGCAACTTGCTACAaGAGGCGTGGGACAGATCCTGATATCAAC

GGTGTGACTGTTGATGCCGCTACTGGTAAATCcTGCTATTGTGAGAAGGGAATGACCGGG

TCGAATtCGTCACCACATGGAGgAC

>contig00629 length=237 numreads=1

aagcagtggtatcaacgcagagtacgcgggggagacatagatcgtctatatactgctaga

aaggagggtggaagaggcctgaagagcgttcttgatgtgtatttagcgacactggtatct

ctggcgagtcatcttgaaaaggctgcaaataacaaacttcctcttagaaatggtaaaaca

gcacgagaaagaacgcctgatgcgtgcatcacacttgcttcagaatgcactgcaaat

>contig00630 length=157 numreads=2

TCTTCTTTATTGCTTTACCCTGCTTGGAGGTTgAAGAAGCATGTGATCTCTGCTTTGGTA

TGGGAAAAGATTGATTGCTGGGgTGCCGTTTACTGCTCTCCATTgacaaggctctctgct

gcatccacttgccttgttgttctttattattaacctt

>contig00631 length=187 numreads=2

AAGCAGTGGTATCAACGCAGAGTTACttttttttttttttttttAattGgTACAGTCATG

ATCTTCAGTGTCTATGCTTAaCAAAAGGAGCTAaCAAATAGCTTCggCCGTAAAACAAAA

aGCACCGGTTGATCATTCACGCAGCCAGAAAGCACAATCCCAATATtGCACCAAATGTTG

CAGTTaa

>contig00632 length=562 numreads=10

GAAGGgCtGGAAATGGCGCGTGCACTCGTCAaCCcAGCcATACTCCCAGGATCTGAACCA

ACtACAGGACTGGcAGGGTTTCcGTTGTCCCcGCCTCCTTGaCacGCGcTAACCAgTGCC

TTGATCTCGTCCCCCTTCAGAGCGTAGTTGTAGATGCGAAaCTCGTCTATCGATCCGCGC

AaGGGtCGATGCTTCTCGTGGTTACCgATACcTGCACGAATGCCCCAGTCACGTGACAGt

GTTCCCTCGCCAATtGACTGATTCGCCAGCCTGCCATTGATaaaaaGCttcgccttgcca

agtttggAGTCATAAGACCCAGCAATATGTGTCCATTtGTTTTTTggAATAAGATGAGCC

ATAGtctcgaaaatgacTTTTTGCTTGCAATCTCTGTGGAACCAGCGAACAACcCCGTCG

TTAACCTCGAAGTGGtACTGTCCTTGCGAGTGGGATGTTCCGATCGTATCGAAGATAGAG

TGCTGACCCTGCTTTTCCTCCAGCTTTACCCAAGCTGCGATTGTGATTGCTCGGCGTGGc

TttgCTTGAAAGgTTtCtCCAA

>contig00633 length=349 numreads=8

ttCTTTGCCCTGCTCTATCTGAGTGCACTTGATTAGCTCTAaCGAAAAACTTCATCACGG

ACATGTTAGCTTCACGGTGATCAAGCGCAGTtGCTGCTGTTGCAAGCTGAACTATCGAAT

CCATGGCTTCGTTTTGTAGGAAGGGCAAAGTAACCTTTTGTAAAAATCTGATACAAAGTC

TGAAAaaGTCATCGACAGTGTCTGGATGGTTTATCAGACCACGCTCTtCTGACAACATCT

GAAAAGTCGGaGTGGTGAATGCCCGCAGCATTTGCATGAGAGATTCCTGAACaTGCTtGA

TCTTCCCATACTCATCTGCCAGAATACTTCCCAAGTACAGAAAGCACGa

>contig00634 length=242 numreads=1

aagcagtggtatcaacgcagagtacgtcggggagtcgaggaaggaatttttgatgcgata

aagggagggcgcgtgctttacgtaactgtattagctgtactctgagtcagaggattgctt

atagactacgacatttggggctttgtaacagtgttagagcacgggaagccgttgtctgtc

cgagtcggcgaattctggcgagttggttgcgtggagtaagttacaggtagcggtgtcata

ac

>contig00635 length=238 numreads=4

TTCATCTTCTATATGgACGAACcAGGAtCcTTTAAAGGCAACTCGGATGATgTCaCTTTT

AAAGAGCTACCTGCGGACCCTGAGAGGgAGCGCTTAACATCTAtCTGATTCAACCTTGCG

AATTTGATCTGATATAAGTAGAGTATGAGATTTTGAGGAATATTCGCAACATGGACTCAa

GGAGGCATtGCTCCTATATTTGttAGAGGGACTGCGTTATCAGCAAGCCGCACGGATa

>contig00636 length=219 numreads=2

aagcagtggtatcaacgcagagtgcgcggggacggataagtctcttgtttattatctttt

gagagacTAAGACGCATGGTTTTAGACGATGAGTCGGAGTTTGGCGATTGCGATTGCGTC

CGGCATTCGTCGACTGTCGTTCAATCCACAAGGACCGCGCTCCATTTCCACGTCGGCATG

CTTGTGCAAGCGGCGCTCGCCTAGCTCTGTAGGTGTCAA

>contig00637 length=670 numreads=23

TTtGCTGGATGATCGATcTCGGACGATATTTGCCGTGTATCTGAATGCACAAGGGCGCTG

TCTGTACGATGCGTtGATCTATCGTCTGTCGGAGAATTTGGgTGACTATTTtGTGGAAGT

TTCAAGTGAGCATGCAAAGGATGCCTTGAGTCATATGCGGCGCTATAAACTGCGATCGAA

AGTGGAGTTTGAGGATTTGGCTGACACTTACAACGTATATGGCGCCTTTGTTACCTCGGC

TAGCACTTTCGGATCGTCGCCcATTCTAAACAACACCTGTCTTCTTCAGATGCAGAATGC

GGTACTGGATCCGAGGTTGCCGATGCTTGGCTCGCGTATCTTATCAATGGGGgCGTTGCC

TCCCCCTGACGgCTTTGAAGTTGTCAGCATTGACACTTACCGAAGATTTCGATATGAGCT

TGGCATTTGCGAAGGACAGGAAGAGTACGAGAATGGGATGCCGTTGGAGTATAACATGGC

TTTACTTAATGGAGTGAGTTTTGACAAGGGCTGCTATATTGGTCAAGAACTGATCGCAAG

AGCTCATCATAcTGGTgTTATTCGGAAACGCGTCaTGCCGATTACTTTTACGCAAGTAaT

GGGaGCTCGGATGCCAcTGTtGCCGCcGgAGCAGCaatctggtcgccttccaaaaagaag

gaatcgggaa

>contig00638 length=231 numreads=3

aTATGTCTCCAACATTTCGCAACCAACATAGATCTCCCATTTCCTCCTTCATAAGCTTCG

TCAAAGAAACCAGAACTTCTcTCATGGACTTGTTcTtGAGaTCCAGCTGCTCcTcGTATG

GCATTTGAAACCATGGGCAGGTAGAATGAGgAACTCTCTCAGAGAAAGGCAACTGCTCTA

ACttGCTCTCTTCTCATTCTCCTCTCCcAaTGACTGCTATTCTTGCGTTTG

>contig00639 length=131 numreads=1

ttttttgccttctccttgcacatctgaatgcagggcgtcatgaaacgcctcgcgtcctca

gctccttcgaccgcctctttatactttaacaatacgagctccttaatttcttgcggataa

agcgggccaat

>contig00640 length=436 numreads=5

aGTATCGGCCTTTGGTAATTGTCCCAAAACCGCGAGGGAATGACTCCGACAGGTACGTCG

TGTGTTAACGCCCGCACATGGCTTGCAaGGgAAGGCAgCgtAATCTCGAAGGTAAGgCAG

GGAGTTTGCCTCTTTGTtAaCTTGATCTTGATGGCCTGTgCGGCTTGGCCACTTTTCATA

GCCCTAAATAGATTTTCATTGATGACTtCcAAaTAAaTCTCGTTCTTCtCATCcTTgCCT

TCTATACGATaCTcATCGAAGAAGTCAGcctGGTTAATCTCGCACCACAAACTACATCCG

CTACTGACAACCCGGTCGCTCAGCGTAAAAATGATTTGCGTCGGAGTAAATCTGAGTGCG

CAAGTCTTAGACACTTTTGCAATCGTCGACAAAATCTTTGTAAACTGGTGAATGCAAACA

ATATCAAGGAGCTTCg

>contig00641 length=538 numreads=7

gTTTCTGAAAAaaGgAAaaGAAGAAAAaaCTTGTGGTTCCTTTCACCAATGCATGAGCCC

ATCCaTGGACAATGATGATCATATCGgCGGACACAGCGACCGCAaTCTTCgCAGTGTTTG

gAGCGTAGCGGCTGCATAATGTTACAATATCCACAGTGACGAagTCTATAATcTCTGGCA

GATGCTGGTTGCgCtAACAATAAGGTATCCTCAGAaGTAGCATCATTATTttGACTATAA

TCACAGGTGGAAATATAAGCAAGCtCCaaaGtcGAaTCCTCATCTTGTCCAAATTGGTTG

TAAGGCACATAACCAGGATTGAGAAAACTTGTTACAAAGTAGAACATCAACGTTATGAAC

AACAGCACaaaaataTACACGCATACCATCTAGGTCCTTTGTACAAACTTTCCTTGAGCT

GAGTATCTTTaaAAAAaGAaCAAAAaCAAaTCCAAGAGtcagagcgacatgtaagatgtg

agtggcccatttttctagagactttgcccttaaatgcatcttgggtaccaagcccaag

>contig00642 length=241 numreads=1

gacaaaagtaacgcttttaaatgtaacattcttggcatccaatttttaaaaagcgttcaa

gcgttaaacgtttaattctttccaaattaaaccctttaatcgttaagtcccctatatctg

aagagaactaacggacctgtgttcgctagagaaaaactgtagcggacttaacggactggg

ttgccaactatggtctgaagcaattctctggtttccgcaggtccgtttaaggtgcagctc

c

>contig00643 length=443 numreads=10

CAaCATGGgATTAaGCAAAGAaCTGTATTTTGTCAAGCTACcAAACTTtCTTAGTGtGgA

GACAAaGCcGTtCGACCCCAATTtCTACGAAGATGAAATCGATGAAGACGAGGTTCTTGA

CGAAGAAGGAAGGGCCAgACTGAAATTAAGAGTTGAAAATACCATtCGgTGGcGTTACAC

TAAGGACAAGgATGGCAaTGAAGTAAAAGAAAGCAATGCTCGCGTCGTAAAATGGTCCGA

CGGAAGTATGTCCCTTCATTTGGGAAACGAGATCTTTGATATATTTAAaATGCCGATCCA

GGGAAAGCAAAATCATTTGTTTgTGCAGCAGGgTaCAGgTTTaCAGgCTcAAGCAGTTtt

tCGAAaCAaGTTGTCTTtCcGTCcGCaCTCGACCGCGaGTCAGActcACcGtAAaaTGAC

caTGtCAATAGCTGAGCGCATGt

>contig00644 length=173 numreads=2

tgctgcctcctcgggagaaatttGTTGTCGCGGACCTATCGGAAGAGGTGCACCTTCAAC

CCGCCCCTACcACGCCGGCAAAGCAGCAGTGGTCAGTACGACAAGAAGGGTGGTTACTAC

GACGAAGATGCTGATCATCGTCATGGCGGACAAGGTGTGCAGTGCCAAACATC

>contig00645 length=239 numreads=1

tcacgcgcttgccatcgtctacccgtgatttgccatcaaccttatctgttcttcgtaagc

gcataaacttacgctgacatattggctggtttttattttattttcattgttttctgcatg

agcaaacgttgcacgattttcattgatgtcttccttgggctgggttggtggacgggatgc

caagcagcagtcgggctggtaggctcgctgctgatagacgggtagtaaggttgcgggta

>contig00646 length=200 numreads=2

ttAATCTTGATGGACAAGTGATTGGAATCAACACTATGACCTTGCTTAACACGACTGGGA

TTTCGTTTGCTGTGCCCATCGACCGCGccAAGGCATTTCTGCTACAAGCCTTAGAGAAGC

AAGACCTTCGGAAATCTGGAAAACGAGTCCCGACGCtGGgTCCTCGGCCCTATATCGGCT

TAAAAATGATGACCCTTGTg

>contig00647 length=230 numreads=1

aagcagtggtatcaacgcagagttactttttttttttttttttttttttttagaagccag

gcttgctacaacatgcatattgtaccatacatctcacaggacgtttatacgcatacttca

gcatgccaagacatctgactttaagaatggacatcgtcgcctcgctgctattggattcgc

aatacactgtctagcgtggctctgcggtaaccagtggcactgcgcgtaat

>contig00648 length=241 numreads=1

ttttcaagtagcttcgaaacaatgtcttcaacacgaataaaccaagatggcacagccttg

taaattagtggtgtgtctgacctccaacaaaatgggtaactatgcttgattgtactctgg

tgaactagcctaccactttctttcagacgacgaataatcagcttgtctgcatctttcaca

tatattcccttaaattccaaaacatcttctgtgaatgccccagctgcgtcaacagggcaa

g

>contig00649 length=241 numreads=1

cctgcacttcttcccttccattctccgtacttagcttcccttcgttgtacgttttgtgga

tcgtaaatacggacgtattcactagcattacatacagtcacattcgtgaagtgcttgcaa

aaatcgtcaaacgacatccaaaactcgccgtcatccttcacctcaatcccaagcttggaa

agttggctcttgctgatcttcttccattcggcagaattgtcactccatgcaccaatccac

t

>contig00650 length=776 numreads=14

aaTAGAGGTCTTCTTCTTTCTCTTTCCaTTTCGATTCCTGTAGTCTAAAtactccaacat

gtcattatccacaatggctttcGCCAAATCAGGCGCGTCGGAGACGGAGGACTTGCtGGA

TGgAGTCCcTCCcATtCTGAATCTGGCCAGAAGCGTGCTTTCCTtCATGCTTGCGTCATT

GATCGACCcAGTGCTGCTCAAACGAGGCTTTCTGGATCGGTCATCTTTGAACGTCAGAGC

TCGGGACAATCTTCGTTCTTTCAGGgAAATTCGGTTCCGCTCCGACGGTTGTCGCCGCaG

GgTCGGCGTTTTATTTTCCGTAAACAAACCATCGACTCCGAGGTACGGCCGAATAACTCG

GGCTACTAGCTTTTCAAGACGCAGAAAGTTTTCGCTAGCTGGAATTGCGTCTTGAACACT

GGAAAGAACTAACAACATTtGAGAAaTATCCGAGgACACTTTCACGCCCGgTTCCAGGgC

GTCcTCGATTTtGCTTTTTAGAAgAATAaTAtCTCGAAATCCAAcAAGCGtCaCAGAGcG

aaTGTAAATCCCTTGCTCTTGTATAGGATaaaaaatagcgagcagagtcggcaaaatttc

cctataaaactgcttccatacatcgcccaatctctctaataagtcgggtcctttcctttc

tttgatagcttctcgtagaacaatcaTACCCTTCTTCAAAATAGAGTCCTTGTAATACTC

AACAATAAACTGACCAATCTTCGAATCCCTGACGttgCgAACTTTTTCGTtCAACg

>contig00651 length=184 numreads=1

ttcggtcgctttgggacttgtacctggtaccagagagattccctggctcaagtggaattc

ctgcaaagtggatgtcccccagccttccgtcgagcaaggaatggggcgattatttggtgc

gaccgaaatcctccaaaaagaaaaagccgctaggagagttgtcttcggtgtaaattattc

tttc

>contig00652 length=119 numreads=1

aagtcagcaaggacgcagcctgggctagcagccttaaaagcctccatatcagacagaagg

gatgcgctctgcatctgcgcccgtactctagcagcttcttcagatgtgccgagttctac

>contig00653 length=242 numreads=4

TGAACTCGCTTCAGCAAGGCTTTGCtGACACCGGCCTCGGAGAAGGGCGTCTCCTAGGTG

GGTAGTGGTTGTCGGACCGTTCGATCAAGGTATCCTTTTTTACCACATGGAGAAGTTTTT

TGGTtGATCACGCCGATGTGAATGAATTAAGGAGAGTGATTGATTCTGTAGGAAATGCTT

AAGaTAGTACAAAGGATTTCAACCGTACTTGATATTTGCCgggcggcgattttgttgtat

ta

>contig00654 length=255 numreads=9

AAGCAGTGGTATCAACGTCAGAGTACGCGGGGGACCACGTGCGAATCGAGCTACGCGTAC

TACAGCTGACACGCGACATGGAAATGTCGCATGATCAACAAACAGTAAgTCGCTTCTATA

ATATAACAATCATTTATTCACTGCTGAAaTAAAAaCAaGCTtCCTCCAaTGGAAGAATGC

CATTAATATGTAGTACTTATAAACAAAATTcGTtAATaTCCGCACCGCGCTATTCCCCGA

GTTGTTTGAGAcaag

>contig00655 length=259 numreads=3

AAGCAGtGgTATCAACGcAgAGtACGcGGgCaCATACGGggacGCGGATCTTTATcTCcA

GACAGAGAtAATGCACACCTTTtGTAGGGGTCGTTCTTATtACTACTGGTAGTTATAATT

ATCGGAAAACGGAGGACATCGCAAGAAGAAGAAAGGCCGGTCTAGCTGGCAACTCCGATT

TTCACTACACCTGCTATTtCGCAGAGAGTGCTGAGAAAAATCATGATTTCCACATTACTT

ACCCTGCTACCACTACGCa

>contig00656 length=241 numreads=2

AAGCAGTGGTATCAACGCAGAGTACGCGGGGACGGATAAGTCTCTTGTTTATTATCTTTT

GAgggACTAAGCAAAACTGCCCTCCTACTGGGTGTCACTTTTATGTCACGAAGctactag

actgtggatttagtgtcatctaaagactgtttcaaagatgtctggcacctcaggacttca

acatgacgttagggcagatgctactggttcagcagaccagaactcggaatctgtccatgc

a

>contig00657 length=391 numreads=7

aTGAGTTTGCTAGAAGAATCATCGTGCTTTCGGTGATGGACAAAAGCgggTTGGGTAACG

ACAAGCGGATGGGAGACGTTCACATAccgctatccGGTTTaaaCTTAAGCggCCATGGTG

CTCGCAGGACATCCTACGATTTGAAAGATCTGCGCAACACGCTGCAAACTAGAAGCAAGT

GGTCCACAGAGGGTCTGGCCGTGGAGTtCcGaGAAGCCATGCTCGCCcAcGCCATATATG

TCTGTCCGAaGTTTTTGTTtAAgAAGCAGCaTCGTGGtAGCATGCTTGTTTCTcTGCACA

GCACGAAAGCGAGGgCGCAAGCAAAGATGGTTCTtGCGAATGGTGTGCCTGTAAtCGGAG

CCaTGTGATTAGCCTACCCTCAAGCACAGGC

>contig00658 length=559 numreads=6

aagcagtggtatcaacgcagagtacgccggggatatagagggggctccagcggcaggggc

cgcAGCGGTCCACCAAGGAGGTCAAACTATCGAGTTATAGTGTCCGGTCTGCCTCCTACT

GGGAGCTGgCAAGAtATtAaGGAtCACaTGaGagAAGCTGGTGATGTGAACTaCGCCGAA

gTGCTTCGAGATGGAaCTGGtGTTGTtGaGTTTTCCagACGTGAAGATATGgAAaTGGGC

TATcAAAAATTTAGATGATtCtAAATTTAAgTCTcaCCaGAaCgAATCAGCcTTTGtCCG

GgTTAAGGCTGAAAGTGGAGGTCGCAGCAGAaGTCGgAGCAGATCCCGTaGtaGAaGTCG

GAGCCGCAATCGGAGCCGtaGtAGGAGTCgcAgTCGAAgCCCTgTCAGGCGaCGACGTAg

TccTTCTCGgTCTCGCTCAAGATCACGGTCAAAATCGCcTGCTgCTcGTAaGTCGGGTTC

GaGAtCACcGAgTCcAAGAAGAAgctagtcagagatgcttcggacacttaggagtcccgg

tgtagcgtaaaggcgtgca

>contig00659 length=290 numreads=4

GCAGGAATAGGAAGAACAGGAACGTTTATTGCGATCGATTGGCTTATACATAAACTAACA

ATACAAATATCTAATTTTCCAAAAACTGCAACTTTAGATATAGTTAATCAAGTATTAATG

CTAAGAATGCAAAGAAATGgAATGgTTGAAaCAAAaGAGCAaTTTCAaTTTATATATACA

TTTTTGTAGATATTATATAaTTAATAAATaTCATGATAATTTAATTAAcTTAAaTTTAac

AAGAAAtCAAATTAAAaTTtAAAAaGAAAaTAaGCCGATATAGaaaatag

>contig00660 length=157 numreads=3

ggTTGCTTGCGAAGTGTTGGGACACGTTCGCGCCGGTGGGAGAGTTCATTCCGTCTGCTG

AAGTTCGGGAACCTCAGAATTTgAACTtCTGGCTCAAAATTGATGgACAgaTtCgTCAGC

GaGGCAGCacAAAGGAAaTGATATTTCCCATCGCCCa

>contig00661 length=1189 numreads=52

ccttgactttctgcaagtagaagtcatgttcgtcctcgtcggacgccaccagctcatcca

tcatGgTCCCGCTCTTCTTGCTGCCCTGTTTCTAATGCGCAACTGCTTCTGGTTTACGAA

GTCAAACAAATGACtGTACTCGTCCcTCGGCAGgTTGCTGAaGACGACTTGGgAGCCGTT

CTTCAACTCCAGCTCGAAGTCGAACGTACGGCCCGTGGTGCTTCCTCGAGCGAaGTTCAC

GTTGGCGATCTCGTCGAATCGAATGTGCACGGGGGgCTTGTGCACGAACATGAAGCCACG

CTCCAAAGGGTAGAGCAAGCCTGCGCCCGCCTTGTAGGAACAGGTCACCGAGTTGGTACC

CGTATGCCCTTTGAAGTTtCcAGGAACGgTGATCTTCCTTCCGCAAACCTCTTTGAGCAC

TCTACTGACAACTTCAAAAaGTGCTCCGTGCATCTCTTGCTTtAGTTTATTGCTGTACTT

TGACTCGATCTCCTCTTCTGTTAGGTTGAGCTTGCATGTAATTTCCTCATCCCTCTCGAA

TTGCAGGATTAAAAACGGgTACCGCGTTTGACCCTGACGGATAGGCGGATCCATGCTCAC

CACGAAAAaCATGAATCTGATGTCCTGGTGCGGCAAAAGGAATAAGCGAAGGATGGTGGT

GTGGGGAATCTTGTAATCGTAGGTTTTGCCATGCAGCTGAAGGAAGGTTGGGTAGACTTt

GATGCTGTACCGACCTCTTGGCGTTAGGCAAGCAACcTCAGCAAATTCAATAATGGCATC

GCCAGTGACTTGGATGATATCTGCTTTCGACATGATTTGTTGATGAAAGGCTTTtACtGG

ATCACTAATGGCATCATCACTACCAGATGGCACGTAAAACCTCATCTCCATCAGTGCgaC

TTTCGCATCTTCACTCCGGTGAAATTCTATGGTGACCTCGTTCTTGCCAGTTGTACCCTG

GGAAACATCTTTCAGTGGAATCTCAAAGGCTGGCTTCTTGTCGACTTCAAATGTCATAAC

CGAACCTTTGAACTTAGCAGTTCCCCAGTTCCACCcTTTGACACACAACTCAACCTCTTC

CAGTTGCATCTTGTAGTAGTTTAAAATAAATTCTTTAACCTTGTcaTATtCCACTTCCtt

gaatccatcaaactttacagactgaccattttttagtaaaagcttcaat

>contig00662 length=203 numreads=8

ttAGGAATGCTGGGGCGTTTGCGGTCATCtGCAGCATCGTCTTCTTTGCCGACTTCTTGA

TTCACATcAAAGTCaTCATATCTGGTGGGgTcTCGTCTCAAGTAGCTGCAAAGCAGGACT

CCGACCGTAACAGGACGTCCAGTGGAGAGTCGCTTTTCAGAAATCACGCTCAGCCTAAGT

TTTGATCGAATATAAGGCGCGtt

>contig00663 length=1164 numreads=23

tttattggtggctttgttcggttagcatttcatgattgtgttgggcctggtcactgcgat

ggatgcgtgaaccatggattggctgacaacgcagggttgaaaggatacacagatgccttg

gataaaagCTTTGCAGAtAATGGaCATAAtCGTGTGATGtCTAGAgCTgAcTTTTACGCA

CTAGCGtCTTTaGTGGCTttGGAGAGAGCGACGgActttGTTCCAGATAAGTTCACAGCT

CTCAAAGAcGGTCTGTTtCtaGTTGGCCgcaaaGACTGTTCTGCTtCaCCGAGTGAAGAT

GACGTTGCTGAATTTCCCGGAGCGAAATTCAACATCGACGAGACGCTTaTTTACTTTGCC

AATGAATTCGGTTTCTCGTCACAGGAAGTCGTTGCACTGTtGGGTGCGCACACACTtGGT

CGCAGTAGTATTGCCAACTCGGGCTACGAAGGAAaGTGGATaaGaGGTCAAGTGAATGGG

AACGCACAGTCCGATATTCTGGACAATCAATTTTACTtGCAAGTTCGAGGAGATTGgACG

CAAGTCCccAtcAAGGCgaGCGGTAAATTtCAATGgCAAAGACCGGCCACATTGCCTAAT

ACTGGATCTGAGGTCAGAAACCAACCAAACaTGTTTTtAAataGCGaCATGAGTCtGCtG

TGGgACCTTGATCcAGTTGGCCAAGATGGCAGCGTGCAGTGTCGTGTcAACTGcaTAGAC

GCCACGTGTtGCCcAAGGTCGCTAACAAGCGCACTCACCCGAaGCTACGGCAACTCAAAC

aGCCTTTGGCTAAAAGaCTTTACTGCGGTATTTTtGAAAaTGATCAATATGAATGATGCT

ACGCTGtCGAAGCCATCTGCAGCCGTAGCTgAAGATAATCCTAATTGGATCGACGACGTC

ACGCAGAGCTTGgACGCAGTCAACGGTCTCATCGAAGACATTGTAAACGACATAAAGCCG

TGATGAaTCGCTGCTACTCTGgCAcGGCACGCGTAGAACATTtGtATCTtCCTTTTCAAA

GACGACTTtGACTCGGtAATCACGAATTAGTgAGgTGCAcGCTAAtGTTCTTtCATTCAT

AGTTTCCCtcATGGACAACAaTTCGAaTTTGAaGgCgCAACAGGCAGTGAAaGTCGCCcT

GACTCCGccgAaTGATtGAAAGGt

>contig00664 length=547 numreads=12

ccTGTATTTAGGTTTGTCTGGACTTGCCACAGACaTAGAAACTGTATCCAACcTGgCACG

gTTTCgtCTGAACTTGTACGAGCTTCGaGAAGGACGAAAAATGACGCcACATGCGTTTAT

GAACTtGGTTTCAAaCATTCTATACAGCAGGAGATTCGgACCCTATTTCGTAGAGCCGAT

TTAatAGCCGGTCTAGATCCGAAAACCGGtGAACCTTACGTGGCTTCCTGCGACTTAATT

GGTTGCCCTATGGTACCAGAGGACTTtGTGGTTGGTGGCACCTGCGGTGATCAGCTTtAC

GGTATGTGCGAaGCTCTTTATGAACCTGATCTCGAACCCGAcGATCTCTTTGAAAGCACT

GCCCAAGCTTTACTAAATGCGCAAGACCGAGATGCTATCAGTGGCTGGGGCGGtGTTGTt

CATATCATAGAAAAAGATAAAGTTACTACAAaGACCTTGAGGGCAAGAATGGACTAGAGC

tGGGTCTtCTGTATtGGATGGCTCtaCTAGAaaTtGTTTATGGCTTgcAATCTGCGtGgt

AACAAcT

>contig00665 length=592 numreads=14

GGTGCGAGCAGTATGACATCTTTCACCAGCTATGGACAGTCTTACTTAGTGATAGCAAAT

CGTGAGGATCGTTCGACCGATGGACATTCGTCATATGaCGTGGCTGTGGAAATATATGTT

TACGTGAACACgACGAAaTCATTCGCcTGGTTTCAGTCTATCCCCTCGTCTCCATCCATC

CACGTGGAGTCTTTTTATgTGGGAAACACTGTCTACTTAGCATTGACCAGAGTTGACCAG

CGACTTGTCATATACGAGTTTGGATTGGAAaTTGGCTTCAACGAAATTTACAGTATGGCG

GTCAGTTACCTCCAAAGTGCTCGGTCcTTCAGTACAGATGGGGAATTCTTCCTTGCTGTC

TCGGCAGAAaGCGCCCcTTTACcGAGTAGCACACTTGtCGACACAAAaCTCCTCAAGTTA

GAAaCTGTAGGTGTTGCGACTGACCGGTTGAGCGACGTTTTGCTCAAGTGCTGAAGCAAT

GAGAATGATCTcTTATTAAGCTGCGTGTAGTTTACATGAGGAGATTTACTATATTCTGTA

ATCATGTTGCAAGTCATCTTGGAAGGAAAgtGgCgAaCAggTGCCATTGGCG

>contig00666 length=499 numreads=15

GATGGCAGACAGTGCCCAGGGAACCAAGTTtGGTCGCCGACTGGTGCTGAGTGTGCTCGC

ACATGTGAAACGATCCACCTACCTTGTCTGAACAGCATTCACATGGCAGGTTGCCTCTGC

CCTGGAACCTCcGTGTGGAACGCCCAGCTGCAGATGTGTGTCGAACCACACGACTGTCCT

TGCCATTACCAGGGTAGGAGTTATTCGGAAGGAGAAGCaTTtAAaTGGGACTGTAATACT

TGTacctgCGTAAGTAGCaaTGGTCATGTACGACCAACGCTtGTCCAGGCACGtGTAGAA

TGTACGGAGATCCCCACTACACCACGTTTGAcGGCAAACGTTTCAAGTTCcAaGGCCGGT

GCGaTTACAtCATAGCGCAAGaTTTTTGCAACGGCAATGACGGTGTATTTCGTATACAGG

CTGAAAATGTACCATGTGGAACTACGGGTGTCACGTGCACTAAGTCtaTCACGGTCACTC

TGCGTTGATACCACTGCTT

>contig00667 length=241 numreads=3

gTTAGAaTATCcAaGGAAaCAGaGTCTGcAGTcTGATTTAGCGCTGGctCCAGCATACTA

ATGAaGAtATTGAACTGCTtgAaGTtGCcaGTCTTGCGGCTtAGCtCTTcGATGtAtGCA

GCATCAaaTGTTCCAcTCCAGTTGTCAAGCGATGAACAGTCTTcaacctccaaacccaag

gtatcgccgtttacaacattgatggttgctttatatttaatacctctaacctctaggtgg

c

>contig00668 length=238 numreads=4

AAGCAGTGGTATCAACGCAGAGTAGATGGgAATCTTGGCTAGGTGACCTAGACCTGCTGC

GTAGTTTTAAGGTTCCGCGCTGCTTTAAGCCTAAAACCTtcGGGACAgTAAGTGCaGCgg

AgTtACACCATTTCTCCGATGCATCTACACTGGGCATTGGACAGGTCACTTATCTgCGTT

TAGTtGACGAACAgaatcaagttcacattagcttcgtgtctgacaaaggcaagagttg

>contig00669 length=996 numreads=16

aaatgcctaaCAAAATAATCttGTTGTTAAAAATGAAGAATATGTGCTAAACCTTCAGGA

CCACTAAGGCGCcTTtCTAAGCCTTATATAAAGCATCGGGAATAAGTACAGTCTTTCCTT

GGCCTTTCGTCGTCATGATGTGgTtCTGTGCCTCACCGGCATGCGACAGTGGAAAGCGCT

ttCCAACCCGAGGCTTGATCCAGCCcAGATCCAGCCCaGAGTTGATGTGCGCAGCCGCGC

TTGCGTgCTCTTCCTCTGAATTAGCTGTCGTGTTGACACCAAAAATCGTTAtGTCTTTtG

CAAACaCcaaGcGAGgggAAaaTtGgcAATCCCCTTtACAACcGATGATAGCAATGCGGC

CTCCCTGCGCCACAAGACTCAAATCTTTTTGCCAAGTGGCATCAGCAACCAACTCGACGA

TGGCTGAAACGCCCTTGCCATCAGTGAGATCCAAGATCTTCTGAGTATAACCGTCTTCAT

AGTGATTAAACACGAAGTGAGCACCGTTGTCCTTGACCTCTTGGCAGCCCTGTTCCGTAC

CGGCTGTGGCAATCACTCTCAGCCCAAGAGCACGGgCCAATTGCACAACAGCAAAACCAA

TtCCTCCGCTAGCCccATgAACCAAaCaCGtCTCCccAGCTcGAGCGCGTAATTTctaaa

taaaacccaaacgataattctccagtagaaaattttacttaaacctataccagctcttga

tgttcgtttaaaggccgaataatatcaaagcctatactATAACTCACAACAACACAAGAT

ATggCGAGAAGTCAGGCGGATATACCCAGCTCAAAGCGGCCTCGCCTGAAATTCTgtctc

atccttgccgctcgaggctttcgatgacgcacaatcttgtaattctctaagggattatcg

catcgggtatatcatactaccagtggctgtaacgggttacgcagtcttcaacaaagtcaa

atgaaatccggtacagcaaactggcgggaaggctaa

>contig00670 length=236 numreads=1

aagcagtggtatcaacgcagagtacgcgggggctaccattggagcattctggtccggaat

tgaagacgaatagtgagtattgatgcttaagcgataagttggtatattatagaagcatga

gccacatggagcagaatttaaaggaggaggaagcagaagatccgatgatgacatcgcatc

cagtggccgatgccaactcaaactaccaaaccggctctgtgcagccaacgcaaaca

>contig00671 length=436 numreads=8

AAGCAGTGGTATCAACGCAGAGTGATAATCCCCAGCCCGGATCCATTCCTTGTCAGTTTT

GCACCAGCCCACATTAAGCACTGCGTTCAGAaCGTAATCATTGCTCTTCCTTGGtGTCaT

GTCAAaGGCATAtCGGATtCGACTGCcACGGGTTATTtGAGGaTTGGGTATCTTCATACC

GGcGATtACGGgCACATCGCAGCCTTCGCaCTGGaTGTtCTCGCGGGCTTCAATGGCGAG

acAggacccaggagccagcgactgcggtgcgtcagttggaaaaatcacgtgaccagcaat

tcttacattgccgccatttggatcaggaagatcTTTAACGcATTTCTTATGgCGGGCATG

AGCACTTGATACCGGCGACAGGAATGTCCTCAGTCTCAGGATGCcTCCCCGCGtCTCTGC

GTTGATACCACTGCTT

>contig00672 length=241 numreads=1

aagctacgctttgatttggagcgaggatcacgtattcaattcaagaattgattatactcc

tggagcgactacttaagcgaatatgcattccagtacaacactggcgcacaccaaagtttg

ggtggtctctgtccgttccaagtgttttttgggcgcaaaccgaacgtggatcaaattttg

ttcgaaagcttttcagtggatgcagaagcagcagcgaccacattggacaactgaaaggaa

g

>contig00673 length=499 numreads=4

ATCCGCTTTCAAATCAGCCGCTCATTTtGGAATCGCTAGGTCTCGATCGACTCAAATCCG

CTCTGATGGCGCTTGGAATGAAATGTGGTGGCACGCTTCAGGAGCGGGCTCAGAGGCTCT

TCcTTGCCAGGGACACACCGgTTGACCAACTCGACCCCtCCTtGTTtGCGAaGAGCGTGA

AGacGAAAGCGAAGAAGAAGACTGCAAGCTAATCcATTTTTTtCAAAGCAGCGCGTGCAC

TGCttgacgggagttttatttctttgctaggttttcttgtgtgtGGTGTTGCTAACCCAT

CCTTTTATAAGTCGTTATTTTTGCACTAGGACTTACAAGTTAAGGTGTGTTGATAAGCTA

GGAAAGCCTTGTTAGTTATAAGATCGTggggcattattaggcctgccatcgcagcacgtg

gaagttcttggcatgtgcgttttatcggaatgttatgagtttttgtgacaatgcctagga

acggaggtgacctttcgat

>contig00674 length=361 numreads=12

TAcAtATAGGAAGAaGgACTGATGAAACTTTTCCAGCAAGTtGTTGAAaCTTCGAAACAt

GCcTtCGACAAGCCTGCCAGCTATCTCAAAaTCCACCAAATGCCTATATCTCCTGTTGAG

ATCATGCCTaGgCTGCATGCCTTGCAAAGTGATCGCCTCGATGCGGTACTTGTAAAACAT

TCCGTGATTGCCAGtCGGATAGCCCGAGGCTAACACTAaCATCATTCTGGCAAGCGTTtG

CaGTCTGTTAAGAAAGgCTTCATGGTcAGgCCTGTGaTCTTGTgcGTAAACCTGgTACTG

AAATCGTAACGGAACACCcTCtaCaTTGCCGATCCGGAGGGCCAAGTTGATGAGATCCAA

g

>contig00675 length=388 numreads=7

aaCCACAAATTATTTTgCAGGtGCTTCGgCGcTCTTTTCAGATACCCAGGAATCaTTCTG

GTtAACcTCTtCCTTACTTgCTTCTATTTCAtcAAGACGCTTATGgCGAaTTAAGTCGTT

CATAACATTCGTCATTGATGCAGTCTGAtCCTTCTTGATTCGCTTCGCAGCAGATGGAGT

CTTCCGCTTTGCTGAaGGgCTCTCAATGTCTTCAGTATGAAAATCAAaCGCTTCAGCCTT

CCTAGGGCCAAAGGAATTCTCTTGATTCAATACCACAGGTACCACACTTCCTTCCAGTCG

CTTTTTGTCTGCAGGTATTACAACCaCTGGCATATccttgtcatccttggaattgcttcg

ttgctcacctagtctagggcaataacta

>contig00676 length=321 numreads=4

AAGCAGTGGTATCAACGCAGAGTACgCGGGGGAGCTTGCAAGTGACTTTCTGGATCGTAG

TACCCCAATCTTCCGAGATGATGTGTGCTTCTTCATCAGTCAGTCAGGTGAGACGGCCGA

TACACTGATGgCCcTGCGCTATTGCAAACAACGTGGCGCGCTTCtGGTTGGGATCACGAA

CACCGTTGGCAGCAGCAtATGtCGGGAGACGATGTGtGGCGTGCATGTGAACGCTGGACC

GGAGATCGGTGTCGCCAGCACGAAAGCGTACACGAGCCAGTTCCTCGCTCTTGTCATGTT

CGCCTGCATGATGGCCGAGGa

>contig00677 length=127 numreads=3

TCATCGCTGGCAAGTATACATGGGAGTTAAATTTTGCAGCCGTCCATTTAGCAAAGCAcG

GGCAaTgaTGACTGTCCCaGATTGAtaGGTAGTATCAtGGTACAATACaTtGAAAATCTG

CGTTGCT

>contig00678 length=499 numreads=5

gCTTGCCGACTATAACATGATTGTCGACAAGCTAAATACGAACGCAGGCTTTGCAGACCT

TGAGGAAGACTACGTGAaGgCTCAAGGGTCAGAACGATCGGGAAGTGAAAGTTATtGATG

AGCTTTTTGCTCAGAGACAaGaCAAGGAGGAGCAGTTAAaGCGaCTtGATATtGAAaTGG

ACcAAgAAaGGCGCATGACTGAAAGTGTTGTCGCGGACATGGGCGCTGCTCAGAAAGAGA

AGTACgcctttactaaaaagtCGGAATACCTCTCTCCAAGCTGACCTTGAAaTAAaGCAG

CAGGAATTAGATCAaGTTTaaagccaaaattgatGCTATGGAAGAGGAAATTTCACAGTC

TCCCGTTAAGCAGGAAGCCGTCACGCTGCACGAGAAGATACTTGAACTAAAAGATAAGAA

AGCTTCTTTGGAAGACGAGGTGCGCAAAAAAGAAACTCCTGTGGAAGAGCGAGAACGCCT

GCTAAAACAggtcaaaagg

>contig00679 length=230 numreads=1

aagcagtggtatcaacgcagagtacgcgggcatgtcggaaattcccaaatttttcaatac

ttcattgtgaaccagcatgaccaaacagcaaaggtcatcgacacgatcaaactgccgact

tgcacgcacttcgaccacgaccagaacgtcactcttcgaggaggcatcaaagctggcaag

ttcaccaagcatggcgtcgtcagcaacatgaaggactgcattgatgcctg

>contig00680 length=241 numreads=1

ctttggtacctcaatgagacagtcttttttaacaaaaaacactctgcaacacatactaat

gagtaggggaagcagggtttcgccaaaatcccctagatctgaccttgcgggtcataagtg

agcttttctctcttgtccagagtcttcgagcattttttcaatttctctaacttcagccct

ttcgactttcaactttctcgtgacatctgcgaagagcgacagactctttgctcgtatcaa

a

>contig00681 length=983 numreads=12

tcTTTTGACAttttgACGGCACCcATCGACTGACACTTATCATGCACGTTATAACCGCaC

tGcGTgCaTtGAAGGCCTgTtCCTAGTCCCCAGATACTTGATAAGCAGTAATTAcaacta

gtagcggtcaggtaattatggtagatgaactgatgagggtgttccaaagccaaaatcATT

CCGCGTAaGTTcGCCAaCTtCGGaCAACTtgCCctGCGtCaCCaTCTCcACTGTtGCGCG

TTTGTGCAtagcagaaatcctgtctttagccccgagcaatttccagctttgtcgattaTG

TCGTTTATCCTGAACATCGAGCTCGTGCcAGGTCTtatGCcAcATCtGCGtaTGCtGGTC

CAGctcgtcctttaaacggttgagttcattcagaaggtacgtggtctcgtgacgaggaaa

ggcaagagtcgaaccATAGCAGCCGGTCAAGCACCTAGAAAAGTCTTCGCGTTCGACGTC

GGCGCCCAGGGAGCTCGACTCGcctGTAGGGTTTTCCATCTCGAAGTCGGACAtCaaCTC

TAaGTcGTAAaCagggCtCGtATACAGgTtctCGTCcGTGTAGTACTCCCACACTTCTAA

ATTGGCGCATGCGCAGAATGGTGCTAGTGGACCCTTCtGCAAGCTtGGCtGgtATTTAAA

ATTTTGAAaGaCGgTAAACTTTGagCaGtGTACgTCaatgtagtcccatatcgaaactcc

tatAAAAGTACTTGTCGAAAGTGTTGACTGTGGAGTCTTAGCATCTTTCGGCAACTCATG

TCCAGAGCCAGCTTTCTTGCCGGTACCGACACCAAACTTGGGGAAACCACTTTGCTTCTT

AGCGAGCTTGATCTGCGCGTCATCGGgTGGCATACTTCTTCCCAGCAGACCGTGCTGACA

GCGTTCGTACTCCGAATCAAGGAGGAACGTGTTGAAGCGCATGGATACGTGATGAAACGC

GAGAAACCGCAaGGAAAAaCTCg

>contig00682 length=576 numreads=11

GAGAAGTGGCAGGAACcTCCGCcTGTTAAGgAAGtAaaGGCGTTACcTCGgCcTGaTGAT

GCtCAAaGACccAAACGAGGAGgCCGCAGAGTTCGTAAaGATGAaGGAGAAGTTCGCCGT

GACTGAaATGAGAAGACAAGCTAACcGAGTCCAGTTCGGCCAAATTGCCGAAGATGTTTt

CCAGACGGAAaTAGGGTTCGgTATAGGCTCTCTCGgCCAGAAAGAGTCTTCGGGTAAAGT

GAGGAaCCCTGCcATGGAGCAGAaGAcTCGgATCtCgaTATCAAAGCGACTACAGCgCAA

TTTGGCGAACATGAATCAGTCGTACGGCGGGAAGgTcTACGGTTCGTACACACGtATCGG

GCACGGCgTCcAGCGTTGCATTCACTCcACTCCAAGGAATCGAGATCGTCAATCCGAAGG

CTGCTGAAAAACGTGTGCAAGAGGCGAACGCAAAGTACTTTTCAAaCGAAGCTGGcTTTT

TCAACGTCAaaaGGAaGGAGAAGAAATCTCAAgACAaGTAGTTTtGAAAcAAAAAAAAAA

AAAAAAAAAAAGTaCTctGCGTTGATACCACTGCTT

>contig00683 length=468 numreads=16

TTTTgCACCGCgCTTCGGTGTCATCATTgCTACCGgctGGGATTACACTAAGCAcTGCGG

aGTGGTAAGCCTGCATCACCACTTTCAGAGTCTTGATAgATCtAGCcTCTAAGCCAGCcT

TCCACTtGCATATCATTTGAGGGgTAACAACTTTTTTAGGCCGAAGACTTTTATGGCCAA

CACTGTCCACCTCAGCTTCCTCATCTTCGTCAACATTATCAGCAGATGCTTCCTCTGAGG

GTTTAGAGGTCTCTCTCAATGGTGCTTCCTCCTTCCCATTTTCTTCAGTACCACTATCAT

CTGACTCATGGAAATCTAAGAGATTAGGCTCATTGaCCAGCAAGAACTTGTAGAACTCTG

GATCCTTATCTTTTAAGgACATCATTtGAGCAGCATGCTcTGCCTCTTCAGAaTtGAGGC

TGgAaCtCTTTTTAAtGGCTTCGGAGGCACTTTCAAGTTTGATCCACt

>contig00684 length=114 numreads=1

aagcagtggtatcaacgcagagttaagagcagaatgggagagattcttagacaaagatgg

cataacgacaatagcaacgaggatggacgggagtcgagcctaaaaactagaaag

>contig00685 length=512 numreads=16

gAAGAAAGTATTTtCCAAAGTGAAACGAaGGgaTACAaTTgTCTTCAaagACCCTAAcTG

GTCAGAGATCGTCGAGGAAGATGAACCTATtCCTGAAgAGGaGgAAGACGACAGtCGAGA

CAAGggCTATGTTcTTGTTGATTCTGACTCCAAAGGGAATGTGTCAGTTAATGAAGGTAA

ACTTTtGTTCGgCAGGGCTGGCAGGGATGGTAGCAAGGCAGTAGTGAGAACAGAGGCAAA

GGGTAAATTTCAGGCTGGTGGGGGGAGGCATTCTTCAGCGACAGATGACGAGAAGCTTGT

AGGACTGTCTGAAAATGTtCAAGTGTATtCCCcGAGGTCTTTTTTCAGTCCTGTCGATCC

GCCATCTAGCCTGCTCGCTCAAGCCAGAGAAAaTTaCGTTGGAGAATCAGACTCCTGTAT

AGGTTCAGAGGTAACCTCGCCAGTGCACGaTTtGGCAGAAGgTGAgAGaGAGCCTCGTGG

CTATGATACGGGCAGTGTGTGTGATGAAGGaa

>contig00686 length=237 numreads=1

agcttccattagctcggtaaggaggttagcttcattatgagctgtgataatgttacgaca

aagcttctagctgacgtctcacagtatcttgcaactcatgccgattaagccattccataa

gatgaagattgtgaaccagtttgtttgctccgaattgcgccggatattttctatctatga

cttaaccagcacgtgggtgataaggacaatgtgtcgacaatgatatctccactagtt

>contig00687 length=1025 numreads=39

AAGCaGTGgTATCAACGCaGAGtACGCGGGGCTtGCagggCTTGTGCCCTTACCGGCTtG

TGTCAaaCCGCTACGGtAGGATTGAAACCTTAGAACGAAGGCACTGTGAAAGAATtAAAt

tgTTTTtAAACGTAgTGGgaCGtAaTTTTCGAGAGctAGTTttGTATCGAtCGTGaCTaG

AATAGCATGAAACTTTGGTCAAGtGAGCACAtCTtCAACCACCcTTGGAGTACCGTCACC

GAGGCCTGCTGGAGAAAGTACCCAAATGAACTAAATCCAAATGtAAAAGCAATTGATGTG

ATAGACaGGAAAATTACGGACAATGGATGTCTTCTTACAACTCGCATCTTTGGATCCAAC

TGGAATTTACCCAGCATAATtACCACCCTTTTTGGAATGCCAGAAATGTGTTATGCTGTG

GAGCACATCGAAGTAGACCCAAATAACAAGAAAATGACCCTTAAAATGATAAACTACACT

TTCTGGGGATTAATGGCTGTGAAAGAGAACATTGTGTACGAACCATGTGCGTCAGACTCA

GAAAAAACTGTAATGAAACAGGGAGCAGAGATCAGTGTtACAGGCGTCCAATTCAAGGAC

TACCTGGAGGGACTTATTGCTAAGGGTTTTGAAGGGAATTCATCAAAGGGTCGCGTTGCT

CTCGAAGGCGTCATCGCGAAGATCAACCTGGAAGGCGCCCTGAGGACTCTGAGCGATGAA

GTCCAGGACCTGTCGCGCTCCTTAGATAAAGCTAAaGTATTtATTGACTCCGAGGTGCAA

GAGTTTTCCAGGAAGcTtAATTCGGAGCtCACATCGCTCGTTAgCAAAGTGGACGATGAG

TTGAGCCAAATTACCgTCAATATTtcGCCCATGGTGGAGGCaTCTAGAAGCCCTgCGGCG

ACTGACCTTACCTCCACCAACCTGACTGAGGCGGTAAAGCTCGCAGGCCTTAGCTCAAAG

GCCTTCGAATGACATGTTATTCGAATGAAATCTTCGAAATTAATAGAACGGATTTGTGTC

GCCaa

>contig00688 length=923 numreads=56

TGTCCTCCCGATGCCCTTTCCATTCCTTCTCCAAGGCGCCcGACCGACTATCCCAGCTGC

GAACTGCACCATCTAAGCAGCCGGTGAGGATCGATGTCGGATTTGACTCGTGCCAGATGA

CGCGACTGATCCCATGCGGGTGGGGACATCGATGGCGGACGCGCTGCGAAGGAATGTCCC

ACACGACAAGGTtCCCATTCAAGCCTCCACATGCTACGTACTGCTGATCTGGCGAGAAAG

TAAGCGCTTCAATGCTACGCAGTTCCTGCGAATCACTGGCTTGCGACGCGGTCGAGGCAG

GACCAGAGCTGTCAAGCAAGGTGCCCACAACCTTTCCATTGGATGAAATAATTAACTTTG

TTGTTCCGTTTAAGAAACCGGACGCCACCAGGTTTCCATTCTTGTGGCATGCGACGCAGG

ATACAGGTACGCTAGATTCACCTGTTATGTGAAATAATACAGAAGTTGACTTCAAATCCC

ATAGTTTTATGCTTCCATCCTCATAGCCAGCAACCAACTGTCTTCCATTAGGTAGCAATT

CGCCAGCAGTGCACTTAGAACCATGACCATTGCAGATTTtACACTCGCCACTCGGCATTT

TCAACATGTAAAGATCCCCATCAGTTGTGCCGGCAAATAGCACATAGGCGGATGGGTGCC

ACTTGGCCCACTCGATATCGGAGCACTTGAAATCCCAAATGCTTTTGGCTTCATCGACAT

TGTGGACCACAATGACGCCACCCATGTCGGCCGACATCACAAACCTCCCGTCATAcGAAA

AACCGCAGCCGACTACCGAATCATTGTGACCTCCTAGTTCAAATAAAaCaTCACCGCTTT

CAGTGTTCCAGACAAAaGCcTTAtCATCCTCACCACCAGACACTGCCATTttCTtGTtAT

TTGGATTGAAACCAACacagaat

>contig00689 length=255 numreads=3

ttAAGCTAATTTTCATATTCACCAGTTATGCATCCGCATTGTTCTACTtCAGACCTTTgt

ATCATGAATTttCGGCATTTCAGaGgCCAGACTtAcTtCCTTTCTTgACTAGTATCAaTT

GGAcATAaTCTGCTtCGTCTGCGAAaCcATCcTTaTCCTTGAATTCCTGAtGaTTaTTCT

CcTTTGATACTACTGTATCGACAAAcgtgacgtaatctttcgtgccgctcggttcttagt

tatcttgctttaacg

>contig00690 length=675 numreads=11

ttAAGAAGATCACTCAGGATTTAGATAAGCTTCATGTAGCATTCATGGAGGCAAAAGACA

TGATTGTTTTGGAAGGACCTCCTGCTGAggTtCAGCAAGCAGAAGCTTTGCTAGCGACAT

CCATCCATGAATTGAAAAATACCATGTCATTTGCTGACATCGaaaTTGATCAGAAGTGGC

acAGgCATaTTATcGGgAAAaGTGGGGCcACAATTGGGCGCATTAAAAATGAAACGggCA

CTTCGATCAACATCCCACCCGATACTGAAAAaTCTAACATCATTCGCATTGAAGGCAGCC

CGGAGGGCGTGGCTGCCGCTAAGGCTGAAaTATTAGGCATGgCCGCTAAAATGGACAACG

AAAAGTCTCGTGATaTAATtATCGAaCAGCGTTTTCACAAGAaTATTATTGGCCAgAAAG

GtGAGAAGGTTAGGGAGATTCGTGAGAAGTTtGGGGACGTGCAGATTtCTTtCCCAGAaG

CtGGCAAAAAAaGCGACATCGTCACTTTACGCGGGCCcAAGgATGAGGTCgacAAatGTT

ACGCGTtCATGAAgAAATTAGCCACTGAACTGGTTGCATCCAACTTTCGCCTGGAGGTGC

CCATCTTCAAGCGTTTCCATGGAAATGTGATCGGACGCAATGGTGCTAACATAACaaaaa

aaaaaaaaaaaaaaa

>contig00691 length=147 numreads=1

ggcaacttgtagcgtgcgacatccaagataagaatcaaatcgcgcttcttgtggtagccg

ccaatgggcacgaaatggccactgcccgtttgcagcatcgtcgcgcgagcatacgaacac

acgagaacagtgtcggtcgaccggcaa

>contig00692 length=233 numreads=2

GTGAAAGAATCCTGTATTTACTAGACTGTGTGAGTAAGGAGTGATTGTGGAGGGTGGTGA

ATCGACATGCTGTGCATATGTTTGGAATGCATGTCTCACCGTATCTATTTCTTCTCCAAG

TATGCTCTTCAGCTTTTGAACCTTCTTACCCcAATCCTGtgttggtacatcagttattga

ttgcttactactatcatcatgcatcaaatgtgtggaagataagctccgtccat

>contig00693 length=212 numreads=4

AAGCAGTGGTATCAACGCAGAGTACGTTTTCGGGATgATTGCAGcgACGaCcGAGtAaaG

cAAcTCCTATTgCGAAGAGgCtGGCAAAAATCCAtATTGACTTGGGAaaCTCCAGGAGAG

AATGTCCTTGAATACAACAaTTAAACTTATACCTTAAATCTCAGGACCAGAGCTAACGCG

GGAGAAGTTACGCTAGACGCGGGgAGCATCGc

>contig00694 length=241 numreads=1

agacgactcttgcgtaatcagaagaaacattctcaaattcgaaagtgggactttgaaatt

cgagcaagatcttgatatcgccattatcggattaagtgtatcggccttgttaatcgtcat

ctccctggtcgttgggatcgctctcgcttgttcgaggaacccgtccaaatactcaactgt

ttggctcgctgttcttgcgttgctgagtggactctgtttcttgtttcccgccgtctggct

t

>contig00695 length=236 numreads=3

gACAACGCTGCTATCAAGCGTTGCCTGTTTAGTGCCACGCTAGCTAATAACGTGGAAGAC

TGGTGCAAGCTTCATcTTGACAACgTTGTTAGAGTGACTGTTGGTGTtcgTAACtCTGCA

gCCGATCTCGtGCAGCAGGaGCTCATGTTCGTCGGaCAAGAGGCGGGAAAGTTGCTCGCc

ATTAGACAAATTATTCAGCGAGGACTCCAACCGCCCGTTCTGGTGTTTGTGCAGAg

>contig00696 length=373 numreads=4

AAGCAGTGGTATCAACGCAGAGTTTCCGATATAACTCCTCTTCCatAAATTTATTTCAGC

GTTGCTGCCAGACTATTGAAGTAGTCATGCGCTCAAAACCGCCACTTCGCCTGCAAAGCC

ATATTTGAGCGCAAACTTACAAGAGTCCCAATCTCCCAGGACAGGTCATTCGGCAAAaGC

ACTTTtGATAGATTACGAtCCCCcGgAGgCTGCTACtGGTAtGGCCTTCATTTCATAGCG

CATACagaggtactcgctcgtcaatgatgtaacagtgatgcagactatatgcaacaacca

ccaaccccagtgagacgggaaagcaaatgccatccagcaaaataattaaatgaaataggt

gagctgtacaagt

>contig00697 length=257 numreads=5

AAGCAGTGGTATCAACGCAGAGTACGCGGGGGgCTGCATAATTGTGATtGGTAGGAaGAG

TTGATGCGAAGCTGCTAATGCTGTAGTATTTCcTTTCTACTTTCTTGCTGGCTCCGTGGA

GCGGAGGCATTAGCGCAaGTTGGGAAgTACTCCGTcTATCAAGGCCTGGAGATGCTGACC

ATAACGgTTAAAAAatAATCGtAGAAAGTCGGcAACAATTTCCAGAGTCGTTCAacaaga

aaataaggaagatttca

>contig00698 length=159 numreads=2

aGGATTTCGAGAAGGTTTtAaTtGCGACTCCATATGCTATGAATTgtaccactatgaaga

atgctaaattgtgttagtgtttatggttgctggtcggctaagcagcccgattctgctact

ccatgccattttccgctggtgtttgtatgatgtaaagca

>contig00699 length=240 numreads=4

TGGGCcTaGCTCTCcGAAAAAGATAGCAACctCTCAGTcACCGCCAGCtGTTAAAATAGA

GCCGTCGCCTCAACCTGCCGCTGCCgtGcGtaCcacaccAGCAGCtAAGatGTCCACTTC

TTCCAAtGTTAAAaCTGCtCAAAGTCTGCTGTAGCAGCAACtcaaattgtgcgcacccct

gaatgagaaaaaagctcatggttcatacctattagtggatttatttgtgcggcatgtcaa

>contig00700 length=241 numreads=2

aagcagtggtatcaacgcagagtacgcggggATGTGAAGGTGGAGCTAGGTTTGTCTTCC

AGGTCAGGAATTGCCGAAGAAAACGTCTGCTGTAACAAACAGCGCTAGTGAAATGTTAGG

GGAGGTGGATTCTCACAGAAGCTTGGGTCCAAATGCAAGAAGTCTTCGCTTGACCGGCCA

TGTCGGCTTTGAcGgCTTgCcAGACCAGCTTGTTAACAAATCCGTCAACAAAggATTTTC

G

>contig00701 length=516 numreads=9

TTCAAAAGaTTTTCAAGATGAGTGATCACCtGTACTTGGGTTTGTCTGGGTTGGCTACTG

ACATAGAAACAGTATCCAACCTTGCACGATTTCGACTGAACTtGTACGAgCTTCGCGAAG

GAAGGAAAaTGTCACCACATGCCTTtATGAACTtGGTTTCAAACATtCTTtATAGCAGAa

GATTCGGACCGTaTTTTGTAGAGCCGATCATAGCTGGGCTAGATCCGAGAACAGGTGATC

cGTaTGTGGCaTcgTgcGATCTgATAGGTTGCCCAAtGGTGCCTGAAGATTTTGTTGTTG

GTGGAACATGTGGTGACCAGCtCTATGGCATGTGCGAAGCGCtCTGGGaGCCTGACCTTg

AACCTGaCGAtCTTTTTGAAAGCGCcGCCCAAGCTTTaCTAAaTGCACAAGACAGAGACG

CTATCAGTGGTTGGGgTGgCGTTGTTCATATAATAGAGAAAGATAAAGTTACTACGAAGT

ctttacgggcgaggatggactagaaagctagatctg

>contig00702 length=259 numreads=4

AAAATGGTTTAAGAAAATCGGTACTCATGACAAGgCCATTCACGGCTAGTCGTGAAAGCT

AGTTTTATAaGCAGGgAGCAAACAaTGAGAAAAAATCTTTtgAAATTCGAAAAGCTGACT

GAAaGTaGGCaCTGTGTGCAGAGATtCATATCACGCGCATATAATTTAAGCATACaTAAC

AAAAAgTGATTCTGGTgaTAAAACCatGTtgAttcTTtaCtCACCATaGAgTTTTgAAAC

CTGCGATGGGGcATGGGCG

>contig00703 length=474 numreads=8

AAggCGACGATCACAAAACCTAAaTGCACTAGTCGTATTCCTTCGCTAAAGCATTTAGGA

CTGTTTATGTCTTTTCCAGTACTTTCTCACTTCATCATGACCAGCTTTAaGAAACAACCA

TTAAACGCTTCGTGCtgtttccccataacaaaacattaagatcttccgcatacTTCCTAA

GCATTTCAAAATCACTTTGAGACAGAAACTGATTGTACAgCACGCCCTcGgTGTATCGCA

ACCgATcTCgTtCCATttCCCACAaTTTGATCtGATCcGtGATCGTTGGGGGAATTATCG

GATTCTGaGAAcGCATCTGTGGGTGgCCACGAGTtCTCAAAAaGTCAATGATTtGCTCAG

CCGAAATTCCGCAaGCcAGGgCTTGCTGAACGCTTtCCCTGCTTAGAAGACCgaCGCAAA

aTTCTGgAAACCTACaTTtAATATCtGTAAaCAGACTTAGAAgAGcAATCtGcA

>contig00704 length=238 numreads=1

aagcagtggtatcaacgcagagtcttgtctttgatgttctggtttgtatgacaggcctgg

cgcctttgtggtcttttgctatttatggttcacccgcatagctgttcgttgttgtccttg

ccctttcttcaatgtcagtatcggtgtggctaatggatggcggcatcaaatcggggcaag

gcttgggctgcgacagtatgccatgccaactgtgactagatcacaacaactgttcatt

>contig00705 length=238 numreads=3

TACGCcGGTCcACACACGGCcgTATAAgATGGGCTCGCCcaCTCCTGAGcAGATCcTGAG

TtCTCACctCCTCTAGAATTTGGCTCACAGAAACTTCAACTTTGATGGGACGAAATCTAT

TCAAATCGGACGGAACACTTTTGCCATCATCACTGTCTTCACTGCTATAATCGTAGGGCT

CAACCTGAGACCGCCACATGGACAAGAGATCTGGACTGGTCCtGTCAGAAaTGatgag

>contig00706 length=229 numreads=5

AAGCAGTGGTATCAACGCAGAGTACGCGGGGTATCcAGCTtGCCATGCAGCAGATACAGC

CAcAGCTGAATCAAATaTTGAGTTCAcTGtGtCCgCAGCCcTGTcAAgCCCCAAcATGCC

cAGCAGGcTGTCCTTACACACAGCTTCcAGCTCCCTCGGTGGtGTGTCCTCAaCCTTGCT

ACTCAAaTTGTCCTCCGGGTTCTaCCaGTCTCAGCAGgTTCCAGTGCCG

>contig00707 length=798 numreads=22

aaTTTCAGACTGCAAGCCAGATGACTTAATCCAGACATTATTATTATGTGGGTGATAGCT

ACGACGCTTtgCCGGtAGAACAGgAAAAGgTGTCtCTTCcTCATtCtCCTTTGTATCAAA

GCTGTATGGATTTACAGGCAGTTCAAGTGCAGGGCCTAGGAGGTTCAAGCTGCCCAACcA

TGGAAATGGCACATTTGGTtCAAATATCGACAGCATTAACGAAGACTTCTTTTTGTTATC

TGACCATGAATAGACCAcACCATACCAAGTTTCACTCAGCTGCACAATAGCAGTCATTTt

CTCCGTTTTtAGACCTCCATGTAACAACACACAAAATGATGGGACTTtCCCGTCCTCTTC

ATTTTtGATCTTTtCTGGCCAACAACTGGAGGGTCATGGACTGGCAGAaCTAGGTGACGT

GACAgAAAGGGaGGgTTGgATATATCTTGCAAGTCAAGAAAGCCAATCACaTTGaGTgTt

gaGGGAACTTTTAAGCTGCTGAAAtctatgatctcactagggtgcttgtacatattcaaT

atGCTTCCCCAAGTGTTTTGAAATGATGGTGCTGGGTGAAGGGTTACCAATGATTTGAAG

TGTCCACATGTAATTACTCCCTCATACTTGCAGTAACGACGCTTTAGAAGGTAATCAATG

CacTGTGATACtGTTTtATAgTTTAGAGAGCCTTCAGGTTGCAAAAGTTCTCCAAGGTCA

TTGTTAAGCTGAATAATCTTATCAAAGCAGCTTTTGCTTccTTTTtGGCAATTGCTGGTT

TGGCGACAAAAGCAAAAt

>contig00708 length=422 numreads=4

caagttggtgatggaaaagaattaaaatccaggcatcgtttctCCAATACAAATCTccAT

CCTTCAACaaTCGTCCATTCCCAAACTCGcgacacaacaaatgaacagctgaggattact

atattgacgcatgttataaaccaactcgCCACCTTTACCGCAGAGCGGATGTTTATCCAG

TGGTGGAATTTGCGACCTTTtCATCACAGGCtGAAGAAAATCTCGAACGATTTTTGgAgA

AtCGGaGCTCAGCGTAGATGCACCAAGTAATCCGAAATAAATCGGTGAAACTccccGgAA

AATATTCCTGCTTGATAAGTTCatAaTCTAATGTTGCGCGGGTGGAAAACAAATGAACGG

CAACGAAGAAATCCACAAAAGTTGaatatatttaaaaacgtccggatataaagctcgcaa

ag

>contig00709 length=401 numreads=10

AAGCAGTGGTATCAACGCAGAGTACGCGGGCAGGAGAATCAAAGTTTGCTTCGTATGGCG

CGCTTGCCTGCTTTtACGAGCGCCATCTGCCAAGTGCTGAGTGAAAATTCATCGGCTCTG

ATCGAAACTCGTACTGAACTGTACATATGGGCGTATATTCTTcATCTAACTCGCCATTTG

AACAAAAaTCGCCCATTCGTCGACACgATTtCTTACGAcAAGTTGTTGAacTACGAGATT

CTGTTggatgctctgctgaagttaggaaagatggcgttcgacgtggtaagcactgacaag

aaggtttttgaattcgatgagtttttcagatcaactgaaattgcatcggcgctagtaacg

cctttcctcgttcatcgtcagcaatttcttcgcttctcatc

>contig00710 length=110 numreads=2

TTCCACCACTGCACGCACCATCCCTGCATTGTTGACCTTGTAGACATACGTCATtGTTGC

ACTCGCCTTGGATATTCTCACATCCGGTTGAAGGGgTTGCAAATCTGACA

>contig00711 length=206 numreads=1

cttcacttattttcggaacataaatcgacgttttcgtctgtttttccagctctcgtcttt

tcctcgcttccttgccaactatgtggaccgaaaaaaaccgggaacatccgccagacggac

gcagtatccgctttcggtcttctccacatcaacaagctctgcggcgcatgcgtcagcctc

ttcaaagtagccacaatcttcttcca

>contig00712 length=183 numreads=1

ttatgtaaacaaaattggaataaaagggtcttttatacaatcttcgtaagagattattac

tacttgcataaaactgatggtctgagcaacggcagtaccagcctctcatggtacagctac

gaccttcgataaaactactgaccaggtagcagcatgtttaaggttagatagtccgaaaac

cgt

>contig00713 length=268 numreads=4

ttCGAACAGAAACCAATCCATAGCCAGAACTGAAATGCATCGACAGACTTGTCTCAGTCG

TCATTTTGTTTAAGTCGCCAACATAGACTACTGAACTAAAGTCTTTGTCGCCAACCATGT

CAGCTTGAGGTATCGTTCCCTTTCCTGCTACTCCTATGGTcTgAaTTGGTGTAGCTAAaC

tCATtGACGTTAATtCATTAGTCAAACTTTtAGCCATTttATTTTttaTTTTTtttCtcc

cGCGTACTCTGCGTTGATACCaCTgCTt

>contig00714 length=1115 numreads=41

ttgctgaaggtgtttttggtttcctcgctcggaagactgacttttttcatggggctgcac

ctggggttccaagacagattggacacAATAAAAaTCATATGAAGAACAAGCATCGgAGAA

ACATAAGAAGGCTGAGCTGGCAAAGAAAGAAAGGGAACAGAAACTAAAAGAGCAAAGGCA

GAAGGAGAAaGAAAAGGAGGaGGAAGCCTTTCGACAAAATGTGCAAGCTGGTCCACGCAT

CGAGGAGGTtACTGACGAGGAAGCGCAGAGAATTATAGAGgAGAGCAAGCAGGTGGAGGT

CGTGAATGCGGAGCCAGAAAaGACTGATGACAAGAAGAAGGACAAGAAGAAGGACTCCGA

TGATGAAGATGAAGCGGATAAAGGTAAAATGAAACCGAATGCGGGAAACGGCGCTGATCT

GGAAAAGTATCGTTGGGTGCAAACGCTGTCAGACATTGACCTCTATGTCCCGACAGGCGT

tAGTATGCCCTTGAAGTCGAAAGATATTATTGTtGAGTTCACTCAGACCCATCTGAAGGT

CGCCTTAAaGGgACACCCTCCGATTATTGATGGGGATCTCCAGAAGAAGGCCAAGGTAGA

GGAGTGCTACTGGACTTTGGAGGACAAAAGGcTGATCCAtATTTTtATCGAAAAGGTCAA

CAAGATGGAGTGGTGGGACAAGCTAGTCGTGACTGACCCCGAGATCAACACAAAGAAaGT

CCAGCCAGAAAACTCAAAGTTGGGTGACCTGGATGGCGAGACGAGAAGCATGGTGGAGAA

GATGATGTACGACCAAAGACAGAAGGAGATGGgTCTGCCTAcGTCGGACGAGGCGAAGAA

ACACGATaTGCTCAAGAAATTTATGGCGCAGCACCCGGAGATGGATTTCTccAAGGcAAA

AATTTCGTtAGAGTATCACGAACTCTTGAGTTCCtGttGAAGACGTTCcGATTGaGAATG

ATGGCATCCAGCCTGAATGGGTATCATGTTATGGATCGTGCCGCATGATCGAGGAATTCG

ATCGAGTGTGATGCATAAGCCCGGATTGGCCTTCATTAAATGTTTGTGTGTATGCTTTTG

AAGTGTTTTCTACGAAAAGAGTTTAGGTTCCTCGT

>contig00715 length=1246 numreads=47

AAGCAGTGGTATCAACGCAGAGTACgCGGGgCTTTCCCACTGGAGCAACTAAGCTCGAAG

GTCGTGGCTCACCGCTGTTCCTTCGTGCACGAGTGTAAGAAGgcACAAGTCGGCCACAAC

AATCATGTCTGCATACAaGGAAAaGGGAGGCCAAGAAGAGGAGgCACAGGTTCATCGCAT

TCGGATAACACTTACAAGCCGTGACGTAAAAaGTCTAGAAAAGGTaTGTGCTGAGCTAAT

TCGTGGTGCTAAAGACAaGAaaCTGATGGTTAAAGGTCcTGTTCGCCTCCCCaCCAAGAC

tCTCCGCATcAccACCCGGAAGAcGCCGTGCGGTGAGGGTTCAAaGACCTGGGACCGATA

CGAAATGCGCATTCACAAGCGTCTCATTGATCTTCACAGTCCTGCAGAAGTTGTGAAGCA

GATCACCTCCGTGAGAATTGAGCCGGGggTGGAGGTTGAAGTCACGATAGCTGACGCATG

aGTGCTTTtGCATAGATCTCTCTAAGGACGCGGATGAAAGAaACCTTCAGTTGATAAaGC

TCTCGTGAAAAAGACTACTCTTTTtCcCAGAGGCCcGAGTGTCTGAATCGAATGGAGAGC

TATGCATGTAaTATACATGTGATTTCAGAAGGAAAAAAAcCAGgTTGAAACTCAGAAGTC

AACTCGTtGGAAGgCAGAATATATCCTCTGATACGGCAaGGTCTGTGCCAGGCTTtAtgT

CTgTAGCTCtAGATACtGTtttttttCTCAAaGCAAGCCTCcTTGGAGGGTTgAAttGCT

GGCaGCGAAAATGAAGCCAAGGAAGTATGATTTtGGTAATTGCGTGTTCATATGGCATAA

TTGTAGAGAGTGCACCCGATCTTCGTCGATCTCACTATGCATCGAGGAGTCAGTATGGAT

TGACAATGAAAGGgAGTTGTTGTGTTCGTATTGAGGGTTGTTTGTGCGAATGTTATCTGT

GGCCCTGTGAGATCGCAAaGTaGCGAAGGAaGgTTGAaGAATTTAGCTGAaGAAAAAGAA

aCGTaGCTGCTCATGTAGAGGTTGCTAGAATCATTATAGTTGTTGTTATTAGTTGTTTGt

GCGTTATTGTTATTTGTTTGTtATtgTtATTTGTTTGTTATTGTtAGCGTTAATATCCcT

CATCAGACAGACCAATTTTTAAAtGATGGaTTcAGTCGcatatcaggaagtggtacttgg

tcttcctactgatgaaatctatcaaccggtagagttgtgtgcacgg

>contig00716 length=427 numreads=7

TGAaGCCGAGAACATGGCGATTATCGATCGTCAaGATGGTGTACGGCTGCTGTGGTCTCT

TTTAAAaTCACcTAATCCAGAGGTTCAAGCAAGCgCTGCATGGgCGATACGCCCcTGCAT

CGAGAATGCAAAAGATGCAGGAGAGATGGTCCGCTCTTTCGTCGGTGGCTTGGAGTTAAt

CGTCTCGCTGCTGAAGTCAACCGaGaCtGAGgTTTTAGCAAGTGTTTGtGctGCTAtcGC

CAACATTGCCAAAGATGAaGAAAATCTGGCCGTTATCACTGATCACGGTGTTGTACCGAT

GCTTGCGCGTCtCACAGAAaCGaTAGaCGACCGACTTCGCCACCACCTtGCGGAGGCgAT

AgCCAGATGCTGCGCATGGGGGAACAATCGAGTTGCTTTCGGCAAGGCAGGGGCTGTGCC

TCCTCTa

>contig00717 length=755 numreads=22

gccggatgccgaaacgatgattccAAAAaCAATGAAAACCACAGGGCAACAaGAaCGTAG

TGGTACAAGACATTTGGTGAATATtCGCAAATGAACTTGTGTCTGTTGCTAATCGTATGT

CTGGCATCGCGAGCCCcTTCTAATACCTCGCAGAGTCCGAAAGTTGGTAGCATAAGTTCG

CCTGCTTTCGACTGGgCCCGGTCGTGTATATAATCATACATAATGTCATCTGGCAAAAGA

GCCCAATCGATCCACTGCTTGCCATATgCCTTGAAGTTACcgTTAAGGgTCTTATCGATC

AGGAGAAAGGCTATCACGTTTGCGATTATGTAGCAGAGCTTCaCCAAAACaTTTAtCAGA

ATTCTGATACGCATGCGCGTGGGTGAATTGATTTtGtAGTTAAAGAAATTATTGGCGATG

TTCTCGTCGTTGCTGTCAGCTAtGGCCTtGCGAAGACTTATCAAaTCGgtGTTGaCTatC

TTGtAGACcATATAaGgAATGTAGAATAAtAATCCAaGCGCAGCTAGAAAAaTGGATACC

ACTGGTAATGAAGGAAGAAGGTCTTCGTCATCGAGGTGCAGgCTGCATCAGCTTtGCCTG

CAGGCGTGGTTGTtGAGCACAAGCTTCCGTCGGCATAAACGCCGTCATTGCCGATGTCGT

TTGGGATACCATAATAGCCGAGGTTGTTCTTCTGATGGCGGATTTCGCGATATACGTAGA

AGCCGTTTATCCAGCAAGCTTCAGCTATGAATCCG

>contig00718 length=606 numreads=14

TtCaTtGACATGCcTGATGAAaTtAGCATGCCAATTGTAATAGGAACCAAGGTTACTGCC

AAaGTTCACTCTAAAGGAAATGGTTTGCTGTTATTTACGGGGACTGTTGATGCTGTTGAC

ACTTTAATTAATCACTACCGTGTTGTATTtGACAGGGAAGGTGTTGGAACACAATCAATA

CCAGATGTTGATGTGGCTAGCGCGCTGTACGAGACAATGAACTGGCAGTTATTTATCGCG

AAAGAGCgACCGAaGATCGCTGGGCcAACCTTGAGTCCAaCGAaTTCAACGATaagccct

gtgctgatgctgtctgacgctgccatgcccggttcatcgactcctgcaCGTGTTCATCTC

AAaGGATGTTCTTAGTTGCGATCTAAGtgccacctacggcggctttcccattaagtttct

cttgcttattgtcaagctatctaaaattctgaagcataagcgtaaattagttgaccagat

aaccgatatgaactcagtagttgagcggagacgTTCTTTTCGTCAACTGATCGGTCCCGG

TTTCCAGAAGAAGTATGCGTCTGTTGTGCTAGACCTTGAGAAGCTGAACCTAACAATGCA

ATCTAC

>contig00719 length=238 numreads=1

aagaaacggcgtaccaaatgtattgaatgtttcctttctgaaaatgtgcgacaggcacca

aaatctgattcgcggattcctcaagctcatcggcaggaatctcctcaatcctataaattt

tctgcgcctgagtgaccaccatatcaagagcttggtcttctggcaccacacggtggatct

tgctacctacaatctcaagcaaacgcagctttttcgactccatcgacttgtcgatgtg

>contig00720 length=233 numreads=1

aagcagtggtatcaacgcagagtacgcgggacggataagtctcttgtttattatcttttg

agagactaagataagcagacatggcagatggatttgatttcaaggctcttactgctgacc

tcaacaaaagcttccaatcaggcaaaacactttcgtatgaataccgcatccagcagttgc

agcagtttggtctcaagattgatgaaaatgaagatgccatatatgaagctctt

>contig00721 length=240 numreads=2

gTCTGGCTCTCCAGACCCAGTCGCGTTCTTCGTCAAGTGCTTCCCGTGTGGAAAACCAAa

GCCGTCGTAGTGGCAGACATTGcAGTTTCgtGCTAGGGGcgaaacggaaacgcgaagtta

cggaggcattctccgacgtttgccgacacagcgtgtctaggaagtgttcattgtgttctc

tgcactgggtctcatcgagtttgctcaattcgacgtttttcgacaaagcgaaaaagtcaa

>contig00722 length=1641 numreads=65

AaGCAGTGGTATCAACGCAGAGTACGcGGGGCCTCGTGgTTTcTTTTCACCcTAGCAAGG

TGTGTCGATTGTGTAGCTTtGCGGGTCTtAAATATATTCATtCCTGGACTTCAACTGACC

ATGCCAGAACAAAGCAAAAGTTCGCACAATGCATACAAAGACaGAGAAaaGCCGACACAT

GTACGGGAGAGTAAcATTATGGcTGCGAAAGGTGTTGCaGAaGCTAtCCGGaCAaGTCTG

GgTCCcAAAGgCATGGACAAAATGATTAAGGCTGCCAATGGAAATGTTACTATTACTAAT

GATGGTgCCACAaTATTGAAGCAAatGCAAGTTCTTCAtCcAGTGGCTAAAATGCTTGTA

GAGCTAtCAAAGGCACAAGACGTAGAAGcAGGAGATGGAaCCACCTCTGTtGTTGTGATT

GCAGGTAGTTTGCTATCtGCTGCCCAAAGATTACTTGAAAAAGGAAtCcACCCTACGATT

ATATCGGAGGCATTTCAGAAGGCAGCTGCTAAAGCTTGTGAGTTTTTGAAGGACATGTCA

ACTCCCGTCGATCTGTCTGACCGTGAATCTTTAATAAAAaGTGCTACGACCTCTCTCAGT

TCGAAGGTTGTATCCCAGTACTCAAACATTCTAGCGCCGCTCAGTGTTGATGCAGTACTA

AAGATAATTGACCCTACGAAaGATAAATCGGTGGACTTGCGAGATATAAAAATTGTCACA

AAACTTGGGGGAACTATTGAAGACACAGAGcTGgTCGATGGATTaGTTCTTGACCATCGC

ATCTCTCACTtCGCtGAAGGTGTCaGCcGTGTAGAGAAGGCcAaGATTGGTCTAATTCAA

TTTTGCATCTCGCCTCCCAAGACGGATATGGAGAATAACGTTATTGTTAGTGACTACACg

CAAATGGACAGAGTGCTAaGGGAGGAGAGgCAGTACATCCTGGAAATTtGTAAGAAGATT

AAGAAAGCAGGTTGCAATGTGCTGCTTATTCAGAAGTCGATTCTGAGGGATGCTATCAGT

GACATGGGTCTGCATTtCTTAGGAAAGATGAAGATCATGGTTGTACGTGACATCGAGCGG

GACGAAAtCGACTTCGTTTGCAAGTCACTTAGCTGCCGCCCGATTGCGAGCTTGGACCAC

TTCCTCCCGGAGATGCTTGGTTCAGCGGACCTCGTAGAAGAGTTCTCTACTGGCGCGGCA

AAGGTCATCAAGATAACAGGAGTACAGAACCCCGGAAAGACTGTGAGCATACTGGTGCGG

GGCTCGAACAAGCTCGTTATTGAGGAGGCGGAACGATCCATTCACGACGCCCTTTGCGTC

ATTCGATGTCTGGTCAAGCAACGAGCATTAATTGCTGGTGGCGGTGCTCCGGAAATAGAA

ACAAGCATACGACTTGCTGATTATGCCAAGACCCTGATTGGGAAGGAGGCATTTTGCGTG

AAATCTTTCGCGGAAGCGCTGGAGATCATTCCGTACACTTTGGCAGAGAACGCTGGTCTT

CATCCTATCTCTATTGTGACTGAGCTCCGTAACAGGCACGTTAATGGCGAGAAGTACGCA

GGCATCAATGTTCGCAAGTCCGCTGTCAGCAACATCTTAGAAGAAAACGTGTTGCAACCg

cTGCTCGTCTCGACGAGTGCT

>contig00723 length=270 numreads=4

TTTtGTTTtGTATATTGTGCTTATGCGGGAAAggCGGTTtGAATCAACAACCCTATGCGC

TAGTCGATGAAAACCCTTATtCTTAGCTCCCACTGTcAGCTTCATGTCCTTTTCGgTGCC

TATTTtCTCTTTCTGGCTGCCGgAAaTATTGGTTCTTCtCGTTCAGTTtGTTTGaTTAGT

GTaGAAAAAGCGGTGATAGTAAGCATGAGCACGGCGTtAATGTCGAtCGTTAcGAgTTTC

TGGCTTTGATAGAAAGCGGCATTGCACGGG

>contig00724 length=241 numreads=1

gtatatgtcagtccatcccccaagaagttcagagctgtaatggtaaagcctgttgctgta

ttgccttccgctgggtcgatcatcaaggtaccggctattggcttgggtggtatggtaact

tcaacatctgccactgaacttgcaaattcattagaagcagtcagctgaaatacgtatggc

acttttgcgttcagagcttcgcccttcaacttcaatttcattacaccaccaaaattcttg

t

>contig00725 length=236 numreads=1

aagcagtggtatcaacgcagagtcaggactagtttccacatctttgaaggaagtcacttc

agaaacggccgaggtaccagcatcagccatggtcgtgatcttaggcgcaccggtgctggc

ttccaatgcagaagttgttaaatccactacagccatagtctccgcagcagccgctgttgc

tacctcggcgggactggtctcagcagaagacgctatggtaatttggttgctgcggt

>contig00726 length=186 numreads=1

agcccttcaaggttccactttaaaagatgacgttcgtcagtagtgggagaacgcaaattc

gtcggggagctcgccaattataacccaacaccgaacgtagaaggacgccgtcaagcagag

ctgggcatacgaccacgagatgccaatctttaaaatcttttcactgtgtttcttaaattc

agggaa

>contig00727 length=405 numreads=3

cTTGACGTCGGGTAAGGATGCCATctCAAGCTGTGGGAACTGTCGACAGGGCGACCGCTT

GTTGtataccgaggagctgtcatccaccagaacaaccacgctgctgccatattcaaccac

accgaggactttgtcttattcccggatgagaaaaacatggcgatcGCCTGTTGGGATAGC

CGTACCGGCGAAAAGCTCCcTTCCCTGTCATCAGGTCACAACAATGCCATCCGCCATCTT

GTACACtctccttgctcgcccgccttcctgtcgtgcagcgaagattaccgctctcgattc

tggtactgcaagggcagttaggctgttggccgctcgcaccgatgtgaagagtttgctcta

cgatttgatacgagtctcgcgttctttgtaccctgaccatggagt

>contig00728 length=159 numreads=1

gtagaggctttgctctgaggtgacggaggagacgagggatttttgtgagagacgagaaat

gcaagctactttcgcgctcatcgtggtcacgctgaccctggcttcagcgaagtccatacc

acgtaattcgaactgggaaggagaaaagaatacgacgcc

>contig00729 length=525 numreads=4

CTccctGTTtCgAGCTGACGGCGgTCAAACtGGCAGCAGCAgtAgCcATGgCATAGgCGT

GGCCGGAaTCAGCCACCGACATcGCcAagtCGTTCGCTtgCaCGTTAATCAGcGtCTTGa

GTcGCTcGgcGTTGTTGAaCAtCGgTtCGTTgAACAcGTCCtGCCaTAAGgAcAGCATGT

TGGGAATATTTCGATCCaggcaatacgaagagaactccacgcccagttcgaactgcacag

ggtccgtgTGGTCGGGGGACATGTGAGGACCAACCCCTAGACCGCCAGTGAAAAgttcca

cttgctgagcgagcacacggtggtccatacctccagctcccaatttggtaagcacgttgc

aaaatagcggaaggtagggcttcagcacgtaaggtatggttgacgtggtcacgatcgaac

ggaagtaggtgattccattggtaggttccttgcacgtttggacgttaaccccctcaaggc

aaactgttcactgacagtttcttttaagtcctattgatgtctgcc

>contig00730 length=236 numreads=3

AAGCAGTGGTATCAaCGCAgAgTAGCGGgCAtAGtGGgAAACGGtCTTACcAGGTAGAtA

CTCAACATcAaCTTGCACCTCAGaTGTTtGATCACtGTTTGCAAATGTCAGCGTCAGGGA

TGAGCTGCGGGTCGTAGCATATGCCAAGACAAACTTGACGGCGTGAGCAGTCTTGCTTGC

GGTgcttttgtccgtgtagatgacgctgtagccgtattggtaggttctgcccaagg

>contig00731 length=238 numreads=2

ggATAGTAAAAGCTTCCGGGTCAAGAATATGCACATCTTTGCATTCACTCCCAATCACAA

GGCAGCTGATGGCATCGTCTTCAGCGAGGCTTTTTTtCAAGGTCGAAAGACACgtGATCA

CGGTCTGCTTTCTTAGAGGAGCATGCttgtgaactgcagcaaaagattcgatttcttctg

gatccagttgaaggaatttcaaagagcgtacagtcaagactgaagccccatgttcatg

>contig00732 length=244 numreads=5

cTGTGCTAGACTAAGCAGAGCATTAGCTGCCTCTTTAGCATTTtCATGCTCAAGCCTCTT

TTTCTTTGTAGCCTGACGCTTCTCAAACTTGCCTACTAGCTGGATGAGGTGTCGCTTTTT

GGGGGACGAGACGTGTTtGAATAAAGTAGGCACGTAATCTGGGCTCAGTTTATCATCAGA

CTTTGCCCCTGACAAGAAGTGCCTACTGCACAGGCGAcTGAATATtGTCcGgAAccccaa

ttcc

>contig00733 length=242 numreads=2

atCCAGTGTCTTGGCGAGAATCTCAGGCTTAGGCAGCAAGTTATTGCGACAGCTACCgtG

TACTTTAAGCGCTTCTATGCTCGGGCTTCCTTTAAATCAGTTGACCCACTTCTAATGGCA

CCTACATGTTTGTTTGTTGCTTCAAAAGTCGAGGAATCAGGACCCATGTCAAACAGTCGC

TTAaTTGAGCTCGTGTTCAAATGTTTGCAAAAGCAAGTTTAGCTTTGCTTACCCTGCCGA

AT

>contig00734 length=1310 numreads=44

CAAATAATCTGCTGAATTtATCTCCTTCAaGGCCAAAaCATTGATTCATGTAGTTCACAG

CAGCTTCATAtGTTTTATGAAGCTCTTCAaTCTTTCTcTTGTCtAACGtCCAATCAGTTA

CCCTGCGCCGATGATAGTCGCAGTAGCATATCCAAAGCTGCAAAAAATCACTAGCGCCAG

AAAAGCCACCTTTtAACGCTTGATCGAAGACATCTTTCACTCTTTCGTAGGTAGCACCGC

TTCTCTCCAGAGCCTTtAGAAAGTTTTGCCaCaGTTGCACCACCCATGGACAATTACGAA

CTGCTCTTTCGTGGACAGGCAAAGCAACGGCTGATATCTTGAGTTtAAAGTCCATATAGT

TGGTGTATTTTATCCACAAATCATATTGCAGAGGATTGTCCTTGATGgCTCTCTCAAACA

TGCATTGAAtACGTCCAGGGTCACCTCcTTTCATcTcAtATtCGATATATTCCAAATAcT

CTGGCAATTTACTTCCAaGCAGCAGCGTGCAGCTTCttcTCAAACTCCATGCAAGCCAGc

aTTTTCTGACTGCCTCGCTCATACGATCTTGTAaCCTCTtCTGGAaTTGGGTGACTTGAC

cAGTCTGCGTATTCCTGGTATGTGCTTGACATGCCAAGAaGTGgAACAGCCAGTTGCcTC

TTGAAAATaGCATCAATTCGCTCAATTTGCACATTGACCTTCACATCCATCCCAACTGTA

TCACCAGAATCTTGAAGTTGCTTATAAGAATCAAGGATTGCCAATTCAAACTCACGATAT

CCATCCCAGATAGACGCTCCCTCCGTAATATGTAATCCTACAGATGTGATAGCACGTTCA

AAAACATTTCGAGCAGTGATTATGTCcGATGGGgATTCAATGTTGTTCATAACGAATTGA

CAGTACTCATGCCAGATCTTAATTGAAGTATAGTCATTTACTGCTGCCTCAAaCTTtCCc

ATcAAAAATTCTTtGTgCTcTGGAATAaCGgCCAaCGGAaGTTCATCTTGAAACCATTCT

AACCAAAgctcttccgatagtggaTAAATTGAAGCCATCTTTTCACGTGCTTCAcGCAAT

GGTTCCAAATCACCAGTAACGCGCAAGGCCTtGATAAGTTCCACATGCTTATCGTACTGg

TAAGGGCTAGCTTCCACCTCTAGCGTGAGCATTtGTATATTCGCTTTATTTtCTTTGTCA

TCTCCCGAGTCACTCTCATCCGTGGAACTATTGCTGTCACAATCAACTTCCAGATCTGCC

ATCTTGCTTCAGCATGTGCCCCcGcGTACTCTGCGTtGATACCACTGCTT

>contig00735 length=432 numreads=9

CGAGACTcTAGATTCATCATCAGCATGGACTGAAGTTGCTTCTTAGCACGCgCAaCCTCG

TCCTCcGCGAAAGGCTCCGTCACCAACGTCAAGAaCTCCTGGCTAACcaCTTTCGCTACA

TCTCgAGATtGACTTGGGTGAGCGCTTGCGTgAATGCAAAaCAAGCCGGAATCAGCATAA

GCGTGATTGAAGgCcGTAGCTGAGTACATCCAGTGATGCCGGTTCAGGACaTTCGTGTAC

AAACGAGAATACATGcccttgccaggaccgcccgcagagaacgagcctccgccgcctagA

AGCATGTTCAGAACTGCAAAGCTGAACATGTccggctcgcagtagcttgaactttcaaaa

CCGATGGCGACGTGCACAAGCTCCGGAAGCTGCGTGAGGCCCGGTTGCAATCGCGGCttG

GACCGGTGCTCC

>contig00736 length=161 numreads=2

aagcagtggtatcaacgcagagtacgcgggtttgAAGAAGTTAAAGGAAAACCAAGAGAA

GACGATCGGATCGGtAaTGTCCACAAATGAATCTCTgaGGTTAAGGGCCGAaGgAAGTGA

TATCTAGTCGCGAtacaaacttcaaactgcttgaagaggag

>contig00737 length=242 numreads=1

aagcagtggtatcaacgcagagtacgcggggcaaaagaagacttacaagggcattctcgc

caaggagcttgcgacgtactctgctgaaggtgtcctgaaaaagacccgattggtgaacat

cttgatgcagttgaggaagtgctgcaatcatccttacatatttcccggcatagaagagga

gccgttccaaaataggagaacacttagtcgaggccagtgggaagttattgctgcttgata

gg

>contig00738 length=173 numreads=3

tGGGAAAGttACGGTGGtGcGCcGGaCGCTGgTGAGCATTGTaGCGgATTGgCGGCACAG

ACagAGGTAAATGcTCTGTgTGctACTTCCTCCACTCTGACGCCGGATACTGGAACAACA

CTGTCATCTTCATCTTGCACGAAAGTCCTTTCCTCTGTCAATGACTCCGTTgg

>contig00739 length=226 numreads=3

aTAGCGCTTCCTGAACTGATCGTAGTTCTTGACGaTGAAGTCATAGACGCGCcTCCGTGC

GTtGACACGcAcAaGAtGCGTCAaCATCGGAATCGTGTCTTGTATCCTCATCATCTTCAA

ATCCAGCGAGTTATTTAAAAGCTGATCGACTTTTTCTTTTCGCTTGAACTGAGAGAATGC

CTTCATGATAAGTTTCCGATCGGAGTCGACCTCGCTCTTTGCATAc

>contig00740 length=656 numreads=23

GATACATTCCCCATGTCCGTGGAGCCCAAGGACAAATCTTTATCCACCGGATTCATGAAA

TTTACACCCACCATCTCGGCGTTTGATGCAAATAGgTTGGCCAAAATGGAATTCGTTAaT

AAAGATTGTAATACTtCGTCGGTCTCACGAaTTTCAACGCAGCATCcTGTAGCATCAGCA

GCTGCATTTGCACATTTCTCAAGTTTCGCTTTCAACTGACTCACGTCCTCgTCcGTCAAT

CcGCgAACgTAGAAATTCAATTCCGATCgCcTCGGGATAATGTTGGGCTTTTCACCACCG

TCAACGATGATTCCGTGCACGCGACAAGAAGGCTTCATCTGCTGTCGCaTCATGCTCACA

CTCGtATAGCATGCCACAGcTGCATCCAAaGCGTTAATCCCTTCCCAAGGCGCAGCTGCT

GCATGCGATGCCTTACCATGAAATATAATGCTGAGTTGAGTGATAGCCAGGCAAGTAGGA

AAAAGTATGTTAAATGGACTaGggTGAACCATCATGCACACATCACAGTCATCAAAAGAA

CCTAGGCTAATCATCTTTATCTTGCcACCACCACCCTCTTCCGCTGgtgTTCCAAtACAa

CAACTTCTCCCTTtAGTTGCGgAAAaGCCAacAATGCAGCTTTTaGCCCAATGCAg

>contig00741 length=243 numreads=3

AAGCAGTGGTATCAACGCAGAGTACGCGGGGAaGGtGCTACAACaGATCAAGATTGTCTT

TCGTTGAaTAGCAACAAGAAaTCcAaTGAaTCcAaTtATtCTGCaTGAAGAATCAaCGAG

CGGTTCAGACAGtCTTTttGAAGCTGCATGCTTAGTAATGAgCACATcATAGTAAtAgAa

CcaTGCtATTTGGCGcTTAACAATAAGAGCAgacttcaaacccctggcacaagggttcaa

acg

>contig00742 length=273 numreads=3

TACGGCTTGATGAAAACCACTGTGGTTTTCATtCAAGCTGTATTaTTATTATtACCAAGT

ACAAACtCACTTTGgaGTTtGTAGCcTTGAGAGTGgATtCCTTGAGGTGTGGACTCAGAA

AGATCTATTTATTCAGTGTATAGAGTTTAATGAAAGTTTGCGGAGTGCGATTTGTTCCAA

GGCTGCATGAATTAATGTAAGTGCTAAtcaaaaaccttcagtttactatgtataattacc

agagatctgttgcggtaaaagggagtaaaaggg

>contig00743 length=1617 numreads=42

ttAATCAGATTGATATATTCTATGTAATtGCCTCCACCAACCaCGAACACAATTGCcTCC

TGAAAATTCGTCTTGTTTCGCTGCGAGGCCGGCGCCTCGTTTCCACGTACCATCTTCGGg

TCAAAATACCGGTAaTCTTCAATCgCCTGCGCGTTCTtGTTTTCCATAACGGCGTCCACA

ACTCGCGTTGCCGGTAGATtCTtCGTTCCGACCACCAAGCTTTTCACGCCTTCCATcACG

AACTGCGAACCAGTGCTCATTATCCGATTAAaCATGGCGCTGTACGTTGACTGCCCGCTG

CCCGTCTGGATGGGGCCAGCCGCCACcTTAGCGAATGCCTTCCACTTCTTGACGTAATTC

AGAGCcGTCGTATCGCAGCCAGCTGCTTGCAaGgCTTCCAAATACTGGTCAAACTCCACT

GAAGACAAAGCAGGCGTGGACAAATAAAAAaTTATGAAGAGGCGAaCTTTATCTTCCGGA

GAACCAGCTTCGGGATCCTGGAGAATTTCCATCACAGACTTGTCTATGGAAGACTTGCTC

ATGATCTTTTCTTCGGTCTCGAAaTAAATATCcAATTTCCGGGCCTtGATGTGCTCAAGC

ATTGCGgTAGCAATGTtCATGTGCATGTCAATCAACCTTTTCTtCTCGAGTAaTtCAGGA

AGCGAGCTGACAGCAGAAGTCAGCTTGGAAGTGCTATCAGACCACaTCTGTTCAATAAaT

tCATCATcTtcGTTTtgAACTCCcATAATATTCTTCAaTTtCTTTACTtCTTCTtCCGAA

GCTCGATATtCaTCTAACtCcTTTTGAaTTGCCTCGGCGACAGTCGGGAAAGGGCTTCCC

TTATGTTGCTCCCAAAACTTATCCTTAACTCCGAGGTCATATTTCTtGGGCTGCGGTTTC

GCTGAGCGGTCCTGCGCCGTtGTCGTATcTGCCTGTGCGGGTTCCcGAACGGTGACGCGG

TTTAGTTGcAAATCGAAGACGTCATGACACAAAGCTTGATaaGTCCAAGtATGATGCAAT

GGTGTACACAAGTCGATATTTCTGTCcAGTATTATGAGAACTGGCCTCTGAAaGCTGAAa

TtGCCTGACTGAATATCGGACGTGAACAGAGAaTTTCtCGCATCTCGTAGATTCTCCCTA

AGTTTCTTGTCTAATGCCTCAGCAACcATTTCAGCCGCATTTCCTCTGGGACATCTGATA

ACAGGAACAGTGCCTAGAGTGACCAaTGCAGAAAAgaGGCTGTCAACCAGACTATCgCAA

ATGTGCTCCATatctgtgtccttcaaatcgggcccaTTTATTGCATAATATGATATGTtC

ATCCCTGTTACTATGCttCaCTaTgAAAAgATCATCTTCCAAgCATATATAGTTCAGGTA

CTGATCTACAACCTTTGTGATAAGTTCTACATtGTTGAACTGAAGAGCCGCTGTAGCCAG

ATCCTCCAAaCGATGCCTTGTTATCGGTGAAATAAAGTTTAAATAGTATTTGTCGTACAA

GCCACTGCGAAAGTCCTGACAAATTCGCTGTATGTTTTCATCACTTGgACAGACAAAATA

AACTGCAGGCACATCAGGAATAGTATCCCtGTCTGAaTGCAGTTGCAGATGAAGAGt

>contig00744 length=234 numreads=2

TTGACAAATCTGGGCATGTCGAAGGTTcATTCGGATCGATGCCATCTGTGACAGAGTCTT

TGAAATCCGATCACGCATCCCCTGAAGAATGCGaggactacgaaattgtcgacaggggct

atgaaatcgatggcaagggctacgaagttgatgaaaagcatgacgagtggtctaatgcgc

cagagacaccgcaccctgtttgaaagcaatttttcccagcactagtccgtttag

>contig00745 length=410 numreads=8

TGTGATCCCAGGTaCGCAACTCGGACAGAGgAGTGAGCCTTTCGAAaCTCAGGGTAGAAg

CTTGCGAAATACAGCATGAAGCAGATCACCATtAGCCACTCCGAAAACGTACTTACcAAA

TGAGcGGcATAGCCCCcATCTTCCGcTgaCcACtCCAACCGGGTGTGGTTATTCTTCGAT

GTAAAatGCACATTGCCAAtAGCAGcGCCCGCcAAaGTTGAGATGAaGAATGCTGTtGAt

CcAAGAGCAaGAATAGcTcGtATCCAAACGATGCGAAACCTAGTGAAGCCAgtAGAAAtC

GCgaGTTtCGATAgATAACTGaCAATCcAGCAaTaCAAaTTaCcAcATCCAAAaCaCATG

gtcGCTCCCActAtGTgAACGGAGATCACATTAgtGACCTGAAAGTTGcc

>contig00746 length=224 numreads=3

AAGCAGTGGTATCAACGCAGAGTCGtACACTTGATATtCGTTGCAGTCTTTAGCACTACG

GAGACACACTTCCATACAGGAGGTGTGGTTGaCGAATtGCATGGCGGCGCTATtGCCaCT

aGCCAGAGATACAACGTGCATGCTCACCaGCTGGaTcGACGTTTcAATCGGGACgCaTCT

cATGAGTGCTGGGTCgTtGTTCGCACCACGACATCGGTGCAACT

>contig00747 length=925 numreads=34

GGCAaTCTGTACCATaGGCTCATCGCCAGTTTttGCCTGCTGAAGGTTAGAGCtGTtGAT

GTAGTCGATGATTtCCTTCAAATCCTGCATCATGTGCTTCAGTTGGCAATCAATACTTTC

AGCAAGCTTGTAGGTTTTttCCCTCTCGATGTCAGCATGCTGAGAATACTGCAGGGTTTC

TTGCAATTTAAGCTGTGTCTCTATAGGCACAAGAAGCTCCTCTAGTTCTTGCTGCTGCGA

AAGAATGAAGTCAAGTTCACGGTCTAGTCGCTGTTGTTCAGCTTTTACTCTAGCCACCTC

GTAGTGAAGTTCGGTAATTTGTtCTCCATTTTCCATTAACTGTCTATCCCAAATGTTtAC

CTGACTTGCCTGCCGTAAGAATGCATTTTCTTGTTCAGTCAAGTCTTGGAGCCATCTGCc

AACAACATCTTCAAGCTCCTTGTACGACATTGGTTTAGTAGCAGGAGCAGCTACGGACAC

AGATGAAGTCACAGGAGCCAAAaCAGATGATGCTGGGACGGACATGCTTGCAACAACTGT

AGATTCACTTGAAATAGCAGAAGCAGGTGGCAAAGAAGTACCACTTAACTTGAAGGCAGT

ACTgACCGGGGCCAATAGTGATGCACCACTGGTGGATAGCGTAGCTATAGCGGATCCAGG

TGCagAGgCTGCTTgTCCAaTACCAAaTGAAAaCCcAGGCTTTGCTGCAATGGTCGaGCC

TCcTAAAGAAAAACTTGGGGCTGACAAAGACGTAGCCGTAGgTGCAAaTACTGTAGgTGC

TGTaGAAGAAATAGCTGAAGAcACCTgTGGCACAACAGCAGCACTTGATAcGGCAAAaGG

aaGCCCcTGCTGAcTaCcAaTGCCTATTGACCCTAGAGAAAAACCTgATGATGgCTGCAT

AGTCAAAGATCcACCAcTTGACaaa

>contig00748 length=396 numreads=6

gacatgatgtttatggcggtctgcTTCACAATGACGTAGAATACCAAGCCTtAAGCTATA

GACATCAGCTGAaTCTACTGTACCTCCCATCCcAAACCTCTGGGGCCATAAAAaaTTTtG

tACCAgCGAATGAGCCCATGTACGTCGAAtACtGTTGATCTGTCGCAGCCGGGTtCTGAA

GAGCATAAAATTCCCGAGCCATtCCAAAGTCGGCAACTTTTAGtATACCATtATAGGTCA

GCAAAATGTTGTCGGGCTTtAAGTCCCTATGGACTaTATTTTTGCCATGAAGaTACTCGA

TGGCGTAGGCCAACTGCATGCACCACCACTTCTTTGTtGTGACGTCAATTGGTTTGGACA

AGGCAtGgTTTAAAgCCCACCATGACACATCTCCAt

>contig00749 length=156 numreads=4

AAGCAGTGGTATCAACGCAGAGTCAaTGCCAACGgTATAGATCgAGTTAaCATAACCATC

AGCATTACAGtCGTCTTGCATGATACCgccattccccgtggcccatatgaacgacgaccc

tagaccattgcgtccaaattttacaccgtgttccag

>contig00750 length=240 numreads=2

aagcagtggtatcaacgcagagtacgtcgggattcaatttgtaaaaatacattttatatt

taaaaatgccattaattttcgaaacaggatcgaaaaggcgcattacatactatcacgatc

cagccgtttcttcttattattacggcTCgggCATCCTATGAAACCACATAGaaTAGCAAT

GGCTCACTCGCTAATAGTAAATTACGGCCTTTATGAAAaCATGGAAGTGGTTCGTCCAGT

>contig00751 length=508 numreads=4

atgatgatattccgacctattaagtccattatggcttatagagctactttcaaaatgttg

gatgggggcgcacaacagtcgatccttcagaaattggtttggatggtaggaaattggctc

ggaatcggcatagccctttATAAGTGTCATtCGATGGGGCTCCTGCcTACTTCGCCGTCG

GaCTGGCTTGCTTTCAAGGAGCACAGAACAAGAGTGGAATATGTgaTGGGTGGGCCAGTa

CTGtAATGTTtGGTAAAAaGCTGAGTCAACCCGGCTGCCGTTTGCTAGAAAGCAATGCGC

TTTCGGATTATCTGGTTCCGGACGTTGCATATTGACaatcggtttgggTAAtTCGGcGTA

AAAtGGTTGTGATCTGCATACGTAGTGTAAAACTTggacggatgcagcgaattgtgtgca

acatgctatacaagcagacatgttgttgacgagttttcatgttttacttgatgtctaatt

aaaggctgcaagctttgccttcccggct

>contig00752 length=614 numreads=16

tAAGTAACTCTTGTCGAGCAACCTCACAAGAAGGTGGACTTCAagTGAGGACTtCCTTTT

CTAGCGGCTAcACCTCCTCGTCGACaTCGGAATCTtGGgTGGAtGAcTCcGACGGAGGTG

TcTCCGgTGaTtcGTGTCGTGCCTTGCTCAGCCGTTGCCTGACCGCAGTGCCAATCGTCC

CACATGACAACCcGgCCTtCGTTGTCACAGTAGgAaCCATAGCACCAATTCAGTCCATCA

AATCCCCTTGAGATCTCtGCATTCGGGGGTAAAaGCGTTCGTTTtGGTAACAGCcGAAAG

GTGTAGTCGGCTGTTCCTCTGCTGTTGTGGgAACCGGGCTTGCCATGAGATCCTCGTCAC

CGTCTTCTCCTTGCTCACCAATATCGATACCTTTGCGTGCGGCACCGTCTTCACCCACTt

CGACAGTGCCGGCGTCACCTGATGGCGTTCTTGCGTCCGGTACCGTGGTTGGCGTCTCAC

TGGTCGTGATGCAGTTGAAGTTGTCAGCATTTAtGATGTTTCCATCGCCACCACAGAgAG

TCCACCAGCACCAGTTGGAGCTTGAGTCGTAGCCGTTTTCGATTTCCATTCCtggcggat

agaccctgccgtta

>contig00753 length=688 numreads=13

taatcactttcgagtaattctttactggcccaaagtctcacagaattaccccagcgttaa

aagatatccttccgacggatggcggcaaagggaGTACACTACCCCTTTATGCGCGTCCTT

CAATAcATAAGTCACCGATGCCTCAACTAAGTCCCAAAAAAaGATAGAGCCATCTTCCGA

GCCACTAATTATGTGACTGtCACAGTGTgaGAGGCAACTGTCAATCTTGTACTCCTTGTT

CACATGtCCCGTAAaTTTATTAAGAGCCGTTCCGgtATCCATGTCGaTCAACCGCAAtGT

GCTGTCCAGTGACGAtGACAGAAcACACTGATTGTCATTTGTAAAACACACACTCGTCAC

AGgTTCTCCTATACAATCCTCAGTCAGGCGACCATGTCGAATGTCATAAACACGCGTATA

TCCATCAacAgACCCagTAAGGATCTGACGATCTGTAACAAaTAAGCTGGGAATACTGTC

CTTAGCATCGTCTAAAATTTGAACTGGCTCCATGGCTCTAGAGCGACAATCCCAGCATCG

CACAGTAGCATCATAGGaGCCTGAcACCAGtATTGATGAATCAAccGGGCTGAACCGAAC

ACAGTTCACACGACTCAAATGGCCTCTATACTTCCTGAGGACTTTTCCAGTAGCAACATC

CCATAGCACCACAGTTCGATCAGCACTa

>contig00754 length=389 numreads=13

AACCTTAAaGAGACAACCtGGTttCtACGtGTACAACGTCaTCATGCCcAGTCTCGTAAT

AACTTTCTtCGCGCTGTTCATCTTCGCGTTCCcGCATGCTACTGGAGAgCGCATGGGGCT

GGCAATCCAGTGCTTCTtAaCGACCTCAATCTTGACCATGATGATTTCtGATCTcTTGCC

GATTGACTCGAGCGTTAcACCTCTGCTCGGATCGTTCATGTtAAGCAGTATGGCACTCAT

GACATTGTCtATTTTTGCAATACTGCGGTTTTAaCTTGCTGCGAAGCACACCGATGCCCG

GCTGGCTGCACGACCTTGCTTTCGATCGCATTGCCCcTCTGGTGATGTGGAAGTCTTGCC

TTGCTCACTGGCAGAAGAGAGGCTCTTGc

>contig00755 length=243 numreads=1

gtcgggctgactatcgaagcaaataaatcgttcagatggcaaggtacacgacaagcagat

cgaaccgatagcccttgcgcgctctcctgccgaatacgtagatcagatacgcatttcccg

ttatgccgatgacaaacacgagaagaaagccagccatcaaaactatcttgcgccaatcac

acagatggcaagcaggtaatgacgagtttcttggagtctcagtagagttttctgtcgagt

tag

>contig00756 length=648 numreads=15

aGCCATGCCAACAGAGGCTAGTGACGCCGCAATGGCGTGGGCAGTTCCACTCAAGTGCGG

AGGAGCATTTtCCATAACAACCAGTCCATAGAGTGTCTGTGGACCATAGAGCAAAAATCC

CATAACGAAAACAAGTCCATTAATAGATGCCAAGCTAGCCATGACCTTTTCTTGTATTGC

ATAAaCTACTAGCAGCAAAGGgAaTGTGTAAAgAaGAATGAGAACAaCTCGAGGACTGGT

ACGCCATAAAGAaCCATACTGaGCGAcTaGCcAATCGctGGCGTAACCAGaCACAACCGT

gCCCAcGAATCCACcGATCTGAAGtAtCATGACAGCATAGCTGGCTTCAaGTTGACTGAA

GGATATTtCTCCCATTAGATACAGTTGCATCCAATCCTCAaGACCACTTTtCAAAAaGCA

TCCAaGAAAaTaCGAGAAaCTCGTCAGCCATAAAAaaCTaCTACCAAGAACATCCCTCAT

cGAACCAATGTCTTGGTTTtGCGacGAATGGCTAAcgTCCCCATCTTTCTTGGATCCGCC

GGACCTCGTTAGCAAAGAGGGGTCGtCAACATCACTTggCTCGCGCACCAACCCAAGGaG

AAGcAGTGAAAAAgTGCAAGcAAaGATGCCAACGGCAACAAACCCAAg

>contig00757 length=241 numreads=2

TTTTAAATTACTGCAGATCAGCTGAAggAATAGGACGATAACATCTCTTCATATTAATAA

AGCAAGGCAGAAGAGGATTGCTTGAGGATCATCACAAACCATGTCGCGAAGGAATCTTCG

AATAGAGTCCTGGCAGAGGAAAGTAATCGAGGATCGTCAAGAAGAGTGAAGTGGTTGGGT

AGGCACCTTGTGCTAACAGCAATTAGGCAGAGAACCGTCATAGCAAGACAtaatttaaaa

a

>contig00758 length=220 numreads=3

cGTAAaCTAGAAACGCCACCGGAAGTCCTAAGTGCAGAGCATCCTCTGTTCGTGCTAGCG

ACTaGgCaTCCCtCTgtcGTTgCcATCGGGaTAtGATAcGGCACGCCGTCcAACAACagC

GgCCcAGCAATGCCGACAGGGATAGGAATATAGCCGACGACATTCTCGCAGCACGTGCCG

AACACCTTTTCATAGTCGTAATGAGCGTACGGAATCTCGt

>contig00759 length=615 numreads=37

CCCCCAAGCcAAACaGATGACCACAATAcTTGTCGTCATCGATGTTATCTTCAAAGAACA

TCTCTCGCATCTTGAAGAAAGCAGCCCCTGTAAGTAGACTATCACTTCCGGCTTGGTGCT

GTGGTCCAATGCGCTCCAACTCCAGAAGCTCTGACACTTCTTGCAGACCTCCTTTTAAAC

TCTTGCAACTTTTCATGAGGTATTTTACGTCGTAGATTTtCGGAAAAAATATACGCAAGA

GCTCAAAGAACTCAGATTCTTCCGAGGGTAGAGCTTCGTTCGTCAGAATTTTtAGAAGAT

ATCCAAAATCGTAGCCACTGTGGAATGATAACCAACTGATGTCCTCGGTAAGGACAATAC

CCGACGTGTAGAGCAGCTCCGCAAAGTGCAAAACATCAACACCcTCTTCcTCGTGTCCCT

TGAACTGGATACCAGAACCCATTAGCAGATCGATTGAATCTTGAGCATACATGTCTTCCC

CAAGATTGAATCTAAAATTAAATTGCCACGTTGAACCACCAGGAGGCTGTTCACCCTTGT

CATTATAGAAAGTGAAACCAATTTGTATAATCTTCAGTAAGTCAACATTGCATCTGAGCA

GCTGGTATTGATaGT

>contig00760 length=186 numreads=4

tACTTGACTGACTTTATTCTGAAATCtaCGAAaTTTGTGgACCTTTGtGAtCTCAGTCAA

AaGATACTACCACTGCccGCcGTACAGCcTTaCTtACATGAtGTTGAAAAGACTGaTCTA

GCGGACAgaGGgTTGAgCcTATTCGTAAaTCGCGTGCTTGGGAAAAAGCTTAACAAGGCA

TACCAG

>contig00761 length=636 numreads=19

ggTTGGCAGCaTATGCAATATCTAAGGAGAtCCTTATTATTCATGAAGAGACTCTGTTGG

CGGTCGTCATGGCTGGTACAATGATGTGGCTCTCAaaaaaGATTGGCCCATCTGTAGGCA

AGATGCTGGATGCTCGATCAAATGAAATCTTGGAGAACATGAACAAAGGGCGTGTCGCTC

AGATGAATCAAATCGAGTCCGATATGGAGCGCGAACGCAATTTGGAGGCCACGCTGGCCA

CTCGCAAAGaTTTATTTGAGATCGTCaGGgAAACgCGAGATGCGACTCGAGGCGGAgTAt

CGCAGACGATTATtAGAAGTGGAACAGGaaGTACAAAAACGCCtGGACtACCAAATCGAC

TTGCAAAATCTCCAGCGCAAGATCGAGCAGCAGCacATTGCCAACTGGATCGAGCAACAG

GTCATCAAGAGCATCACTCCGCAACAGGAAAAAGAATGCGAtCGCCcAGTGCATTGCTgA

CaTCaGCacGaTGGcGgCGGCTCGGCCTGTATAAAGGCATAACTAAtttttcttgattta

agaagccttggctttctgatgtcttgattgcttgcccgatttgtgtattatttagaggta

tgtttagttatggcgtccgaaaagacaataaaggta

>contig00762 length=370 numreads=3

cagctgtgcgctctcccttggacgtcagcatgaggtcctattattgggcacaccgccgtg

gcccggtatccatccatgtaggtcgcgctaaccttgtaatctccagaagggggctgacct

cgcgctcccgtgactttcacaaacgactcacgacccccgtcacctttattgcgcagcgtt

tccagggtgacctcggtgaaatcacaagctacatccggCAGCAAGTAACTCTTGGGATCA

CCGATCTCGTACACAAgctgctcagacacagttccagtactaacgagtccaccagtgttc

ggcggcttcagaacaaTGAAGCTGCCATCAGAAGTgCACTCCACAATAGGGAAACCGATA

TTGTCCCAGt

>contig00763 length=205 numreads=11

cttttatactggatttcattCGTTTCAATATTGGTTTCTTCTCCAGTGAGATGTAACttt

GTAGTCCATGTGGCAGGTAAGGCTACAATTGCTCTTGTAAGAATGTCAGGTACCTTTGCA

AGTTTcGgACTTtCCTCAATCCATGTATGGGgAACcGAGTCAAaGGCTTTTtGGTAATCT

AaCCATGTACAAAaTATATCAcGCc

>contig00764 length=218 numreads=2

ccACACCTGCATTGGGGCGTAGTGAAATCTCTGCTGAAGAATCTCCAGGCTTCTGGGTAG

TTTTCGTAGTTTGATTCACAGGAGCTACCCCTGGAGGGGGAGCTGGATCTGACATTTTCT

GTGGCGGTGTCTGTCGGTCTTTGGCACCCCATCCTTGGCTTCCTGTTGGTACAAGGGCTA

TTCGTGGATCGTTACCAAGATTTTCTTTTTTTAGTGAA

>contig00765 length=241 numreads=1

aagcagtggtatcaacgcagagtacgcggggacggaaggtctcttttgttaaagatctgt

tgagagactaagcaaaggaaaagatagtgtgaatgtaattatgaaacttagcagcgctga

cggtggatctgtgggagaaacgttccaattggctgttgacttggtgaggtgtcttcccca

aagtggtccttttcaaccttcatacagtgacgcagcaaaggtctacagttacttcaagca

a

>contig00766 length=879 numreads=20

ccGTCGATAGTCTCTAAAaTtCCAGCGGGATATGTATCTAATCtGGATCAATTTATATCA

AaGCTTCCAAAaGAAaGTGAATCGTTtCTTCCtATGGGCAGCAAGCTTCATCAATACTCG

AATTCGAATCGTGATtGTGACTACGAAATTTACcAGGCAGACATAAACGTACCTCGACTG

AAaGAATaTCACGAACGGCTGCAGACGTTTCTGTTGTGGTACATCGATGCGGCCTCCTAC

ATCGATGTGGATGACGAGAAATGGAATTTCTTTCTGTTGTTTGAAAAAAAGAGAAGTCCG

GCAAGTGACGTGTACAGCATTGTGGGCTATGTTACTGTTTATCACTACTATGCCTATCCT

GCAAGTTTCAGACCcAGAGTGAGTCAAATGCTGATATTGCCACCCTATCAAAAGAAAGgA

CACGGAGCGGAGTTGTTGGAAGTTGTAAGCAAGTACTATATCGCCAATCCTAAGGCTGTA

GACATAACAGTGGAAGATCCATCGGAAGATTTCGCAACTATTAGAGATTTTGTTGACTGT

CGAGGCTGCGCTGCGCTGGATTGCTTTCATAAGAaGAATCTTCTCGCTGGTTTTTCTGGA

GAAaTGGAGAGGATGGCCTtcGAGAAAAGgAAaaTCACCAAAAAGCAaGCTCGCCGAGTA

TACGAAATACTtCGTCTCCAAgCGACCAaCAtGCAaGATTTTGACTCTTACCGAATGTAC

CGCCTGGACGTAAAGCGAAGATTGAACATGCCATATCAGAAGCAAGCtCGTGATTTGAAA

AAACTTcGGCAAgCACTttCACCaGAaGAGCAaCAGGCTGCTATGATGGGAAACTCACCC

CaGGAAAGGATACACAGGCTAGATGAGGCTTACAAAGaa

>contig00767 length=123 numreads=1

gatttgggtgcttccaccgtgctgtgtatactgcttcaacggatagcagagaggcgaagc

gcaagtgcgtacgtagacattcgtgatgatcgatcaacactgcatgaatcagtaaggaag

tgg

>contig00768 length=1021 numreads=33

AAAGTCAATGAGGACGAAGTATCTAAGGCTCTTATTCTCGGATGGTGCGTaGAACTGCTt

CAGgCTGtGTTtctCGTTgCTGATgACATtATGGATCAGTCagAACTAcGCCGCGGACAG

CGATGCTGGTACAAGGAGTGTGGCTTGGTGGCcGTCAaCGACACGTTTCTCATGGAAGCT

TGCATCTACAAGTTaCTTCGCAAGCACTTTTCcGACACGCCCTATTACTTATCGGTAGTC

GAAGCCTTCCACGAAaTtaCATACATAACTGCGATGGGACAGGGGCTTGACATGCTTGTG

TCTGACCCCACTAGGgAaCTCGATTTATCAAaGTTTaCTATTGAGAaGTACAAGTCAaTT

GTGAaGTACAAGACTGCGTATTATTCcTTTTACTTGCCTATATCACTAGGCATGCGAATG

gCGGGTGTAAGCGATGAAAAGCAGTTTGAAAAAGCCAGGACtGTTTTAATGGAGCTCGGG

GAGTACTTTCAAATTCAAGACGATTACTTGGATTGCTACGGTGATCCGGCAGTGACTGGA

AAGATTGGCACTGATATTCAAGACGGAAaGTGTTCGTGGTTGATTGTGAAAGCCCTTGAG

AAGTGCTCGGTAGAGGACGAGCAAGTTTTGCgtaGGAATTACGGTAAGCACAGcGAGGAT

TGCATCGCAGAAGTGAAGTCGGCGTATGTTCGACTGAGGCTGGAAGAAGAGTTCAGCATT

TACGAAGAGCAGAGTCGCCAGCGCATCTTGGATCTGATTGATAGTCAGGCGAACGGACTt

CCGAAAGGCTTGTTTAtCCAGCTGGTGAAaGAAAATATaCAAGCGcgaaaaaTAAAAGTG

CACGTGGTGATGGCTTggCGTAggCAAGgTTtGGGAAAggATTTTgtCTTGtGACGaGTT

GAGCAAGCGGCTATACATCAGaTGACATTGCtgTAGATgTTGtgCGTTGCcAGAGCGCGa

GTACGTCACTGTTGAaGGAGCGCTACTTAATTTATTCGTCGAATTtATCGAGATCGgAAt

t

>contig00769 length=512 numreads=9

ccaaatgagaagatcttggagagtgagttcatcagatggtttgttagcagtattgctaca

ttGTCCAAAgCCCTGTCACCATCCGAAAGCAACTTATCCAATGCGCTGAAAACGAAAGTT

TCTTTACGCGAGCAGAAGGAGGTGACATCTTCTAAAAATGAAAAAGTTAAGCGTGATGCC

GATGTGCTTGAAGACACCGGATGGAAACCTTGGCACCACATCTGGCCAAAGGAGAGTTCA

AaGTCTACAAGTGTGCCTATTTTGAATGTAGCTGGGAAGTATGCTGTGAAACTGTTCTGG

ATGGGTTGCTGGCGCAAAATAATTATCGATGATTCCATTCCATGCAACCAGGAAGGTTcT

CCTCTACTGCcTTTtGCtGGGcGACCGAATGAgCTGTGGCCAGCTTtACTATGCAAGgCc

TTGATGAaGATtGCCTCTCTCGATTACCcAagTGGgAGTCCCCATAGCGAGTtCGGTgAa

CTTAGCATCATCcAGTGTTtgACTGGATGgAt

>contig00770 length=582 numreads=8

ATTACTTCTAGAATCGATATTCGCAAAGATGCCGAGCTCCTCAGATTTCAGTACGAATCT

GGAAAGCTGAAAGACGTATCCGATGAACCGACTTTGTTTAAAACGGTTCGCCAGTTAATT

GAGGAAGGCCACGTGCAGTCGTTTCAGAGGTTTTTACACCGGGAGAGGgtGCTgAATGAG

ACGCATAGgCTGAAGAAACaGTTAGACcAAGTCAATAAAGAAataGCTAGAAAAGTAGCT

GCGAAGTTCGGCGGTGATGCTGAaGACGTTACAGTGaTAaGgCGATCACTGcACTTGCAG

CAAGAGCACGCCTCCCTAtCGGAGGCGGtCAGTTCAgTTCGGCAaCAAATGGAAGATTTG

CACGTGGACCGAAAaGCGCGCGCGGACGCCATGGACGCACTGGTACTgAaGTCCGAGAGA

atACGTAaCTTTGAGGAACTGGTGCAGTCcAAACAGACGTTAaTTCGCAaCTTGATTAaG

GACAaCGGgCATGCCCGgCTCAAaCACgACCAaGAGTtCCACcAGTTGaTtGAGTCGACG

GAAGGgAAaCTTgaaagaaccgaatcggagatccgtaccctc

>contig00771 length=400 numreads=4

aCTAAGACAAATACACAGATAGAAGTAAGTTTGGCAAAAGATCAAAGCCTTACAATTATG

GTGTCTGGCAAAAATGATGCTGTAATGTTGGCAAGAAGTCAAATTCTGAGAGCTTTGCAA

ACTCAAGGTAGTGTTGAAATGGAAATTCCTCATGATCATCaTCGaTTTATTTTGGGCAAA

GGGGGCAAGAAATTGCAAGAACTGGAGCTGGCCACTGccAaCAAAAATAAccATACCTCG

AGattcagacatcattcgtattgttggctcaaaagaaggcattgacagggctcgtcacga

aattcaggttatatctgatgagcaggcaaaattggCATTTGaaggTGAACATTCCAAAGT

TGTACCACCCATTCATTTGTGGTCCAAATAATGCAACTGC

>contig00772 length=236 numreads=1

aagcagtggtatcaacgcagagtgcgcgggatccgaggtgatggctattggtggtgacgt

tatttcaaaagaagatacccctgttagggctgcacatatcactacagctccctctgtagg

tgatgttgctattggcgtgttttcaaccgagctcagtccacatggcgttgttgctggatt

gttactagacgtcagaacaacgtgatttcaagtcctattgcgactggaggtgtcca

>contig00773 length=241 numreads=3

TGGACAtGTTTATAAACATCTGTtGTGCATGTTTTtGTTCTAGGTGGTTATACAAAaTTT

tGgCGgAtGCCAaTTAAGATAGTTAAaGGATGTGgTTTAaaCGCATtGTTAGTGCACCAG

CTTCTCTGTCtGTCAGGAaCTtCAGTtATTATTCCCATTTtATCTTTAGAAGTCTTCAAT

AATTAGTGTATTTGATACTTTTACTGTGTCTGGGAAACTATTTCTAGTATCCAAgaaagc

c

>contig00774 length=240 numreads=6

TATAaTATCAGCATCAGCtATTCGCGTCAAGGCAACTCGAATAaCCCTGCAATGTAGAGG

ATGCAaGAACACAATACcGAACgTTaTTTGAAGCCAGGTCTGGaGggTTTtGCAATGCCA

CGATCTTGTCCGACAGATcaggctggtaaaattccatgccctgtggatccgtttttcatt

cttcctgataagtgcaagtgtgttgattttcaggttttaaagcttcaggagtccccagat

>contig00775 length=172 numreads=5

GAAAACCAgCGAGTtCCGAAaCGACAGTACGACAGACcAGAGTGTAGGCACAGACCCTCT

GCAAcAAGAAGATTCAATCGAGTGGAGACCTTGCATTGAaTAttCTcaaacttcgtgccc

aaattgattgaacacgatcaggtcaatgaagagcaaatcaggaaggattatg

>contig00776 length=136 numreads=1

tggacactgcgtttgcgcagcatggttcgcatttatgctaatggtttaataaagacggag

atatcgatgtgtgcttcgttgaggcattataattttggagtcatttttttaaggctagat

gtttgcttattgtagg

>contig00777 length=205 numreads=3

cattgttcaccgcaacgccagtttcctcatttggaacgcaagtggTAACTTCATCACACT

TTTTGTcaTCTACtGcAAAATCTTCTTTTACGCCTTCGCCTACTCCTTCATCGAGGGTTC

CAATTGTTTCTACATCTGCGGGATGTCCTGCTTGCTACCATAAGGGCTCATTCTATTCGG

TGAATGAGACGTGGCAGGACGAGAc

>contig00778 length=624 numreads=8

aaaaTGCCCAACGATACAGACGTGACACGACATTAGGAAGAGGAAAAAAGACCGTGGCCG

AGAGTAGCGTAAACGTACTGCCTGTAAACGTAACGTACAAAGCCGAAGATTTGCGTCGCA

GCTTAGCCGTAAAGgCGTCAcGAAGCATCGAGTCTAGCGAAAGCATTAGgacAACCATAT

ACAgTGGGCAATtGgTAATGTCCGAAACCATTACAggAATCAATACAGCTCCTACCAAAA

ACTTGGAGAGcAGTTtGTGGACGAcTAATGCGTCAGCTGAAGTCAATGTCACTACCGACG

CTTcTAAaCcAAGgCcGACTgAaCTCcTaTCCACGACAaCtCGAGcACcGgACcACGATA

GCaGCGGTAACTaCGCcGACATCgTCGGCACGAGAAAGTACATTTCATGCTCATTATGTC

ACGTGGCGCAGGCATcTACGCAaGTAATGATCCCGTcTGGgACGAACTGTCCCgCcGGGT

GGCgCcGTGAATATCACGGCTTTCtCATGACATTtCATCATcTCaCCGCCCAGTCGgAAc

TGATCTGCGtGGATTATCGGGCGCTtGgACTTGAGCCGGGCGAAGACCAAGGACTAAAAg

CATACTGCGAAAGTACATGACTCa

>contig00779 length=243 numreads=1

aagcagtggtatcaacgcagagtacgtcgggaataaattgtttgcggatctgcggaatat

taagcttggaagtttaagggtaacattttgattgggagttgagtgtaagtcttaatgtga

ctgatggggctgctcgcttgggagaggatggcagcagtgctggcttgttcctgaccctgc

agggagtggggttaaataaagcagaggcttttctttttgtttagaggaactgattcttgc

tat

>contig00780 length=325 numreads=5

tCACAGCTTCACCAACTCCATCCTTCCTCTCAGGAGTGAACATATAAACTCTGCGATTAT

ATTCAACAAGAAAAAACGgATCCTCGcGAGGTGGCAAATAATCAAAAAtGGtGAGCAACC

TCcTAGAATCAACGGCATAaCCcTtaTGCCCCGatGGtAACGAaCGCACCTCcATTACGg

AGAAGCTTCTAAGgTCAATGCTGaTGaTAgCGgTAAATTCtgAAGGGgaTCgcAaGtCAC

cATTAAACaacaGATGtAtGCAATCCCCGAGCAAATGCAGGACGTTAGCaagggcagaga

ctcacgagcatgaaggtttccgaag

>contig00781 length=1205 numreads=50

ttttttttttttGGGTtaGcaTcTTTTtCCGtAAGGGCGAAGTGAGaTACAGATAACaCc

GCaCTTcATAAAaaTtAGAAaGaGTTCATAAACATATcATTAGCATATTCCAAGCAAGCA

AAGCAACTCGGACGAAGAATCGATGACaTTTTTAAATCTTCAGCAtAATTTTCCTTTTAA

TTTtCGAGATCACTTtGCAAATCtATTCGTtGGATATTCATCAGGGTAtttcttGACGTG

CTCCATGCATTCGTCGTACTGGCtCTTGATGCgAGGGGTCCAGTTCTTGTACACATcGgT

GAACATGTCCTTGATtGGGGGCTTCAatCCTTCTTCAGCGgCTTTGAAGGCAGCCATAAC

TTGCTGGAGAGCTtCTTTTCTCCAATCGTCGTTCTCCTTGTCCGTCCACCAACcTtCAAG

CTCCAAGTAACTTCTGAGTCTATTAATCGGCGAATCCACGTCCTTCCAGTACTTCACCTC

TTTGGCTGACCGATACGCGGACGAATCATCGCTTGTGCTGTGATGACCCACTCTGTAAGT

CATGGCTTCGATCATGACAGGCCGCGATTCTTGGACGCAGATTTCACGGGCCTTCTTCGT

AGCGTTGTGCACAGCAAGGACATCGTTCCCGTCCACTCGAATAGCAGGCATACCGTACGC

TGGACCGCGACCAGCTATGCCATCTCCCCTGTACTGCTCAACAaCCGGTGTAGAGATGGC

ATATCCGTTATTGCGGCAGAaGAAAATTACCGGACAGTCGAGAGTGGCAGCGAAGTTGAA

CGCAGCATGGGCaTCCCCTTCACTGGCGGCGCCATCACCAAAGTAGCAAATCACACAGTT

ATTCGTCTTCTTGTgCTTAAGAGCGTAAGCAGAGCCAGCGGCTTGAGGCATTTGCGTGGC

CAATGTTGAAGATATGGTCACAAaGTTGTGCACCTTCGAACCATAGTGAACAGGCATTTG

TCGACCACGCCCCAGATCGTAGTCATTTCCGTAACACTGGTTCATGAACGACTGCAATGG

GTATCCCCGCCACATtAGCACCCTTGTtCCCTGTATTGCCCGTATATCAGATCAGCCTCT

TCTAGAGCAGTGGCTGTTCCAAaGTGGGTTgCTTCTTCCCcATAGCAGgTCATGTAAAAT

GATATACGgCCCTgACGCTGAGACTCATaTAaGAtCTTATCcaTAGTaTTCaGAAgAACC

ATTTg

>contig00782 length=178 numreads=1

aaactctcatcagagtagctcggtcaacaacgtactttctgctgattgtgacaaatataa

acagcagcgtataaaaggaaaaattgcaaagataaagcaagggaagttgagttttccgaa

cagccttcgatgcagaacagcatccattcatcgaattctgacgcagcgtatatagaat

>contig00783 length=1001 numreads=33

aGTCTCACTTCTCTTCATTCTCTGGGAGGATTGGCCCCTGTTTGACTTTCTGGCCTTTGT

TCATCCAAACTTTGCATTAAACGTCATTAATAAATAGGGCCTTGTCTATCGAATCTGGCA

ACTGCTCTAAAgTtGtAaCACCTCtGCAagAAcGCTGATGCTCTTTGATttCAGAAAAGC

CCTtACATTTCTTCCCGcAGaTAaaCGTTGtgAcgAACtgAgCGCtGtGAGCAGgAaaTT

CTGATTCAatGCGATAGgTGAaGTCTGacGAGGgTGCAtCcTTGCTGAGCTTGTATTTGC

TAATGCCATGTATGCTGACTAAGTCTTGCTGTTtATGCAaTCTTTGATTGATGAATACAT

TAACCATGAAAAACCGCTTGAAAGGCAACAAATGCAACACTTtCcATGATCTCAAAAAAA

TCTTTTtGCATGGATCcATCCACCAATCAGCAAATCAACTCTCATAaataGTTAACCCAA

CAAAATTTTTTGCTAGAACATTAGAGGTCGGTAGTGGCATCCATTTGCCTCCCCAATTAA

GATTCAAGTGTACCTACATAATTTtCAAaCTTCAAAATGTCTTTGCTCTAGGTTTACACT

AGTAGACTGCAAATCTCACTTTTGATCAGgCATGACTTGCGGTtGACAAGCAGCCTTTTT

aGCAGCTTCATCAGGAGAAGTTTTTCATTCCAGGCTTttGTCTGCAGAAAGTATAGTATG

TTGCTCAAATACAAATTCtaTTTTTtGCAGGTGCTTTTCGCTTTTTCAGATTTGaatGgT

GGACCTGCCcATATGCGCTCTTGAGCTCCTTCTCTAAGATGTAATGTTTTTCAGACTTTC

AAATcTTGTGTTtCAGGTTtAGCCTCAAAaCTGGTACTAAGTTCCAACAAAAaCGATTCC

CAGCgTTGCTGAACAAGGTCTGGTCGTTGAATTGCGCTTGTTATCAAATCTGTTtATCAA

aGtGTTgTtGAgTCTGGgTaCATTTTGAGACcTTTAACTGT

>contig00784 length=382 numreads=8

ccACTTTCtCCACTcGAgATAGCTGGACTCACTTAaTtCGCAaTCtGCTGATTtaCAGAC

AAAaGAAATAAaTGgATCTTCCCTtcTAATATtAGAAaTTGTtACTaTAAAaCTTACGAa

GCCCAAaaaCTTtACATCCAGAGTTCGAGAAGTCACGttAAGCTAAAGATGACTATCGAA

CAAGTAAAATAAATAAGGAATCTCAACTTTAAACTCAGCTTGGAAGCCCACGCAGATCGC

ATTTTTTGAGTTGAAATGAAAATAAACAGTCTAaCTAAaTAATCACAAATCCGTCTGTAA

GCTGTTcACTAATACACGTAGCAATAaTTTCtGAGACGAAGCAAAATaCTGGAAAGAaCG

TCCGGAACAAATTCTGCCTTAC

>contig00785 length=143 numreads=7

TTCgAcAAaTGAAaCGGTAACAAAaTaCTTCTTAAGATCGGCAGCATCAGCAATAACATT

AAAGAGAGCTTCAGACATCATACACAAAAGACCACTCTCTTCTGCAGTGCCCATTATAGT

TTCGCTCTTGCCCGAACCCGTCT

>contig00786 length=243 numreads=1

ccagacgttgaggtaggttcgaggatatgtaagtaatcgggtttagaggttccttatgtt

cgtctcagaaaccaatggatgggtaaaagccggaatcgttctgcgtccggtgtattaaat

ataactttttctttcttcgggcgctatgtcgcgacaaaagacatccctttggcaggcttg

aaccttcaaggaaatgcctgccttcgaatgacgagaatggagtctgacacaactgggcat

cta

>contig00787 length=854 numreads=35

aaGCGGGTGAAGATTGAAGCTGTTAGCtttGACTGGAAGCTTGCACATTTCAGAGAACGT

GCTTATCAAATtACTAATATCAAGCCATGTGACCAAGATGTCATATTTAtGGGACAATGT

TTGGAAGATGATGGCAAAAGTCTGCATTCTTATGGTGTTAAGAATAGCTTGACAATTTAT

GTGATAAAAAAAGCTCCATCAGACAGTGAAGGGCCTTCAGAACATGTGGATTTTGAAATG

CTTAACAAAGTTCTTGCATATGCCATTCAAAATCCTCTACACAAACACTcTcTGAAAAAG

GCCTTGCGTGACAcGcGAAGGATCGAAAAaTtGATCGCATCTACCCCAGGAATCAAGaCT

GATCCTGTTGCCCTGGGTATGCTTAAAGACCCAGAGTTGTTCTGGCTGATTTCTGAAaCT

GTAAATATAGAAAGAATTGTTAAAGAGCACCCTTACCTGGCCATCGTAGCGCAAAAGGCT

CTGGCAGATGTTACCAGTGATCCGTCTGTGAGCTCTCAGTTGCTGCGcGAAGctCGCGAA

GAAGACATCGATTATCCGGGTGTTGATCCGGCTGTGCTTGCTCAAGCAGAATTGTCAGGC

CAAGGGGCAGCTGCTGCGCAGTCCTCGTCTACCGAATCTAACCCATCTACATCTGGTCAA

GCTAGCGATCGTCAGAGAATATCGCAGACTGATCTTGCTAACGCCCTATCGTTTGCGCGC

ATGGgTCTtCcGCAAaaCATCGCTGGAGGTGATGCTGGCAGCAgCACCGGGGGTCaGaCA

TCATCTGCAAaCCaGgAGgAAGcGCTTCAGCAGATGCGTGACATGGGTATACATGACACG

GCGTTGAGTCGACg

>contig00788 length=580 numreads=9

gCATCCACCATaaaTGATATCGCTATAACTATAGCTAATCATCACTATATTCCAAGGCGA

TTAGCTCGTGTTTTCCAATGAAGCCTCGAGGTATGAGCCTGGAGTGAATTTGACgacacc

gcgattccgttccttccACcGCcAAAgCTAGAGCGATCATTGCGACTGCATTCGAGGTAC

ATTAAAACTTCGTCGAAAaCTCCGTCACGTTCATATAAaCaTTTTTGTcGCCctCaTGCC

AGCTAGAGcGAAaTcTGTCGTTCTTTTtAAaCTGTTTCTTCTtCgcAACGGCAACaGGGT

GATCCGaGCGtCGCAGGTtGAAaGGACTTCCCTTACCTGCCGgCcGGGCAAAGTTCtCAG

CGACAGCCCAaCGCAAATTCATTACcGATTTCGGTGACAGCGTGAAaGCCtCCTGATAGA

TACGCGTAATGGCGGCGTAGCAAGAATCAGTGACGGACTTTGgTAGCAACACATccGACg

CaGAcaTCACTTCctCCAAAaTAGGAACAAGGGTCACATTAGACCTTgAGAgCTTAAGCC

GCGTGTAGCCCCCGCGTACTcTGCGTTgATACCACTGCTT

>contig00789 length=638 numreads=14

AAGGGCTCATCACcTTgtCTGGTTCACACTcTCCGCTTCGAAAGTATCGTTtCCATtATG

TTTCAGgCCTCGgATTCGGTCTCATGGGTGGTCTGTTTGCTATGGTGAATATTCTCGCAG

AAGTCACTGGACCAGGAACCATCGGACtctATGGGGATTATGGAGGCCTAGTCCTCGCGT

CcGCCTGCTTGACGAACTGCTTCATTCTGTTGCATACGTTTTGGGGCGTGGTTTtCTATG

ACGGGTTGGACTACAAGAAATGgaTGCAGGTGGCGTCcGTGGTGCTGTCTCACATGCTGG

TATCCGGACTGACCCTGCTAAACAaGAGCGTATCGATATGGCCTTCGCTGGTTGTTGGTT

ACTTCCTTGTCGTTGTCATGGCCGTGTGGAGTTTCCGGATTGTCGgTGGTAGCTtGGgCA

ATATCGGTCGCTCATGGaGAAaGCaGTtGGTtCAGGaGTCCTGATAAaCTTTTCtGGTGT

GgAGTTGGTTCAaGCCTTCCTACGTATaCCAAAGTTAagCAGAaGGGaaCaGATagcGGT

CATTGACTGCAATGACCGTAGCATggcctttgtaaaccatgattttgcataattagcaca

tctcccatatgcagtaagctgtacgcacgattgacata

>contig00790 length=239 numreads=1

gtggtataagtcacttgcctttgcgactccattcactcctgggtttcgctcaaggaagta

gctcggtaccaaattcgccttgtcattcttgaagaagagccatttatgaggatcgaggaa

gctcctcgtatcttcgctaacgttgactttcccctcgacacttcccgactccattttatt

agccacagagacggttccaccaaacaggcaataccgaggcattttcgaccaacaacccc

>contig00791 length=241 numreads=2

AAGCAGTGGTATCAACGCAGAGTACGCGGGCATGCTAAAACAGCAACAAGGAGTCCTAAG

GGcAGGACCAAACCCCGGCTTGTTTTTGTTTTATCATCCAACAAACTTTTtGCACTTGTT

TAAGGGTGATATGGAATTCGAAACTACATAGGTTTTTGGCTATGAACGATTCTTCACTCT

AATGAGCTATCCTAAGTTGCTTTGATGTAGATTCCCacAGGTTTctgaccgacatgttgt

g

>contig00792 length=248 numreads=4

AAGCAGTGGTATCAACGCAGAGTACGCGGGGGCCcAAATGACTAAGGTAGCGTCCTTGCG

TAGCGTTTTTTGTGTGTTTTTAtGTGTATGCGTGTGTGcGTGTGTGTGTCATTTGTGTGT

TTTGCGAGTATTGATCCtGAGCTtGTTTCtGCTCGTACACAACgTAGGATATGTTTAAga

agacgaaattgttacaattgtgtgctgagttttctcgatgctcctgtttagatgcggctg

tggtcgct

>contig00793 length=239 numreads=1

aagcagtggtatcaacgcagagtacgcgggtgactacttatttattaatttgttattttt

gttcgcaagtcccaaacccattctcgctttcaattgtatacgctgaagaagaatgcctgc

tctaatttaggcgtcacctatcactccctttctcagccatgtcatatcttgatacagtgc

atgctggcaaactagttgctttctcccaatttaattcacccttgtttatttacctactt

>contig00794 length=265 numreads=7

ACCcGAATCCAATCATGTCAGGAAGAACAATTCGACCAAAAAATCGTtGCAACGGTTTGA

TAAGCTTTCTCCAAtCATAGCTGCTTGTAGGAAAGCCATGAAGAATCAATAGGACGGGCG

AAGCCCTAaCCGgCTcTAaCGTGTGTATATCTTGAAAAAATaTTTtGtGGCCTCGGTAAa

TAAaCGTGTAaCCACTGTCCATCCAAAaaGCTGgTctcTGCATGagTTGCGCtccatcga

attcgatgttacatattaagaagta

>contig00795 length=156 numreads=5

CTCTTGTGTTATAAGGgAGGACAAGGAAACTCTGTTTGGGGTCATGTCCATGGTCAGGAG

AACCCTGCGGACTTGCAGTCACGTGGTGTCACTCCCcAGGGagtCgTTAGGAGTAGACTA

AATTGGTTGGTTTCGACGGCCCAGAGGTGGTctttg

>contig00796 length=241 numreads=2

TATGGTCGCTTTGTATACAAGTGCCAGCGGCGAATCGGATAGCGTTTACGAGTGCTTAAA

GGCCTAaCCAAATCGCTGGAAAGCAGCAGCTGGCCAACGTCATTTGCAAAGACGCTTGGG

TGCCGTCGCACTACATTAGTTCGTACCTACCTCAGTCGGTGACGGTCACATTGGAAGTCA

AGGATCGAACCATTCCAGTTGCAGTCCAcaTCTCAAaCTGGAATgttgaaggtggaaatc

t

>contig00797 length=241 numreads=3

ttCATAAGGGGCAGTTTATTGTCAACACTAGAACGAACAAGCGAGTCAAGGTTCCTCGTT

TAGTACACGTGCATGCTGATGTTATGAAGGATATTtCGCAcTCgCcTGCCGgcgATATAT

GCGCCTTGTTtGGCGTGgACTGCTCTtCtGGAGACAcGTTCGTTTCtGAAGgTTCCCCCT

TGCTCGCTATGGAGTCGATTTACGTTCCCGATCCAGTGATTTCTTTGGCCATAGAACCTA

A

>contig00798 length=239 numreads=1

actgcgttgtgacacccaccgtggatgcggaccatcccacacgttggagcctcctaaaag

attcatgcgtgatggacgacacattcactatttctgagacttggtcaggtcaaaggatgg

gcttcgaggcgttcgcgttcacttctgattccagcgcttccctgtatcttcactgcaacc

tggtagcctgcctaccagacgcagcgtgcggagtttgcaacgctcgcaagcggcgatca

>contig00799 length=422 numreads=6

cTCATTCCACCAGTGATAATCTTGAAcATATGACTGACCAGCCCGgAGGCAACGCcTCCc

GTCATATAGAaTCCtATAAACTGcTCaGGACcAAGCAGgTGGTGTATCAGTGGAGCAAaa

CtCCACAAAACAACcAtATtGGCACCCAGGtGCcAAAaTTCGGCATGGCTAAAACAGGAG

AGAaGAagAGGAAGGgAGCGTCCTGAACcAGGaTTGGCAGCAAACCATTTCATAAGAAAG

GAACGACATCGAGGAGCTtGCCAGCACAAAAACACGgCAGTATTTAAAAAGATGAtGCCc

GCTgcAaTTttttCCccTCgTTTAaGcGCTGCcACCAAAGGTGcACCTtCattcTCcAAC

tgTATGCcTTTTCCACTtCCtgActtGgAaaccAACttGgctgaGCATAGCGACTTTCGA

GC

>contig00800 length=238 numreads=1

tctggcacacgggtgatacagaaggagaggtgaagctgttgtggcatgacaagaagtacg

taggctggaaagatcgcaccgcatatagatgggagcttcaacatcggccagtcagtggtc

ttatccggatggtgatgtacgagggcggcactcagatgtttgacactggctgtctgtatg

acaagacgatgcttggcgggaaagttggcctgtttgttttctctcaaaaggacgtcat

>contig00801 length=112 numreads=2

CAAAGAGGAGTCAATAGTAACCAGAGCTGAAGTGAAAGTATTTGACCACGGCAAGCTTAA

GCATGTCGAAACGGAGGAGAAAAACCCTCTGCCgaccggtggcaaccttgcg

>contig00802 length=238 numreads=6

TAAGGTGGAAGGTGgATCTGCCcTGAACAACTtGGTtACAaTGTGTCTGCGGCATGCAgT

GCcAaCTCTGGAaTcGTACCTTGACTATTCTAAGCAAAAGCAGAaGAAAGGaaGAAGGgC

GCcTCTGCcTTCTtCAAGCAAGCTATGGAAGACTGTCAAAGCAACAGTAAAGGTCTACCT

AATGGATAGTTtGCAGATGTTGCGgCaGTtgAcGGACgATtCcATGTTGAGtGTCATt

>contig00803 length=241 numreads=1

aacaggcacgaattgtctgcgggtactaccctgaaaggacgaataaacataaagactcag

atacttttagtcccagcaacccagagaccacagttcaaagtacagatgtggcgaagtttt

aagctggtgattaacaatagtaaacgaagtcggggacggacgaaaaattgatcggcgcag

acccaagacgtcggcacagacgaggcggccaattaaaaaactaaatagatttgacaaata

a

>contig00804 length=319 numreads=7

GGCACAGATGGATAAAATCTTCCTGAGAAAaTCCTTTAAACcGAAAGTAAAAGCAGCGTC

TCATGGACGGCAAGATTGCCAGAACGCAAGTGTTGGTGAGAGCTTCAGCATCCGaGACGA

GAATCGCTACCCAAATATTGTCTACAaGACAGCAAAATTGAAgCCAAaGTCAACGAAaGG

CGAAGGATTTTTCGGTTTTCTGAAAAGGAAAGCGAAAGGCTTGGAAAAgAATTCCAAGAa

TTCCAAGCGAAAGTAAGCCAACTGAAACGCACTCCTTTGCTCCACATGCTACATTGCAAT

GGGCTATGACTCTTCgACg

>contig00805 length=604 numreads=25

AAGCAGTGGTATCAACGCAGAGTACGCGGGGGATCTTTtAGTGACGGTATTGTTAGGTGA

CTTCTTACTTGCTGCtGTGATCGAGGCACGGACAAaGACTGGcAACCATGCAAAAGGATG

GCCcTCATAATGTCTCCAGATGcGCAAATgCCcGTCAtATTATATCGgCGTGGTGCAGTG

AATTtGACATtGTCATGGGATGAGGCTGAACTGGCAATGTTtGCGGGATCCTTTTTcGGc

acgttcgtggacacggtgtactacttctttgcttacagtgtggcgatcgctgcccctgtt

ctcactcaagacagcaccactgctgaatacttgggaggccGCGCTaGAaCCTTTATCGTG

TaCAAGCCCGGTGGATCCGTTGACTTCaTAGTCGaGATCAGcGACCTGAAATTCAgCgAT

AGCTTAACAaTAACTTTGCGAAAGATTaGAAAAaTCTCcGGAGGTCtcACGTACGGCGAt

ATTTCCACAGCTTACTTAGTCCTTGGCCCTCCTGAAGAATGTGGAGCTGGTTTGTACGCG

TCGTCTGTTGTCAAGGGAACAAGCTTCTACGCAAACGTCACACTCTGCGTTGATACCACT

GCTT

>contig00806 length=242 numreads=1

aagcagtggtatcaacgcagagttttgccaccaattctcactgtaattgctttgtcaact

gtacatatggactcatcaatatctcctacttcaactaccactggcagccattttgagaaa

ctggtgccaaaaatggcagaaaatagggtcagacagaaaacctcttttaagcaagaacag

gtcattgtcctaaaaataaaggcacataagaagctatataattgagaccttaattctact

gc

>contig00807 length=422 numreads=7

aTCAGCAGCTTTAGACTACTTCTCAACTGAGAAGGTCTCACGTTCTCAGGAGGAAAATTC

AGACTCGCCGAAGTGTGAGAAAATGGAAAGGACGATGGATCTTTCAAGTCCGGGAGAAAC

AACCTCTGATTCATtGCCGCTCTCTTGTTCTTCTAGTGACAaTTTtGAACTGCTGGATGA

AGACTTTTCTGTTCAGtCAGAGAAATTTtCCtGGGgTGGAAAATGGGATGATGATGATCA

GCTTCCATCAATTACTtCAAGTGCAGATGGCCTGGCAGCACCCATTTCGGAAGCTGACGT

CAATGATTTCTTGGACGCAGTTCTTAAAggGAAAGTCTCTCACTCAGCCCGGAATGGAAT

CAACTGCGAGATATTCAGATACAAGTAGTAAGCAATGCGTTGTCAGGGCAAAcatccaag

aa

>contig00808 length=241 numreads=1

aagcagtggtatcaacgcagagtacgcgggatgattgagggttattttaccaaaatctct

gtgggaaaatgctttctattggggaattaagacgttgcatgcactattaaagtaagagag

tgttgtcccgtgcgctgatggtcattaaggacatgcatgtctttgtcgaaagcatcaaat

ctccaaccttcgagcttaaaagatgtcaagtttgaacttttcactttgctgacaacgcgg

c

>contig00809 length=226 numreads=4

aaatCGGCTCCGACCAcATaGGGCCACcAATACGAAATaCatGgCcACACTCTCCACacG

TTtgCCcAACAACAGgACCcGTGGCAGGgTTGTAcTTTTTGcTATTACCCACTtCAaTTG

ATTTCCcGACGGGCtGCAAGTgAAACGTTTCACAACCGgAGCAgTgAAaCACGTACGATT

TCTTGCTGgCAGAAcGCTTCACTTCACTGGCaCTGGTGAAGACTcG

>contig00810 length=303 numreads=3

CATGCTTCAGaaaCAGCTAGACGAGCTCTCCGAAAGCGTCCAAGCGAAAGAGACGACGTT

GGAAGAGCTGCAGATAGAGCTGAGTAGAAAAAaggaTTTTgAgAaTAGGAGAAACAaCGG

CATCGATTTGAAaGCTgTgTCGGCATCCtGTCATGATTtAGtgCGAaGCAATGCGGAAAA

GgcGAGgCTATTATTGCACTGCAAgcccggttgctggcccggaacgatcatatggagcga

gcgaacaaactcatcgatgaaatgaagcgaaaaaacgagaacctgatgcagcaagtgcga

gat

>contig00811 length=812 numreads=17

ccGAGAaCAACAACCAAAAAGCAAACTCTAAATGACAGTCTTAGCAGTGCTCCAGTCCAT

ACTTCTAAGGAATTGGCTTCATATTTTCAGATGTATGATGAAGGACTTCACGATAAGCAT

TGGCCTGTATTTAATGCAGCTaaGAAGGTTGCTTCCCCTGTTCGTGTTCTTTtATCCGGG

ATCTGGgCGTCacaTAaCCCCATCTctGGTGTTCcGTGACGTCGTcTaCGtCGACtCGTT

TGCTCTAGTGGAGCCAACCTTCTCTGATTTGAAGGTTCTGGAGtGGGTCGCCTTGCATAA
[truncated: 4,591,761 more chars]
